# A revision of European species of the genus *Tetrastichus* Haliday (Hymenoptera: Eulophidae) using integrative taxonomy

**DOI:** 10.3897/BDJ.8.e59177

**Published:** 2020-12-16

**Authors:** Christer Hansson, Stefan Schmidt

**Affiliations:** 1 The Natural History Museum, London, United Kingdom The Natural History Museum London United Kingdom; 2 Biological Museum (Entomology), Lund University, Lund, Sweden Biological Museum (Entomology), Lund University Lund Sweden; 3 SNSB-Zoologische Staatssammlung München, Munich, Germany SNSB-Zoologische Staatssammlung München Munich Germany

**Keywords:** integrative taxonomy, DNA barcoding, new species, parasitoids, Tetrastichinae, taxonomic revision, morphology, parasitoid, gregarious endoparasitoids, distribution.

## Abstract

**Background:**

The European species of the genus *Tetrastichus* (Insecta, Hymenoptera, Eulophidae, Tetrastichinae) are revised with 93 species, including 50 species described as new. The revision was conducted using an integrative taxonomic approach, based on DNA barcoding in combination with morphological characters. The Tetrastichinae are a biologically diverse and species-rich group of parasitoid wasps with numerous complexes of morphologically often very similar species that attack a wide range of hosts in over 100 insect families in 10 different orders. The genus *Tetrastichus* is, with almost 500 described species, the third largest genus of Tetrastichinae. Although biological information is lacking for most species, current data indicate that *Tetrastichus* species are gregarious koinobiont endoparasitoids developing on juvenile stages of mainly holometabolous insects. Due to their host specificity, several species of *Tetrastichus* are used as biological control agents.

**New information:**

The European species of *Tetrastichus* Haliday (Hymenoptera: Eulophidae) are revised using a combination of externo-morphological and DNA barcoding data. This is the first integrative approach for any of the large genera of the Tetrastichinae. A total of 93 species are included, of which 50 are described as new: *T. agonus* **sp. n.**, *T. antonjanssoni* **sp. n.**, *T. argei* **sp. n.**, *T. argutus* **sp. n.**, *T. asilis* **sp. n.**, *T. ballotus* **sp. n.**, *T. bledius* **sp. n.**, *T. broncus* **sp. n.**, *T. calcarius* **sp. n.**, *T. calmius* **sp. n.**, *T. clisius* **sp. n.**, *T. cosidis* **sp. n.**, *T. cumulus* **sp. n.**, *T. cyprus* **sp. n.**, *T. delvarei* **sp. n.**, *T. doczkali* **sp. n.**, *T. elanus* **sp. n.**, *T. elodius* **sp. n.**, *T. ennis* **sp. n.**, *T. enodis* **sp. n.**, *T. erinus* **sp. n.**, *T. evexus* **sp. n.**, *T. fadus* **sp. n.**, *T. fenrisi* **sp. n.**, *T. flaccius* **sp. n.**, *T. gredius* **sp. n.**, *T. iasi* **sp. n.**, *T. illydris* **sp. n.**, *T. incanus* **sp. n.**, *T. inscitus* **sp. n.**, *T. intruitus* **sp. n.**, *T. johnnoyesi* **sp. n.**, *T. lacustrinus* **sp. n.**, *T. ladrus* **sp. n.**, *T. lanius* **sp. n.**, *T. lazius* **sp. n.**, *T. lixalius* **sp. n.**, *T. lycus* **sp. n.**, *T. marcusgrahami* **sp. n.**, *T. minius* **sp. n.**, *T. mixtus* **sp. n.**, *T. nataliedaleskeyae* **sp. n.**, *T. nymphae* **sp. n.**, *T. pixius* **sp. n.**, *T. scardiae* **sp. n.**, *T. splendens* **sp. n.**, *T. sti* **sp. n.**, *T. suecus* **sp. n.**, *T. tacitus* **sp. n.** and *T. tartus* **sp. n.** Two keys for the identification of species are presented, one for females and one for males. Based on DNA barcode sequences for 70 of the species, a Maximum Likelihood tree to assess phylogenetic relationships within the genus is presented. These 70 species are also characterised by a combination of CO1 and morphological data. The remaining 23 species, without a DNA barcode, are characterised by morphological data. Using a combination of data from the morphology and CO1 or morphological data only, the species are separated into three species groups (*clito*-, *hylotomarum*-, *murcia*-groups) with 41 unplaced species outside these groups. Hosts are known for 27 of the species and they are gregarious, koinobiont endoparasitoids on a wide range of immature stages of holometabolous insects and appear to be very host specific. The first host record for Lepidoptera (Tineidae) in Europe is included.

## Introduction

### Tetrastichinae

The Tetrastichinae (Hymenoptera: Eulophidae) are one of the largest groups of parasitoid Hymenoptera. They are, with currently about 1650 species in 91 genera, the largest subfamily of the family Eulophidae, that include 297 genera and about 4500 species (Noyes 2020). Species of tetrastichines are found throughout the world and they occur in most habitats on Earth. The majority of species has been described and recorded from the temperate parts and mainly from Europe, but very little is known about this group from tropical areas. Thus, the recorded number of species is very probably a minor part of the actual number. Apart from species richness, it is also one of the biologically most diverse groups of parasitoids and the range of tetrastichine hosts comprise over 100 insect families in 10 different orders and, in addition, spider eggs and even (through their galls) mites and nematodes are targeted (LaSalle 1994).

The subfamily is a group of particular importance, even within parasitoid wasps that are generally essential for maintaining biological diversity in terrestrial ecosystems (LaSalle and Gauld 1991, LaSalle 1994, LaSalle and Gauld 1993). It is extremely species-rich and has been used in numerous ecological, biological and behavioural studies and several species have been successfully used as biological control agents (see Graham (1987), LaSalle (1994) and references therein). Despite their importance, virtually all genera lack comprehensive taxonomic revisions and the identification of species from most geographic regions is hampered by the lack of identification tools. Furthermore, the classification of Tetrastichinae has been subject to major re-arrangements throughout the history of the subfamily since it was established by A. Förster (Förster 1856) and is treated in further detail below for the genus *Tetrastichus*.

Taxonomists have largely ignored tetrastichines because they are taxonomically extremely difficult due to a large number of species and their morphological similarity. Both factors have been a major obstacle for taxonomic revisions, based on morphological characteristics. For the present study, we chose an integrative taxonomic approach, based on DNA barcoding combined with features in the external morphology. Barcoding allows for sequencing large numbers of specimens at a reasonable cost and it provides a standardised set of molecular characters that, together and in combination with morphological data, can be used to assess species boundaries. Examination of morphology allows the inclusion of species for which DNA data could not be gathered, for example, museum specimens of rare species that are often very old and pose problems for DNA sequencing.

### The genus *Tetrastichus*

The genus *Tetrastichus* has been attributed to Haliday (1844), even though it was first described by Walker in 1842, but the Walker name has been suppressed by the International Commission on Zoological Nomenclature (1965). Haliday included a single species (*Cirrospilus attalus* Walker (= *T. miser* (Nees)) which then became the type species for *Tetrastichus*. The subsequent fate of *Tetrastichus* has been quite turbulent. Burks (1943) gives a detailed account of nomenclatural and classificatory events up to his publication. The history after 1943 is equally turbulent. Graham (1961) synonymised *Tetrastichus* with *Aprostocetus* Westwood (priority made *Aprostocetus* senior to *Tetrastichus*) and recombined all European species previously in *Tetrastichus* to *Aprostocetus*. Domenichini (1966) rejected this action by Graham, resurrected *Tetrastichus* and recombined all species back to *Tetrastichus*. Bouček (1977) published a key to all genera of Tetrastichinae and maintained the wide interpretation of *Tetrastichus* by Domenichini, as did Kostjukov (1977). Graham (1987) revised the classification of European genera in Tetrastichinae and kept *Tetrastichus* as a valid genus, but now in a very restricted sense, including only species in the so-called *miser*-group (sensu Graham (1961)Domenichini (1966)). The remainder of the species in *Tetrastichus* s.lat. were recombined to other existing, or newly created, genera. The bulk of species were transferred to *Aprostocetus*, which then became a very large genus with several hundred species on a world basis. Bouček (1988) and LaSalle (1994) followed the classification proposed by Graham. Finally, Graham (1991) revised the European species of *Tetrastichus* s.str., including 42 species, of which 20 were described as new. The species were classified into six species groups and with three unplaced species. In this publication, we follow the definition of *Tetrastichus* proposed by Graham (1987), Graham (1991).

*Tetrastichus* species have been found on all continents (Bouček 1988, Graham 1991, LaSalle 1994) and it is the third largest genus of the subfamily Tetrastichinae, after *Aprostocetus* and *Baryscapus* Förster (LaSalle 1994), including hundreds of species. Noyes (2020) lists 492 species names in the genus, but, due to the chaotic history of *Tetrastichus*, it is difficult to establish how many of these names actually represent species of *Tetrastichus* s.str. without examining the type specimens.

### Biology

Biological information is known for about one-third of the species (29 out of 93) treated here. The available information from literature and from our own experience strongly indicate that *Tetrastichus* species are gregarious koinobiont endoparasitoids developing on juvenile stages of mainly holometabolous insects from all the four major orders: Coleoptera, Diptera, Hymenoptera and Lepidoptera (Table 1). Most species are associated with Coleoptera and mainly with phytophagous and xylophagous groups (Buprestidae, Cerambycidae, Chrysomelidae and Curculionidae). The Diptera host groups include a wide range of biologies: saprophagous (*Brachyopa* spp. *Sargus* sp.), xylophagous (*Xylomyia cabrerae*) and gall-making (*Lipara lucens*) species. In Hymenoptera, larvae and pupae of sawflies (Argidae, Tenthredinidae) are targeted. Prior to this article, no host was recorded from Lepidoptera in Europe, but here we present the first record from this order, *Scardia boletella* (Fabricius) (Tineidae), that develops in different species of shelf fungi. According to Bouček (1988), Indo-Australian species of *Tetrastichus* frequently target Lepidoptera, mainly various moths in rice fields, emerging from host pupae and sometimes also act as hyperparasitoids. The Neotropical species *T. periplanetae* Crawford targets cockroach oothecae (LaSalle 1994).

Van Alphen (1980) investigated the life history of *T. coeruleus* (as *T. asparagi*) and *T. crioceridis* (as an undescribed *Tetrastichus* species). Both species are parasitoids of *Crioceris* spp. (Coleoptera: Chrysomelidae) on asparagus (*Asparagus officinalis*), *T. coeruleus* on *C. asparagi* and *T. crioceridis* on *C. duodecimpunctata*. The females of both species oviposit in eggs with advanced embryos or in the larvae of their respective host. Both species are monophagous and, in spite of occurring on the same host plant, they do not overlap with each other. In another paper, Duan et al. (2011) outlined the life history of *T. planipennisi* Yang. *Tetrastichus planipennisi* is an Asiatic species targeting the emerald ash borer (*Agrilus planipennis* Fairmaire) (Coleoptera: Buprestidae). The wasp female oviposits in larval stages 2-4 and it took about four weeks to complete the development from egg to adult wasp.

The sex ratio of clutches is female biased (e.g. Duan et al. 2011 and through experiences from our own rearings). In some cases, no males have been reared, suggesting thelytoky, for example, *T. coeruleus* in North America (Graham 1991).

## Materials and methods

### Morphology

Species of *Tetrastichus* are, like most other Tetrastichinae, taxonomically exceptional amongst Eulophidae because of their high morphological similarity, hampering a taxonomic revision, based on morphology alone. Unless collected through rearing when the host is known, species of *Tetrastichus* are very difficult from which to get a series of specimens. Sweep netting and Malaise trapping only yield one or a few specimens at best per collecting event. The rarity of many species, with only very few specimens of each species available for study, prevents the assessment of intraspecific variation and further complicates the definition of species borders. To alleviate the situation, for the present study, an integrative approach was employed, with DNA barcoding as a first step to obtain operational taxonomic units that were subsequently examined morphologically, using criteria that were traditionally used for delimiting species, i.e. morphologically diagnostic characters. In the absence of sufficient morphological differences between OTUs, a conservative approach was followed in that the OTUs were regarded as conspecific until further evidence is available to assess their species status. In these cases, additional genetic markers are needed to disentangle complexes of closely-related species. In several species, the taxonomy has to rely on very few specimens, often with even fewer DNA barcodes and their species status will need to be evaluated as further specimens (and sequence data) become available. Furthermore, the number of species in Europe is probably much higher than currently known and there is, thus, a high probability that species of *Tetrastichus* that are not included in the present revision will be encountered in the future, in particular with material that is collected in areas other than the main study area in this article and in Graham (1991), i.e. Northern and Central Europe. For these reasons, DNA barcodes, in combination with a thorough morphological examination, will be essential for accurate species level identifications of*Tetrastichus*.

### DNA sequencing

For DNA extraction, whole specimens were sent to the Canadian Centre for DNA Barcoding (CCDB) in Guelph, Canada, for DNA extraction and barcode sequencing and subsequent recovery of vouchers for preparation and morphological study. A complete list of voucher specimens included in the revision is given in Suppl. material S1. DNA extraction, PCR amplification and sequencing were conducted at CCDB, using standardised high-throughput protocols (Ivanova et al. 2006, deWaard et al. 2008). The 658 bp target region, starting from the 5’ end of the mitochondrial cytochrome c oxidase I (COI) gene, includes the DNA barcode region of the animal kingdom (Hebert et al. 2003). The DNA extracts are stored at CCDB. Specimens that were successfully sequenced are listed in Suppl. material S1. All specimen data are accessible in BOLD as a single citable dataset (dx.doi.org/10.5883/DS-TTSEUR).

The data include collecting locality, geographic coordinates, elevation, collector, one or more digital images, identifier and voucher depository. Sequence data can be obtained through BOLD and include a detailed LIMS report, primer information and access to trace files. The sequences are also available on GenBank (for accession numbers, see Suppl. material S1).

Due to the expected presence of complexes of cryptic species, i.e. species that are morphologically virtually indistinguishable, all suitable specimens were subjected to DNA barcoding, even at the risk of obtaining a long series of the same species. The final BOLD dataset (DS-TTSEUR) contains 1,128 specimen records, 1,126 of which are associated with a DNA sequence. Amongst the specimens with a barcode sequence, 860 were assigned a Barcode Index Number (BIN) by the BOLD system, resulting in 73 species with one or more BINs. A total of 60 species were represented by a single BIN, 24 species with two BINs and one species (*T. sinope*) with three BINs (with a single specimen each).

**Figure 1.**
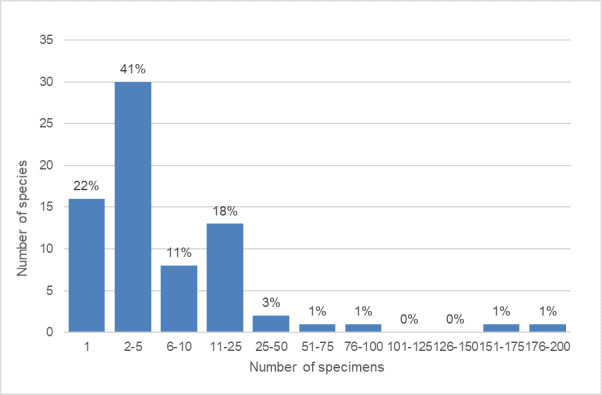
Number of specimens of 73 *Tetrastichus* species that were analysed by DNA barcoding.

The initial concerns with obtaining multiple sequences of the same species turned out to be unwarranted. A total of 22% of the species were represented by singletons, almost two thirds (63%) by 1-5 specimens and only two species with over 100 specimens each (Fig. 1). The species with the largest number of specimens in our dataset include*Tetrastichus atratulus* (178 specimens), *T. halidayi* (155 specimens), followed by *T. miser* (80 specimens), *T. temporalis* (62 specimens), *T. lyridice* (34 specimens) and *T. leocrates* (31 specimens). The remaining 67 species were represented by at most 25 specimens.

### Data analysis

Sequence divergence statistics were calculated using the Kimura two parameter model of sequence evolution (Kimura 1980). Barcode Index Numbers (BINs) were assigned by the BOLD system, representing globally-unique identifiers for clusters of sequences that correspond closely to biological species (Ratnasingham and Hebert 2013). For BIN assignment, a minimum sequence length of 500 bp is required and sequences between 300 and 500 bp can join an existing BIN, but will not create or split BINs. In the present study, BINs were used to delineate Molecular Operational Taxonomic Units (MOTUs) prior to a detailed taxonomic study, based on morphological characters. Sequences were aligned using the BOLD Aligner (amino acid-based hidden Markov models). The analyses are based on sequences with a minimum length of 500 bp and less than 1% ambiguous bases. Genetic distances and summary statistics were calculated using analytical tools in BOLD and are given as mean and maximum pairwise distances for intraspecific variation and as minimum pairwise distances for interspecific variations.

For phylogenetic analyses, the COI sequences were aligned using Muscle (Edgar 2004). Maximum Likelihood (ML) trees were inferred using IQ-TREE, version 2.0.6 (Minh et al. 2020). The best-fit substitution models were inferred separately for the DNA sequence and the gap data partitions using ModelFinder (Kalyaanamoorthy et al. 2017) as implemented in IQ-TREE, prior to tree reconstruction. Branch support was estimated with ultrafast bootstrap estimation (Hoang et al. 2018) using 10,000 bootstrap replicates. The phylogenetic inferences were based on sequences with a minimum length of 500 bp, resulting in a dataset with 748 sequences.

### Museum acronyms

**ETHZ** Swiss Federal Institute of Technology, Entomological Collection, Zürich, Switzerland; **GD** Private collection of Gérard Delvare, Montpellier, France; **HNHM** Hungarian Natural History Museum, Budapest, Hungary; **MZLU** Biological Museum, Entomology, Lund University, Sweden; **NHM** the Natural History Museum, London, United Kingdom; **NHRS** Swedish Museum of Natural History, Stockholm, Sweden; **NMW** Naturhistorisches Museum, Wien, Austria; **OUMNH** Oxford University Museum of Natural History, United Kingdom; **SMTP** Swedish Malaise Trap Project, Station Linné, Ölands Skogsby, Sweden; **UCRC** University of California, Riverside, Entomology, USA; **USNM** United States National Museum of Natural History, Washington, D.C., USA; **ZSM** SNSB-Zoologische Staatssammlung München, Munich, Germany.

### Abbreviations of morphological terms and nomenclature

**Figure 12.**
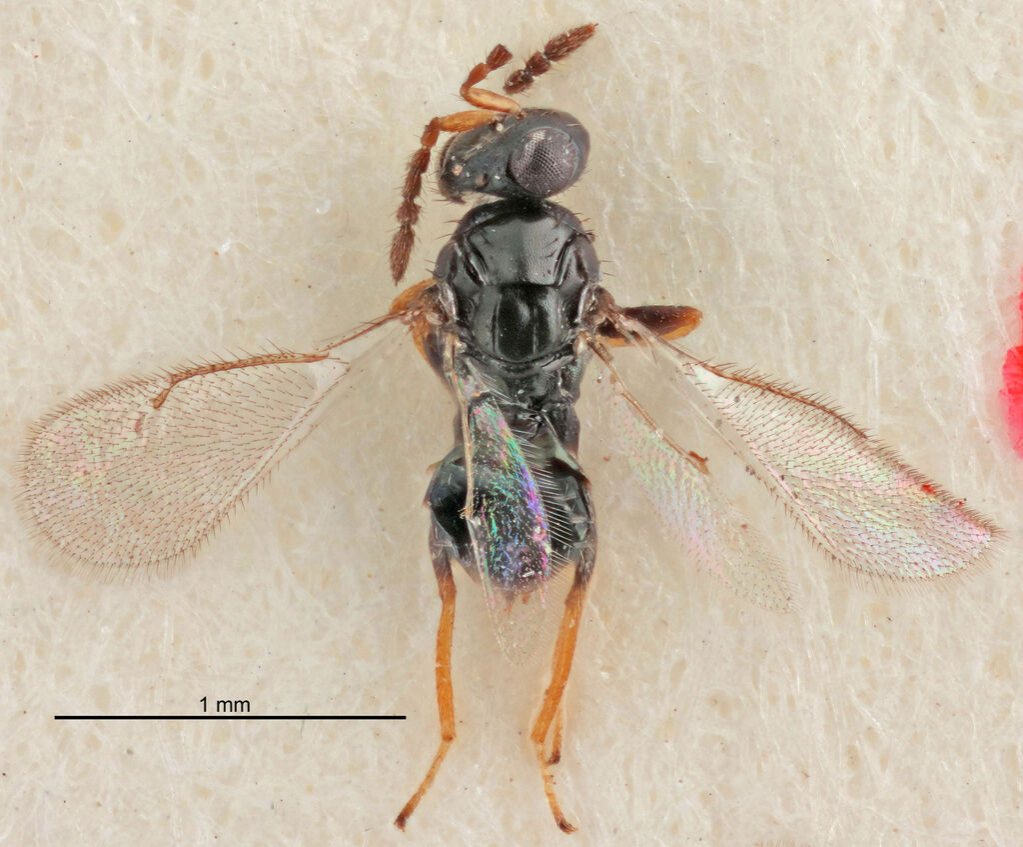
*Tetrastichus solvae* Graham. Holotype female, dorsal.

With some exceptions (below), the morphological terms used here are explained and illustrated (figs. 6–12) in Graham (1987). Abbreviations of morphological terms not used in Graham (1987): Gt_1-7_ gastral tergites 1–7; MG median groove on mid-lobe of mesoscutum (as median line in Graham (1987), Graham (1991)); MV marginal vein; OD diameter of lateral ocelli; ST stigmal vein; SLG sublateral groove on mesoscutellum (as sublateral line in Graham (1987), Graham (1991)); SMG submedian groove on mesoscutellum (as submedian line in Graham (1987), Graham (1991)); T1–4 tarsomeres 1–4.

To avoid the problems associated with the requirement by the International Code of Zoological Nomenclature (International Commission of Zoological Nomenclature 1999) that the gender of species names must agree with that of the genus, we follow Noyes (2004) in that all new names proposed in the present study, except indicated otherwise, must be treated as arbitrary combinations of letters or nouns in apposition. As a consequence, species names will not change if species are transferred to a genus with a different gender at any time in the future.

### Imaging

The colour images of the type specimens of previously-described species were made using Canon camera equipment including an EOS 5D Mark IV body, MP E-65 macro lens and macro twin lite MT-24 EX. The camera was attached to a Cognisys stackshot macro rail system. The images of type specimens for the newly-described species were done with the same equipment, except that the macro lens was substituted with a Canon tele-zoom lens, 70–300 mm (using only 135 & 200 mm), with a 10× Mitutoyo microscope lens attached. The picture stacking was done with Helicon Focus version 6 software and Adobe Photoshop was used for image processing.

The SEM micrographs are from uncoated specimens and were done with a Hitachi SU 3500 scanning electron microscope, in low vacuum.

## Taxon treatments

### Tetrastichus

Haliday, 1844

D9E0ACBA-9BED-52F0-B5B5-3947CE0E7F46

Tetrastichus Haliday 1844:297–298. Type species *Cirrospilus attalus* Walker, by monotypy (=*T. miser* (Nees)). Not *Tetrastichus*Walker 1842:116, a name suppressed by the International Commission on Zoological Nomenclature (1965).
Tetrastichus Graham (1991)
Tetrastichus

#### Diagnosis

European species of *Tetrastichus* can be diagnosed by a combination of three features: submarginal vein in fore wing with one dorsal seta, mid-lobe of mesoscutum with at least three adnotaular setae on each side and propodeum with a ± complete lateral longitudinal carina that splits into two in posterior part (like an inverted "Y", Fig. 2c). In addition, most species have a strong exoskeleton, preventing specimens from collapsing when dried. This last feature is not unique to *Tetrastichus*, but in combination with the other three features, it helps when diagnosing this group.

#### Identification keys

Figs 2, 3:The identification keys are based on the two keys in Graham (1991), one for females and one for males, with the new species included and with some modifications of features included and order of couplets slightly changed. The main reason for keeping the sexes apart is that differences between species are frequently based on features unique to each sex (antennal characteristics and length of female gaster). Since males are unknown for several of the species, the male key is distinctly shorter than the female key.

### Tetrastichus murcia group

B4350BD8-FCA6-5189-A2F9-BD62EFFDB713

#### Diagnosis

Eyes with long setae (e.g. Fig. 7d); female antenna with relatively short flagellomeres and with a distinct clava (e.g. Fig. 5a); male flagellomeres 1–4 with an externo-dorsal, sub-basal compact whorl of long setae (e.g. Fig. 5d); setae along hind margin of pronotum and adnotaular setae on mesoscutum long and erect (e.g. Fig. 7a); body black non-metallic or with weak metallic tinges; female gaster circular or short ovate.

### Tetrastichus antonjanssoni sp. n.

9BAE3D66-5D30-5352-B652-31BD71ABE15F

urn:lsid:zoobank.org:act:BD5F1E7B-17EB-4401-96CA-C18F233A1A11

#### Materials

- **Type status:** Holotype. **Occurrence:** occurrenceDetails: http://www.boldsystems.org/index.php/API_Public/specimen?ids=BC-ZSM-HYM-29813-C08; catalogNumber: BC-ZSM-HYM-29813-C08; recordNumber: BC-ZSM-HYM-29813-C08; recordedBy: Anton Jansson; individualID: BC-ZSM-HYM-29813-C08; individualCount: 1; sex: female; lifeStage: adult; **Taxon:** scientificName: Tetrastichus antonjanssoni; phylum: Arthropoda; class: Insecta; order: Hymenoptera; family: Eulophidae; genus: Tetrastichinae; taxonRemarks: Holotype deposited in MZLU; **Location:** country: Sweden; decimalLatitude: 59.2753; decimalLongitude: 15.2134; **Record Level:** type: PhysicalObject; language: en; institutionCode: MZLU; basisOfRecord: PreservedSpecimen

#### Description

FEMALE holotype (Fig. 4). Body length 1.5 mm. *Head*. Width/length (dorsal view) 2.3, width/length (frontal view) 1.2, POL/OOL 2.2, widths head/mesosoma 1.1, mouth width/malar space 1.0, malar space/eye height 0.8. *Antenna*. Scape length/eye height 1.0, pedicel+flagellum length/mesosoma width 1.2, length/width F1, F2, F3 1.6, 1.4, 1.1, clava length/width 2.2, lengths pedicel/F1 0.8, lengths F1/F2 1.1, F1/F3 1.2, lengths F1, F2, F3/clava 0.5, 0.5, 0.5, widths F1/pedicel (dorsal view) 1.4, lengths antennal spicule/C3 0.3. *Mesosoma*. Length/width 1.3, mesoscutal midlobe length/width (width measured in anterior part) 0.8, mid-lobe with median groove in posterior ½, with three adnotaular setae on each side, lengths mesoscutum/mesoscutellum (measured medially) 1.2, mesoscutellum length/width 0.8, length/width of enclosed space between submedian grooves 2.1, distance between SMG/distance between SMG and SLG 1.7, lengths dorsellum/propodeum (measured medially) 0.5, propodeum with strong reticulation, propodeal callus with four setae. *Fore wing*. Costal cell length/width 12.5, lengths costal cell/marginal vein 1.0, lengths marginal/stigmal veins 3.1. *Gaster*. Subcircular to ovate, length/width 1.7, lengths gaster/mesosoma 1.1, Gt_7_ length/width 0.4, length longest seta of each cercus/next longest seta 1.6, longest cercal seta nearly straight, ovipositor sheaths projecting slightly beyond apex of Gt_7_.

Colour. Body with weak metallic greenish-blue tinges, scape yellowish-brown with dorsal edge brown, pedicel and flagellum dark brown, tegulae dark brown, wings hyaline with veins pale yellowish-brown, coxae and femora concolorous with body, trochanters dark brown, tibiae and tarsi yellowish-brown.

MALE. Unknown.

#### Diagnosis

Antenna very short with F1 1.6×, F2 1.4×, F3 1.1× and clava 2.2× as long as wide; POL/OOL 2.2; distance between SMG 1.7× distance SMG to SLG; ovipositor sheaths protruding slightly beyond apex of Gt_7_.

#### Etymology

Named after the collector of the holotype, the Swedish entomologist Anton Jansson, who collected insects mainly in the vicinities of the city Örebro (Närke Province), where he also lived and worked as a journalist.

#### Distribution

Sweden.

#### Ecology

##### Host

Unknown.

#### Notes

Holotype deposited in MZLU.

### Tetrastichus atratulus

(Nees, 1834)

53E3D40A-B249-580A-9AE7-92AF08C53641

Eulophus atratulus Nees von Esenbeck 1834:180. Neotype ♀ in NHM, designated by Graham 1991:264, examined (Fig. 5). Combined to *Tetrastichus* by Domenichini 1966:194.
Tetrastichus puncticoxae Kurdjumov 1913:253, 255. Holotype ♀ in Leningrad, not examined. Synonymised with *T. atratulus* by Graham 1991:264.

#### Description

See Graham 1991.

#### Diagnosis

Female antenna with length of pedicel+flagellum 1.15–1.35× width of mesoscutum, F1 2.0–2.5×, F2 1.6–2.0× and clava (incl. spicule) 2.4–3.0× as long as wide; submedian grooves of mesoscutellum nearer to sublateral grooves than to each other; apical part of ovipositor sheaths does not reach apex of Gt_7_. Similar to *T. dasyops*, but with longer flagellum and shorter ovipositor sheaths. Male with relatively short hairs on eyes, about 0.3× OD.

#### Distribution

(Former) Czechoslovakia, France, Germany, Italy, Japan, (former) Yugoslavia, Russia, United Kingdom (Graham 1991), Sweden (Hedqvist 2003), Austria and Romania (**new records**).

#### Ecology

##### Host

*Ptecticus tenebrifer* (Walker) (Diptera: Stratiomyiidae) (in Japan) (Graham 1991).

##### Material examined

Type material: neotype ♀ (NHM, type no. 5.3627). Additional material (292♀ 55♂): Austria 2♀ (MZLU, UCRC), France 51♀ 2♂ (CIRAD, NHM, ZSM), Germany 10♀ (MZLU, ZSM, UCRC), Italy 10♀2♂ (NHM, UCRC), Romania 132♀ 50♂ (MZLU, NHM, ZSM), Russia 3♀ (UCRC), Sweden 64♀ 1♂ (MZLU, NHM, NHRS, SMTP, ZSM), United Kingdom 20♀ (NHM).

#### Material examined

### Tetrastichus brachyopae

Graham, 1991

0E8CBC2B-AF9A-51D3-A2CA-9989AAEA4C36

Tetrastichus brachyopae Graham 1991:265–266. Holotype ♀ in NHM, examined (Fig. 6).

#### Description

See Graham (1991).

#### Diagnosis

Submedian grooves of mesoscutellum equidistant from each other and from sublateral grooves; female gaster with ovipositor sheaths projecting beyond apex of Gt_7_ ; female antennal funicle rather stout, its parts relatively short, F1 1.5–1.7×, F2 1.4–1.5× as long as broad; male with relatively short setae on eyes, about 0.3× OD.

#### Distribution

(Former) Czechoslovakia (Graham 1991), The Netherlands (van Eck et al. 2016) and Sweden (Hedqvist 2003).

#### Ecology

##### Host

Gregarious koinobiont endoparasitoid on *Brachyopa* spp. (Diptera: Syrphidae), clutch size varying from 7–18 specimens, with a strong female bias (van Eck et al. 2016). Recorded from *Brachyopa pilosa* Collin (Graham 1991), *B. bicolor* (Fallén) & *B. insensilis* Collin (van Eck et al. 2016). In Sweden, collected at sap runs on *Ulmus glabra* and *Carpinus betulus* (R. Bygebjerg (MZLU), verbal communication).

##### Material examined

Type material: holotype ♀ (NHM, type no. 5.3628). Additional material (39♀ 3♂): the Netherlands 13♀ 3♂ (NHM), Sweden 26♀ (MZLU, NHM, ZSM).

### Tetrastichus dasyops

Graham, 1991

762DF3E3-CEE6-50C5-8D58-F55B3E9ECADA

Tetrastichus dasyops Graham 1991:263–264. Holotype ♀ in NHM, examined (Fig. 7).

#### Description

See Graham (1991).

#### Diagnosis

Female with length of pedicel+flagellum 1.0–1.05× width of mesoscutum, F1 1.7–2.0×, F2 1.2–1.6× and antennal clava (incl. spicule) 2.1–2.35× as long as wide; submedian grooves of mesoscutellum nearer to sublateral grooves than to each other; ovipositor sheaths reach apex of Gt_7_or slightly beyond. Similar to *T. atratulus*, but with shorter antennal flagellum and longer ovipositor sheaths.

#### Distribution

United Kingdom (Graham 1991), Sweden (Hedqvist 2003), France, Italy, Romania, Russia, Spain, Switzerland (**new records**).

#### Ecology

##### Host

Unknown.

#### Material examined

Type material: holotype ♀ (NHM, type no. 5.3626). Additional material (158♀ 14♂): France 7♀ 1♂ (GD, NHM), Italy 5♀ (NHM), Romania 3♀ (NHM), Russia 16♀ 1♂ (MZLU, UCRC), Spain 1♀ (NHM), Sweden 122♀ 12♂ (MZLU, NHM, NHRS), Switzerland 1♀ (NHM), United Kingdom 3♀ (NHM).

### Tetrastichus intruitus sp. n.

A0D1C2C3-9AE4-5A3F-AF30-5C70AB9452B1

urn:lsid:zoobank.org:act:D25BBA20-4628-4516-B454-876A16F12F92

#### Materials

- **Type status:** Holotype. **Occurrence:** occurrenceDetails: http://www.boldsystems.org/index.php/API_Public/specimen?ids=BC-ZSM-HYM-27768-D01; catalogNumber: BC-ZSM-HYM-27768-D01; recordNumber: BC-ZSM-HYM-27768-D01; recordedBy: J.S. Noyes; individualID: BC-ZSM-HYM-27768-D01; individualCount: 1; sex: female; lifeStage: adult; **Taxon:** scientificName: Tetrastichus intruitus; phylum: Arthropoda; class: Insecta; order: Hymenoptera; family: Eulophidae; genus: Tetrastichinae; taxonRemarks: Holotype deposited in NHM; **Location:** country: Romania; decimalLatitude: 46.985; decimalLongitude: 27.585; **Record Level:** type: PhysicalObject; language: en; institutionCode: NHM; basisOfRecord: PreservedSpecimen
- **Type status:** Paratype. **Occurrence:** occurrenceDetails: http://www.boldsystems.org/index.php/API_Public/specimen?ids=BC-ZSM-HYM-27768-A07; catalogNumber: BC-ZSM-HYM-27768-A07; recordNumber: BC-ZSM-HYM-27768-A07; recordedBy: J.S. Noyes; individualID: BC-ZSM-HYM-27768-A07; individualCount: 1; sex: female; lifeStage: adult; **Taxon:** scientificName: Tetrastichus intruitus; phylum: Arthropoda; class: Insecta; order: Hymenoptera; family: Eulophidae; genus: Tetrastichinae; **Location:** country: Romania; decimalLatitude: 47.011; decimalLongitude: 27.603; **Record Level:** type: PhysicalObject; language: en; basisOfRecord: PreservedSpecimen
- **Type status:** Paratype. **Occurrence:** occurrenceDetails: http://www.boldsystems.org/index.php/API_Public/specimen?ids=BC-ZSM-HYM-27768-C12; catalogNumber: BC-ZSM-HYM-27768-C12; recordNumber: BC-ZSM-HYM-27768-C12; recordedBy: J.S. Noyes; individualID: BC-ZSM-HYM-27768-C12; individualCount: 1; sex: female; lifeStage: adult; **Taxon:** scientificName: Tetrastichus intruitus; phylum: Arthropoda; class: Insecta; order: Hymenoptera; family: Eulophidae; genus: Tetrastichinae; **Location:** country: Romania; decimalLatitude: 46.985; decimalLongitude: 27.585; **Record Level:** type: PhysicalObject; language: en; basisOfRecord: PreservedSpecimen
- **Type status:** Paratype. **Occurrence:** occurrenceDetails: http://www.boldsystems.org/index.php/API_Public/specimen?ids=BC-ZSM-HYM-27768-C06; catalogNumber: BC-ZSM-HYM-27768-C06; recordNumber: BC-ZSM-HYM-27768-C06; recordedBy: J.S. Noyes; individualID: BC-ZSM-HYM-27768-C06; individualCount: 1; sex: female; lifeStage: adult; **Taxon:** scientificName: Tetrastichus intruitus; phylum: Arthropoda; class: Insecta; order: Hymenoptera; family: Eulophidae; genus: Tetrastichinae; **Location:** country: Romania; decimalLatitude: 47.011; decimalLongitude: 27.603; **Record Level:** type: PhysicalObject; language: en; basisOfRecord: PreservedSpecimen
- **Type status:** Paratype. **Occurrence:** occurrenceDetails: http://www.boldsystems.org/index.php/API_Public/specimen?ids=BC-ZSM-HYM-22524-C12; catalogNumber: BC-ZSM-HYM-22524-C12; recordNumber: BC-ZSM-HYM-22524-C12; recordedBy: C. Hansson; individualID: BC-ZSM-HYM-22524-C12; individualCount: 1; sex: male; lifeStage: adult; **Taxon:** scientificName: Tetrastichus intruitus; phylum: Arthropoda; class: Insecta; order: Hymenoptera; family: Eulophidae; genus: Tetrastichinae; **Location:** country: Hungary; decimalLatitude: 47; decimalLongitude: 17.27; **Record Level:** type: PhysicalObject; language: en; institutionCode: MZLU; basisOfRecord: PreservedSpecimen
- **Type status:** Paratype. **Occurrence:** occurrenceDetails: http://www.boldsystems.org/index.php/API_Public/specimen?ids=BC-ZSM-HYM-27768-B03; catalogNumber: BC-ZSM-HYM-27768-B03; recordNumber: BC-ZSM-HYM-27768-B03; recordedBy: J.S. Noyes; individualID: BC-ZSM-HYM-27768-B03; individualCount: 1; sex: female; lifeStage: adult; **Taxon:** scientificName: Tetrastichus intruitus; phylum: Arthropoda; class: Insecta; order: Hymenoptera; family: Eulophidae; genus: Tetrastichinae; **Location:** country: Romania; decimalLatitude: 47.011; decimalLongitude: 27.603; **Record Level:** type: PhysicalObject; language: en; basisOfRecord: PreservedSpecimen
- **Type status:** Paratype. **Occurrence:** occurrenceDetails: http://www.boldsystems.org/index.php/API_Public/specimen?ids=BC-ZSM-HYM-27770-A10; catalogNumber: BC-ZSM-HYM-27760-A10; recordNumber: BC-ZSM-HYM-27760-A10; recordedBy: J.S. Noyes; individualID: BC-ZSM-HYM-27770-A10; individualCount: 1; sex: female; lifeStage: adult; **Taxon:** scientificName: Tetrastichus intruitus; phylum: Arthropoda; class: Insecta; order: Hymenoptera; family: Eulophidae; genus: Tetrastichinae; **Location:** country: Romania; decimalLatitude: 46.9853; decimalLongitude: 27.5847; **Record Level:** type: PhysicalObject; language: en; institutionCode: NHM; basisOfRecord: PreservedSpecimen
- **Type status:** Paratype. **Occurrence:** occurrenceDetails: http://www.boldsystems.org/index.php/API_Public/specimen?ids=BC-ZSM-HYM-27770-A08; catalogNumber: BC-ZSM-HYM-27760-A08; recordNumber: BC-ZSM-HYM-27760-A08; recordedBy: J.S. Noyes; individualID: BC-ZSM-HYM-27770-A08; individualCount: 1; sex: female; lifeStage: adult; **Taxon:** scientificName: Tetrastichus intruitus; phylum: Arthropoda; class: Insecta; order: Hymenoptera; family: Eulophidae; genus: Tetrastichinae; **Location:** country: Romania; decimalLatitude: 46.9853; decimalLongitude: 27.5847; **Record Level:** type: PhysicalObject; language: en; institutionCode: NHM; basisOfRecord: PreservedSpecimen
- **Type status:** Paratype. **Occurrence:** occurrenceDetails: http://www.boldsystems.org/index.php/API_Public/specimen?ids=BC-ZSM-HYM-27768-A06; catalogNumber: BC-ZSM-HYM-27768-A06; recordNumber: BC-ZSM-HYM-27768-A06; recordedBy: J.S. Noyes; individualID: BC-ZSM-HYM-27768-A06; individualCount: 1; sex: female; lifeStage: adult; **Taxon:** scientificName: Tetrastichus intruitus; phylum: Arthropoda; class: Insecta; order: Hymenoptera; family: Eulophidae; genus: Tetrastichinae; **Location:** country: Romania; decimalLatitude: 47.011; decimalLongitude: 27.603; **Record Level:** type: PhysicalObject; language: en; basisOfRecord: PreservedSpecimen
- **Type status:** Paratype. **Occurrence:** occurrenceDetails: http://www.boldsystems.org/index.php/API_Public/specimen?ids=BC-ZSM-HYM-27768-F09; catalogNumber: BC-ZSM-HYM-27768-F09; recordNumber: BC-ZSM-HYM-27768-F09; recordedBy: J.S. Noyes; individualID: BC-ZSM-HYM-27768-F09; individualCount: 1; sex: F; lifeStage: a; associatedMedia: http://www.boldsystems.org/pics/BCHYM/BC-ZSM-HYM-27768-F09+1445344240.jpg; **Taxon:** scientificName: Tetrastichus intruitus; phylum: Arthropoda; class: Insecta; order: Hymenoptera; family: Eulophidae; genus: Tetrastichus; **Location:** country: Romania; locality: Iasi Botanical Garden; decimalLatitude: 47.188; decimalLongitude: 27.549; **Identification:** identifiedBy: Christer Hansson

#### Description

FEMALE holotype (Fig. 8). Body length 1.4 mm (paratypes 1.4–1.6 mm). *Head*. Width/length (dorsal view) 2.2, width/length (frontal view) 2.0, POL/OOL 2.3, widths head/mesosoma 1.1, mouth width/malar space 1.0, malar space/eye height 0.7. *Antenna*. Scape length/eye height 1.0, pedicel+flagellum length/mesosoma width 1.3, length/width F1, F2, F3 2.5, 2.5, 1.9, clava length/width 3.0, lengths pedicel/F1 0.7, lengths F1/F2 1.0, F1/F3 1.2, lengths F1, F2, F3/clava 0.6, 0.6, 0.5, widths F1/pedicel (dorsal view) 1.0, lengths antennal spicule/C3 0.4. *Mesosoma*. Length/width 1.4, mesoscutal mid-lobe length/width (width measured in anterior part) 0.9, mid-lobe with median groove in posterior ⅓, with four adnotaular setae on each side, lengths mesoscutum/mesoscutellum (measured medially) 1.1, mesoscutellum length/width 0.9, length/width of enclosed space between submedian grooves 2.6, distance between SMG/distance between SMG and SLG 1.2, lengths dorsellum/propodeum (measured medially) 0.6, propodeum with strong reticulation, propodeal callus with three setae. *Fore wing*. Costal cell length/width 11.0, lengths costal cell/marginal vein 0.9, lengths marginal/stigmal veins 3.3. *Gaster*. Subcircular to ovate, length/width 1.5, lengths gaster/mesosoma 1.1, Gt_7_ length/width 0.5, length of longest cercal seta/next longest seta 2.0, longest cercal seta slightly kinked in apical ¼, ovipositor sheaths projecting slightly beyond apex of Gt_7_.

Colour. Body with weak coppery tinges, entire antenna dark brown, tegulae dark brown, wings hyaline with veins yellowish-white, coxae and femora black, trochanters dark brown, tibiae dark brown to black with apex yellowish-brown, tarsi yellowish-brown or dark brown.

Variation. Scape yellowish-brown (one specimen) or pale brown (one specimen).

MALE. Body length 1.3 mm. *Head*. Width/length (dorsal view) 2.5, width/length (frontal view) 2.1, mouth width/malar space 1.4, widths head/mesosoma 1.2. *Antenna*. F1–F4 without basal whorls of setae, scape length/eye height 0.9, scape length/width 2.5, ventral plaque placed in central part of scape, lengths ventral plaque/scape 0.6, pedicel+flagellum length/mesosoma width 2.0, length/width F1, F2, F3, F4 3.0, 2.7, 2.7, 2.7, clava length/width 6.7, lengths pedicel/F1 0.7, lengths F1/F2 1.1, F1/F3 1.1, F1/F4 1.1, lengths F1, F2, F3, F4/clava 0.5, 0.4, 0.4, 0.4.

Colour. As in female.

#### Diagnosis

Female with antennal flagellum long, for example, F1 and F2 both 2.5× and F3 1.9× as long as wide and pedicel+flagellum 1.32× as long as width of mesoscutum; ovipositor sheaths not protruding beyond apex of Gt_7_. Male with scattered setae and without externo-dorsal, sub-basal compact whorls of long dark setae on funiculars. Through the erect setae on vertex and erect adnotaular setae, similar to species in the *murcia* -group.

#### Distribution

Hungary, Romania.

#### Ecology

##### Host

Unknown.

#### Notes

Holotype deposited in NHM, paratypes in MZLU, NHM.

### Tetrastichus lacustrinus sp. n.

37723386-61B2-5D1E-8C45-DBBB48E5FA1D

urn:lsid:zoobank.org:act:9171ECE1-C208-45C1-A34F-F0C4693F428F

#### Materials

- **Type status:** Holotype. **Occurrence:** occurrenceDetails: http://www.boldsystems.org/index.php/API_Public/specimen?ids=BC-ZSM-HYM-21587-E06; catalogNumber: BC-ZSM-HYM-21587-E06; recordNumber: BC-ZSM-HYM-21587-E06; recordedBy: C.Hansson; individualID: BC-ZSM-HYM-21587-E06; individualCount: 1; sex: female; lifeStage: adult; **Taxon:** scientificName: Tetrastichus lacustrinus; phylum: Arthropoda; class: Insecta; order: Hymenoptera; family: Eulophidae; genus: Tetrastichinae; taxonRemarks: Holotype deposited in MZLU; **Location:** country: Sweden; decimalLatitude: 55.802; decimalLongitude: 13.747; **Record Level:** type: PhysicalObject; language: en; institutionCode: MZLU; basisOfRecord: PreservedSpecimen
- **Type status:** Paratype. **Occurrence:** occurrenceDetails: http://www.boldsystems.org/index.php/API_Public/specimen?ids=BC-ZSM-HYM-21587-B11; catalogNumber: BC-ZSM-HYM-21587-B11; recordNumber: BC-ZSM-HYM-21587-B11; recordedBy: C.Hansson; individualID: BC-ZSM-HYM-21587-B11; individualCount: 1; sex: female; lifeStage: adult; **Taxon:** scientificName: Tetrastichus lacustrinus; phylum: Arthropoda; class: Insecta; order: Hymenoptera; family: Eulophidae; genus: Tetrastichinae; **Location:** country: Sweden; decimalLatitude: 56.714; decimalLongitude: 16.763; **Record Level:** type: PhysicalObject; language: en; basisOfRecord: PreservedSpecimen
- **Type status:** Paratype. **Occurrence:** occurrenceDetails: http://www.boldsystems.org/index.php/API_Public/specimen?ids=BC-ZSM-HYM-21587-E01; catalogNumber: BC-ZSM-HYM-21587-E01; recordNumber: BC-ZSM-HYM-21587-E01; recordedBy: C.Hansson; individualID: BC-ZSM-HYM-21587-E01; individualCount: 1; sex: female; lifeStage: adult; **Taxon:** scientificName: Tetrastichus lacustrinus; phylum: Arthropoda; class: Insecta; order: Hymenoptera; family: Eulophidae; genus: Tetrastichinae; **Location:** country: Sweden; decimalLatitude: 55.834; decimalLongitude: 13.528; **Record Level:** type: PhysicalObject; language: en; basisOfRecord: PreservedSpecimen
- **Type status:** Paratype. **Occurrence:** occurrenceDetails: http://www.boldsystems.org/index.php/API_Public/specimen?ids=BC-ZSM-HYM-21587-A09; catalogNumber: BC-ZSM-HYM-21587-A09; recordNumber: BC-ZSM-HYM-21587-A09; recordedBy: C.Hansson; individualID: BC-ZSM-HYM-21587-A09; individualCount: 1; sex: female; lifeStage: adult; **Taxon:** scientificName: Tetrastichus lacustrinus; phylum: Arthropoda; class: Insecta; order: Hymenoptera; family: Eulophidae; genus: Tetrastichinae; **Location:** country: Sweden; decimalLatitude: 55.834; decimalLongitude: 13.528; **Record Level:** type: PhysicalObject; language: en; basisOfRecord: PreservedSpecimen
- **Type status:** Paratype. **Occurrence:** occurrenceDetails: http://www.boldsystems.org/index.php/API_Public/specimen?ids=BC-ZSM-HYM-29813-D09; catalogNumber: BC-ZSM-HYM-29813-D09; recordNumber: BC-ZSM-HYM-29813-D09; recordedBy: C. Hansson; individualID: BC-ZSM-HYM-29813-D09; individualCount: 1; sex: male; lifeStage: adult; **Taxon:** scientificName: Tetrastichus lacustrinus; phylum: Arthropoda; class: Insecta; order: Hymenoptera; family: Eulophidae; genus: Tetrastichinae; **Location:** country: Sweden; decimalLatitude: 55.6833; decimalLongitude: 13.4833; **Record Level:** type: PhysicalObject; language: en; basisOfRecord: PreservedSpecimen

#### Description

FEMALE holotype (Fig. 9). Body length 1.9 mm (paratypes 1.7–1.9 mm). *Head*. Width/length (dorsal view) 2.4, width/length (frontal view) 1.2, POL/OOL 2.0, widths head/mesosoma 1.1, mouth width/malar space 1.0, malar space/eye height 0.8. *Antenna*. Scape length/eye height 1.0, pedicel+flagellum length/mesosoma width 1.2, length/width F1, F2, F3 2.0, 1.9, 1.4, clava length/width 2.8, lengths pedicel/F1 0.9, lengths F1/F2 1.1, F1/F3 1.3, lengths F1, F2, F3/clava 0.6, 0.5, 0.4, widths F1/pedicel (dorsal view) 1.3, lengths antennal spicule/C3 0.3. *Mesosoma*. Length/width 1.5, mesoscutal mid-lobe length/width (width measured in anterior part) 0.9, mid-lobe with median groove in posterior ½, with six adnotaular setae on each side, lengths mesoscutum/mesoscutellum (measured medially) 1.2, mesoscutellum length/width 0.8, length/width of enclosed space between submedian grooves 2.2, distance between SMG/distance between SMG and SLG 1.7, lengths dorsellum/propodeum (measured medially) 0.4, propodeum with strong reticulation, propodeal callus with five setae. *Fore wing*. Costal cell length/width 10.0, lengths costal cell/marginal vein 1.0, lengths marginal/stigmal veins 3.1. *Gaster*. Subcircular to ovate, length/width 1.5, lengths gaster/mesosoma 1.0, Gt_7_ length/width 0.5, length of longest cercal seta/next longest seta 1.8, longest seta almost straight, ovipositor sheaths not protruding beyond apex of Gt_7_.

Colour. Body with weak coppery tinges, scape, flagellum and pedicel dark brown, tegulae dark brown, wings hyaline with veins yellowish-brown to brown, coxae black with metallic green tinges, trochanters dark brown, femora black, tibiae yellowish-brown, tarsi dark yellowish-brown to infuscate.

Variation. Paratypes with scape pale brown to yellowish-brown with dorsal edge brown, wing veins yellowish-white.

MALE. Body length 1.2 mm. *Head*. Width/length (dorsal view) 2.1, width/length (frontal view) 1.3, mouth width/malar space 1.1, eye height/malar space 1.3, widths head/mesosoma 1.1. *Antenna*. F1–F4 with basal whorls of setae, these setae reaching beyond apex of flagellomere attached to, scape length/eye height 1.0, scape length/width 2.6, ventral plaque placed in central part of scape, lengths ventral plaque/scape 0.8, length/width F1, F2, F3, F4 1.7, 1.8, 1.5, 2.0, lengths pedicel/F1 1.0, lengths F1/F2 0.9, F1/F3 1.1, F1/F4 0.8.

#### Diagnosis

Female with antennal clava (incl. spicule) 2.8× as long as wide; POL/OOL 2.0; distance between SMG 1.7× distance SMG to SLG; ovipositor sheaths not protruding beyond apex of Gt_7_.

#### Distribution

Sweden.

#### Ecology

##### Host

Unknown.

#### Notes

Holotype deposited in MZLU, paratypes in MZLU.

### Tetrastichus mixtus sp. n.

E0035483-26B4-5D94-92B8-613A91F24F51

urn:lsid:zoobank.org:act:664AFD68-1D68-42A0-AA27-BF1656CD49E3

#### Materials

- **Type status:** Holotype. **Occurrence:** occurrenceDetails: http://www.boldsystems.org/index.php/API_Public/specimen?ids=BC-ZSM-HYM-27768-H02; catalogNumber: BC-ZSM-HYM-27768-H02; recordNumber: BC-ZSM-HYM-27768-H02; recordedBy: J.S. Noyes; individualID: BC-ZSM-HYM-27768-H02; individualCount: 1; sex: female; lifeStage: adult; **Taxon:** scientificName: Tetrastichus mixtus; phylum: Arthropoda; class: Insecta; order: Hymenoptera; family: Eulophidae; genus: Tetrastichinae; taxonRemarks: Holotype deposited in NHM; **Location:** country: Romania; decimalLatitude: 47.244; decimalLongitude: 27.483; **Record Level:** type: PhysicalObject; language: en; institutionCode: NHM; basisOfRecord: PreservedSpecimen
- **Type status:** Paratype. **Occurrence:** occurrenceDetails: http://www.boldsystems.org/index.php/API_Public/specimen?ids=BC-ZSM-HYM-27770-B01; catalogNumber: BC-ZSM-HYM-27760-B01; recordNumber: BC-ZSM-HYM-27760-B01; recordedBy: J.S. Noyes; individualID: BC-ZSM-HYM-27770-B01; individualCount: 1; sex: female; lifeStage: adult; **Taxon:** scientificName: Tetrastichus mixtus; phylum: Arthropoda; class: Insecta; order: Hymenoptera; family: Eulophidae; genus: Tetrastichinae; **Location:** country: Romania; decimalLatitude: 47.2442; decimalLongitude: 27.4828; **Record Level:** type: PhysicalObject; language: en; basisOfRecord: PreservedSpecimen
- **Type status:** Paratype. **Occurrence:** occurrenceDetails: http://www.boldsystems.org/index.php/API_Public/specimen?ids=BC-ZSM-HYM-27768-H07; catalogNumber: BC-ZSM-HYM-27768-H07; recordNumber: BC-ZSM-HYM-27768-H07; recordedBy: J.S. Noyes; individualID: BC-ZSM-HYM-27768-H07; individualCount: 1; sex: female; lifeStage: adult; **Taxon:** scientificName: Tetrastichus mixtus; phylum: Arthropoda; class: Insecta; order: Hymenoptera; family: Eulophidae; genus: Tetrastichinae; **Location:** country: Romania; decimalLatitude: 47.244; decimalLongitude: 27.483; **Record Level:** type: PhysicalObject; language: en; basisOfRecord: PreservedSpecimen
- **Type status:** Paratype. **Occurrence:** occurrenceDetails: http://www.boldsystems.org/index.php/API_Public/specimen?ids=BC-ZSM-HYM-27768-H06; catalogNumber: BC-ZSM-HYM-27768-H06; recordNumber: BC-ZSM-HYM-27768-H06; recordedBy: J.S. Noyes; individualID: BC-ZSM-HYM-27768-H06; individualCount: 1; sex: female; lifeStage: adult; **Taxon:** scientificName: Tetrastichus mixtus; phylum: Arthropoda; class: Insecta; order: Hymenoptera; family: Eulophidae; genus: Tetrastichinae; **Location:** country: Romania; decimalLatitude: 47.244; decimalLongitude: 27.483; **Record Level:** type: PhysicalObject; language: en; basisOfRecord: PreservedSpecimen
- **Type status:** Paratype. **Occurrence:** occurrenceDetails: http://www.boldsystems.org/index.php/API_Public/specimen?ids=BC-ZSM-HYM-27768-H05; catalogNumber: BC-ZSM-HYM-27768-H05; recordNumber: BC-ZSM-HYM-27768-H05; recordedBy: J.S. Noyes; individualID: BC-ZSM-HYM-27768-H05; individualCount: 1; sex: female; lifeStage: adult; **Taxon:** scientificName: Tetrastichus mixtus; phylum: Arthropoda; class: Insecta; order: Hymenoptera; family: Eulophidae; genus: Tetrastichinae; **Location:** country: Romania; decimalLatitude: 47.244; decimalLongitude: 27.483; **Record Level:** type: PhysicalObject; language: en; basisOfRecord: PreservedSpecimen
- **Type status:** Paratype. **Occurrence:** occurrenceDetails: http://www.boldsystems.org/index.php/API_Public/specimen?ids=BC-ZSM-HYM-27770-A11; catalogNumber: BC-ZSM-HYM-27760-A11; recordNumber: BC-ZSM-HYM-27760-A11; recordedBy: J.S. Noyes; individualID: BC-ZSM-HYM-27770-A11; individualCount: 1; sex: female; lifeStage: adult; **Taxon:** scientificName: Tetrastichus mixtus; phylum: Arthropoda; class: Insecta; order: Hymenoptera; family: Eulophidae; genus: Tetrastichinae; **Location:** country: Romania; decimalLatitude: 47.2442; decimalLongitude: 27.4828; **Record Level:** type: PhysicalObject; language: en; basisOfRecord: PreservedSpecimen
- **Type status:** Paratype. **Occurrence:** occurrenceDetails: http://www.boldsystems.org/index.php/API_Public/specimen?ids=BC-ZSM-HYM-27770-C09; catalogNumber: BC-ZSM-HYM-27760-C09; recordNumber: BC-ZSM-HYM-27760-C09; recordedBy: J.S. Noyes; individualID: BC-ZSM-HYM-27770-C09; individualCount: 1; sex: female; lifeStage: adult; **Taxon:** scientificName: Tetrastichus mixtus; phylum: Arthropoda; class: Insecta; order: Hymenoptera; family: Eulophidae; genus: Tetrastichinae; **Location:** country: Romania; decimalLatitude: 47.2442; decimalLongitude: 27.4828; **Record Level:** type: PhysicalObject; language: en; basisOfRecord: PreservedSpecimen
- **Type status:** Paratype. **Occurrence:** occurrenceDetails: http://www.boldsystems.org/index.php/API_Public/specimen?ids=BC-ZSM-HYM-27770-C08; catalogNumber: BC-ZSM-HYM-27760-C08; recordNumber: BC-ZSM-HYM-27760-C08; recordedBy: J.S. Noyes; individualID: BC-ZSM-HYM-27770-C08; individualCount: 1; sex: female; lifeStage: adult; **Taxon:** scientificName: Tetrastichus mixtus; phylum: Arthropoda; class: Insecta; order: Hymenoptera; family: Eulophidae; genus: Tetrastichinae; **Location:** country: Romania; decimalLatitude: 47.2442; decimalLongitude: 27.4828; **Record Level:** type: PhysicalObject; language: en; basisOfRecord: PreservedSpecimen
- **Type status:** Paratype. **Occurrence:** occurrenceDetails: http://www.boldsystems.org/index.php/API_Public/specimen?ids=BC-ZSM-HYM-27770-C07; catalogNumber: BC-ZSM-HYM-27760-C07; recordNumber: BC-ZSM-HYM-27760-C07; recordedBy: J.S. Noyes; individualID: BC-ZSM-HYM-27770-C07; individualCount: 1; sex: male; lifeStage: adult; **Taxon:** scientificName: Tetrastichus mixtus; phylum: Arthropoda; class: Insecta; order: Hymenoptera; family: Eulophidae; genus: Tetrastichinae; **Location:** country: Romania; decimalLatitude: 47.2442; decimalLongitude: 27.4828; **Record Level:** type: PhysicalObject; language: en; basisOfRecord: PreservedSpecimen
- **Type status:** Paratype. **Occurrence:** occurrenceDetails: http://www.boldsystems.org/index.php/API_Public/specimen?ids=BC-ZSM-HYM-27770-C04; catalogNumber: BC-ZSM-HYM-27760-C04; recordNumber: BC-ZSM-HYM-27760-C04; recordedBy: J.S. Noyes; individualID: BC-ZSM-HYM-27770-C04; individualCount: 1; sex: male; lifeStage: adult; **Taxon:** scientificName: Tetrastichus mixtus; phylum: Arthropoda; class: Insecta; order: Hymenoptera; family: Eulophidae; genus: Tetrastichinae; **Location:** country: Romania; decimalLatitude: 47.2442; decimalLongitude: 27.4828; **Record Level:** type: PhysicalObject; language: en; basisOfRecord: PreservedSpecimen
- **Type status:** Paratype. **Occurrence:** occurrenceDetails: http://www.boldsystems.org/index.php/API_Public/specimen?ids=BC-ZSM-HYM-27770-C02; catalogNumber: BC-ZSM-HYM-27760-C02; recordNumber: BC-ZSM-HYM-27760-C02; recordedBy: J.S. Noyes; individualID: BC-ZSM-HYM-27770-C02; individualCount: 1; sex: male; lifeStage: adult; **Taxon:** scientificName: Tetrastichus mixtus; phylum: Arthropoda; class: Insecta; order: Hymenoptera; family: Eulophidae; genus: Tetrastichinae; **Location:** country: Romania; decimalLatitude: 47.2442; decimalLongitude: 27.4828; **Record Level:** type: PhysicalObject; language: en; basisOfRecord: PreservedSpecimen
- **Type status:** Paratype. **Occurrence:** occurrenceDetails: http://www.boldsystems.org/index.php/API_Public/specimen?ids=BC-ZSM-HYM-27770-C01; catalogNumber: BC-ZSM-HYM-27760-C01; recordNumber: BC-ZSM-HYM-27760-C01; recordedBy: J.S. Noyes; individualID: BC-ZSM-HYM-27770-C01; individualCount: 1; sex: female; lifeStage: adult; **Taxon:** scientificName: Tetrastichus mixtus; phylum: Arthropoda; class: Insecta; order: Hymenoptera; family: Eulophidae; genus: Tetrastichinae; **Location:** country: Romania; decimalLatitude: 47.2442; decimalLongitude: 27.4828; **Record Level:** type: PhysicalObject; language: en; basisOfRecord: PreservedSpecimen
- **Type status:** Paratype. **Occurrence:** occurrenceDetails: http://www.boldsystems.org/index.php/API_Public/specimen?ids=BC-ZSM-HYM-27770-B12; catalogNumber: BC-ZSM-HYM-27760-B12; recordNumber: BC-ZSM-HYM-27760-B12; recordedBy: J.S. Noyes; individualID: BC-ZSM-HYM-27770-B12; individualCount: 1; sex: male; lifeStage: adult; **Taxon:** scientificName: Tetrastichus mixtus; phylum: Arthropoda; class: Insecta; order: Hymenoptera; family: Eulophidae; genus: Tetrastichinae; **Location:** country: Romania; decimalLatitude: 47.2442; decimalLongitude: 27.4828; **Record Level:** type: PhysicalObject; language: en; basisOfRecord: PreservedSpecimen
- **Type status:** Paratype. **Occurrence:** occurrenceDetails: http://www.boldsystems.org/index.php/API_Public/specimen?ids=BC-ZSM-HYM-27770-B10; catalogNumber: BC-ZSM-HYM-27760-B10; recordNumber: BC-ZSM-HYM-27760-B10; recordedBy: J.S. Noyes; individualID: BC-ZSM-HYM-27770-B10; individualCount: 1; sex: female; lifeStage: adult; **Taxon:** scientificName: Tetrastichus mixtus; phylum: Arthropoda; class: Insecta; order: Hymenoptera; family: Eulophidae; genus: Tetrastichinae; **Location:** country: Romania; decimalLatitude: 47.2442; decimalLongitude: 27.4828; **Record Level:** type: PhysicalObject; language: en; basisOfRecord: PreservedSpecimen
- **Type status:** Paratype. **Occurrence:** occurrenceDetails: http://www.boldsystems.org/index.php/API_Public/specimen?ids=BC-ZSM-HYM-27770-B07; catalogNumber: BC-ZSM-HYM-27760-B07; recordNumber: BC-ZSM-HYM-27760-B07; recordedBy: J.S. Noyes; individualID: BC-ZSM-HYM-27770-B07; individualCount: 1; sex: female; lifeStage: adult; **Taxon:** scientificName: Tetrastichus mixtus; phylum: Arthropoda; class: Insecta; order: Hymenoptera; family: Eulophidae; genus: Tetrastichinae; **Location:** country: Romania; decimalLatitude: 47.2442; decimalLongitude: 27.4828; **Record Level:** type: PhysicalObject; language: en; basisOfRecord: PreservedSpecimen
- **Type status:** Paratype. **Occurrence:** occurrenceDetails: http://www.boldsystems.org/index.php/API_Public/specimen?ids=BC-ZSM-HYM-27770-B02; catalogNumber: BC-ZSM-HYM-27760-B02; recordNumber: BC-ZSM-HYM-27760-B02; recordedBy: J.S. Noyes; individualID: BC-ZSM-HYM-27770-B02; individualCount: 1; sex: female; lifeStage: adult; **Taxon:** scientificName: Tetrastichus mixtus; phylum: Arthropoda; class: Insecta; order: Hymenoptera; family: Eulophidae; genus: Tetrastichinae; **Location:** country: Romania; decimalLatitude: 47.2442; decimalLongitude: 27.4828; **Record Level:** type: PhysicalObject; language: en; basisOfRecord: PreservedSpecimen
- **Type status:** Paratype. **Occurrence:** occurrenceDetails: http://www.boldsystems.org/index.php/API_Public/specimen?ids=BC-ZSM-HYM-27770-A12; catalogNumber: BC-ZSM-HYM-27760-A12; recordNumber: BC-ZSM-HYM-27760-A12; recordedBy: J.S. Noyes; individualID: BC-ZSM-HYM-27770-A12; individualCount: 1; sex: female; lifeStage: adult; **Taxon:** scientificName: Tetrastichus mixtus; phylum: Arthropoda; class: Insecta; order: Hymenoptera; family: Eulophidae; genus: Tetrastichinae; **Location:** country: Romania; decimalLatitude: 47.2442; decimalLongitude: 27.4828; **Record Level:** type: PhysicalObject; language: en; basisOfRecord: PreservedSpecimen
- **Type status:** Paratype. **Occurrence:** occurrenceDetails: http://www.boldsystems.org/index.php/API_Public/specimen?ids=BC-ZSM-HYM-27768-H01; catalogNumber: BC-ZSM-HYM-27768-H01; recordNumber: BC-ZSM-HYM-27768-H01; recordedBy: J.S. Noyes; individualID: BC-ZSM-HYM-27768-H01; individualCount: 1; sex: female; lifeStage: adult; **Taxon:** scientificName: Tetrastichus mixtus; phylum: Arthropoda; class: Insecta; order: Hymenoptera; family: Eulophidae; genus: Tetrastichinae; **Location:** country: Romania; decimalLatitude: 47.244; decimalLongitude: 27.483; **Record Level:** type: PhysicalObject; language: en; basisOfRecord: PreservedSpecimen
- **Type status:** Paratype. **Occurrence:** occurrenceDetails: http://www.boldsystems.org/index.php/API_Public/specimen?ids=BC-ZSM-HYM-27768-G11; catalogNumber: BC-ZSM-HYM-27768-G11; recordNumber: BC-ZSM-HYM-27768-G11; recordedBy: J.S. Noyes; individualID: BC-ZSM-HYM-27768-G11; individualCount: 1; sex: female; lifeStage: adult; **Taxon:** scientificName: Tetrastichus mixtus; phylum: Arthropoda; class: Insecta; order: Hymenoptera; family: Eulophidae; genus: Tetrastichinae; **Location:** country: Romania; decimalLatitude: 47.244; decimalLongitude: 27.483; **Record Level:** type: PhysicalObject; language: en; basisOfRecord: PreservedSpecimen
- **Type status:** Paratype. **Occurrence:** occurrenceDetails: http://www.boldsystems.org/index.php/API_Public/specimen?ids=BC-ZSM-HYM-27768-G06; catalogNumber: BC-ZSM-HYM-27768-G06; recordNumber: BC-ZSM-HYM-27768-G06; recordedBy: J.S. Noyes; individualID: BC-ZSM-HYM-27768-G06; individualCount: 1; sex: male; lifeStage: adult; **Taxon:** scientificName: Tetrastichus mixtus; phylum: Arthropoda; class: Insecta; order: Hymenoptera; family: Eulophidae; genus: Tetrastichinae; **Location:** country: Romania; decimalLatitude: 47.244; decimalLongitude: 27.483; **Record Level:** type: PhysicalObject; language: en; basisOfRecord: PreservedSpecimen
- **Type status:** Paratype. **Occurrence:** occurrenceDetails: http://www.boldsystems.org/index.php/API_Public/specimen?ids=BC-ZSM-HYM-27768-G05; catalogNumber: BC-ZSM-HYM-27768-G05; recordNumber: BC-ZSM-HYM-27768-G05; recordedBy: J.S. Noyes; individualID: BC-ZSM-HYM-27768-G05; individualCount: 1; sex: female; lifeStage: adult; **Taxon:** scientificName: Tetrastichus mixtus; phylum: Arthropoda; class: Insecta; order: Hymenoptera; family: Eulophidae; genus: Tetrastichinae; **Location:** country: Romania; decimalLatitude: 47.244; decimalLongitude: 27.483; **Record Level:** type: PhysicalObject; language: en; basisOfRecord: PreservedSpecimen
- **Type status:** Paratype. **Occurrence:** occurrenceDetails: http://www.boldsystems.org/index.php/API_Public/specimen?ids=BC-ZSM-HYM-27768-G04; catalogNumber: BC-ZSM-HYM-27768-G04; recordNumber: BC-ZSM-HYM-27768-G04; recordedBy: J.S. Noyes; individualID: BC-ZSM-HYM-27768-G04; individualCount: 1; sex: female; lifeStage: adult; **Taxon:** scientificName: Tetrastichus mixtus; phylum: Arthropoda; class: Insecta; order: Hymenoptera; family: Eulophidae; genus: Tetrastichinae; **Location:** country: Romania; decimalLatitude: 47.244; decimalLongitude: 27.483; **Record Level:** type: PhysicalObject; language: en; basisOfRecord: PreservedSpecimen
- **Type status:** Paratype. **Occurrence:** occurrenceDetails: http://www.boldsystems.org/index.php/API_Public/specimen?ids=BC-ZSM-HYM-27768-G03; catalogNumber: BC-ZSM-HYM-27768-G03; recordNumber: BC-ZSM-HYM-27768-G03; recordedBy: J.S. Noyes; individualID: BC-ZSM-HYM-27768-G03; individualCount: 1; sex: female; lifeStage: adult; **Taxon:** scientificName: Tetrastichus mixtus; phylum: Arthropoda; class: Insecta; order: Hymenoptera; family: Eulophidae; genus: Tetrastichinae; **Location:** country: Romania; decimalLatitude: 47.244; decimalLongitude: 27.483; **Record Level:** type: PhysicalObject; language: en; basisOfRecord: PreservedSpecimen
- **Type status:** Paratype. **Occurrence:** occurrenceDetails: http://www.boldsystems.org/index.php/API_Public/specimen?ids=BC-ZSM-HYM-29751-A10; catalogNumber: BC-ZSM-HYM-29751-A10; recordNumber: BC-ZSM-HYM-29751-A10; recordedBy: C. Hansson; individualID: BC-ZSM-HYM-29751-A10; individualCount: 1; sex: female; lifeStage: adult; **Taxon:** scientificName: Tetrastichus mixtus; phylum: Arthropoda; class: Insecta; order: Hymenoptera; family: Eulophidae; genus: Tetrastichinae; **Location:** country: Sweden; decimalLatitude: 55.6861; decimalLongitude: 13.4611; **Record Level:** type: PhysicalObject; language: en; institutionCode: MZLU; basisOfRecord: PreservedSpecimen

#### Description

FEMALE holotype (Fig. 10). Body length 2.0 mm (paratypes 1.2–2.0 mm). *Head*. Width/length (dorsal view) 2.3, width/length (frontal view) 1.3, POL/OOL 2.4, widths head/mesosoma 1.0, mouth width/malar space 1.2, malar space/eye height 0.8. *Antenna*. Scape length/eye height 1.1, pedicel+flagellum length/mesosoma width 1.0, length/width F1, F2, F3 1.7, 1.8, 1.3, clava length/width 3.4, lengths pedicel/F1 0.8, lengths F1/F2 0.9, F1/F3 1.2, lengths F1, F2, F3/clava 0.4, 0.5, 0.4, widths F1/pedicel (dorsal view) 1.1, lengths antennal spicule/C3 0.6. *Mesosoma*. Length/width 1.3, mesoscutal mid-lobe length/width (width measured in anterior part) 0.8, mid-lobe with median groove in posterior ¾, with three adnotaular setae on each side, lengths mesoscutum/mesoscutellum (measured medially) 1.1, mesoscutellum length/width 0.8, length/width of enclosed space between submedian grooves 2.3, distance between SMG/distance between SMG and SLG 1.5, lengths dorsellum/propodeum (measured medially) 0.6, propodeum with weak reticulation, propodeal callus with four setae. *Fore wing*. Costal cell length/width 11.0, lengths costal cell/marginal vein 1.0, lengths marginal/stigmal veins 3.0. *Gaster*. Subcircular to ovate, length/width 1.2, lengths gaster/mesosoma 0.9, Gt_7_ length/width 0.4, length of longest cercal seta/next longest seta 1.5, longest cercal seta almost straight, ovipositor sheaths reaching to, but not protruding beyond, apex of Gt_7_.

Colour. Body with weak metallic green and golden tinges, scape dark yellowish-brown, pedicel and flagellum dark brown, tegulae dark brown, wings hyaline with veins yellowish-white, coxae concolorous with body, trochanters dark brown, femora dark brown with very weak metallic tinges, tibiae and tarsi yellowish-brown.

MALE. Body length 1.1–1.4 mm. *Head*. Width/length (dorsal view) 2.0, width/length (frontal view) 1.2, mouth width/malar space 1.3, eye height/malar space 1.4, widths head/mesosoma 1.2. *Antenna*. F1–F4 with basal whorls of setae, these setae subequal in length to flagellomere attached to, scape length/eye height 1.1, scape length/width 3.2, ventral plaque placed in central part of scape, lengths ventral plaque/scape 0.7, pedicel+flagellum length/mesosoma width 1.8, length/width F1, F2, F3, F4 2.0, 2.7, 2.7, 2.3, clava length/width 5.3, lengths pedicel/F1 0.8, lengths F1/F2 0.8, F1/F3 0.8, F1/F4 0.9, lengths F1, F2, F3, F4/clava 0.4, 0.5, 0.5, 0.4.

Colour. Similar to female, but antennal scape dark brown.

#### Diagnosis

Female with distance between posterior ocelli relatively long, POL/OOL= 2.4; F1 1.7× as long as wide; distance between SMG 1.5× distance SMG to SLG; ovipositor sheaths reaching to, but not protruding beyond, apex of Gt_7_. Male with antennal clava 5.3×, F4 2.3× and scape 3.2× as long as wide.

#### Distribution

Hungary, Romania, Sweden.

#### Ecology

##### Host

Unknown.

#### Notes

Holotype deposited in MZLU, paratypes in MZLU, NHM, ZSM.

#### Additional paratype (not barcoded)

1♀ “HUNGARY, Balaton-Zamárdi, 1.VIII.1961, A. Sundholm” (NHM, ex coll. Hedqvist).

### Tetrastichus murcia

(Walker, 1839)

F5FF6D28-3ABE-5A78-AF82-EF4B389E0299

Cirrospilus Walker 1839c:177. Lectotype ♂ in NHM, designated by Graham 1961:39, examined (Fig. 11). Combined to *Tetrastichus* by Walker 1848:146 and to *Aprostocetus* by Graham 1961:39 and back to *Tetrastichus* by Graham 1991:261.
Tetrastichus trichops Thomson 1878:282. Lectotype ♀ in MZLU, designated by Graham 1961:39, examined. Synonymised with *T. murcia* by Graham 1991:261.

#### Description

See Graham (1991).

#### Diagnosis

Female gaster with ovipositor sheaths projecting distinctly beyond apex of Gt_7_ , length of projecting part about equal to length of hind basitarsus; setae on eyes very long, 0.8× OD; distance between posterior ocelli relatively short, POL/OOL= 1.5–1.6.

#### Distribution

France, Germany, Sweden, United Kingdom (Graham 1991), and Russia (**new record**).

#### Ecology

##### Host

*Sargus* (*Geosargus)* sp. (Diptera: Stratiomyiidae), endoparasitoid emerging from pupa (Domenichini 1966). Larvae of *Sargus* develop in decaying matter, some in ulcers of elm trees, i.e. saprophagous (Johannsen 1922).

##### Material examined

Type material: lectotypes ♂ of *C. murcia* (type no. 5.1944) and ♀ of *T. trichops* (type no. 4869:1). Additional material (4♀): France 2♀ (GD), Russia 1♀ (MZLU), Sweden 1♀ (SMTP).

#### Material examined

### Tetrastichus solvae

Graham, 1991

6DB66AC1-BAEF-5CC7-9FD8-2E8692DE344C

Tetrastichus solvae Graham 1991:266. Holotype ♀ in NHM, examined (Fig. 12).

#### Description

See Graham (1991).

#### Diagnosis

Female with antennal funicle stout, F1 and F2 both 1.1–1.25× as long as wide, pedicel 1.35–1.5× as long as F1; mid-lobe of mesoscutum without a median groove or with the groove indicated only near mesoscutellum.

#### Distribution

Spain (Canary Islands, Tenerife) (Graham 1991).

#### Ecology

##### Host

*Xylomyia cabrerae* (Becker) (Diptera: Xylomyiidae) in twigs of *Euphorbia canariensis* (Graham 1991).

#### Material examined

Type material: holotype ♀ (NHM, type no. 5.3629).

### Tetrastichus tacitus sp. n.

3AA58A4A-AD81-5B3B-BF97-C4ACD5C2DA79

urn:lsid:zoobank.org:act:2EAC3BB9-DC8F-4B7A-9C19-302D09F09D9D

#### Materials

- **Type status:** Holotype. **Occurrence:** occurrenceDetails: http://www.boldsystems.org/index.php/API_Public/specimen?ids=BC-ZSM-HYM-22524-B10; catalogNumber: BC-ZSM-HYM-22524-B10; recordNumber: BC-ZSM-HYM-22524-B10; recordedBy: C. Hansson; individualID: BC-ZSM-HYM-22524-B10; individualCount: 1; sex: female; lifeStage: adult; **Taxon:** scientificName: Tetrastichus tacitus; phylum: Arthropoda; class: Insecta; order: Hymenoptera; family: Eulophidae; genus: Tetrastichinae; taxonRemarks: Holotype deposited in MZLU; **Location:** country: Hungary; decimalLatitude: 47.22; decimalLongitude: 16.313; **Record Level:** type: PhysicalObject; language: en; institutionCode: MZLU; basisOfRecord: PreservedSpecimen
- **Type status:** Paratype. **Occurrence:** occurrenceDetails: http://www.boldsystems.org/index.php/API_Public/specimen?ids=BC-ZSM-HYM-13565-F03; catalogNumber: BC-ZSM-HYM-13565-F03; recordNumber: BC-ZSM-HYM-13565-F03; recordedBy: C. Hansson; individualID: BC-ZSM-HYM-13565-F03; individualCount: 1; sex: female; lifeStage: adult; **Taxon:** scientificName: Tetrastichus tacitus; phylum: Arthropoda; class: Insecta; order: Hymenoptera; family: Eulophidae; genus: Tetrastichinae; **Location:** country: Hungary; decimalLatitude: 46.903; decimalLongitude: 16.454; **Record Level:** type: PhysicalObject; language: en; basisOfRecord: PreservedSpecimen
- **Type status:** Paratype. **Occurrence:** occurrenceDetails: http://www.boldsystems.org/index.php/API_Public/specimen?ids=BC-ZSM-HYM-22523-G01; catalogNumber: BC-ZSM-HYM-22523-G01; recordNumber: BC-ZSM-HYM-22523-G01; recordedBy: O.Popovici; individualID: BC-ZSM-HYM-22523-G01; individualCount: 1; sex: female; lifeStage: adult; **Taxon:** scientificName: Tetrastichus tacitus; phylum: Arthropoda; class: Insecta; order: Hymenoptera; family: Eulophidae; genus: Tetrastichinae; **Location:** country: Romania; decimalLatitude: 46.594; decimalLongitude: 27.353; **Record Level:** type: PhysicalObject; language: en; basisOfRecord: PreservedSpecimen
- **Type status:** Paratype. **Occurrence:** occurrenceDetails: http://www.boldsystems.org/index.php/API_Public/specimen?ids=BC-ZSM-HYM-22524-A10; catalogNumber: BC-ZSM-HYM-22524-A10; recordNumber: BC-ZSM-HYM-22524-A10; recordedBy: C. Hansson; individualID: BC-ZSM-HYM-22524-A10; individualCount: 1; sex: male; lifeStage: adult; **Taxon:** scientificName: Tetrastichus tacitus; phylum: Arthropoda; class: Insecta; order: Hymenoptera; family: Eulophidae; genus: Tetrastichinae; **Location:** country: Hungary; decimalLatitude: 47.381; decimalLongitude: 16.552; **Record Level:** type: PhysicalObject; language: en; basisOfRecord: PreservedSpecimen
- **Type status:** Paratype. **Occurrence:** occurrenceDetails: http://www.boldsystems.org/index.php/API_Public/specimen?ids=BC-ZSM-HYM-22524-B02; catalogNumber: BC-ZSM-HYM-22524-B02; recordNumber: BC-ZSM-HYM-22524-B02; recordedBy: C. Hansson; individualID: BC-ZSM-HYM-22524-B02; individualCount: 1; sex: male; lifeStage: adult; **Taxon:** scientificName: Tetrastichus tacitus; phylum: Arthropoda; class: Insecta; order: Hymenoptera; family: Eulophidae; genus: Tetrastichinae; **Location:** country: Hungary; decimalLatitude: 47.22; decimalLongitude: 16.313; **Record Level:** type: PhysicalObject; language: en; basisOfRecord: PreservedSpecimen
- **Type status:** Paratype. **Occurrence:** occurrenceDetails: http://www.boldsystems.org/index.php/API_Public/specimen?ids=BC-ZSM-HYM-22524-B09; catalogNumber: BC-ZSM-HYM-22524-B09; recordNumber: BC-ZSM-HYM-22524-B09; recordedBy: C. Hansson; individualID: BC-ZSM-HYM-22524-B09; individualCount: 1; sex: female; lifeStage: adult; **Taxon:** scientificName: Tetrastichus tacitus; phylum: Arthropoda; class: Insecta; order: Hymenoptera; family: Eulophidae; genus: Tetrastichinae; **Location:** country: Hungary; decimalLatitude: 47.22; decimalLongitude: 16.313; **Record Level:** type: PhysicalObject; language: en; basisOfRecord: PreservedSpecimen
- **Type status:** Paratype. **Occurrence:** occurrenceDetails: http://www.boldsystems.org/index.php/API_Public/specimen?ids=BC-ZSM-HYM-22524-D03; catalogNumber: BC-ZSM-HYM-22524-D03; recordNumber: BC-ZSM-HYM-22524-D03; recordedBy: J. Straka; individualID: BC-ZSM-HYM-22524-D03; individualCount: 1; sex: female; lifeStage: adult; **Taxon:** scientificName: Tetrastichus tacitus; phylum: Arthropoda; class: Insecta; order: Hymenoptera; family: Eulophidae; genus: Tetrastichinae; **Location:** decimalLatitude: 49.719; decimalLongitude: 10.158; **Record Level:** type: PhysicalObject; language: en; basisOfRecord: PreservedSpecimen
- **Type status:** Paratype. **Occurrence:** occurrenceDetails: http://www.boldsystems.org/index.php/API_Public/specimen?ids=BC-ZSM-HYM-27770-A06; catalogNumber: BC-ZSM-HYM-27760-A06; recordNumber: BC-ZSM-HYM-27760-A06; recordedBy: J.S. Noyes; individualID: BC-ZSM-HYM-27770-A06; individualCount: 1; sex: female; lifeStage: adult; **Taxon:** scientificName: Tetrastichus tacitus; phylum: Arthropoda; class: Insecta; order: Hymenoptera; family: Eulophidae; genus: Tetrastichinae; **Location:** country: Romania; decimalLatitude: 46.9853; decimalLongitude: 27.5847; **Record Level:** type: PhysicalObject; language: en; basisOfRecord: PreservedSpecimen
- **Type status:** Paratype. **Occurrence:** occurrenceDetails: http://www.boldsystems.org/index.php/API_Public/specimen?ids=BC-ZSM-HYM-22524-A11; catalogNumber: BC-ZSM-HYM-22524-A11; recordNumber: BC-ZSM-HYM-22524-A11; recordedBy: C. Hansson; individualID: BC-ZSM-HYM-22524-A11; individualCount: 1; sex: male; lifeStage: adult; **Taxon:** scientificName: Tetrastichus tacitus; phylum: Arthropoda; class: Insecta; order: Hymenoptera; family: Eulophidae; genus: Tetrastichinae; **Location:** country: Hungary; decimalLatitude: 47.22; decimalLongitude: 16.313; **Record Level:** type: PhysicalObject; language: en; basisOfRecord: PreservedSpecimen
- **Type status:** Paratype. **Occurrence:** occurrenceDetails: http://www.boldsystems.org/index.php/API_Public/specimen?ids=BC-ZSM-HYM-22524-A12; catalogNumber: BC-ZSM-HYM-22524-A12; recordNumber: BC-ZSM-HYM-22524-A12; recordedBy: C. Hansson; individualID: BC-ZSM-HYM-22524-A12; individualCount: 1; sex: male; lifeStage: adult; **Taxon:** scientificName: Tetrastichus tacitus; phylum: Arthropoda; class: Insecta; order: Hymenoptera; family: Eulophidae; genus: Tetrastichinae; **Location:** country: Hungary; decimalLatitude: 47.22; decimalLongitude: 16.313; **Record Level:** type: PhysicalObject; language: en; basisOfRecord: PreservedSpecimen
- **Type status:** Paratype. **Occurrence:** occurrenceDetails: http://www.boldsystems.org/index.php/API_Public/specimen?ids=BC-ZSM-HYM-22524-B03; catalogNumber: BC-ZSM-HYM-22524-B03; recordNumber: BC-ZSM-HYM-22524-B03; recordedBy: C. Hansson; individualID: BC-ZSM-HYM-22524-B03; individualCount: 1; sex: female; lifeStage: adult; **Taxon:** scientificName: Tetrastichus tacitus; phylum: Arthropoda; class: Insecta; order: Hymenoptera; family: Eulophidae; genus: Tetrastichinae; **Location:** country: Hungary; decimalLatitude: 47.22; decimalLongitude: 16.313; **Record Level:** type: PhysicalObject; language: en; basisOfRecord: PreservedSpecimen
- **Type status:** Paratype. **Occurrence:** occurrenceDetails: http://www.boldsystems.org/index.php/API_Public/specimen?ids=BC-ZSM-HYM-22524-B05; catalogNumber: BC-ZSM-HYM-22524-B05; recordNumber: BC-ZSM-HYM-22524-B05; recordedBy: C. Hansson; individualID: BC-ZSM-HYM-22524-B05; individualCount: 1; sex: male; lifeStage: adult; **Taxon:** scientificName: Tetrastichus tacitus; phylum: Arthropoda; class: Insecta; order: Hymenoptera; family: Eulophidae; genus: Tetrastichinae; **Location:** country: Hungary; decimalLatitude: 47.22; decimalLongitude: 16.313; **Record Level:** type: PhysicalObject; language: en; basisOfRecord: PreservedSpecimen
- **Type status:** Paratype. **Occurrence:** occurrenceDetails: http://www.boldsystems.org/index.php/API_Public/specimen?ids=BC-ZSM-HYM-22524-B08; catalogNumber: BC-ZSM-HYM-22524-B08; recordNumber: BC-ZSM-HYM-22524-B08; recordedBy: C. Hansson; individualID: BC-ZSM-HYM-22524-B08; individualCount: 1; sex: male; lifeStage: adult; **Taxon:** scientificName: Tetrastichus tacitus; phylum: Arthropoda; class: Insecta; order: Hymenoptera; family: Eulophidae; genus: Tetrastichinae; **Location:** country: Hungary; decimalLatitude: 47.22; decimalLongitude: 16.313; **Record Level:** type: PhysicalObject; language: en; basisOfRecord: PreservedSpecimen
- **Type status:** Paratype. **Occurrence:** occurrenceDetails: http://www.boldsystems.org/index.php/API_Public/specimen?ids=BC-ZSM-HYM-22524-B11; catalogNumber: BC-ZSM-HYM-22524-B11; recordNumber: BC-ZSM-HYM-22524-B11; recordedBy: C. Hansson; individualID: BC-ZSM-HYM-22524-B11; individualCount: 1; sex: male; lifeStage: adult; **Taxon:** scientificName: Tetrastichus tacitus; phylum: Arthropoda; class: Insecta; order: Hymenoptera; family: Eulophidae; genus: Tetrastichinae; **Location:** country: Hungary; decimalLatitude: 47.22; decimalLongitude: 16.313; **Record Level:** type: PhysicalObject; language: en; basisOfRecord: PreservedSpecimen

#### Description

FEMALE holotype (Fig. 13). Body length 1.7 mm (paratypes 1.5–1.7 mm). *Head*. Width/length (dorsal view) 2.2, width/length (frontal view) 1.3, POL/OOL 2.1, widths head/mesosoma 1.1, mouth width/malar space 1.1, malar space/eye height 0.7. *Antenna*. Scape length/eye height 1.0, pedicel+flagellum length/mesosoma width 1.2, length/width F1, F2, F3 2.0, 1.8, 1.2, clava length/width 2.7, lengths pedicel/F1 0.8, lengths F1/F2 1.1, F1/F3 1.3, lengths F1, F2, F3/clava 0.5, 0.5, 0.4, widths F1/pedicel (dorsal view) 1.0, lengths antennal spicule/C3 0.5. *Mesosoma*. Length/width 1.4, mesoscutal mid-lobe length/width (width measured in anterior part) 0.9, mid-lobe with median groove in posterior ⅔, with three adnotaular setae on each side, lengths mesoscutum/mesoscutellum (measured medially) 1.2, mesoscutellum length/width 0.8, length/width of enclosed space between submedian grooves 2.2, distance between SMG/distance between SMG and SLG 1.7, lengths dorsellum/propodeum (measured medially) 0.5, propodeum with strong reticulation, propodeal callus with three setae. *Fore wing*. Costal cell length/width 8.7, lengths costal cell/marginal vein 0.9, lengths marginal/stigmal veins 3.1. *Gaster*. Subcircular to ovate, length/width 1.5, lengths gaster/mesosoma 1.1, Gt_7_ length/width 0.5, length longest cercal seta/next longest seta 1.8, longest cercal seta almost straight, ovipositor sheaths projecting distinctly beyond apex of Gt_7_.

Colour. Body with weak metallic green tinges, scape, pedicel and flagellum dark brown, tegulae dark brown, wings hyaline with veins yellowish-brown to brown, coxae and femora concolorous with body, trochanters dark brown, tibiae yellowish-brown, fore tarsus pale brown, mid and hind tarsi yellowish-brown with T4 dark brown.

Variation. Scape pale brown in some paratypes.

MALE. Body length 1.2–1.5 mm. *Head*. Width/length (dorsal view) 1.8, width/length (frontal view) 1.1, mouth width/malar space 1.1, eye height/malar space 1.4, widths head/mesosoma 0.9. *Antenna*. F1–F4 with basal whorls of setae, these setae reaching beyond apex of flagellomere attached to, scape length/eye height 0.9, scape length/width 2.9, ventral plaque placed in central part of scape, lengths ventral plaque/scape 0.6, pedicel+flagellum length/mesosoma width 1.6, length/width F1, F2, F3, F4 2.0, 2.8, 2.8, 2.7, clava length/width 6.0, lengths pedicel/F1 1.0, lengths F1/F2 0.7, F1/F3 0.7, F1/F4 0.8, lengths F1, F2, F3, F4/clava 0.3, 0.4, 0.4, 0.4.

Colour. Similar to female but scape always dark brown and tibiae brown in some specimens.

#### Diagnosis

Female with antennal clava (incl. spicule) 2.7× as long as wide; ovipositor sheaths protruding beyond apex of Gt_7_. Male with antennal clava 6.0×, F4 2.3× and scape 2.9× as long as wide.

#### Distribution

Germany, Hungary, Romania.

#### Ecology

##### Host

Unknown.

#### Notes

Holotype deposited in MZLU, paratypes in MZLU, NHM, ZSM.

### Tetrastichus tartus sp. n.

DD5138B7-4BEE-53CA-8F2A-D005A62EDC1B

urn:lsid:zoobank.org:act:998D49C4-0B49-4B9E-91ED-7C8EF3CD670E

#### Materials

- **Type status:** Holotype. **Occurrence:** occurrenceDetails: http://www.boldsystems.org/index.php/API_Public/specimen?ids=BC-ZSM-HYM-27768-C04; catalogNumber: BC-ZSM-HYM-27768-C04; recordNumber: BC-ZSM-HYM-27768-C04; recordedBy: J.S. Noyes; individualID: BC-ZSM-HYM-27768-C04; individualCount: 1; sex: female; lifeStage: adult; **Taxon:** scientificName: Tetrastichus tartus; phylum: Arthropoda; class: Insecta; order: Hymenoptera; family: Eulophidae; genus: Tetrastichinae; taxonRemarks: Holotype deposited in NHM; **Location:** country: Romania; decimalLatitude: 47.011; decimalLongitude: 27.603; **Record Level:** type: PhysicalObject; language: en; institutionCode: NHM; basisOfRecord: PreservedSpecimen

#### Description

FEMALE holotype (Fig. 14). Body length 1.8 mm. *Head*. Width/length (dorsal view) 2.4, width/length (frontal view) 1.2, POL/OOL 1.9, widths head/mesosoma 1.1, mouth width/malar space 1.1, malar space/eye height 0.7. *Antenna*. Scape length/eye height 0.9, pedicel+flagellum length/mesosoma width 1.2, length/width F1, F2, F3 2.0, 2.3, 1.8, clava length/width 3.3, lengths pedicel/F1 0.8, lengths F1/F2 0.9, F1/F3 1.1, lengths F1, F2, F3/clava 0.5, 0.6, 0.4, widths F1/pedicel (dorsal view) 1.1, lengths antennal spicule/C3 0.4. *Mesosoma*. Length/width 1.5, mesoscutal mid-lobe length/width (width measured in anterior part) 0.9, mid-lobe with median groove in posterior ⅔, with four adnotaular setae on each side, lengths mesoscutum/mesoscutellum (measured medially) 1.3, mesoscutellum length/width 0.9, length/width of enclosed space between submedian grooves 2.1, distance between SMG/distance between SMG and SLG 2.0, lengths dorsellum/propodeum (measured medially) 0.5, propodeum with weak reticulation, propodeal callus with four setae. *Fore wing*. Costal cell length/width 12.0, lengths costal cell/marginal vein 1.0, lengths marginal/stigmal veins 3.3. *Gaster*. Subcircular to ovate, length/width 1.5, lengths gaster/mesosoma 1.1, Gt_7_ length/width 0.5, length of longest cercal seta/next longest seta 1.8, longest seta kinked in apical ⅓, ovipositor sheaths not reaching apex of Gt_7_.

Colour. Body with weak metallic green and coppery tinges, scape yellowish-brown with dorsal edge brown, pedicel and flagellum dark brown, tegulae blackish, wings hyaline with veins yellowish-brown, coxae and femora concolorous with body, trochanters dark brown, tibiae yellowish-brown, tarsi yellowish-brown with T4 brown.

MALE. Unknown.

#### Diagnosis

Distance between submedian grooves 2.0× distance between submedian and sublateral grooves; antennal clava (incl. spicule) long, 3.3× as long as wide; ovipositor sheaths not protruding beyond apex of Gt_7_.

#### Distribution

Romania.

#### Ecology

##### Host

Unknown.

#### Notes

Holotype deposited in NHM.

### Tetrastichus hylotomarum group

D9566E82-100C-564E-A27F-C3564BCE35FE

#### Diagnosis

Body bright metallic and shiny; ovipositor sheaths retracted in dry specimens and do not reach apex of Gt_7_ (as in Fig. 2b); female gaster circular to short ovate; male flagellomeres 1–4 without externo-dorsal, sub-basal compact whorls of long setae (e.g. Fig. 15c); usually relatively large specimens.

### Tetrastichus argei sp. n.

C11C7B62-CFA6-56BE-9684-EED05E805B93

urn:lsid:zoobank.org:act:DBDA353F-05C8-4374-A96E-CD5D62A0C11F

#### Materials

- **Type status:** Holotype. **Occurrence:** occurrenceDetails: http://www.boldsystems.org/index.php/API_Public/specimen?ids=BC-ZSM-HYM-20721-E05; catalogNumber: BC-ZSM-HYM-20721-E05; recordNumber: BC-ZSM-HYM-20721-E05; recordedBy: C. Hansson; individualID: BC-ZSM-HYM-20721-E05; individualCount: 1; sex: female; lifeStage: adult; **Taxon:** scientificName: Tetrastichus argei; phylum: Arthropoda; class: Insecta; order: Hymenoptera; family: Eulophidae; genus: Tetrastichinae; taxonRemarks: Holotype deposited in MZLU; **Location:** country: Sweden; decimalLatitude: 55.683; decimalLongitude: 13.5; **Record Level:** type: PhysicalObject; language: en; institutionCode: MZLU; basisOfRecord: PreservedSpecimen
- **Type status:** Paratype. **Occurrence:** occurrenceDetails: http://www.boldsystems.org/index.php/API_Public/specimen?ids=BC-ZSM-HYM-22523-C07; catalogNumber: BC-ZSM-HYM-22523-C07; recordNumber: BC-ZSM-HYM-22523-C07; recordedBy: Swedish Malaise Trap Project; individualID: BC-ZSM-HYM-22523-C07; individualCount: 1; sex: female; lifeStage: adult; **Taxon:** scientificName: Tetrastichus argei; phylum: Arthropoda; class: Insecta; order: Hymenoptera; family: Eulophidae; genus: Tetrastichinae; **Location:** country: Sweden; decimalLatitude: 57.322; decimalLongitude: 18.203; **Record Level:** type: PhysicalObject; language: en; institutionCode: SMTP; basisOfRecord: PreservedSpecimen
- **Type status:** Paratype. **Occurrence:** occurrenceDetails: http://www.boldsystems.org/index.php/API_Public/specimen?ids=BC-ZSM-HYM-21587-A01; catalogNumber: BC-ZSM-HYM-21587-A01; recordNumber: BC-ZSM-HYM-21587-A01; recordedBy: C.Hansson; individualID: BC-ZSM-HYM-21587-A01; individualCount: 1; sex: female; lifeStage: adult; **Taxon:** scientificName: Tetrastichus argei; phylum: Arthropoda; class: Insecta; order: Hymenoptera; family: Eulophidae; genus: Tetrastichinae; **Location:** country: Sweden; decimalLatitude: 56.633; decimalLongitude: 16.623; **Record Level:** type: PhysicalObject; language: en; institutionCode: MZLU; basisOfRecord: PreservedSpecimen
- **Type status:** Paratype. **Occurrence:** occurrenceDetails: http://www.boldsystems.org/index.php/API_Public/specimen?ids=BC-ZSM-HYM-22526-F12; catalogNumber: BC-ZSM-HYM-22526-F12; recordNumber: BC-ZSM-HYM-22526-F12; recordedBy: S. Schmidt; individualID: BC-ZSM-HYM-22526-F12; individualCount: 1; sex: female; lifeStage: adult; **Taxon:** scientificName: Tetrastichus argei; phylum: Arthropoda; class: Insecta; order: Hymenoptera; family: Eulophidae; genus: Tetrastichinae; **Location:** country: Sweden; decimalLatitude: 56.609; decimalLongitude: 16.508; **Record Level:** type: PhysicalObject; language: en; institutionCode: ZSM; basisOfRecord: PreservedSpecimen
- **Type status:** Paratype. **Occurrence:** occurrenceDetails: http://www.boldsystems.org/index.php/API_Public/specimen?ids=BC-ZSM-HYM-25461-C06; catalogNumber: BC-ZSM-HYM-25461-C06; recordNumber: BC-ZSM-HYM-25461-C06; recordedBy: P. Benander; individualID: BC-ZSM-HYM-25461-C06; individualCount: 1; sex: female; lifeStage: adult; **Taxon:** scientificName: Tetrastichus argei; phylum: Arthropoda; class: Insecta; order: Hymenoptera; family: Eulophidae; genus: Tetrastichinae; **Location:** country: Sweden; decimalLatitude: 56.294; decimalLongitude: 12.495; **Record Level:** type: PhysicalObject; language: en; basisOfRecord: PreservedSpecimen
- **Type status:** Paratype. **Occurrence:** occurrenceDetails: http://www.boldsystems.org/index.php/API_Public/specimen?ids=BC-ZSM-HYM-27493-G09; catalogNumber: BC-ZSM-HYM-27493-G09; recordNumber: BC-ZSM-HYM-27493-G09; recordedBy: SMTP; individualID: BC-ZSM-HYM-27493-G09; individualCount: 1; sex: female; lifeStage: adult; **Taxon:** scientificName: Tetrastichus argei; phylum: Arthropoda; class: Insecta; order: Hymenoptera; family: Eulophidae; genus: Tetrastichinae; **Location:** country: Sweden; decimalLatitude: 64.114; decimalLongitude: 19.363; **Record Level:** type: PhysicalObject; language: en; institutionCode: SMTP; basisOfRecord: PreservedSpecimen
- **Type status:** Paratype. **Occurrence:** occurrenceDetails: http://www.boldsystems.org/index.php/API_Public/specimen?ids=BC-ZSM-HYM-25461-C07; catalogNumber: BC-ZSM-HYM-25461-C07; recordNumber: BC-ZSM-HYM-25461-C07; recordedBy: P. Benander; individualID: BC-ZSM-HYM-25461-C07; individualCount: 1; lifeStage: adult; **Taxon:** scientificName: Tetrastichus argei; phylum: Arthropoda; class: Insecta; order: Hymenoptera; family: Eulophidae; genus: Tetrastichinae; **Location:** country: Sweden; decimalLatitude: 56.294; decimalLongitude: 12.495; **Record Level:** type: PhysicalObject; language: en; basisOfRecord: PreservedSpecimen
- **Type status:** Paratype. **Occurrence:** occurrenceDetails: http://www.boldsystems.org/index.php/API_Public/specimen?ids=BC-ZSM-HYM-25461-C08; catalogNumber: BC-ZSM-HYM-25461-C08; recordNumber: BC-ZSM-HYM-25461-C08; recordedBy: P. Benander; individualID: BC-ZSM-HYM-25461-C08; individualCount: 1; lifeStage: adult; **Taxon:** scientificName: Tetrastichus argei; phylum: Arthropoda; class: Insecta; order: Hymenoptera; family: Eulophidae; genus: Tetrastichinae; **Location:** country: Sweden; decimalLatitude: 56.294; decimalLongitude: 12.495; **Record Level:** type: PhysicalObject; language: en; basisOfRecord: PreservedSpecimen
- **Type status:** Paratype. **Occurrence:** occurrenceDetails: http://www.boldsystems.org/index.php/API_Public/specimen?ids=BC-ZSM-HYM-25461-C09; catalogNumber: BC-ZSM-HYM-25461-C09; recordNumber: BC-ZSM-HYM-25461-C09; recordedBy: P. Benander; individualID: BC-ZSM-HYM-25461-C09; individualCount: 1; lifeStage: adult; **Taxon:** scientificName: Tetrastichus argei; phylum: Arthropoda; class: Insecta; order: Hymenoptera; family: Eulophidae; genus: Tetrastichinae; **Location:** country: Sweden; decimalLatitude: 56.294; decimalLongitude: 12.495; **Record Level:** type: PhysicalObject; language: en; basisOfRecord: PreservedSpecimen
- **Type status:** Paratype. **Occurrence:** occurrenceDetails: http://www.boldsystems.org/index.php/API_Public/specimen?ids=BC-ZSM-HYM-25461-C10; catalogNumber: BC-ZSM-HYM-25461-C10; recordNumber: BC-ZSM-HYM-25461-C10; recordedBy: P. Benander; individualID: BC-ZSM-HYM-25461-C10; individualCount: 1; lifeStage: adult; **Taxon:** scientificName: Tetrastichus argei; phylum: Arthropoda; class: Insecta; order: Hymenoptera; family: Eulophidae; genus: Tetrastichinae; **Location:** country: Sweden; decimalLatitude: 56.294; decimalLongitude: 12.495; **Record Level:** type: PhysicalObject; language: en; basisOfRecord: PreservedSpecimen
- **Type status:** Paratype. **Occurrence:** occurrenceDetails: http://www.boldsystems.org/index.php/API_Public/specimen?ids=BC-ZSM-HYM-25461-C11; catalogNumber: BC-ZSM-HYM-25461-C11; recordNumber: BC-ZSM-HYM-25461-C11; recordedBy: P. Benander; individualID: BC-ZSM-HYM-25461-C11; individualCount: 1; lifeStage: adult; **Taxon:** scientificName: Tetrastichus argei; phylum: Arthropoda; class: Insecta; order: Hymenoptera; family: Eulophidae; genus: Tetrastichinae; **Location:** country: Sweden; decimalLatitude: 56.294; decimalLongitude: 12.495; **Record Level:** type: PhysicalObject; language: en; basisOfRecord: PreservedSpecimen
- **Type status:** Paratype. **Occurrence:** occurrenceDetails: http://www.boldsystems.org/index.php/API_Public/specimen?ids=BC-ZSM-HYM-25461-C12; catalogNumber: BC-ZSM-HYM-25461-C12; recordNumber: BC-ZSM-HYM-25461-C12; recordedBy: P. Benander; individualID: BC-ZSM-HYM-25461-C12; individualCount: 1; lifeStage: adult; **Taxon:** scientificName: Tetrastichus argei; phylum: Arthropoda; class: Insecta; order: Hymenoptera; family: Eulophidae; genus: Tetrastichinae; **Location:** country: Sweden; decimalLatitude: 56.294; decimalLongitude: 12.495; **Record Level:** type: PhysicalObject; language: en; basisOfRecord: PreservedSpecimen
- **Type status:** Paratype. **Occurrence:** occurrenceDetails: http://www.boldsystems.org/index.php/API_Public/specimen?ids=BC-ZSM-HYM-25461-D01; catalogNumber: BC-ZSM-HYM-25461-D01; recordNumber: BC-ZSM-HYM-25461-D01; recordedBy: P. Benander; individualID: BC-ZSM-HYM-25461-D01; individualCount: 1; lifeStage: adult; **Taxon:** scientificName: Tetrastichus argei; phylum: Arthropoda; class: Insecta; order: Hymenoptera; family: Eulophidae; genus: Tetrastichinae; **Location:** country: Sweden; decimalLatitude: 56.294; decimalLongitude: 12.495; **Record Level:** type: PhysicalObject; language: en; basisOfRecord: PreservedSpecimen
- **Type status:** Paratype. **Occurrence:** occurrenceDetails: http://www.boldsystems.org/index.php/API_Public/specimen?ids=BC-ZSM-HYM-25461-D02; catalogNumber: BC-ZSM-HYM-25461-D02; recordNumber: BC-ZSM-HYM-25461-D02; recordedBy: P. Benander; individualID: BC-ZSM-HYM-25461-D02; individualCount: 1; lifeStage: adult; **Taxon:** scientificName: Tetrastichus argei; phylum: Arthropoda; class: Insecta; order: Hymenoptera; family: Eulophidae; genus: Tetrastichinae; **Location:** country: Sweden; decimalLatitude: 56.294; decimalLongitude: 12.495; **Record Level:** type: PhysicalObject; language: en; basisOfRecord: PreservedSpecimen
- **Type status:** Paratype. **Occurrence:** occurrenceDetails: http://www.boldsystems.org/index.php/API_Public/specimen?ids=BC-ZSM-HYM-25461-D03; catalogNumber: BC-ZSM-HYM-25461-D03; recordNumber: BC-ZSM-HYM-25461-D03; recordedBy: P. Benander; individualID: BC-ZSM-HYM-25461-D03; individualCount: 1; lifeStage: adult; **Taxon:** scientificName: Tetrastichus argei; phylum: Arthropoda; class: Insecta; order: Hymenoptera; family: Eulophidae; genus: Tetrastichinae; **Location:** country: Sweden; decimalLatitude: 56.294; decimalLongitude: 12.495; **Record Level:** type: PhysicalObject; language: en; basisOfRecord: PreservedSpecimen
- **Type status:** Paratype. **Occurrence:** occurrenceDetails: http://www.boldsystems.org/index.php/API_Public/specimen?ids=BC-ZSM-HYM-25461-D04; catalogNumber: BC-ZSM-HYM-25461-D04; recordNumber: BC-ZSM-HYM-25461-D04; recordedBy: P. Benander; individualID: BC-ZSM-HYM-25461-D04; individualCount: 1; lifeStage: adult; **Taxon:** scientificName: Tetrastichus argei; phylum: Arthropoda; class: Insecta; order: Hymenoptera; family: Eulophidae; genus: Tetrastichinae; **Location:** country: Sweden; decimalLatitude: 56.294; decimalLongitude: 12.495; **Record Level:** type: PhysicalObject; language: en; basisOfRecord: PreservedSpecimen
- **Type status:** Paratype. **Occurrence:** occurrenceDetails: http://www.boldsystems.org/index.php/API_Public/specimen?ids=BC-ZSM-HYM-25461-D05; catalogNumber: BC-ZSM-HYM-25461-D05; recordNumber: BC-ZSM-HYM-25461-D05; recordedBy: P. Benander; individualID: BC-ZSM-HYM-25461-D05; individualCount: 1; lifeStage: adult; **Taxon:** scientificName: Tetrastichus argei; phylum: Arthropoda; class: Insecta; order: Hymenoptera; family: Eulophidae; genus: Tetrastichinae; **Location:** country: Sweden; decimalLatitude: 56.294; decimalLongitude: 12.495; **Record Level:** type: PhysicalObject; language: en; basisOfRecord: PreservedSpecimen
- **Type status:** Paratype. **Occurrence:** occurrenceDetails: http://www.boldsystems.org/index.php/API_Public/specimen?ids=BC-ZSM-HYM-25461-D06; catalogNumber: BC-ZSM-HYM-25461-D06; recordNumber: BC-ZSM-HYM-25461-D06; recordedBy: P. Benander; individualID: BC-ZSM-HYM-25461-D06; individualCount: 1; lifeStage: adult; **Taxon:** scientificName: Tetrastichus argei; phylum: Arthropoda; class: Insecta; order: Hymenoptera; family: Eulophidae; genus: Tetrastichinae; **Location:** country: Sweden; decimalLatitude: 56.294; decimalLongitude: 12.495; **Record Level:** type: PhysicalObject; language: en; basisOfRecord: PreservedSpecimen
- **Type status:** Paratype. **Occurrence:** occurrenceDetails: http://www.boldsystems.org/index.php/API_Public/specimen?ids=BC-ZSM-HYM-25461-D07; catalogNumber: BC-ZSM-HYM-25461-D07; recordNumber: BC-ZSM-HYM-25461-D07; recordedBy: P. Benander; individualID: BC-ZSM-HYM-25461-D07; individualCount: 1; lifeStage: adult; **Taxon:** scientificName: Tetrastichus argei; phylum: Arthropoda; class: Insecta; order: Hymenoptera; family: Eulophidae; genus: Tetrastichinae; **Location:** country: Sweden; decimalLatitude: 56.294; decimalLongitude: 12.495; **Record Level:** type: PhysicalObject; language: en; basisOfRecord: PreservedSpecimen
- **Type status:** Paratype. **Occurrence:** occurrenceDetails: http://www.boldsystems.org/index.php/API_Public/specimen?ids=BC-ZSM-HYM-25461-D08; catalogNumber: BC-ZSM-HYM-25461-D08; recordNumber: BC-ZSM-HYM-25461-D08; recordedBy: P. Benander; individualID: BC-ZSM-HYM-25461-D08; individualCount: 1; lifeStage: adult; **Taxon:** scientificName: Tetrastichus argei; phylum: Arthropoda; class: Insecta; order: Hymenoptera; family: Eulophidae; genus: Tetrastichinae; **Location:** country: Sweden; decimalLatitude: 56.294; decimalLongitude: 12.495; **Record Level:** type: PhysicalObject; language: en; basisOfRecord: PreservedSpecimen

#### Description

FEMALE holotype (Fig. 15). Body length 1.8 mm (paratypes 1.5–1.9 mm). *Head*. Width/length in dorsal view 2.2, width/length in frontal view 1.3, POL/OOL 1.8, widths head/mesosoma 1.1, mouth width/malar space 0.9, malar space/eye height 0.8. *Antenna*. Scape length/eye height 1.0, pedicel+flagellum length/mesosoma width 1.3, length/width F1, F2, F3 2.0, 2.0, 1.6, clava length/width 3.4, lengths pedicel/F1 0.7, lengths F1/F2 1.0, F1/F3 1.1, lengths F1, F2, F3/clava 0.5, 0.5, 0.5, widths F1/pedicel (dorsal view) 1.1, lengths antennal spicule/C3 0.2. *Mesosoma*. Length/width 1.5, mesoscutal mid-lobe length/width 1.0 (width measured in anterior part), mid-lobe with median groove in posterior ¾, with four adnotaular setae on each side, lengths mesoscutum/mesoscutellum (measured medially) 1.3, mesoscutellum length/width 0.9, length/width of enclosed space between submedian grooves 2.7, distance between SMG/distance between SMG and SLG 1.4, lengths dorsellum/propodeum (measured medially) 0.5, propodeum with strong reticulation, propodeal callus with six setae. *Fore wing*. Costal cell length/width 12.8, lengths costal cell/marginal vein 1.0, lengths marginal/stigmal veins 2.9. *Gaster*. Subcircular to ovate, length/width 1.5, lengths gaster/mesosoma 1.2, Gt_7_ length/width 0.3, length of longest cercal seta/next longest seta 1.4, longest cercal seta straight, ovipositor sheaths not reaching apex of Gt_7_.

Colour. Body metallic blue-green, entire antenna dark brown, tegulae black with metallic tinge, wings hyaline with veins yellowish-brown, coxae and femora concolorous with body, trochanters dark brown, tibiae yellowish-brown, fore tarsus brown, mid and hind tarsi yellowish-brown with T3–4 brown.

MALE. Body length 1.7–1.8 mm. *Head*. Width/length in dorsal view 2.3, width/length in frontal view 1.2, eye height/malar space 1.2, mouth width/malar space 1.1, widths head/mesosoma 1.0. *Antenna*. F1–F4 without basal whorls of setae, scape length/eye height 1.1, scape length/width 2.4, ventral plaque placed in central part of scape, lengths ventral plaque/scape 0.7, pedicel+flagellum length/mesosoma width 1.5, length/width F1, F2, F3, F4 1.7, 2.0, 2.0, 2.2, clava length/width 4.4, lengths pedicel/F1 1.0, lengths F1/F2 0.8, F1/F3 0.8, F1/F4 0.7, lengths F1, F2, F3, F4/clava 0.3, 0.5, 0.5, 0.5.

Colour. As in female.

#### Diagnosis

Tibiae yellowish-brown; female with malar space 0.8× eye height, scape 3.5× as long as wide, antennal clava 3.4× as long as wide; mesoscutellum with ratio distance between submedian grooves to distance between submedian and sublateral grooves 1.4.

#### Distribution

Sweden.

#### Notes

Holotype deposited in MZLU, paratypes in MZLU, NHM, ZSM.

#### Host

Gregarious endoparasitoid on *Arge ustulata* (L.) (Hymenoptera: Argidae), 14♀ and 3♂ have been reared from the same host specimen. The label information does not specify which stage of the sawfly that was parasitised. Another *Tetrastichus* species, *T. hylotomarum*, has been recorded from larvae and pupae of *Arge* spp. (Graham 1991), but not specifically *A. ustulata*.

#### Additional paratypes (not barcoded)

1♀ 1♂ ”SWEDEN, Skåne, Kullaberg, 28.ii.1966, P. Benander, ex Arge ustulata”.

### Tetrastichus asilis sp. n.

30BB7768-616C-5D2C-A2ED-83A37C405831

urn:lsid:zoobank.org:act:14CCE9D7-3579-422B-A7A0-3951D456CA08

#### Materials

- **Type status:** Holotype. **Occurrence:** occurrenceDetails: http://www.boldsystems.org/index.php/API_Public/specimen?ids=BC-ZSM-HYM-25459-G12; catalogNumber: BC-ZSM-HYM-25459-G12; recordNumber: BC-ZSM-HYM-25459-G12; recordedBy: D. Doczkal; individualID: BC-ZSM-HYM-25459-G12; individualCount: 1; sex: female; lifeStage: adult; **Taxon:** scientificName: Tetrastichus asilis; phylum: Arthropoda; class: Insecta; order: Hymenoptera; family: Eulophidae; genus: Tetrastichinae; taxonRemarks: Holotype deposited in ZSM; **Location:** country: Germany; decimalLatitude: 49.985; decimalLongitude: 9.769; **Record Level:** type: PhysicalObject; language: en; institutionCode: ZSM; basisOfRecord: PreservedSpecimen

#### Description

FEMALE holotype (Fig. 16). Body length 1.4 mm. *Head.* Width/length in dorsal view 2.1, width/length in frontal view 1.1, POL/OOL 2.4, widths head/mesosoma 1.2, mouth width/malar space 1.0, malar space/eye height 0.8. *Antenna.* Scape length/eye height 1.2, pedicel+flagellum length/mesosoma width 1.5, length/width F1, F2, F3 1.6, 1.9, 1.7, clava length/width 4.0, lengths pedicel/F1 0.9, lengths F1/F2 0.9, F1/F3 0.9, lengths F1, F2, F3/clava 0.3, 0.4, 0.4, widths F1/pedicel (dorsal view) 1.0, lengths antennal spicule/C3 0.2. *Mesosoma.* Length/width 1.7, mesoscutal mid-lobe length/width 1.1 (width measured in anterior part), mid-lobe with a weak median groove in posterior ⅓, with three adnotaular setae on each side, lengths of mesoscutum/scutellum (measured medially) 1.4, mesoscutellum length/width 1.0, length/width of enclosed space between submedian grooves 2.5, distance between SMG/distance between SMG and SLG 2.0, lengths dorsellum/propodeum 0.6, propodeum with very weak reticulation partly smooth, propodeal callus with two setae. *Fore wing.* Costal cell length/width 13.5, lengths costal cell/marginal vein 1.1, lengths marginal/stigmal veins 2.4. *Gaster*. Short ovate, length/width 1.5, lengths gaster/mesosoma 1.1, Gt_7_ length/width 0.3, length of longest cercal seta/next longest seta 1.5, longest cercal seta evenly curved, ovipositor sheaths not reaching apex of Gt_7_.

Colour. Body metallic bluish-green, scape yellowish-brown, pedicel and flagellum pale brown, tegulae dark brown with metallic tinges, wing venation yellowish-brown, coxae and femora concolorous with body, trochanters dark brown, tibiae and tarsi yellowish-brown, T4 pale brown.

MALE. Unknown.

#### Diagnosis

Tibiae yellowish-brown; female with malar space 0.9× eye height, POL/OOL 2.4, F1 1.6×, F2 1.9×, F3 1.7× as long as wide.

#### Distribution

Germany.

#### Biology

##### Host

Unknown.

#### Notes

Holotype deposited in ZSM.

### Tetrastichus brevicalcar

Graham, 1991

201BAAAB-7BC6-5BCF-8B57-48F20932A80B

Tetrastichus brevicalcar Graham 1991:243. Holotype ♀ in NHM, examined (Fig. 17).

#### Description

See Graham (1991).

#### Diagnosis

Female flagellum long and slender, for example, F1 2.4–2.7×, F3 2.2–2.5× and clava 4.5–5.2× as long as wide.

#### Distribution

United Kingdom (Graham 1991), Sweden (Hedqvist 2003) and Czech Republic (**new record**).

#### Host

Unknown.

#### Additional material examined

24♀: Czech Republic 1♀ (NHM), Sweden 20♀ (MZLU, NHM, SMTP), United Kingdom 3♀ (NHM).

### Tetrastichus calmius sp. n.

C703D6B4-3C71-5F19-BDA9-C905655CE552

urn:lsid:zoobank.org:act:A1C32083-BE37-4AC2-9C54-07E8A62C07FB

#### Materials

- **Type status:** Holotype. **Occurrence:** occurrenceDetails: http://www.boldsystems.org/index.php/API_Public/specimen?ids=BC-ZSM-HYM-29751-B09; catalogNumber: BC-ZSM-HYM-29751-B09; recordNumber: BC-ZSM-HYM-29751-B09; recordedBy: C. Hansson; individualID: BC-ZSM-HYM-29751-B09; individualCount: 1; sex: female; lifeStage: adult; **Taxon:** scientificName: Tetrastichus calmius; phylum: Arthropoda; class: Insecta; order: Hymenoptera; family: Eulophidae; genus: Tetrastichinae; taxonRemarks: Holotype deposited in MZLU; **Location:** country: Sweden; decimalLatitude: 55.6861; decimalLongitude: 13.4611; **Record Level:** type: PhysicalObject; language: en; institutionCode: MZLU; basisOfRecord: PreservedSpecimen

#### Description

FEMALE holotype (Fig. 18). Body length 1.7 mm. *Head.* Width/length in dorsal view 2.0, width/length in frontal view 1.2, POL/OOL 1.9, widths head/mesosoma 1.1, mouth width/malar space 1.0, malar space/eye height 0.8. *Antenna.* Scape length/eye height 1.0, pedicel+flagellum length/mesosoma width 1.4, length/width F1, F2, F3 2.0, 2.1, 2.1, clava length/width 3.8, lengths pedicel/F1 0.8, lengths F1/F2 0.9, F1/F3 0.9, lengths F1, F2, F3/clava 0.4, 0.5, 0.5, widths F1/pedicel (dorsal view) 1.0, lengths antennal spicule/C3 0.2. *Mesosoma.* Length/width 1.5, mesoscutal mid-lobe length/width 1.0 (width measured in anterior part), mid-lobe with a weak median groove, with seven adnotaular setae on each side, lengths mesoscutum/mesoscutellum (measured medially) 1.2, lengths dorsellum/propodeum (measured medially) 0.7, mesoscutellum length/width 0.9, length/width of enclosed space between submedian grooves 2.7, distance between SMG/distance between SMG and SLG 1.4, propodeum with strong reticulation, propodeal callus with four setae. *Fore wing.* Costal cell length/width 10.7, lengths costal cell/marginal vein 1.0, lengths marginal/stigmal veins 2.8. *Gaster.* Ovate, length/width 1.6, lengths gaster/mesosoma 1.1, Gt_7_ length/width 0.6, lengths longest cercal seta/next longest seta 1.4, longest cercal seta straight, ovipositor sheaths not reaching apex of Gt_7_.

Colour. Body golden-green, scape yellowish-brown with dorsal edge dark brown, pedicel and flagellum dark brown, tegulae black with metallic tinge, wings hyaline with venation yellowish-white, coxae and femora concolorous with body, trochanters black, tibiae yellowish-brown, fore tarsus brown, mid and hind tarsi with T1–3 yellowish-brown, T4 brown.

MALE. Unknown.

#### Diagnosis

Antennal clava 3.8×, F1 1.0× and F2 2.1× as long as wide, F1 1.3× as long as pedicel; ratio POL/OOL = 1.9; length/width of enclosed space between submedian grooves 2.7.

#### Distribution

Sweden.

#### Notes

Holotype deposited in MZLU.

#### Host

Unknown.

### Tetrastichus coelarchus

Graham, 1991

6B444BFF-23F4-5D29-8AFE-6ABB9676C79F

Tetrastichus coelarchus Graham 1991:240. Holotype ♀ in NHM, examined (Fig. 19).

#### Description

See Graham (1991).

#### Diagnosis

Antennal clava 3.4–3.6× as long as wide; malar space 0.8–0.9× height of eye; mesoscutellum with enclosed space between submedian grooves 2.7× as long as wide.

#### Distribution

(Former) Czechoslovakia, Ireland, Sweden, United Kingdom and (former) Yugoslavia (Graham 1991).

#### Notes

Holotype deposited in MZLU, paratypes in MZLU.

#### Host

Unknown.

#### Additional material examined

9♀, Sweden (NHM).

### Tetrastichus coeruleus

(Nees, 1834)

47CA95B7-4790-580E-9109-89E0FE1D442A

Eulophus coeruleus Nees von Esenbeck 1834:174. Lectotype ♀ in OUMNH, designated by Graham 1961:39–40, examined (Fig. 20). Transferred to *Aprostocetus* by Graham 1961:39–40 and to *Tetrastichus* by Graham 1991:238.
Tetrastichus coeruleus *Tetrastichus asparagiCrawford 1909*Graham 1961

#### Description

See Graham (1991).

#### Diagnosis

Mouth opening very wide, 1.8–2.0 × malar space; body bright metallic blue to bluish-purple; ovipositor does not reach apex of Gt_7_ ; male funiculars with short whorled setae, 0.5–0.7 × as long as funicular attached to.

#### Distribution

France, Germany, Hungary, Italy, The Netherlands, United Kingdom (Graham 1991); Norway and Sweden (**new records**).

#### Ecology

##### Host

*Crioceris asparagi* (L.) (Coleoptera: Chrysomelidae), emerges from host cocoons, but oviposits in host eggs or larvae (Van Alphen 1980).

##### Material examined

Type material: lectotype ♀ of *E. coeruleus* (OUMNH). Additional material (72♀ 18♂): China 1♀ (UCRC), France 2♀ (GD, NHM), Norway 3♀ 1♂ (NHM), Sweden 63♀ 17♂ (MZLU, NHM, ZSM), United Kingdom 3♀ (NHM).

### Tetrastichus cosidis sp. n.

3B6E5DFE-87C4-524A-9D56-EC6AF3AD42B9

urn:lsid:zoobank.org:act:D7F3B936-4C8D-4550-9C1E-EDDF367EE3DC

#### Materials

- **Type status:** Holotype. **Occurrence:** occurrenceDetails: http://www.boldsystems.org/index.php/API_Public/specimen?ids=BC-ZSM-HYM-20721-B06; catalogNumber: BC-ZSM-HYM-20721-B06; recordNumber: BC-ZSM-HYM-20721-B06; recordedBy: C. Hansson; individualID: BC-ZSM-HYM-20721-B06; individualCount: 1; lifeStage: adult; **Taxon:** scientificName: Tetrastichus cosidis; phylum: Arthropoda; class: Insecta; order: Hymenoptera; family: Eulophidae; genus: Tetrastichinae; taxonRemarks: Holotype deposited in MZLU; **Location:** country: Sweden; decimalLatitude: 56.617; decimalLongitude: 16.567; **Record Level:** type: PhysicalObject; language: en; institutionCode: MZLU; basisOfRecord: PreservedSpecimen

#### Description

FEMALE holotype (Fig. 21). Body length 2.1 mm. *Head.* Width/length in dorsal view 2.1, width/length in frontal view 1.3, POL/OOL 1.8, widths head/mesosoma 1.1, mouth width/malar space 0.9, malar space/eye height 0.9, scape length/eye height 1.1, pedicel+flagellum length/mesosoma width 1.3, length/width F1, F2, F3 2.0, 1.9, 2.0, clava length/width 4.2, lengths pedicel/F1 0.6, lengths F1/F2 1.1, F1/F3 1.0, lengths F1, F2, F3/clava 0.5, 0.5, 0.5, widths F1/pedicel (dorsal view) 1.3, lengths antennal spicule/C3 0.3. *Mesosoma.* Length/width 1.5, mesoscutal mid-lobe length/width 1.0 (width measured in anterior part), mid-lobe with a median groove that is weak in anterior part, with four adnotaular setae on each side, lengths mesoscutum/mesoscutellum (measured medially) 1.3, lengths dorsellum/propodeum 0.6, mesoscutellum length/width 1.1, length/width of enclosed space between submedian grooves 2.6, distance between SMG/distance between SMG and SLG 1.8, propodeum with weak reticulation, propodeal callus with five setae. *Fore wing.* Costa cell length/width 10.0, lengths costal cell/marginal vein 1.0, lengths marginal/stigmal veins 2.6. *Gaster.* Ovate, length/width 1.5, lengths gaster/mesosoma 1.1, Gt_7_ length/width 0.6, cercal setae nm, ovipositor sheaths not reaching apex of Gt_7_.

Colour. Body golden-green, antenna dark brown, tegulae black with metallic tinges, wings hyaline, wing venation yellowish-white, coxae and femora concolorous with body, trochanters blackish, tibiae and tarsi yellowish-brown.

MALE. Unknown.

#### Diagnosis

Hind coxa with a strong, sharp and complete carina along posterior margin; malar space 0.9× eye height; antennal clava 3.9× as long as wide; mesoscutellum with ratio distance between SMG/distance between SMG and SLG 1.4, length/width of enclosed space between submedian grooves 2.6.

#### Distribution

Sweden.

#### Notes

Holotype deposited in MZLU.

#### Host

Unknown.

### Tetrastichus cumulus sp. n.

3392B568-89AA-5034-9647-70ACB747B221

urn:lsid:zoobank.org:act:AC5ACFA0-7AF2-403A-ABA5-31F37EF65969

#### Materials

- **Type status:** Holotype. **Occurrence:** occurrenceDetails: http://www.boldsystems.org/index.php/API_Public/specimen?ids=BC-ZSM-HYM-29750-C09; catalogNumber: BC-ZSM-HYM-29750-C09; recordNumber: BC-ZSM-HYM-29750-C09; recordedBy: C. Hansson; individualID: BC-ZSM-HYM-29750-C09; individualCount: 1; sex: female; lifeStage: adult; **Taxon:** scientificName: Tetrastichus cumulus; phylum: Arthropoda; class: Insecta; order: Hymenoptera; family: Eulophidae; genus: Tetrastichinae; taxonRemarks: Holotype deposited in MZLU; **Location:** country: Sweden; decimalLatitude: 55.6861; decimalLongitude: 13.4611; **Record Level:** type: PhysicalObject; language: en; institutionCode: MZLU; basisOfRecord: PreservedSpecimen
- **Type status:** Paratype. **Occurrence:** occurrenceDetails: http://www.boldsystems.org/index.php/API_Public/specimen?ids=BC-ZSM-HYM-29750-D02; catalogNumber: BC-ZSM-HYM-29750-D02; recordNumber: BC-ZSM-HYM-29750-D02; recordedBy: C. Hansson; individualID: BC-ZSM-HYM-29750-D02; individualCount: 1; sex: male; lifeStage: adult; **Taxon:** scientificName: Tetrastichus cumulus; phylum: Arthropoda; class: Insecta; order: Hymenoptera; family: Eulophidae; genus: Tetrastichinae; **Location:** country: Sweden; decimalLatitude: 55.6861; decimalLongitude: 13.4611; **Record Level:** type: PhysicalObject; language: en; basisOfRecord: PreservedSpecimen
- **Type status:** Paratype. **Occurrence:** occurrenceDetails: http://www.boldsystems.org/index.php/API_Public/specimen?ids=BC-ZSM-HYM-29750-C05; catalogNumber: BC-ZSM-HYM-29750-C05; recordNumber: BC-ZSM-HYM-29750-C05; recordedBy: C. Hansson; individualID: BC-ZSM-HYM-29750-C05; individualCount: 1; sex: female; lifeStage: adult; **Taxon:** scientificName: Tetrastichus cumulus; phylum: Arthropoda; class: Insecta; order: Hymenoptera; family: Eulophidae; genus: Tetrastichinae; **Location:** country: Sweden; decimalLatitude: 55.6861; decimalLongitude: 13.4611; **Record Level:** type: PhysicalObject; language: en; basisOfRecord: PreservedSpecimen
- **Type status:** Paratype. **Occurrence:** occurrenceDetails: http://www.boldsystems.org/index.php/API_Public/specimen?ids=BC-ZSM-HYM-29814-D08; catalogNumber: BC-ZSM-HYM-29814-D08; recordNumber: BC-ZSM-HYM-29814-D08; recordedBy: C. Hansson; individualID: BC-ZSM-HYM-29814-D08; individualCount: 1; sex: female; lifeStage: adult; **Taxon:** scientificName: Tetrastichus cumulus; phylum: Arthropoda; class: Insecta; order: Hymenoptera; family: Eulophidae; genus: Tetrastichinae; **Location:** country: Sweden; decimalLatitude: 55.7078; decimalLongitude: 13.475; **Record Level:** type: PhysicalObject; language: en; basisOfRecord: PreservedSpecimen
- **Type status:** Paratype. **Occurrence:** occurrenceDetails: http://www.boldsystems.org/index.php/API_Public/specimen?ids=BC-ZSM-HYM-27493-F01; catalogNumber: BC-ZSM-HYM-27493-F01; recordNumber: BC-ZSM-HYM-27493-F01; recordedBy: SMTP; individualID: BC-ZSM-HYM-27493-F01; individualCount: 1; sex: female; lifeStage: adult; **Taxon:** scientificName: Tetrastichus cumulus; phylum: Arthropoda; class: Insecta; order: Hymenoptera; family: Eulophidae; genus: Tetrastichinae; **Location:** country: Sweden; decimalLatitude: 60.276; decimalLongitude: 17.191; **Record Level:** type: PhysicalObject; language: en; basisOfRecord: PreservedSpecimen
- **Type status:** Paratype. **Occurrence:** occurrenceDetails: http://www.boldsystems.org/index.php/API_Public/specimen?ids=BC-ZSM-HYM-29751-C07; catalogNumber: BC-ZSM-HYM-29751-C07; recordNumber: BC-ZSM-HYM-29751-C07; recordedBy: C. Hansson; individualID: BC-ZSM-HYM-29751-C07; individualCount: 1; sex: female; lifeStage: adult; **Taxon:** scientificName: Tetrastichus cumulus; phylum: Arthropoda; class: Insecta; order: Hymenoptera; family: Eulophidae; genus: Tetrastichinae; **Location:** country: Sweden; decimalLatitude: 55.6967; decimalLongitude: 13.47; **Record Level:** type: PhysicalObject; language: en; basisOfRecord: PreservedSpecimen
- **Type status:** Paratype. **Occurrence:** occurrenceDetails: http://www.boldsystems.org/index.php/API_Public/specimen?ids=BC-ZSM-HYM-21587-G07; catalogNumber: BC-ZSM-HYM-21587-G07; recordNumber: BC-ZSM-HYM-21587-G07; recordedBy: C.Hansson; individualID: BC-ZSM-HYM-21587-G07; individualCount: 1; sex: female; lifeStage: adult; **Taxon:** scientificName: Tetrastichus cumulus; phylum: Arthropoda; class: Insecta; order: Hymenoptera; family: Eulophidae; genus: Tetrastichinae; **Location:** country: Sweden; decimalLatitude: 55.834; decimalLongitude: 13.528; **Record Level:** type: PhysicalObject; language: en; basisOfRecord: PreservedSpecimen
- **Type status:** Paratype. **Occurrence:** occurrenceDetails: http://www.boldsystems.org/index.php/API_Public/specimen?ids=BC-ZSM-HYM-22523-D05; catalogNumber: BC-ZSM-HYM-22523-D05; recordNumber: BC-ZSM-HYM-22523-D05; recordedBy: Swedish Malaise Trap Project; individualID: BC-ZSM-HYM-22523-D05; individualCount: 1; sex: male; lifeStage: adult; **Taxon:** scientificName: Tetrastichus cumulus; phylum: Arthropoda; class: Insecta; order: Hymenoptera; family: Eulophidae; genus: Tetrastichinae; **Location:** country: Sweden; decimalLatitude: 58.185; decimalLongitude: 14.379; **Record Level:** type: PhysicalObject; language: en; basisOfRecord: PreservedSpecimen
- **Type status:** Paratype. **Occurrence:** occurrenceDetails: http://www.boldsystems.org/index.php/API_Public/specimen?ids=BC-ZSM-HYM-13565-A02; catalogNumber: BC-ZSM-HYM-13565-A02; recordNumber: BC-ZSM-HYM-13565-A02; recordedBy: SMTP project; individualID: BC-ZSM-HYM-13565-A02; individualCount: 1; sex: female; lifeStage: adult; **Taxon:** scientificName: Tetrastichus cumulus; phylum: Arthropoda; class: Insecta; order: Hymenoptera; family: Eulophidae; genus: Tetrastichinae; **Location:** country: Sweden; decimalLatitude: 56.933; decimalLongitude: 18.267; **Record Level:** type: PhysicalObject; language: en; basisOfRecord: PreservedSpecimen
- **Type status:** Paratype. **Occurrence:** occurrenceDetails: http://www.boldsystems.org/index.php/API_Public/specimen?ids=BC-ZSM-HYM-26563-E11; catalogNumber: BC-ZSM-HYM-26563-E11; recordNumber: BC-ZSM-HYM-26563-E11; recordedBy: C. Hansson; individualID: BC-ZSM-HYM-26563-E11; individualCount: 1; sex: female; lifeStage: adult; **Taxon:** scientificName: Tetrastichus cumulus; phylum: Arthropoda; class: Insecta; order: Hymenoptera; family: Eulophidae; genus: Tetrastichinae; **Location:** country: Sweden; decimalLatitude: 55.6619; decimalLongitude: 13.5472; **Record Level:** type: PhysicalObject; language: en; basisOfRecord: PreservedSpecimen
- **Type status:** Paratype. **Occurrence:** occurrenceDetails: http://www.boldsystems.org/index.php/API_Public/specimen?ids=BC-ZSM-HYM-22524-F07; catalogNumber: BC-ZSM-HYM-22524-F07; recordNumber: BC-ZSM-HYM-22524-F07; recordedBy: E. Shevtsova; individualID: BC-ZSM-HYM-22524-F07; individualCount: 1; sex: female; lifeStage: adult; **Taxon:** scientificName: Tetrastichus cumulus; phylum: Arthropoda; class: Insecta; order: Hymenoptera; family: Eulophidae; genus: Tetrastichinae; **Location:** country: Russia; decimalLatitude: 59.567; decimalLongitude: 30.133; **Record Level:** type: PhysicalObject; language: en; basisOfRecord: PreservedSpecimen
- **Type status:** Paratype. **Occurrence:** occurrenceDetails: http://www.boldsystems.org/index.php/API_Public/specimen?ids=BC-ZSM-HYM-20721-F12; catalogNumber: BC-ZSM-HYM-20721-F12; recordNumber: BC-ZSM-HYM-20721-F12; recordedBy: C. Hansson; individualID: BC-ZSM-HYM-20721-F12; individualCount: 1; sex: female; lifeStage: adult; **Taxon:** scientificName: Tetrastichus cumulus; phylum: Arthropoda; class: Insecta; order: Hymenoptera; family: Eulophidae; genus: Tetrastichinae; **Location:** country: Sweden; decimalLatitude: 55.7; decimalLongitude: 13.5; **Record Level:** type: PhysicalObject; language: en; basisOfRecord: PreservedSpecimen
- **Type status:** Paratype. **Occurrence:** occurrenceDetails: http://www.boldsystems.org/index.php/API_Public/specimen?ids=BC-ZSM-HYM-20721-F09; catalogNumber: BC-ZSM-HYM-20721-F09; recordNumber: BC-ZSM-HYM-20721-F09; recordedBy: C. Hansson; individualID: BC-ZSM-HYM-20721-F09; individualCount: 1; sex: male; lifeStage: adult; **Taxon:** scientificName: Tetrastichus cumulus; phylum: Arthropoda; class: Insecta; order: Hymenoptera; family: Eulophidae; genus: Tetrastichinae; **Location:** country: Sweden; decimalLatitude: 55.7; decimalLongitude: 13.5; **Record Level:** type: PhysicalObject; language: en; basisOfRecord: PreservedSpecimen
- **Type status:** Paratype. **Occurrence:** occurrenceDetails: http://www.boldsystems.org/index.php/API_Public/specimen?ids=BC-ZSM-HYM-20721-G08; catalogNumber: BC-ZSM-HYM-20721-G08; recordNumber: BC-ZSM-HYM-20721-G08; recordedBy: C. Hansson; individualID: BC-ZSM-HYM-20721-G08; individualCount: 1; sex: female; lifeStage: adult; **Taxon:** scientificName: Tetrastichus cumulus; phylum: Arthropoda; class: Insecta; order: Hymenoptera; family: Eulophidae; genus: Tetrastichinae; **Location:** country: Sweden; decimalLatitude: 55.7; decimalLongitude: 13.483; **Record Level:** type: PhysicalObject; language: en; basisOfRecord: PreservedSpecimen
- **Type status:** Paratype. **Occurrence:** occurrenceDetails: http://www.boldsystems.org/index.php/API_Public/specimen?ids=BC-ZSM-HYM-20721-D05; catalogNumber: BC-ZSM-HYM-20721-D05; recordNumber: BC-ZSM-HYM-20721-D05; recordedBy: C. Hansson; individualID: BC-ZSM-HYM-20721-D05; individualCount: 1; sex: female; lifeStage: adult; **Taxon:** scientificName: Tetrastichus cumulus; phylum: Arthropoda; class: Insecta; order: Hymenoptera; family: Eulophidae; genus: Tetrastichinae; **Location:** country: Sweden; decimalLatitude: 55.667; decimalLongitude: 13.55; **Record Level:** type: PhysicalObject; language: en; basisOfRecord: PreservedSpecimen
- **Type status:** Paratype. **Occurrence:** occurrenceDetails: http://www.boldsystems.org/index.php/API_Public/specimen?ids=BC-ZSM-HYM-20721-B08; catalogNumber: BC-ZSM-HYM-20721-B08; recordNumber: BC-ZSM-HYM-20721-B08; recordedBy: C. Hansson; individualID: BC-ZSM-HYM-20721-B08; individualCount: 1; sex: female; lifeStage: adult; **Taxon:** scientificName: Tetrastichus cumulus; phylum: Arthropoda; class: Insecta; order: Hymenoptera; family: Eulophidae; genus: Tetrastichinae; **Location:** country: Sweden; decimalLatitude: 56.75; decimalLongitude: 16.65; **Record Level:** type: PhysicalObject; language: en; basisOfRecord: PreservedSpecimen
- **Type status:** Paratype. **Occurrence:** occurrenceDetails: http://www.boldsystems.org/index.php/API_Public/specimen?ids=BC-ZSM-HYM-20721-A03; catalogNumber: BC-ZSM-HYM-20721-A03; recordNumber: BC-ZSM-HYM-20721-A03; recordedBy: C. Hansson; individualID: BC-ZSM-HYM-20721-A03; individualCount: 1; sex: female; lifeStage: adult; **Taxon:** scientificName: Tetrastichus cumulus; phylum: Arthropoda; class: Insecta; order: Hymenoptera; family: Eulophidae; genus: Tetrastichinae; **Location:** country: Sweden; decimalLatitude: 55.583; decimalLongitude: 13.417; **Record Level:** type: PhysicalObject; language: en; basisOfRecord: PreservedSpecimen
- **Type status:** Paratype. **Occurrence:** occurrenceDetails: http://www.boldsystems.org/index.php/API_Public/specimen?ids=BC-ZSM-HYM-27493-F10; catalogNumber: BC-ZSM-HYM-27493-F10; recordNumber: BC-ZSM-HYM-27493-F10; recordedBy: SMTP; individualID: BC-ZSM-HYM-27493-F10; individualCount: 1; sex: female; lifeStage: adult; **Taxon:** scientificName: Tetrastichus cumulus; phylum: Arthropoda; class: Insecta; order: Hymenoptera; family: Eulophidae; genus: Tetrastichinae; **Location:** country: Sweden; decimalLatitude: 59.107; decimalLongitude: 18.138; **Record Level:** type: PhysicalObject; language: en; basisOfRecord: PreservedSpecimen
- **Type status:** Paratype. **Occurrence:** occurrenceDetails: http://www.boldsystems.org/index.php/API_Public/specimen?ids=BC-ZSM-HYM-27493-H11; catalogNumber: BC-ZSM-HYM-27493-H11; recordNumber: BC-ZSM-HYM-27493-H11; recordedBy: SMTP; individualID: BC-ZSM-HYM-27493-H11; individualCount: 1; sex: female; lifeStage: adult; **Taxon:** scientificName: Tetrastichus cumulus; phylum: Arthropoda; class: Insecta; order: Hymenoptera; family: Eulophidae; genus: Tetrastichinae; **Location:** country: Sweden; decimalLatitude: 57.004; decimalLongitude: 16.066; **Record Level:** type: PhysicalObject; language: en; basisOfRecord: PreservedSpecimen
- **Type status:** Paratype. **Occurrence:** occurrenceDetails: http://www.boldsystems.org/index.php/API_Public/specimen?ids=BC-ZSM-HYM-13565-A11; catalogNumber: BC-ZSM-HYM-13565-A11; recordNumber: BC-ZSM-HYM-13565-A11; recordedBy: SMTP; individualID: BC-ZSM-HYM-13565-A11; individualCount: 1; sex: female; lifeStage: adult; **Taxon:** scientificName: Tetrastichus cumulus; phylum: Arthropoda; class: Insecta; order: Hymenoptera; family: Eulophidae; genus: Tetrastichinae; **Location:** country: Sweden; decimalLatitude: 64.183; decimalLongitude: 19.60; **Record Level:** type: PhysicalObject; language: en; basisOfRecord: PreservedSpecimen

#### Description

FEMALE holotype (Fig. 22). Body length 2.5 mm (paratypes 1.7–2.7 mm). *Head*. Width/length in dorsal view 2.2, width/length in frontal view 1.3, POL/OOL 2.0, widths head/mesosoma 1.0, mouth width/malar space 1.0, malar space/eye height 0.9. *Antenna*. Scape length/eye height 1.0, pedicel+flagellum length/mesosoma width 1.2, length/width F1, F2, F3 2.3, 2.0, 1.8, clava length/width 3.8, lengths pedicel/F1 0.6, lengths F1/F2 1.0, F1/F3 1.1, lengths F1, F2, F3/clava 0.5, 0.5, 0.5, widths F1/pedicel (dorsal view) 1.2, lengths antennal spicule/C3 0.3. *Mesosoma*. Length/width 1.5, mesoscutal mid-lobe length/width 0.9 (width measured in anterior part), mid-lobe with median groove in posterior 4/5, with six adnotaular setae on each side, lengths mesoscutum/mesoscutellum (measured medially) 1.3, mesoscutellum length/width 0.9, length/width of enclosed space between submedian grooves 3.3, distance between SMG/distance between SMG and SLG 1.2, lengths dorsellum/propodeum (measured medially) 0.7, propodeum with strong reticulation, propodeal callus with four setae. *Fore wing*. Costal cell length/width 8.8, lengths costal cell/marginal vein 1.0, lengths marginal/stigmal veins 3.1. *Gaster*. Subcircular to ovate, length/width 1.4, lengths gaster/mesosoma 1.1, Gt_7_ length/width 0.4, length of longest cercal seta/next longest seta 1.3, longest cercal seta straight, ovipositor sheaths not reaching apex of Gt_7_.

Colour. Body metallic blue-green, scape dark brown with base dark yellowish-brown, pedicel and flagellum dark brown, tegulae black with metallic tinge, wings hyaline with veins yellowish-brown, coxae and femora concolorous with body, trochanters black, tibiae yellowish-brown, fore tarsus dark brown, mid and hind tarsi yellowish-brown with T4 brown.

Variation. Colour of scape varies from completely dark brown to yellowish-brown with apicodorsal ⅓ dark brown, but most specimens with colour as holotype. Paratypes with body metallic blue or blue-green.

MALE (Fig. 22). Body length 1.3–1.5 mm. *Head*. Width/length in dorsal view 2.3, width/length in frontal view 1.3, eye height/malar space 1.3, mouth width/malar space 1.1, widths head/mesosoma 1.1. *Antenna*. F1–F4 without basal whorls of setae, scape length/eye height 1.0, scape length/width 2.2, ventral plaque placed in central part of scape, lengths ventral plaque/scape 0.7, pedicel+flagellum length/mesosoma width 1.8, length/width F1, F2, F3, F4 1.9, 2.1, 2.1, 2.1, clava length/width 5.0, lengths pedicel/F1 0.8, lengths F1/F2 0.9, F1/F3 0.9, F1/F4 0.9, lengths F1, F2, F3, F4/clava 0.4, 0.4, 0.4, 0.4.

Colour. As in female, but scape completely dark brown. Body metallic blue-green or purple.

#### Diagnosis

Mid and hind tibiae yellowish-brown; mesoscutellum with distance between submedian grooves short, distance between SMG/distance between SMG and SLG 1.2; female with malar space 0.9× height of eye, antenna with F2 2.0×, F3 1.8× and clava 3.8× as long as wide; male antenna with scape 2.2× and clava 5.0× as long as wide and scape length 1.0× height of eye.

#### Distribution

Russia and Sweden.

#### Notes

Holotype deposited in MZLU, paratypes in MZLU, NHM, SMTP and ZSM.

#### Host

Unknown.

#### Additional paratype (not barcoded)

1♀ ”SWEDEN, Skåne, Dalby, Ö. Mölla, 21-27.viii.1998, yellow pan trap, R. Danielsson (MZLU)

### Tetrastichus cyprus sp. n.

6EC118F0-A2B0-54F6-A105-CFE7E7932CDD

urn:lsid:zoobank.org:act:8F2ECA2A-663C-47D9-B10D-A6F6212D6F49

#### Description

FEMALE holotype (Fig. 23). Body length 2.0 mm (paratypes 1.7–2.3 mm). *Head*. Width/length in dorsal view 2.4, width/length in frontal view 2.0, POL/OOL 1.5, widths head/mesosoma 1.2, mouth width/malar space 1.9, malar space/eye height 0.7. *Antenna*. Scape length/eye height 1.0, pedicel+flagellum length/mesosoma width 1.1, length/width F1, F2, F3 1.5, 1.4, 1.3, clava length/width 2.7, lengths pedicel/F1 0.8, lengths F1/F2 1.1, F1/F3 1.2, lengths F1, F2, F3/clava 0.5, 0.5, 0.4, widths F1/pedicel (dorsal view) 1.5, lengths antennal spicule/C3 0.3. *Mesosoma*. Length/width 1.4, mesoscutal mid-lobe length/width (width measured in anterior part) 0.9, mid-lobe with median groove in posterior ⅓, with four adnotaular setae on each side, lengths mesoscutum/mesoscutellum (measured medially) 1.3, mesoscutellum length/width 0.9, length/width of enclosed space between submedian grooves 2.7, distance between SMG/distance between SMG and SLG 1.4, lengths dorsellum/propodeum 0.5, propodeum with weak reticulation, propodeal callus with three setae. *Fore wing*. Costal cell length/width 10.6, lengths costal cell/marginal vein 1.1, lengths marginal/stigmal veins 3.0. *Gaster*. Subcircular to ovate, length/width 1.3, lengths gaster/mesosoma 1.2, Gt_7_ length/width 0.7, length of longest cercal seta/next longest seta 0.6, longest cercal seta straight, ovipositor sheaths not reaching apex of Gt_7_.

Colour. Body with metallic greenish-blue, entire antenna dark brown, tegulae black, wings hyaline with veins yellowish-brown to brown, coxae concolorous with body, trochanters dark brown, fore and mid femora dark brown, hind femur concolorous with body, tibiae and tarsi yellowish-brown.

MALE. Unknown.

#### Diagnosis

Similar to *T. halidayi* with the wide mouth and large and oddly-shaped mandibles (as in Figs. 2a and 65d). Differs from *T. halidayi* in having female gaster short ovate, 1.3× as long as wide, ovipositor sheaths short and not visible in dorsal view, cerci on gaster placed ventrally and not visible in dorsal view, setae on vertex shorter, 0.45× OD and body strongly metallic.

#### Distribution

Cyprus.

#### Notes

The specimens in the type series are not in pristine condition, all specimens being more or less broken and dirty. The holotype is missing right wing pair and right pedicel+flagellum is broken off and glued separately to the card.

#### Host

Reared from an unidentified Coleoptera and from an unidentified leaf miner on broad bean (*Vicia faba* L.).

#### Material examined

Holotype ♀ ”CYPRUS, Phlasson, 30.XII.1967, G.P. Georghiou. Parasite of Coleoptera #521” (UCRC). Paratypes (3♀): 2♀ with same label data as holotype (UCRC), 1♀ from same locality and date as holotype, but ex leaf miner on broad bean (UCRC).

### Tetrastichus erinus sp. n.

58DA369C-4B42-5CB9-ABDC-7D3EC2E71470

urn:lsid:zoobank.org:act:65D13412-7863-4178-B6CB-2C3A6041F429

#### Materials

- **Type status:** Holotype. **Occurrence:** occurrenceDetails: http://www.boldsystems.org/index.php/API_Public/specimen?ids=BC-ZSM-HYM-20721-F10; catalogNumber: BC-ZSM-HYM-20721-F10; recordNumber: BC-ZSM-HYM-20721-F10; recordedBy: C. Hansson; individualID: BC-ZSM-HYM-20721-F10; individualCount: 1; sex: female; lifeStage: adult; **Taxon:** scientificName: Tetrastichus erinus; phylum: Arthropoda; class: Insecta; order: Hymenoptera; family: Eulophidae; genus: Tetrastichinae; taxonRemarks: Holotype deposited in MZLU; **Location:** country: Sweden; decimalLatitude: 55.7; decimalLongitude: 13.5; **Record Level:** type: PhysicalObject; language: en; institutionCode: MZLU; basisOfRecord: PreservedSpecimen
- **Type status:** Paratype. **Occurrence:** occurrenceDetails: http://www.boldsystems.org/index.php/API_Public/specimen?ids=BC-ZSM-HYM-27493-H04; catalogNumber: BC-ZSM-HYM-27493-H04; recordNumber: BC-ZSM-HYM-27493-H04; recordedBy: SMTP; individualID: BC-ZSM-HYM-27493-H04; individualCount: 1; sex: female; lifeStage: adult; **Taxon:** scientificName: Tetrastichus erinus; phylum: Arthropoda; class: Insecta; order: Hymenoptera; family: Eulophidae; genus: Tetrastichinae; **Location:** country: Sweden; decimalLatitude: 67.031; decimalLongitude: 20.232; **Record Level:** type: PhysicalObject; language: en; institutionCode: SMTP; basisOfRecord: PreservedSpecimen

#### Description

FEMALE holotype (Fig. 24). Body length 1.9 mm (paratype 2.1 mm). *Head*. Width/length in dorsal view 2.1, width/length in frontal view 1.3, POL/OOL 2.0, widths head/mesosoma 1.1, mouth width/malar space 0.9, malar space/eye height 0.8. *Antenna*. Scape length/eye height 1.1, pedicel+flagellum length/mesosoma width 1.3, length/width F1, F2, F3 2.0, 2.0, 2.0, clava length/width 4.0, lengths pedicel/F1 0.7, lengths F1/F2 1.0, F1/F3 1.0, lengths F1, F2, F3/clava 0.5, 0.5, 0.5, widths F1/pedicel (dorsal view) 1.3, lengths antennal spicule/C3 0.2. *Mesosoma*. Length/width 1.4, mesoscutal mid-lobe length/width 0.9 (width measured in anterior part), mid-lobe with median groove in posterior ⅔, with three adnotaular setae on each side, lengths mesoscutum/mesoscutellum (measured medially) 1.2, mesoscutellum length/width 0.9, length/width of enclosed space between submedian grooves 2.2, distance between SMG/distance between SMG and SLG 1.9, lengths dorsellum/propodeum (measured medially) 0.6, propodeum with strong reticulation, propodeal callus with six setae. *Fore wing*. Costal cell length/width 12.8, lengths costal cell/marginal vein 1.0, lengths marginal/stigmal veins 3.1. *Gaster*. Subcircular to ovate, length/width 1.2, lengths gaster/mesosoma 1.1, Gt_7_ length/width 0.3, length of longest cercal seta/next longest seta 1.4, longest cercal seta straight, ovipositor sheaths not reaching apex of Gt_7_.

Colour. Body with golden-green, scape yellowish-brown with apical ⅓ brown, pedicel and flagellum dark brown, tegulae black with metallic tinge, wings hyaline with veins brown to fuscous, coxae and femora concolorous with body, trochanters dark brown, tibiae yellowish-brown, fore tarsus brown, mid and hind tarsi yellowish-white with T4 brownish.

Variation. Body blue-green in paratype.

MALE. Unknown.

#### Diagnosis

Mesoscutellum with distance between submedian grooves long, distances between submedian grooves/submedian and sublateral grooves 1.9, ratio length/width of enclosed space between submedian grooves 2.2; tibiae yellowish-brown; female with malar space 0.8× eye height; antennal clava 4× as long as wide; POL/OOL 2.0.

#### Distribution

Sweden.

#### Notes

Holotype deposited in MZLU, paratypes in SMTP.

#### Host

Unknown.

### Tetrastichus evexus sp. n.

B9BB81E8-F328-55D8-8379-DE6E1E2F411A

urn:lsid:zoobank.org:act:543B3F20-DFA1-44B0-B58A-DD88B270E613

#### Materials

- **Type status:** Holotype. **Occurrence:** occurrenceDetails: http://www.boldsystems.org/index.php/API_Public/specimen?ids=BC-ZSM-HYM-25460-A01; catalogNumber: BC-ZSM-HYM-25460-A01; recordNumber: BC-ZSM-HYM-25460-A01; recordedBy: C. Hansson; individualID: BC-ZSM-HYM-25460-A01; individualCount: 1; sex: female; lifeStage: adult; **Taxon:** scientificName: Tetrastichus evexus; phylum: Arthropoda; class: Insecta; order: Hymenoptera; family: Eulophidae; genus: Tetrastichinae; taxonRemarks: Holotype deposited in MZLU; **Location:** country: Sweden; decimalLatitude: 55.6847; decimalLongitude: 13.6767; **Record Level:** type: PhysicalObject; language: en; institutionCode: MZLU; basisOfRecord: PreservedSpecimen
- **Type status:** Paratype. **Occurrence:** occurrenceDetails: http://www.boldsystems.org/index.php/API_Public/specimen?ids=BC-ZSM-HYM-13565-F10; catalogNumber: BC-ZSM-HYM-13565-F10; recordNumber: BC-ZSM-HYM-13565-F10; recordedBy: Bo. W. Svensson; individualID: BC-ZSM-HYM-13565-F10; individualCount: 1; sex: female; lifeStage: adult; **Taxon:** scientificName: Tetrastichus evexus; phylum: Arthropoda; class: Insecta; order: Hymenoptera; family: Eulophidae; genus: Tetrastichinae; **Location:** country: Sweden; decimalLatitude: 55.605; decimalLongitude: 13.004; **Record Level:** type: PhysicalObject; language: en; institutionCode: MZLU; basisOfRecord: PreservedSpecimen
- **Type status:** Paratype. **Occurrence:** occurrenceDetails: http://www.boldsystems.org/index.php/API_Public/specimen?ids=BC-ZSM-HYM-13565-F09; catalogNumber: BC-ZSM-HYM-13565-F09; recordNumber: BC-ZSM-HYM-13565-F09; recordedBy: B. W. Svensson & Co.; individualID: BC-ZSM-HYM-13565-F09; individualCount: 1; sex: female; lifeStage: adult; **Taxon:** scientificName: Tetrastichus evexus; phylum: Arthropoda; class: Insecta; order: Hymenoptera; family: Eulophidae; genus: Tetrastichinae; **Location:** country: Sweden; decimalLatitude: 55.605; decimalLongitude: 13.004; **Record Level:** type: PhysicalObject; language: en; institutionCode: MZLU; basisOfRecord: PreservedSpecimen
- **Type status:** Paratype. **Occurrence:** occurrenceDetails: http://www.boldsystems.org/index.php/API_Public/specimen?ids=BC-ZSM-HYM-25460-A04; catalogNumber: BC-ZSM-HYM-25460-A05; recordNumber: BC-ZSM-HYM-25460-A05; recordedBy: C. Hansson; individualID: BC-ZSM-HYM-25460-A05; individualCount: 1; sex: female; lifeStage: adult; **Taxon:** scientificName: Tetrastichus evexus; phylum: Arthropoda; class: Insecta; order: Hymenoptera; family: Eulophidae; genus: Tetrastichinae; **Location:** country: Sweden; decimalLatitude: 55.6847; decimalLongitude: 13.6767; **Record Level:** type: PhysicalObject; language: en; basisOfRecord: PreservedSpecimen
- **Type status:** Paratype. **Occurrence:** occurrenceDetails: http://www.boldsystems.org/index.php/API_Public/specimen?ids=BC-ZSM-HYM-25460-A04; catalogNumber: BC-ZSM-HYM-25460-A04; recordNumber: BC-ZSM-HYM-25460-A04; recordedBy: C. Hansson; individualID: BC-ZSM-HYM-25460-A04; individualCount: 1; sex: female; lifeStage: adult; **Taxon:** scientificName: Tetrastichus evexus; phylum: Arthropoda; class: Insecta; order: Hymenoptera; family: Eulophidae; genus: Tetrastichinae; **Location:** country: Sweden; decimalLatitude: 55.6847; decimalLongitude: 13.6767; **Record Level:** type: PhysicalObject; language: en; basisOfRecord: PreservedSpecimen
- **Type status:** Paratype. **Occurrence:** occurrenceDetails: http://www.boldsystems.org/index.php/API_Public/specimen?ids=BC-ZSM-HYM-25460-A03; catalogNumber: BC-ZSM-HYM-25460-A03; recordNumber: BC-ZSM-HYM-25460-A03; recordedBy: C. Hansson; individualID: BC-ZSM-HYM-25460-A03; individualCount: 1; sex: female; lifeStage: adult; **Taxon:** scientificName: Tetrastichus evexus; phylum: Arthropoda; class: Insecta; order: Hymenoptera; family: Eulophidae; genus: Tetrastichinae; **Location:** country: Sweden; decimalLatitude: 55.6847; decimalLongitude: 13.6767; **Record Level:** type: PhysicalObject; language: en; basisOfRecord: PreservedSpecimen
- **Type status:** Paratype. **Occurrence:** occurrenceDetails: http://www.boldsystems.org/index.php/API_Public/specimen?ids=BC-ZSM-HYM-25460-A02; catalogNumber: BC-ZSM-HYM-25460-A02; recordNumber: BC-ZSM-HYM-25460-A02; recordedBy: C. Hansson; individualID: BC-ZSM-HYM-25460-A02; individualCount: 1; sex: female; lifeStage: adult; **Taxon:** scientificName: Tetrastichus evexus; phylum: Arthropoda; class: Insecta; order: Hymenoptera; family: Eulophidae; genus: Tetrastichinae; **Location:** country: Sweden; decimalLatitude: 55.6847; decimalLongitude: 13.6767; **Record Level:** type: PhysicalObject; language: en; basisOfRecord: PreservedSpecimen
- **Type status:** Paratype. **Occurrence:** occurrenceDetails: http://www.boldsystems.org/index.php/API_Public/specimen?ids=BC-ZSM-HYM-27770-B11; catalogNumber: BC-ZSM-HYM-27760-B11; recordNumber: BC-ZSM-HYM-27760-B11; recordedBy: J.S. Noyes; individualID: BC-ZSM-HYM-27770-B11; individualCount: 1; sex: M; lifeStage: a; reproductiveCondition: S; associatedMedia: http://www.boldsystems.org/pics/BCHYM/BC-ZSM-HYM-27760-B11+1449268734.jpg; **Taxon:** scientificName: Tetrastichus evexus; phylum: Arthropoda; class: Insecta; order: Hymenoptera; family: Eulophidae; genus: Tetrastichus; **Location:** country: Romania; locality: Breazu MT rzeski; decimalLatitude: 47.2442; decimalLongitude: 27.4828; **Identification:** identifiedBy: Christer Hansson

#### Description

FEMALE holotype (Fig. 25). Body length 2.3 mm (paratypes 1.9–2.0 mm). *Head*. Width/length in dorsal view 2.1, width/length in frontal view 1.3, POL/OOL 2.1, widths head/mesosoma 1.1, mouth width/malar space 1.1, malar space/eye height 1.0. *Antenna*. Scape length/eye height 1.1, pedicel+flagellum length/mesosoma width 1.2, length/width F1, F2, F3 1.8, 2.0, 1.7, clava length/width 3.9, lengths pedicel/F1 0.8, lengths F1/F2 0.9, F1/F3 1.0, lengths F1, F2, F3/clava 0.4, 0.5, 0.4, widths F1/pedicel (dorsal view) 1.1, lengths antennal spicule/C3 0.3. *Mesosoma*. Length/width 1.5, mesoscutal mid-lobe length/width 0.9 (width measured in anterior part), mid-lobe with median groove in posterior ¾, with four adnotaular setae on each side, lengths mesoscutum/mesoscutellum (measured medially) 1.2, mesoscutellum length/width 0.9, length/width of enclosed space between submedian grooves 2.7, distance between submedian/distance between submedian and sublateral grooves 1.4, lengths dorsellum/propodeum (measured medially) 0.5, propodeum with strong reticulation, propodeal callus with nine setae. *Fore wing*. Costal cell length/width 8.0, lengths costal cell/marginal vein 1.0, lengths marginal/stigmal veins 2.6. *Gaster*. Subcircular to ovate, length/width 1.6, lengths gaster/mesosoma 1.1, Gt_7_ length/width 0.4, length of longest cercal seta/next longest seta 1.2, longest cercal setae curved, almost straight, ovipositor sheaths not reaching apex of Gt_7_.

Colour. Body metallic blue-green, scape yellowish-brown with dorsal edge darker, pedicel and flagellum dark brown, tegulae black with metallic tinge, wings hyaline with veins yellowish-white, coxae concolorous with body, trochanters dark brown, fore and mid femora dark brown with metallic tinges and with apical ⅓ yellowish-brown, hind femur concolorous with body, tibiae yellowish-brown, fore tarsus dark brown, mid and hind tarsi yellowish-brown with T4 dark brown.

MALE. Unknown.

#### Diagnosis

Tibiae yellowish-brown; female with malar space 1.0× eye height; scape 3.6× and antennal clava 3.9× as long as wide; mesoscutellum with ratio distance between submedian grooves to distance between submedian and sublateral grooves 1.4. Similar to *T. helviscapus*, but with longer antennal clava in female, 3.9× as long as wide.

#### Distribution

Sweden.

#### Notes

Holotype deposited in MZLU, paratypes in MZLU and NHM.

#### Host

Unknown.

### Tetrastichus flaccius sp. n.

E9A92E73-2348-526B-B915-D0D07B89CFB9

http://zoobank.org/NomenclaturalActs/2353186D-657E-4465-893E-5023654DE141

#### Description

FEMALE holotype (Fig. 26). Body length 2.2 mm. *Head.* Width/length in dorsal view 2.3, width/length in frontal view 1.2, POL/OOL 1.9, widths head/mesosoma 1.0, mouth width/malar space nm, malar space/eye height 0.8. *Antenna.* Scape length/eye height 1.0, pedicel+flagellum length/mesosoma width 1.3, length/width F1, F2, F3 2.2, 2.3, 2.1, clava length/width 4.0, lengths pedicel/F1 0.6, lengths F1/F2 1.0, F1/F3 1.1, lengths F1, F2, F3/clava 0.6, 0.6, 0.5, widths F1/pedicel (dorsal view) 1.1, lengths antennal spicule/C3 0.2. *Mesosoma.* Length/width 1.6, mesoscutal mid-lobe length/width 1.1 (width measured in anterior part), mid-lobe with a weak median groove in posterior ⅔, with 4+5 adnotaular setae, lengths mesoscutum/mesoscutellum (measured medially) 1.4, mesoscutellum length/width 1.1, length/width of enclosed space between submedian grooves 3.1, distance between SMG/distance between SMG and SLG 1.3, lengths dorsellum/propodeum 0.6, propodeum with strong reticulation, propodeal callus with six setae. *Fore wing.* Costal cell length/width 9.0, lengths costal cell/marginal vein 1.0, lengths marginal/stigmal veins 2.7. *Gaster.* Ovate, length/width 1.5, lengths gaster/mesosoma 1.0, Gt_7_ length/width 0.5, length of longest cercal seta/next longest seta 1.5, longest cercal seta curved in apical ⅓, ovipositor sheaths not reaching apex of Gt_7_.

Colour. Body metallic greenish-blue, antenna dark brown, tegulae dark brown, wing venation yellowish-brown, coxae and femora concolorous with body, trochanters dark brown, tibiae yellowish-brown, fore tarsus brown, mid and hind tarsi yellowish-brown with T4 brown.

MALE. Unknown.

#### Diagnosis

Mesoscutellum 1.1× as long as wide with enclosed area between submedian grooves 3.1× as long as wide; length/width F1, F2, F3, clava 2.2, 2.3, 2.1, 4.0; fore wing with marginal vein 2.7× as long as stigmal vein.

#### Distribution

Sweden.

#### Notes

Holotype deposited in SMTP.

#### Host

Unknown.

#### Material examined

Holotype ♀ “SWEDEN, Hälsingland, Hudiksvalls kommun, Stensjön, 62.054°N 16.172°E, 27.vii.2005, SMTP” (SMTP).

### Tetrastichus helviscapus

Graham, 1991

F44DED4E-32AA-511D-8F83-BC9512125F5F

Tetrastichus helviscapus Graham 1991:242–243. Holotype ♀ in NHM (NHM type no. 5.3617), examined (Fig. 27).

#### Description

See Graham (1991).

#### Diagnosis

Tibiae yellowish-brown; female with malar space 0.7× eye height; scape 4.0× and antennal clava 3.0× as long as wide; mesoscutellum with ratio distance between submedian grooves to between submedian and sublateral grooves 1.4 and enclosed space between submedian grooves 2.7× as long as wide.

#### Distribution

Moldova, Russia, United Kingdom, (former) Yugoslavia (Graham 1991); Sweden (Hedqvist 2003); France and Morocco (**new records**).

#### Host

Unknown.

#### Additional material examined

France, 2♀ (GD); Morocco, 2♀ (GD).

### Tetrastichus hylotomarum

(Bouché, 1834)

12091262-3978-5E97-A40D-FA7B9EB57E7C

Tetrastichus hylotomarum *EulophushylotomarumBouché 1834*Graham (1991)28*Tetrastichus*Erdös 1956*Aprostocetus*Graham 1961*Tetrastichus*Domenichini 1966

#### Description

See Graham (1991).

#### Diagnosis

Female antenna: F3 1.0–1.5× as long as wide, clava 2.6–3.0× as long as wide; male antenna: funiculars without an externo-dorsal, sub-basal compact whorl of long setae, F1 1.1–1.4× as long as wide and about as long as pedicel, distinctly shorter than F2; both sexes with mid and hind tibiae broadly infuscate, sometimes mainly black; body bright metallic green.

#### Distribution

Bulgaria, (former) Czechoslovakia, France, Germany, Hungary, The Netherlands, Russia, Sweden and United Kingdom (Graham 1991); Italy and Romania (**new records**).

#### Host

Reared from *Arge ochropus* (Gmelin), *A. pagana* (Panzer) (Hymenoptera: Argidae), *Athalia cordata* Audinet-Serville, *Cladius pectinicornis* (Geoffroy) (Hymenoptera: Tenthredinidae), parasitising host larvae and pupae (Graham 1991).

#### Additional material examined

11♀ 2♂: France 3♀ (NHM), Italy 1♀ (ZSM), Romania 1♀ (NHM), Sweden 6♀ 2♂ (MZLU, NHM, SMTP).

### Tetrastichus iasi sp. n.

AB4459AD-32D6-50A0-AB07-CEC15083F794

urn:lsid:zoobank.org:act:4534B329-B4E9-4E00-9B2D-BC76C917A3F2

#### Materials

- **Type status:** Holotype. **Occurrence:** occurrenceDetails: http://www.boldsystems.org/index.php/API_Public/specimen?ids=BC-ZSM-HYM-27768-E02; catalogNumber: BC-ZSM-HYM-27768-E02; recordNumber: BC-ZSM-HYM-27768-E02; recordedBy: J.S. Noyes; individualID: BC-ZSM-HYM-27768-E02; individualCount: 1; sex: female; lifeStage: adult; **Taxon:** scientificName: Tetrastichus iasi; phylum: Arthropoda; class: Insecta; order: Hymenoptera; family: Eulophidae; genus: Tetrastichinae; taxonRemarks: Holotype deposited in NHM; **Location:** country: Romania; decimalLatitude: 47.188; decimalLongitude: 27.549; **Record Level:** type: PhysicalObject; language: en; institutionCode: NHM; basisOfRecord: PreservedSpecimen
- **Type status:** Paratype. **Occurrence:** occurrenceDetails: http://www.boldsystems.org/index.php/API_Public/specimen?ids=BC-ZSM-HYM-27768-B02; catalogNumber: BC-ZSM-HYM-27768-B02; recordNumber: BC-ZSM-HYM-27768-B02; recordedBy: J.S. Noyes; individualID: BC-ZSM-HYM-27768-B02; individualCount: 1; sex: male; lifeStage: adult; **Taxon:** scientificName: Tetrastichus iasi; phylum: Arthropoda; class: Insecta; order: Hymenoptera; family: Eulophidae; genus: Tetrastichinae; **Location:** country: Romania; decimalLatitude: 47.011; decimalLongitude: 27.603; **Record Level:** type: PhysicalObject; language: en; institutionCode: NHM; basisOfRecord: PreservedSpecimen

#### Description

FEMALE holotype (Fig. 29). Body length 1.9 mm. *Head.* Width/length in dorsal view 2.0, width/length in frontal view 1.3, POL/OOL 1.8, widths head/mesosoma 1.1, mouth width/malar space 0.9, malar space/eye height 0.9. *Antenna.* Scape length/eye height 1.0, pedicel+flagellum length/mesosoma width 1.2, length/width F1, F2, F3 2.0, 1.5, 1.3, clava length/width 3.3, lengths of pedicel/F1 0.8, lengths F1/F2 1.1, F1/F3 1.2, lengths F1, F2, F3/clava 0.5, 0.5, 0.4, widths F1/pedicel (dorsal view) 1.0, lengths antennal spicule/C3 0.2. *Mesosoma.* Length/width 1.5, mesoscutal mid-lobe length/width 1.0 (width measured in anterior part), mid-lobe with a weak median groove that is missing in anterior ¼, with seven adnotaular setae on each side, lengths mesoscutum/mesoscutellum (measured medially) 1.4, dorsellum/propodeum length 0.5, mesoscutellum length/width 0.9, length/width of enclosed space between submedian grooves 2.5, distance between SMG/distance between SMG and SLG 1.5, propodeum with strong reticulation, propodeal callus with five setae. *Fore wing.* Costal cell length/width 10.7, lengths costal cell/marginal vein 1.0, lengths marginal/stigmal veins 3.1. *Gaster.* Short ovate, length/width 1.5, lengths gaster/mesosoma 1.1, Gt_7_ length/width 0.4, lengths longest cercal seta/next longest seta 1.8, longest cercal seta straight, ovipositor sheaths not reaching apex of Gt_7_.

Colour. Body metallic bluish-green, gaster with purple tinges, scape yellowish-brown, flagellum and pedicel dark brown, tegulae black with metallic tinge, wings hyaline and venation yellowish-white, coxae and femora concolorous with body, trochanters black, tibiae yellowish-brown, fore tarsus with T1–3 brown and T4 dark brown, mid and hind tarsi with T1–3 yellowish-brown and T4 dark brown.

MALE (Fig. 29). Body length 1.5 mm. *Head.* Width/length in dorsal view 2.2, width/length in frontal view 1.2, mouth width/malar space 1.0, widths head/mesosoma 1.2. *Antenna.* F1–F4 without basal whorls of setae, scape length/eye height 1.0, scape length/width 3.2, lengths ventral plaque/scape 0.6, plaque placed in central part of scape, pedicel+flagellum length/mesosoma width 1.6, length/width F1, F2, F3, F4 1.6, 1.9, 1.9, 1.8, clava length/width 3.3, lengths pedicel/F1 0.9, lengths F1/F2 0.9, F1/F3 0.9, F1/F4 0.9, lengths F1, F2, F3, F4/clava 0.4, 0.5, 0.5, 0.5.

Colour. Scape dark brown. Otherwise similar to female.

#### Diagnosis

Mesoscutellum with submedian grooves diverging towards posterior part; female antenna with clava 3.3× as long as wide.

#### Distribution

Romania.

#### Notes

Holotype and paratype deposited in NHM.

#### Host

Unknown.

### Tetrastichus illydris sp. n.

C998B021-7FDA-5970-AD3C-58958EF26E56

urn:lsid:zoobank.org:act:E6AC6D4A-3CD4-484C-8A1B-751E3DB94A9B

#### Materials

- **Type status:** Holotype. **Occurrence:** occurrenceDetails: http://www.boldsystems.org/index.php/API_Public/specimen?ids=BC-ZSM-HYM-20699-G06; catalogNumber: BC-ZSM-HYM-20699-G06; recordNumber: BC-ZSM-HYM-20699-G06; recordedBy: D. Doczkal & A. Segerer; individualID: BC-ZSM-HYM-20699-G06; individualCount: 1; sex: female; lifeStage: adult; **Taxon:** scientificName: Tetrastichus illydris; phylum: Arthropoda; class: Insecta; order: Hymenoptera; family: Eulophidae; genus: Tetrastichinae; taxonRemarks: Holotype deposited in ZSM; **Location:** country: Germany; decimalLatitude: 49.03; decimalLongitude: 12.157; **Record Level:** type: PhysicalObject; language: en; institutionCode: ZSM; basisOfRecord: PreservedSpecimen
- **Type status:** Paratype. **Occurrence:** occurrenceDetails: http://www.boldsystems.org/index.php/API_Public/specimen?ids=BC-ZSM-HYM-22523-H07; catalogNumber: BC-ZSM-HYM-22523-H07; recordNumber: BC-ZSM-HYM-22523-H07; recordedBy: Popovici & Fusu; individualID: BC-ZSM-HYM-22523-H07; individualCount: 1; sex: female; lifeStage: adult; **Taxon:** scientificName: Tetrastichus illydris; phylum: Arthropoda; class: Insecta; order: Hymenoptera; family: Eulophidae; genus: Tetrastichinae; **Location:** country: Romania; decimalLatitude: 47.05; decimalLongitude: 27.384; **Record Level:** type: PhysicalObject; language: en; institutionCode: MZLU; basisOfRecord: PreservedSpecimen

#### Description

FEMALE holotype (Fig. 30). Body length 2.1 mm (paratype 1.9 mm). *Head.* Width/length in dorsal view 2.2, width/length in frontal view 1.3, POL/OOL 2.0, widths head/mesosoma 1.0, mouth width/malar space 1.0, malar space/eye height 0.8. *Antenna.* Scape length/eye height 1.0, pedicel+flagellum length/mesosoma width 1.2, length/width F1, F2, F3 2.1, 2.0, 1.5, clava length/width 3.3, lengths pedicel/F1 0.7, lengths F1/F2 0.9, F1/F3 1.1, lengths F1, F2, F3/clava 0.5, 0.6, 0.5, width F1/pedicel (dorsal view) 1.0, lengths antennal spicule/C3 0.3. *Mesosoma.* Length/width 1.4, mesoscutal mid-lobe length/width 0.9 (width measured in anterior part), mid-lobe with a weak median groove that is absent in anterior ¼, with six adnotaular setae on each side, lengths mesoscutum/mesoscutellum (measured medially) 1.2, lengths dorsellum/propodeum 0.8, mesoscutellum length/width 0.8, length/width of enclosed space between submedian grooves 2.6, distance between SMG/distance between SMG and SLG 1.3, propodeum with weak reticulation, propodeal callus with five setae. *Fore wing.* Costal cell length/width 10.0, lengths costal cell/marginal vein 1.2, lengths marginal/stigmal veins 2.9. *Gaster.* Semicircular, length/width 1.6, lengths gaster/mesosoma 1.2, Gt_7_ length/width 0.3, lengths longest cercal seta/next longest seta 1.5, ovipositor sheaths not reaching apex of Gt_7_.

Colour. Mesoscutum metallic blue-green and mesoscutellum golden-green, scape yellowish-brown, pedicel and flagellum dark brown, tegulae black with metallic tinges, wing venation yellowish-white, coxae concolorous with body, trochanters dark brown, fore and mid femora dark brown with apex yellowish-brown, hind femur black with metallic tinge and apex yellowish-brown, tibiae yellowish-brown, tarsi with T1–3 yellowish-brown and T4 brown.

MALE. Unknown.

#### Diagnosis

Mesoscutellum 0.8× as long as wide, length/width of enclosed space between submedian grooves 2.6; mesoscutum bluish and mesoscutellum greenish; antennal clava 3.3× as long as wide.

#### Distribution

Germany and Romania.

#### Notes

Holotype deposited in ZSM, paratype in MZLU.

#### Host

Unknown.

### Tetrastichus inaequalis

Graham, 1991

765EE9C6-ED50-5594-926A-C7738EB9ED21

Tetrastichus inaequalis Graham 1991:241–242. Holotype ♀ in NHM, examined (Fig. 31).

#### Description

See Graham (1991).

#### Diagnosis

Antenna with F1 0.8× as long as F2 and only very slightly longer than the pedicel, F3 1.9× as long as wide; tibiae yellowish-brown; body bright metallic green.

#### Distribution

United Kingdom (Graham 1991), France and Sweden (**new records**).

#### Host

Unknown.

#### Additional material examined

4♀: France 2♀ (NHM), Sweden 1♀ (MZLU), United Kingdom 1♀ (NHM).

### Tetrastichus incanus sp. n.

772E3F7C-5295-57C7-98DA-08DAD69CB7D9

urn:lsid:zoobank.org:act:A0E15318-3134-4D4F-966C-844B49CE5560

#### Materials

- **Type status:** Holotype. **Occurrence:** occurrenceDetails: http://www.boldsystems.org/index.php/API_Public/specimen?ids=BC-ZSM-HYM-21587-G06; catalogNumber: BC-ZSM-HYM-21587-G06; recordNumber: BC-ZSM-HYM-21587-G06; recordedBy: C.Hansson; individualID: BC-ZSM-HYM-21587-G06; individualCount: 1; sex: female; lifeStage: adult; **Taxon:** scientificName: Tetrastichus incanus; phylum: Arthropoda; class: Insecta; order: Hymenoptera; family: Eulophidae; genus: Tetrastichinae; taxonRemarks: Holotype deposited in MZLU; **Location:** country: Sweden; decimalLatitude: 55.792; decimalLongitude: 13.654; **Record Level:** type: PhysicalObject; language: en; institutionCode: MZLU; basisOfRecord: PreservedSpecimen
- **Type status:** Paratype. **Occurrence:** occurrenceDetails: http://www.boldsystems.org/index.php/API_Public/specimen?ids=BC-ZSM-HYM-29751-B08; catalogNumber: BC-ZSM-HYM-29751-B08; recordNumber: BC-ZSM-HYM-29751-B08; recordedBy: C. Hansson; individualID: BC-ZSM-HYM-29751-B08; individualCount: 1; sex: female; lifeStage: adult; **Taxon:** scientificName: Tetrastichus incanus; phylum: Arthropoda; class: Insecta; order: Hymenoptera; family: Eulophidae; genus: Tetrastichinae; **Location:** country: Sweden; decimalLatitude: 55.6861; decimalLongitude: 13.4611; **Record Level:** type: PhysicalObject; language: en; basisOfRecord: PreservedSpecimen
- **Type status:** Paratype. **Occurrence:** occurrenceDetails: http://www.boldsystems.org/index.php/API_Public/specimen?ids=BC-ZSM-HYM-22523-D11; catalogNumber: BC-ZSM-HYM-22523-D11; recordNumber: BC-ZSM-HYM-22523-D11; recordedBy: Swedish Malaise Trap Project; individualID: BC-ZSM-HYM-22523-D11; individualCount: 1; sex: female; lifeStage: adult; **Taxon:** scientificName: Tetrastichus incanus; phylum: Arthropoda; class: Insecta; order: Hymenoptera; family: Eulophidae; genus: Tetrastichinae; **Location:** country: Sweden; decimalLatitude: 56.943; decimalLongitude: 15.946; **Record Level:** type: PhysicalObject; language: en; basisOfRecord: PreservedSpecimen
- **Type status:** Paratype. **Occurrence:** occurrenceDetails: http://www.boldsystems.org/index.php/API_Public/specimen?ids=BC-ZSM-HYM-29814-B02; catalogNumber: BC-ZSM-HYM-29814-B02; recordNumber: BC-ZSM-HYM-29814-B02; recordedBy: C. Hansson; individualID: BC-ZSM-HYM-29814-B02; individualCount: 1; sex: female; lifeStage: adult; **Taxon:** scientificName: Tetrastichus incanus; phylum: Arthropoda; class: Insecta; order: Hymenoptera; family: Eulophidae; genus: Tetrastichinae; **Location:** country: Sweden; decimalLatitude: 55.705; decimalLongitude: 13.4886; **Record Level:** type: PhysicalObject; language: en; basisOfRecord: PreservedSpecimen
- **Type status:** Paratype. **Occurrence:** occurrenceDetails: http://www.boldsystems.org/index.php/API_Public/specimen?ids=BC-ZSM-HYM-26563-F08; catalogNumber: BC-ZSM-HYM-26563-F08; recordNumber: BC-ZSM-HYM-26563-F08; recordedBy: C. Hansson; individualID: BC-ZSM-HYM-26563-F08; individualCount: 1; sex: male; lifeStage: adult; **Taxon:** scientificName: Tetrastichus incanus; phylum: Arthropoda; class: Insecta; order: Hymenoptera; family: Eulophidae; genus: Tetrastichinae; **Location:** country: Sweden; decimalLatitude: 55.6619; decimalLongitude: 13.5472; **Record Level:** type: PhysicalObject; language: en; basisOfRecord: PreservedSpecimen
- **Type status:** Paratype. **Occurrence:** occurrenceDetails: http://www.boldsystems.org/index.php/API_Public/specimen?ids=BC-ZSM-HYM-22523-D10; catalogNumber: BC-ZSM-HYM-22523-D10; recordNumber: BC-ZSM-HYM-22523-D10; recordedBy: Swedish Malaise Trap Project; individualID: BC-ZSM-HYM-22523-D10; individualCount: 1; sex: female; lifeStage: adult; **Taxon:** scientificName: Tetrastichus incanus; phylum: Arthropoda; class: Insecta; order: Hymenoptera; family: Eulophidae; genus: Tetrastichinae; **Location:** country: Sweden; decimalLatitude: 56.943; decimalLongitude: 15.946; **Record Level:** type: PhysicalObject; language: en; basisOfRecord: PreservedSpecimen
- **Type status:** Paratype. **Occurrence:** occurrenceDetails: http://www.boldsystems.org/index.php/API_Public/specimen?ids=BC-ZSM-HYM-27493-H01; catalogNumber: BC-ZSM-HYM-27493-H01; recordNumber: BC-ZSM-HYM-27493-H01; recordedBy: SMTP; individualID: BC-ZSM-HYM-27493-H01; individualCount: 1; sex: female; lifeStage: adult; **Taxon:** scientificName: Tetrastichus incanus; phylum: Arthropoda; class: Insecta; order: Hymenoptera; family: Eulophidae; genus: Tetrastichinae; **Location:** country: Sweden; decimalLatitude: 63.122; decimalLongitude: 15.07; **Record Level:** type: PhysicalObject; language: en; basisOfRecord: PreservedSpecimen
- **Type status:** Paratype. **Occurrence:** occurrenceDetails: http://www.boldsystems.org/index.php/API_Public/specimen?ids=BC-ZSM-HYM-22523-E04; catalogNumber: BC-ZSM-HYM-22523-E04; recordNumber: BC-ZSM-HYM-22523-E04; recordedBy: Swedish Malaise Trap Project; individualID: BC-ZSM-HYM-22523-E04; individualCount: 1; sex: female; lifeStage: adult; **Taxon:** scientificName: Tetrastichus incanus; phylum: Arthropoda; class: Insecta; order: Hymenoptera; family: Eulophidae; genus: Tetrastichinae; **Location:** country: Sweden; decimalLatitude: 56.943; decimalLongitude: 15.946; **Record Level:** type: PhysicalObject; language: en; basisOfRecord: PreservedSpecimen
- **Type status:** Paratype. **Occurrence:** occurrenceDetails: http://www.boldsystems.org/index.php/API_Public/specimen?ids=BC-ZSM-HYM-29813-G08; catalogNumber: BC-ZSM-HYM-29813-G08; recordNumber: BC-ZSM-HYM-29813-G08; recordedBy: A.Jansson; individualID: BC-ZSM-HYM-29813-G08; individualCount: 1; sex: F; lifeStage: Adult; associatedMedia: http://www.boldsystems.org/pics/BCHYM/BC-ZSM-HYM-29813-G08+1516986306.jpg; **Taxon:** scientificName: Tetrastichus incanus; phylum: Arthropoda; class: Insecta; order: Hymenoptera; family: Eulophidae; genus: Tetrastichus; **Location:** country: Sweden; locality: Oerebro; decimalLatitude: 59.2753; decimalLongitude: 15.2134; **Identification:** identifiedBy: Christer Hansson
- **Type status:** Paratype. **Occurrence:** occurrenceDetails: http://www.boldsystems.org/index.php/API_Public/specimen?ids=BC-ZSM-HYM-29813-H01; catalogNumber: BC-ZSM-HYM-29813-H01; recordNumber: BC-ZSM-HYM-29813-H01; recordedBy: A.Jansson; individualID: BC-ZSM-HYM-29813-H01; individualCount: 1; sex: M; lifeStage: Adult; associatedMedia: http://www.boldsystems.org/pics/BCHYM/BC-ZSM-HYM-29813-H01+1516986310.jpg; **Taxon:** scientificName: Tetrastichus incanus; phylum: Arthropoda; class: Insecta; order: Hymenoptera; family: Eulophidae; genus: Tetrastichus; **Location:** country: Sweden; locality: Oerebro; decimalLatitude: 59.2753; decimalLongitude: 15.2134; **Identification:** identifiedBy: Christer Hansson
- **Type status:** Paratype. **Occurrence:** occurrenceDetails: http://www.boldsystems.org/index.php/API_Public/specimen?ids=BC-ZSM-HYM-29813-A02; catalogNumber: BC-ZSM-HYM-29813-A02; recordNumber: BC-ZSM-HYM-29813-A02; recordedBy: A.Jansson; individualID: BC-ZSM-HYM-29813-A02; individualCount: 1; sex: F; lifeStage: Adult; associatedMedia: http://www.boldsystems.org/pics/BCHYM/BC-ZSM-HYM-29813-A02+1516986318.jpg; **Taxon:** scientificName: Tetrastichus incanus; phylum: Arthropoda; class: Insecta; order: Hymenoptera; family: Eulophidae; genus: Tetrastichus; **Location:** country: Sweden; locality: Bispgarden; decimalLatitude: 63.0271; decimalLongitude: 16.6245; **Identification:** identifiedBy: Christer Hansson

#### Description

FEMALE holotype (Fig. 32). Body length 2.2 mm (paratypes 1.5–2.2 mm). *Head*. Width/length in dorsal view 2.3, width/length in frontal view 1.3, POL/OOL 2.1, widths head/mesosoma 1.0, mouth width/malar space 1.1, malar space/eye height 0.9. *Antenna*. Scape length/eye height 1.0, pedicel+flagellum length/mesosoma width 1.3, length/width F1, F2, F3 2.2, 2.5, 2.2, clava length/width 3.8, lengths pedicel/F1 0.5, lengths F1/F2 1.0, F1/F3 1.1, lengths F1, F2, F3/clava 0.5, 0.5, 0.5, widths F1/pedicel (dorsal view) 1.1, lengths antennal spicule/C3 0.3. *Mesosoma*. Length/width 1.5, mesoscutal mid-lobe length/width 0.9 (width measured in anterior part), mid-lobe with median groove in posterior ¾, with six adnotaular setae on each side, lengths mesoscutum/mesoscutellum (measured medially) 1.1, mesoscutellum length/width 0.9, length/width of enclosed space between submedian grooves 2.6, distance between SMG/distance between SMG and SLG 1.6, lengths dorsellum/propodeum (measured medially) 0.7, propodeum with strong reticulation, propodeal callus with three setae. *Fore wing*. Costal cell length/width 9.0, lengths costal cell/marginal vein 1.0, lengths marginal/stigmal veins 3.1. *Gaster*. Subcircular to ovate, length/width 1.5, lengths gaster/mesosoma 1.1, Gt_7_ length/width 0.3, length of longest cercal seta/next longest seta 1.4, longest cercal seta straight, ovipositor sheaths not reaching apex of Gt_7_.

Colour. Body golden-green, scape yellowish-brown, pedicel and flagellum dark brown, tegulae black with metallic tinge, wings hyaline with veins yellowish-white, coxae and femora concolorous with body, trochanters black, tibiae yellowish-brown, tarsi yellowish-brown, mid and hind tarsi with T4 brown.

Variation. Paratypes with body metallic blue or blue-green.

MALE (Fig. 32). Body length 1.7 mm. *Head*. Width/length in dorsal view 2.2, width/length in frontal view 1.3, eye height/malar space 1.2, mouth width/malar space 1.0, widths head/mesosoma 1.1. *Antenna*. F1–F4 without basal whorls of setae, scape length/eye height 1.0, scape length/width 2.7, ventral plaque placed in central part of scape, lengths ventral plaque/scape 0.7, pedicel+flagellum length/mesosoma width 1.7, length/width F1, F2, F3, F4 1.9, 2.3, 2.4, 2.4, clava length/width 4.9, lengths pedicel/F1 0.8, lengths F1/F2 0.8, F1/F3 0.8, F1/F4 0.8, lengths F1, F2, F3, F4/clava 0.3, 0.4, 0.4, 0.4.

Colour. Similar to female, but with scape dark brown. Body metallic blue-green.

#### Diagnosis

Mid and hind tibiae yellowish-brown; mesoscutellum with distance between submedian grooves 1.6× distance between submedian and sublateral grooves; female with malar space 0.9× height of eye, antenna with F2 2.5×, F3 2.2× and clava 3.8× as long as wide; male antenna with scape 2.7× and clava 4.9× as long as wide and scape length 1.0× height of eye.

#### Distribution

Sweden.

#### Notes

Holotype deposited in MZLU, paratypes in MZLU, SMTP.

#### Host

Unknown.

### Tetrastichus johnnoyesi sp. n.

719FC9FD-9F0B-5EE0-91D9-1A8025748DCD

urn:lsid:zoobank.org:act:C919F453-A59B-479D-ABB0-E995120A51C5

#### Materials

- **Type status:** Holotype. **Occurrence:** occurrenceDetails: http://www.boldsystems.org/index.php/API_Public/specimen?ids=BC-ZSM-HYM-27768-D08; catalogNumber: BC-ZSM-HYM-27768-D08; recordNumber: BC-ZSM-HYM-27768-D08; recordedBy: J.S. Noyes; individualID: BC-ZSM-HYM-27768-D08; individualCount: 1; sex: female; lifeStage: adult; **Taxon:** scientificName: Tetrastichus johnnoyesi; phylum: Arthropoda; class: Insecta; order: Hymenoptera; family: Eulophidae; genus: Tetrastichinae; taxonRemarks: Holotype deposited in NHM; **Location:** country: Romania; decimalLatitude: 47.298; decimalLongitude: 25.368; **Record Level:** type: PhysicalObject; language: en; institutionCode: NHM; basisOfRecord: PreservedSpecimen
- **Type status:** Paratype. **Occurrence:** occurrenceDetails: http://www.boldsystems.org/index.php/API_Public/specimen?ids=BC-ZSM-HYM-27768-D12; catalogNumber: BC-ZSM-HYM-27768-D12; recordNumber: BC-ZSM-HYM-27768-D12; recordedBy: J.S. Noyes; individualID: BC-ZSM-HYM-27768-D12; individualCount: 1; sex: female; lifeStage: adult; **Taxon:** scientificName: Tetrastichus johnnoyesi; phylum: Arthropoda; class: Insecta; order: Hymenoptera; family: Eulophidae; genus: Tetrastichinae; **Location:** country: Romania; decimalLatitude: 47.298; decimalLongitude: 25.368; **Record Level:** type: PhysicalObject; language: en; basisOfRecord: PreservedSpecimen
- **Type status:** Paratype. **Occurrence:** occurrenceDetails: http://www.boldsystems.org/index.php/API_Public/specimen?ids=BC-ZSM-HYM-27768-D10; catalogNumber: BC-ZSM-HYM-27768-D10; recordNumber: BC-ZSM-HYM-27768-D10; recordedBy: J.S. Noyes; individualID: BC-ZSM-HYM-27768-D10; individualCount: 1; sex: male; lifeStage: adult; **Taxon:** scientificName: Tetrastichus johnnoyesi; phylum: Arthropoda; class: Insecta; order: Hymenoptera; family: Eulophidae; genus: Tetrastichinae; **Location:** country: Romania; decimalLatitude: 47.298; decimalLongitude: 25.368; **Record Level:** type: PhysicalObject; language: en; basisOfRecord: PreservedSpecimen
- **Type status:** Paratype. **Occurrence:** occurrenceDetails: http://www.boldsystems.org/index.php/API_Public/specimen?ids=BC-ZSM-HYM-27768-D09; catalogNumber: BC-ZSM-HYM-27768-D09; recordNumber: BC-ZSM-HYM-27768-D09; recordedBy: J.S. Noyes; individualID: BC-ZSM-HYM-27768-D09; individualCount: 1; sex: female; lifeStage: adult; **Taxon:** scientificName: Tetrastichus johnnoyesi; phylum: Arthropoda; class: Insecta; order: Hymenoptera; family: Eulophidae; genus: Tetrastichinae; **Location:** country: Romania; decimalLatitude: 47.298; decimalLongitude: 25.368; **Record Level:** type: PhysicalObject; language: en; basisOfRecord: PreservedSpecimen

#### Description

FEMALE holotype (Fig. 33). Body length 2.5 mm (paratypes 2.2 mm). *Head.* Width/length in dorsal view 2.1, width/length in frontal view 1.3, POL/OOL 2.1, widths head/mesosoma 1.1, mouth width/malar space 1.1, malar space/eye height 0.8. *Antenna.* Scape length/eye height 1.1, pedicel+flagellum length/mesosoma width 1.1, length/width F1, F2, F3 2.0, 1.9, 1.7, clava length/width 3.2, lengths pedicel/F1 0.7, lengths F1/F2 1.1, F1/F3 1.2, lengths F1, F2, F3/clava 0.6, 0.5, 0.5, widths F1/pedicel (dorsal view) 1.0, lengths antennal spicule/C3 0.3. *Mesosoma.* Length/width 1.7, mesoscutal mid-lobe length/width 1.0 (width measured in anterior part), mid-lobe with median groove, with four adnotaular setae on either side, length of mesoscutum/mesoscutellum (measured medially) 1.3, lengths dorsellum/propodeum 0.6, mesoscutellum length/width 1.0, length/width of enclosed space between submedian grooves 3.3, distance between SMG/distance between SMG and SLG 1.1, propodeum with strong reticulation, propodeal callus with four setae. *Fore wing.* Costal cell length/width 8.2, lengths costal cell/marginal vein 1.0, lengths marginal/stigmal veins 2.4. *Gaster.* Ovate, length/width 1.8, lengths gaster/mesosoma 1.1, Gt_7_ length/width 0.5, length of longest cercal seta/next longest seta 1.4, longest cercal seta straight, ovipositor sheaths not reaching apex of Gt_7_.

Colour. Body golden-green, scape yellowish-brown, pedicel brownish, flagellum dark brown, tegulae black with metallic tinge, wings hyaline with venation yellowish-white, coxae and femora concolorous with body, trochanters black, tibiae and tarsi yellowish-brown.

MALE (Fig. 33). Body length 2.0 mm. *Head.* Width/length in dorsal view 2.3, width/length in frontal view 1.3, mouth width/malar space 1.1, widths head/mesosoma 1.1. *Antenna.* F1–F4 without basal whorls of setae, scape length/eye height 1.1, scape length/width 3.0, ventral plaque placed in central part of scape, lengths of ventral plaque/scape 0.7, pedicel+flagellum length/mesosoma width 1.4, length/width F1, F2, F3, F4 1.2, 1.9, 1.9, 1.9, clava length/width 3.8, lengths pedicel/F1 1.1, lengths F1/F2 0.6, F1/F3 0.6, F1/F4 0.6, lengths F1, F2, F3, F4/clava 0.3, 0.5, 0.5, 0.5.

Colour. Scape black. Otherwise similar to female.

#### Diagnosis

Mesoscutellum with ratio length/width of enclosed space between submedian grooves 3.3; antennal clava 3.2× as long as wide.

#### Etymology

Named after John S. Noyes (NHM), collector of the type specimens.

#### Distribution

France and Romania.

#### Notes

Holotype and paratypes deposited NHM.

#### Host

Unknown.

#### Additional paratypes

3♀ 1♂: 2♀ 1♂ with same label data as holotype (NHM, MZLU); 1♀ “FRANCE, B. du Rhone, Fonscolombe, 7.vii.1990, M.W.R. de V. Graham” (NHM).

### Tetrastichus marcusgrahami sp. n.

CB502DCC-E538-53DB-99D9-E186B97296E2

urn:lsid:zoobank.org:act:94214DE9-0044-432B-B3BC-5BF726C647B0

#### Description

FEMALE holotype (Fig. 34). Body length 2.6 mm. *Head.* Width/length in dorsal view 2.4, width/length in frontal view 1.3, POL/OOL 1.3, head/mesosoma width 1.1, mouth width/malar space 1.9, malar space/eye height 0.8. *Antenna.* Scape length/eye height 1.0, pedicel+flagellum length/mesosoma width 1.1, length/width F1, F2, F3 1.8, 1.4, 1.2, clava length/width 2.8, lengths pedicel/F1 0.6, lengths F1/F2 1.2, F1/F3 1.3, lengths F1, F2, F3/clava 0.5, 0.5, 0.4, width F1/pedicel (dorsal view) 1.2, lengths antennal spicule/C3 0.2. *Mesosoma.* Length/width 1.5, mesoscutal mid-lobe length/width 1.0 (width measured in anterior part), mid-lobe with a weak median groove in median ⅓, with five adnotaular setae on each side, lengths mesoscutum/mesoscutellum (measured medially) 1.2, lengths dorsellum/propodeum 0.5, mesoscutellum length/width 1.1, length/width of enclosed space between submedian grooves 2.8, distance between SMG/distance between SMG and SLG 1.5, propodeum with strong reticulation in anterior ½, smooth in posterior ½, propodeal callus with five setae. *Fore wing.* Costal cell length/width 9.1, lengths costal cell/marginal vein 1.1, lengths marginal/stigmal veins 3.0. *Gaster.* Ovate, length/width 1.4, lengths gaster/mesosoma 1.1, Gt_7_ length/width 0.6, lengths longest cercal seta/next longest seta 1.3, longest cercal seta curved, ovipositor sheaths not reaching apex of Gt_7_.

Colour. Body golden-green, scape golden-green, pedicel and flagellum dark brown, tegulae black with metallic tinges, wings hyaline, wing venation brown to fuscous, coxae and femora concolorous with body, trochanters blackish, tibiae yellowish-brown, tarsi dark brown.

MALE. Unknown.

#### Diagnosis

Mandibles as in *T. halidayi*, i.e. mandibles very large with outer tooth falcate and separated by a wide gap from the two small inner teeth (as in Figs. 2a and 65d); mouth opening large, 1.9× as wide as malar space. Differs from *T. halidayi* in having a shorter female gaster with ovipositor retracted and apex not reaching apical margin of gaster. Similar also to *T. cyprus*, from which it differs in antennal characters as indicated in the key.

#### Etymology

Named after collector of type specimen, Marcus William Robert de Vere Graham.

#### Distribution

United Kingdom.

#### Notes

The head of the holotype is detached and glued separately on a card and the right antennal flagellum is missing (deposited in NHM).

#### Host

Unknown.

#### Material examined

Holotype ♀ “ENGLAND: Lancashire, Freshfield (3) (Area I), 3.vi.1959”, “M.W.R. de V. Graham coll., BMNH(E) 1995-489” (NHM).

### Tetrastichus splendens sp. n.

39C85AF5-ABAC-56A2-8BBA-AAA6FDD266C9

urn:lsid:zoobank.org:act:F8D4BA07-CC57-4062-9C4E-4E71BD04C6E4

#### Description

FEMALE holotype (Fig. 35). Body length 2.3 mm. *Head.* Width/length in dorsal view 2.3, width/length in frontal view 1.2, POL/OOL 2.1, widths head/mesosoma 1.0, mouth width/malar space 1.0, malar space/eye height 0.9. *Antenna.* Scape length/eye height 1.1, pedicel+flagellum length/mesosoma width 1.1, length/width F1, F2, F3 1.7, 1.6, 1.3, clava length/width 2.5, lengths pedicel/F1 0.7, lengths F1/F2 1.1, F1/F3 1.3, lengths F1, F2, F3/clava 0.5, 0.5, 0.4, width F1/pedicel (dorsal view) 1.2, lengths antennal spicule/C3 0.1. *Mesosoma.* Length/width 1.5, mesoscutal mid-lobe length/width 0.9 (width measured in anterior part), mid-lobe with a weak median groove that is missing in anterior ¼, with four adnotaular setae on each side, lengths mesoscutum/mesoscutellum (measured medially) 1.3, mesoscutellum length/width 1.1, length/width of enclosed space between submedian grooves 3.5, distance between SMG/distance between SMG and SLG 1.1, lengths dorsellum/propodeum 0.5, propodeum with strong reticulation, propodeal callus with five setae. *Fore wing.* Costal cell length/width 8.8, lengths costal cell/marginal vein 1.2, lengths marginal/stigmal veins 2.2. *Gaster.* Semicircular, length/width 1.2, lengths gaster/mesosoma 1.0, Gt_7_ length/width 0.5, lengths longest cercal seta/next longest seta 1.3, longest cercal seta straight, ovipositor sheaths not reaching apex of Gt_7_.

Colour. Body golden-green, scape yellow, pedicel yellowish-brown, flagellum dark brown, tegulae black with metallic tinges, wings hyaline, wing venation yellowish-white, coxae concolorous with body, trochanters dark brown, femora dark brown with golden-green tinges and with apex yellowish-brown, fore tarsus dark yellowish-brown, mid and hind tarsi yellowish-brown with T4 dark brown.

MALE. Unknown.

#### Diagnosis

Flagellum short and stout, length/width F1, F2, F2, clava: 1.7, 1.6, 1.3, 2.5; malar space 0.9× eye height; mesoscutellum with ratio length/width of enclosed space between submedian grooves 3.5.

#### Etymology

Named for the shiny appearance, from the Latin *splendens* = shiny.

#### Distribution

Sweden.

#### Host

Unknown.

#### Material examined

Holotype ♀ ”SWEDEN, Skåne, Lund V., RN 1334/6176, 24.vi.1983, C. Hansson” (MZLU).

### Tetrastichus sti sp. n.

B2833E77-47C2-5C53-B70E-ACA765A4C2EA

urn:lsid:zoobank.org:act:B7076D49-5606-405C-A4E4-8DC8EE3920DE

#### Materials

- **Type status:** Holotype. **Occurrence:** occurrenceDetails: http://www.boldsystems.org/index.php/API_Public/specimen?ids=BC-ZSM-HYM-13565-E11; catalogNumber: BC-ZSM-HYM-13565-E11; recordNumber: BC-ZSM-HYM-13565-E11; recordedBy: SMTP project; individualID: BC-ZSM-HYM-13565-E11; individualCount: 1; sex: female; lifeStage: adult; **Taxon:** scientificName: Tetrastichus sti; phylum: Arthropoda; class: Insecta; order: Hymenoptera; family: Eulophidae; genus: Tetrastichinae; taxonRemarks: Holotype deposited in SMTP; **Location:** country: Sweden; decimalLatitude: 58.3; decimalLongitude: 14.617; **Record Level:** type: PhysicalObject; language: en; institutionCode: SMTP; basisOfRecord: PreservedSpecimen
- **Type status:** Paratype. **Occurrence:** occurrenceDetails: http://www.boldsystems.org/index.php/API_Public/specimen?ids=BC-ZSM-HYM-22523-F12; catalogNumber: BC-ZSM-HYM-22523-F12; recordNumber: BC-ZSM-HYM-22523-F12; recordedBy: O.Popovici; individualID: BC-ZSM-HYM-22523-F12; individualCount: 1; sex: female; lifeStage: adult; **Taxon:** scientificName: Tetrastichus sti; phylum: Arthropoda; class: Insecta; order: Hymenoptera; family: Eulophidae; genus: Tetrastichinae; **Location:** country: Romania; decimalLatitude: 46.594; decimalLongitude: 27.353; **Record Level:** type: PhysicalObject; language: en; basisOfRecord: PreservedSpecimen
- **Type status:** Paratype. **Occurrence:** occurrenceDetails: http://www.boldsystems.org/index.php/API_Public/specimen?ids=BC-ZSM-HYM-21587-G08; catalogNumber: BC-ZSM-HYM-21587-G08; recordNumber: BC-ZSM-HYM-21587-G08; recordedBy: C.Hansson; individualID: BC-ZSM-HYM-21587-G08; individualCount: 1; sex: female; lifeStage: adult; **Taxon:** scientificName: Tetrastichus sti; phylum: Arthropoda; class: Insecta; order: Hymenoptera; family: Eulophidae; genus: Tetrastichinae; **Location:** country: Sweden; decimalLatitude: 55.834; decimalLongitude: 13.528; **Record Level:** type: PhysicalObject; language: en; basisOfRecord: PreservedSpecimen
- **Type status:** Paratype. **Occurrence:** occurrenceDetails: http://www.boldsystems.org/index.php/API_Public/specimen?ids=BC-ZSM-HYM-13565-B10; catalogNumber: BC-ZSM-HYM-13565-B10; recordNumber: BC-ZSM-HYM-13565-B10; recordedBy: SMTP project; individualID: BC-ZSM-HYM-13565-B10; individualCount: 1; sex: female; lifeStage: adult; **Taxon:** scientificName: Tetrastichus sti; phylum: Arthropoda; class: Insecta; order: Hymenoptera; family: Eulophidae; genus: Tetrastichinae; **Location:** country: Sweden; decimalLatitude: 58.3; decimalLongitude: 14.617; **Record Level:** type: PhysicalObject; language: en; basisOfRecord: PreservedSpecimen
- **Type status:** Paratype. **Occurrence:** occurrenceDetails: http://www.boldsystems.org/index.php/API_Public/specimen?ids=BC-ZSM-HYM-22523-A01; catalogNumber: BC-ZSM-HYM-22523-A01; recordNumber: BC-ZSM-HYM-22523-A01; recordedBy: Swedish Malaise Trap Project; individualID: BC-ZSM-HYM-22523-A01; individualCount: 1; sex: female; lifeStage: adult; **Taxon:** scientificName: Tetrastichus sti; phylum: Arthropoda; class: Insecta; order: Hymenoptera; family: Eulophidae; genus: Tetrastichinae; **Location:** country: Sweden; decimalLatitude: 58.185; decimalLongitude: 14.379; **Record Level:** type: PhysicalObject; language: en; basisOfRecord: PreservedSpecimen
- **Type status:** Paratype. **Occurrence:** occurrenceDetails: http://www.boldsystems.org/index.php/API_Public/specimen?ids=BC-ZSM-HYM-13565-E12; catalogNumber: BC-ZSM-HYM-13565-E12; recordNumber: BC-ZSM-HYM-13565-E12; recordedBy: SMTP Project; individualID: BC-ZSM-HYM-13565-E12; individualCount: 1; sex: female; lifeStage: adult; **Taxon:** scientificName: Tetrastichus sti; phylum: Arthropoda; class: Insecta; order: Hymenoptera; family: Eulophidae; genus: Tetrastichinae; **Location:** country: Sweden; decimalLatitude: 58.3; decimalLongitude: 14.617; **Record Level:** type: PhysicalObject; language: en; institutionCode: SMTP; basisOfRecord: PreservedSpecimen

#### Description

FEMALE holotype (Fig. 36). Body length 2.1 mm (paratypes 1.5–2.0 mm). *Head*. Width/length in dorsal view 2.2, width/length in frontal view 1.3, POL/OOL 2.0, widths head/mesosoma 1.0, mouth width/malar space 0.9, malar space/eye height 0.8. *Antenna*. Scape length/eye height 1.1, pedicel+flagellum length/mesosoma width 1.3, length/width F1, F2, F3 2.4, 2.3, 2.2, clava length/width 4.4, lengths pedicel/F1 0.6, lengths F1/F2 1.0, F1/F3 1.1, lengths F1, F2, F3/clava 0.6, 0.5, 0.5, widths F1/pedicel (dorsal view) 1.0, lengths antennal spicule/C3 0.3. *Mesosoma*. Length/width 1.5, mesoscutal midlobe length/width 0.9 (width measured in anterior part), mid-lobe with median groove in posterior ¾, with five adnotaular setae on each side, lengths mesoscutum/mesoscutellum (measured medially) 1.3, mesoscutellum length/width 0.9, length/width of enclosed space between submedian grooves 2.6, distance between SMG/distance between SMG and SLG 1.5, lengths dorsellum/propodeum (measured medially) 0.5, propodeum with strong reticulation, propodeal callus with four setae. *Fore wing*. Costal cell length/width 10.0, lengths costal cell/marginal vein 0.9, lengths marginal/stigmal veins 3.3. *Gaster*. Subcircular to ovate, length/width 1.3, lengths gaster/mesosoma 1.1, Gt_7_ length/width 0.4, length of longest cercal seta/next longest seta 1.3, longest cercal seta curved in apical ⅓, ovipositor sheaths not reaching apex of Gt_7_.

Colour. Body golden-green, entire antenna dark brown, tegulae black with metallic tinge, wings hyaline with veins brown to fuscous, coxae and femora concolorous with body, trochanters dark brown, tibiae yellowish-brown, fore tarsus brown, mid and hind tarsi with T1-2 yellowish-white and T3-4 brown.

Variation. Paratypes with body metallic blue, green or blue-green.

MALE. Body length 1.7 mm. *Head*. Width/length in dorsal view 2.3, width/length in frontal view 1.3, eye height/malar space 1.2, mouth width/malar space 1.0, widths head/mesosoma 1.1. *Antenna*. F1–F4 without basal whorls of setae, scape length/eye height 1.2, scape length/width 3.0, ventral plaque placed in central part of scape, lengths ventral plaque/scape 0.7, length/width F1, F2, F3, F4 1.6, 2.4, 2.3, 2.5, lengths pedicel/F1 0.8, lengths F1/F2 0.7, F1/F3 0.8, F1/F4 0.8.

Colour. As in female. Body metallic blue-green.

#### Diagnosis

Mid and hind tibiae yellowish-brown; mesoscutellum with distance between submedian grooves 1.5× distance between submedian and sublateral grooves; female with malar space 0.8× height of eye, antenna with F2 2.3×, F3 2.2× and clava 4.4× as long as wide, clava with a strong constriction between C1 and C2; male antenna with scape 3.0× as long as wide and 1.0× as long as height of eye, length/width F1, F2, F3, F4 1.6, 2.4, 2.3, 2.5.

#### Etymology

Named after acronym STI = Swedish Taxonomy Initiative, the major funding source for this project.

#### Distribution

Romania, Sweden and United Kingdom.

#### Notes

Holotype deposited in SMTP, paratypes in MZLU, SMTP and NHM.

#### Host

Unknown.

#### Additional paratype (not barcoded)

1♀ ”ENGLAND: Lincolnshire, Woodhall Spa, (2), 27.vii.1968, M.W.R.de V. Graham”. Paratypes in MZLU, NHM, SMTP.

### Tetrastichus suecus sp. n.

4770D9BA-C513-58B0-A8F6-AB26ECFA7F1D

urn:lsid:zoobank.org:act:8B6EE64C-2585-46D0-A4A4-7C74047310A5

#### Materials

- **Type status:** Holotype. **Occurrence:** occurrenceDetails: http://www.boldsystems.org/index.php/API_Public/specimen?ids=BC-ZSM-HYM-29751-F01; catalogNumber: BC-ZSM-HYM-29751-F01; recordNumber: BC-ZSM-HYM-29751-F01; recordedBy: C. Hansson; individualID: BC-ZSM-HYM-29751-F01; individualCount: 1; sex: female; lifeStage: adult; **Taxon:** scientificName: Tetrastichus suecus; phylum: Arthropoda; class: Insecta; order: Hymenoptera; family: Eulophidae; genus: Tetrastichinae; taxonRemarks: Holotype deposited in MZLU; **Location:** country: Sweden; decimalLatitude: 55.0756; decimalLongitude: 13.6778; **Record Level:** type: PhysicalObject; language: en; institutionCode: MZLU; basisOfRecord: PreservedSpecimen
- **Type status:** Paratype. **Occurrence:** occurrenceDetails: http://www.boldsystems.org/index.php/API_Public/specimen?ids=BC-ZSM-HYM-27769-F03; catalogNumber: BC-ZSM-HYM-27769-F03; recordNumber: BC-ZSM-HYM-27769-F03; recordedBy: C. Hansson; individualID: BC-ZSM-HYM-27769-F03; individualCount: 1; sex: female; lifeStage: adult; **Taxon:** scientificName: Tetrastichus suecus; phylum: Arthropoda; class: Insecta; order: Hymenoptera; family: Eulophidae; genus: Tetrastichinae; **Location:** country: Sweden; decimalLatitude: 56.676; decimalLongitude: 16.558; **Record Level:** type: PhysicalObject; language: en; basisOfRecord: PreservedSpecimen
- **Type status:** Paratype. **Occurrence:** occurrenceDetails: http://www.boldsystems.org/index.php/API_Public/specimen?ids=BC-ZSM-HYM-27769-E12; catalogNumber: BC-ZSM-HYM-27769-E12; recordNumber: BC-ZSM-HYM-27769-E12; recordedBy: C. Hansson; individualID: BC-ZSM-HYM-27769-E12; individualCount: 1; sex: female; lifeStage: adult; **Taxon:** scientificName: Tetrastichus suecus; phylum: Arthropoda; class: Insecta; order: Hymenoptera; family: Eulophidae; genus: Tetrastichinae; **Location:** country: Sweden; decimalLatitude: 56.676; decimalLongitude: 16.558; **Record Level:** type: PhysicalObject; language: en; basisOfRecord: PreservedSpecimen
- **Type status:** Paratype. **Occurrence:** occurrenceDetails: http://www.boldsystems.org/index.php/API_Public/specimen?ids=BC-ZSM-HYM-27769-E09; catalogNumber: BC-ZSM-HYM-27769-E09; recordNumber: BC-ZSM-HYM-27769-E09; recordedBy: C. Hansson; individualID: BC-ZSM-HYM-27769-E09; individualCount: 1; sex: female; lifeStage: adult; **Taxon:** scientificName: Tetrastichus suecus; phylum: Arthropoda; class: Insecta; order: Hymenoptera; family: Eulophidae; genus: Tetrastichinae; **Location:** country: Sweden; decimalLatitude: 56.676; decimalLongitude: 16.558; **Record Level:** type: PhysicalObject; language: en; basisOfRecord: PreservedSpecimen
- **Type status:** Paratype. **Occurrence:** occurrenceDetails: http://www.boldsystems.org/index.php/API_Public/specimen?ids=BC-ZSM-HYM-20721-B07; catalogNumber: BC-ZSM-HYM-20721-B07; recordNumber: BC-ZSM-HYM-20721-B07; recordedBy: C. Hansson; individualID: BC-ZSM-HYM-20721-B07; individualCount: 1; sex: female; lifeStage: adult; **Taxon:** scientificName: Tetrastichus suecus; phylum: Arthropoda; class: Insecta; order: Hymenoptera; family: Eulophidae; genus: Tetrastichinae; **Location:** country: Sweden; decimalLatitude: 56.617; decimalLongitude: 16.567; **Record Level:** type: PhysicalObject; language: en; basisOfRecord: PreservedSpecimen
- **Type status:** Paratype. **Occurrence:** occurrenceDetails: http://www.boldsystems.org/index.php/API_Public/specimen?ids=BC-ZSM-HYM-27769-E04; catalogNumber: BC-ZSM-HYM-27769-E04; recordNumber: BC-ZSM-HYM-27769-E04; recordedBy: C. Hansson; individualID: BC-ZSM-HYM-27769-E04; individualCount: 1; sex: female; lifeStage: adult; **Taxon:** scientificName: Tetrastichus suecus; phylum: Arthropoda; class: Insecta; order: Hymenoptera; family: Eulophidae; genus: Tetrastichinae; **Location:** country: Sweden; decimalLatitude: 56.609; decimalLongitude: 16.509; **Record Level:** type: PhysicalObject; language: en; basisOfRecord: PreservedSpecimen
- **Type status:** Paratype. **Occurrence:** occurrenceDetails: http://www.boldsystems.org/index.php/API_Public/specimen?ids=BC-ZSM-HYM-21587-B01; catalogNumber: BC-ZSM-HYM-21587-B01; recordNumber: BC-ZSM-HYM-21587-B01; recordedBy: C.Hansson; individualID: BC-ZSM-HYM-21587-B01; individualCount: 1; sex: female; lifeStage: adult; **Taxon:** scientificName: Tetrastichus suecus; phylum: Arthropoda; class: Insecta; order: Hymenoptera; family: Eulophidae; genus: Tetrastichinae; **Location:** country: Sweden; decimalLatitude: 56.893; decimalLongitude: 16.768; **Record Level:** type: PhysicalObject; language: en; basisOfRecord: PreservedSpecimen
- **Type status:** Paratype. **Occurrence:** occurrenceDetails: http://www.boldsystems.org/index.php/API_Public/specimen?ids=BC-ZSM-HYM-21587-A08; catalogNumber: BC-ZSM-HYM-21587-A08; recordNumber: BC-ZSM-HYM-21587-A08; recordedBy: C.Hansson; individualID: BC-ZSM-HYM-21587-A08; individualCount: 1; sex: female; lifeStage: adult; **Taxon:** scientificName: Tetrastichus suecus; phylum: Arthropoda; class: Insecta; order: Hymenoptera; family: Eulophidae; genus: Tetrastichinae; **Location:** country: Sweden; decimalLatitude: 56.765; decimalLongitude: 16.695; **Record Level:** type: PhysicalObject; language: en; basisOfRecord: PreservedSpecimen
- **Type status:** Paratype. **Occurrence:** occurrenceDetails: http://www.boldsystems.org/index.php/API_Public/specimen?ids=BC-ZSM-HYM-21587-A05; catalogNumber: BC-ZSM-HYM-21587-A05; recordNumber: BC-ZSM-HYM-21587-A05; recordedBy: C.Hansson; individualID: BC-ZSM-HYM-21587-A05; individualCount: 1; sex: female; lifeStage: adult; **Taxon:** scientificName: Tetrastichus suecus; phylum: Arthropoda; class: Insecta; order: Hymenoptera; family: Eulophidae; genus: Tetrastichinae; **Location:** country: Sweden; decimalLatitude: 57.141; decimalLongitude: 17.024; **Record Level:** type: PhysicalObject; language: en; basisOfRecord: PreservedSpecimen
- **Type status:** Paratype. **Occurrence:** occurrenceDetails: http://www.boldsystems.org/index.php/API_Public/specimen?ids=BC-ZSM-HYM-20721-C10; catalogNumber: BC-ZSM-HYM-20721-C10; recordNumber: BC-ZSM-HYM-20721-C10; recordedBy: C. Hansson; individualID: BC-ZSM-HYM-20721-C10; individualCount: 1; sex: female; lifeStage: adult; **Taxon:** scientificName: Tetrastichus suecus; phylum: Arthropoda; class: Insecta; order: Hymenoptera; family: Eulophidae; genus: Tetrastichinae; **Location:** country: Sweden; decimalLatitude: 55.7; decimalLongitude: 13.167; **Record Level:** type: PhysicalObject; language: en; basisOfRecord: PreservedSpecimen
- **Type status:** Paratype. **Occurrence:** occurrenceDetails: http://www.boldsystems.org/index.php/API_Public/specimen?ids=BC-ZSM-HYM-20721-H11; catalogNumber: BC-ZSM-HYM-20721-H11; recordNumber: BC-ZSM-HYM-20721-H11; recordedBy: C. Hansson; individualID: BC-ZSM-HYM-20721-H11; individualCount: 1; sex: male; lifeStage: adult; **Taxon:** scientificName: Tetrastichus suecus; phylum: Arthropoda; class: Insecta; order: Hymenoptera; family: Eulophidae; genus: Tetrastichinae; **Location:** country: Sweden; decimalLatitude: 55.7; decimalLongitude: 13.45; **Record Level:** type: PhysicalObject; language: en; basisOfRecord: PreservedSpecimen
- **Type status:** Paratype. **Occurrence:** occurrenceDetails: http://www.boldsystems.org/index.php/API_Public/specimen?ids=BC-ZSM-HYM-29813-D07; catalogNumber: BC-ZSM-HYM-29813-D07; recordNumber: BC-ZSM-HYM-29813-D07; recordedBy: B.Tjeder; individualID: BC-ZSM-HYM-29813-D07; individualCount: 1; sex: female; lifeStage: adult; **Taxon:** scientificName: Tetrastichus suecus; phylum: Arthropoda; class: Insecta; order: Hymenoptera; family: Eulophidae; genus: Tetrastichinae; **Location:** country: Sweden; decimalLatitude: 55.3834; decimalLongitude: 14.1197; **Record Level:** type: PhysicalObject; language: en; basisOfRecord: PreservedSpecimen
- **Type status:** Paratype. **Occurrence:** occurrenceDetails: http://www.boldsystems.org/index.php/API_Public/specimen?ids=BC-ZSM-HYM-27769-C01; catalogNumber: BC-ZSM-HYM-27769-C01; recordNumber: BC-ZSM-HYM-27769-C01; recordedBy: C. Hansson; individualID: BC-ZSM-HYM-27769-C01; individualCount: 1; sex: female; lifeStage: adult; **Taxon:** scientificName: Tetrastichus suecus; phylum: Arthropoda; class: Insecta; order: Hymenoptera; family: Eulophidae; genus: Tetrastichinae; **Location:** country: Sweden; decimalLatitude: 56.676; decimalLongitude: 16.558; **Record Level:** type: PhysicalObject; language: en; basisOfRecord: PreservedSpecimen
- **Type status:** Paratype. **Occurrence:** occurrenceDetails: http://www.boldsystems.org/index.php/API_Public/specimen?ids=BC-ZSM-HYM-27769-B10; catalogNumber: BC-ZSM-HYM-27769-B10; recordNumber: BC-ZSM-HYM-27769-B10; recordedBy: C. Hansson; individualID: BC-ZSM-HYM-27769-B10; individualCount: 1; sex: female; lifeStage: adult; **Taxon:** scientificName: Tetrastichus suecus; phylum: Arthropoda; class: Insecta; order: Hymenoptera; family: Eulophidae; genus: Tetrastichinae; **Location:** country: Sweden; decimalLatitude: 56.676; decimalLongitude: 16.558; **Record Level:** type: PhysicalObject; language: en; basisOfRecord: PreservedSpecimen
- **Type status:** Paratype. **Occurrence:** occurrenceDetails: http://www.boldsystems.org/index.php/API_Public/specimen?ids=BC-ZSM-HYM-13565-A01; catalogNumber: BC-ZSM-HYM-13565-A01; recordNumber: BC-ZSM-HYM-13565-A01; individualID: BC-ZSM-HYM-13565-A01; individualCount: 1; sex: female; lifeStage: adult; **Taxon:** scientificName: Tetrastichus suecus; phylum: Arthropoda; class: Insecta; order: Hymenoptera; family: Eulophidae; genus: Tetrastichinae; **Location:** country: Sweden; decimalLatitude: 66.767; decimalLongitude: 20.10; **Record Level:** type: PhysicalObject; language: en; basisOfRecord: PreservedSpecimen
- **Type status:** Paratype. **Occurrence:** occurrenceDetails: http://www.boldsystems.org/index.php/API_Public/specimen?ids=BC-ZSM-HYM-25461-E04; catalogNumber: BC-ZSM-HYM-25461-E04; recordNumber: BC-ZSM-HYM-25461-E04; recordedBy: D.M.S.P., J.F.P.; individualID: BC-ZSM-HYM-25461-E04; individualCount: 1; lifeStage: adult; **Taxon:** scientificName: Tetrastichus suecus; phylum: Arthropoda; class: Insecta; order: Hymenoptera; family: Eulophidae; genus: Tetrastichinae; **Location:** country: Sweden; decimalLatitude: 55.433; decimalLongitude: 14.117; **Record Level:** type: PhysicalObject; language: en; basisOfRecord: PreservedSpecimen
- **Type status:** Paratype. **Occurrence:** occurrenceDetails: http://www.boldsystems.org/index.php/API_Public/specimen?ids=BC-ZSM-HYM-25461-E05; catalogNumber: BC-ZSM-HYM-25461-E05; recordNumber: BC-ZSM-HYM-25461-E05; recordedBy: D.M.S.P., J.F.P.; individualID: BC-ZSM-HYM-25461-E05; individualCount: 1; lifeStage: adult; **Taxon:** scientificName: Tetrastichus suecus; phylum: Arthropoda; class: Insecta; order: Hymenoptera; family: Eulophidae; genus: Tetrastichinae; **Location:** country: Sweden; decimalLatitude: 55.433; decimalLongitude: 14.117; **Record Level:** type: PhysicalObject; language: en; basisOfRecord: PreservedSpecimen
- **Type status:** Paratype. **Occurrence:** occurrenceDetails: http://www.boldsystems.org/index.php/API_Public/specimen?ids=BC-ZSM-HYM-25461-E06; catalogNumber: BC-ZSM-HYM-25461-E06; recordNumber: BC-ZSM-HYM-25461-E06; recordedBy: D.M.S.P., J.F.P.; individualID: BC-ZSM-HYM-25461-E06; individualCount: 1; lifeStage: adult; **Taxon:** scientificName: Tetrastichus suecus; phylum: Arthropoda; class: Insecta; order: Hymenoptera; family: Eulophidae; genus: Tetrastichinae; **Location:** country: Sweden; decimalLatitude: 55.433; decimalLongitude: 14.117; **Record Level:** type: PhysicalObject; language: en; basisOfRecord: PreservedSpecimen
- **Type status:** Paratype. **Occurrence:** occurrenceDetails: http://www.boldsystems.org/index.php/API_Public/specimen?ids=BC-ZSM-HYM-29751-G07; catalogNumber: BC-ZSM-HYM-29751-G07; recordNumber: BC-ZSM-HYM-29751-G07; recordedBy: C. Hansson; individualID: BC-ZSM-HYM-29751-G07; individualCount: 1; sex: female; lifeStage: adult; **Taxon:** scientificName: Tetrastichus suecus; phylum: Arthropoda; class: Insecta; order: Hymenoptera; family: Eulophidae; genus: Tetrastichinae; **Location:** country: Sweden; decimalLatitude: 55.0756; decimalLongitude: 13.6778; **Record Level:** type: PhysicalObject; language: en; basisOfRecord: PreservedSpecimen
- **Type status:** Paratype. **Occurrence:** occurrenceDetails: http://www.boldsystems.org/index.php/API_Public/specimen?ids=BC-ZSM-HYM-29813-G09; catalogNumber: BC-ZSM-HYM-29813-G09; recordNumber: BC-ZSM-HYM-29813-G09; recordedBy: A.Jansson; individualID: BC-ZSM-HYM-29813-G09; individualCount: 1; sex: F; lifeStage: Adult; associatedMedia: http://www.boldsystems.org/pics/BCHYM/BC-ZSM-HYM-29813-G09+1516986306.jpg; **Taxon:** scientificName: Tetrastichus suecus; phylum: Arthropoda; class: Insecta; order: Hymenoptera; family: Eulophidae; genus: Tetrastichus; **Location:** country: Sweden; locality: Oerebro; decimalLatitude: 59.2753; decimalLongitude: 15.2134; **Identification:** identifiedBy: Christer Hansson

#### Description

FEMALE holotype (Fig. 37). Body length 2.2 mm (paratypes 1.3–2.6 mm). *Head*. Width/length in dorsal view 2.2, width/length in frontal view 1.3, POL/OOL 1.6, widths head/mesosoma 1.0, mouth width/malar space 1.0, malar space/eye height 1.0. *Antenna*. Scape length/eye height 1.1, pedicel+flagellum length/mesosoma width 1.2, length/width F1, F2, F3 2.4, 2.3, 2.3, clava length/width 4.2, lengths pedicel/F1 0.6, lengths F1/F2 1.0, F1/F3 1.0, lengths F1, F2, F3/clava 0.6, 0.6, 0.6, widths F1/pedicel (dorsal view) 1.1, lengths antennal spicule/C3 0.3. *Mesosoma*. Length/width 1.5, mesoscutal mid-lobe length/width 0.9 (width measured in anterior part), mid-lobe with complete median groove, with six adnotaular setae on each side, lengths mesoscutum/mesoscutellum (measured medially) 1.3, mesoscutellum length/width 0.8, length/width of enclosed space between submedian grooves 2.7, distance between SMG/distance between SMG and SLG 1.4, lengths dorsellum/propodeum 0.5, propodeum with weak reticulation, propodeal callus with four setae. *Fore wing*. Costal cell length/width 8.0, lengths costal cell/marginal vein 1.2, lengths marginal/stigmal veins 2.6. *Gaster*. Subcircular to ovate, length/width 1.6, lengths gaster/mesosoma 1.1, Gt_7_ length/width 0.5, length of longest cercal seta/next longest seta 2.0, longest cercal seta evenly curved, ovipositor sheaths not reaching apex of Gt_7_.

Colour. Body golden-green, scape yellowish-brown with dorsal edge and apical ⅓ dark brown, pedicel and flagellum dark brown, tegulae black with metallic tinges, wing venation yellowish-white, coxae and femora concolorous with body, trochanters black, tibiae yellowish-brown, fore tarsus brownish becoming darker towards apex, mid and hind tarsi yellowish-brown with T4 brown.

Variation. Three paratypes with entire antennal scape dark brown. Colour of body blue, blue-green or green-blue and small specimens with weaker metallic tinges on body.

MALE (Fig. 37). Body length 1.9 mm. *Head*. Width/length in dorsal view 2.4, width/length in frontal view 1.3, eye height/malar space 1.1, mouth width/malar space 1.1, widths head/mesosoma 1.1. *Antenna*. F1–F4 without basal whorls of setae, scape length/eye height 1.1, scape length/width 3.0, ventral plaque placed in central part of scape, lengths ventral plaque/scape 0.7, pedicel+flagellum length/mesosoma width 1.6, length/width F1, F2, F3, F4 1.6, 2.0, 2.2, 2.2, clava length/width 4.4, lengths pedicel/F1 0.8, lengths F1/F2 0.8, F1/F3 0.7, F1/F4 0.7, lengths F1, F2, F3, F4/clava 0.4, 0.5, 0.5, 0.5.

Colour. Scape dark brown and body metallic blue-green. Otherwise similar to female.

#### Diagnosis

Tibiae yellowish-brown; female with malar space 1.0× eye height, POL/OOL 1.6, F1 2.4×, F2 2.3×, F3 2.3× as long as wide; male with antennal scape 3.0×, F1 1.6×, F2 2.0×, F3 2.2×, F4 2.2× and clava 4.4× as long as wide.

#### Distribution

Sweden.

#### Notes

Holotype deposited in MZLU, paratypes in MZLU, NHM, SMTP and ZSM.

#### Host

Unknown.

### Tetrastichus clito group

160CAF69-4842-5F87-B8E1-04CDF789ACF4

#### Diagnosis

Frons with a more or less distinct median longitudinal carina extending from between the toruli to near the median ocellus, the sutures which define the scrobal area laterally tend to diverge ventrally, away from the median carina (Fig. 3b); hind coxae usually finely reticulate on the externo-dorsal surface (Fig. 3d).

### Tetrastichus argutus sp. n.

13FF9F87-6304-55A3-B5BE-1DBA5384B7EE

urn:lsid:zoobank.org:act:9CD75EDB-59FC-4BF2-96EC-1632D8465ABA

#### Materials

- **Type status:** Holotype. **Occurrence:** occurrenceDetails: http://www.boldsystems.org/index.php/API_Public/specimen?ids=BC-ZSM-HYM-29751-E11; catalogNumber: BC-ZSM-HYM-29751-E11; recordNumber: BC-ZSM-HYM-29751-E11; recordedBy: C. Hansson; individualID: BC-ZSM-HYM-29751-E11; individualCount: 1; sex: female; lifeStage: adult; **Taxon:** scientificName: Tetrastichus argutus; phylum: Arthropoda; class: Insecta; order: Hymenoptera; family: Eulophidae; genus: Tetrastichinae; taxonRemarks: Holotype deposited in MZLU; **Location:** country: Sweden; decimalLatitude: 55.7078; decimalLongitude: 13.4528; **Record Level:** type: PhysicalObject; language: en; institutionCode: MZLU; basisOfRecord: PreservedSpecimen
- **Type status:** Paratype. **Occurrence:** occurrenceDetails: http://www.boldsystems.org/index.php/API_Public/specimen?ids=BC-ZSM-HYM-29750-E01; catalogNumber: BC-ZSM-HYM-29750-E01; recordNumber: BC-ZSM-HYM-29750-E01; recordedBy: C. Hansson; individualID: BC-ZSM-HYM-29750-E01; individualCount: 1; sex: female; lifeStage: adult; **Taxon:** scientificName: Tetrastichus argutus; phylum: Arthropoda; class: Insecta; order: Hymenoptera; family: Eulophidae; genus: Tetrastichinae; **Location:** country: Sweden; decimalLatitude: 55.6619; decimalLongitude: 13.5472; **Record Level:** type: PhysicalObject; language: en; basisOfRecord: PreservedSpecimen
- **Type status:** Paratype. **Occurrence:** occurrenceDetails: http://www.boldsystems.org/index.php/API_Public/specimen?ids=BC-ZSM-HYM-25460-H03; catalogNumber: BC-ZSM-HYM-25460-H03; recordNumber: BC-ZSM-HYM-25460-H03; recordedBy: C. Hansson; individualID: BC-ZSM-HYM-25460-H03; individualCount: 1; sex: male; lifeStage: adult; **Taxon:** scientificName: Tetrastichus argutus; phylum: Arthropoda; class: Insecta; order: Hymenoptera; family: Eulophidae; genus: Tetrastichinae; **Location:** country: Sweden; decimalLatitude: 55.6619; decimalLongitude: 13.5472; **Record Level:** type: PhysicalObject; language: en; basisOfRecord: PreservedSpecimen
- **Type status:** Paratype. **Occurrence:** occurrenceDetails: http://www.boldsystems.org/index.php/API_Public/specimen?ids=BC-ZSM-HYM-29751-D04; catalogNumber: BC-ZSM-HYM-29751-D04; recordNumber: BC-ZSM-HYM-29751-D04; recordedBy: C. Hansson; individualID: BC-ZSM-HYM-29751-D04; individualCount: 1; sex: male; lifeStage: adult; **Taxon:** scientificName: Tetrastichus argutus; phylum: Arthropoda; class: Insecta; order: Hymenoptera; family: Eulophidae; genus: Tetrastichinae; **Location:** country: Sweden; decimalLatitude: 55.6967; decimalLongitude: 13.47; **Record Level:** type: PhysicalObject; language: en; basisOfRecord: PreservedSpecimen
- **Type status:** Paratype. **Occurrence:** occurrenceDetails: http://www.boldsystems.org/index.php/API_Public/specimen?ids=BC-ZSM-HYM-22524-C06; catalogNumber: BC-ZSM-HYM-22524-C06; recordNumber: BC-ZSM-HYM-22524-C06; recordedBy: C. Hansson; individualID: BC-ZSM-HYM-22524-C06; individualCount: 1; sex: male; lifeStage: adult; **Taxon:** scientificName: Tetrastichus argutus; phylum: Arthropoda; class: Insecta; order: Hymenoptera; family: Eulophidae; genus: Tetrastichinae; **Location:** country: Hungary; decimalLatitude: 46.522; decimalLongitude: 16.256; **Record Level:** type: PhysicalObject; language: en; basisOfRecord: PreservedSpecimen
- **Type status:** Paratype. **Occurrence:** occurrenceDetails: http://www.boldsystems.org/index.php/API_Public/specimen?ids=BC-ZSM-HYM-27769-C12; catalogNumber: BC-ZSM-HYM-27769-C12; recordNumber: BC-ZSM-HYM-27769-C12; recordedBy: C. Hansson; individualID: BC-ZSM-HYM-27769-C12; individualCount: 1; sex: male; lifeStage: adult; **Taxon:** scientificName: Tetrastichus argutus; phylum: Arthropoda; class: Insecta; order: Hymenoptera; family: Eulophidae; genus: Tetrastichinae; **Location:** country: Sweden; decimalLatitude: 56.788; decimalLongitude: 16.637; **Record Level:** type: PhysicalObject; language: en; basisOfRecord: PreservedSpecimen
- **Type status:** Paratype. **Occurrence:** occurrenceDetails: http://www.boldsystems.org/index.php/API_Public/specimen?ids=BC-ZSM-HYM-25460-C05; catalogNumber: BC-ZSM-HYM-25460-C05; recordNumber: BC-ZSM-HYM-25460-C05; recordedBy: C. Hansson; individualID: BC-ZSM-HYM-25460-C05; individualCount: 1; sex: male; lifeStage: adult; **Taxon:** scientificName: Tetrastichus argutus; phylum: Arthropoda; class: Insecta; order: Hymenoptera; family: Eulophidae; genus: Tetrastichinae; **Location:** country: Sweden; decimalLatitude: 55.705; decimalLongitude: 13.4886; **Record Level:** type: PhysicalObject; language: en; basisOfRecord: PreservedSpecimen
- **Type status:** Paratype. **Occurrence:** occurrenceDetails: http://www.boldsystems.org/index.php/API_Public/specimen?ids=BC-ZSM-HYM-27769-E10; catalogNumber: BC-ZSM-HYM-27769-E10; recordNumber: BC-ZSM-HYM-27769-E10; recordedBy: C. Hansson; individualID: BC-ZSM-HYM-27769-E10; individualCount: 1; sex: female; lifeStage: adult; **Taxon:** scientificName: Tetrastichus argutus; phylum: Arthropoda; class: Insecta; order: Hymenoptera; family: Eulophidae; genus: Tetrastichinae; **Location:** decimalLatitude: 56.676; decimalLongitude: 16.558; **Record Level:** type: PhysicalObject; language: en; basisOfRecord: PreservedSpecimen

#### Description

FEMALE holotype (Fig. 38). Body length 2.0 mm (paratype 1.8 mm). *Head*. Width/length in dorsal view 2.0, width/length in frontal view 1.2, POL/OOL 2.1, widths head/mesosoma 1.1, mouth width/malar space 1.2, malar space/eye height 0.6. *Antenna*. Scape length/eye height 1.0, pedicel+flagellum length/mesosoma width 1.9, length/width F1, F2, F3 2.0, 2.8, 2.6, clava length/width 4.0, lengths pedicel/F1 0.4, lengths F1/F2 0.8, F1/F3 1.0, lengths F1, F2, F3/clava 0.6, 0.7, 0.6, widths F1/pedicel (dorsal view) 1.8, lengths antennal spicule/C3 0.3. *Mesosoma*. Length/width 1.4, mesoscutal mid-lobe length/width (width measured medially) 0.9, mid-lobe with complete median groove, with three adnotaular setae on each side, lengths mesoscutum/mesoscutellum (measured medially) 1.4, mesoscutellum length/width 0.8, length/width of enclosed space between submedian grooves 2.1, distance between SMG/distance between SMG and SLG 1.8, lengths dorsellum/propodeum (measured medially) 0.6, propodeum with strong reticulation, propodeal callus with two setae. *Fore wing*. Costal cell length/width 15.0, lengths costal cell/marginal vein 0.8, lengths marginal/stigmal veins 3.2. *Gaster*. Elongate-acuminate, length/width 2.1, lengths gaster/head+mesosoma 1.0, Gt_7_ length/width 0.6, length of longest cercal seta/next longest seta 1.8, longest cercal seta kinked in apical ¼, ovipositor sheaths reaching beyond apex of Gt_7_.

Colour. Body strongly metallic, coppery and golden, scape yellowish-brown, pedicel and flagellum dark brown, tegulae dark brown, wing hyaline with venation yellowish-brown, coxae concolorous with body, trochanters, femora and tibiae yellowish-brown, tarsi yellowish-brown with T4 brown.

MALE. Body length 1.4–1.6 mm. *Head*. Width/length in dorsal view 2.3, width/length in frontal view 1.2, eye height/malar space 1.5, mouth width/malar space 1.4, widths head/mesosoma 1.1. *Antenna*. F1–F4 with basal whorls of setae, reaching beyond apex of corresponding flagellomere, scape length/eye height 1.1, scape length/width 3.2, ventral plaque placed in central part of scape, lengths ventral plaque/scape 0.3, pedicel+flagellum length/mesosoma width 2.1, length/width F1, F2, F3, F4 1.9, 2.4, 2.8, 2.5, clava length/width 4.8, lengths pedicel/F1 0.7, lengths F1/F2 0.8, F1/F3 0.8, F1/F4 0.9, lengths F1, F2, F3, F4/clava 0.5, 0.6, 0.6, 0.5.

Colour. Different from female in several parts: scape, trochanters and femora dark brown, tibiae pale brown, fore tarsus brown, body less strongly metallic, blue.

#### Diagnosis

Female with ovipositor sheaths extending beyond apex of Gt_7_; antenna with long flagellomeres: F1 2.0×, F2 2.8× and F3 2.6× as long as wide, pedicel 0.4× as long as F1, F1 1.8× as wide as width of pedicel in dorsal view, pedicel+flagellum 1.9× width of mesoscutum; male scape with short plaque, 0.3× length of scape and with apex of scape narrowed, with long flagellomeres: F1 1.9×, F2 2.4×, F3 2.8× and F4 2.5× as long as wide; female trochanters, femora and scape yellowish-brown; hind coxa with a strong and sharp carina along posterior margin.

#### Distribution

Hungary and Sweden.

#### Ecology

##### Host

Unknown.

#### Notes

Holotype deposited in MZLU, paratypes in MZLU.

### Tetrastichus bledius sp. n.

0E4D49DB-3DA5-5BFC-ADB9-AD3E8195D391

urn:lsid:zoobank.org:act:E1A7FCA0-37D7-4E81-BAF9-D9515F7A4327

#### Materials

- **Type status:** Holotype. **Occurrence:** occurrenceDetails: http://www.boldsystems.org/index.php/API_Public/specimen?ids=BC-ZSM-HYM-27768-C01; catalogNumber: BC-ZSM-HYM-27768-C01; recordNumber: BC-ZSM-HYM-27768-C01; recordedBy: J.S. Noyes; individualID: BC-ZSM-HYM-27768-C01; individualCount: 1; sex: female; lifeStage: adult; **Taxon:** scientificName: Tetrastichus bledius; phylum: Arthropoda; class: Insecta; order: Hymenoptera; family: Eulophidae; genus: Tetrastichinae; taxonRemarks: Holotype deposited in NHM; **Location:** country: Romania; decimalLatitude: 47.011; decimalLongitude: 27.603; **Record Level:** type: PhysicalObject; language: en; institutionCode: NHM; basisOfRecord: PreservedSpecimen
- **Type status:** Paratype. **Occurrence:** occurrenceDetails: http://www.boldsystems.org/index.php/API_Public/specimen?ids=BC-ZSM-HYM-22526-F01; catalogNumber: BC-ZSM-HYM-22526-F01; recordNumber: BC-ZSM-HYM-22526-F01; recordedBy: S. Schmidt; individualID: BC-ZSM-HYM-22526-F01; individualCount: 1; sex: female; lifeStage: adult; **Taxon:** scientificName: Tetrastichus bledius; phylum: Arthropoda; class: Insecta; order: Hymenoptera; family: Eulophidae; genus: Tetrastichinae; **Location:** country: Sweden; decimalLatitude: 56.776; decimalLongitude: 16.624; **Record Level:** type: PhysicalObject; language: en; institutionCode: ZSM; basisOfRecord: PreservedSpecimen
- **Type status:** Paratype. **Occurrence:** occurrenceDetails: http://www.boldsystems.org/index.php/API_Public/specimen?ids=BC-ZSM-HYM-29751-B01; catalogNumber: BC-ZSM-HYM-29751-B01; recordNumber: BC-ZSM-HYM-29751-B01; recordedBy: C. Hansson; individualID: BC-ZSM-HYM-29751-B01; individualCount: 1; sex: male; lifeStage: adult; **Taxon:** scientificName: Tetrastichus bledius; phylum: Arthropoda; class: Insecta; order: Hymenoptera; family: Eulophidae; genus: Tetrastichinae; **Location:** country: Sweden; decimalLatitude: 55.6861; decimalLongitude: 13.4611; **Record Level:** type: PhysicalObject; language: en; institutionCode: MZLU; basisOfRecord: PreservedSpecimen
- **Type status:** Paratype. **Occurrence:** occurrenceDetails: http://www.boldsystems.org/index.php/API_Public/specimen?ids=BC-ZSM-HYM-27768-H03; catalogNumber: BC-ZSM-HYM-27768-H03; recordNumber: BC-ZSM-HYM-27768-H03; recordedBy: J.S. Noyes; individualID: BC-ZSM-HYM-27768-H03; individualCount: 1; sex: female; lifeStage: adult; **Taxon:** scientificName: Tetrastichus bledius; phylum: Arthropoda; class: Insecta; order: Hymenoptera; family: Eulophidae; genus: Tetrastichinae; **Location:** country: Romania; decimalLatitude: 47.244; decimalLongitude: 27.483; **Record Level:** type: PhysicalObject; language: en; basisOfRecord: PreservedSpecimen
- **Type status:** Paratype. **Occurrence:** occurrenceDetails: http://www.boldsystems.org/index.php/API_Public/specimen?ids=BC-ZSM-HYM-27770-D03; catalogNumber: BC-ZSM-HYM-27760-D03; recordNumber: BC-ZSM-HYM-27760-D03; recordedBy: J.S. Noyes; individualID: BC-ZSM-HYM-27770-D03; individualCount: 1; sex: female; lifeStage: adult; **Taxon:** scientificName: Tetrastichus bledius; phylum: Arthropoda; class: Insecta; order: Hymenoptera; family: Eulophidae; genus: Tetrastichinae; **Location:** country: Romania; decimalLatitude: 47.1875; decimalLongitude: 27.5489; **Record Level:** type: PhysicalObject; language: en; institutionCode: NHM; basisOfRecord: PreservedSpecimen
- **Type status:** Paratype. **Occurrence:** occurrenceDetails: http://www.boldsystems.org/index.php/API_Public/specimen?ids=BC-ZSM-HYM-26563-D02; catalogNumber: BC-ZSM-HYM-26563-D02; recordNumber: BC-ZSM-HYM-26563-D02; recordedBy: C. Hansson; individualID: BC-ZSM-HYM-26563-D02; individualCount: 1; sex: male; lifeStage: adult; **Taxon:** scientificName: Tetrastichus bledius; phylum: Arthropoda; class: Insecta; order: Hymenoptera; family: Eulophidae; genus: Tetrastichinae; **Location:** country: Sweden; decimalLatitude: 55.5214; decimalLongitude: 13.9369; **Record Level:** type: PhysicalObject; language: en; basisOfRecord: PreservedSpecimen
- **Type status:** Paratype. **Occurrence:** occurrenceDetails: http://www.boldsystems.org/index.php/API_Public/specimen?ids=BC-ZSM-HYM-27768-G08; catalogNumber: BC-ZSM-HYM-27768-G08; recordNumber: BC-ZSM-HYM-27768-G08; recordedBy: J.S. Noyes; individualID: BC-ZSM-HYM-27768-G08; individualCount: 1; sex: female; lifeStage: adult; **Taxon:** scientificName: Tetrastichus bledius; phylum: Arthropoda; class: Insecta; order: Hymenoptera; family: Eulophidae; genus: Tetrastichinae; **Location:** country: Romania; decimalLatitude: 47.244; decimalLongitude: 27.483; **Record Level:** type: PhysicalObject; language: en; basisOfRecord: PreservedSpecimen
- **Type status:** Paratype. **Occurrence:** occurrenceDetails: http://www.boldsystems.org/index.php/API_Public/specimen?ids=BC-ZSM-HYM-27770-C12; catalogNumber: BC-ZSM-HYM-27760-C12; recordNumber: BC-ZSM-HYM-27760-C12; recordedBy: J.S. Noyes; individualID: BC-ZSM-HYM-27770-C12; individualCount: 1; sex: female; lifeStage: adult; **Taxon:** scientificName: Tetrastichus bledius; phylum: Arthropoda; class: Insecta; order: Hymenoptera; family: Eulophidae; genus: Tetrastichinae; **Location:** country: Romania; decimalLatitude: 47.2442; decimalLongitude: 27.4828; **Record Level:** type: PhysicalObject; language: en; basisOfRecord: PreservedSpecimen
- **Type status:** Paratype. **Occurrence:** occurrenceDetails: http://www.boldsystems.org/index.php/API_Public/specimen?ids=BC-ZSM-HYM-27770-C11; catalogNumber: BC-ZSM-HYM-27760-C11; recordNumber: BC-ZSM-HYM-27760-C11; recordedBy: J.S. Noyes; individualID: BC-ZSM-HYM-27770-C11; individualCount: 1; sex: female; lifeStage: adult; **Taxon:** scientificName: Tetrastichus bledius; phylum: Arthropoda; class: Insecta; order: Hymenoptera; family: Eulophidae; genus: Tetrastichinae; **Location:** country: Romania; decimalLatitude: 47.2442; decimalLongitude: 27.4828; **Record Level:** type: PhysicalObject; language: en; basisOfRecord: PreservedSpecimen
- **Type status:** Paratype. **Occurrence:** occurrenceDetails: http://www.boldsystems.org/index.php/API_Public/specimen?ids=BC-ZSM-HYM-27770-B03; catalogNumber: BC-ZSM-HYM-27760-B03; recordNumber: BC-ZSM-HYM-27760-B03; recordedBy: J.S. Noyes; individualID: BC-ZSM-HYM-27770-B03; individualCount: 1; sex: male; lifeStage: adult; **Taxon:** scientificName: Tetrastichus bledius; phylum: Arthropoda; class: Insecta; order: Hymenoptera; family: Eulophidae; genus: Tetrastichinae; **Location:** country: Romania; decimalLatitude: 47.2442; decimalLongitude: 27.4828; **Record Level:** type: PhysicalObject; language: en; basisOfRecord: PreservedSpecimen

#### Description

FEMALE holotype (Fig. 39). Body length 1.7 mm (paratypes 1.5–1.7 mm). *Head*. Width/length in dorsal view 2.4, width/length in frontal view 1.3, POL/OOL 2.3, widths head/mesosoma 1.1, mouth width/malar space 1.2, malar space/eye height 0.8. *Antenna*. Scape length/eye height 1.1, pedicel+flagellum length/mesosoma width 1.4, length/width F1, F2, F3 1.6, 1.6, 1.5, clava length/width 3.8, lengths pedicel/F1 0.9, lengths F1/F2 0.9, F1/F3 0.9, lengths F1, F2, F3/clava 0.4, 0.4, 0.4, widths F1/pedicel (dorsal view) 1.1, lengths antennal spicule/C3 0.4. *Mesosoma*. Length/width 1.4, mesoscutal mid-lobe length/width 1.0 (width measured in anterior part), mid-lobe with a median groove in posterior ½, with three adnotaular setae on each side, lengths mesoscutum/mesoscutellum (measured medially) 1.3, mesoscutellum length/width 0.9, length/width of enclosed space between submedian grooves 2.2, distance between SMG/distance between SMG and SLG 1.9, lengths dorsellum/propodeum 0.8, propodeum with strong reticulation, propodeal callus with two setae. *Fore wing*. Costal cell length/width 14.0, lengths costal cell/marginal vein 1.0, lengths marginal/stigmal veins 3.0. *Gaster*. Ovate, length/width 1.7, lengths gaster/head+mesosoma 1.0, Gt_7_ length/width 0.5, length of longest cercal seta/next longest seta 1.3, longest cercal seta almost straight, ovipositor sheaths not reaching apex of Gt_7_.

Colour. Body with weak metallic blue-green tinges, entire antenna dark brown, tegulae dark brown, wings hyaline with venation brown to fuscous, coxae and femora concolorous with body, trochanters dark brown, fore tibia yellowish-brown, mid and hind tibiae dark brown, fore tarsus brown, mid and hind tarsi yellowish-brown with T4 dark brown.

MALE. Body length 1.4 mm. *Head*. Width/length in dorsal view 2.3, width/length in frontal view 1.3, mouth width/malar space 1.2, eye height/malar space 1.4, widths head/mesosoma 1.1. *Antenna*. F1–F4 with basal whorls of setae, reaching beyond apex of corresponding flagellomere, scape length/eye height 1.0, scape length/width 2.4, ventral plaque placed in central part of scape, lengths ventral plaque/scape 0.9, pedicel+flagellum length/mesosoma width 1.6, length/width F1, F2, F3, F4 1.0, 1.3, 1.8, 1.7, clava length/width 3.8, lengths pedicel/F1 1.2, lengths F1/F2 0.8, F1/F3 0.6, F1/F4 0.6, lengths F1, F2, F3, F4/clava 0.3, 0.4, 0.5, 0.4.

Colour similar to female, but with all tarsi brown.

#### Diagnosis

Ratio POL/OOL 2.3; mesoscutellum with distance between SMG/distance between SMG and SLG 1.9; female gaster ovate, 1.7× as long as wide. See also key to distinguish from similar species.

#### Distribution

Romania and Sweden.

#### Ecology

##### Host

Unknown.

#### Notes

Holotype deposited in NHM, paratypes in MZLU, NHM and ZSM.

### Tetrastichus calcarius sp. n.

991C872C-09AB-5465-B6FC-5D90DAACFB93

urn:lsid:zoobank.org:act:69FF6AD2-C120-4EEC-B6F8-C52E37CEBE4E

#### Materials

- **Type status:** Holotype. **Occurrence:** occurrenceDetails: http://www.boldsystems.org/index.php/API_Public/specimen?ids=BC-ZSM-HYM-22526-G03; catalogNumber: BC-ZSM-HYM-22526-G03; recordNumber: BC-ZSM-HYM-22526-G03; recordedBy: S. Schmidt; individualID: BC-ZSM-HYM-22526-G03; individualCount: 1; sex: female; lifeStage: adult; **Taxon:** scientificName: Tetrastichus calcarius; phylum: Arthropoda; class: Insecta; order: Hymenoptera; family: Eulophidae; genus: Tetrastichinae; taxonRemarks: Holotype deposited in ZSM; **Location:** country: Sweden; decimalLatitude: 56.609; decimalLongitude: 16.508; **Record Level:** type: PhysicalObject; language: en; institutionCode: ZSM; basisOfRecord: PreservedSpecimen
- **Type status:** Other material. **Occurrence:** occurrenceDetails: http://www.boldsystems.org/index.php/API_Public/specimen?ids=BC-ZSM-HYM-20721-B05; catalogNumber: BC-ZSM-HYM-20721-B05; recordNumber: BC-ZSM-HYM-20721-B05; recordedBy: C. Hansson; individualID: BC-ZSM-HYM-20721-B05; individualCount: 1; sex: F; lifeStage: Adult; associatedMedia: http://www.boldsystems.org/pics/BCHYM/BC-ZSM-HYM-20721-B05+1398631454.jpg; **Taxon:** scientificName: Tetrastichus calcarius; phylum: Arthropoda; class: Insecta; order: Hymenoptera; family: Eulophidae; genus: Tetrastichus; **Location:** country: Sweden; locality: Kalkstad; decimalLatitude: 56.617; decimalLongitude: 16.533; **Identification:** identifiedBy: Christer Hansson; identificationRemarks: CH08560

#### Description

FEMALE holotype (Fig. 40). Body length 1.7 mm. *Head*. Width/length in dorsal view 2.3, width/length in frontal view 1.3, POL/OOL 1.5, widths head/mesosoma 1.1, mouth width/malar space 1.0, malar space/eye height 0.7. *Antenna*. Scape length/eye height 0.9, pedicel+flagellum length/mesosoma width 1.4, length/width F1, F2, F3 1.8, 1.6, 1.6, clava length/width 3.8, lengths pedicel/F1 0.8, lengths F1/F2 1.0, F1/F3 1.0, lengths F1, F2, F3/clava 0.4, 0.4, 0.4, widths F1/pedicel (dorsal view) 1.1, lengths antennal spicule/C3 0.2. *Mesosoma*. Length/width 1.4, mesoscutal mid-lobe length/width (width measured in anterior part) 1.0, mid-lobe with a complete median groove, with four adnotaular setae on each side, lengths mesoscutum/mesoscutellum (measured medially) 1.3, mesoscutellum length/width 0.9, length/width of enclosed space between submedian grooves 3.1, distance between SMG/distance between SMG and SLG 1.0, lengths dorsellum/propodeum 0.8, propodeum with strong reticulation, propodeal callus with three setae. *Fore wing*. Costal cell length/width 10.0, lengths costal cell/marginal vein 1.1, lengths marginal/stigmal veins 2.6. *Gaster*. Ovate, length/width 1.6, lengths gaster/head+mesosoma 0.9, Gt_7_ length/width 0.5, length of longest cercal seta/next longest seta 1.8, longest cercal seta almost straight, ovipositor sheaths not reaching apex of Gt_7_.

Colour. Body with weak metallic blue-green tinges, entire antenna dark brown, tegulae dark brown, wings hyaline with veins yellowish-white, coxae and trochanters dark brown, femora concolorous with body, fore tibia yellowish-brown, mid and hind tibiae dark brown, tarsi brownish.

MALE. Unknown.

#### Diagnosis

Body weakly metallic; scape, mid and hind tibiae dark brown.

#### Distribution

Sweden.

#### Ecology

##### Host

Unknown.

#### Notes

Holotype deposited in ZSM. A second specimen of the species from the same locality is severely damaged and, therefore, excluded as a paratype.

### Tetrastichus clisius sp. n.

FFF06FA8-2D59-5372-8243-51171CEB50A7

urn:lsid:zoobank.org:act:9C584AD8-6214-4397-A354-E1DC68BD983B

#### Materials

- **Type status:** Holotype. **Occurrence:** occurrenceDetails: http://www.boldsystems.org/index.php/API_Public/specimen?ids=BC-ZSM-HYM-27768-G07; catalogNumber: BC-ZSM-HYM-27768-G07; recordNumber: BC-ZSM-HYM-27768-G07; recordedBy: J.S. Noyes; individualID: BC-ZSM-HYM-27768-G07; individualCount: 1; sex: female; lifeStage: adult; **Taxon:** scientificName: Tetrastichus clisius; phylum: Arthropoda; class: Insecta; order: Hymenoptera; family: Eulophidae; genus: Tetrastichinae; taxonRemarks: Holotype deposited in NHM; **Location:** country: Romania; decimalLatitude: 47.244; decimalLongitude: 27.483; **Record Level:** type: PhysicalObject; language: en; institutionCode: NHM; basisOfRecord: PreservedSpecimen
- **Type status:** Paratype. **Occurrence:** occurrenceDetails: http://www.boldsystems.org/index.php/API_Public/specimen?ids=BC-ZSM-HYM-27770-C03; catalogNumber: BC-ZSM-HYM-27760-C03; recordNumber: BC-ZSM-HYM-27760-C03; recordedBy: J.S. Noyes; individualID: BC-ZSM-HYM-27770-C03; individualCount: 1; sex: male; lifeStage: adult; **Taxon:** scientificName: Tetrastichus clisius; phylum: Arthropoda; class: Insecta; order: Hymenoptera; family: Eulophidae; genus: Tetrastichinae; **Location:** country: Romania; decimalLatitude: 47.2442; decimalLongitude: 27.4828; **Record Level:** type: PhysicalObject; language: en; institutionCode: NHM; basisOfRecord: PreservedSpecimen

#### Description

FEMALE holotype (Fig. 41). Body length 1.4 mm. *Head*. Width/length in dorsal view 2.3, width/length in frontal view 1.3, POL/OOL 1.6, widths head/mesosoma 1.1, mouth width/malar space 1.1, malar space/eye height 0.8. *Antenna*. Scape length/eye height 1.1, pedicel+flagellum length/mesosoma width 1.4, length/width F1, F2, F3 1.4, 1.6, 1.7, clava length/width 4.2, lengths pedicel/F1 0.9, lengths F1/F2 0.9, F1/F3 0.9, lengths F1, F2, F3/clava 0.3, 0.4, 0.4, widths F1/pedicel (dorsal view) 1.3, lengths antennal spicule/C3 0.3. *Mesosoma*. Length/width 1.5, mesoscutal mid-lobe length/width 1.0 (width measured in anterior part), mid-lobe with median groove in posterior ½, with two adnotaular setae on each side, lengths mesoscutum/mesoscutellum (measured medially) 1.5, mesoscutellum length/width 0.9, length/width of enclosed space between submedian grooves 2.4, distance between SMG/distance between SMG and SLG 1.4, lengths dorsellum/propodeum (measured medially) 0.8, propodeum with strong reticulation, propodeal callus with two setae. *Fore wing*. Costal cell length/width 13.5, lengths costal cell/marginal vein 1.1, lengths marginal/stigmal veins 2.7. *Gaster*. Ovate, length/width 1.4, lengths gaster/head+mesosoma 0.7, length of longest cercal seta/next longest seta 1.8, longest cercal seta almost straight, ovipositor sheaths not reaching apex of Gt_7_.

Colour. Body black without metallic tinges, entire antenna dark brown, tegulae dark brown, wings hyaline with venation brown to fuscous, coxae and femora concolorous with body, trochanters dark brown, fore tibia yellowish-brown, mid and hind tibiae dark brown, tarsi brownish.

MALE. Body length 1.6 mm. *Head*. Width/length in dorsal view 2.3, width/length in frontal view 1.3, mouth width/malar space 1.1, height of eye/malar space 1.1, widths head/mesosoma 1.1. *Antenna*. F1–F4 with basal whorls of setae, reaching beyond apex of corresponding flagellomere, scape length/eye height 1.1, scape length/width 2.1, ventral plaque placed in central part of scape, lengths ventral plaque/scape 0.8, pedicel+flagellum length/mesosoma width 1.5, length/width F1, F2, F3, F4 1.1, 1.4, 1.5, 1.7, clava length/width 4.6, lengths pedicel/F1 1.1, lengths F1/F2 0.8, F1/F3 0.7, F1/F4 0.7, lengths F1, F2, F3, F4/clava 0.3, 0.4, 0.4, 0.4.

Colour similar to female, but body with weak metallic blue tinges.

#### Diagnosis

Female with antennal clava long, 4.2× as long as wide; gaster ovate, 1.4× as long as wide; male with scape 1.1× as long as eye and 2.1× as long as wide. See key for separation from similar species.

#### Distribution

Romania.

#### Ecology

##### Host

Unknown.

#### Notes

Holotype deposited in NHM, paratype in NHM.

### Tetrastichus clito

(Walker, 1840)

BCA19439-915C-5CC3-A61F-20387CCDFD99

Tetrastichus clito *Cirrospilus Clito Walker 1840*^42^*Aprostocetus*Graham 1961*Tetrastichus*Peck 1963
Eulophus cassidae Dufour 1846:20. Syntypes not located. Synonymised by Graham 1991:267.
Entedon Ratzeburg 1852:248. Type material destroyed Graham 1991:267. Synonymised by Graham 1991:267.

#### Description

See Graham (1991).

#### Diagnosis

Female antenna short, at most 1.1× as long as width of mesoscutum; mesoscutellum with distance between submedian grooves 1.5× the distance between submedian and sublateral grooves; female gaster short ovate, 1.5× as long as wide.

#### Distribution

(Former) Czechoslovakia, France, Germany, United Kingdom, Canada (Graham 1991) and Sweden (Hedqvist 2003).

#### Ecology

##### Host

*Cassida murraea* L., *C. rubiginosa* Müller, *C. deflorata* Suffrian (Graham 1991), *C. humeralis* Kraatz (**new record**). All hosts are Coleoptera: Chrysomelidae.

#### Material examined

Type material: lectotype ♂ of C. Clito (NHM, type no. 5.1943). Additional material (28♀ 5♂): (former) Czechoslovakia 1♀ (NHM), France 14♀ 2♂ ex *Cassida humeralis* (GD), 2♀ (GD, NHM), Sweden 5♀ (NHM, SMTP), United Kingdom 6♀ 3♂ (NHM).

### Tetrastichus decrescens

Graham, 1991

76BD41F5-8899-5259-A710-C9919A42CE01

Tetrastichus decrescens Graham 1991:271–272. Holotype ♀ in NHM, examined (Fig. 43).

#### Description

See Graham (1991). Male is unknown.

#### Diagnosis

Antenna with F1 1.5× as long and 1.4× as wide as pedicel; mesoscutellum with distance between submedian grooves 1.2× the distance between submedian and sublateral grooves; female gaster elongate-acuminate, 2.0–2.2× as long as wide, ovipositor sheaths reach beyond apex of Gt_7_.

#### Distribution

(Former) Czechoslovakia, France, Sweden and United Kingdom (Graham 1991).

#### Ecology

##### Host

Unknown.

##### Material examined

Type material: holotype ♀ (NHM, type no. 5.3631). Additional material (7♀): France 1♀ (GD), Sweden 6♀ (MZLU, SMTP).

### Tetrastichus elanus sp. n.

7E1C5498-6537-5AE9-8060-8FA0F1789ED8

urn:lsid:zoobank.org:act:54E6D90B-52D8-431D-804D-3127A4D79C43

#### Materials

- **Type status:** Holotype. **Occurrence:** occurrenceDetails: http://www.boldsystems.org/index.php/API_Public/specimen?ids=BC-ZSM-HYM-29814-C07; catalogNumber: BC-ZSM-HYM-29814-C07; recordNumber: BC-ZSM-HYM-29814-C07; recordedBy: C. Hansson; individualID: BC-ZSM-HYM-29814-C07; individualCount: 1; sex: female; lifeStage: adult; **Taxon:** scientificName: Tetrastichus elanus; phylum: Arthropoda; class: Insecta; order: Hymenoptera; family: Eulophidae; genus: Tetrastichinae; taxonRemarks: Holotype deposited in MZLU; **Location:** country: Sweden; decimalLatitude: 55.7078; decimalLongitude: 13.475; **Record Level:** type: PhysicalObject; language: en; institutionCode: MZLU; basisOfRecord: PreservedSpecimen

#### Description

FEMALE holotype (Fig. 44). Body length 1.5 mm. *Head*. Width/length (dorsal view) 2.2, width/length (frontal view) 1.2, POL/OOL 1.6, widths head/mesosoma 1.1, mouth width/malar space 1.2, malar space/eye height 0.5. *Antenna*. Scape length/eye height 0.9, pedicel+flagellum length/mesosoma width 1.7, length/width F1, F2, F3 1.6, 1.8, 1.7, clava length/width 3.7, lengths pedicel/F1 0.6, lengths F1/F2 0.9, F1/F3 0.9, lengths F1, F2, F3/clava 0.4, 0.5, 0.5, widths F1/pedicel (dorsal view) 1.7, lengths antennal spicule/C3 0.3. *Mesosoma*. Length/width 1.4, mesoscutal mid-lobe length/width (width measured in anterior part) 0.9, mid-lobe with a median groove in posterior ⅔, with three adnotaular setae on each side, lengths mesoscutum/mesoscutellum (measured medially) 0.7, mesoscutellum length/width 0.8, length/width of enclosed space between submedian grooves 1.9, distance between SMG/distance between SMG and SLG 1.6, lengths dorsellum/propodeum (measured medially) 0.9, propodeum with strong reticulation, propodeal callus with two setae. *Fore wing*. Costal cell length/width 14.7, lengths costal cell/marginal vein 0.9, lengths marginal/stigmal veins 2.5. *Gaster*. Elongate, length/width 2.2, lengths gaster/mesosoma 1.6, Gt_7_ length/width 0.7, length of longest cercal seta/next longest seta 1.6, longest cercal seta slightly kinked in apical ¼, ovipositor sheaths projecting slightly beyond apex of Gt_7_.

Colour. Head, mesoscutum and propodeum metallic bluish-green, mesoscutellum and gaster golden-green, entire antenna dark brown, tegulae black, wings hyaline with veins yellowish-brown, coxae and femora black with metallic tinges, trochanters dark brown, tibiae yellowish-brown, fore tarsus dark brown, mid and hind tarsi yellowish-white with T4 brown.

MALE. Unknown.

#### Diagnosis

Antenna with F1 1.6× as long and 1.7× as wide as pedicel; mesoscutellum with distance between submedian grooves 1.6× the distance between submedian and sublateral grooves and with enclosed area between submedian grooves 1.9× as long as wide; female gaster elongate-acuminate, 2.2× as long as wide, ovipositor sheaths reach beyond apex of Gt_7_.

#### Distribution

Sweden.

#### Ecology

##### Host

Unknown.

#### Notes

Holotype deposited in MZLU.

### Tetrastichus ennis sp. n.

63AB26AF-87D2-5E07-B827-0EE17381523D

urn:lsid:zoobank.org:act:92CF571A-6977-4FE1-B6C0-246AE0780E9E

#### Materials

- **Type status:** Holotype. **Occurrence:** occurrenceDetails: http://www.boldsystems.org/index.php/API_Public/specimen?ids=BC-ZSM-HYM-27768-B04; catalogNumber: BC-ZSM-HYM-27768-B04; recordNumber: BC-ZSM-HYM-27768-B04; recordedBy: J.S. Noyes; individualID: BC-ZSM-HYM-27768-B04; individualCount: 1; sex: female; lifeStage: adult; **Taxon:** scientificName: Tetrastichus ennis; phylum: Arthropoda; class: Insecta; order: Hymenoptera; family: Eulophidae; genus: Tetrastichinae; taxonRemarks: Holotype deposited in NHM; **Location:** country: Romania; decimalLatitude: 47.011; decimalLongitude: 27.603; **Record Level:** type: PhysicalObject; language: en; institutionCode: NHM; basisOfRecord: PreservedSpecimen
- **Type status:** Paratype. **Occurrence:** occurrenceDetails: http://www.boldsystems.org/index.php/API_Public/specimen?ids=BC-ZSM-HYM-27770-A07; catalogNumber: BC-ZSM-HYM-27760-A07; recordNumber: BC-ZSM-HYM-27760-A07; recordedBy: J.S. Noyes; individualID: BC-ZSM-HYM-27770-A07; individualCount: 1; sex: female; lifeStage: adult; **Taxon:** scientificName: Tetrastichus ennis; phylum: Arthropoda; class: Insecta; order: Hymenoptera; family: Eulophidae; genus: Tetrastichinae; **Location:** country: Romania; decimalLatitude: 46.9853; decimalLongitude: 27.5847; **Record Level:** type: PhysicalObject; language: en; institutionCode: NHM; basisOfRecord: PreservedSpecimen
- **Type status:** Paratype. **Occurrence:** occurrenceDetails: http://www.boldsystems.org/index.php/API_Public/specimen?ids=BC-ZSM-HYM-22524-F08; catalogNumber: BC-ZSM-HYM-22524-F08; recordNumber: BC-ZSM-HYM-22524-F08; recordedBy: E. Shevtsova; individualID: BC-ZSM-HYM-22524-F08; individualCount: 1; sex: female; lifeStage: adult; **Taxon:** scientificName: Tetrastichus ennis; phylum: Arthropoda; class: Insecta; order: Hymenoptera; family: Eulophidae; genus: Tetrastichinae; **Location:** country: Russia; decimalLatitude: 59.567; decimalLongitude: 30.133; **Record Level:** type: PhysicalObject; language: en; institutionCode: MZLU; basisOfRecord: PreservedSpecimen
- **Type status:** Paratype. **Occurrence:** occurrenceDetails: http://www.boldsystems.org/index.php/API_Public/specimen?ids=BC-ZSM-HYM-27770-A07; catalogNumber: BC-ZSM-HYM-27760-A07; recordNumber: BC-ZSM-HYM-27760-A07; recordedBy: J.S. Noyes; individualID: BC-ZSM-HYM-27770-A07; individualCount: 1; sex: F; lifeStage: a; reproductiveCondition: S; associatedMedia: http://www.boldsystems.org/pics/BCHYM/BC-ZSM-HYM-27760-A07+1449268698.jpg; **Taxon:** scientificName: Tetrastichus ennis; phylum: Arthropoda; class: Insecta; order: Hymenoptera; family: Eulophidae; genus: Tetrastichus; **Location:** country: Romania; locality: Barnova Forest; decimalLatitude: 46.9853; decimalLongitude: 27.5847; **Identification:** identifiedBy: Christer Hansson

#### Description

FEMALE holotype (Fig. 45). Body length 1.8 mm (paratypes 1.5–1.7 mm). *Head*. Width/length in dorsal view 2.3, width/length in frontal view 1.3, POL/OOL 1.7, widths head/mesosoma 1.1, mouth width/malar space 1.2, malar space/eye height 0.7. *Antenna*. Scape length/eye height 1.0, pedicel+flagellum length/mesosoma width 1.3, length/width F1, F2, F3 1.6, 1.7, 1.7, clava length/width 3.8, lengths pedicel/F1 0.9, lengths F1/F2 0.9, F1/F3 0.9, lengths F1, F2, F3/clava 0.4, 0.4, 0.4, widths F1/pedicel (dorsal view) 1.3, lengths antennal spicule/C3 0.3. *Mesosoma*. Length/width 1.4, mesoscutal mid-lobe length/width 1.0 (width measured in anterior part), mid-lobe with median groove in posterior ½, with three adnotaular setae on each side, lengths mesoscutum/mesoscutellum (measured medially) 1.5, mesoscutellum length/width 0.8, length/width of enclosed space between submedian grooves 2.2, distance between SMG/distance between SMG and SLG 1.8, lengths dorsellum/propodeum 0.8, propodeum with weak reticulation, propodeal callus with three setae. *Fore wing*. Costal cell length/width 10.0, lengths costal cell/marginal vein 1.2, lengths marginal/stigmal veins 2.6. *Gaster*. Ovate, length/width 1.6, lengths gaster/head+mesosoma 0.9, Gt_7_ length/width 0.5, length of longest cercal seta/next longest seta 1.3, longest cercal seta evenly curved, ovipositor sheaths not reaching apex of Gt_7_.

Colour. Body with weak metallic blue tinges, entire antenna dark brown, tegulae black, wings hyaline with venation yellowish-white, coxae and femora concolorous with body, trochanters dark brown, fore tibia yellowish-brown, mid and hind tibiae dark brown, tarsi brownish.

MALE. Unknown.

#### Diagnosis

Female gaster ovate, 1.6× as long as wide. See key for separation from similar species.

#### Distribution

Romania and Russia.

#### Ecology

##### Host

Unknown.

#### Notes

Holotype deposited in NHM, paratype in MZLU and NHM.

### Tetrastichus epilachnae

(Giard, 1896)

6635A644-FD51-5F98-A2A7-6C77A1833B5C

Lygellus epilachnae Giard 1896:839. Type material not located. Transferred to *Tetrastichus* by Domenichini 1966:92.
Tetrastichus Jablonowskii Szelenyi 1940:85, 86–88. Type material destroyed Graham 1991:270. Synonymised by Graham 1991:269.

#### Description

See Graham (1991).

#### Diagnosis

Very similar to *T. clito* , female differs in having a longer and more acute gaster; hosts are different (Graham 1991).

#### Distribution

France, Hungary, Sweden (Graham 1991).

#### Ecology

##### Host

*Epilachna argus* (Geoffroy in Fourcroy), *E. chrysomelina* F., *Subcoccinella vigintiquattropunctata* L. (Coleoptera: Coccinellidae) (Graham 1991).

##### Material examined

No material has been examined.

#### Material examined

### Tetrastichus fenrisi sp. n.

A7C3E994-1503-5696-83E6-F5EBBF200E93

urn:lsid:zoobank.org:act:91B3378D-6EE4-47A4-8CDE-E4E3C94A43E2

#### Materials

- **Type status:** Holotype. **Occurrence:** occurrenceDetails: http://www.boldsystems.org/index.php/API_Public/specimen?ids=BC-ZSM-HYM-27493-G06; catalogNumber: BC-ZSM-HYM-27493-G06; recordNumber: BC-ZSM-HYM-27493-G06; recordedBy: SMTP; individualID: BC-ZSM-HYM-27493-G06; individualCount: 1; sex: female; lifeStage: adult; **Taxon:** scientificName: Tetrastichus fenrisi; phylum: Arthropoda; class: Insecta; order: Hymenoptera; family: Eulophidae; genus: Tetrastichinae; taxonRemarks: Holotype deposited in SMTP; **Location:** country: Sweden; decimalLatitude: 58.553; decimalLongitude: 17.313; **Record Level:** type: PhysicalObject; language: en; institutionCode: SMTP; basisOfRecord: PreservedSpecimen

#### Description

FEMALE holotype (Fig. 46). Body length 1.7 mm. *Head.* Width/length in dorsal view 2.3, width/length in frontal view 1.3, POL/OOL 1.8, widths head/mesosoma 1.1, mouth width/malar space 0.9, malar space/eye height 1.1. *Antenna.* Scape length/eye height 1.0, pedicel+flagellum length/mesosoma width 1.2, length/width F1, F2, F3 1.3, 1.4, 1.4, clava length/width 3.7, lengths pedicel/F1 1.0, lengths F1/F2 0.9, F1/F3 0.9, lengths F1, F2, F3/clava 0.3, 0.4, 0.4, widths F1/pedicel (dorsal view) 1.3, lengths antennal spicule/C3 0.2. *Mesosoma.* Length/width 1.4, mesoscutal mid-lobe length/width 1.1, mid-lobe with a weak median groove in posterior ½, with three adnotaular setae on each side, lengths mesoscutum/mesoscutellum (measured medially) 1.6, mesoscutellum length/width 0.9, length/width of enclosed space between submedian grooves 2.8, distance between submedian groove to distance between submedian and sublateral grooves 1.0, lengths dorsellum/propodeum 0.6, propodeum with strong reticulation, propodeal callus with two setae. *Fore wing.* Costal cell length/width 14.0, lengths costal cell/marginal vein 1.0, lengths marginal/stigmal veins 2.8. *Gaster.* Ovate, length/width 1.6, lengths gaster/mesosoma 1.2, Gt_7_ length/width 0.3, length of longest cercal seta/next longest seta 1.3, longest cercal seta curved, ovipositor sheaths not reaching Gt_7_.

Colour. Head and mesoscutum black with weak metallic tinges, mesoscutellum dark brown, dorsellum pale brown, propodeum black, gaster dark brown with metallic tinges, antenna dark brown, tegulae dark brown, wings hyaline with venation yellowish-brown, coxae, trochanters and femora dark brown, fore tibia infuscate, mid and hind tibiae dark brown, fore tarsus dark brown, mid and hind tarsi dark brown with T1 yellowish-white.

MALE. Unknown.

#### Diagnosis

Mesoscutellum with distance between submedian groove 1.0× the distance between submedian and sublateral grooves and median part with weaker reticulation and more shiny than lateral parts; mesoscutum black with weak metallic tinges, mesoscutellum dark brown, dorsellum pale brown; fore tibia infuscate, mid and hind tibiae dark brown, fore tarsus dark brown, mid and hind tarsi dark brown with T1 yellowish-white.

#### Distribution

Sweden.

#### Ecology

##### Host

Unknown.

#### Notes

Holotype deposited in SMTP.

### Tetrastichus ladrus sp. n.

A87BE869-4C08-5FCF-8A92-678C13645032

urn:lsid:zoobank.org:act:83F01BA8-FCE6-4E2A-82B6-2A921F09AEBB

#### Materials

- **Type status:** Holotype. **Occurrence:** occurrenceDetails: http://www.boldsystems.org/index.php/API_Public/specimen?ids=BC-ZSM-HYM-20721-F02; catalogNumber: BC-ZSM-HYM-20721-F02; recordNumber: BC-ZSM-HYM-20721-F02; recordedBy: C. Hansson; individualID: BC-ZSM-HYM-20721-F02; individualCount: 1; sex: female; lifeStage: adult; **Taxon:** scientificName: Tetrastichus ladrus; phylum: Arthropoda; class: Insecta; order: Hymenoptera; family: Eulophidae; genus: Tetrastichinae; taxonRemarks: Holotype deposited in MZLU; **Location:** country: Sweden; decimalLatitude: 55.7; decimalLongitude: 13.45; **Record Level:** type: PhysicalObject; language: en; institutionCode: MZLU; basisOfRecord: PreservedSpecimen

#### Description

FEMALE holotype (Fig. 47). Body length 1.6 mm. *Head*. Width/length in dorsal view 2.3, width/length in frontal view 1.3, POL/OOL 1.7, widths head/mesosoma 1.1, mouth width/malar space 1.2, malar space/eye height 0.7. *Antenna*. Scape length/eye height 1.0, pedicel+flagellum length/mesosoma width 1.3, length/width F1, F2, F3 1.4, 1.4, 1.3, clava length/width 3.7, lengths pedicel/F1 0.9, lengths F1/F2 0.9, F1/F3 1.0, lengths F1, F2, F3/clava 0.4, 0.4, 0.4, widths F1/pedicel (dorsal view) 1.3, lengths antennal spicule/C3 0.3. *Mesosoma*. Length/width 1.4, mesoscutal mid-lobe length/width 0.9 (width measured in anterior part), mid-lobe with median groove in posterior ½, with two adnotaular setae on each side, lengths mesoscutum/mesoscutellum (measured medially) 1.4, mesoscutellum length/width 0.8, length/width of enclosed space between submedian grooves 2.3, distance between SMG/distance between SMG and SLG 1.4, lengths dorsellum/propodeum 0.7, propodeum with strong reticulation, propodeal callus with two setae. *Fore wing*. Costal cell length/width 13.0, lengths costal cell/marginal vein 0.9, lengths marginal/stigmal veins 3.1. *Gaster*. Ovate, length/width 1.4, lengths gaster/head+mesosoma 0.9, Gt_7_ length/width 0.4, length of longest cercal seta/next longest seta 1.3, longest cercal seta almost straight, ovipositor sheaths not reaching apex of Gt_7_.

Colour. Body with weak metallic greenish-blue tinges, entire antenna dark brown, tegulae dark brown, wings hyaline with venation brown to fuscous, coxae and femora concolorous with body, trochanters dark brown, tibiae brownish, fore tarsus brownish, mid and hind tarsi yellowish-brown with T4 brown.

MALE. Unknown.

#### Diagnosis

Female gaster ovate, 1.4× as long as wide. See key for separation from similar species.

#### Distribution

Sweden.

#### Ecology

##### Host

Unknown.

#### Notes

Holotype deposited in MZLU.

### Tetrastichus lazius sp. n.

7C5B629F-C8D5-5635-BCD9-C065FA4392A4

urn:lsid:zoobank.org:act:2D7C33A0-5750-4257-8DBA-5D478FDDDC15

#### Materials

- **Type status:** Holotype. **Occurrence:** occurrenceDetails: http://www.boldsystems.org/index.php/API_Public/specimen?ids=BC-ZSM-HYM-29813-G01; catalogNumber: BC-ZSM-HYM-29813-G01; recordNumber: BC-ZSM-HYM-29813-G01; recordedBy: Munro et al; individualID: BC-ZSM-HYM-29813-G01; individualCount: 1; sex: female; lifeStage: adult; **Taxon:** scientificName: Tetrastichus lazius; phylum: Arthropoda; class: Insecta; order: Hymenoptera; family: Eulophidae; genus: Tetrastichinae; taxonRemarks: Holotype deposited in UCRC; **Location:** country: Italy; decimalLatitude: 41.6552; decimalLongitude: 12.9896; **Record Level:** type: PhysicalObject; language: en; institutionCode: UCRC; basisOfRecord: PreservedSpecimen

#### Description

FEMALE holotype (Fig. 48). Body length 1.9 mm. *Head*. Width/length in dorsal view 2.4, width/length in frontal view 1.3, POL/OOL 1.4, widths head/mesosoma 1.2, mouth width/malar space 1.2, malar space/eye height 0.8. *Antenna*. Scape length/eye height 1.0, pedicel+flagellum length/mesosoma width 1.3, length/width F1, F2, F3 1.4, 1.7, 1.4, clava length/width 4.2, lengths pedicel/F1 1.0, lengths F1/F2 0.9, F1/F3 1.0, lengths F1, F2, F3/clava 0.3, 0.4, 0.3, widths F1/pedicel (dorsal view) 1.3, lengths antennal spicule/C3 0.3. *Mesosoma*. Length/width 1.5, mesoscutal mid-lobe length/width 1.1 (width measured in anterior part), mid-lobe with median groove in posterior 4/5, with three adnotaular setae on each side, lengths mesoscutum/mesoscutellum (measured medially) 1.6, mesoscutellum length/width 1.0, length/width of enclosed space between submedian grooves 2.4, distance between SMG/distance between SMG and SLG 1.0, lengths dorsellum/propodeum 0.6, propodeum with strong reticulation, propodeal callus with two setae. *Fore wing*. Costal cell length/width 10.4, lengths costal cell/marginal vein 0.9, lengths marginal/stigmal veins 2.8. *Gaster*. Ovate, length/width nm, lengths gaster/head+mesosoma nm, Gt_7_ length/width 0.4, length of longest cercal seta/next longest seta 1.5, longest cercal seta curved, ovipositor sheaths not reaching apex of Gt_7_.

Colour. Body metallic blue, antenna dark brown, tegulae black, wings hyaline with venation yellowish-brown, coxae and hind femur concolorous with body, trochanters and fore and mid femora dark brown, tibiae pale yellowish-brown, fore tarsus pale brown, mid and hind tarsi yellowish-white with T4 pale brown.

MALE. Unknown.

#### Diagnosis

Mesosoma distinctly blue metallic. See key for separation from similar species.

#### Distribution

Italy.

#### Ecology

##### Host

Unknown.

#### Notes

Holotype deposited in UCRC.

### Tetrastichus lixalius sp. n.

D92ECD9F-14CC-58B4-89F1-FB1B7F2E1E97

urn:lsid:zoobank.org:act:15D723F0-2C0F-496A-B72E-D77124F0D161

#### Materials

- **Type status:** Holotype. **Occurrence:** occurrenceDetails: http://www.boldsystems.org/index.php/API_Public/specimen?ids=BC-ZSM-HYM-21587-A03; catalogNumber: BC-ZSM-HYM-21587-A03; recordNumber: BC-ZSM-HYM-21587-A03; recordedBy: C.Hansson; individualID: BC-ZSM-HYM-21587-A03; individualCount: 1; sex: female; lifeStage: adult; **Taxon:** scientificName: Tetrastichus lixalius; phylum: Arthropoda; class: Insecta; order: Hymenoptera; family: Eulophidae; genus: Tetrastichinae; taxonRemarks: Holotype deposited in MZLU; **Location:** country: Sweden; decimalLatitude: 56.625; decimalLongitude: 16.702; **Record Level:** type: PhysicalObject; language: en; institutionCode: MZLU; basisOfRecord: PreservedSpecimen
- **Type status:** Paratype. **Occurrence:** occurrenceDetails: http://www.boldsystems.org/index.php/API_Public/specimen?ids=BC-ZSM-HYM-21587-D09; catalogNumber: BC-ZSM-HYM-21587-D09; recordNumber: BC-ZSM-HYM-21587-D09; recordedBy: C.Hansson; individualID: BC-ZSM-HYM-21587-D09; individualCount: 1; sex: female; lifeStage: adult; **Taxon:** scientificName: Tetrastichus lixalius; phylum: Arthropoda; class: Insecta; order: Hymenoptera; family: Eulophidae; genus: Tetrastichinae; **Location:** country: Sweden; decimalLatitude: 56.57; decimalLongitude: 16.531; **Record Level:** type: PhysicalObject; language: en; basisOfRecord: PreservedSpecimen
- **Type status:** Paratype. **Occurrence:** occurrenceDetails: http://www.boldsystems.org/index.php/API_Public/specimen?ids=BC-ZSM-HYM-21587-D10; catalogNumber: BC-ZSM-HYM-21587-D10; recordNumber: BC-ZSM-HYM-21587-D10; recordedBy: C.Hansson; individualID: BC-ZSM-HYM-21587-D10; individualCount: 1; sex: female; lifeStage: adult; **Taxon:** scientificName: Tetrastichus lixalius; phylum: Arthropoda; class: Insecta; order: Hymenoptera; family: Eulophidae; genus: Tetrastichinae; **Location:** country: Sweden; decimalLatitude: 56.57; decimalLongitude: 16.531; **Record Level:** type: PhysicalObject; language: en; basisOfRecord: PreservedSpecimen
- **Type status:** Paratype. **Occurrence:** occurrenceDetails: http://www.boldsystems.org/index.php/API_Public/specimen?ids=BC-ZSM-HYM-13565-G10; catalogNumber: BC-ZSM-HYM-13565-G10; recordNumber: BC-ZSM-HYM-13565-G10; recordedBy: SMTP; individualID: BC-ZSM-HYM-13565-G10; individualCount: 1; sex: male; lifeStage: adult; **Taxon:** scientificName: Tetrastichus lixalius; phylum: Arthropoda; class: Insecta; order: Hymenoptera; family: Eulophidae; genus: Tetrastichinae; **Location:** country: Sweden; decimalLatitude: 56.378; decimalLongitude: 16.295; **Record Level:** type: PhysicalObject; language: en; basisOfRecord: PreservedSpecimen
- **Type status:** Paratype. **Occurrence:** occurrenceDetails: http://www.boldsystems.org/index.php/API_Public/specimen?ids=BC-ZSM-HYM-20721-F05; catalogNumber: BC-ZSM-HYM-20721-F05; recordNumber: BC-ZSM-HYM-20721-F05; recordedBy: C. Hansson; individualID: BC-ZSM-HYM-20721-F05; individualCount: 1; sex: female; lifeStage: adult; **Taxon:** scientificName: Tetrastichus lixalius; phylum: Arthropoda; class: Insecta; order: Hymenoptera; family: Eulophidae; genus: Tetrastichinae; **Location:** country: Sweden; decimalLatitude: 55.533; decimalLongitude: 12.917; **Record Level:** type: PhysicalObject; language: en; basisOfRecord: PreservedSpecimen
- **Type status:** Paratype. **Occurrence:** occurrenceDetails: http://www.boldsystems.org/index.php/API_Public/specimen?ids=BC-ZSM-HYM-20721-C08; catalogNumber: BC-ZSM-HYM-20721-C08; recordNumber: BC-ZSM-HYM-20721-C08; recordedBy: C. Hansson; individualID: BC-ZSM-HYM-20721-C08; individualCount: 1; sex: male; lifeStage: adult; **Taxon:** scientificName: Tetrastichus lixalius; phylum: Arthropoda; class: Insecta; order: Hymenoptera; family: Eulophidae; genus: Tetrastichinae; **Location:** country: Sweden; decimalLatitude: 55.533; decimalLongitude: 13.133; **Record Level:** type: PhysicalObject; language: en; basisOfRecord: PreservedSpecimen

#### Description

FEMALE holotype (Fig. 49). Body length 1.9 mm (paratypes 1.7–1.9 mm). *Head*. Width/length in dorsal view 2.4, width/length in frontal view 1.3, POL/OOL 1.9, widths head/mesosoma 1.1, mouth width/malar space 1.1, malar space/eye height 0.7. *Antenna*. Scape length/eye height 0.9, pedicel+flagellum length/mesosoma width 1.2, length/width F1, F2, F3 1.3, 1.3, 1.2, clava length/width 2.8, lengths pedicel/F1 0.9, lengths F1/F2 1.0, F1/F3 1.1, lengths F1, F2, F3/clava 0.4, 0.4, 0.4, widths F1/pedicel (dorsal view) 1.4, lengths antennal spicule/C3 0.3. *Mesosoma*. Length/width 1.4, mesoscutal mid-lobe length/width 1.0 (width measured in anterior part), mid-lobe without median groove, with four adnotaular setae on each side, lengths mesoscutum/mesoscutellum (measured medially) 1.4, mesoscutellum length/width 0.8, length/width of enclosed space between submedian grooves 2.6, distance between SMG/distance between SMG and SLG 1.3, lengths dorsellum/propodeum 0.6, propodeum with strong reticulation, propodeal callus with two setae. *Fore wing*. Costal cell length/width 14.4, lengths costal cell/marginal vein 1.1, lengths marginal/stigmal veins 3.6. *Gaster*. Long ovate, length/width 1.8, lengths gaster/head+mesosoma 0.9, Gt_7_ length/width 0.6, length of longest cercal seta/next longest seta 1.6, longest cercal seta evenly curved, ovipositor sheaths not reaching apex of Gt_7_.

Colour. Body with weak metallic greenish-blue tinges, entire antenna dark brown, tegulae black, wing hyaline with venation brown to fuscous, coxae and femora concolorous with body, trochanters dark brown, fore tibia yellowish-brown, mid tibia brownish, hind tibia dark brown, tarsi brownish with T4 darkest.

MALE. Body length 1.6–1.7 mm. *Head*. Width/length in dorsal view 2.4, width/length in frontal view 1.3, mouth width/malar space 1.2, widths head/mesosoma 1.1, eye height/malar space 1.3. *Antenna*. F1–F4 with basal whorls of setae, reaching beyond apex of corresponding flagellomere, scape length/eye height 1.0, scape length/width 2.6, ventral plaque placed in central part of scape, lengths ventral plaque/scape 0.8, pedicel+flagellum length/mesosoma width 1.2, length/width F1, F2, F3, F4 1.2, 1.6, 1.8, 1.7, clava length/width 3.9, lengths pedicel/F1 1.0, lengths F1/F2 0.8, F1/F3 0.7, F1/F4 0.7, lengths F1, F2, F3, F4/clava 0.3, 0.4, 0.5, 0.4.

Colour as in female.

#### Diagnosis

Female gaster long ovate, 1.8× as long as wide. See key for separation from similar species.

#### Distribution

Sweden.

#### Ecology

##### Host

Unknown.

#### Notes

Holotype deposited in MZLU, paratypes in MZLU and SMTP.

### Tetrastichus lycus sp. n.

CC5D63FC-9BF5-5E23-94F2-67A65236D51D

urn:lsid:zoobank.org:act:89E861D0-839F-4869-962A-3E9CF3FEA956

#### Materials

- **Type status:** Holotype. **Occurrence:** occurrenceDetails: http://www.boldsystems.org/index.php/API_Public/specimen?ids=BC-ZSM-HYM-27770-B05; catalogNumber: BC-ZSM-HYM-27760-B05; recordNumber: BC-ZSM-HYM-27760-B05; recordedBy: J.S. Noyes; individualID: BC-ZSM-HYM-27770-B05; individualCount: 1; sex: female; lifeStage: adult; **Taxon:** scientificName: Tetrastichus lycus; phylum: Arthropoda; class: Insecta; order: Hymenoptera; family: Eulophidae; genus: Tetrastichinae; taxonRemarks: Holotype deposited in NHM; **Location:** country: Romania; decimalLatitude: 47.2442; decimalLongitude: 27.4828; **Record Level:** type: PhysicalObject; language: en; institutionCode: NHM; basisOfRecord: PreservedSpecimen
- **Type status:** Paratype. **Occurrence:** occurrenceDetails: http://www.boldsystems.org/index.php/API_Public/specimen?ids=BC-ZSM-HYM-27768-G09; catalogNumber: BC-ZSM-HYM-27768-G09; recordNumber: BC-ZSM-HYM-27768-G09; recordedBy: J.S. Noyes; individualID: BC-ZSM-HYM-27768-G09; individualCount: 1; sex: female; lifeStage: adult; **Taxon:** scientificName: Tetrastichus lycus; phylum: Arthropoda; class: Insecta; order: Hymenoptera; family: Eulophidae; genus: Tetrastichinae; **Location:** country: Romania; decimalLatitude: 47.244; decimalLongitude: 27.483; **Record Level:** type: PhysicalObject; language: en; basisOfRecord: PreservedSpecimen
- **Type status:** Paratype. **Occurrence:** occurrenceDetails: http://www.boldsystems.org/index.php/API_Public/specimen?ids=BC-ZSM-HYM-29813-F08; catalogNumber: BC-ZSM-HYM-29813-F08; recordNumber: BC-ZSM-HYM-29813-F08; recordedBy: Munro et al; individualID: BC-ZSM-HYM-29813-F08; individualCount: 1; sex: female; lifeStage: adult; **Taxon:** scientificName: Tetrastichus lycus; phylum: Arthropoda; class: Insecta; order: Hymenoptera; family: Eulophidae; genus: Tetrastichinae; **Location:** country: Italy; decimalLatitude: 41.6552; decimalLongitude: 12.9896; **Record Level:** type: PhysicalObject; language: en; institutionCode: UCRC; basisOfRecord: PreservedSpecimen
- **Type status:** Paratype. **Occurrence:** occurrenceDetails: http://www.boldsystems.org/index.php/API_Public/specimen?ids=BC-ZSM-HYM-29813-F07; catalogNumber: BC-ZSM-HYM-29813-F07; recordNumber: BC-ZSM-HYM-29813-F07; recordedBy: Munro et al; individualID: BC-ZSM-HYM-29813-F07; individualCount: 1; sex: female; lifeStage: adult; **Taxon:** scientificName: Tetrastichus lycus; phylum: Arthropoda; class: Insecta; order: Hymenoptera; family: Eulophidae; genus: Tetrastichinae; **Location:** country: Italy; decimalLatitude: 41.6552; decimalLongitude: 12.9896; **Record Level:** type: PhysicalObject; language: en; institutionCode: UCRC; basisOfRecord: PreservedSpecimen

#### Description

FEMALE holotype (Fig. 50). Body length 1.6 mm (paratypes 1.6–1.7 mm). *Head*. Width/length in dorsal view 2.5, width/length in frontal view 1.3, POL/OOL 1.8, widths head/mesosoma 1.1, mouth width/malar space 1.1, malar space/eye height 0.8. *Antenna*. Scape length/eye height 1.0, pedicel+flagellum length/mesosoma width 1.2, length/width F1, F2, F3 1.4, 1.4, 1.8, clava length/width 3.8, lengths pedicel/F1 0.9, lengths F1/F2 1.0, F1/F3 0.9, lengths F1, F2, F3/clava 0.4, 0.4, 0.5, widths F1/pedicel (dorsal view) 1.4, lengths antennal spicule/C3 0.2. *Mesosoma*. Length/width 1.4, mesoscutal mid-lobe length/width 0.9 (width measured in anterior part), mid-lobe with a median groove in posterior ⅔, with three adnotaular setae on each side, lengths mesoscutum/mesoscutellum (measured medially) 1.3, mesoscutellum length/width 0.9, length/width of enclosed space between submedian grooves 2.5, distance between submedian/distance between submedian and sublateral grooves 1.3, lengths dorsellum/propodeum 0.7, propodeum with strong reticulation, propodeal callus with two setae. *Fore wing*. Costal cell length/width 10.1, lengths costal cell/marginal vein 0.9, lengths marginal/stigmal veins 2.9. *Gaster*. Ovate, length/width 1.5, lengths gaster/head+mesosoma 0.8, Gt_7_ length/width 0.4, ovipositor sheaths not reaching apex of Gt_7_.

Colour. Body with weak golden-green tinges, entire antenna dark brown, tegulae dark brown, wings hyaline with venation yellowish-brown to brown, coxae and femora concolorous with body, trochanters dark brown, tibiae pale brown, fore tarsus brown, mid and hind tarsi yellowish-brown with T4 brown.

MALE. Unknown.

#### Diagnosis

Female gaster ovate, 1.5× as long as wide. See key for separation from similar species.

#### Distribution

Italy, Romania.

#### Ecology

##### Host

Unknown.

#### Notes

Holotype deposited in NHM, paratypes in NHM, UCRC.

### Tetrastichus nymphae sp. n.

FC531BFD-9B8C-5A8B-9D20-06FBF260CA1D

urn:lsid:zoobank.org:act:15968C8B-DF5E-4A4C-88D2-8C843687128C

#### Materials

- **Type status:** Holotype. **Occurrence:** recordedBy: R. Gregorek & M. Suvak; individualID: BC-ZSM-HYM-26562-H10; individualCount: 1; sex: female; lifeStage: adult; **Taxon:** scientificName: Tetrastichus nymphae; phylum: Arthropoda; class: Insecta; order: Hymenoptera; family: Eulophidae; genus: Tetrastichinae; taxonRemarks: Holotype deposited in MZLU; **Location:** country: Slovakia; decimalLatitude: 48.3689; decimalLongitude: 21.705; **Record Level:** type: PhysicalObject; language: en; institutionCode: MZLU; basisOfRecord: PreservedSpecimen
- **Type status:** Paratype. **Occurrence:** occurrenceDetails: http://www.boldsystems.org/index.php/API_Public/specimen?ids=BC-ZSM-HYM-26562-H11; catalogNumber: BC-ZSM-HYM-26562-H11; recordNumber: BC-ZSM-HYM-26562-H11; recordedBy: R. Gregorek & M. Suvak; individualID: BC-ZSM-HYM-26562-H11; individualCount: 1; sex: female; lifeStage: adult; **Taxon:** scientificName: Tetrastichus nymphae; phylum: Arthropoda; class: Insecta; order: Hymenoptera; family: Eulophidae; genus: Tetrastichinae; **Location:** country: Slovakia; decimalLatitude: 48.3689; decimalLongitude: 21.705; **Record Level:** type: PhysicalObject; language: en; institutionCode: MZLU; basisOfRecord: PreservedSpecimen
- **Type status:** Paratype. **Occurrence:** occurrenceDetails: http://www.boldsystems.org/index.php/API_Public/specimen?ids=BC-ZSM-HYM-26562-H10; catalogNumber: BC-ZSM-HYM-26562-H10; recordNumber: BC-ZSM-HYM-26562-H10; recordedBy: R. Gregorek & M. Suvak; individualID: BC-ZSM-HYM-26562-H10; individualCount: 1; sex: male; lifeStage: adult; **Taxon:** scientificName: Tetrastichus nymphae; phylum: Arthropoda; class: Insecta; order: Hymenoptera; family: Eulophidae; genus: Tetrastichinae; **Location:** country: Slovakia; decimalLatitude: 48.3689; decimalLongitude: 21.705; **Record Level:** type: PhysicalObject; language: en; institutionCode: MZLU; basisOfRecord: PreservedSpecimen

#### Description

FEMALE holotype (Fig. 51). Body length 1.3 mm (paratypes 1.4 mm). *Head.* Width/length in dorsal view 2.5, width/length in frontal view 1.2, POL/OOL 1.8, widths head/mesosoma 0.9, mouth width/malar space 1.4, malar space/eye height 0.5. *Antenna.* Scape length/eye height 0.9, pedicel+flagellum length/mesosoma width nm, length/width F1, F2, F3 1.3, 1.2, 1.1, clava length/width nm, lengths pedicel/F1 0.9, lengths F1/F2 1.0, F1/F3 1.1, lengths F1, F2, F3/clava nm, widths F1/pedicel (dorsal view) 1.0, lengths antennal spicule/C3 nm. *Mesosoma.* Length/width 1.3, mesoscutal mid-lobe length/width 0.8, mid-lobe with a weak median groove that is absent in anterior ¼, with three adnotaular setae on each side, lengths mesoscutum/mesoscutellum (measured medially) 1.2, mesoscutellum length/width 1.0, length/width of enclosed space between submedian grooves 1.2, distance between SMG/distance between SMG and SLG 2.1, lengths dorsellum/propodeum 0.6, propodeum with rather strong reticulation and partly smooth, propodeal callus with two setae. *Fore wing.* Costal cell length/width 11.0, lengths costal cell/marginal vein 1.0, lengths marginal/stigmal veins 2.5. *Gaster.* Ovate, length/width 1.5, lengths gaster/mesosoma 1.2, Gt_7_ length/width 0.6, length of longest cercal seta/next longest seta 2.0, longest cercal seta sinuate, ovipositor sheaths projecting beyond Gt_7_.

Colour. Body dark brown to black non-metallic, antenna dark brown, tegulae black, wings hyaline with venation yellowish-brown, coxae, trochanters and femora concolorous with body, tibiae yellowish-brown, fore tarsus pale brown, mid and hind tarsi yellowish-brown with T4 brown.

MALE. Body length 1.3 mm. *Head.* Width/length in dorsal view 2.5, width/length in frontal view 1.3, eye height/malar space 1.5, mouth width/malar space 1.2, widths head/mesosoma 1.1. *Antenna.* F1–F4 with basal whorls of setae, reaching beyond apex of corresponding flagellomere, whorled setae on F1 2.0× as long as F1 length, scape length/eye height 1.0, scape length/width 2.6, ventral plaque placed centrally, lengths ventral plaque/scape 0.4, pedicel+flagellum length/mesosoma width 1.7, length/width F1, F2, F3, F4 1.3, 1.5, 1.4, 1.3, clava length/width 4.0, lengths pedicel/F1 1.2, lengths F1/F2 0.8, F1/F3 0.7, F1/F4 0.8, lengths F1, F2, F3 F4 /clava 0.3, 0.4, 0.4, 0.4.

Colour. Similar to female.

#### Diagnosis

Mesoscutellum ±flattened, distance between submedian groove 2.1× distance between submedian and sublateral grooves, female gaster ovate, 1.5× as long as wide, with ovipositor sheaths reaching beyond apex of Gt_7_.

#### Distribution

Slovakia.

#### Ecology

##### Host

Gregarious endoparasitoid on *Galerucella nymphaeae* (L.) (Coleoptera: Chrysomelidae) (Suvak et al. 2012 parasitoid identified as *Tetrastichus clito*).

##### Remarks

The type specimens are in poor condition, shrivelled and with parts missing: the holotype lacks antennal clava on both sides and left mid tibia and tarsus.

##### Additional paratype examined

1 ♀, same data as holotype (MZLU).

#### Notes

Holotype deposited in MZLU, paratypes in MZLU.

### Tetrastichus pilemostomae

Graham 1991

D6705BDF-F6EE-51D3-BDF5-F4B75E6A8063

Tetrastichus pilemostomae Graham 1991:270–271. Holotype ♀ in OUMNH (according to Graham 1991), but could not be found there (James Hogan in mail). See Fig. 52.

#### Description

See Graham (1991). Male is unknown.

#### Diagnosis

Antenna with F1 1.3× as long as and 1.3× as wide as pedicel; mesoscutellum with distance between submedian grooves 1.7–1.9× the distance between submedian and sublateral grooves; female gaster short ovate, 1.4× as long as wide, with ovipositor sheaths reaching beyond apex of Gt_7_.

#### Distribution

United Kingdom (Graham 1991), Sweden (Hedqvist 2003) and Romania (**new record**).

#### Ecology

##### Host

*Pilemostoma fastuosa* (Schaller) (Coleoptera: Chrysomelidae) (Graham 1991).

##### Material examined

Type material: 7♀ paratypes (1♀ with head missing) (NHM). Additional material: Romania 2♀ (NHM).

### Tetrastichus pixius sp. n.

E2A6D1CE-9FC7-5985-A136-A4D25A40C3E8

urn:lsid:zoobank.org:act:3805D744-51AE-4BA9-A17E-431437C89D4A

#### Materials

- **Type status:** Holotype. **Occurrence:** occurrenceDetails: http://www.boldsystems.org/index.php/API_Public/specimen?ids=BC-ZSM-HYM-26563-A02; catalogNumber: BC-ZSM-HYM-26563-A02; recordNumber: BC-ZSM-HYM-26563-A02; recordedBy: C. Hansson; individualID: BC-ZSM-HYM-26563-A02; individualCount: 1; sex: female; lifeStage: adult; **Taxon:** scientificName: Tetrastichus pixius; phylum: Arthropoda; class: Insecta; order: Hymenoptera; family: Eulophidae; genus: Tetrastichinae; taxonRemarks: Holotype deposited in MZLU; **Location:** country: Sweden; decimalLatitude: 55.6619; decimalLongitude: 13.5472; **Record Level:** type: PhysicalObject; language: en; institutionCode: MZLU; basisOfRecord: PreservedSpecimen
- **Type status:** Paratype. **Occurrence:** occurrenceDetails: http://www.boldsystems.org/index.php/API_Public/specimen?ids=BC-ZSM-HYM-26563-A04; catalogNumber: BC-ZSM-HYM-26563-A04; recordNumber: BC-ZSM-HYM-26563-A04; recordedBy: C. Hansson; individualID: BC-ZSM-HYM-26563-A04; individualCount: 1; sex: female; lifeStage: adult; **Taxon:** scientificName: Tetrastichus pixius; phylum: Arthropoda; class: Insecta; order: Hymenoptera; family: Eulophidae; genus: Tetrastichinae; **Location:** country: Sweden; decimalLatitude: 55.6619; decimalLongitude: 13.5472; **Record Level:** type: PhysicalObject; language: en; basisOfRecord: PreservedSpecimen
- **Type status:** Paratype. **Occurrence:** occurrenceDetails: http://www.boldsystems.org/index.php/API_Public/specimen?ids=BC-ZSM-HYM-26563-A03; catalogNumber: BC-ZSM-HYM-26563-A03; recordNumber: BC-ZSM-HYM-26563-A03; recordedBy: C. Hansson; individualID: BC-ZSM-HYM-26563-A03; individualCount: 1; sex: female; lifeStage: adult; **Taxon:** scientificName: Tetrastichus pixius; phylum: Arthropoda; class: Insecta; order: Hymenoptera; family: Eulophidae; genus: Tetrastichinae; **Location:** country: Sweden; decimalLatitude: 55.6619; decimalLongitude: 13.5472; **Record Level:** type: PhysicalObject; language: en; basisOfRecord: PreservedSpecimen
- **Type status:** Paratype. **Occurrence:** occurrenceDetails: http://www.boldsystems.org/index.php/API_Public/specimen?ids=BC-ZSM-HYM-21587-C11; catalogNumber: BC-ZSM-HYM-21587-C11; recordNumber: BC-ZSM-HYM-21587-C11; recordedBy: C.Hansson; individualID: BC-ZSM-HYM-21587-C11; individualCount: 1; sex: female; lifeStage: adult; **Taxon:** scientificName: Tetrastichus pixius; phylum: Arthropoda; class: Insecta; order: Hymenoptera; family: Eulophidae; genus: Tetrastichinae; **Location:** country: Sweden; decimalLatitude: 55.84; decimalLongitude: 13.58; **Record Level:** type: PhysicalObject; language: en; institutionCode: MZLU; basisOfRecord: PreservedSpecimen
- **Type status:** Paratype. **Occurrence:** occurrenceDetails: http://www.boldsystems.org/index.php/API_Public/specimen?ids=BC-ZSM-HYM-22523-D06; catalogNumber: BC-ZSM-HYM-22523-D06; recordNumber: BC-ZSM-HYM-22523-D06; recordedBy: Swedish Malaise Trap Project; individualID: BC-ZSM-HYM-22523-D06; individualCount: 1; sex: female; lifeStage: adult; **Taxon:** scientificName: Tetrastichus pixius; phylum: Arthropoda; class: Insecta; order: Hymenoptera; family: Eulophidae; genus: Tetrastichinae; **Location:** country: Sweden; decimalLatitude: 56.9216; decimalLongitude: 16.1012; **Record Level:** type: PhysicalObject; language: en; institutionCode: SMTP; basisOfRecord: PreservedSpecimen

#### Description

FEMALE holotype (Fig. 53). Body length 1.6 mm (paratypes 1.3–1.6 mm). *Head*. Width/length in dorsal view 2.3, width/length in frontal view 1.2, POL/OOL 1.9, widths head/mesosoma 1.1, mouth width/malar space 0.9, malar space/eye height 0.8. *Antenna*. Scape length/eye height 0.9, pedicel+flagellum length/mesosoma width 1.3, length/width F1, F2, F3 1.5, 1.6, 1.4, clava length/width 3.9, lengths pedicel/F1 1.0, lengths F1/F2 0.9, F1/F3 0.9, lengths F1, F2, F3/clava 0.3, 0.4, 0.4, widths F1/pedicel (dorsal view) 1.3, lengths antennal spicule/C3 0.3. *Mesosoma*. Length/width 1.4, mesoscutal mid-lobe length/width 1.0 (width measured in anterior part), mid-lobe with median groove in posterior ½, with three adnotaular setae on each side, lengths mesoscutum/mesoscutellum (measured medially) 1.5, mesoscutellum length/width 0.8, length/width of enclosed space between submedian grooves 2.7, distance between SMG/distance between SMG and SLG 1.0, lengths dorsellum/propodeum 0.7, propodeum with strong reticulation, propodeal callus with two setae. *Fore wing*. Costal cell length/width 13.5, lengths costal cell/marginal vein 1.0, lengths marginal/stigmal veins 3.3. *Gaster*. Ovate, length/width 1.3, lengths gaster/head+mesosoma 0.9, Gt_7_ length/width 0.4, length of longest cercal seta/next longest seta 1.5, longest cercal seta almost straight, ovipositor sheaths not reaching apex of Gt_7_.

Colour. Body with weak metallic blue tinges, entire antenna dark brown, tegulae dark brown, wings hyaline with venation yellowish-white, coxae and femora concolorous with body, trochanters dark brown, fore tibia yellowish-brown, mid and hind tibiae brownish, fore tarsus brown, mid and hind tarsi yellowish-brown with T3–4 brown.

MALE. Unknown.

#### Diagnosis

Female gaster ovate, 1.3× as long as wide. See key for separation from similar species.

#### Distribution

Sweden.

#### Ecology

##### Host

Unknown.

#### Notes

Holotype deposited in MZLU, paratypes in MZLU and SMTP.

### Tetrastichus unplaced species

DCE4969E-6348-5FDE-8B10-705F699BE6A8

#### Taxon discussion

The following species could not be assigned to any of the existing species groups.

### Tetrastichus acutiusculus

Graham, 1991

7467FC28-A062-5A84-BCB9-FB1AAE2269C9

Tetrastichus acutiusculus Graham 1991:261. Holotype ♀ in NHM, examined (Fig. 54).

#### Materials

- **Type status:** Other material. **Occurrence:** occurrenceDetails: http://www.boldsystems.org/index.php/API_Public/specimen?ids=BC-ZSM-HYM-21587-B10|BC-ZSM-HYM-22524-B07|BC-ZSM-HYM-20721-F07|BC-ZSM-HYM-21587-B03; catalogNumber: BC-ZSM-HYM-20721-F07; recordNumber: BC-ZSM-HYM-20721-F07; recordedBy: C. Hansson; individualID: BC-ZSM-HYM-20721-F07; individualCount: 1; sex: F; lifeStage: Adult; associatedMedia: http://www.boldsystems.org/pics/BCHYM/BC-ZSM-HYM-20721-F07+1398632230.jpg; **Taxon:** scientificName: Tetrastichus acutiusculus; phylum: Arthropoda; class: Insecta; order: Hymenoptera; family: Eulophidae; genus: Tetrastichus; **Location:** country: Sweden; locality: Klagshamn; decimalLatitude: 55.533; decimalLongitude: 12.917; **Identification:** identifiedBy: Christer Hansson; identificationRemarks: CH00225
- **Type status:** Other material. **Occurrence:** occurrenceDetails: http://www.boldsystems.org/index.php/API_Public/specimen?ids=BC-ZSM-HYM-21587-B10|BC-ZSM-HYM-22524-B07|BC-ZSM-HYM-20721-F07|BC-ZSM-HYM-21587-B03; catalogNumber: BC-ZSM-HYM-22524-B07; recordNumber: BC-ZSM-HYM-22524-B07; recordedBy: C. Hansson; individualID: BC-ZSM-HYM-22524-B07; individualCount: 1; sex: F; lifeStage: a; reproductiveCondition: S; **Taxon:** scientificName: Tetrastichus acutiusculus; phylum: Arthropoda; class: Insecta; order: Hymenoptera; family: Eulophidae; genus: Tetrastichus; **Location:** country: Hungary; locality: Koeszeg; decimalLatitude: 47.22; decimalLongitude: 16.313; **Identification:** identifiedBy: Christer Hansson; identificationRemarks: CH09270
- **Type status:** Other material. **Occurrence:** occurrenceDetails: http://www.boldsystems.org/index.php/API_Public/specimen?ids=BC-ZSM-HYM-21587-B10|BC-ZSM-HYM-22524-B07|BC-ZSM-HYM-20721-F07|BC-ZSM-HYM-21587-B03; catalogNumber: BC-ZSM-HYM-21587-B10; recordNumber: BC-ZSM-HYM-21587-B10; recordedBy: C.Hansson; individualID: BC-ZSM-HYM-21587-B10; individualCount: 1; sex: F; lifeStage: a; associatedMedia: http://www.boldsystems.org/pics/BCHYM/BC-ZSM-HYM-21587-B10+1423081070.jpg; **Taxon:** scientificName: Tetrastichus acutiusculus; phylum: Arthropoda; class: Insecta; order: Hymenoptera; family: Eulophidae; genus: Tetrastichus; **Location:** country: Sweden; locality: Bostorpsvaegen; decimalLatitude: 56.776; decimalLongitude: 16.707; **Identification:** identifiedBy: Christer Hansson
- **Type status:** Other material. **Occurrence:** occurrenceDetails: http://www.boldsystems.org/index.php/API_Public/specimen?ids=BC-ZSM-HYM-21587-B03; catalogNumber: BC-ZSM-HYM-21587-B03; recordNumber: BC-ZSM-HYM-21587-B03; recordedBy: C.Hansson; individualID: BC-ZSM-HYM-21587-B03; individualCount: 1; sex: F; lifeStage: a; associatedMedia: http://www.boldsystems.org/pics/BCHYM/BC-ZSM-HYM-21587-B03+1423081074.jpg; **Taxon:** scientificName: Tetrastichus acutiusculus; phylum: Arthropoda; class: Insecta; order: Hymenoptera; family: Eulophidae; genus: Tetrastichus; **Location:** country: Sweden; locality: Ismantorp; decimalLatitude: 56.893; decimalLongitude: 16.768; **Identification:** identifiedBy: Christer Hansson

#### Description

See Graham (1991). The male is unknown.

#### Diagnosis

Gaster 2× as long as wide with Gt_7_ 1× as long as width at base. Similar to *T. leocrates*, but with longer gaster and longer antenna, as mentioned in the key.

#### Distribution

United Kingdom (Graham 1991), Sweden (Hedqvist 2003), andHungary (**new record**).

#### Ecology

##### Host

Unknown.

##### Additional material examined

Type material: holotype ♀ (NHM, type no. 5.3625). Additional material: Hungary 1♀ (NHM), Sweden 5♀ (NHRS, MZLU, ZSM).

### Tetrastichus agonus sp. n.

F8388846-D85E-52E0-B610-DCEEAD747E9D

urn:lsid:zoobank.org:act:73F51106-D2ED-4D87-9D18-363B1D665065

#### Materials

- **Type status:** Holotype. **Occurrence:** occurrenceDetails: http://www.boldsystems.org/index.php/API_Public/specimen?ids=BC-ZSM-HYM-25460-E09; catalogNumber: BC-ZSM-HYM-25460-E09; recordNumber: BC-ZSM-HYM-25460-E09; recordedBy: C. Hansson; individualID: BC-ZSM-HYM-25460-E09; individualCount: 1; sex: female; lifeStage: adult; **Taxon:** scientificName: Tetrastichus agonus; phylum: Arthropoda; class: Insecta; order: Hymenoptera; family: Eulophidae; genus: Tetrastichinae; taxonRemarks: Holotype deposited in MZLU; **Location:** country: Sweden; decimalLatitude: 55.6983; decimalLongitude: 13.4994; **Record Level:** type: PhysicalObject; language: en; institutionCode: MZLU; basisOfRecord: PreservedSpecimen
- **Type status:** Paratype. **Occurrence:** occurrenceDetails: http://www.boldsystems.org/index.php/API_Public/specimen?ids=BC-ZSM-HYM-21587-C08; catalogNumber: BC-ZSM-HYM-21587-C08; recordNumber: BC-ZSM-HYM-21587-C08; recordedBy: C.Hansson; individualID: BC-ZSM-HYM-21587-C08; individualCount: 1; sex: female; lifeStage: adult; **Taxon:** scientificName: Tetrastichus agonus; phylum: Arthropoda; class: Insecta; order: Hymenoptera; family: Eulophidae; genus: Tetrastichinae; **Location:** country: Sweden; decimalLatitude: 55.84; decimalLongitude: 13.58; **Record Level:** type: PhysicalObject; language: en; basisOfRecord: PreservedSpecimen
- **Type status:** Paratype. **Occurrence:** occurrenceDetails: http://www.boldsystems.org/index.php/API_Public/specimen?ids=BC-ZSM-HYM-20721-F08; catalogNumber: BC-ZSM-HYM-20721-F08; recordNumber: BC-ZSM-HYM-20721-F08; recordedBy: C. Hansson; individualID: BC-ZSM-HYM-20721-F08; individualCount: 1; sex: male; lifeStage: adult; **Taxon:** scientificName: Tetrastichus agonus; phylum: Arthropoda; class: Insecta; order: Hymenoptera; family: Eulophidae; genus: Tetrastichinae; **Location:** country: Sweden; decimalLatitude: 55.533; decimalLongitude: 12.917; **Record Level:** type: PhysicalObject; language: en; basisOfRecord: PreservedSpecimen
- **Type status:** Paratype. **Occurrence:** occurrenceDetails: http://www.boldsystems.org/index.php/API_Public/specimen?ids=BC-ZSM-HYM-20721-E10; catalogNumber: BC-ZSM-HYM-20721-E10; recordNumber: BC-ZSM-HYM-20721-E10; recordedBy: C. Hansson; individualID: BC-ZSM-HYM-20721-E10; individualCount: 1; sex: female; lifeStage: adult; **Taxon:** scientificName: Tetrastichus agonus; phylum: Arthropoda; class: Insecta; order: Hymenoptera; family: Eulophidae; genus: Tetrastichinae; **Location:** country: Sweden; decimalLatitude: 55.7; decimalLongitude: 13.517; **Record Level:** type: PhysicalObject; language: en; basisOfRecord: PreservedSpecimen
- **Type status:** Paratype. **Occurrence:** occurrenceDetails: http://www.boldsystems.org/index.php/API_Public/specimen?ids=BC-ZSM-HYM-25460-G12; catalogNumber: BC-ZSM-HYM-25460-G12; recordNumber: BC-ZSM-HYM-25460-G12; recordedBy: C. Hansson; individualID: BC-ZSM-HYM-25460-G12; individualCount: 1; sex: female; lifeStage: adult; **Taxon:** scientificName: Tetrastichus agonus; phylum: Arthropoda; class: Insecta; order: Hymenoptera; family: Eulophidae; genus: Tetrastichinae; **Location:** country: Sweden; decimalLatitude: 55.6967; decimalLongitude: 13.4408; **Record Level:** type: PhysicalObject; language: en; basisOfRecord: PreservedSpecimen
- **Type status:** Paratype. **Occurrence:** occurrenceDetails: http://www.boldsystems.org/index.php/API_Public/specimen?ids=BC-ZSM-HYM-25460-F04; catalogNumber: BC-ZSM-HYM-25460-F04; recordNumber: BC-ZSM-HYM-25460-F04; recordedBy: C. Hansson; individualID: BC-ZSM-HYM-25460-F04; individualCount: 1; sex: female; lifeStage: adult; **Taxon:** scientificName: Tetrastichus agonus; phylum: Arthropoda; class: Insecta; order: Hymenoptera; family: Eulophidae; genus: Tetrastichinae; **Location:** country: Sweden; decimalLatitude: 55.6983; decimalLongitude: 13.4994; **Record Level:** type: PhysicalObject; language: en; basisOfRecord: PreservedSpecimen
- **Type status:** Paratype. **Occurrence:** occurrenceDetails: http://www.boldsystems.org/index.php/API_Public/specimen?ids=BC-ZSM-HYM-25460-E11; catalogNumber: BC-ZSM-HYM-25460-E11; recordNumber: BC-ZSM-HYM-25460-E11; recordedBy: C. Hansson; individualID: BC-ZSM-HYM-25460-E11; individualCount: 1; sex: female; lifeStage: adult; **Taxon:** scientificName: Tetrastichus agonus; phylum: Arthropoda; class: Insecta; order: Hymenoptera; family: Eulophidae; genus: Tetrastichinae; **Location:** country: Sweden; decimalLatitude: 55.6983; decimalLongitude: 13.4994; **Record Level:** type: PhysicalObject; language: en; basisOfRecord: PreservedSpecimen
- **Type status:** Paratype. **Occurrence:** occurrenceDetails: http://www.boldsystems.org/index.php/API_Public/specimen?ids=BC-ZSM-HYM-25460-E04; catalogNumber: BC-ZSM-HYM-25460-E04; recordNumber: BC-ZSM-HYM-25460-E04; recordedBy: C. Hansson; individualID: BC-ZSM-HYM-25460-E04; individualCount: 1; sex: female; lifeStage: adult; **Taxon:** scientificName: Tetrastichus agonus; phylum: Arthropoda; class: Insecta; order: Hymenoptera; family: Eulophidae; genus: Tetrastichinae; **Location:** country: Sweden; decimalLatitude: 55.6983; decimalLongitude: 13.4994; **Record Level:** type: PhysicalObject; language: en; basisOfRecord: PreservedSpecimen
- **Type status:** Paratype. **Occurrence:** occurrenceDetails: http://www.boldsystems.org/index.php/API_Public/specimen?ids=BC-ZSM-HYM-25460-D05; catalogNumber: BC-ZSM-HYM-25460-D05; recordNumber: BC-ZSM-HYM-25460-D05; recordedBy: C. Hansson; individualID: BC-ZSM-HYM-25460-D05; individualCount: 1; sex: female; lifeStage: adult; **Taxon:** scientificName: Tetrastichus agonus; phylum: Arthropoda; class: Insecta; order: Hymenoptera; family: Eulophidae; genus: Tetrastichinae; **Location:** country: Sweden; decimalLatitude: 55.6983; decimalLongitude: 13.4994; **Record Level:** type: PhysicalObject; language: en; basisOfRecord: PreservedSpecimen
- **Type status:** Paratype. **Occurrence:** occurrenceDetails: http://www.boldsystems.org/index.php/API_Public/specimen?ids=BC-ZSM-HYM-25460-E06; catalogNumber: BC-ZSM-HYM-25460-E06; recordNumber: BC-ZSM-HYM-25460-E06; recordedBy: C. Hansson; individualID: BC-ZSM-HYM-25460-E06; individualCount: 1; sex: F; lifeStage: a; reproductiveCondition: S; associatedMedia: http://www.boldsystems.org/pics/BCHYM/BC-ZSM-HYM-25460-E06+1449268384.jpg; **Taxon:** scientificName: Tetrastichus agonus; phylum: Arthropoda; class: Insecta; order: Hymenoptera; family: Eulophidae; genus: Tetrastichus; **Location:** country: Sweden; locality: Krankesjoen, Fiskeplats; decimalLatitude: 55.6983; decimalLongitude: 13.4994; **Identification:** identifiedBy: Christer Hansson

#### Description

FEMALE holotype (Fig. 55). Body length 2.0 mm (paratypes 1.6–2.0 mm). *Head*. Width/length in dorsal view 2.5, width/length in frontal view 1.2, POL/OOL 1.7, widths head/mesosoma 1.0, mouth width/malar space 0.9, malar space/eye height 0.9. *Antenna*. Scape length/eye height 0.9, pedicel+flagellum length/mesosoma width 1.2, length/width F1, F2, F3 2.3, 2.0, 2.0, clava length/width 3.3, lengths pedicel/F1 0.6, lengths F1/F2 1.1, F1/F3 1.1, lengths F1, F2, F3/clava 0.6, 0.6, 0.6, widths F1/pedicel (dorsal view) 1.1, lengths antennal spicule/C3 0.2. *Mesosoma*. Length/width 1.4, mesoscutal mid-lobe length/width 1.0 (width measured in anterior part), mid-lobe with a median groove in posterior ½, with six adnotaular setae on each side, lengths mesoscutum/mesoscutellum (measured medially) 1.4, mesoscutellum length/width 0.8, length/width of enclosed space between submedian grooves 2.5, distance between SMG/distance between SMG and SLG 1.4, lengths dorsellum/propodeum (measured medially) 0.7, propodeal callus with six setae. *Fore wing*. Costal cell length/width 7.4, lengths costal cell/marginal vein 0.9, lengths marginal/stigmal veins 3.3. *Gaster*. Elongate-acuminate, length/width 1.4, lengths gaster/mesosoma 0.9, Gt_7_ length/width 0.5, length of longest cercal seta/next longest seta 1.6, longest cercal seta curved, ovipositor sheaths reach apex of Gt_7_, but not beyond.

Colour. Head and mesosoma with golden tinges, gaster metallic blue, entire antenna dark brown, tegulae black, wings hyaline with venation yellowish-brown, coxae and femora golden-green, trochanters black, tibiae yellowish-brown, tarsi yellowish-brown with T4 brown.

MALE. Body length 1.7 mm. *Head.* Width/length in dorsal view 2.5, width/length in frontal view 1.3, eye height/malar space 1.3, mouth width/malar space 1.0, widths head/mesosoma 1.1. *Antenna.* F1–F4 with basal whorls of setae, reaching beyond apex of corresponding flagellomere, whorled setae on F1 1.4× as long as F1 length, scape length/eye height 0.9, scape length/width 2.4, ventral plaque placed centrally, lengths ventral plaque/scape 0.7, pedicel+flagellum length/mesosoma width 1.8, length/width F1, F2, F3, F4 1.5, 1.9, 2.0, 2.0, clava length/width 5.0, lengths pedicel/F1 2.4, lengths F1/F2 0.8, F1/F3 0.8, F1/F4 0.8, lengths F1, F2, F3 F4 /clava 0.3, 0.4, 0.4, 0.4.

Colour. Entire body with golden-green tinges, mid and hind tibiae dark brown. Otherwise as in female.

#### Diagnosis

Very similar to *T. miser*, but the male differs in having whorled setae of F1–F4 reaching slightly beyond the tip of funicular attached to; female tentatively separated through characters mentioned in the key.

#### Distribution

Sweden.

#### Ecology

##### Host

Unknown.

#### Notes

Holotype deposited in MZLU, paratypes in MZLU, NHM, ZSM.

### Tetrastichus agrilocidus

Graham, 1991

7BFAA0A3-4BD7-5DCE-BCAD-D8EC28B698B0

Tetrastichus agrilocidus Graham 1991:250. Holotype ♀ in NHM, examined (Fig. 56).

#### Description

See Graham (1991).

#### Diagnosis

Female antenna with clava 0.7–0.8× as long as F2+F3, F1 about as wide as pedicel; male antenna with whorled setae of funiculars reaching beyond the tips of funicular attached to, scape 2.5× as long as wide; female gaster 2.8× as long as wide.

#### Distribution

(Former) Czechoslovakia, Hungary, Poland (Graham 1991), Sweden (Hedqvist 2003), Finland, France and Germany (**new records**).

#### Ecology

##### Host

*Agrilus* sp. (Coleoptera: Buprestidae), *Xylotrechus pantherinus* Savenius (Coleoptera: Cerambycidae) (Graham 1991).

#### Material examined

Type material: holotype ♀ of *T. agrilocidus* (NHM, type no. 5.3620). Additional material (11♀ 2♂): Czech Republic 1♀ (NHM), Finland 2♀ (NHM), France 6♀ (NHM), Germany 2♀ 1♂ (NHM, ZSM), Sweden 4♀ 1♂ (MZLU).

### Tetrastichus ballotus sp. n.

0E120561-A1D4-5637-9F20-678419169929

urn:lsid:zoobank.org:act:52C31218-ADD7-474A-97D7-A7B03ABCEE32

#### Materials

- **Type status:** Holotype. **Occurrence:** occurrenceDetails: http://www.boldsystems.org/index.php/API_Public/specimen?ids=BC-ZSM-HYM-26563-A07; catalogNumber: BC-ZSM-HYM-26563-A07; recordNumber: BC-ZSM-HYM-26563-A07; recordedBy: C. Hansson; individualID: BC-ZSM-HYM-26563-A07; individualCount: 1; sex: female; lifeStage: adult; **Taxon:** scientificName: Tetrastichus ballotus; phylum: Arthropoda; class: Insecta; order: Hymenoptera; family: Eulophidae; genus: Tetrastichinae; taxonRemarks: Holotype deposited in MZLU; **Location:** country: Sweden; decimalLatitude: 55.6967; decimalLongitude: 13.4408; **Record Level:** type: PhysicalObject; language: en; institutionCode: MZLU; basisOfRecord: PreservedSpecimen
- **Type status:** Paratype. **Occurrence:** occurrenceDetails: http://www.boldsystems.org/index.php/API_Public/specimen?ids=BC-ZSM-HYM-26563-A11; catalogNumber: BC-ZSM-HYM-26563-A11; recordNumber: BC-ZSM-HYM-26563-A11; recordedBy: C. Hansson; individualID: BC-ZSM-HYM-26563-A11; individualCount: 1; sex: female; lifeStage: adult; **Taxon:** scientificName: Tetrastichus ballotus; phylum: Arthropoda; class: Insecta; order: Hymenoptera; family: Eulophidae; genus: Tetrastichinae; **Location:** country: Sweden; decimalLatitude: 55.6664; decimalLongitude: 13.6242; **Record Level:** type: PhysicalObject; language: en; basisOfRecord: PreservedSpecimen
- **Type status:** Paratype. **Occurrence:** occurrenceDetails: http://www.boldsystems.org/index.php/API_Public/specimen?ids=BC-ZSM-HYM-13565-C04; catalogNumber: BC-ZSM-HYM-13565-C04; recordNumber: BC-ZSM-HYM-13565-C04; recordedBy: SMTP project; individualID: BC-ZSM-HYM-13565-C04; individualCount: 1; sex: female; lifeStage: adult; **Taxon:** scientificName: Tetrastichus ballotus; phylum: Arthropoda; class: Insecta; order: Hymenoptera; family: Eulophidae; genus: Tetrastichinae; **Location:** country: Sweden; decimalLatitude: 56.418; decimalLongitude: 16.068; **Record Level:** type: PhysicalObject; language: en; institutionCode: SMTP; basisOfRecord: PreservedSpecimen
- **Type status:** Paratype. **Occurrence:** occurrenceDetails: http://www.boldsystems.org/index.php/API_Public/specimen?ids=BC-ZSM-HYM-13565-C03; catalogNumber: BC-ZSM-HYM-13565-C03; recordNumber: BC-ZSM-HYM-13565-C03; recordedBy: SMTP project; individualID: BC-ZSM-HYM-13565-C03; individualCount: 1; sex: female; lifeStage: adult; **Taxon:** scientificName: Tetrastichus ballotus; phylum: Arthropoda; class: Insecta; order: Hymenoptera; family: Eulophidae; genus: Tetrastichinae; **Location:** country: Sweden; decimalLatitude: 58.553; decimalLongitude: 11.273; **Record Level:** type: PhysicalObject; language: en; basisOfRecord: PreservedSpecimen
- **Type status:** Paratype. **Occurrence:** occurrenceDetails: http://www.boldsystems.org/index.php/API_Public/specimen?ids=BC-ZSM-HYM-13565-C02; catalogNumber: BC-ZSM-HYM-13565-C02; recordNumber: BC-ZSM-HYM-13565-C02; recordedBy: SMTP; individualID: BC-ZSM-HYM-13565-C02; individualCount: 1; sex: female; lifeStage: adult; **Taxon:** scientificName: Tetrastichus ballotus; phylum: Arthropoda; class: Insecta; order: Hymenoptera; family: Eulophidae; genus: Tetrastichinae; **Location:** decimalLatitude: 58.553; decimalLongitude: 11.273; **Record Level:** type: PhysicalObject; language: en; basisOfRecord: PreservedSpecimen
- **Type status:** Paratype. **Occurrence:** occurrenceDetails: http://www.boldsystems.org/index.php/API_Public/specimen?ids=BC-ZSM-HYM-13565-C05; catalogNumber: BC-ZSM-HYM-13565-C05; recordNumber: BC-ZSM-HYM-13565-C05; recordedBy: SMTP; individualID: BC-ZSM-HYM-13565-C05; individualCount: 1; sex: female; lifeStage: adult; **Taxon:** scientificName: Tetrastichus ballotus; phylum: Arthropoda; class: Insecta; order: Hymenoptera; family: Eulophidae; genus: Tetrastichinae; **Location:** decimalLatitude: 56.418; decimalLongitude: 16.068; **Record Level:** type: PhysicalObject; language: en; institutionCode: SMTP; basisOfRecord: PreservedSpecimen
- **Type status:** Paratype. **Occurrence:** occurrenceDetails: http://www.boldsystems.org/index.php/API_Public/specimen?ids=BC-ZSM-HYM-29750-H03; catalogNumber: BC-ZSM-HYM-29750-H03; recordNumber: BC-ZSM-HYM-29750-H03; recordedBy: C. Hansson; individualID: BC-ZSM-HYM-29750-H03; individualCount: 1; sex: F; lifeStage: a; associatedMedia: http://www.boldsystems.org/pics/BCHYM/BC-ZSM-HYM-29750-H03+1510087798.jpg; **Taxon:** scientificName: Tetrastichus ballotus; phylum: Arthropoda; class: Insecta; order: Hymenoptera; family: Eulophidae; genus: Tetrastichus; **Location:** country: Sweden; locality: Lake Kranke, vid baeck; decimalLatitude: 55.7078; decimalLongitude: 13.475; **Identification:** identifiedBy: Christer Hansson
- **Type status:** Paratype. **Occurrence:** occurrenceDetails: http://www.boldsystems.org/index.php/API_Public/specimen?ids=BC-ZSM-HYM-13565-C01; catalogNumber: BC-ZSM-HYM-13565-C01; recordNumber: BC-ZSM-HYM-13565-C01; recordedBy: SMTr Project; individualID: BC-ZSM-HYM-13565-C01; individualCount: 1; lifeStage: a; reproductiveCondition: S; associatedMedia: http://www.boldsystems.org/pics/BCHYM/BC-ZSM-HYM-13565-C01+1444992588.jpg; **Taxon:** scientificName: Tetrastichus ballotus; phylum: Arthropoda; class: Insecta; order: Hymenoptera; family: Eulophidae; genus: Tetrastichus; **Location:** country: Sweden; locality: Tanums kn, Hamburgsund; decimalLatitude: 58.553; decimalLongitude: 11.273; **Identification:** identifiedBy: Christer Hansson
- **Type status:** Paratype. **Occurrence:** occurrenceDetails: http://www.boldsystems.org/index.php/API_Public/specimen?ids=BC-ZSM-HYM-20721-D11; catalogNumber: BC-ZSM-HYM-20721-D11; recordNumber: BC-ZSM-HYM-20721-D11; recordedBy: C. Hansson; individualID: BC-ZSM-HYM-20721-D11; individualCount: 1; sex: F; lifeStage: Adult; associatedMedia: http://www.boldsystems.org/pics/BCHYM/BC-ZSM-HYM-20721-D11+1398631912.jpg; **Taxon:** scientificName: Tetrastichus ballotus; phylum: Arthropoda; class: Insecta; order: Hymenoptera; family: Eulophidae; genus: Tetrastichus; **Location:** country: Sweden; locality: Lk Kranke, Ekskogen; decimalLatitude: 55.683; decimalLongitude: 13.45; **Identification:** identifiedBy: Christer Hansson; identificationRemarks: CH04246
- **Type status:** Paratype. **Occurrence:** occurrenceDetails: http://www.boldsystems.org/index.php/API_Public/specimen?ids=BC-ZSM-HYM-29750-F02; catalogNumber: BC-ZSM-HYM-29750-F02; recordNumber: BC-ZSM-HYM-29750-F02; recordedBy: C. Hansson; individualID: BC-ZSM-HYM-29750-F02; individualCount: 1; sex: F; lifeStage: a; associatedMedia: http://www.boldsystems.org/pics/BCHYM/BC-ZSM-HYM-29750-F02+1510087672.jpg; **Taxon:** scientificName: Tetrastichus ballotus; phylum: Arthropoda; class: Insecta; order: Hymenoptera; family: Eulophidae; genus: Tetrastichus; **Location:** country: Sweden; locality: Lake Kranke, Ekskogen; decimalLatitude: 55.6861; decimalLongitude: 13.4611; **Identification:** identifiedBy: Christer Hansson
- **Type status:** Paratype. **Occurrence:** occurrenceDetails: http://www.boldsystems.org/index.php/API_Public/specimen?ids=BC-ZSM-HYM-26563-C07; catalogNumber: BC-ZSM-HYM-26563-C07; recordNumber: BC-ZSM-HYM-26563-C07; recordedBy: C. Hansson; individualID: BC-ZSM-HYM-26563-C07; individualCount: 1; sex: F; lifeStage: a; associatedMedia: http://www.boldsystems.org/pics/BCHYM/BC-ZSM-HYM-26563-C07+1510087182.jpg; **Taxon:** scientificName: Tetrastichus ballotus; phylum: Arthropoda; class: Insecta; order: Hymenoptera; family: Eulophidae; genus: Tetrastichus; **Location:** country: Sweden; locality: Lake Kranke, baeck; decimalLatitude: 55.7078; decimalLongitude: 13.475; **Identification:** identifiedBy: Christer Hansson

#### Description

FEMALE holotype (Fig. 57). Body length 1.9 mm (paratypes 1.4–2.0 mm). *Head*. Width/length in dorsal view 2.4, width/length in frontal view 1.3, POL/OOL 2.2, widths head/mesosoma 1.0, mouth width/malar space 1.0, malar space/eye height 0.8. *Antenna*. Scape length/eye height 0.9, pedicel+flagellum length/mesosoma width 1.2, length/width F1, F2, F3 2.2, 1.9, 1.7, clava length/width 3.6, lengths pedicel/F1 0.7, lengths F1/F2 1.1, F1/F3 1.1, lengths F1, F2, F3/clava 0.6, 0.5, 0.5, widths F1/pedicel (dorsal view) 1.1, lengths antennal spicule/C3 0.2. *Mesosoma*. Length/width 1.4, mesoscutal mid-lobe length/width 1.0 (width measured in anterior part), mid-lobe with a median groove in posterior ⅔, with four adnotaular setae on each side, lengths mesoscutum/mesoscutellum (measured medially) 1.3, mesoscutellum length/width 0.9, length/width of enclosed space between submedian grooves 2.5, distance between SMG/distance between SMG and SLG 1.7, lengths dorsellum/propodeum (measured medially) 0.7, propodeal callus with four setae. *Fore wing*. Costal cell length/width 9.1, lengths costal cell/marginal vein 0.9, lengths marginal/stigmal veins 3.3. *Gaster*. Ovate, length/width 1.4, lengths gaster/mesosoma 1.0, Gt_7_ length/width 0.5, length of longest cercal seta/next longest seta 1.6, longest cercal seta curved, ovipositor sheaths projecting slightly beyond apex of Gt_7_.

Colour. Body with golden-green tinges, entire antenna dark brown, tegulae black, wings hyaline with venation dark yellowish-brown, coxae and femora concolorous with body, trochanters black, fore tibia dark yellowish-brown, mid and hind tibiae pale brown, fore tarsus dark brown, mid and hind tarsi yellowish-brown with T3-4 dark brown.

MALE. Unknown.

#### Diagnosis

Very similar to *T. miser*, differs morphologically in having a longer distance between lateral ocelli and eyes and in having a longer antennal clava.

#### Distribution

Sweden.

#### Ecology

##### Host

Unknown.

#### Notes

Holotype deposited in MZLU, paratypes in MZLU, SMTP.

### Tetrastichus broncus sp. n.

6DE5E073-7834-52B9-BF8B-37CD7E13029D

urn:lsid:zoobank.org:act:18B532FE-61A6-4E1D-B5E9-E189A5042D20

#### Materials

- **Type status:** Holotype. **Occurrence:** occurrenceDetails: http://www.boldsystems.org/index.php/API_Public/specimen?ids=BC-ZSM-HYM-21587-G03; catalogNumber: BC-ZSM-HYM-21587-G03; recordNumber: BC-ZSM-HYM-21587-G03; recordedBy: C.Hansson; individualID: BC-ZSM-HYM-21587-G03; individualCount: 1; sex: female; lifeStage: adult; **Taxon:** scientificName: Tetrastichus broncus; phylum: Arthropoda; class: Insecta; order: Hymenoptera; family: Eulophidae; genus: Tetrastichinae; taxonRemarks: Holotype deposited in MZLU; **Location:** country: Sweden; decimalLatitude: 55.822; decimalLongitude: 13.81; **Record Level:** type: PhysicalObject; language: en; institutionCode: MZLU; basisOfRecord: PreservedSpecimen
- **Type status:** Paratype. **Occurrence:** occurrenceDetails: http://www.boldsystems.org/index.php/API_Public/specimen?ids=BC-ZSM-HYM-22523-B04; catalogNumber: BC-ZSM-HYM-22523-B04; recordNumber: BC-ZSM-HYM-22523-B04; recordedBy: Swedish Malaise Trap Project; individualID: BC-ZSM-HYM-22523-B04; individualCount: 1; sex: female; lifeStage: adult; **Taxon:** scientificName: Tetrastichus broncus; phylum: Arthropoda; class: Insecta; order: Hymenoptera; family: Eulophidae; genus: Tetrastichinae; **Location:** country: Sweden; decimalLatitude: 56.016; decimalLongitude: 13.134; **Record Level:** type: PhysicalObject; language: en; basisOfRecord: PreservedSpecimen
- **Type status:** Paratype. **Occurrence:** occurrenceDetails: http://www.boldsystems.org/index.php/API_Public/specimen?ids=BC-ZSM-HYM-22523-B03; catalogNumber: BC-ZSM-HYM-22523-B03; recordNumber: BC-ZSM-HYM-22523-B03; recordedBy: Swedish Malaise Trap Project; individualID: BC-ZSM-HYM-22523-B03; individualCount: 1; sex: female; lifeStage: adult; **Taxon:** scientificName: Tetrastichus broncus; phylum: Arthropoda; class: Insecta; order: Hymenoptera; family: Eulophidae; genus: Tetrastichinae; **Location:** country: Sweden; decimalLatitude: 56.016; decimalLongitude: 13.134; **Record Level:** type: PhysicalObject; language: en; basisOfRecord: PreservedSpecimen
- **Type status:** Paratype. **Occurrence:** occurrenceDetails: http://www.boldsystems.org/index.php/API_Public/specimen?ids=BC-ZSM-HYM-26563-D12; catalogNumber: BC-ZSM-HYM-26563-D12; recordNumber: BC-ZSM-HYM-26563-D12; recordedBy: C. Hansson; individualID: BC-ZSM-HYM-26563-D12; individualCount: 1; sex: female; lifeStage: adult; **Taxon:** scientificName: Tetrastichus broncus; phylum: Arthropoda; class: Insecta; order: Hymenoptera; family: Eulophidae; genus: Tetrastichinae; **Location:** country: Sweden; decimalLatitude: 55.6619; decimalLongitude: 13.5472; **Record Level:** type: PhysicalObject; language: en; basisOfRecord: PreservedSpecimen
- **Type status:** Paratype. **Occurrence:** occurrenceDetails: http://www.boldsystems.org/index.php/API_Public/specimen?ids=BC-ZSM-HYM-22526-G10; catalogNumber: BC-ZSM-HYM-22526-G10; recordNumber: BC-ZSM-HYM-22526-G10; recordedBy: S. Schmidt; individualID: BC-ZSM-HYM-22526-G10; individualCount: 1; sex: male; lifeStage: adult; **Taxon:** scientificName: Tetrastichus broncus; phylum: Arthropoda; class: Insecta; order: Hymenoptera; family: Eulophidae; genus: Tetrastichinae; **Location:** country: Sweden; decimalLatitude: 56.683; decimalLongitude: 16.641; **Record Level:** type: PhysicalObject; language: en; basisOfRecord: PreservedSpecimen
- **Type status:** Paratype. **Occurrence:** occurrenceDetails: http://www.boldsystems.org/index.php/API_Public/specimen?ids=BC-ZSM-HYM-21587-D04; catalogNumber: BC-ZSM-HYM-21587-D04; recordNumber: BC-ZSM-HYM-21587-D04; recordedBy: C.Hansson; individualID: BC-ZSM-HYM-21587-D04; individualCount: 1; sex: female; lifeStage: adult; **Taxon:** scientificName: Tetrastichus broncus; phylum: Arthropoda; class: Insecta; order: Hymenoptera; family: Eulophidae; genus: Tetrastichinae; **Location:** country: Sweden; decimalLatitude: 55.683; decimalLongitude: 13.517; **Record Level:** type: PhysicalObject; language: en; basisOfRecord: PreservedSpecimen
- **Type status:** Paratype. **Occurrence:** occurrenceDetails: http://www.boldsystems.org/index.php/API_Public/specimen?ids=BC-ZSM-HYM-27770-A02; catalogNumber: BC-ZSM-HYM-27760-A02; recordNumber: BC-ZSM-HYM-27760-A02; recordedBy: R. Bygebjerg; individualID: BC-ZSM-HYM-27770-A02; individualCount: 1; sex: female; lifeStage: adult; **Taxon:** scientificName: Tetrastichus broncus; phylum: Arthropoda; class: Insecta; order: Hymenoptera; family: Eulophidae; genus: Tetrastichinae; **Location:** country: Sweden; decimalLatitude: 55.6192; decimalLongitude: 13.5497; **Record Level:** type: PhysicalObject; language: en; basisOfRecord: PreservedSpecimen
- **Type status:** Paratype. **Occurrence:** occurrenceDetails: http://www.boldsystems.org/index.php/API_Public/specimen?ids=BC-ZSM-HYM-29750-B05; catalogNumber: BC-ZSM-HYM-29750-B05; recordNumber: BC-ZSM-HYM-29750-B05; recordedBy: C. Hansson; individualID: BC-ZSM-HYM-29750-B05; individualCount: 1; sex: female; lifeStage: adult; **Taxon:** scientificName: Tetrastichus broncus; phylum: Arthropoda; class: Insecta; order: Hymenoptera; family: Eulophidae; genus: Tetrastichinae; **Location:** country: Sweden; decimalLatitude: 55.0756; decimalLongitude: 13.6778; **Record Level:** type: PhysicalObject; language: en; basisOfRecord: PreservedSpecimen

#### Description

FEMALE holotype (Fig. 58). Body length 2.1 mm (paratypes 2.2–2.6 mm). *Head*. Width/length (dorsal view) 2.1, width/length (frontal view) 1.3, POL/OOL 1.9, widths head/mesosoma 1.1, mouth width/malar space 1.0, malar space/eye height 0.8. *Antenna*. Scape length/eye height 0.9, pedicel+flagellum length/mesosoma width 1.4, length/width F1, F2, F3 2.0, 1.9, 1.9, clava length/width 3.5, lengths pedicel/F1 0.6, lengths F1/F2 1.1, F1/F3 1.1, lengths F1, F2, F3/clava 0.5, 0.5, 0.5, widths F1/pedicel (dorsal view) 1.3, lengths antennal spicule/C3 0.3. *Mesosoma*. Length/width 1.4, mesoscutal mid-lobe length/width (width measured in anterior part) 1.0, mid-lobe with a median groove in posterior ½, with five adnotaular setae on each side, lengths mesoscutum/mesoscutellum (measured medially) 1.4, mesoscutellum length/width 0.8, length/width of enclosed space between submedian grooves 2.4, distance between SMG/distance between SMG and SLG 1.3, lengths dorsellum/propodeum (measured medially) 0.6, propodeum with strong reticulation, propodeal callus with five setae. *Fore wing*. Costal cell length/width 13.6, lengths costal cell/marginal vein 0.9, lengths marginal/stigmal veins 3.8. *Gaster*. Elongate-acuminate, length/width 2.6, lengths gaster/head+mesosoma 1.1, Gt_7_ length/width 1.3, length of longest cercal seta/next longest seta 1.8, longest cercal seta almost straight, ovipositor sheaths projecting beyond apex of Gt_7_.

Colour. Body with weak golden-green tinges, scape yellowish-brown, pedicel and flagellum dark brown, tegulae dark brown, wings hyaline with veins yellowish-white, coxae and femora concolorous with body, trochanters dark brown, tibiae and tarsi yellowish-brown.

MALE. Unknown.

#### Diagnosis

Gaster elongate-acuminate, length/width 2.6, lengths gaster/head+mesosoma 1.1, Gt_7_ length/width 1.3; antennal clava 1.0× as long as F2+F3 and with distinct constriction between C1 and C2.

#### Distribution

Sweden.

#### Ecology

##### Host

Unknown.

#### Notes

Holotype deposited in MZLU, paratypes in MZLU, SMTP and ZSM.

### Tetrastichus crioceridis

Graham, 1983

E6AB440A-9E96-52D2-83C4-642F6F8C789C

Tetrastichus crioceridis Graham 1983:275–277. Holotype ♀ in Naturalis Biodiversity Center, Leiden, The Netherlands, not examined. Fig. 59

#### Description

See Graham (1983).

#### Diagnosis

Mouth opening 1.4–1.6× malar space in both sexes; apex of female antennal clava blunt; male funiculars 2–4 without distinct whorl of setae.

#### Distribution

France, The Netherlands (Graham 1983), Sweden (Hedqvist 2003) and United Kingdom (**new record**).

#### Ecology

##### Host

*Crioceris duodecimpunctata* L. (Coleoptera: Chrysomelidae), gregarious egg-larval parasitoid (Van Alphen 1980).

##### Material examined

Type material: paratype(s) ♀ & ♂ of *T. crioceridis* (NHM). Additional material (118♀ 26♂): France 5♀ 1♂ (NHM), The Netherlands 20♀ 8♂ (NHM), Sweden 92♀ 17♂ (all ex *C. duodecimpunctata*) (MZLU, ZSM), United Kingdom 1♀ (NHM).

### Tetrastichus delvarei sp. n.

613FC571-05D7-55BB-B19E-742BADB4A77D

urn:lsid:zoobank.org:act:97131D3D-B98D-4079-B914-0E49A45AE920

#### Materials

- **Type status:** Holotype. **Occurrence:** occurrenceDetails: http://www.boldsystems.org/index.php/API_Public/specimen?ids=BC-ZSM-HYM-29813-E02; catalogNumber: BC-ZSM-HYM-29813-E02; recordNumber: BC-ZSM-HYM-29813-E02; recordedBy: G.Delvare; individualID: BC-ZSM-HYM-29813-E02; individualCount: 1; sex: female; lifeStage: adult; **Taxon:** scientificName: Tetrastichus delvarei; phylum: Arthropoda; class: Insecta; order: Hymenoptera; family: Eulophidae; genus: Tetrastichinae; taxonRemarks: Holotype deposited in NHM; **Location:** country: France; decimalLatitude: 44.124; decimalLongitude: 5.18037; **Record Level:** type: PhysicalObject; language: en; institutionCode: NHM; basisOfRecord: PreservedSpecimen
- **Type status:** Paratype. **Occurrence:** occurrenceDetails: http://www.boldsystems.org/index.php/API_Public/specimen?ids=BC-ZSM-HYM-25459-G08; catalogNumber: BC-ZSM-HYM-25459-G08; recordNumber: BC-ZSM-HYM-25459-G08; recordedBy: D. Doczkal; individualID: BC-ZSM-HYM-25459-G08; individualCount: 1; sex: female; lifeStage: adult; **Taxon:** scientificName: Tetrastichus delvarei; phylum: Arthropoda; class: Insecta; order: Hymenoptera; family: Eulophidae; genus: Tetrastichinae; **Location:** country: Germany; decimalLatitude: 50.027; decimalLongitude: 9.8; **Record Level:** type: PhysicalObject; language: en; institutionCode: ZSM; basisOfRecord: PreservedSpecimen

#### Description

FEMALE holotype (Fig. 60). Body length 2.1 mm. *Head.* Width/length in dorsal view 2.4, width/length in frontal view 1.2, POL/OOL 1.8, widths head/mesosoma 1.1, mouth width/malar space 1.1, malar space/eye height 0.7. *Antenna.* Scape length/eye height 0.8, pedicel+flagellum length/mesosoma width 1.3, length/width F1, F2, F3 2.6, 2.6, 1.9, clava length/width 3.4, lengths pedicel/F1 0.5, lengths F1/F2 1.0, F1/F3 1.2, lengths F1, F2, F3/clava 0.6, 0.6, 0.5, width F1/pedicel (dorsal view) 1.1, lengths antennal spicule/C3 0.2. *Mesosoma.* Length/width 1.5, mesoscutal mid-lobe length/width 0.9 (width measured in anterior part), mid-lobe with median groove almost complete, missing in very anterior part, with four adnotaular setae on each side, lengths mesoscutum/mesoscutellum (measured medially) 1.2, lengths dorsellum/propodeum 0.7, mesoscutellum length/width 0.9, length/width of enclosed space between submedian grooves 2.4, distance between SMG/distance between SMG and SLG 1.6, propodeum with strong reticulation, propodeal callus with six setae. *Fore wing.* Costal cell length/width 10.3, lengths costal cell/marginal vein 0.9, lengths marginal/stigmal veins 3.2. *Gaster.* Elongate, length/width 2.3, lengths gaster/mesosoma 1.4, Gt_7_ length/width 1.0, lengths longest cercal seta/next longest seta nm, ovipositor sheaths reaching beyond apex of Gt_7_.

Colour. Body with weak metallic bluish-green tinges, scape pale brown with dorsal edge dark brown, pedicel and flagellum dark brown, tegulae dark brown, wing venation yellowish-white, coxae concolorous with body, trochanters dark brown, fore and mid femora dark brown with apex yellowish-brown, hind femur concolorous with body, fore tibia yellowish-brown, mid tibia pale brown with apex yellowish-brown, hind tibia dark brown with apex yellowish-brown, tarsi with T1–3 yellowish-brown and T4 brown.

MALE. Unknown.

#### Diagnosis

Similar to *T. agrilocidus*, but differs in having a shorter gaster, 1.4× as long as length of mesosoma and with antennal flagellomeres shorter and clava longer.

#### Etymology

Named after the collector of holotype, Gerard Delvare (CIRAD).

#### Distribution

France.

#### Ecology

##### Host

Unknown.

#### Notes

Holotype deposited in NHM, paratype in ZSM.

### Tetrastichus doczkali sp. n.

158B98F6-0B60-5E44-8B7E-389D8B2097E3

urn:lsid:zoobank.org:act:F17802D2-E6F9-4D9E-97E7-D1513D165003

#### Materials

- **Type status:** Holotype. **Occurrence:** occurrenceDetails: http://www.boldsystems.org/index.php/API_Public/specimen?ids=BC-ZSM-HYM-21585-G01; catalogNumber: BC-ZSM-HYM-21585-G01; recordNumber: BC-ZSM-HYM-21585-G01; recordedBy: D. Doczkal; individualID: BC-ZSM-HYM-21585-G01; individualCount: 1; sex: female; lifeStage: adult; **Taxon:** scientificName: Tetrastichus doczkali; phylum: Arthropoda; class: Insecta; order: Hymenoptera; family: Eulophidae; genus: Tetrastichinae; taxonRemarks: Holotype deposited in ZSM; **Location:** country: Germany; decimalLatitude: 47.554; decimalLongitude: 7.67; **Record Level:** type: PhysicalObject; language: en; institutionCode: ZSM; basisOfRecord: PreservedSpecimen

#### Description

FEMALE holotype (Fig. 61). Body length 2.5 mm. *Head.* Width/length in dorsal view 2.3, width/length in frontal view 1.3, POL/OOL 2.3, widths head/mesosoma 1.0, mouth width/malar space 1.1, malar space/eye height 0.7. *Antenna.* Scape length/eye height 0.9, pedicel+flagellum length/mesosoma width 1.2, length/width F1, F2, F3 2.4, 2.1, 1.8, clava length/width 3.1, lengths pedicel/F1 0.7, lengths F1/F2 1.0, F1/F3 1.1, lengths F1, F2, F3/clava 0.7, 0.6, 0.6, widths F1/pedicel (dorsal view) 1.1, lengths antennal spicule/C3 0.2. *Mesosoma.* Length/width 1.3, mesoscutal mid-lobe length/width 0.9 (width measured in anterior part), mid-lobe with complete median groove, with six adnotaular setae on each side, lengths mesoscutum/scutellum (measured medially) 1.3, mesoscutellum length/width 0.9, length/width of enclosed space between submedian lines 2.4, distance between SMG/distance between SMG and SLG 1.4, lengths dorsellum/propodeum 0.5, propodeum with strong reticulation, propodeal callus with four setae. *Fore wing.* Costal cell length/width 8.0, lengths costal cell/marginal vein 0.9, lengths marginal/stigmal veins 3.5. *Gaster.* Elongate, length/width 2.6, lengths gaster/mesosoma 1.6, Gt_7_ length/width 0.8, length of longest cercal seta to next longest seta 1.8, longest cercal seta evenly curved, ovipositor sheaths reaching beyond apex of Gt_7_.

Colour. Head and mesosoma black with golden-green tinges, gaster dark brown with metallic tinges, scape yellowish-brown with dorsal edge pale brown, pedicel brown with dorsal part dark brown, flagellum dark brown, tegulae dark brown, wing venation yellowish-white, fore and mid coxae dark brown and hind coxa concolorous with body, trochanters and femora dark brown, tibiae yellowish-brown, tibiae yellowish-white.

MALE. Unknown.

#### Diagnosis

Similar to *T. agrilocidus*, but differs in having a longer distance between posterior ocelli and with shorter F1 and F2.

#### Etymology

Named after the collector of the species, Dieter Doczkal (ZSM).

#### Distribution

Germany.

#### Biology

##### Host

Unknown.

#### Notes

Holotype deposited in ZSM.

### Tetrastichus elodius sp. n.

A47EE0AB-45D3-5E98-AA24-6C7F0643674A

urn:lsid:zoobank.org:act:634458CE-6FDD-43BD-98B1-5381A8DF24DC

#### Materials

- **Type status:** Holotype. **Occurrence:** occurrenceDetails: http://www.boldsystems.org/index.php/API_Public/specimen?ids=BC-ZSM-HYM-13565-F04; catalogNumber: BC-ZSM-HYM-13565-F04; recordNumber: BC-ZSM-HYM-13565-F04; recordedBy: C. Hansson; individualID: BC-ZSM-HYM-13565-F04; individualCount: 1; sex: female; lifeStage: adult; **Taxon:** scientificName: Tetrastichus elodius; phylum: Arthropoda; class: Insecta; order: Hymenoptera; family: Eulophidae; genus: Tetrastichinae; taxonRemarks: Holotype deposited in MZLU; **Location:** country: Hungary; decimalLatitude: 47; decimalLongitude: 17.45; **Record Level:** type: PhysicalObject; language: en; institutionCode: MZLU; basisOfRecord: PreservedSpecimen
- **Type status:** Paratype. **Occurrence:** occurrenceDetails: http://www.boldsystems.org/index.php/API_Public/specimen?ids=BC ZSM HYM AE339; catalogNumber: BC ZSM HYM AE339; recordNumber: BC ZSM HYM AE339; recordedBy: G. Merkel-Wallner; individualID: BC ZSM HYM AE339; individualCount: 1; sex: female; lifeStage: adult; **Taxon:** scientificName: Tetrastichus elodius; phylum: Arthropoda; class: Insecta; order: Hymenoptera; family: Eulophidae; genus: Tetrastichinae; **Location:** country: Germany; decimalLatitude: 48.518; decimalLongitude: 13.726; **Record Level:** type: PhysicalObject; language: en; institutionCode: ZSM; basisOfRecord: PreservedSpecimen

#### Description

FEMALE holotype (Fig. 62). Body length 2.0 mm (paratype 2.1 mm). *Head*. Width/length in dorsal view 2.1, width/length in frontal view 1.3, POL/OOL 2.2, widths head/mesosoma 1.2, mouth width/malar space 1.4, malar space/eye height 0.7. *Antenna*. Scape length/eye height 1.0, pedicel+flagellum length/mesosoma width 1.4, length/width F1, F2, F3 2.2, 2.2, 2.0, clava length/width 3.8, lengths pedicel/F1 0.8, lengths F1/F2 1.0, F1/F3 1.1, lengths F1, F2, F3/clava 0.5, 0.5, 0.5, widths F1/pedicel (dorsal view) 1.1, lengths antennal spicule/C3 0.3. *Mesosoma*. Length/width 1.5, mesoscutal mid-lobe length/width 1.0 (width measured in anterior part), mid-lobe with a median groove in posterior ½, with five adnotaular setae on each side, lengths mesoscutum/mesoscutellum (measured medially) 1.2, mesoscutellum length/width 0.9, length/width of enclosed space between submedian grooves 2.4, distance between SMG/distance between SMG and SLG 1.5, lengths dorsellum/propodeum (measured medially) 0.5, propodeal callus with five setae. *Fore wing*. Costal cell length/width 10.0, lengths costal cell/marginal vein 1.0, lengths marginal/stigmal veins 2.8. *Gaster*. Ovate, length/width 1.7, lengths gaster/mesosoma 1.2, Gt_7_ length/width 0.6, length of longest cercal seta/next longest seta 2.2, longest cercal seta sinuate, ovipositor sheaths reach apex of Gt_7_, but not beyond.

Colour. Body metallic bluish, entire antenna dark brown, tegulae black, wings hyaline with venation yellowish-white, coxae and femora concolorous with body, trochanters black, tibiae yellowish-brown, fore tarsus dark brown, mid and hind tarsi yellowish-brown with T4 brown.

MALE. Unknown.

#### Diagnosis

Very similar to *T. julis*, female differs morphologically in having a longer distance between lateral ocelli and eyes and in having a shorter marginal vein compared to length of stigmal vein (see key).

#### Distribution

Germany, Hungary.

#### Ecology

##### Host

Unknown.

#### Notes

Holotype deposited in MZLU, paratype ZSM.

### Tetrastichus enodis sp. n.

E493B005-37FF-53CE-8DE6-A65C1443588C

urn:lsid:zoobank.org:act:03EF990E-E488-4C71-A451-A6E12F0B46F1

#### Materials

- **Type status:** Holotype. **Occurrence:** occurrenceDetails: http://www.boldsystems.org/index.php/API_Public/specimen?ids=BC-ZSM-HYM-29813-F03; catalogNumber: BC-ZSM-HYM-29813-F03; recordNumber: BC-ZSM-HYM-29813-F03; recordedBy: G.Delvare; individualID: BC-ZSM-HYM-29813-F03; individualCount: 1; sex: female; lifeStage: adult; **Taxon:** scientificName: Tetrastichus enodis; phylum: Arthropoda; class: Insecta; order: Hymenoptera; family: Eulophidae; genus: Tetrastichinae; taxonRemarks: Holotype deposited in NHM; **Location:** country: France; decimalLatitude: 43.5912; decimalLongitude: 3.25836; **Record Level:** type: PhysicalObject; language: en; institutionCode: NHM; basisOfRecord: PreservedSpecimen

#### Description

FEMALE holotype (Fig. 63). Body length 1.5 mm. *Head*. Width/length (dorsal view) 2.1, width/length (frontal view) 1.2, POL/OOL 1.8, widths head/mesosoma 1.2, mouth width/malar space 1.1, malar space/eye height 0.7. *Antenna*. Scape length/eye height 0.9, pedicel+flagellum length/mesosoma width 1.4, length/width F1, F2, F3 1.8, 1.6, 1.3, clava length/width 3.0, lengths pedicel/F1 0.8, lengths F1/F2 1.0, F1/F3 1.1, lengths F1, F2, F3/clava 0.4, 0.4, 0.4, widths F1/pedicel (dorsal view) 1.0, lengths antennal spicule/C3 0.2. *Mesosoma*. Length/width 1.5, mesoscutal mid-lobe length/width (width measured in anterior part) 1.0, mid-lobe with a median groove that is missing in very furthest anterior part, with four adnotaular setae on each side, lengths mesoscutum/mesoscutellum (measured medially) 1.5, mesoscutellum length/width 0.7, length/width of enclosed space between submedian grooves 2.0, distance between SMG/distance between SMG and SLG 1.8, lengths dorsellum/propodeum (measured medially) 0.9, propodeum smooth, propodeal callus with four setae. *Fore wing*. Costal cell length/width 11.8, lengths costal cell/marginal vein 1.0, lengths marginal/stigmal veins 3.1. *Gaster*. Ovate, length/width 2.4, lengths gaster/head+mesosoma 1.6, Gt_7_ length/width 0.7, length of longest cercal seta/next longest seta 1.3, longest seta weakly curved almost straight, ovipositor sheaths projecting beyond apex of Gt_7_.

Colour. Body dark brown to black with weak metallic tinges, antenna pale brown, tegulae dark brown, wings hyaline with veins pale yellowish-brown, coxae black with weak metallic tinges, femora and trochanters dark brown, fore tibia and tarsus infuscate, mid and hind tibiae pale yellowish-brown, mid and and hind tarsi white with T4 pale brown.

MALE. Unknown.

#### Diagnosis

Frons with interscrobal area as a wide carina reaching almost to median ocellus; propodeum smooth and shiny.

#### Distribution

France.

#### Ecology

##### Host

Unknown.

##### Remarks

The left femora, tibia and tarsus of mid and hind leg are detached and glued to a separate card.

#### Notes

Holotype deposited in NHM.

### Tetrastichus fadus sp. n.

8B26B498-8DCB-56B6-B88E-C73F62EC490C

urn:lsid:zoobank.org:act:7D70AAF5-262D-4830-8523-AF7E4713C15F

#### Materials

- **Type status:** Holotype. **Occurrence:** occurrenceDetails: http://www.boldsystems.org/index.php/API_Public/specimen?ids=BC-ZSM-HYM-22524-H10; catalogNumber: BC-ZSM-HYM-22524-H10; recordNumber: BC-ZSM-HYM-22524-H10; recordedBy: E. Shevtsova; individualID: BC-ZSM-HYM-22524-H10; individualCount: 1; sex: female; lifeStage: adult; **Taxon:** scientificName: Tetrastichus fadus; phylum: Arthropoda; class: Insecta; order: Hymenoptera; family: Eulophidae; genus: Tetrastichinae; taxonRemarks: Holotype deposited in MZLU; **Location:** country: Russia; decimalLatitude: 60.401; decimalLongitude: 30.373; **Record Level:** type: PhysicalObject; language: en; institutionCode: MZLU; basisOfRecord: PreservedSpecimen

#### Description

FEMALE holotype (Fig. 64). Body length 2.6 mm. *Head*. Width/length (dorsal view) 2.2, width/length (frontal view) 1.2, POL/OOL 1.8, widths head/mesosoma 1.1, mouth width/malar space 1.3, malar space/eye height 0.7. *Antenna*. Scape length/eye height 0.8, pedicel+flagellum length/mesosoma width 1.3, length/width F1, F2, F3 2.1, 2.0, 2.0, clava length/width 3.1, lengths pedicel/F1 0.6, lengths F1/F2 1.1, F1/F3 1.1, lengths F1, F2, F3/clava 0.6, 0.6, 0.6, widths F1/pedicel (dorsal view) 1.4, lengths antennal spicule/C3 0.2. *Mesosoma*. Length/width 1.4, mesoscutal mid-lobe length/width (width measured in anterior part) 1.0, mid-lobe without median groove, with four adnotaular setae on each side, lengths mesoscutum/mesoscutellum (measured medially) 1.5, mesoscutellum length/width 0.8, length/width of enclosed space between submedian grooves 2.1, distance between SMG/distance between SMG and SLG 1.9, lengths dorsellum/propodeum (measured medially) 0.6, propodeum with strong reticulation, propodeal callus with five setae. *Fore wing*. Costal cell length/width 10.0, lengths costal cell/marginal vein 1.0, lengths marginal/stigmal veins 3.3. *Gaster*. Elongate-acuminate, length/width 2.4, lengths gaster/head+mesosoma 1.3, Gt_7_ length/width 1.2, length of longest cercal seta/next longest seta 1.7, longest cercal seta almost straight, ovipositor sheaths projecting beyond apex of Gt_7_.

Colour. Body with weak golden-green tinges, scape yellowish-brown, pedicel and flagellum dark brown, tegulae dark brown, wings hyaline with veins yellowish-brown, coxae concolorous with body, trochanters and femora dark brown, tibiae and tarsi yellowish-brown.

MALE. Unknown.

#### Diagnosis

Gaster elongate-acuminate, length/width 2.4, lengths gaster/head+mesosoma 1.3, Gt_7_ length/width 1.2; antennal clava 1.0× as long as F2+F3 and with distinct constriction between C1 and C2.

#### Distribution

Russia.

#### Ecology

##### Host

Unknown.

#### Notes

Holotype deposited in MZLU.

### Tetrastichus gredius sp. n.

2BCFA82F-84BA-5099-8584-F78082D4ADC5

urn:lsid:zoobank.org:act:BD8FE3EA-DB41-47AA-8FD2-FF60EF3DDF9A

#### Materials

- **Type status:** Holotype. **Occurrence:** occurrenceDetails: http://www.boldsystems.org/index.php/API_Public/specimen?ids=BC-ZSM-HYM-26563-D08; catalogNumber: BC-ZSM-HYM-26563-D08; recordNumber: BC-ZSM-HYM-26563-D08; recordedBy: C. Hansson; individualID: BC-ZSM-HYM-26563-D08; individualCount: 1; sex: female; lifeStage: adult; **Taxon:** scientificName: Tetrastichus gredius; phylum: Arthropoda; class: Insecta; order: Hymenoptera; family: Eulophidae; genus: Tetrastichinae; taxonRemarks: Holotype deposited in MZLU; **Location:** country: Sweden; decimalLatitude: 55.0501; decimalLongitude: 12.915; **Record Level:** type: PhysicalObject; language: en; institutionCode: MZLU; basisOfRecord: PreservedSpecimen
- **Type status:** Paratype. **Occurrence:** occurrenceDetails: http://www.boldsystems.org/index.php/API_Public/specimen?ids=BC-ZSM-HYM-20721-F06; catalogNumber: BC-ZSM-HYM-20721-F06; recordNumber: BC-ZSM-HYM-20721-F06; recordedBy: C. Hansson; individualID: BC-ZSM-HYM-20721-F06; individualCount: 1; sex: female; lifeStage: adult; **Taxon:** scientificName: Tetrastichus gredius; phylum: Arthropoda; class: Insecta; order: Hymenoptera; family: Eulophidae; genus: Tetrastichinae; **Location:** country: Sweden; decimalLatitude: 55.533; decimalLongitude: 12.917; **Record Level:** type: PhysicalObject; language: en; institutionCode: MZLU; basisOfRecord: PreservedSpecimen

#### Description

FEMALE holotype (Fig. 65). Body length 2.6 mm. *Head*. Width/length in dorsal view 2.2, width/length in frontal view 1.2, POL/OOL 1.8, widths head/mesosoma 1.1, mouth width/malar space 1.3, malar space/eye height 0.7. *Antenna*. Scape length/eye height 0.8, pedicel+flagellum length/mesosoma width 1.3, length/width F1, F2, F3 2.1, 2.0, 2.0, clava length/width 3.1, lengths pedicel/F1 0.6, lengths F1/F2 1.1, F1/F3 1.1, lengths F1, F2, F3/clava 0.6, 0.6, 0.6, widths F1/pedicel (dorsal view) 1.4, lengths antennal spicule/C3 0.2. *Mesosoma*. Length/width 1.4, mesoscutal mid-lobe length/width 1.0 (width measured in anterior part), mid-lobe with a complete median groove , with five adnotaular setae on each side, lengths mesoscutum/mesoscutellum (measured medially) 1.5, mesoscutellum length/width 0.8, length/width of enclosed space between submedian grooves 2.1, distance between SMG/distance between SMG and SLG 1.9, lengths dorsellum/propodeum (measured medially) 0.6, propodeal callus with five setae. *Fore wing*. Costal cell length/width 8.2, lengths costal cell/marginal vein 1.0, lengths marginal/stigmal veins 3.3. *Gaster*. Elongate-acuminate, length/width 2.4, lengths gaster/head+mesosoma 1.3, Gt_7_ length/width 1.2, length of longest cercal seta/next longest seta 1.7, longest cercal seta straight, ovipositor sheaths projecting beyond apex of Gt_7_.

Colour. Body with weak golden-green tinges, entire antenna dark brown, tegulae black, wings hyaline with venation yellowish-white, coxae and femora concolorous with body, trochanters black, fore and hind tibia brownish, mid tibia dark brown, tarsi yellowish-brown.

MALE. Unknown.

#### Diagnosis

Mouth opening 1.3× (1.27×) malar space; scape dark brown; gaster elongate-acuminate, length/width 2.4, lengths gaster/head+mesosoma 1.3, Gt_7_ length/width 1.2.

#### Distribution

Sweden.

#### Ecology

##### Host

Unknown.

#### Notes

Holotype deposited in MZLU, paratype in MZLU.

### Tetrastichus halidayi

(Graham, 1961)

0FA0A0BC-C503-5C22-BE47-8127BDF217B3

Aprostocetus halidayi Graham 1961:5–8. Holotype ♀ in OUMNH, examined (Fig. 66). Transferred to *Tetrastichus* by Domenichini 1966:94.

#### Description

See Graham (1961).

#### Diagnosis

Mouth opening very wide, 2.0–2.2× malar space; mandibles very large, with outer tooth falcate and separated by a wide gap from the two small inner teeth, which are subacute and closely approximated; female gaster relatively long, 1.8× as long as wide with ovipositor sheaths protruding beyond apex of Gt_7_.

#### Distribution

(Former) Czechoslovakia, Germany, Ireland, Norway, Sweden, United Kingdom (Graham 1961), Russia and Switzerland (**new records**).

#### Ecology

##### Host

*Agropus ahrensi* Germar (Coleoptera: Chrysomelidae) (Graham 1991).

##### Material examined

Type material: holotype ♀ (OUMNH). Additional material (219♀ 57♂): Germany 2♂ (UCRC), Russia 8♀ (MZLU), Sweden 197♀ 51♂ (MZLU, NHM, NHRS, ZSM), Switzerland 1♀ (ZSM), United Kingdom 13♀ 4♂ (NHM).

### Tetrastichus heeringi

Delucchi, 1954

E1DAE348-B08B-5A1D-888F-3AA58C57008E

Tetrastichus heeringi Delucchi 1954:99–101. Lectotype ♀ in ETHZ, designated by Baur 2001, not examined.

#### Description

See Graham (1991) and Fig. 67.

#### Diagnosis

Diagnosis. Female antenna with Fl 3.0–3.2× as long as wide and 0.7–0.8× length of clava, the latter 0.8–0.9× as long as F2+F3, F1 about as wide as width of pedicel; male antenna with whorled setae of funiculars reaching beyond the tips of funicular attached to, scape 2.4–2.6× as long as wide; female gaster 1.8–1.9× as long as wide.

#### Distribution

Bulgaria, (former) Czechoslovakia, France, Germany, Hungary, Italy (Graham 1991), Sweden (Hedqvist 2003) and Finland **(new record**).

#### Ecology

##### Host

*Agrilus aurichalceus* Redtenbacher, *A. integerrimus* Ratzeburg, *A. viridis* (L.) (Coleoptera: Buprestidae) (Graham 1991).

#### Material examined

Non-type material (15♀ 4♂): Finland 5♀ 2♂ (NHM), France 3♀ (GD, NHM), Sweden 7♀ 2♂ (NHM). Fig.

### Tetrastichus heterus

Graham, 1991

6C4A70BB-1308-53C5-9ED1-337D38E6E13D

Tetrastichus heterus Graham 1991:247–248. Holotype ♀ in NHM, examined (Fig. 68).

#### Description

See Graham (1991).

#### Diagnosis

Antennal clava 0.8× as long as F2+F3; gaster 2.3× as long as wide, as long as head+mesosoma.

#### Distribution

Montenegro (Graham 1991).

#### Ecology

##### Host

Unknown.

#### Material examined

Type material: holotype ♀ (NHM, type no. 5.3619).

### Tetrastichus ilithyia

(Walker, 1839)

E29CD346-B50B-5E9D-9EE1-63FFF1615D6D

Cirrospilus Walker 1839b:355. Lectotype ♀ in NHM, designated by Graham 1961:38, examined (Fig. 69). Transferred to *Tetrastichus* by Walker 1846:74, to *Aprostocetus* by Graham 1961:38 and back to *Tetrastichus* by Domenichini 1966:91.

#### Description

See Graham (1991).

#### Diagnosis

Female with antennal spicule 0.5× as long as C3 and scape longer than eye; mouth opening 1.14× malar space in both sexes.

#### Distribution

(Former) Czechoslovakia, Germany, Ireland, United Kingdom (Graham 1991), Sweden (Hedqvist 2003) and Russia (**new record**).

#### Ecology

##### Host

Unknown.

##### Material examined

Type material: lectotype ♀ of *C. Ilithyia* (NHM type no. 5.1940). Additional material (10♀ 3♂): Russia 5♀ 3♂ (MZLU, ZSM), United Kingdom 5♀ (NHM).

### Tetrastichus inscitus sp. n.

2746DB52-7C52-51AA-9301-ADC536403122

urn:lsid:zoobank.org:act:F0E8692D-E47A-4145-99BD-31D7F2928D7C

#### Materials

- **Type status:** Holotype. **Occurrence:** occurrenceDetails: http://www.boldsystems.org/index.php/API_Public/specimen?ids=BC-ZSM-HYM-20721-F11; catalogNumber: BC-ZSM-HYM-20721-F11; recordNumber: BC-ZSM-HYM-20721-F11; recordedBy: C. Hansson; individualID: BC-ZSM-HYM-20721-F11; individualCount: 1; sex: female; lifeStage: adult; **Taxon:** scientificName: Tetrastichus inscitus; phylum: Arthropoda; class: Insecta; order: Hymenoptera; family: Eulophidae; genus: Tetrastichinae; taxonRemarks: Holotype deposited in MZLU; **Location:** country: Sweden; decimalLatitude: 55.7; decimalLongitude: 13.5; **Record Level:** type: PhysicalObject; language: en; institutionCode: MZLU; basisOfRecord: PreservedSpecimen
- **Type status:** Paratype. **Occurrence:** occurrenceDetails: http://www.boldsystems.org/index.php/API_Public/specimen?ids=BC-ZSM-HYM-29813-B12; catalogNumber: BC-ZSM-HYM-29813-B12; recordNumber: BC-ZSM-HYM-29813-B12; recordedBy: A.Sundholm; individualID: BC-ZSM-HYM-29813-B12; individualCount: 1; sex: female; lifeStage: adult; **Taxon:** scientificName: Tetrastichus inscitus; phylum: Arthropoda; class: Insecta; order: Hymenoptera; family: Eulophidae; genus: Tetrastichinae; **Location:** country: Sweden; decimalLatitude: 55.7047; decimalLongitude: 13.191; **Record Level:** type: PhysicalObject; language: en; basisOfRecord: PreservedSpecimen
- **Type status:** Paratype. **Occurrence:** occurrenceDetails: http://www.boldsystems.org/index.php/API_Public/specimen?ids=BC-ZSM-HYM-22523-A05; catalogNumber: BC-ZSM-HYM-22523-A05; recordNumber: BC-ZSM-HYM-22523-A05; recordedBy: Swedish Malaise Trap Project; individualID: BC-ZSM-HYM-22523-A05; individualCount: 1; sex: female; lifeStage: adult; **Taxon:** scientificName: Tetrastichus inscitus; phylum: Arthropoda; class: Insecta; order: Hymenoptera; family: Eulophidae; genus: Tetrastichinae; **Location:** country: Sweden; decimalLatitude: 56.378; decimalLongitude: 16.592; **Record Level:** type: PhysicalObject; language: en; basisOfRecord: PreservedSpecimen
- **Type status:** Paratype. **Occurrence:** occurrenceDetails: http://www.boldsystems.org/index.php/API_Public/specimen?ids=BC-ZSM-HYM-26563-A09; catalogNumber: BC-ZSM-HYM-26563-A09; recordNumber: BC-ZSM-HYM-26563-A09; recordedBy: C. Hansson; individualID: BC-ZSM-HYM-26563-A09; individualCount: 1; sex: male; lifeStage: adult; **Taxon:** scientificName: Tetrastichus inscitus; phylum: Arthropoda; class: Insecta; order: Hymenoptera; family: Eulophidae; genus: Tetrastichinae; **Location:** country: Sweden; decimalLatitude: 55.6664; decimalLongitude: 13.6242; **Record Level:** type: PhysicalObject; language: en; basisOfRecord: PreservedSpecimen
- **Type status:** Paratype. **Occurrence:** occurrenceDetails: http://www.boldsystems.org/index.php/API_Public/specimen?ids=BC-ZSM-HYM-27769-E08; catalogNumber: BC-ZSM-HYM-27769-E08; recordNumber: BC-ZSM-HYM-27769-E08; recordedBy: C. Hansson; individualID: BC-ZSM-HYM-27769-E08; individualCount: 1; sex: female; lifeStage: adult; **Taxon:** scientificName: Tetrastichus inscitus; phylum: Arthropoda; class: Insecta; order: Hymenoptera; family: Eulophidae; genus: Tetrastichinae; **Location:** country: Sweden; decimalLatitude: 56.676; decimalLongitude: 16.558; **Record Level:** type: PhysicalObject; language: en; basisOfRecord: PreservedSpecimen
- **Type status:** Paratype. **Occurrence:** occurrenceDetails: http://www.boldsystems.org/index.php/API_Public/specimen?ids=BC-ZSM-HYM-22523-C09; catalogNumber: BC-ZSM-HYM-22523-C09; recordNumber: BC-ZSM-HYM-22523-C09; recordedBy: Swedish Malaise Trap Project; individualID: BC-ZSM-HYM-22523-C09; individualCount: 1; sex: female; lifeStage: adult; **Taxon:** scientificName: Tetrastichus inscitus; phylum: Arthropoda; class: Insecta; order: Hymenoptera; family: Eulophidae; genus: Tetrastichinae; **Location:** country: Sweden; decimalLatitude: 57.322; decimalLongitude: 18.203; **Record Level:** type: PhysicalObject; language: en; basisOfRecord: PreservedSpecimen
- **Type status:** Paratype. **Occurrence:** occurrenceDetails: http://www.boldsystems.org/index.php/API_Public/specimen?ids=BC-ZSM-HYM-20721-H09; catalogNumber: BC-ZSM-HYM-20721-H09; recordNumber: BC-ZSM-HYM-20721-H09; recordedBy: C. Hansson; individualID: BC-ZSM-HYM-20721-H09; individualCount: 1; sex: female; lifeStage: adult; **Taxon:** scientificName: Tetrastichus inscitus; phylum: Arthropoda; class: Insecta; order: Hymenoptera; family: Eulophidae; genus: Tetrastichinae; **Location:** country: Sweden; decimalLatitude: 55.7; decimalLongitude: 13.45; **Record Level:** type: PhysicalObject; language: en; basisOfRecord: PreservedSpecimen
- **Type status:** Paratype. **Occurrence:** occurrenceDetails: http://www.boldsystems.org/index.php/API_Public/specimen?ids=BC-ZSM-HYM-20721-F01; catalogNumber: BC-ZSM-HYM-20721-F01; recordNumber: BC-ZSM-HYM-20721-F01; recordedBy: C. Hansson; individualID: BC-ZSM-HYM-20721-F01; individualCount: 1; sex: female; lifeStage: adult; **Taxon:** scientificName: Tetrastichus inscitus; phylum: Arthropoda; class: Insecta; order: Hymenoptera; family: Eulophidae; genus: Tetrastichinae; **Location:** country: Sweden; decimalLatitude: 55.7; decimalLongitude: 13.45; **Record Level:** type: PhysicalObject; language: en; basisOfRecord: PreservedSpecimen
- **Type status:** Paratype. **Occurrence:** occurrenceDetails: http://www.boldsystems.org/index.php/API_Public/specimen?ids=BC-ZSM-HYM-13565-C06; catalogNumber: BC-ZSM-HYM-13565-C06; recordNumber: BC-ZSM-HYM-13565-C06; recordedBy: SMTP project; individualID: BC-ZSM-HYM-13565-C06; individualCount: 1; sex: female; lifeStage: adult; **Taxon:** scientificName: Tetrastichus inscitus; phylum: Arthropoda; class: Insecta; order: Hymenoptera; family: Eulophidae; genus: Tetrastichinae; **Location:** country: Sweden; decimalLatitude: 59.767; decimalLongitude: 13.467; **Record Level:** type: PhysicalObject; language: en; basisOfRecord: PreservedSpecimen
- **Type status:** Paratype. **Occurrence:** occurrenceDetails: http://www.boldsystems.org/index.php/API_Public/specimen?ids=BC-ZSM-HYM-20721-D04; catalogNumber: BC-ZSM-HYM-20721-D04; recordNumber: BC-ZSM-HYM-20721-D04; recordedBy: C. Hansson; individualID: BC-ZSM-HYM-20721-D04; individualCount: 1; sex: female; lifeStage: adult; **Taxon:** scientificName: Tetrastichus inscitus; phylum: Arthropoda; class: Insecta; order: Hymenoptera; family: Eulophidae; genus: Tetrastichinae; **Location:** country: Sweden; decimalLatitude: 55.7; decimalLongitude: 13.15; **Record Level:** type: PhysicalObject; language: en; basisOfRecord: PreservedSpecimen
- **Type status:** Paratype. **Occurrence:** occurrenceDetails: http://www.boldsystems.org/index.php/API_Public/specimen?ids=BC-ZSM-HYM-20721-D03; catalogNumber: BC-ZSM-HYM-20721-D03; recordNumber: BC-ZSM-HYM-20721-D03; recordedBy: C. Hansson; individualID: BC-ZSM-HYM-20721-D03; individualCount: 1; sex: female; lifeStage: adult; **Taxon:** scientificName: Tetrastichus inscitus; phylum: Arthropoda; class: Insecta; order: Hymenoptera; family: Eulophidae; genus: Tetrastichinae; **Location:** country: Sweden; decimalLatitude: 55.7; decimalLongitude: 13.15; **Record Level:** type: PhysicalObject; language: en; basisOfRecord: PreservedSpecimen
- **Type status:** Paratype. **Occurrence:** occurrenceDetails: http://www.boldsystems.org/index.php/API_Public/specimen?ids=BC-ZSM-HYM-20721-D01; catalogNumber: BC-ZSM-HYM-20721-D01; recordNumber: BC-ZSM-HYM-20721-D01; recordedBy: C. Hansson; individualID: BC-ZSM-HYM-20721-D01; individualCount: 1; sex: female; lifeStage: adult; **Taxon:** scientificName: Tetrastichus inscitus; phylum: Arthropoda; class: Insecta; order: Hymenoptera; family: Eulophidae; genus: Tetrastichinae; **Location:** country: Sweden; decimalLatitude: 55.7; decimalLongitude: 13.15; **Record Level:** type: PhysicalObject; language: en; basisOfRecord: PreservedSpecimen
- **Type status:** Paratype. **Occurrence:** occurrenceDetails: http://www.boldsystems.org/index.php/API_Public/specimen?ids=BC-ZSM-HYM-27770-H09; catalogNumber: BC-ZSM-HYM-27760-H09; recordNumber: BC-ZSM-HYM-27760-H09; recordedBy: A.C.Galsworthy; individualID: BC-ZSM-HYM-27770-H09; individualCount: 1; sex: female; lifeStage: adult; **Taxon:** scientificName: Tetrastichus inscitus; phylum: Arthropoda; class: Insecta; order: Hymenoptera; family: Eulophidae; genus: Tetrastichinae; **Location:** country: United Kingdom; decimalLatitude: 51.4114; decimalLongitude: 0.0358; **Record Level:** type: PhysicalObject; language: en; basisOfRecord: PreservedSpecimen
- **Type status:** Paratype. **Occurrence:** occurrenceDetails: http://www.boldsystems.org/index.php/API_Public/specimen?ids=BC-ZSM-HYM-20721-H10; catalogNumber: BC-ZSM-HYM-20721-H10; recordNumber: BC-ZSM-HYM-20721-H10; recordedBy: C. Hansson; individualID: BC-ZSM-HYM-20721-H10; individualCount: 1; sex: female; lifeStage: adult; **Taxon:** scientificName: Tetrastichus inscitus; phylum: Arthropoda; class: Insecta; order: Hymenoptera; family: Eulophidae; genus: Tetrastichinae; **Location:** country: Sweden; decimalLatitude: 55.7; decimalLongitude: 13.45; **Record Level:** type: PhysicalObject; language: en; basisOfRecord: PreservedSpecimen
- **Type status:** Paratype. **Occurrence:** occurrenceDetails: http://www.boldsystems.org/index.php/API_Public/specimen?ids=BC-ZSM-HYM-25460-B03; catalogNumber: BC-ZSM-HYM-25460-B03; recordNumber: BC-ZSM-HYM-25460-B03; recordedBy: C. Hansson; individualID: BC-ZSM-HYM-25460-B03; individualCount: 1; sex: male; lifeStage: adult; **Taxon:** scientificName: Tetrastichus inscitus; phylum: Arthropoda; class: Insecta; order: Hymenoptera; family: Eulophidae; genus: Tetrastichinae; **Location:** country: Sweden; decimalLatitude: 55.6928; decimalLongitude: 13.1678; **Record Level:** type: PhysicalObject; language: en; basisOfRecord: PreservedSpecimen
- **Type status:** Paratype. **Occurrence:** occurrenceDetails: http://www.boldsystems.org/index.php/API_Public/specimen?ids=BC-ZSM-HYM-27493-G01; catalogNumber: BC-ZSM-HYM-27493-G01; recordNumber: BC-ZSM-HYM-27493-G01; recordedBy: SMTP; individualID: BC-ZSM-HYM-27493-G01; individualCount: 1; sex: female; lifeStage: adult; **Taxon:** scientificName: Tetrastichus inscitus; phylum: Arthropoda; class: Insecta; order: Hymenoptera; family: Eulophidae; genus: Tetrastichinae; **Location:** country: Sweden; decimalLatitude: 58.339; decimalLongitude: 11.151; **Record Level:** type: PhysicalObject; language: en; basisOfRecord: PreservedSpecimen
- **Type status:** Paratype. **Occurrence:** occurrenceDetails: http://www.boldsystems.org/index.php/API_Public/specimen?ids=BC-ZSM-HYM-29813-D06|BC-ZSM-HYM-29813-B07; catalogNumber: BC-ZSM-HYM-29813-B07; recordNumber: BC-ZSM-HYM-29813-B07; recordedBy: A.Jansson; individualID: BC-ZSM-HYM-29813-B07; individualCount: 1; sex: F; lifeStage: Adult; associatedMedia: http://www.boldsystems.org/pics/BCHYM/BC-ZSM-HYM-29813-B07+1516986268.jpg; **Taxon:** scientificName: Tetrastichus inscitus; phylum: Arthropoda; class: Insecta; order: Hymenoptera; family: Eulophidae; genus: Tetrastichus; **Location:** country: Sweden; locality: Oerebro; decimalLatitude: 59.2753; decimalLongitude: 15.2134; **Identification:** identifiedBy: Christer Hansson
- **Type status:** Paratype. **Occurrence:** occurrenceDetails: http://www.boldsystems.org/index.php/API_Public/specimen?ids=BC-ZSM-HYM-29813-D06|BC-ZSM-HYM-29813-B07; catalogNumber: BC-ZSM-HYM-29813-D06; recordNumber: BC-ZSM-HYM-29813-D06; recordedBy: SMTP; individualID: BC-ZSM-HYM-29813-D06; individualCount: 1; sex: F; lifeStage: Adult; associatedMedia: http://www.boldsystems.org/pics/BCHYM/BC-ZSM-HYM-29813-D06+1516986282.jpg; **Taxon:** scientificName: Tetrastichus inscitus; phylum: Arthropoda; class: Insecta; order: Hymenoptera; family: Eulophidae; genus: Tetrastichus; **Location:** country: Sweden; locality: Roleks; decimalLatitude: 57.5391; decimalLongitude: 18.3498; **Identification:** identifiedBy: Christer Hansson

#### Description

FEMALE holotype (Fig. 70). Body length 2.0 mm (paratypes 1.6–2.2 mm). *Head*. Width/length in dorsal view 2.0, width/length in frontal view 1.4, POL/OOL 1.6, widths head/mesosoma 1.2, mouth width/malar space 1.6, malar space/eye height 0.7. *Antenna*. Scape length/eye height 1.1, pedicel+flagellum length/mesosoma width 1.5, length/width F1, F2, F3 2.0, 1.7, 1.7, clava length/width 3.6, lengths pedicel/F1 0.8, lengths F1/F2 1.1, F1/F3 1.1, lengths F1, F2, F3/clava 0.5, 0.5, 0.5, widths F1/pedicel (dorsal view) 1.3, lengths antennal spicule/C3 0.3. *Mesosoma*. Length/width 1.5, mesoscutal mid-lobe length/width 0.9 (width measured in anterior part), mid-lobe with a median groove in posterior ½, with 3+4 adnotaular setae on each side, lengths mesoscutum/mesoscutellum (measured medially) 1.2, mesoscutellum length/width 1.0, length/width of enclosed space between submedian grooves 2.5, distance between SMG/distance between SMG and SLG 1.7, lengths dorsellum/propodeum (measured medially) 0.5, propodeal callus with five setae. *Fore wing*. Costal cell length/width 11.7, lengths costal cell/marginal vein 1.0, lengths marginal/stigmal veins 3.2. *Gaster*. Ovate, length/width 1.6, lengths gaster/mesosoma 1.3, Gt_7_ length/width 0.6, length of longest cercal seta/next longest seta nm, longest cercal seta sinuate, ovipositor sheaths reach apex of Gt_7_, but not beyond.

Colour. Body metallic bluish, entire antenna dark brown, tegulae dark brown, wings hyaline with venation dark yellowish-brown, coxae and femora concolorous with body, trochanters black, fore tibia dark yellowish-brown, mid and hind tibiae dark brown, fore tarsus dark brown, mid and hind tarsi tarsi dark yellowish-brown with T4 darkest.

MALE. Body length 1.4–1.6 mm. *Head.* Width/length in dorsal view 2.2, width/length in frontal view 1.2, eye height/malar space 1.5, mouth width/malar space 1.1, widths head/mesosoma 1.2. *Antenna.* F1–F4 with basal whorls of setae, reaching beyond apex of corresponding flagellomere, whorled setae on F1 1.2× as long as F1 length, scape length/eye height 1.2, scape length/width 2.8, ventral plaque placed centrally, lengths ventral plaque/scape 0.7, pedicel+flagellum length/mesosoma width 1.8, length/width F1, F2, F3, F4 1.8, 1.8, 2.0, 1.8, clava length/width 4.0, lengths pedicel/F1 0.9, lengths F1/F2 0.9, F1/F3 0.8, F1/F4 0.9, lengths F1, F2, F3 F4 /clava 0.4, 0.5, 0.5, 0.5.

Colour. As in female.

#### Diagnosis

Very similar to *T. julis*, female differs morphologically as indicated in the key.

#### Distribution

Sweden and United Kingdom.

#### Ecology

##### Host

Unknown.

#### Notes

Holotype deposited in MZLU, paratypes in MZLU, NHM, SMTP and ZSM.

### Tetrastichus julis

(Walker, 1839)

843F7CF6-9C13-50C1-98EA-7A52D1CC0831

Cirrospilus Walker 1839b:354. Lectotype ♂ in NHM, designated by Graham 1961:38, examined (Fig. 71). Combined to *Aprostocetus* by Graham 1961:38 and to *Tetrastichus* by Domenichini 1966:90.
Tetrastichus Walker 1872:128. Lectotype ♀ in NHM, designated by Graham 1961:40, examined. Synonymised with *T. julis* by Graham 1991:235.

#### Description

See Graham (1991).

#### Diagnosis

Mouth opening 1.4–1.6× malar space; more???

#### Distribution

France, Germany, Poland, Portugal (Madeira), Romania, Sweden, United Kingdom (Graham 1991), Austria and Russia (**new records**).

#### Ecology

##### Host

Lema (Oulema) spp. (Coleoptera: Chrysomelidae), gregarious endoparasitoid of host larva (Graham 1991).

##### Material examined

Type material: lectotypes ♂ of *C. julis* (NHM, type no. 5.1941), and ♀ of *T. maderae* (NHM, type no. 5.1370). Additional material (18♀ 8♂): Austria 1♀ (UCRC), France 1♀ (G), Germany 2♀ (MZLU), Russia 1♀ (UCRC), Sweden 13♀ 8♂ (MZLU, SMTP, ZSM).

### Tetrastichus lanius sp. n.

227A2FAD-A3DB-5E9E-B144-89948672E1D0

urn:lsid:zoobank.org:act:C5A5DCE4-E547-4ADC-AAD4-00305DEDCCB0

#### Materials

- **Type status:** Holotype. **Occurrence:** occurrenceDetails: http://www.boldsystems.org/index.php/API_Public/specimen?ids=BC-ZSM-HYM-27768-D02; catalogNumber: BC-ZSM-HYM-27768-D02; recordNumber: BC-ZSM-HYM-27768-D02; recordedBy: J.S. Noyes; individualID: BC-ZSM-HYM-27768-D02; individualCount: 1; sex: female; lifeStage: adult; **Taxon:** scientificName: Tetrastichus lanius; phylum: Arthropoda; class: Insecta; order: Hymenoptera; family: Eulophidae; genus: Tetrastichinae; taxonRemarks: Holotype deposited in NHM; **Location:** country: Romania; decimalLatitude: 46.985; decimalLongitude: 27.585; **Record Level:** type: PhysicalObject; language: en; institutionCode: NHM; basisOfRecord: PreservedSpecimen
- **Type status:** Paratype. **Occurrence:** occurrenceDetails: http://www.boldsystems.org/index.php/API_Public/specimen?ids=BC-ZSM-HYM-22523-G05; catalogNumber: BC-ZSM-HYM-22523-G05; recordNumber: BC-ZSM-HYM-22523-G05; recordedBy: O.Popovici; individualID: BC-ZSM-HYM-22523-G05; individualCount: 1; sex: female; lifeStage: adult; **Taxon:** scientificName: Tetrastichus lanius; phylum: Arthropoda; class: Insecta; order: Hymenoptera; family: Eulophidae; genus: Tetrastichinae; **Location:** country: Romania; decimalLatitude: 47.045; decimalLongitude: 27.603; **Record Level:** type: PhysicalObject; language: en; institutionCode: MZLU; basisOfRecord: PreservedSpecimen
- **Type status:** Paratype. **Occurrence:** occurrenceDetails: http://www.boldsystems.org/index.php/API_Public/specimen?ids=BC-ZSM-HYM-27768-D06; catalogNumber: BC-ZSM-HYM-27768-D06; recordNumber: BC-ZSM-HYM-27768-D06; recordedBy: J.S. Noyes; individualID: BC-ZSM-HYM-27768-D06; individualCount: 1; sex: female; lifeStage: adult; **Taxon:** scientificName: Tetrastichus lanius; phylum: Arthropoda; class: Insecta; order: Hymenoptera; family: Eulophidae; genus: Tetrastichinae; **Location:** country: Romania; decimalLatitude: 46.985; decimalLongitude: 27.585; **Record Level:** type: PhysicalObject; language: en; institutionCode: NHM; basisOfRecord: PreservedSpecimen
- **Type status:** Paratype. **Occurrence:** occurrenceDetails: http://www.boldsystems.org/index.php/API_Public/specimen?ids=BC-ZSM-HYM-29813-E03; catalogNumber: BC-ZSM-HYM-29813-E03; recordNumber: BC-ZSM-HYM-29813-E03; recordedBy: H.Tussac; individualID: BC-ZSM-HYM-29813-E03; individualCount: 1; sex: female; lifeStage: adult; **Taxon:** scientificName: Tetrastichus lanius; phylum: Arthropoda; class: Insecta; order: Hymenoptera; family: Eulophidae; genus: Tetrastichinae; **Location:** country: France; decimalLatitude: 44.4064; decimalLongitude: 1.49961; **Record Level:** type: PhysicalObject; language: en; institutionCode: GD; basisOfRecord: PreservedSpecimen
- **Type status:** Paratype. **Occurrence:** occurrenceDetails: http://www.boldsystems.org/index.php/API_Public/specimen?ids=BC-ZSM-HYM-27768-A08; catalogNumber: BC-ZSM-HYM-27768-A08; recordNumber: BC-ZSM-HYM-27768-A08; recordedBy: J.S. Noyes; individualID: BC-ZSM-HYM-27768-A08; individualCount: 1; sex: female; lifeStage: adult; **Taxon:** scientificName: Tetrastichus lanius; phylum: Arthropoda; class: Insecta; order: Hymenoptera; family: Eulophidae; genus: Tetrastichinae; **Location:** country: Romania; decimalLatitude: 47.011; decimalLongitude: 27.603; **Record Level:** type: PhysicalObject; language: en; institutionCode: NHM; basisOfRecord: PreservedSpecimen

#### Description

FEMALE holotype (Fig. 72). Body length 2.2 mm (paratypes 2.0–2.4 mm). *Head*. Width/length in dorsal view 2.2, width/length in frontal view 1.3, POL/OOL 1.9, widths head/mesosoma 1.2, mouth width/malar space 1.5, malar space/eye height 0.6. *Antenna*. Scape length/eye height 0.9, pedicel+flagellum length/mesosoma width 1.5, length/width F1, F2, F3 2.3, 2.1, 1.8, clava length/width 3.8, lengths pedicel/F1 0.6, lengths F1/F2 1.1, F1/F3 1.2, lengths F1, F2, F3/clava 0.6, 0.5, 0.5, widths F1/pedicel (dorsal view) 1.3, lengths antennal spicule/C3 0.3. *Mesosoma*. Length/width 1.2, mesoscutal mid-lobe length/width (width measured in anterior part) 1.0, mid-lobe with complete median groove, with six adnotaular setae on each side, lengths mesoscutum/mesoscutellum (measured medially) 1.2, mesoscutellum length/width 1.0, length/width of enclosed space between submedian grooves 2.4, distance between SMG/distance between SMG and SLG 1.7, lengths dorsellum/propodeum 0.5, propodeum with strong reticulation, propodeal callus with six setae. *Fore wing*. Costal cell length/width 10.0, lengths costal cell/marginal vein 0.9, lengths marginal/stigmal veins 3.0. *Gaster*. Elongate-acuminate, length/width 1.7, lengths gaster/head+mesosoma 0.8, Gt_7_ length/width 0.6, length of longest cercal seta/to next longest seta 1.8, longest cercal seta almost straight, ovipositor sheaths reach slightly beyond apex of Gt_7_.

Colour. Body with weak metallic blue tinges, entire antenna dark brown, tegulae black, wings hyaline with veins yellowish-brown, coxae and femora concolorous with body, trochanters black, tibiae and tarsi yellowish-brown.

MALE. Unknown.

#### Diagnosis

Mouth opening 1.4–1.5× malar space; scape 0.9× as long as eye. Similar to *T. crioceridis*, differs only in having F2 and antennal clava longer.

#### Distribution

France and Romania.

#### Ecology

##### Host

Unknown.

#### Notes

Holotype deposited in NHM, paratypes in MZLU, NHM, GD.

### Tetrastichus legionarius

Giraud, 1863

C1EB98BF-9E1F-5DFF-9FBC-459E81D56126

Tetrastichus legionarius Giraud 1863:1273. Lectotype ♀ in NMW, designated by Domenichini 1966:95, not examined. Transferred to *Aprostocetus* by Graham 1961:38-39 and back to *Tetrastichus* by Domenichini 1966:95. Fig. 73

#### Description

See Graham (1991).

#### Diagnosis

Female gaster very long, 2.6–3.3× as long as wide, with Gt_7_ 1.5–1.9× as long as wide; female antenna with funiculars elongate, F1 4.1–4.3×, F2 3.2–3.9×, F3 2.8–3.1× as long as wide; male scape with ventral plaque 0.6–0.7× length of scape, whorled setae of funiculars not reaching to the tips of funiculars attached to; eye height 1.0× malar space in both sexes; both sexes with relatively bright metallic blue colour.

#### Distribution

Austria, (former) Czechoslovakia, France, Hungary, Italy, The Netherlands, Spain (Graham 1991), Sweden and United Kingdom (**new records**).

#### Ecology

##### Host

Gregarious endoparasitoid of larvae and pupae of *Lipara lucens* Meigen (Diptera: Chloropidae) (Graham 1991).

##### Material examined

Non-type material (95♀ 17♂): Austria 1♀ (UCRC), France 16♀ 2♂ (GD, MZLU, NHM), Sweden 69♀ 15♂ (NHM, MZLU, ZSM), United Kingdom 9♀ (NHM).

### Tetrastichus leionotus

Graham, 1991

191E4EE3-3792-5F02-80CC-DDE729BD58A4

Tetrastichus leionotus *Tetrastichus leionotusGraham 1991*^74^

#### Description

See Graham (1991). Male is unknown.

#### Diagnosis

Antenna with scape and pedicel yellowish-brown; frons with a median longitudinal carina that reaches above the middle of frons (as in Fig. 3b); thoracic dorsum strongly shiny with very weak reticulation; propodeal plicae missing in anterior half.

#### Distribution

France (Graham 1991).

#### Ecology

##### Host

Unknown.

##### Material examined

Holotype ♀ (NHM, type no. 5.3612).

### Tetrastichus leocrates

(Walker, 1839)

C0B40766-484B-5E53-ACDA-5BD13194E0B7

Cirrospilus Walker 1839a:319. Lectotype ♂ in NHM, designated by Graham 1961:38, examined (Fig. 75). Transferred to *Tetrastichus* by Walker 1848:150, to *Aprostocetus* by Graham 1961:38 and back to *Tetrastichus* by Domenichini 1966:90.

#### Description

See Graham (1991).

#### Diagnosis

The female is similar to the female of *T. miser* and difficult to separate by morphology; male antenna is without whorls of setae on F1–F4, which separates it from males of the very similar *T. miser*, in which males have these whorls.

#### Distribution

Denmark, Finland, France, Italy, Spain, United Kingdom (Graham 1991) and Sweden (Hedqvist 2003).

#### Ecology

##### Host

*Rhynchaenus alni* (L.) (Graham 1991), *R. testaceus* (Müller) (**new record**) (Coleoptera: Curculionidae).

##### Material examined

Type material: lectotype ♀ of *C. leocrates* (NHM, type no. 5.1938). Additional material: Sweden 19♀ 21♂ (NHM, MZLU, SMTP, ZSM).

### Tetrastichus leptosoma

Graham, 1991

431DA2D8-BD7F-5D67-B976-E27A2E14C3C1

Tetrastichus leptosoma Graham 1991:253. Holotype ♀ in NHM, examined (Fig. 76).

#### Description

See Graham (1991). Male is unknown.

#### Diagnosis

Female antenna with sensilla on F1–F3 sparse, in one (sometimes irregular) row on each funicular; clava without a constriction between C1 and C2.

#### Distribution

(Former) Czechoslovakia, France (Graham 1991).

#### Ecology

##### Host

Unknown.

##### Material examined

Holotype ♀ of *T. leptosoma* (NHM, type no. 5.3622).

### Tetrastichus lyridice

(Walker, 1839)

29558ECD-DD4C-5752-BA31-C15486E3EBE0

Tetrastichus lyridice *Cirrospilus Lyridice Walker 1839a*Graham 1961^77^*Tetrastichus*Walker 1848*Aprostocetus*Graham 1961*Tetrastichus*Domenichini 1966

#### Description

See Graham (1991).

#### Diagnosis

Female antenna with pedicel+flagellum 1.3–1.4× width of mesoscutum, setae of flagellum long and standing out at a greater angle, clava with a distinct constriction between C1 and C2 and 0.9× as long as F2+F3; male scape with short ventral plaque, 0.5× as long as length of scape.

#### Distribution

The Netherlands, United Kingdom (Graham 1991), Sweden (Hedqvist 2003), France and Romania (**new records**).

#### Ecology

##### Host

*Plagiodera versicolora* (Laicharting) (Coleoptera: Chrysomelidae). This record is doubtful, needs checking (Graham 1991).

##### Material examined

Type material: lectotype ♀ of *C. lyridice* (NHM, type no. 5.1939). Additional material (45♀ 5♂): France 2♀ (NHM), Romania 2♀ (NHM), Sweden 37♀ 5♂ (NHM, MZLU, ZSM), United Kingdom 4♀ (NHM).

### Tetrastichus macrops

(Graham, 1961)

D762A768-B9EB-55AA-9884-A755058A734A

Aprostocetus macrops Graham 1961:9–10. Holotype ♀ in OUMNH, examined (Fig. 78). Transferred to *Tetrastichus* by Domenichini 1966:94.

#### Description

See Graham (1961), Graham (1991). The male is unknown.

#### Diagnosis

Eyes relatively large, separated by a distance of 1.1–1.3× the length of an eye and with narrow temples; antenna with clava 0.7–0.9× as long as F2+F3.

#### Distribution

The Netherlands and United Kingdom (Graham 1991).

#### Biology

##### Host

Probably *Cis* spp. (Coleoptera: Cisiidae) in Polyporaceae (Graham 1991).

#### Notes

##### Material examined

Type material: holotype ♀ of *A. macrops* (OUMNH). Additional material (2♀): United Kingdom 2♀ (NHM).

### Tetrastichus melasomae

Graham, 1991

129DB571-9DBD-5F42-8EDE-494FB40B322C

Tetrastichus melasomae *Tetrastichus melasomaeGraham 1991*^79^

#### Description

See Graham (1991). Male is unknown.

#### Diagnosis

Fore wing with costal cell very narrow, 13–17× as long as broad; antenna with claval spine about 0.5× length of C3; scape yellow; body bright green to blue.

#### Distribution

(Former) Czechoslovakia (Graham 1991) and Sweden (Hedqvist 2003).

#### Ecology

##### Host

*Chrysomela vigintipunctata* (Scopoli) (Coleoptera: Chrysomelidae) (Graham 1991).

##### Material examined

Holotype ♀ of *T. melasomae* (NHM, type no. 5.3630).

### Tetrastichus minius sp. n.

B748D62A-B15D-5142-9046-078A5DE95612

urn:lsid:zoobank.org:act:4EA5E6D5-D906-428F-8729-2B49B1943754

#### Materials

- **Type status:** Holotype. **Occurrence:** occurrenceDetails: http://www.boldsystems.org/index.php/API_Public/specimen?ids=BC-ZSM-HYM-27768-A10; catalogNumber: BC-ZSM-HYM-27768-A10; recordNumber: BC-ZSM-HYM-27768-A10; recordedBy: J.S. Noyes; individualID: BC-ZSM-HYM-27768-A10; individualCount: 1; sex: female; lifeStage: adult; **Taxon:** scientificName: Tetrastichus minius; phylum: Arthropoda; class: Insecta; order: Hymenoptera; family: Eulophidae; genus: Tetrastichinae; taxonRemarks: Holotype deposited in NHM; **Location:** country: Romania; decimalLatitude: 47.011; decimalLongitude: 27.603; **Record Level:** type: PhysicalObject; language: en; institutionCode: NHM; basisOfRecord: PreservedSpecimen
- **Type status:** Paratype. **Occurrence:** occurrenceDetails: http://www.boldsystems.org/index.php/API_Public/specimen?ids=BC-ZSM-HYM-27768-B08; catalogNumber: BC-ZSM-HYM-27768-B08; recordNumber: BC-ZSM-HYM-27768-B08; recordedBy: J.S. Noyes; individualID: BC-ZSM-HYM-27768-B08; individualCount: 1; sex: female; lifeStage: adult; **Taxon:** scientificName: Tetrastichus minius; phylum: Arthropoda; class: Insecta; order: Hymenoptera; family: Eulophidae; genus: Tetrastichinae; **Location:** country: Romania; decimalLatitude: 47.011; decimalLongitude: 27.603; **Record Level:** type: PhysicalObject; language: en; institutionCode: NHM; basisOfRecord: PreservedSpecimen

#### Description

FEMALE holotype (Fig. 80). Body length 1.4 mm (paratype 1.3 mm). *Head.* Width/length in dorsal view 2.2, width/length in frontal view 1.3, POL/OOL 1.7, widths head/mesosoma 1.2, mouth width/malar space 1.4, malar space/eye height 0.7. *Antenna.* Scape length/eye height 1.0, pedicel+flagellum length/mesosoma width 1.2, length/width F1, F2, F3 1.4, 1.1, 1.1, clava length/width 2.2, lengths pedicel/F1 0.9, lengths F1/F2 1.1, F1/F3 1.1, lengths F1, F2, F3/clava 0.4, 0.4, 0.4, widths F1/pedicel (dorsal view) 1.1, lengths antennal spicule/C3 0.3. *Mesosoma.* Length/width 1.3, mesoscutal mid-lobe length/width 0.9 (width measured in anterior part), mid-lobe with a weak median groove in posterior ½, with three adnotaular setae on either side, length of mesoscutum/mesoscutellum (measured medially) 1.3, lengths dorsellum/propodeum 0.4, mesoscutellum length/width 0.8, length/width of enclosed space between submedian grooves 2.3, distance between SMG/distance between SMG and SLG 1.3, propodeum with weak reticulation, propodeal callus with five setae. *Fore wing.* Costal cell length/width 12.5, lengths costal cell/marginal vein 1.1, lengths marginal/stigmal veins 2.9. *Gaster.* Short ovate, length/width shrivelled and difficult to measure, but appears slightly longer than wide, lengths gaster/mesosoma nm, Gt_7_ length/width 0.3, length of longest cercal seta/next longest seta 1.7, longest cercal seta sinuate, ovipositor sheaths not reaching apex of Gt_7_.

Colour. Body dark brown to black, partly with weak metallic tinges, antenna dark brown, tegulae black with metallic tinges, wings hyaline with venation dark yellowish-brown, coxae, trochanters and femora concolorous with body, tibiae and tarsi dark yellowish-brown.

MALE. Unknown.

#### Diagnosis

Mouth opening 1.4× malar space; female flagellum short, for example, F3 1.1× as long as wide and clava 2.2× as long as wide; body dark brown black with metallic tinges. Similar to *T. polyporinus*, see key for characters to separate.

#### Distribution

Romania.

#### Ecology

##### Host

Unknown.

#### Notes

Holotype deposited in NHM, paratype in NHM.

### Tetrastichus miser

(Nees, 1834)

90BA099B-F7C7-591A-B640-6B44429E8E6C

Tetrastichus miser *EulophusmiserNees von Esenbeck 1834*Graham 1991^81^*Tetrastichus*Walker 1848*Aprostocetus*Graham 1961*Tetrastichus*Domenichini 1966
Cirrospilus Attalus Walker 1839b:353. Lectotype ♀ in NHM, designated by Graham 1961:37, examined. Synonymised by Walker 1848:145.
Entedon medianus Ratzeburg 1848:169. Lectotype ♂ in NMW, designated by Graham 1991:257, not examined. Synonymised by Domenichini 1966:93.

#### Description

See Graham (1991).

#### Diagnosis

Female with gaster at most 1.6× as long as wide and with Gt_7_ transverse; male with whorled setae on F1–F4 at most reaching to apex of funicular attached to, or slightly beyond apex. See key for delimitation from similar species.

#### Distribution

Austria, (former) Czechoslovakia, Denmark, Finland, France, Germany, Hungary, Ireland, The Netherlands, Spain, Sweden, United Kingdom and (former) Yugoslavia (Graham 1991).

#### Ecology

##### Host

*Rhynchaenus alni* (L.), *R. fagi* (L.), *R. pilosus* (F.), *R. quercus* (F.), *R. salicis* (L.), *Ramphus oxyacanthae* (Marsham) (Coleoptera: Curculionidae) (Graham 1991).

##### Material examined

Type material: neotype ♀ of *E. miser* (NHM, type no. 5.1933), lectotype of *C. Attalus* (NHM, type no. 5.1933). Additional material: Sweden 124♀ 13♂ (NHM, MZLU, ZSM), of which 12♀ 6♂ were reared from *Ramphus oxyacanthae*.

### Tetrastichus nataliedaleskeyae sp. n.

F9331AB2-55DB-5190-9E64-FDF305268B98

urn:lsid:zoobank.org:act:87DB0533-3BE8-4712-89B2-30655FB2CA03

#### Materials

- **Type status:** Holotype. **Occurrence:** occurrenceDetails: http://www.boldsystems.org/index.php/API_Public/specimen?ids=BC-ZSM-HYM-27768-D04; catalogNumber: BC-ZSM-HYM-27768-D04; recordNumber: BC-ZSM-HYM-27768-D04; recordedBy: J.S. Noyes; individualID: BC-ZSM-HYM-27768-D04; individualCount: 1; sex: female; lifeStage: adult; **Taxon:** scientificName: Tetrastichus nataliedaleskeyae; phylum: Arthropoda; class: Insecta; order: Hymenoptera; family: Eulophidae; genus: Tetrastichinae; taxonRemarks: Holotype deposited in NHM; **Location:** country: Romania; decimalLatitude: 46.985; decimalLongitude: 27.585; **Record Level:** type: PhysicalObject; language: en; institutionCode: NHM; basisOfRecord: PreservedSpecimen
- **Type status:** Paratype. **Occurrence:** occurrenceDetails: http://www.boldsystems.org/index.php/API_Public/specimen?ids=BC-ZSM-HYM-27770-H06; catalogNumber: BC-ZSM-HYM-27760-H06; recordNumber: BC-ZSM-HYM-27760-H06; recordedBy: N. Dale-Skey; individualID: BC-ZSM-HYM-27770-H06; individualCount: 1; sex: female; lifeStage: adult; **Taxon:** scientificName: Tetrastichus nataliedaleskeyae; phylum: Arthropoda; class: Insecta; order: Hymenoptera; family: Eulophidae; genus: Tetrastichinae; **Location:** country: United Kingdom; decimalLatitude: 51.4669; decimalLongitude: 0.2358; **Record Level:** type: PhysicalObject; language: en; institutionCode: NHM; basisOfRecord: PreservedSpecimen

#### Description

FEMALE holotype (Fig. 82). Body length 1.7 mm (paratype 1.4–2.0 mm). *Head*. Width/length in dorsal view 2.5, width/length in frontal view 1.3, POL/OOL 2.2, widths head/mesosoma 1.1, mouth width/malar space 1.0, malar space/eye height 0.7. *Antenna*. Scape length/eye height 0.9, pedicel+flagellum length/mesosoma width 1.3, length/width F1, F2, F3 2.3, 2.0, 1.7, clava length/width 3.3, lengths pedicel/F1 0.6, lengths F1/F2 1.0, F1/F3 1.1, lengths F1, F2, F3/clava 0.5, 0.6, 0.5, widths F1/pedicel (dorsal view) 1.1, lengths antennal spicule/C3 0.2. *Mesosoma*. Length/width 1.4, mesoscutal mid-lobe length/width 1.0 (width measured in anterior part), mid-lobe with a median groove in posterior ⅔, with three adnotaular setae on each side, lengths mesoscutum/mesoscutellum (measured medially) 1.3, mesoscutellum length/width 1.0, length/width of enclosed space between submedian grooves 2.4, distance between SMG/distance between SMG and SLG 1.6, lengths dorsellum/propodeum (measured medially) 0.6, propodeal callus with four setae. *Fore wing*. Costal cell length/width 9.0, lengths costal cell/marginal vein 0.9, lengths marginal/stigmal veins 2.8. *Gaster*. Ovate, length/width 1.5, lengths gaster/mesosoma 1.1, Gt_7_ length/width 0.5, length of longest cercal seta/next longest seta 1.6, longest cercal seta curved, ovipositor sheaths reach apex of Gt_7_, but not beyond.

Colour. Head and mesosoma with metallic bluish-green tinges, gaster golden-green, entire antenna dark brown, tegulae black, wings hyaline with venation yellowish-white, coxae and femora concolorous with body, trochanters black, tibiae dark yellowish-brown, fore tarsus dark yellowish-brown, mid and hind tarsi yellowish-white with T4 dark brown.

MALE. Unknown.

#### Diagnosis

Similar to *T. leocrates*, but with gaster and Gt_7_ shorter; also similar to *T. sinope*, but with longer distance between submedian grooves on mesoscutellum and to *T. ballotus*, but with shorter antennal clava and shorter marginal vein in fore wing.

#### Etymology

Named after Natalie Dale-Skey, curator of the Hymenoptera section at the NHM, who collected one type specimen and for having been a great help with logistics at the NHM.

#### Distribution

Romania and United Kingdom.

#### Ecology

##### Host

Unknown.

#### Notes

Holotype and parytype deposited in NHM.

### Tetrastichus pachycerus

Graham, 1991

110B2006-ED0C-54E0-8C35-A031F9C5C78E

Tetrastichus pachycerus *Tetrastichus pachycerusGraham 1991*^83^

#### Description

See Graham (1991).

#### Diagnosis

Ocellar triangle encircled by groove; vertex with numerous piliferous punctures; antennal flagellum stout, distinctly wider than width of pedicel, clava with apex blunt; F1 2.2–2.4× as long as wide; funiculars strongly hairy and with sensilla usually in three rows; large species, 2.4–3.1 mm.

#### Distribution

(Former) Czechoslovakia, France, United Kingdom (Graham 1991), Norway and Sweden (**new records**).

#### Ecology

##### Host

Unknown.

#### Material examined

Type material: holotype ♀ of *T. pachycerus* (NHM, type no. 5.3621). Additional material (10♀): France 1♀ (NHM), Norway 2♀ (NHM), Sweden 5♀ (MZLU, NHM, NHRS), United Kingdom 2♀ (NHM).

### Tetrastichus paululus

Graham, 1991

B5F15FF0-8301-506F-A5C9-A34DD8CB930A

Tetrastichus paululus *Tetrastichus paululusGraham 1991*^84^

#### Description

See Graham (1991). Male is unknown.

#### Diagnosis

Antennal scape, tibiae and wing veins dark brown to black; gaster lanceolate 2.4–2.9× as long as wide with Gt_7_ 1.1–1.5× as long as wide.

#### Distribution

France and United Kingdom (Graham 1991).

#### Ecology

##### Host

Unknown.

##### Material examined

Holotype ♀ of *T. paululus* (NHM, type no. 5.3623).

### Tetrastichus perkinsorum

Graham, 1991

3832A42C-7846-5667-8C50-E9CD54FFF148

Tetrastichus perkinsorum Graham 1991:234. Holotype ♀ in NHM, examined (Fig. 85).

#### Description

See Graham (1991).

#### Diagnosis

Mouth opening 1.4× malar space; antenna with scape 1.2× as long as an eye, funiculars 1.5–1.6× as long as wide.

#### Distribution

Sweden (Graham 1991).

#### Ecology

##### Host

Unknown.

##### Material examined

Holotype ♀ of *T. perkinsorum* (NHM, type no. 5.3624).

### Tetrastichus polyporinus

Askew, 2007

536C6E4C-8C06-50E2-B59C-F2CDA3FE256A

Tetrastichus polyporinus Askew 2007:233-234. Holotype ♀ in NHM, examined (Fig. 86).

#### Description

Female, see Askew (2007).

MALE. Body length 1.7 mm. *Head*. Width/length in dorsal view 2.6, width/length in frontal view 1.4, eye height/malar space 1.5, mouth width/malar space 1.5, widths head/mesosoma 1.1. *Antenna*. F1–F4 with basal whorls of setae, reaching beyond apex of corresponding flagellomere, scape length/eye height 1.0, scape length/width 2.5, ventral plaque placed in central part of scape, lengths ventral plaque/scape 0.7, pedicel+flagellum length/mesosoma width 1.5, length/width F1, F2, F3, F4 1.7, 1.9, 2.0, 2.0, clava length/width 4.9, lengths pedicel/F1 0.8, lengths F1/F2 0.9, F1/F3 0.9, F1/F4 0.9, lengths F1, F2, F3, F4/clava 0.4, 0.4, 0.5, 0.5.

Colour. Body with weak metallic blue tinges, entire antenna dark brown, tegulae dark brown, wing venation yellowish-brown to brown, coxae concolorous with body, trochanters and femora dark brown, tibiae and tarsi yellowish-brown.

#### Diagnosis

Mouth opening 1.3–1.5× malar space; female antenna: flagellum short, for example, F3 at most 1.1× as long as wide, sometimes transverse and clava (incl. spicule) 2.5× as long as wide; male antenna: length of whorled setae on funiculars at least 1.4× the length of funicular attached to; F4 2.0× as long as wide; body black with very weak blue tinges.

#### Distribution

France, Germany (Askew 2007), Romania and Sweden (**new records**).

#### Ecology

##### Host

Possibly *Dacne* sp. (Askew 2007); *Triplax rufipes* Fabricius and *T. russica* (L.) **new records**. All hosts are Coleoptera: Erotylidae.

##### Material examined

Type material: holotype ♀ (NHM, type no. 5.4457). Additional material (23♀ 3♂): France 11♀ 3♂ ex *Triplax russica* from *Polyporus* sp. on *Fraxinus* (G), Romania 2♀ (MZLU, NHM), Sweden 10♀ ex *Triplax rufipes* (NHM).

### Tetrastichus scardiae sp. n.

02163611-210D-54FF-9082-163668545ECF

urn:lsid:zoobank.org:act:12F1A2BE-914B-4B5B-86EE-18147639557A

#### Materials

- **Type status:** Holotype. **Occurrence:** occurrenceDetails: http://www.boldsystems.org/index.php/API_Public/specimen?ids=BC-ZSM-HYM-25461-B08; catalogNumber: BC-ZSM-HYM-25461-B08; recordNumber: BC-ZSM-HYM-25461-B08; recordedBy: H. Lappalainen; individualID: BC-ZSM-HYM-25461-B08; individualCount: 1; lifeStage: adult; **Taxon:** scientificName: Tetrastichus scardiae; phylum: Arthropoda; class: Insecta; order: Hymenoptera; family: Eulophidae; genus: Tetrastichinae; taxonRemarks: Holotype deposited in ZSM; **Location:** country: Finland; decimalLatitude: 62.601; decimalLongitude: 29.759; **Record Level:** type: PhysicalObject; language: en; institutionCode: ZSM; basisOfRecord: PreservedSpecimen
- **Type status:** Paratype. **Occurrence:** occurrenceDetails: http://www.boldsystems.org/index.php/API_Public/specimen?ids=BC-ZSM-HYM-25461-B12; catalogNumber: BC-ZSM-HYM-25461-B12; recordNumber: BC-ZSM-HYM-25461-B12; recordedBy: H. Lappalainen; individualID: BC-ZSM-HYM-25461-B12; individualCount: 1; lifeStage: adult; **Taxon:** scientificName: Tetrastichus scardiae; phylum: Arthropoda; class: Insecta; order: Hymenoptera; family: Eulophidae; genus: Tetrastichinae; **Location:** country: Finland; decimalLatitude: 62.601; decimalLongitude: 29.759; **Record Level:** type: PhysicalObject; language: en; basisOfRecord: PreservedSpecimen
- **Type status:** Paratype. **Occurrence:** occurrenceDetails: http://www.boldsystems.org/index.php/API_Public/specimen?ids=BC-ZSM-HYM-25461-B11; catalogNumber: BC-ZSM-HYM-25461-B11; recordNumber: BC-ZSM-HYM-25461-B11; recordedBy: H. Lappalainen; individualID: BC-ZSM-HYM-25461-B11; individualCount: 1; lifeStage: adult; **Taxon:** scientificName: Tetrastichus scardiae; phylum: Arthropoda; class: Insecta; order: Hymenoptera; family: Eulophidae; genus: Tetrastichinae; **Location:** country: Finland; decimalLatitude: 62.601; decimalLongitude: 29.759; **Record Level:** type: PhysicalObject; language: en; basisOfRecord: PreservedSpecimen
- **Type status:** Paratype. **Occurrence:** occurrenceDetails: http://www.boldsystems.org/index.php/API_Public/specimen?ids=BC-ZSM-HYM-25461-B07; catalogNumber: BC-ZSM-HYM-25461-B07; recordNumber: BC-ZSM-HYM-25461-B07; recordedBy: H. Lappalainen; individualID: BC-ZSM-HYM-25461-B07; individualCount: 1; lifeStage: adult; **Taxon:** scientificName: Tetrastichus scardiae; phylum: Arthropoda; class: Insecta; order: Hymenoptera; family: Eulophidae; genus: Tetrastichinae; **Location:** country: Finland; decimalLatitude: 62.601; decimalLongitude: 29.759; **Record Level:** type: PhysicalObject; language: en; basisOfRecord: PreservedSpecimen
- **Type status:** Paratype. **Occurrence:** occurrenceDetails: http://www.boldsystems.org/index.php/API_Public/specimen?ids=BC-ZSM-HYM-25461-B05; catalogNumber: BC-ZSM-HYM-25461-B05; recordNumber: BC-ZSM-HYM-25461-B05; recordedBy: H. Lappalainen; individualID: BC-ZSM-HYM-25461-B05; individualCount: 1; lifeStage: adult; **Taxon:** scientificName: Tetrastichus scardiae; phylum: Arthropoda; class: Insecta; order: Hymenoptera; family: Eulophidae; genus: Tetrastichinae; **Location:** country: Finland; decimalLatitude: 62.601; decimalLongitude: 29.759; **Record Level:** type: PhysicalObject; language: en; basisOfRecord: PreservedSpecimen
- **Type status:** Paratype. **Occurrence:** occurrenceDetails: http://www.boldsystems.org/index.php/API_Public/specimen?ids=BC-ZSM-HYM-25461-B04; catalogNumber: BC-ZSM-HYM-25461-B04; recordNumber: BC-ZSM-HYM-25461-B04; recordedBy: H. Lappalainen; individualID: BC-ZSM-HYM-25461-B04; individualCount: 1; lifeStage: adult; **Taxon:** scientificName: Tetrastichus scardiae; phylum: Arthropoda; class: Insecta; order: Hymenoptera; family: Eulophidae; genus: Tetrastichinae; **Location:** country: Finland; decimalLatitude: 62.601; decimalLongitude: 29.759; **Record Level:** type: PhysicalObject; language: en; basisOfRecord: PreservedSpecimen
- **Type status:** Paratype. **Occurrence:** occurrenceDetails: http://www.boldsystems.org/index.php/API_Public/specimen?ids=BC-ZSM-HYM-25461-B03; catalogNumber: BC-ZSM-HYM-25461-B03; recordNumber: BC-ZSM-HYM-25461-B03; recordedBy: H. Lappalainen; individualID: BC-ZSM-HYM-25461-B03; individualCount: 1; lifeStage: adult; **Taxon:** scientificName: Tetrastichus scardiae; phylum: Arthropoda; class: Insecta; order: Hymenoptera; family: Eulophidae; genus: Tetrastichinae; **Location:** country: Finland; decimalLatitude: 62.601; decimalLongitude: 29.759; **Record Level:** type: PhysicalObject; language: en; basisOfRecord: PreservedSpecimen
- **Type status:** Paratype. **Occurrence:** occurrenceDetails: http://www.boldsystems.org/index.php/API_Public/specimen?ids=BC-ZSM-HYM-25461-B02; catalogNumber: BC-ZSM-HYM-25461-B02; recordNumber: BC-ZSM-HYM-25461-B02; recordedBy: H. Lappalainen; individualID: BC-ZSM-HYM-25461-B02; individualCount: 1; lifeStage: adult; **Taxon:** scientificName: Tetrastichus scardiae; phylum: Arthropoda; class: Insecta; order: Hymenoptera; family: Eulophidae; genus: Tetrastichinae; **Location:** country: Finland; decimalLatitude: 62.601; decimalLongitude: 29.759; **Record Level:** type: PhysicalObject; language: en; basisOfRecord: PreservedSpecimen
- **Type status:** Paratype. **Occurrence:** occurrenceDetails: http://www.boldsystems.org/index.php/API_Public/specimen?ids=BC-ZSM-HYM-25461-B01; catalogNumber: BC-ZSM-HYM-25461-B01; recordNumber: BC-ZSM-HYM-25461-B01; recordedBy: H. Lappalainen; individualID: BC-ZSM-HYM-25461-B01; individualCount: 1; lifeStage: adult; **Taxon:** scientificName: Tetrastichus scardiae; phylum: Arthropoda; class: Insecta; order: Hymenoptera; family: Eulophidae; genus: Tetrastichinae; **Location:** country: Finland; decimalLatitude: 62.601; decimalLongitude: 29.759; **Record Level:** type: PhysicalObject; language: en; basisOfRecord: PreservedSpecimen
- **Type status:** Paratype. **Occurrence:** occurrenceDetails: http://www.boldsystems.org/index.php/API_Public/specimen?ids=BC-ZSM-HYM-25461-A12; catalogNumber: BC-ZSM-HYM-25461-A12; recordNumber: BC-ZSM-HYM-25461-A12; recordedBy: H. Lappalainen; individualID: BC-ZSM-HYM-25461-A12; individualCount: 1; lifeStage: adult; **Taxon:** scientificName: Tetrastichus scardiae; phylum: Arthropoda; class: Insecta; order: Hymenoptera; family: Eulophidae; genus: Tetrastichinae; **Location:** country: Finland; decimalLatitude: 62.601; decimalLongitude: 29.759; **Record Level:** type: PhysicalObject; language: en; basisOfRecord: PreservedSpecimen
- **Type status:** Paratype. **Occurrence:** occurrenceDetails: http://www.boldsystems.org/index.php/API_Public/specimen?ids=BC-ZSM-HYM-25461-A11; catalogNumber: BC-ZSM-HYM-25461-A11; recordNumber: BC-ZSM-HYM-25461-A11; recordedBy: H. Lappalainen; individualID: BC-ZSM-HYM-25461-A11; individualCount: 1; lifeStage: adult; **Taxon:** scientificName: Tetrastichus scardiae; phylum: Arthropoda; class: Insecta; order: Hymenoptera; family: Eulophidae; genus: Tetrastichinae; **Location:** country: Finland; decimalLatitude: 62.601; decimalLongitude: 29.759; **Record Level:** type: PhysicalObject; language: en; basisOfRecord: PreservedSpecimen
- **Type status:** Paratype. **Occurrence:** occurrenceDetails: http://www.boldsystems.org/index.php/API_Public/specimen?ids=BC-ZSM-HYM-25461-A10; catalogNumber: BC-ZSM-HYM-25461-A10; recordNumber: BC-ZSM-HYM-25461-A10; recordedBy: H. Lappalainen; individualID: BC-ZSM-HYM-25461-A10; individualCount: 1; lifeStage: adult; **Taxon:** scientificName: Tetrastichus scardiae; phylum: Arthropoda; class: Insecta; order: Hymenoptera; family: Eulophidae; genus: Tetrastichinae; **Location:** country: Finland; decimalLatitude: 62.601; decimalLongitude: 29.759; **Record Level:** type: PhysicalObject; language: en; basisOfRecord: PreservedSpecimen
- **Type status:** Paratype. **Occurrence:** occurrenceDetails: http://www.boldsystems.org/index.php/API_Public/specimen?ids=BC-ZSM-HYM-25461-A09; catalogNumber: BC-ZSM-HYM-25461-A09; recordNumber: BC-ZSM-HYM-25461-A09; recordedBy: H. Lappalainen; individualID: BC-ZSM-HYM-25461-A09; individualCount: 1; lifeStage: adult; **Taxon:** scientificName: Tetrastichus scardiae; phylum: Arthropoda; class: Insecta; order: Hymenoptera; family: Eulophidae; genus: Tetrastichinae; **Location:** country: Finland; decimalLatitude: 62.601; decimalLongitude: 29.759; **Record Level:** type: PhysicalObject; language: en; basisOfRecord: PreservedSpecimen
- **Type status:** Paratype. **Occurrence:** occurrenceDetails: http://www.boldsystems.org/index.php/API_Public/specimen?ids=BC-ZSM-HYM-25461-A08; catalogNumber: BC-ZSM-HYM-25461-A08; recordNumber: BC-ZSM-HYM-25461-A08; recordedBy: H. Lappalainen; individualID: BC-ZSM-HYM-25461-A08; individualCount: 1; lifeStage: adult; **Taxon:** scientificName: Tetrastichus scardiae; phylum: Arthropoda; class: Insecta; order: Hymenoptera; family: Eulophidae; genus: Tetrastichinae; **Location:** country: Finland; decimalLatitude: 62.601; decimalLongitude: 29.759; **Record Level:** type: PhysicalObject; language: en; basisOfRecord: PreservedSpecimen
- **Type status:** Paratype. **Occurrence:** occurrenceDetails: http://www.boldsystems.org/index.php/API_Public/specimen?ids=BC-ZSM-HYM-25461-A07; catalogNumber: BC-ZSM-HYM-25461-A07; recordNumber: BC-ZSM-HYM-25461-A07; recordedBy: H. Lappalainen; individualID: BC-ZSM-HYM-25461-A07; individualCount: 1; lifeStage: adult; **Taxon:** scientificName: Tetrastichus scardiae; phylum: Arthropoda; class: Insecta; order: Hymenoptera; family: Eulophidae; genus: Tetrastichinae; **Location:** country: Finland; decimalLatitude: 62.601; decimalLongitude: 29.759; **Record Level:** type: PhysicalObject; language: en; basisOfRecord: PreservedSpecimen
- **Type status:** Paratype. **Occurrence:** occurrenceDetails: http://www.boldsystems.org/index.php/API_Public/specimen?ids=BC-ZSM-HYM-25461-A05; catalogNumber: BC-ZSM-HYM-25461-A05; recordNumber: BC-ZSM-HYM-25461-A05; recordedBy: H. Lappalainen; individualID: BC-ZSM-HYM-25461-A05; individualCount: 1; lifeStage: adult; **Taxon:** scientificName: Tetrastichus scardiae; phylum: Arthropoda; class: Insecta; order: Hymenoptera; family: Eulophidae; genus: Tetrastichinae; **Location:** country: Finland; decimalLatitude: 62.601; decimalLongitude: 29.759; **Record Level:** type: PhysicalObject; language: en; basisOfRecord: PreservedSpecimen
- **Type status:** Paratype. **Occurrence:** occurrenceDetails: http://www.boldsystems.org/index.php/API_Public/specimen?ids=BC-ZSM-HYM-25461-B10; catalogNumber: BC-ZSM-HYM-25461-B10; recordNumber: BC-ZSM-HYM-25461-B10; recordedBy: H. Lappalainen; individualID: BC-ZSM-HYM-25461-B10; individualCount: 1; lifeStage: adult; **Taxon:** scientificName: Tetrastichus scardiae; phylum: Arthropoda; class: Insecta; order: Hymenoptera; family: Eulophidae; genus: Tetrastichinae; **Location:** country: Finland; decimalLatitude: 62.601; decimalLongitude: 29.759; **Record Level:** type: PhysicalObject; language: en; basisOfRecord: PreservedSpecimen
- **Type status:** Paratype. **Occurrence:** occurrenceDetails: http://www.boldsystems.org/index.php/API_Public/specimen?ids=BC-ZSM-HYM-25461-B09; catalogNumber: BC-ZSM-HYM-25461-B09; recordNumber: BC-ZSM-HYM-25461-B09; recordedBy: H. Lappalainen; individualID: BC-ZSM-HYM-25461-B09; individualCount: 1; lifeStage: adult; **Taxon:** scientificName: Tetrastichus scardiae; phylum: Arthropoda; class: Insecta; order: Hymenoptera; family: Eulophidae; genus: Tetrastichinae; **Location:** country: Finland; decimalLatitude: 62.601; decimalLongitude: 29.759; **Record Level:** type: PhysicalObject; language: en; basisOfRecord: PreservedSpecimen
- **Type status:** Paratype. **Occurrence:** occurrenceDetails: http://www.boldsystems.org/index.php/API_Public/specimen?ids=BC-ZSM-HYM-25461-B06; catalogNumber: BC-ZSM-HYM-25461-B06; recordNumber: BC-ZSM-HYM-25461-B06; recordedBy: H. Lappalainen; individualID: BC-ZSM-HYM-25461-B06; individualCount: 1; lifeStage: adult; **Taxon:** scientificName: Tetrastichus scardiae; phylum: Arthropoda; class: Insecta; order: Hymenoptera; family: Eulophidae; genus: Tetrastichinae; **Location:** country: Finland; decimalLatitude: 62.601; decimalLongitude: 29.759; **Record Level:** type: PhysicalObject; language: en; basisOfRecord: PreservedSpecimen
- **Type status:** Paratype. **Occurrence:** occurrenceDetails: http://www.boldsystems.org/index.php/API_Public/specimen?ids=BC-ZSM-HYM-25461-A06; catalogNumber: BC-ZSM-HYM-25461-A06; recordNumber: BC-ZSM-HYM-25461-A06; recordedBy: H. Lappalainen; individualID: BC-ZSM-HYM-25461-A06; individualCount: 1; lifeStage: adult; **Taxon:** scientificName: Tetrastichus scardiae; phylum: Arthropoda; class: Insecta; order: Hymenoptera; family: Eulophidae; genus: Tetrastichinae; **Location:** country: Finland; decimalLatitude: 62.601; decimalLongitude: 29.759; **Record Level:** type: PhysicalObject; language: en; basisOfRecord: PreservedSpecimen

#### Description

FEMALE holotype (Fig. 87). Body length 2.3 mm (paratypes 2.0–2.2 mm). *Head*. Width/length in dorsal view 2.0, width/length in frontal view 1.0, POL/OOL 0.9, widths head/mesosoma 0.9, mouth width/malar space 1.3, malar space/eye height 1.1. *Antenna*. Scape length/eye height 1.1, pedicel+flagellum length/mesosoma width 0.9, length/width F1, F2, F3 1.3, 1.3, 1.5, clava length/width 3.1, lengths pedicel/F1 1.4, lengths F1/F2 0.8, F1/F3 0.7, lengths F1, F2, F3/clava 0.3, 0.4, 0.4, widths F1/pedicel (dorsal view) 0.9, lengths antennal spicule/C3 0.3. *Mesosoma*. Length/width 1.4, mesoscutal mid-lobe length/width 0.8 (width measured in anterior part), mid-lobe with median groove in posterior ⅓, with four adnotaular setae on each side, lengths mesoscutum/mesoscutellum (measured medially) 1.3, mesoscutellum length/width 0.8, length/width of enclosed space between submedian grooves 2.6, distance between SMG/distance between SMG and SLG 1.1, lengths dorsellum/propodeum 0.5, propodeum with weak reticulation, propodeal callus with four setae. *Fore wing*. Costal cell length/width 8.2, lengths costal cell/marginal vein 1.1, lengths marginal/stigmal veins 2.4. *Gaster*. Ovate, length/width 1.6, lengths gaster/mesosoma 1.1, Gt_7_ length/width 0.4, length of longest cercal seta/next longest seta 2.0, longest cercal seta strongly curved, ovipositor sheaths reach apex of Gt_7_, but not beyond.

Colour. Body with weak metallic green tinges, scape yellowish-brown, pedicel brownish, flagellum dark brown, tegulae dark brown, wings hyaline with veins yellow-white to brown, coxae and femora concolorous with body, trochanters dark brown, tibiae and tarsi yellowish-brown.

MALE. There are four males in the type series, but all specimens are badly damaged and fragmented and all specimens lack pedicel+flagellum. The antenna holds several diagnostic characters and without them and, as the specimens are broken, it is not possible to give a useful description. However, the male shares the same diagnostic features as the female, except characters in the antenna and it should be possible to recognise the characters through these.

#### Diagnosis

Posterior ocelli close, POL/OOL= 0.9; eyes small and malar space large, length of eye/malar space = 0.9 in female, 1.0 in male; female antenna short, length pedicel+flagellum/width of mesosoma = 0.9, with F1 and F2 ± merged in most specimens (incl. holotype, only two of the paratypes have these flagellomeres separated); submedian grooves on mesoscutellum distinctly converging towards posterior part; mesoscutum and mesoscutellum with weak reticulation and shiny.

#### Distribution

Finland.

#### Ecology

##### Host

*Scardia boletella* (Fabricius) (Lepidoptera: Tineidae). There is no information if all specimens in the type series are from the same host specimen, but as the identical barcode in all specimens indicates that they are from the same clutch (including 20♀ and 4♂), this is probably the case. Thus, this species is a gregarious endoparasitoid.

#### Notes

Holotype deposited in ZSM, paratypes in MZLU, NHM and ZSM.

### Tetrastichus setifer

Thomson, 1878

3E2683D8-8B66-511B-91F8-A16A44FE0C80

Tetrastichus setifer Thomson 1878:283. Lectotype ♀ in MZLU, designated by Graham 1961:39, examined (Fig. 88). Transferred to *Aprostocetus* by Graham 1961:39 and back to *Tetrastichus* by Domenichini 1966:91.

#### Description

Female see Graham (1991).

MALE. Body length 1.9 mm. *Head*. Width/length in dorsal view 2.6, width/length in frontal view 1.3, eye height/malar space 1.2, mouth width/malar space 1.6, widths head/mesosoma 1.0. *Antenna*. F1–F4 with basal whorls of setae, reaching to but not beyond apex of corresponding flagellomere, scape length/eye height 1.1, scape length/width 3.0, ventral plaque placed in central part of scape, lengths ventral plaque/scape 0.8, pedicel+flagellum length/mesosoma width 1.4, length/width F1, F2, F3, F4 1.5, 1.7, 1.8,1.6, clava length/width 4.5, lengths pedicel/F1 0.9, lengths F1/F2 0.9, F1/F3 0.9, F1/F4 1.0, lengths F1, F2, F3, F4/clava 0.4, 0.4, 0.4, 0.4.

Colour. Body with metallic blue tinges, entire antenna dark brown, tegulae dark brown, wing venation pale brown, coxae concolorous with body, trochanters and femora dark brown, tibiae yellowish-brown, tarsi yellowish-brown with T4 brown.

#### Diagnosis

Ovipositor retracted and does not reach apex of Gt_7_; cerci placed laterally; colour dull compared to other species in this group; spine of antennal claval long, 0.5× C3; mouth opening 1.3–1.4× malar space.

#### Distribution

(Former) Czechoslovakia, France, (former) Yugoslavia (Graham 1991) and Sweden (Thomson 1878).

#### Ecology

##### Host

*Lilioceris lilii* (Scopoli) (Gold et al. 2001) and *L. tibialis* (Villa) (Kenis et al. 2002) (Coleoptera: Chrysomelidae).

##### Material examined

Type material: lectotype ♀ of *T. setifer* (MZLU, type no. 4868:1). Additional material (27♀ 5♂): France 2♀ (GD); Sweden 25♀ 5♂ ex *Lilioceris lilii* (MZLU, NHM).

### Tetrastichus sinope

(Walker, 1839)

BDF964AE-D7B3-5CF1-BF3E-D2D7B9B554E8

Tetrastichus sinope *CirrospilussinopeWalker 1839a*Graham 1961^89^*Tetrastichus*Walker 1848*Aprostocetus*Graham 1961*Tetrastichus*Domenichini 1966
Cirrospilus hippis Walker 1839a: 304. Lectotype ♀ in NHM, designated by Graham 1961:38, examined. Synonymised by Graham 1961:38.
Cirrospilus agathocles Walker 1839b: 353. Lectotype ♀ in NHM, designated by Graham 1961:38, examined. Synonymised by Graham 1961:38.
Cirrospilus rapo Walker 1839d: 415. Lectotype ♀ in NHM, designated by Graham 1961:38, examined. Synonymised by Graham 1961:38.

#### Materials

- **Type status:** Other material. **Occurrence:** occurrenceDetails: http://www.boldsystems.org/index.php/API_Public/specimen?ids=BC-ZSM-HYM-21587-A11|BC-ZSM-HYM-26563-A05|BC-ZSM-HYM-20721-D06; catalogNumber: BC-ZSM-HYM-26563-A05; recordNumber: BC-ZSM-HYM-26563-A05; recordedBy: C. Hansson; individualID: BC-ZSM-HYM-26563-A05; individualCount: 1; sex: F; lifeStage: a; associatedMedia: http://www.boldsystems.org/pics/BCHYM/BC-ZSM-HYM-26563-A05+1510087062.jpg; **Taxon:** scientificName: Tetrastichus sinope; phylum: Arthropoda; class: Insecta; order: Hymenoptera; family: Eulophidae; genus: Tetrastichus; **Location:** country: Sweden; locality: Vombs vattenverk; decimalLatitude: 55.6619; decimalLongitude: 13.5472; **Identification:** identifiedBy: Christer Hansson
- **Type status:** Other material. **Occurrence:** occurrenceDetails: http://www.boldsystems.org/index.php/API_Public/specimen?ids=BC-ZSM-HYM-21587-A11|BC-ZSM-HYM-26563-A05|BC-ZSM-HYM-20721-D06; catalogNumber: BC-ZSM-HYM-20721-D06; recordNumber: BC-ZSM-HYM-20721-D06; recordedBy: C. Hansson; individualID: BC-ZSM-HYM-20721-D06; individualCount: 1; sex: F; lifeStage: Adult; associatedMedia: http://www.boldsystems.org/pics/BCHYM/BC-ZSM-HYM-20721-D06+1398631828.jpg; **Taxon:** scientificName: Tetrastichus sinope; phylum: Arthropoda; class: Insecta; order: Hymenoptera; family: Eulophidae; genus: Tetrastichus; **Location:** country: Sweden; locality: Vomb; decimalLatitude: 55.667; decimalLongitude: 13.55; **Identification:** identifiedBy: Christer Hansson; identificationRemarks: CH03417
- **Type status:** Other material. **Occurrence:** occurrenceDetails: http://www.boldsystems.org/index.php/API_Public/specimen?ids=BC-ZSM-HYM-21587-A11|BC-ZSM-HYM-26563-A05|BC-ZSM-HYM-20721-D06; catalogNumber: BC-ZSM-HYM-21587-A11; recordNumber: BC-ZSM-HYM-21587-A11; recordedBy: C.Hansson; individualID: BC-ZSM-HYM-21587-A11; individualCount: 1; sex: F; lifeStage: a; associatedMedia: http://www.boldsystems.org/pics/BCHYM/BC-ZSM-HYM-21587-A11+1423081076.jpg; **Taxon:** scientificName: Tetrastichus sinope; phylum: Arthropoda; class: Insecta; order: Hymenoptera; family: Eulophidae; genus: Tetrastichus; **Location:** country: Sweden; locality: Ismantorp; decimalLatitude: 56.893; decimalLongitude: 16.768; **Identification:** identifiedBy: Christer Hansson

#### Description

See Graham (1991).

#### Diagnosis

Scape, femora, mid and hind tibiae, and wing veins dark brown to black; female gaster 1.4–1.6× as long as wide and with Gt_7_ transverse.

#### Distribution

Ireland, The Netherlands, United Kingdom (Graham 1991), Sweden (Hedqvist 2003) and France (**new record**).

#### Ecology

##### Host

Unknown.

##### Additional material examined

Type material: lectotypes ♀ of *C. sinope* (type no. 5.1935), *C. hippis* (type no. 5.1934), *C. agathocles* (type no. 5.1936), *C. rapo* (type no. 5.1937), all in NHM. Additional material: France, 3♀ (G). Sweden, Skåne & Öland, 3♀ (MZLU).

##### Remarks

In the barcoded nontype material analysed, there are three groups (ACM5794, ADP1347 and ACT4395), each represented by a single female specimen. The barcode strongly indicates that these are three different species, but they are not possible to separate by their morphology. Morphologically, all three specimens agree well with the type of *T. sinope*. Since we do not have any molecular information from the types of, and under, this species, it is not possible to identify these “barcode species” better than to *T. sinope*, even though they probably represent different species. Therefore, we conclude that *T. sinope* is an aggregate of morphologically-inseparable species.

### Tetrastichus sodalis

Graham 1991

34904DE5-4578-53ED-A83A-543102EA841E

Tetrastichus sodalis Graham 1991:256. Holotype ♀ in NHM, examined (Fig. 90).

#### Description

See Graham (1991). Male is unknown.

#### Diagnosis

Mouth 1.4× malar space; antenna with scape 0.9× as long as an eye, F3 2.0–2.4× as long as broad, clava short, 0.8× as long as F2+F3 in holotype.

#### Distribution

(Former) Czechoslovakia and France (Graham 1991).

#### Ecology

##### Host

Unknown.

##### Material examined

Holotype ♀ of *T. sodalis* (NHM, type no. 5.3624).

### Tetrastichus tachos

(Walker, 1839)

F0AEFD76-7152-586F-85C6-99A76DFB56B8

Tetrastichus tachos *CirrospilustachosWalker 1839b*Graham 1961^91^*Tetrastichus*Walker 1848*Aprostocetus*Graham 1961*Tetrastichus*Domenichini 1966

#### Description

See Graham (1991).

#### Diagnosis

Scape in both sexes with numerous (about ten) setae along frontal edge; female with a long antennal flagellum, pedicel+flagellum 1.6× as long as width of mesoscutum; ovipositor sheaths do not reach apex of Gt_7_ ; male antenna with scape and pedicel dark brown almost black, flagellum pale brown.

#### Distribution

(Former) Czechoslovakia, United Kingdom (Graham 1991) and Sweden (Hedqvist 2003).

#### Host

Unknown.

##### Material examined

Type material: lectotype ♂ of *C. tachos* (NHM type no. 5.1942). Additional material: Sweden 1♂ (MZLU).

### Tetrastichus telon

(Graham, 1961)

8F68E9B9-0147-5B5E-B0E7-A6CA13F90163

Tetrastichus telon *AprostocetustelonGraham 1961*^92^*Tetrastichus*Domenichini 1966

#### Description

See Graham (1991), Graham (1961).

#### Diagnosis

Female gaster very long, 2.9–5.0× as long as wide with Gt_7_ 1.6–2.5× as long as wide. The only other species with this long gaster is *T. legionarius*, that differs from *T. telon* in having the body bright metallic bluish-green to green, antennal scape longer than eye and a longer flagellum, about 2.8× the length of scape (about 2.6× in *T. telon*) and F1 about 4× as long as wide (about 2.8× in *T. telon*).

#### Distribution

(Former) Czechoslovakia, France, United Kingdom (Graham 1991), Germany, Italy (Domenichini 1966) and Sweden (Hedqvist 2003).

#### Ecology

##### Host

*Agrilus viridis* L. (Coleoptera: Buprestidae) (Domenichini 1966).

#### Material examined

Type material: holotype ♀ *of A. telon* (OUMNH). Additional material (10♀): France 1♀ (NHM), Sweden 2♀ (MZLU, NHM), United Kingdom 7♀ (NHM).

### Tetrastichus temporalis

(Graham, 1961)

95B2AA4C-F742-5367-91EC-6B881BE5C016

Aprostocetus temporalis Graham 1961:10–11. Holotype ♀ in OUMNH, examined (Fig. 93). Transferred to *Tetrastichus* by Domenichini 1966:95.

#### Description

See Graham (1961), Graham (1991).

#### Diagnosis

Female gaster long, 1.9–2.3× as long as wide, with Gt_7_ 0.9–1.0× as long as wide; female antenna with F1 2.6–2.9×, F2 2.1–2.8×, F3 1.9–2.4× as long as wide; male scape with ventral plaque 0.6–0.7× length of scape, whorled setae of funiculars reaching the tips of funicular attached to or beyond tips; eye height 1.3× malar space in both sexes; both sexes with relatively bright metallic green (usually) or blue (more seldom) colour.

#### Distribution

Sweden, United Kingdom (Graham 1991) and France (**new record**).

#### Ecology

##### Host

Unknown, but according to Graham 1991, possibly associated with *Phalaris arundinacea* (Poaceae).

##### Material examined

Type material: holotype ♀ (OUMNH). Additional material (105♀ 24♂): France 6♀ (NHM), Sweden 68♀ 21♂ (MZLU, NHM, ZSM), United Kingdom 31♀ 3♂ (NHM).

### Tetrastichus theoi

Graham 1991

33D28AA5-E78E-52C1-A6AC-856F5779B931

Tetrastichus theoi Graham 1991:254. Holotype ♀ in Naturalis Biodiversity Center, Leiden, The Netherlands, not examined.Fig. 94

#### Description

See Graham (1991). Male is unknown.

#### Diagnosis

Head 2.1× as long as wide in dorsal view; mesoscutellum with submedian grooves 1.8–2.0× their distance from sublateral grooves; median part of propodeum, between plicae, smooth or with very weak reticulation.

#### Distribution

France (Graham 1991).

#### Ecology

##### Host

Unknown.

##### Material examined

One ♀ paratype from FRANCE, Agay, on *Inula viscosa* (NHM).

### Tetrastichus ulmi

Erdös, 1954

133963C8-E461-5814-A01F-2AD9486066E8

Tetrastichus ulmi *Tetrastichus ulmiErdös 1954Aprostocetus*Graham 1961*Tetrastichus*Domenichini 1966

#### Description

See Graham (1991). The male is unknown.

#### Diagnosis

Similar to *T. agrilocidus*, but with female antenna shorter and stouter (Fig. 95).

#### Distribution

Bulgaria, (former) Czechoslovakia, Hungary, Italy, United Kingdom, (former) Yugoslavia (Graham 1991), France, Germany and Sweden (**new records**).

#### Ecology

##### Host

*Scolytus rugulosus* (Müller) and *Leperisinus orni* Fuchs (Coleoptera: Scolytidae), *Agrilus* sp., and possibly *Anthaxia* sp. (Coleoptera: Buprestidae) (Graham 1991).

#### Material examined

Non-type material (21♀): France 2♀ (GD), Germany 2♀ (ZSM), Hungary 1♀ (NHM, identified as *T. ulmi* by Erdös), Sweden 16♀ (NHM, MZLU, SMTP).

## Identification Keys

### Identification keys to females and males of European species of *Tetrastichus*

**Table:** 

1	Females	see Table 2
–	Males	see Table 3

## Analysis

### Phylogenetic analysis

**Figure 96.**
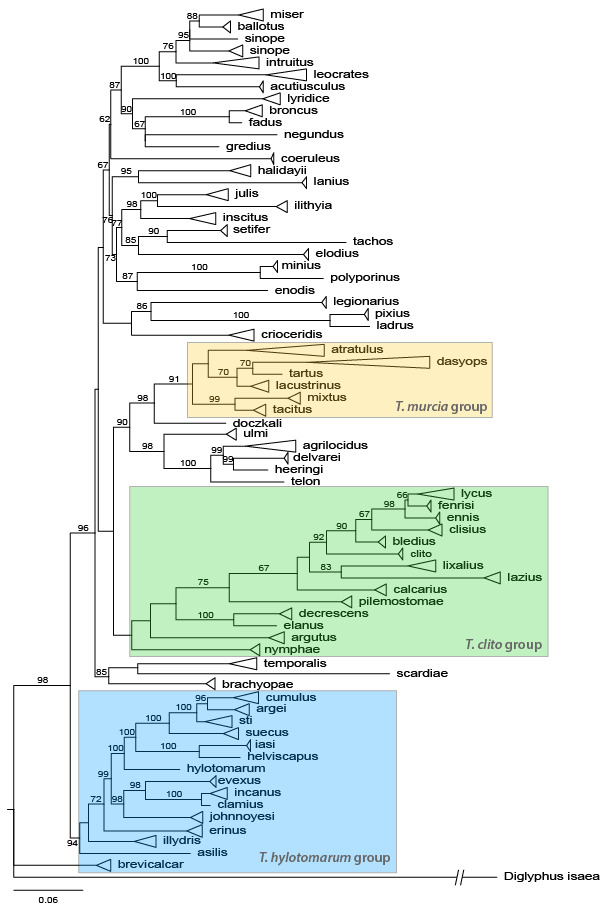
Maximum Likelihood tree, based on COI sequences of 70 species of *Tetrastichus*, with *Diglyphus isaea* as an outgroup taxon. Numbers above branches represent bootstrap support values (only values > 50% are shown). Triangles at terminal branches indicate the presence of two or more specimens for a species. The three species groups that are recognised, based on morphological characters (*T. clito*, *T. hylotomarum* and *T. murcia* groups), are highlighted by different colours.

Of the 93 species, 70 were represented by sequences of at least 500 bp, and the sequences were used for assessing phylogenetic relationships within the genus. The Maximum Likelihood analysis resulted in trees with several distinct clades (Fig. 96), most of which were supported by bootstrap support values of 80% or higher, but only three of them can be defined unequivocally by morphological characters. These are the *Tetrastichus hylotomarum* group, the *T. murcia* group and - albeit with low bootstrap support, the *T. clito* group (Fig. 96).

Species of the *T. clito* group differ from other*Tetrastichus* species in having the frons with a more or less distinct median longitudinal carina that is extending from between the toruli to near the median ocellus, the sutures that define the scrobal area laterally tending to diverge ventrally and the hind coxa having relatively weak sculpture on the outer surface. These characters are shared with other species of the subfamily Tetrastichinae, for example, by species of *Aprostocetus*. Within the *clito* group, there are species with a protruding or a retracted ovipositor, with the latter also occurring in other*Tetrastichus* species, suggesting that it is homoplasious and that the "*T. clito* group" is non-monophyletic. Species of the *T. hylotomarum* group are regarded as monophyletic, despite the disparate placement of *T. brevicalcar* in the tree, because it came out as monophyletic in other tree reconstructions. The group is morphologically characterised by the retracted ovipositor sheaths that do not not reach the apex of the gaster, gaster subcircular to short ovate and colour usually strongly metallic. Species of the *T. murcia* group can be recognised by the eyes usually with long, conspicuous pubescence, setae on hind margin of pronotum and adnotaular setae relatively long and suberect, gaster short ovate to subcircular and body black non-metallic or with weak metallic tinges.

The remaining 41, or about half of the 93 species recognised in the current study, were regarded as unplaced with respect to species groups, due to the absence of supporting morphological characters. We refrained from establishing species groups, based on molecular characters. Despite high bootstrap support values of a clade, based on mitochondrial COI providing support for its monophyly, additional markers, in particular from nuclear genes, are needed for the reliable assessment of phylogenetic relationships within the genus *Tetrastichus*.

The present analysis includes only species of the subfamily Tetrastichinae that were traditionally regarded as species of *Tetrastichus*, based on morphological characters. This genus is morphologically much more readily definable than some other genera of Tetrastichinae, in particular compared to the genus *Aprostocetus* that has been used as a "dump" for species that did not seem to fit elsewhere. European species of *Tetrastichus* can, amongst other characters, be recognised by having a single seta on the submarginal vein and by having branched (Y-shaped) carinae laterally on the propodeum. The morphologically-close genera *Quadrastichus* and *Oomyzus* have also one seta on the submarginal vein, but they lack the Y-shaped carina on the propodeum (but may have a simple longitudinal carina laterally). Females of *Oomyzus* differ from *Quadrastichus* by their antennae, with flagellomeres being short in *Oomyzus* and comparatively long in *Quadrastichus*. In *Tetrastichus*, there are species with females having short or long flagellomeres.

Whereas 46 of the 50 (92%) species described as new are associated (and can be identified) with a DNA barcode, from one third (14) of the 43 described species, no sequences could be obtained and their identification had to rely on morphological characters.

## Supplementary Material

XML Treatment for Tetrastichus

XML Treatment for Tetrastichus murcia group

XML Treatment for Tetrastichus antonjanssoni

XML Treatment for Tetrastichus atratulus

XML Treatment for Tetrastichus brachyopae

XML Treatment for Tetrastichus dasyops

XML Treatment for Tetrastichus intruitus

XML Treatment for Tetrastichus lacustrinus

XML Treatment for Tetrastichus mixtus

XML Treatment for Tetrastichus murcia

XML Treatment for Tetrastichus solvae

XML Treatment for Tetrastichus tacitus

XML Treatment for Tetrastichus tartus

XML Treatment for Tetrastichus hylotomarum group

XML Treatment for Tetrastichus argei

XML Treatment for Tetrastichus asilis

XML Treatment for Tetrastichus brevicalcar

XML Treatment for Tetrastichus calmius

XML Treatment for Tetrastichus coelarchus

XML Treatment for Tetrastichus coeruleus

XML Treatment for Tetrastichus cosidis

XML Treatment for Tetrastichus cumulus

XML Treatment for Tetrastichus cyprus

XML Treatment for Tetrastichus erinus

XML Treatment for Tetrastichus evexus

XML Treatment for Tetrastichus flaccius

XML Treatment for Tetrastichus helviscapus

XML Treatment for Tetrastichus hylotomarum

XML Treatment for Tetrastichus iasi

XML Treatment for Tetrastichus illydris

XML Treatment for Tetrastichus inaequalis

XML Treatment for Tetrastichus incanus

XML Treatment for Tetrastichus johnnoyesi

XML Treatment for Tetrastichus marcusgrahami

XML Treatment for Tetrastichus splendens

XML Treatment for Tetrastichus sti

XML Treatment for Tetrastichus suecus

XML Treatment for Tetrastichus clito group

XML Treatment for Tetrastichus argutus

XML Treatment for Tetrastichus bledius

XML Treatment for Tetrastichus calcarius

XML Treatment for Tetrastichus clisius

XML Treatment for Tetrastichus clito

XML Treatment for Tetrastichus decrescens

XML Treatment for Tetrastichus elanus

XML Treatment for Tetrastichus ennis

XML Treatment for Tetrastichus epilachnae

XML Treatment for Tetrastichus fenrisi

XML Treatment for Tetrastichus ladrus

XML Treatment for Tetrastichus lazius

XML Treatment for Tetrastichus lixalius

XML Treatment for Tetrastichus lycus

XML Treatment for Tetrastichus nymphae

XML Treatment for Tetrastichus pilemostomae

XML Treatment for Tetrastichus pixius

XML Treatment for Tetrastichus unplaced species

XML Treatment for Tetrastichus acutiusculus

XML Treatment for Tetrastichus agonus

XML Treatment for Tetrastichus agrilocidus

XML Treatment for Tetrastichus ballotus

XML Treatment for Tetrastichus broncus

XML Treatment for Tetrastichus crioceridis

XML Treatment for Tetrastichus delvarei

XML Treatment for Tetrastichus doczkali

XML Treatment for Tetrastichus elodius

XML Treatment for Tetrastichus enodis

XML Treatment for Tetrastichus fadus

XML Treatment for Tetrastichus gredius

XML Treatment for Tetrastichus halidayi

XML Treatment for Tetrastichus heeringi

XML Treatment for Tetrastichus heterus

XML Treatment for Tetrastichus ilithyia

XML Treatment for Tetrastichus inscitus

XML Treatment for Tetrastichus julis

XML Treatment for Tetrastichus lanius

XML Treatment for Tetrastichus legionarius

XML Treatment for Tetrastichus leionotus

XML Treatment for Tetrastichus leocrates

XML Treatment for Tetrastichus leptosoma

XML Treatment for Tetrastichus lyridice

XML Treatment for Tetrastichus macrops

XML Treatment for Tetrastichus melasomae

XML Treatment for Tetrastichus minius

XML Treatment for Tetrastichus miser

XML Treatment for Tetrastichus nataliedaleskeyae

XML Treatment for Tetrastichus pachycerus

XML Treatment for Tetrastichus paululus

XML Treatment for Tetrastichus perkinsorum

XML Treatment for Tetrastichus polyporinus

XML Treatment for Tetrastichus scardiae

XML Treatment for Tetrastichus setifer

XML Treatment for Tetrastichus sinope

XML Treatment for Tetrastichus sodalis

XML Treatment for Tetrastichus tachos

XML Treatment for Tetrastichus telon

XML Treatment for Tetrastichus temporalis

XML Treatment for Tetrastichus theoi

XML Treatment for Tetrastichus ulmi

## Figures and Tables

**Figure 2a.**
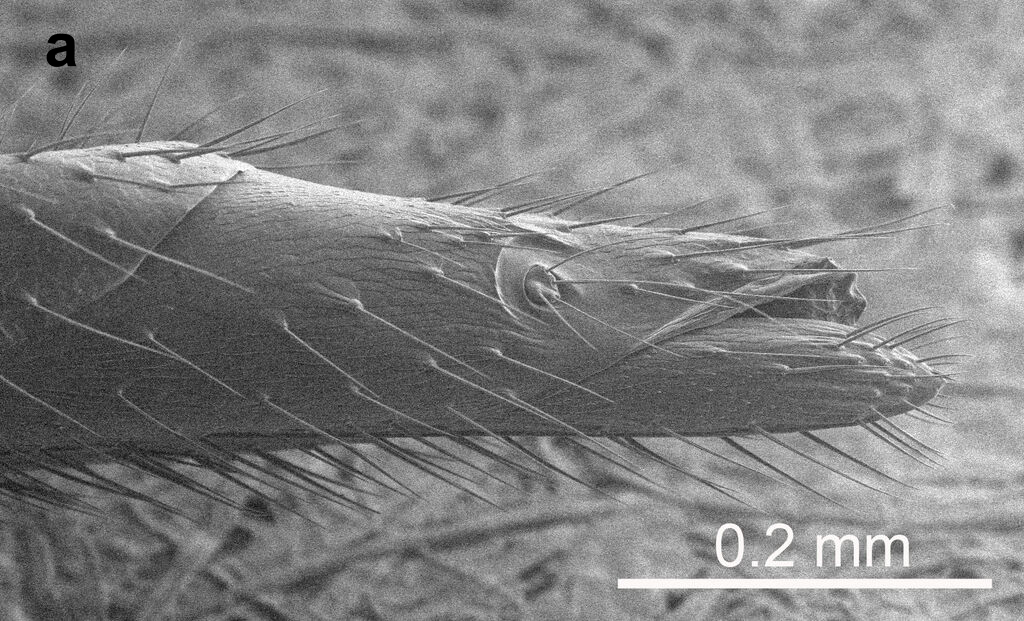
*T. legionarius* female nontype, ovipositor sheaths protruding beyond Gt7, lateral view.

**Figure 2b.**
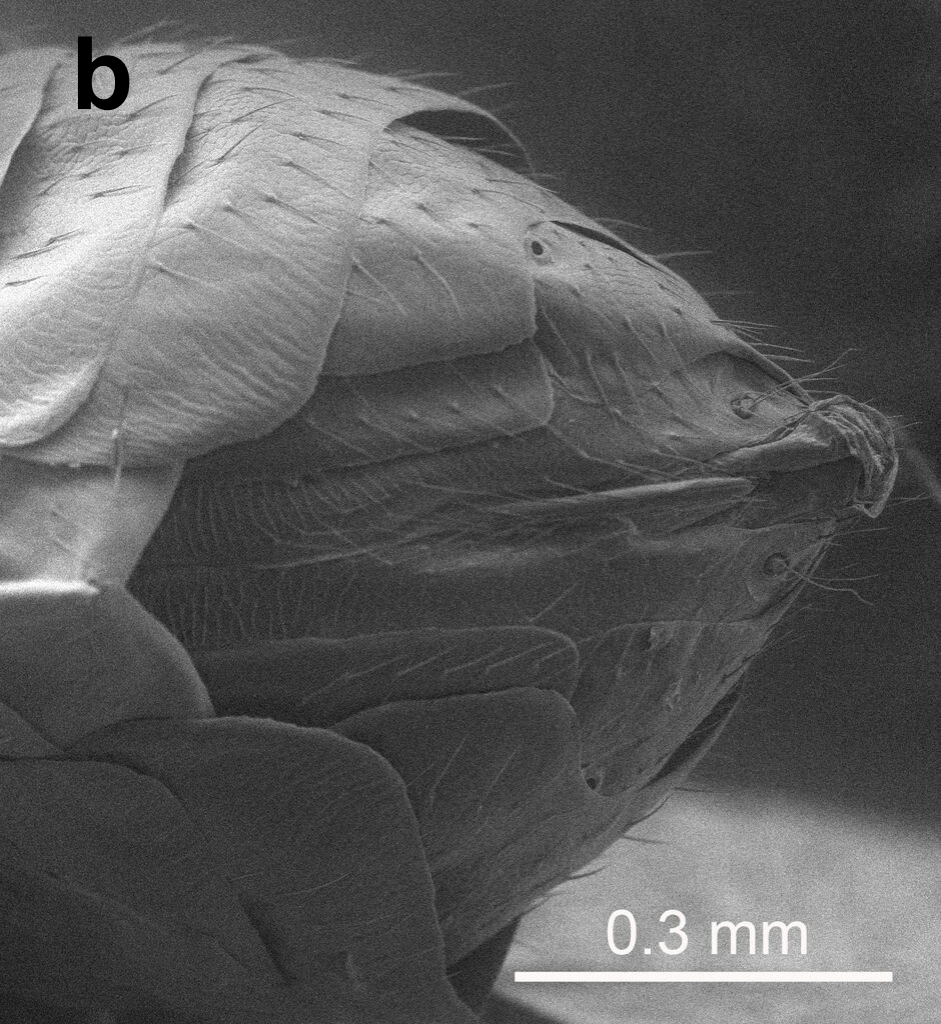
*T. coeruleus* female nontype, ovipositor retracted, ventral view.

**Figure 2c.**
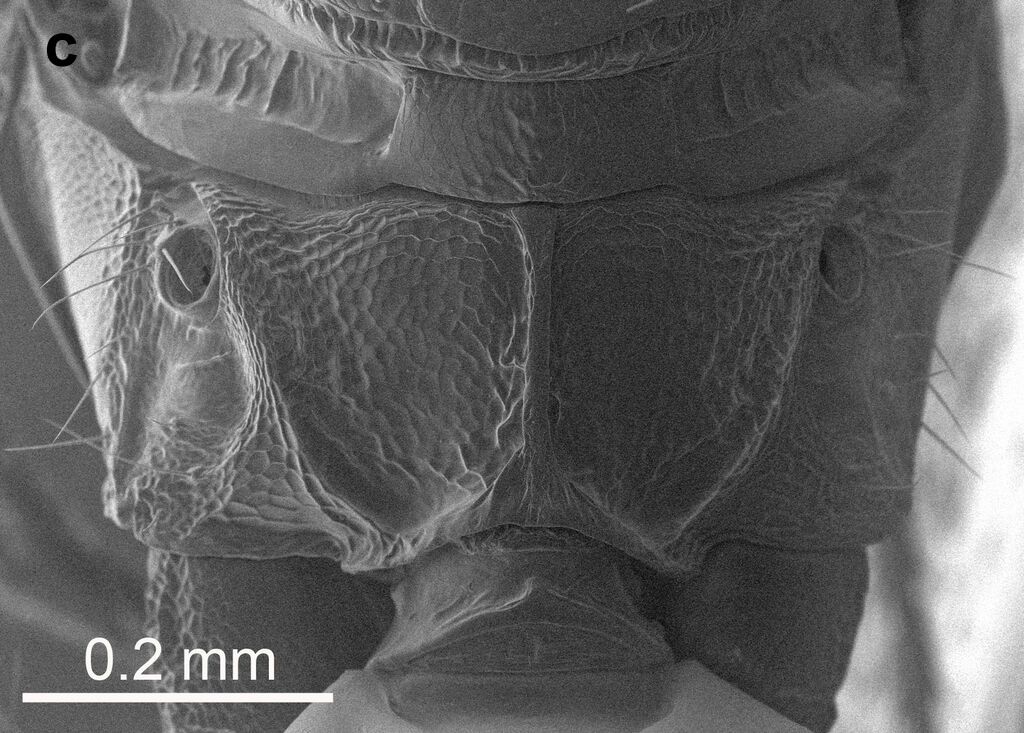
*T. halidayi* female nontype, propodeum, dorsal view.

**Figure 2d.**
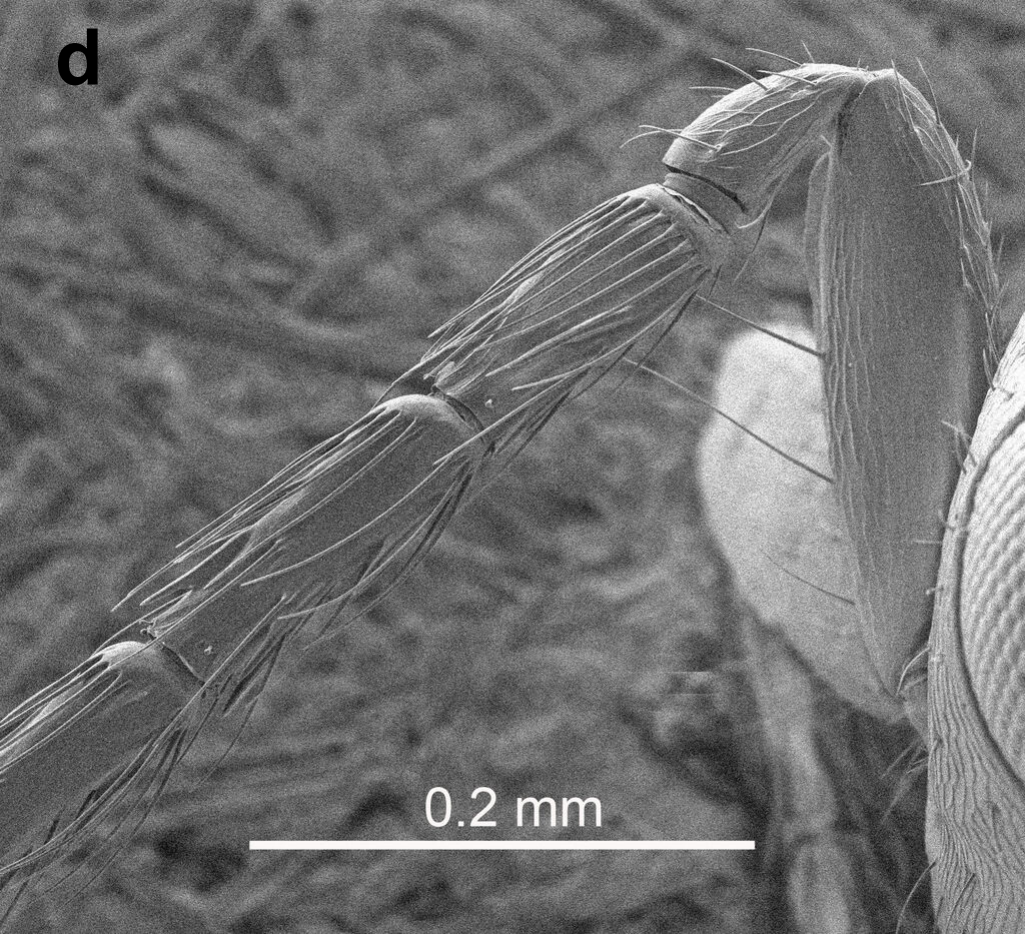
*T. legionarius* male nontype, antennal F1-F3, lateral view.

**Figure 2e.**
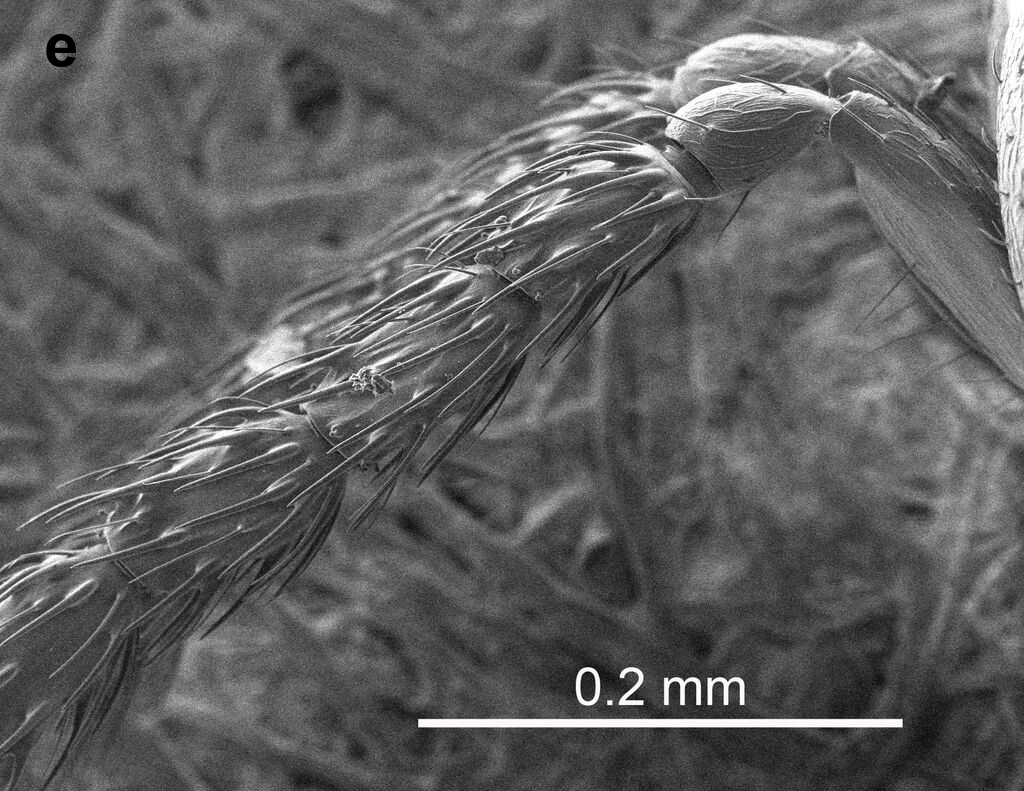
*T. leocrates* male nontype, antennal F1-F3, lateral view.

**Figure 3a.**
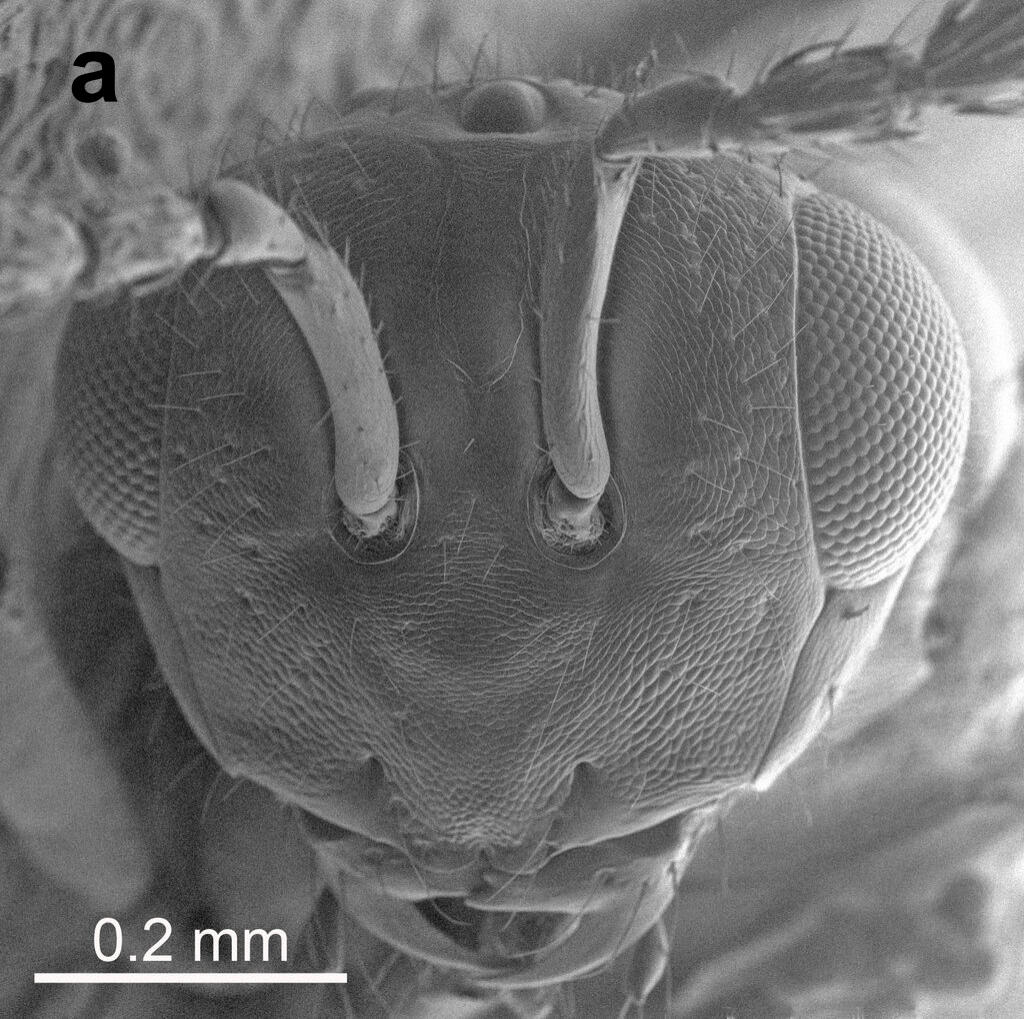
*T. halidayi* female nontype, head, frontal view.

**Figure 3b.**
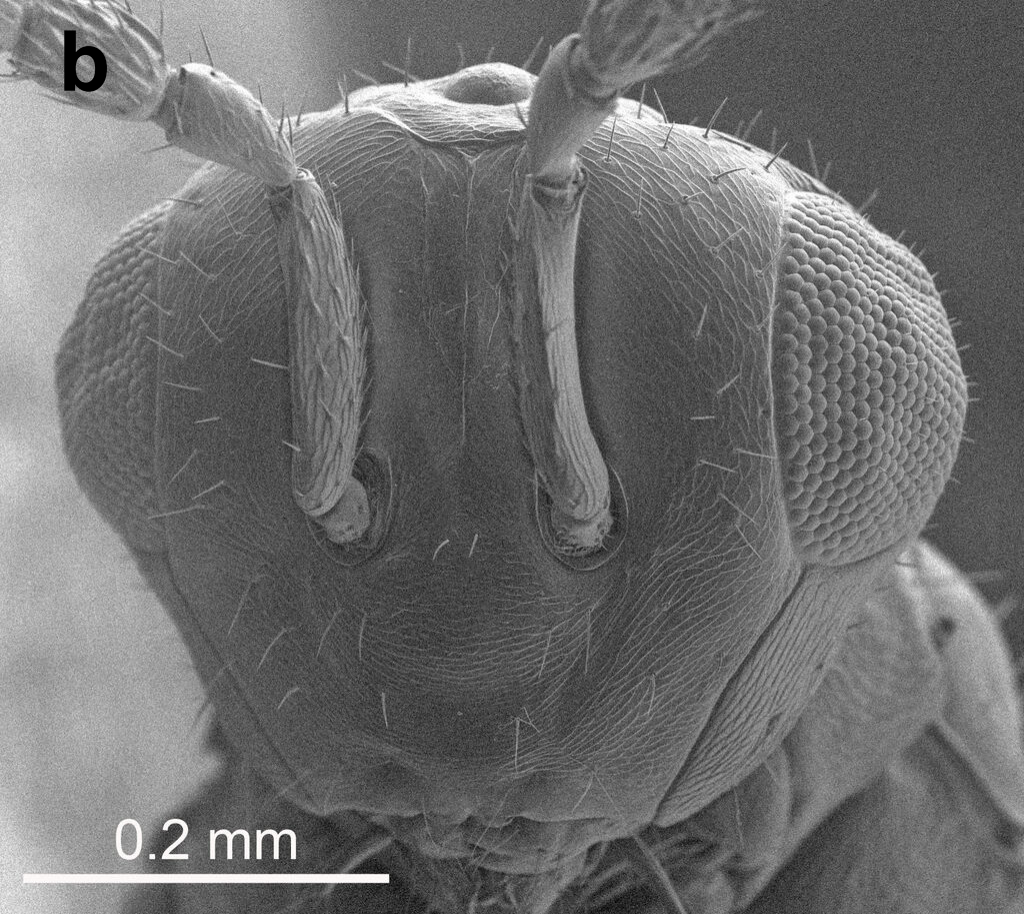
*T. clito* female nontype, head, frontal view.

**Figure 3c.**
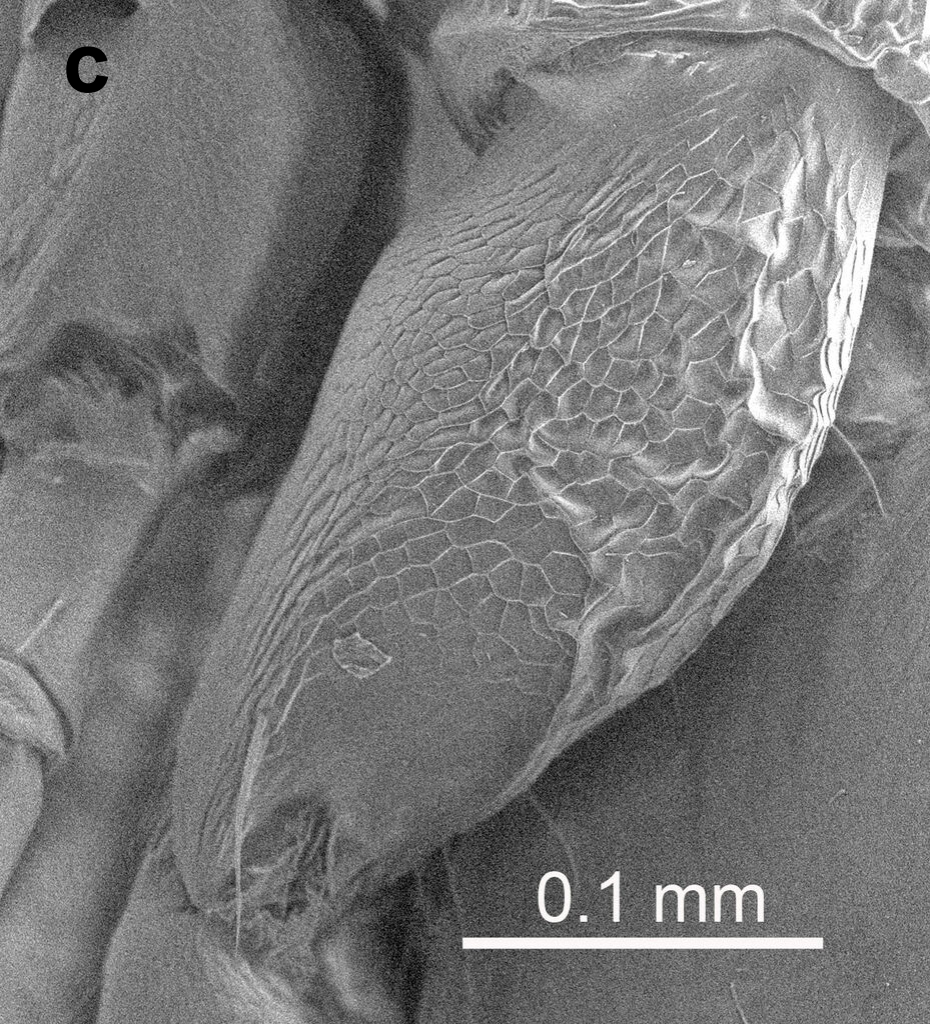
*T. halidayi* female nontype, hind coxa, lateral view.

**Figure 3d.**
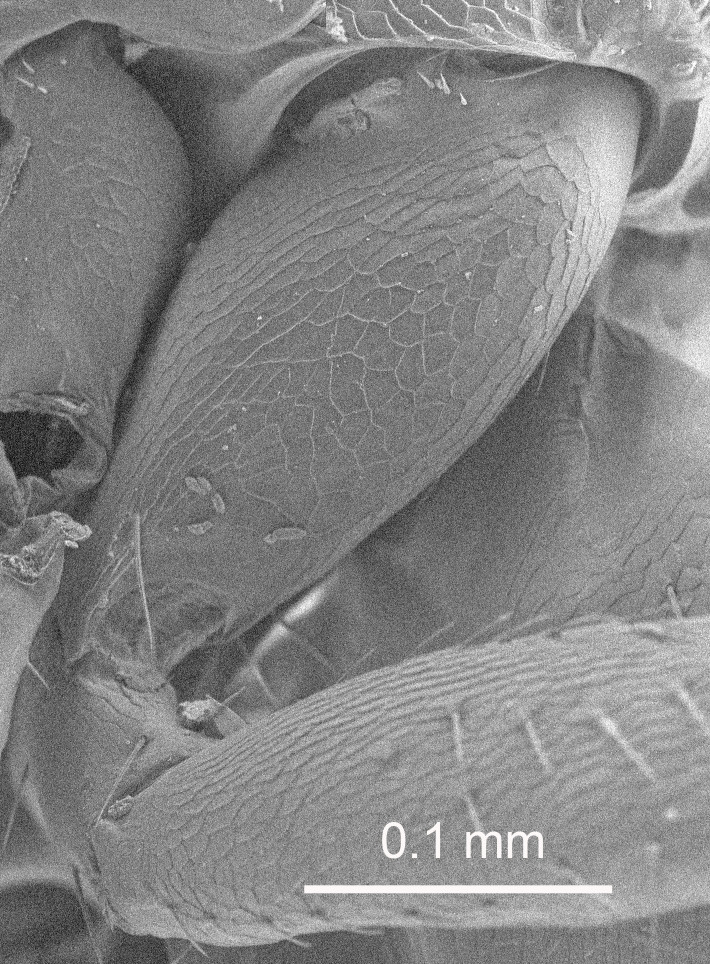
*T. clito* female nontype, hind coxa, lateral view.

**Figure 4a.**
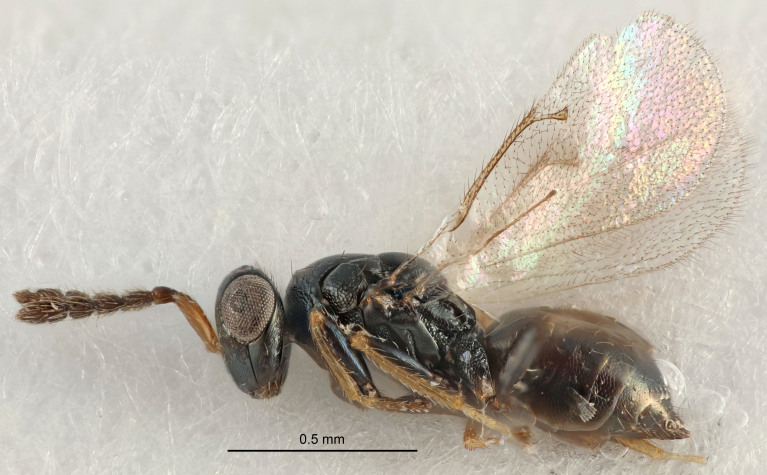
Holotype female, lateral.

**Figure 4b.**
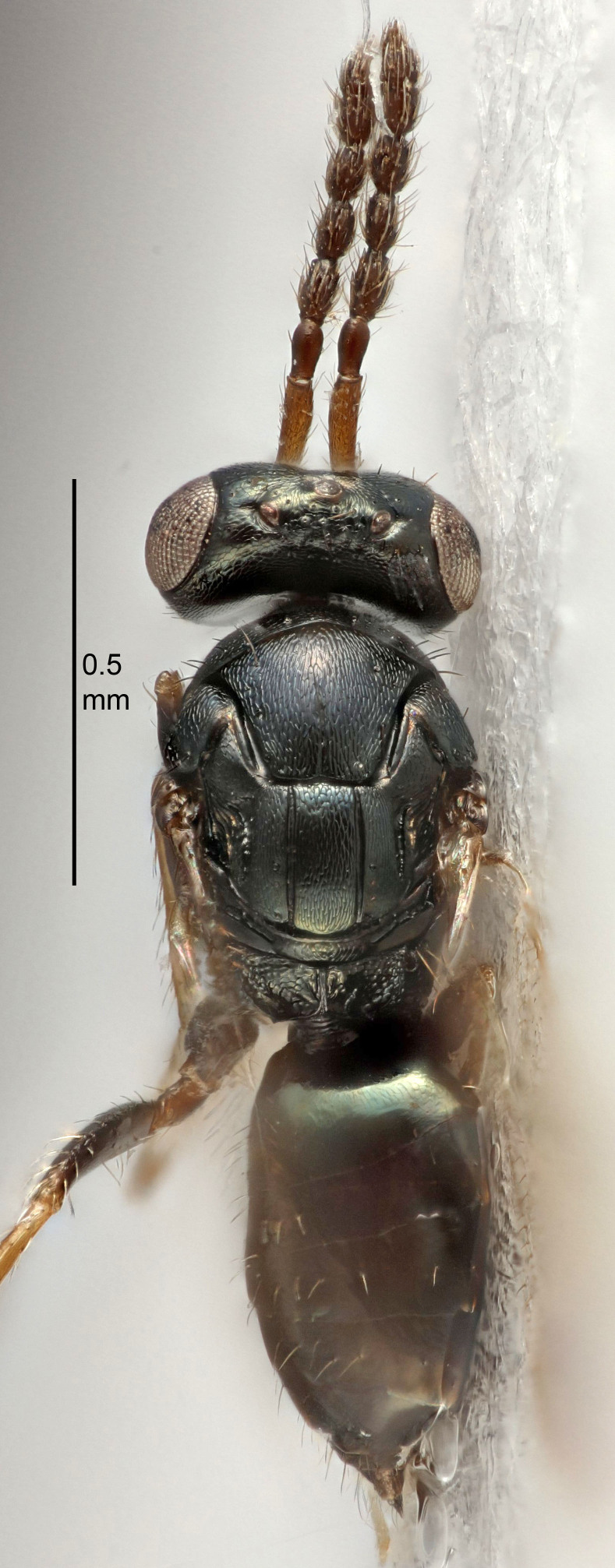
Holotype female, dorsal.

**Figure 5a.**
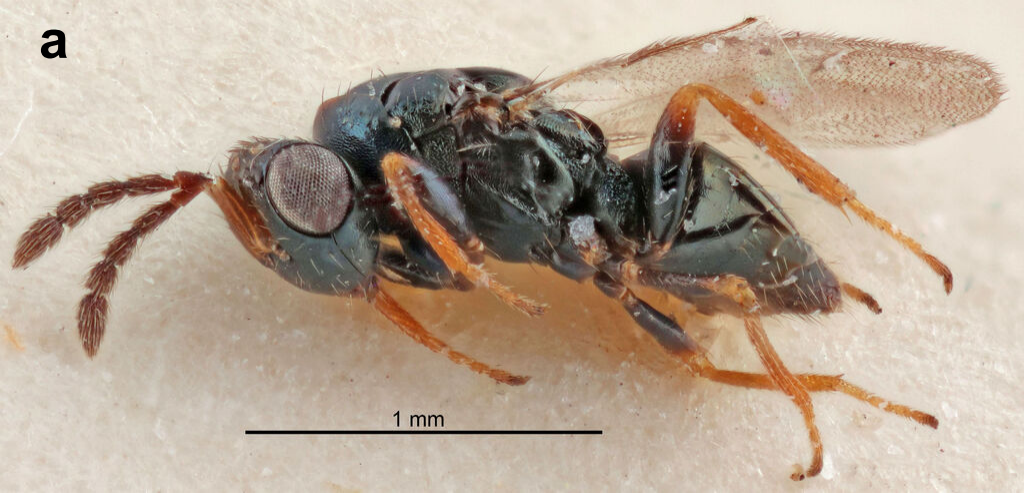
Neotype female, lateral.

**Figure 5b.**
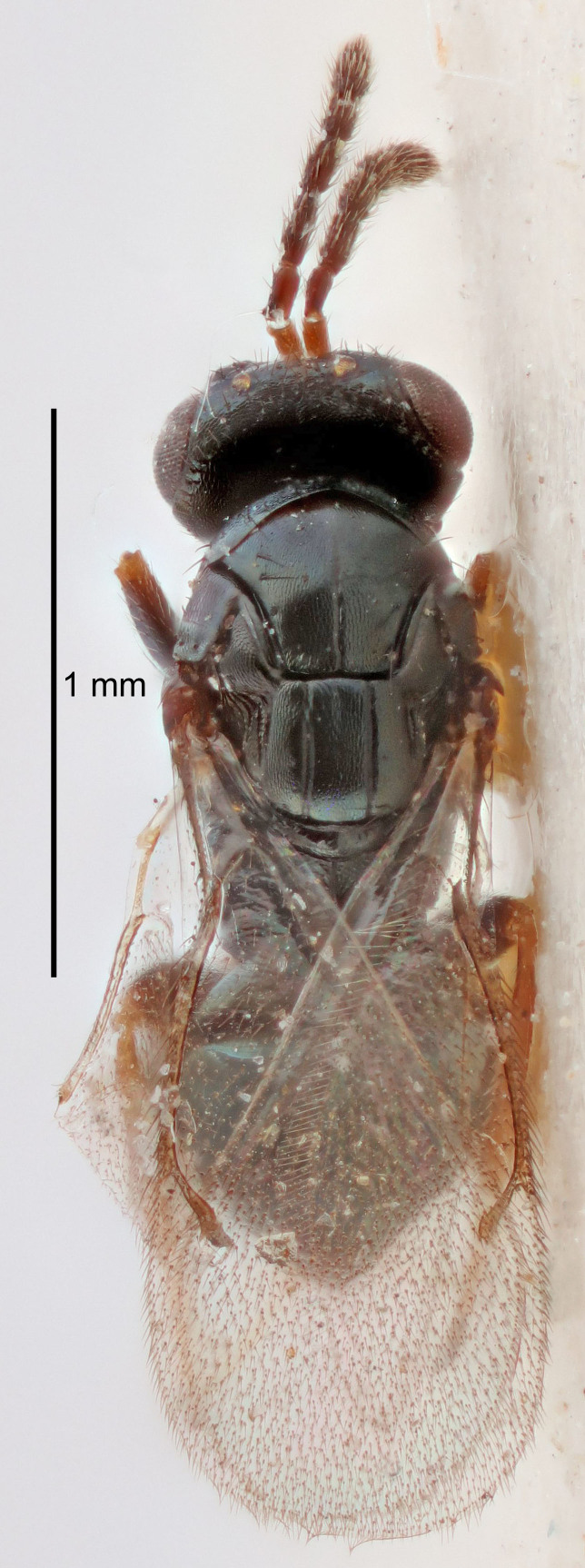
Neotype female, dorsal.

**Figure 5c.**
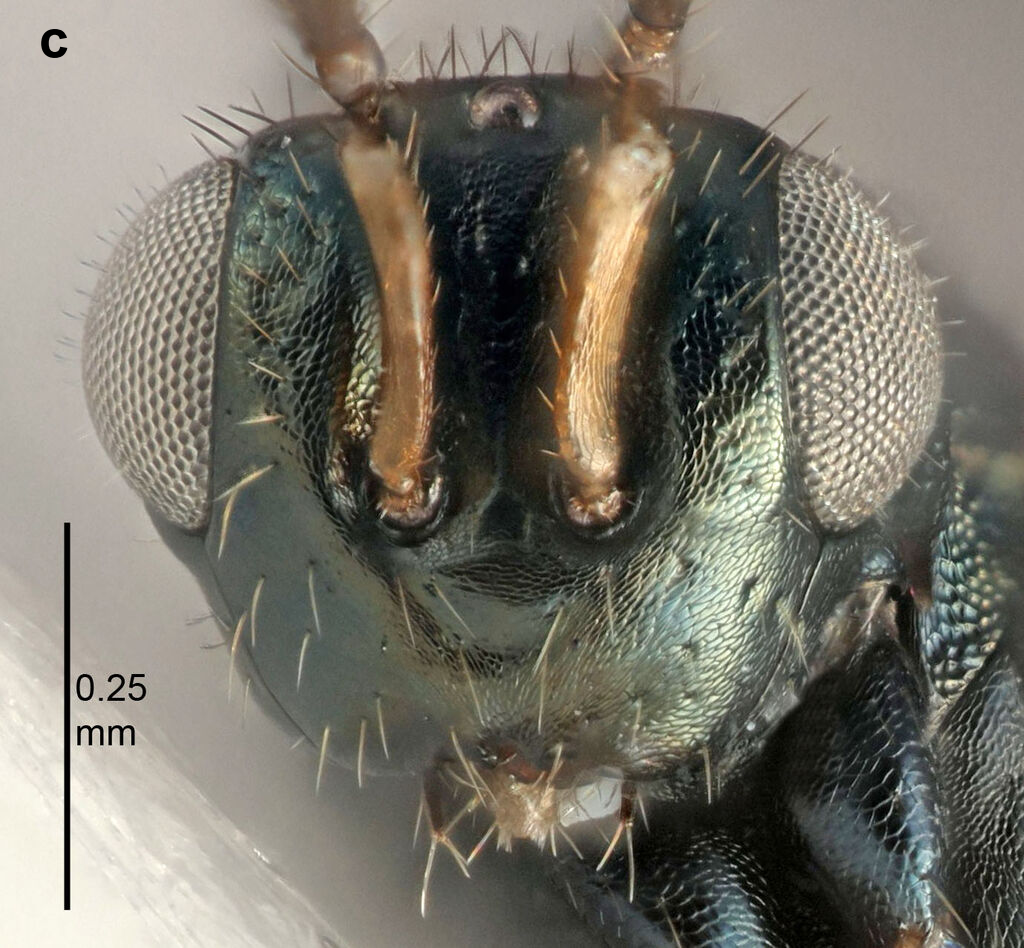
Nontype female, head frontal.

**Figure 5d.**
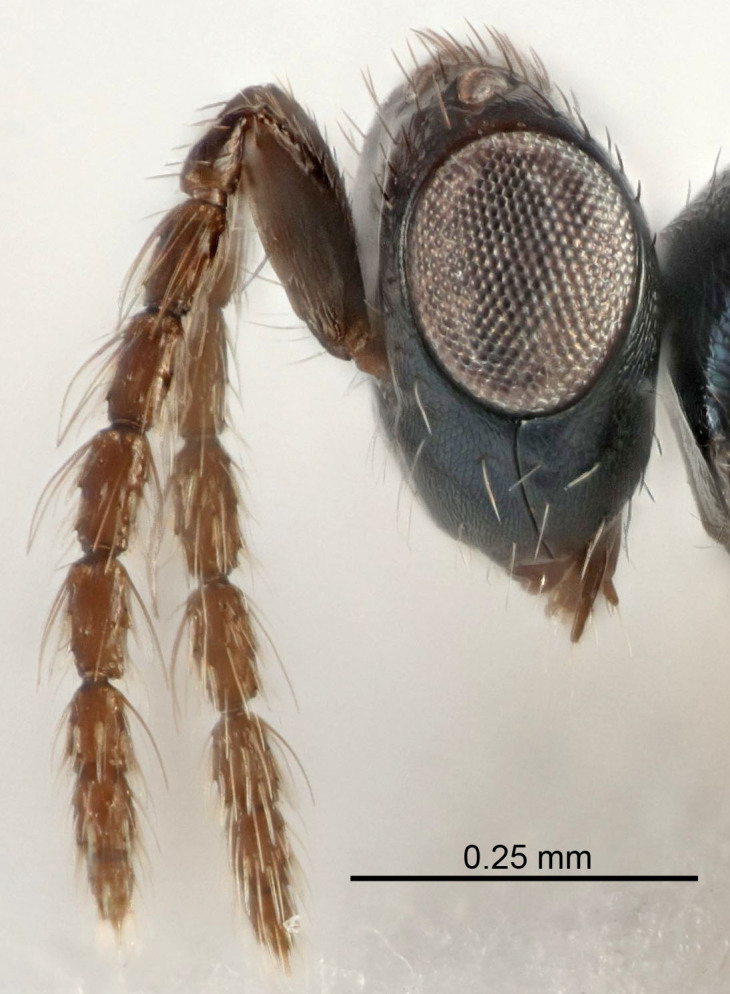
Nontype male, head and antenna lateral.

**Figure 6a.**
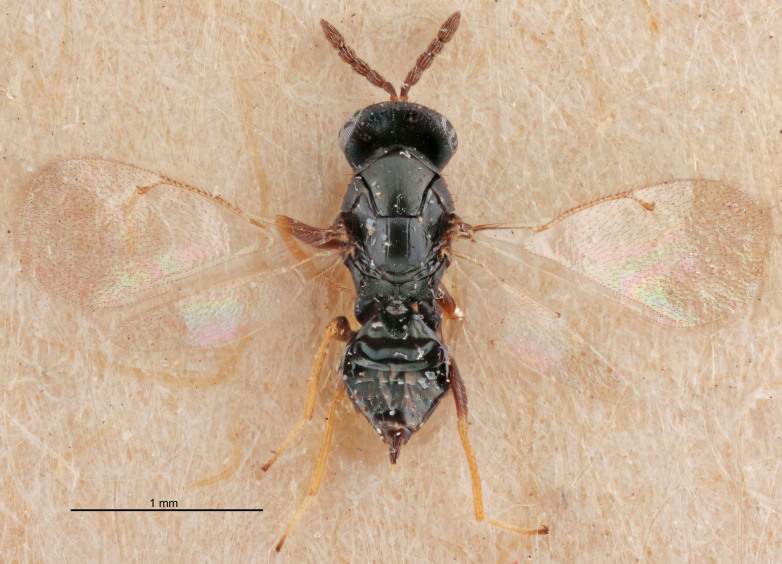
Holotype female, dorsal

**Figure 6b.**
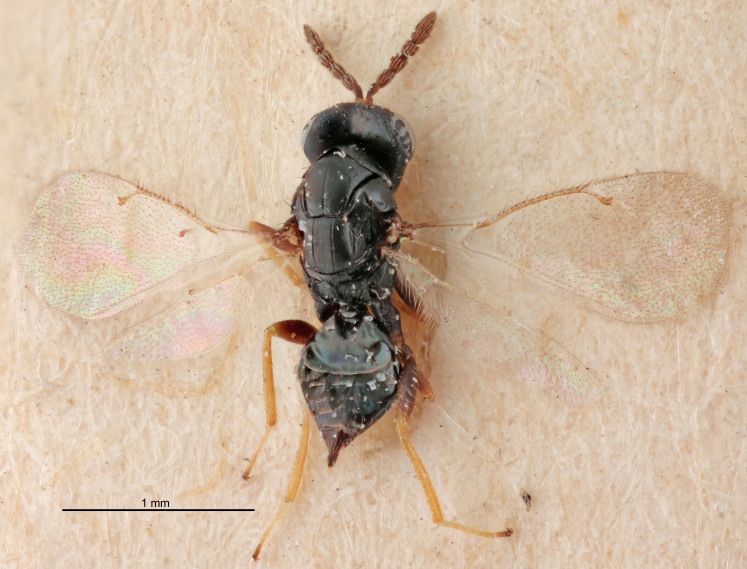
Holotype female, dorso-lateral.

**Figure 6c.**
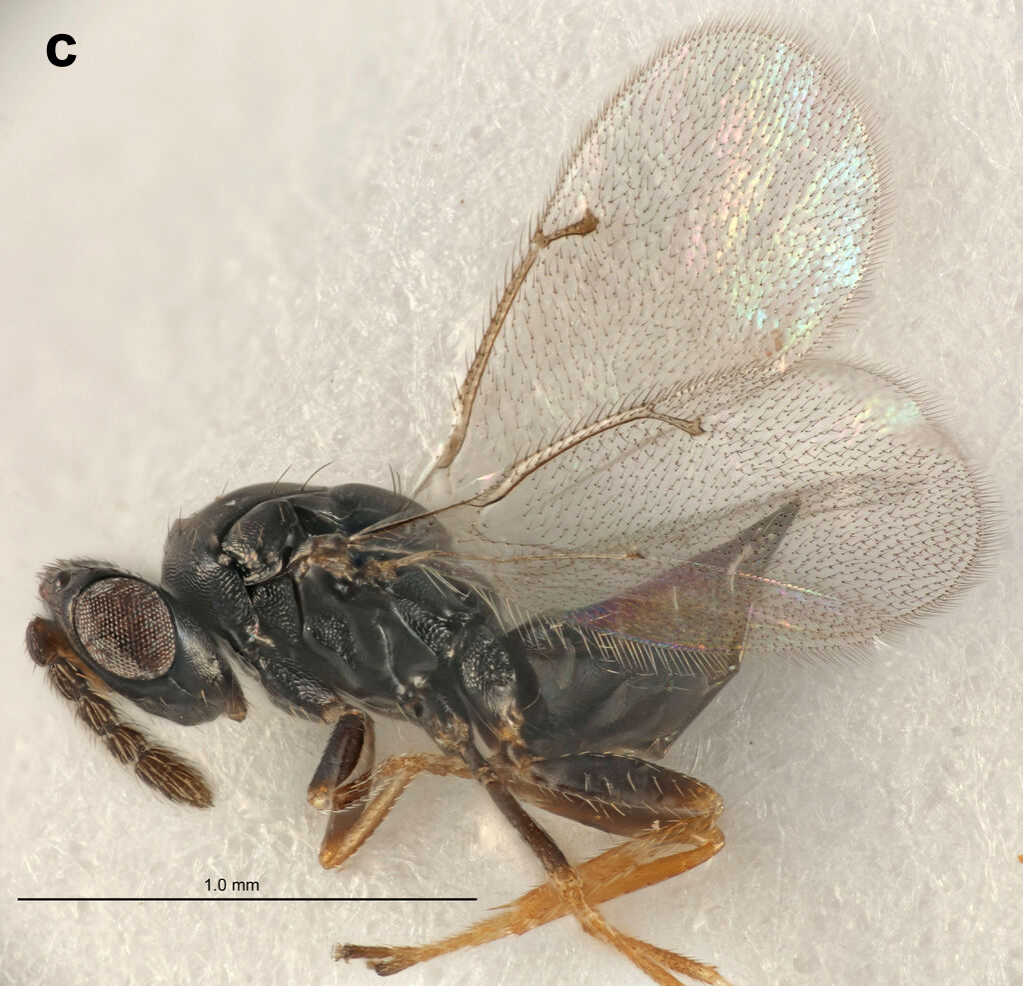
Nontype female, lateral.

**Figure 6d.**
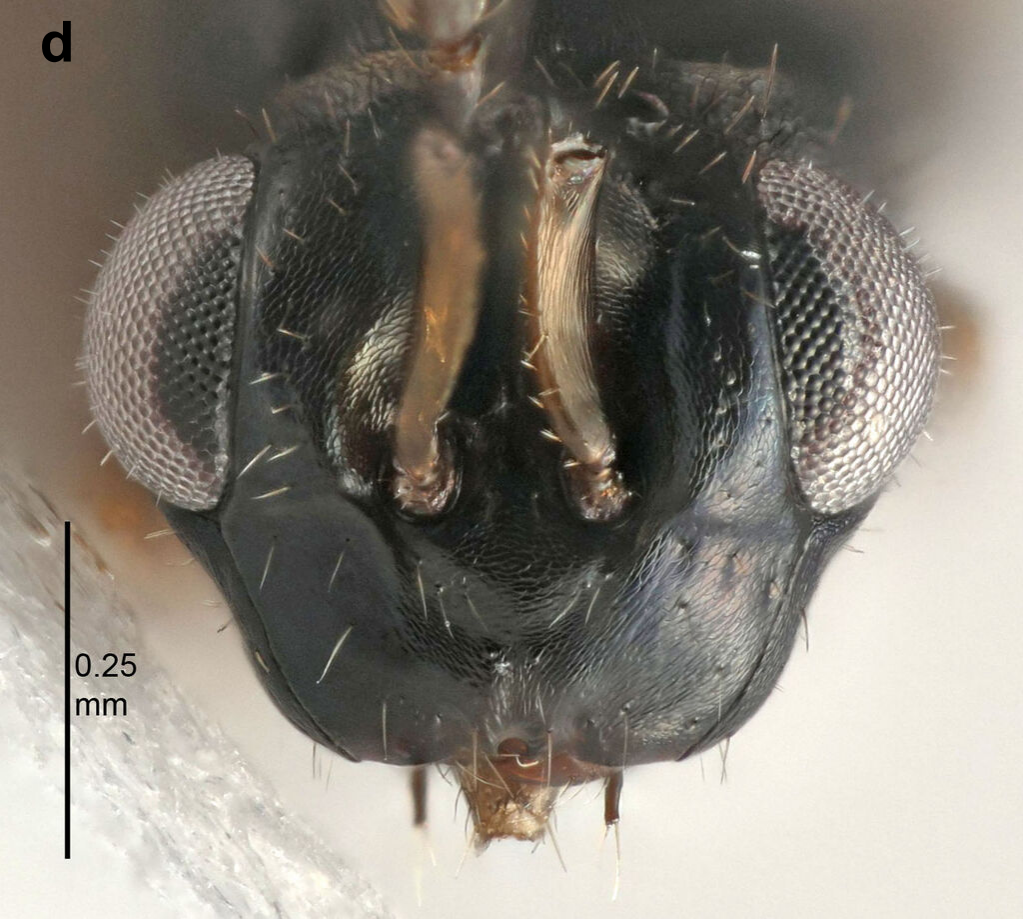
Nontype female, head frontal.

**Figure 7a.**
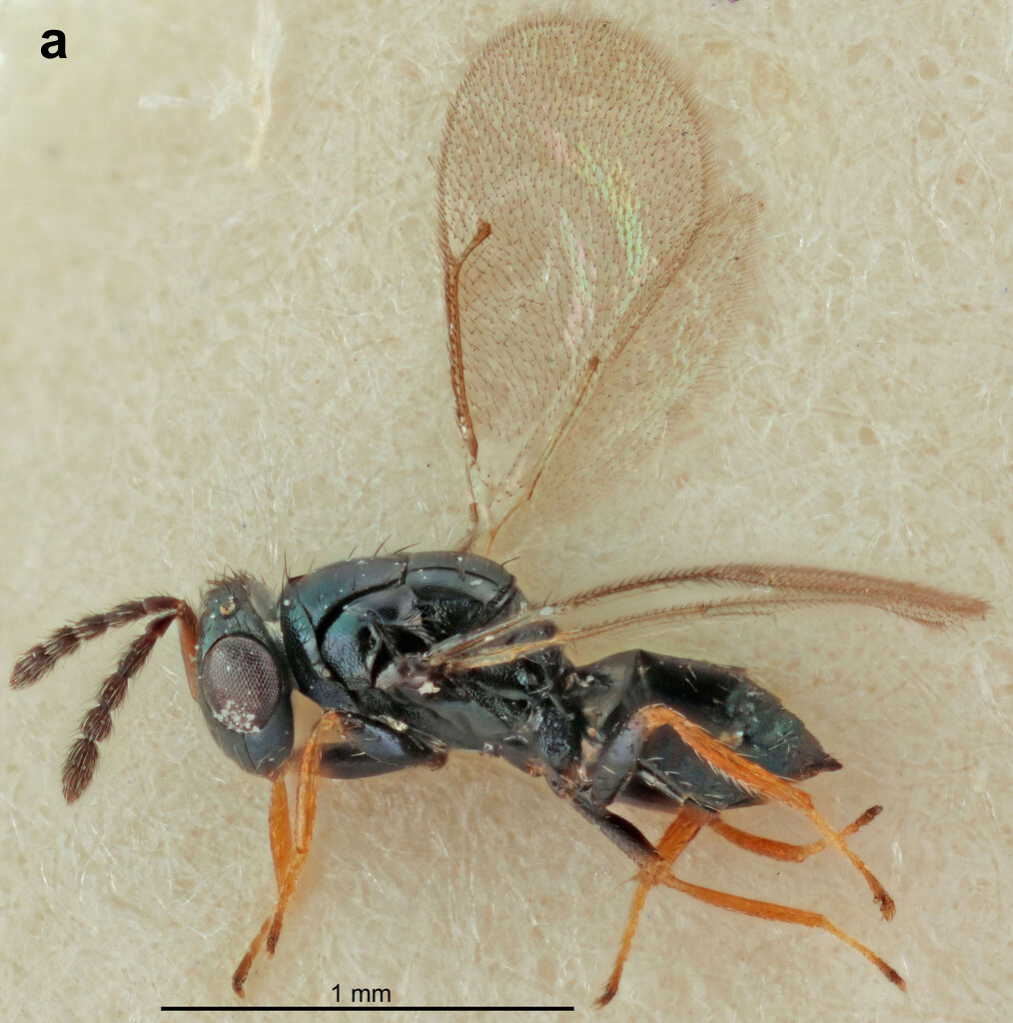
Holotype female, lateral.

**Figure 7b.**
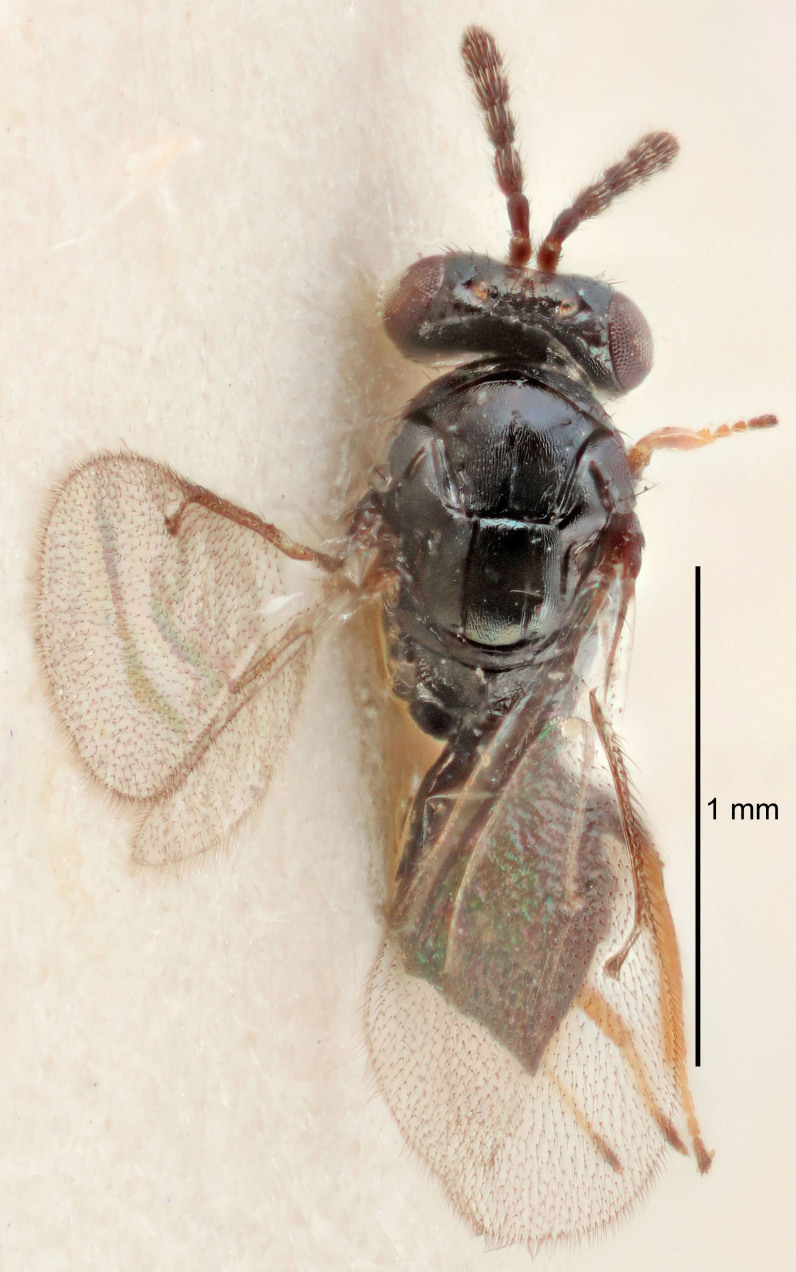
Holotype female, dorsal.

**Figure 7c.**
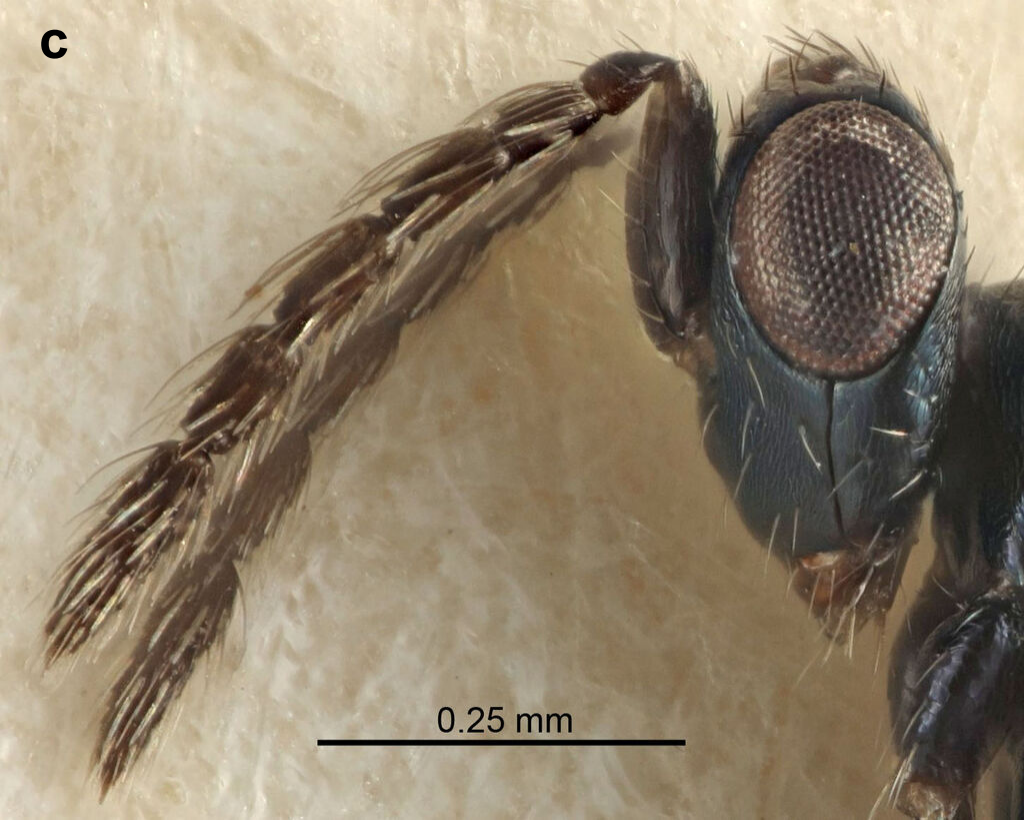
Nontype male, head & antenna lateral.

**Figure 7d.**
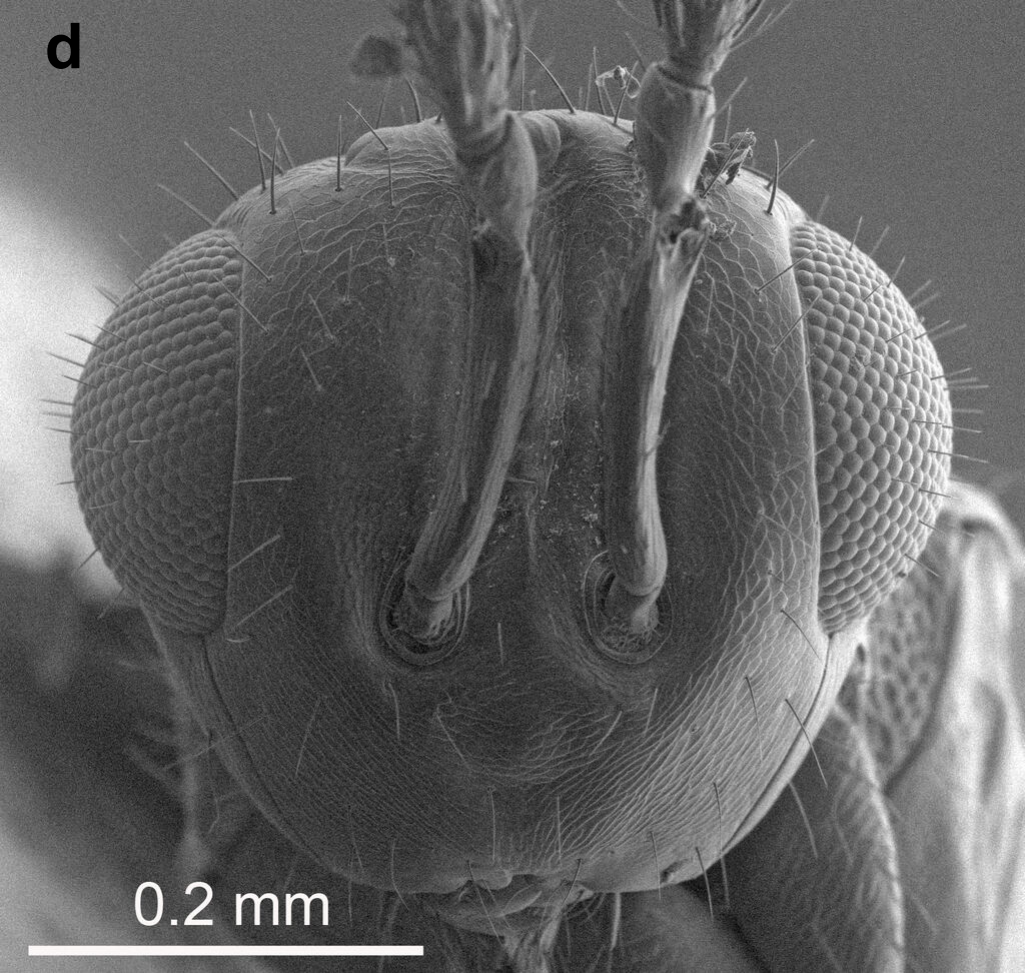
Nontype female, head frontal.

**Figure 8a.**
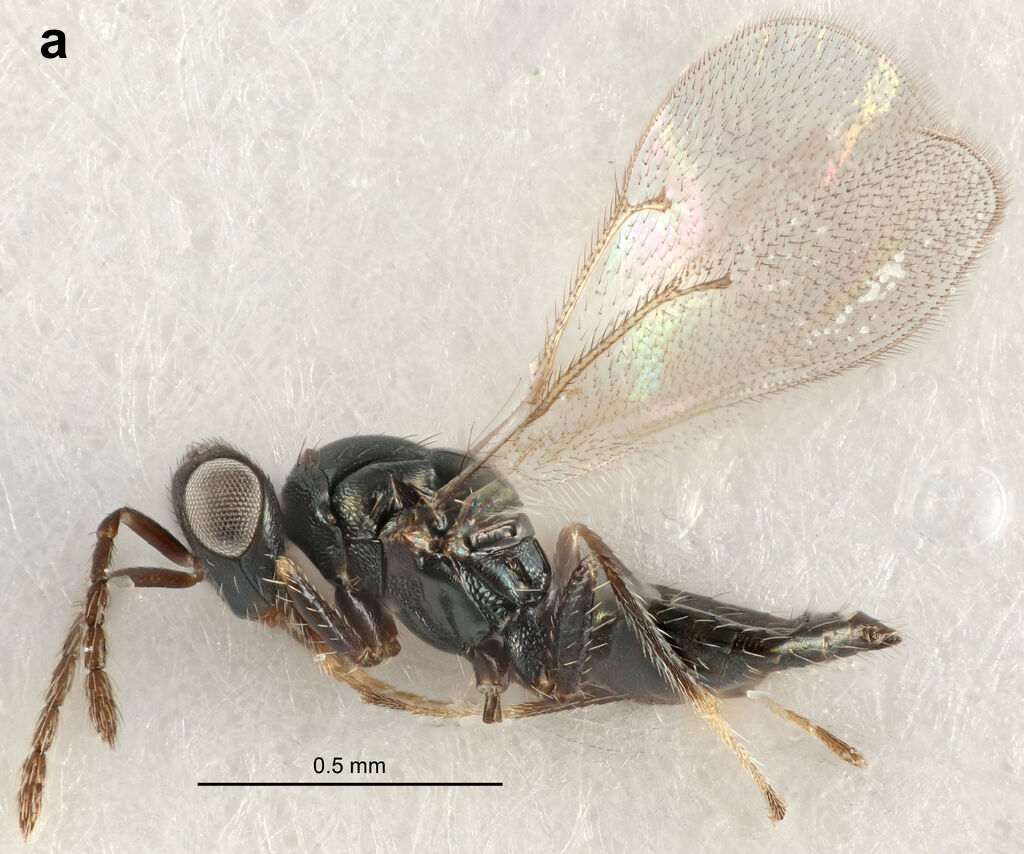
Holotype female, lateral.

**Figure 8b.**
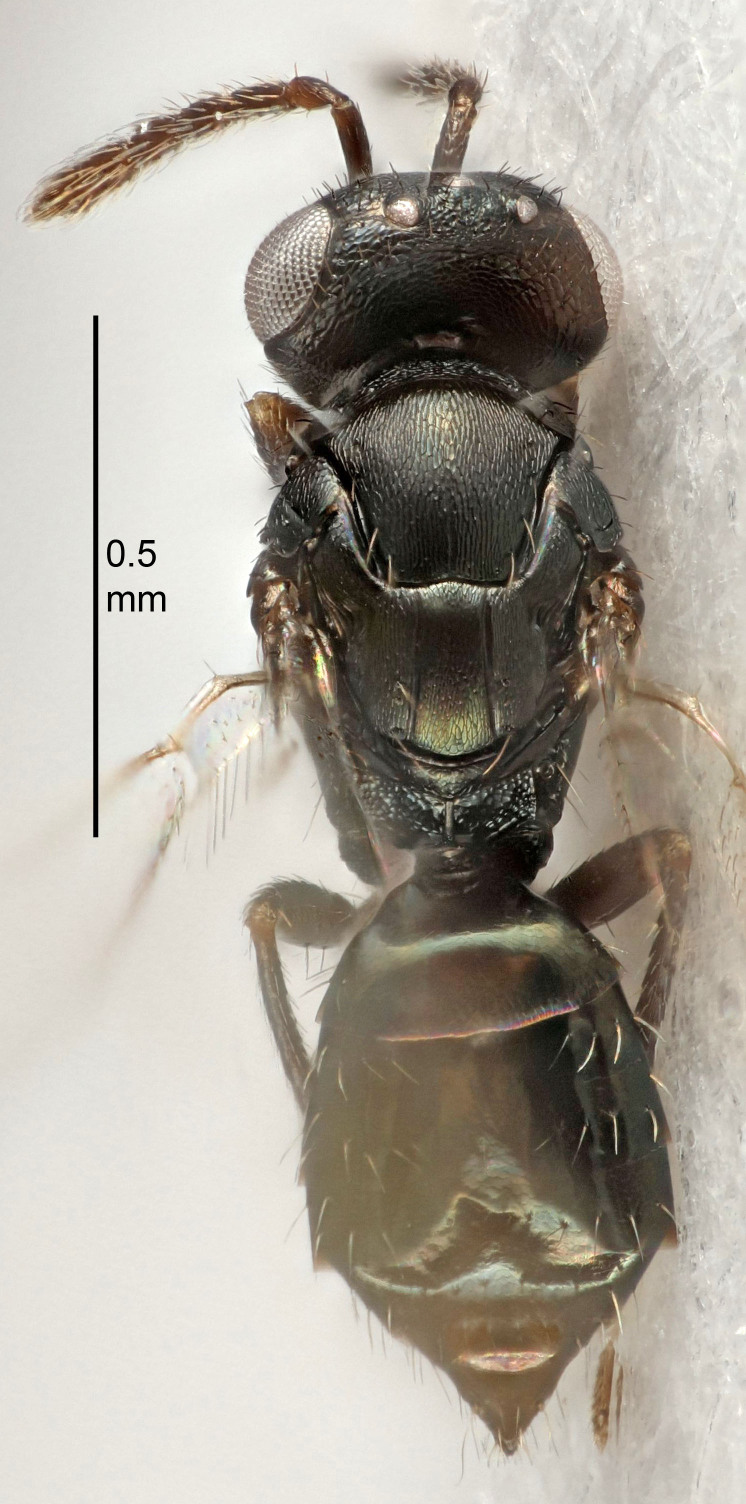
Holotype female, dorsal.

**Figure 8c.**
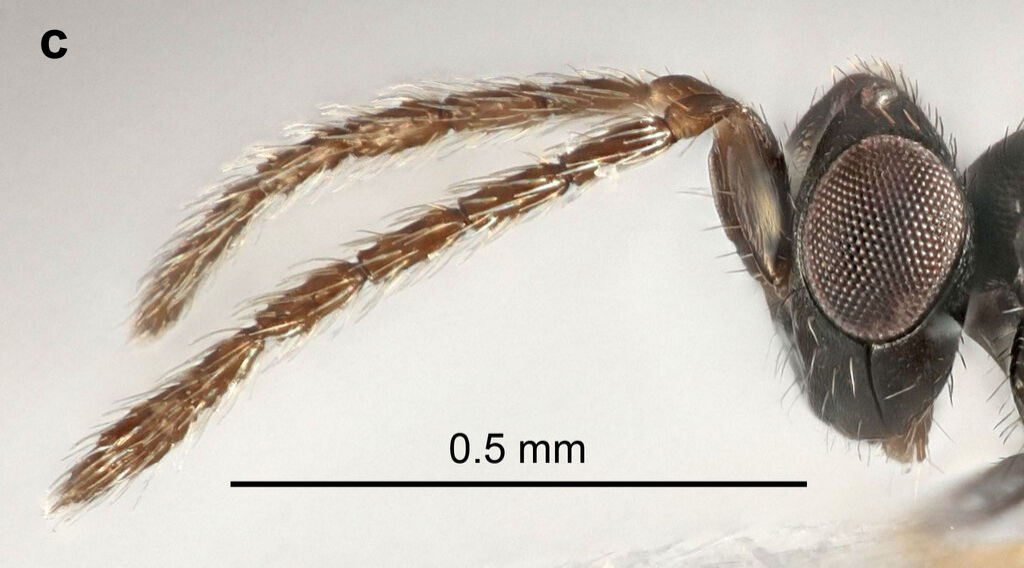
Paratype male, head and antenna lateral.

**Figure 9a.**
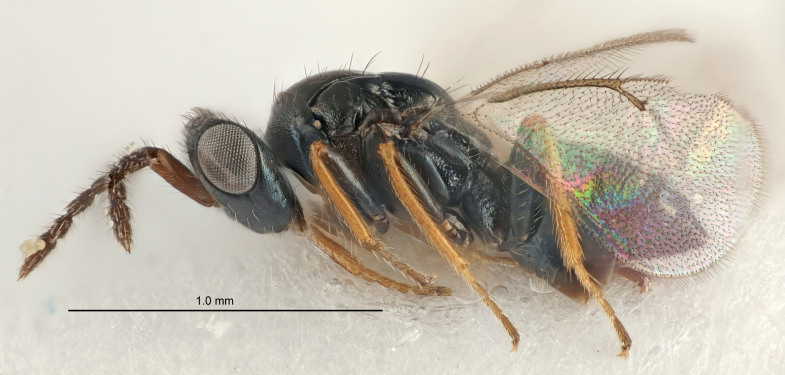
Holotype female, lateral.

**Figure 9b.**
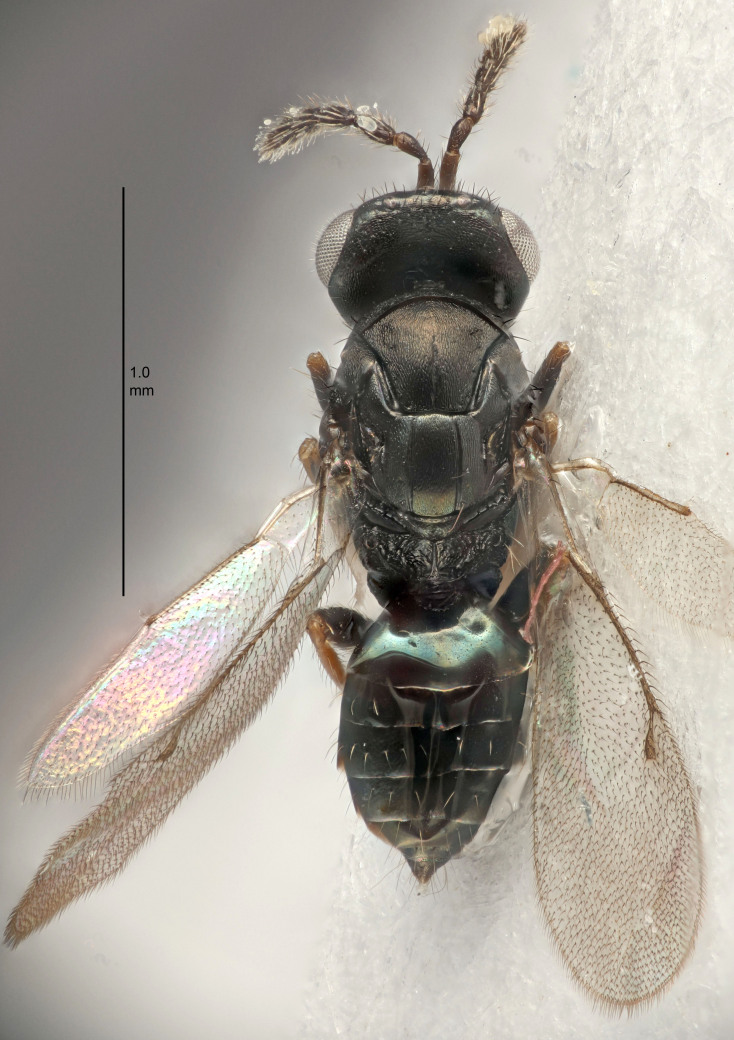
Holotype female, dorsal.

**Figure 9c.**
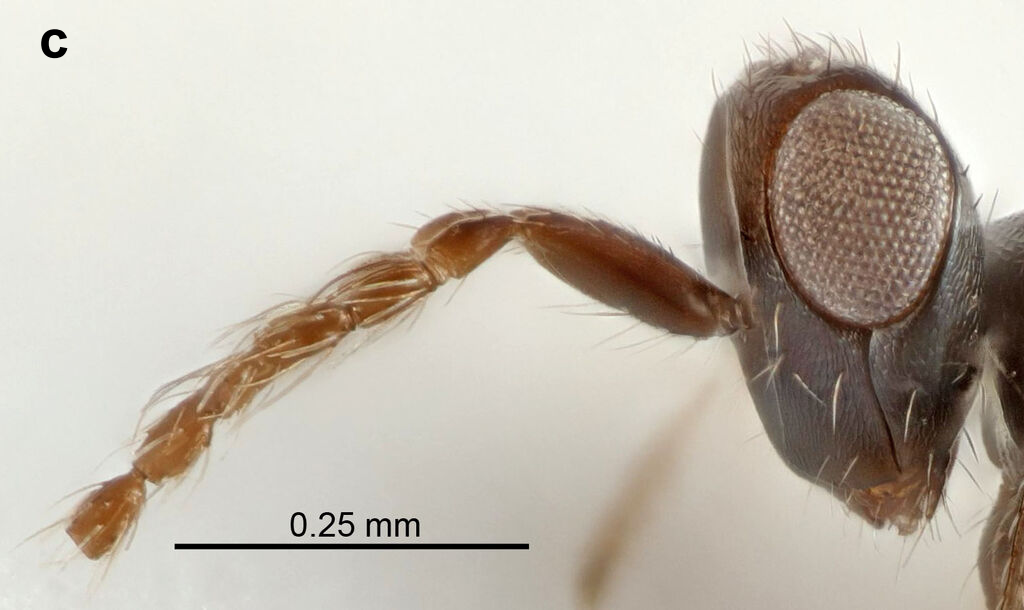
Paratype male, head and antenna lateral.

**Figure 10a.**
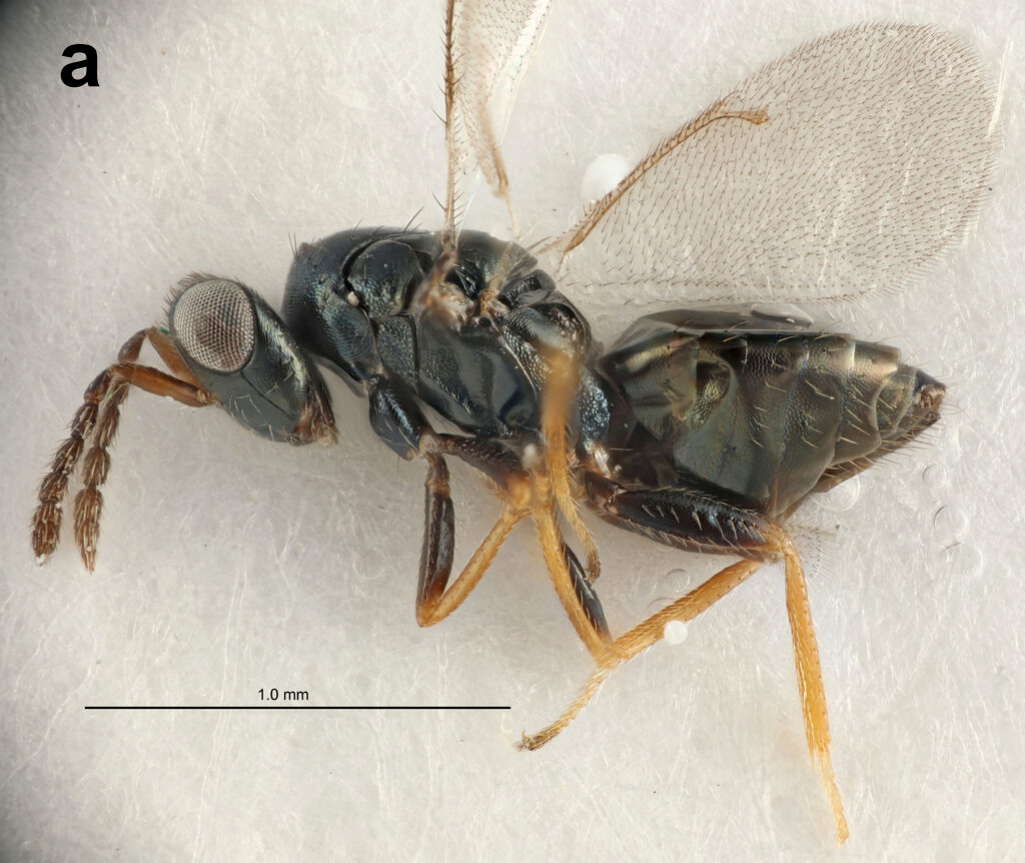
Holotype female, lateral.

**Figure 10b.**
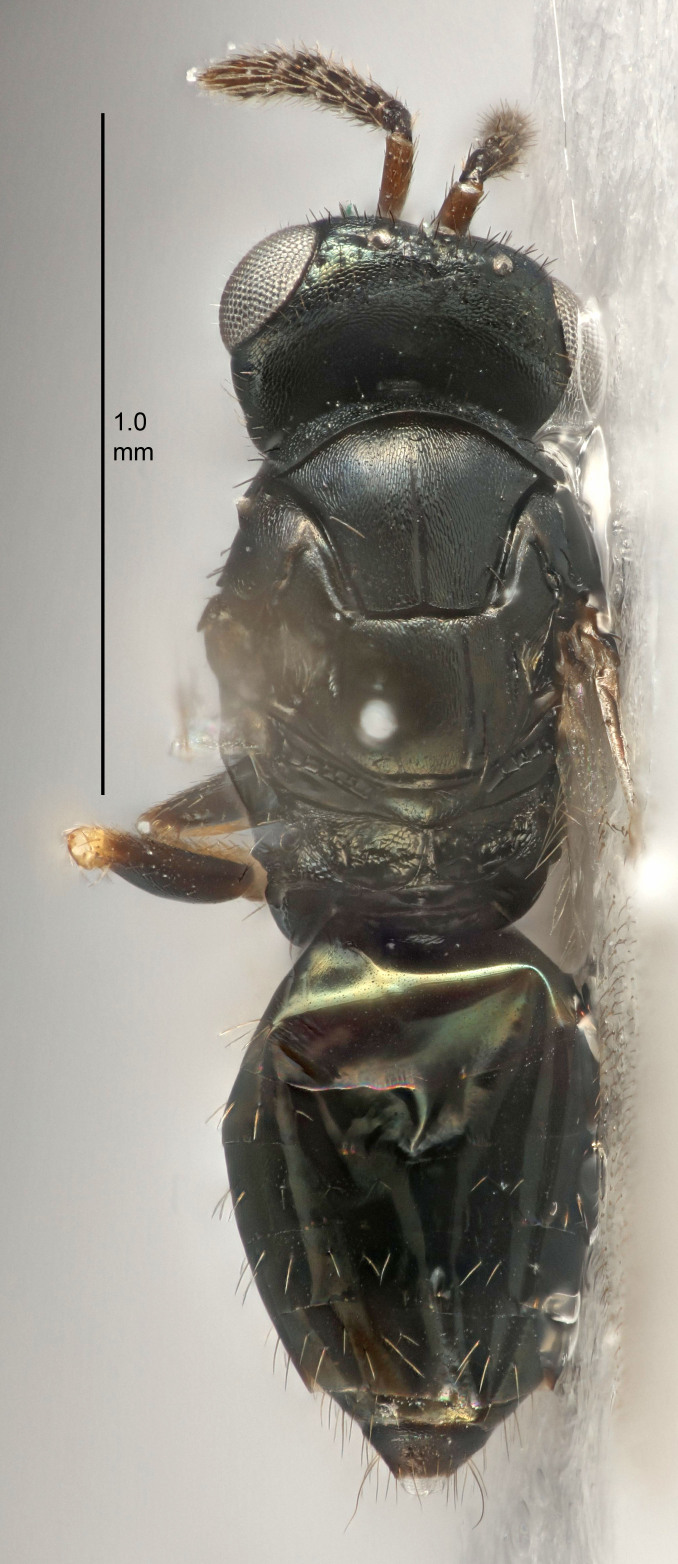
Holotype female, dorsal.

**Figure 10c.**
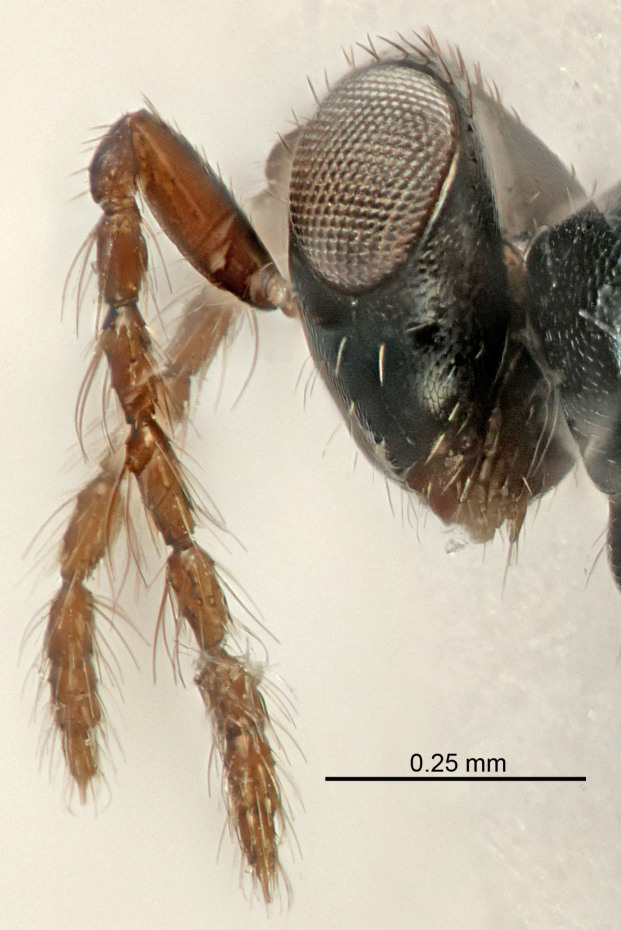
Paratype male, head and antenna lateral.

**Figure 11a.**
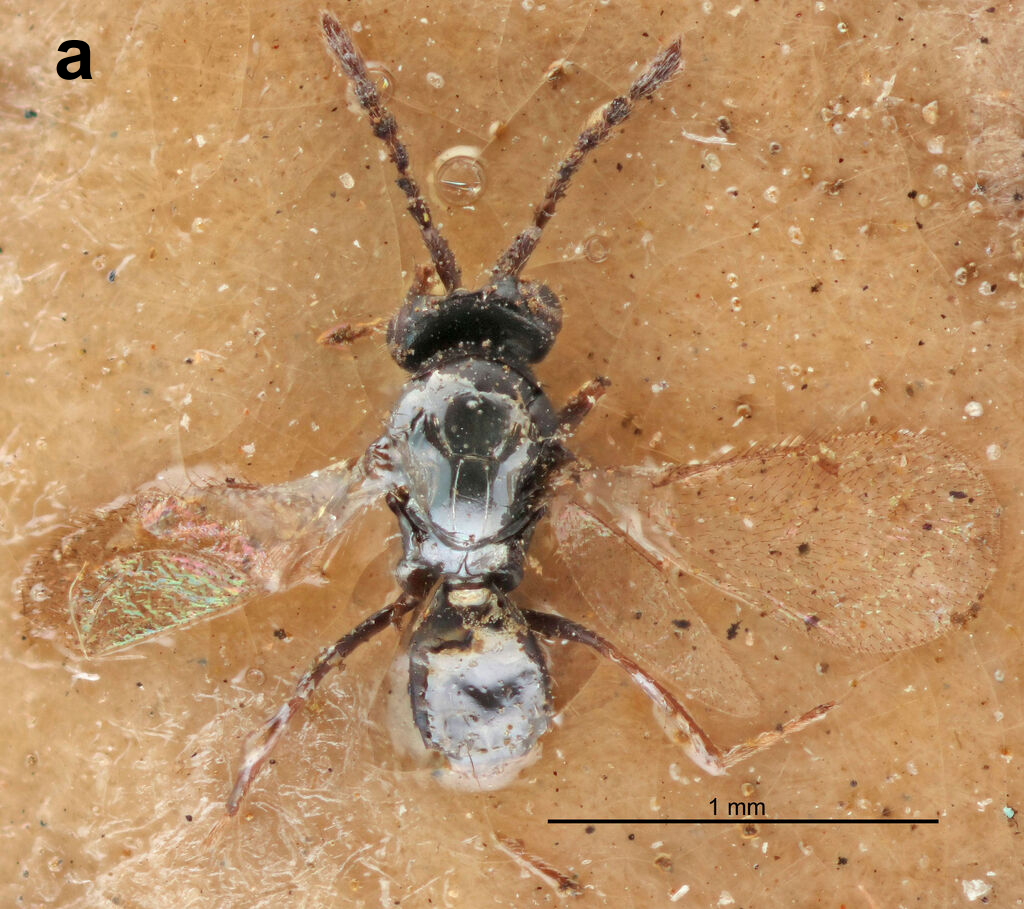
Lectotype male, dorsal.

**Figure 11b.**
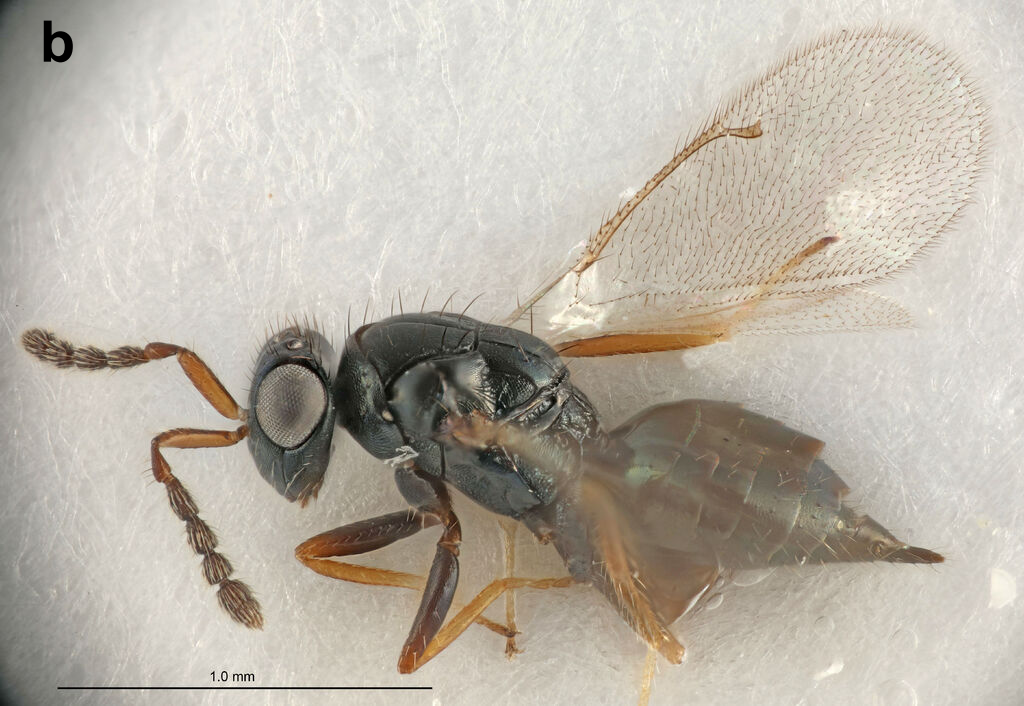
Nontype female, lateral.

**Figure 11c.**
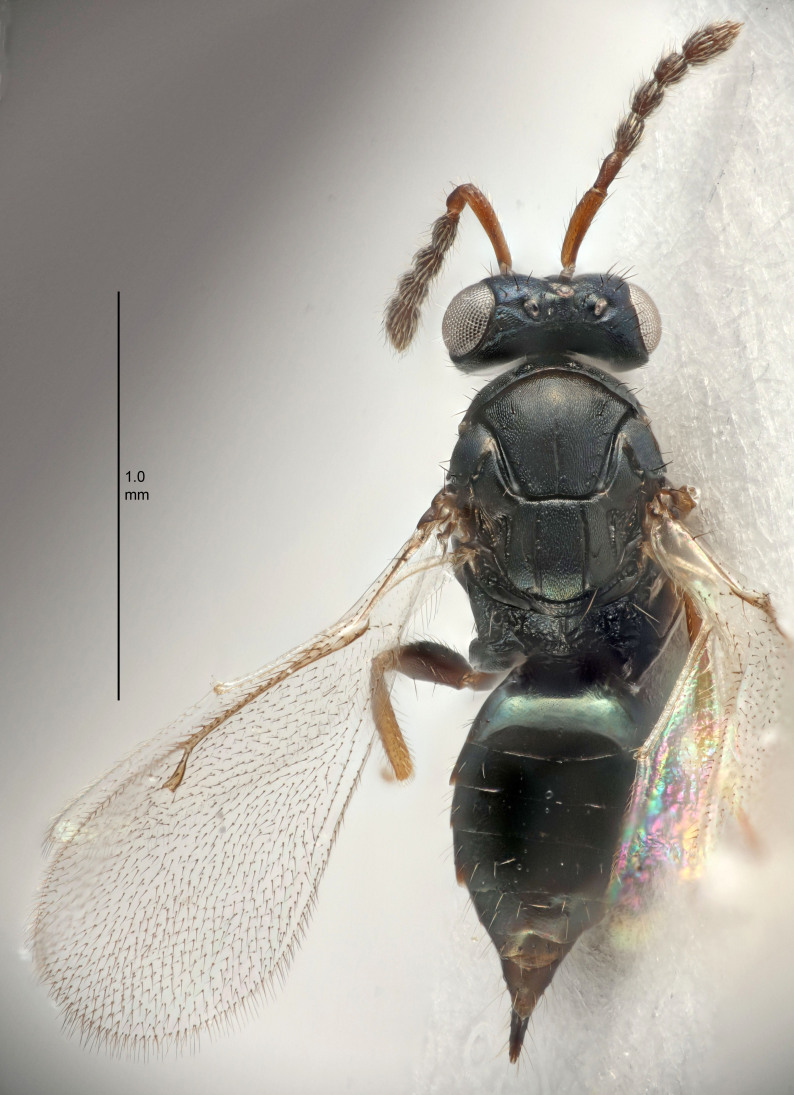
Nontype female, dorsal.

**Figure 13a.**
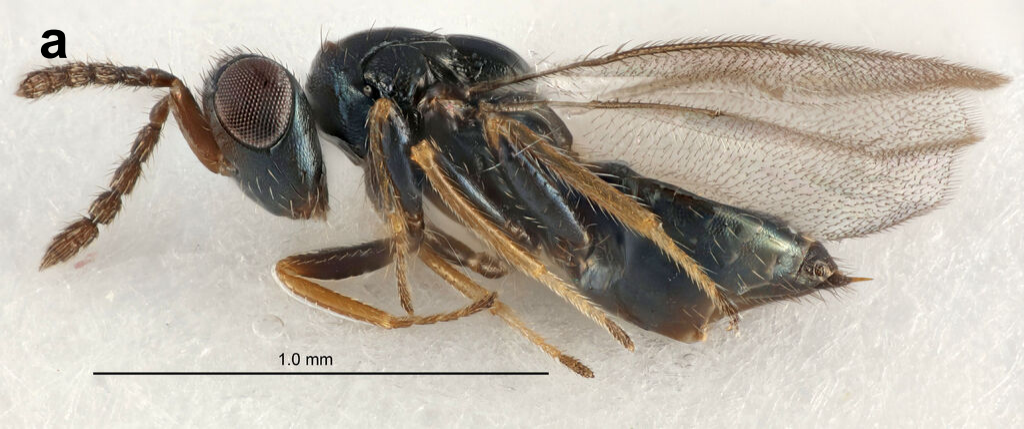
Holotype female, lateral.

**Figure 13b.**
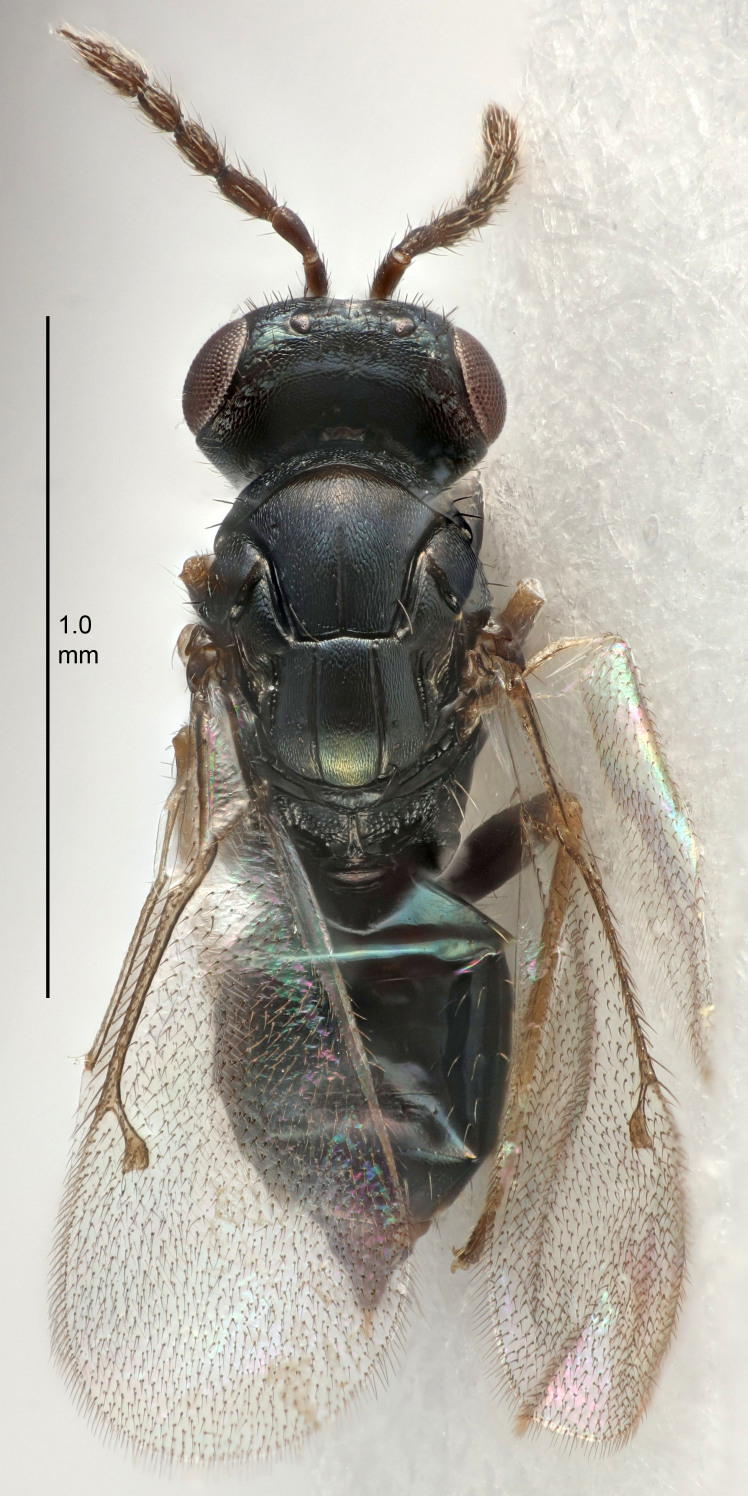
Holotype female, dorsal.

**Figure 13c.**
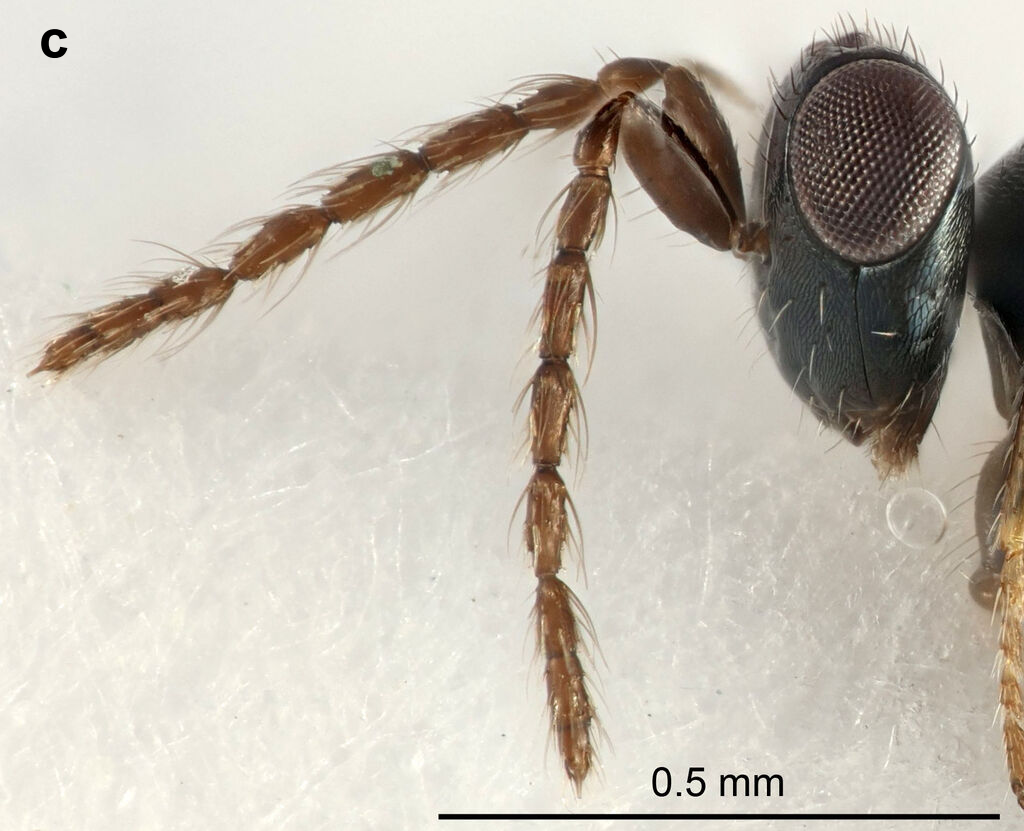
Paratype male, head and antenna lateral.

**Figure 14a.**
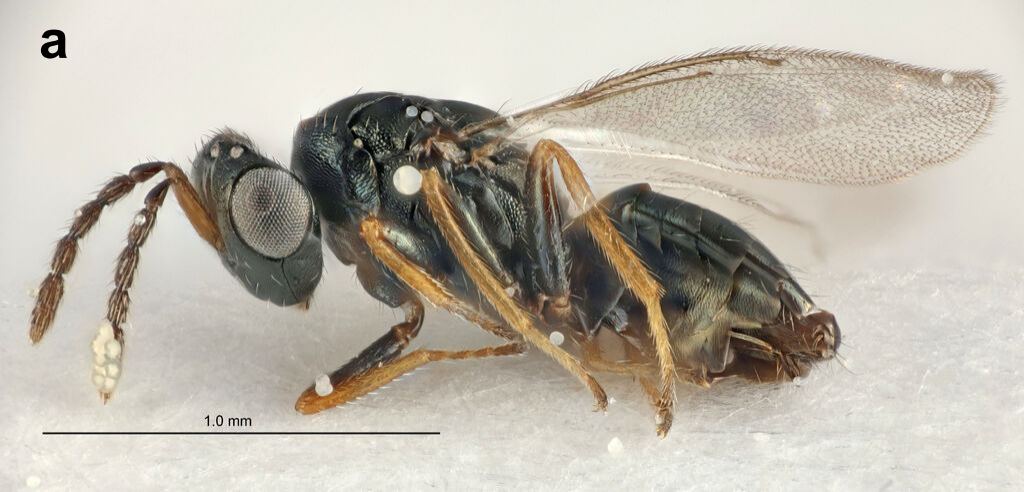
Holotype female, lateral.

**Figure 14b.**
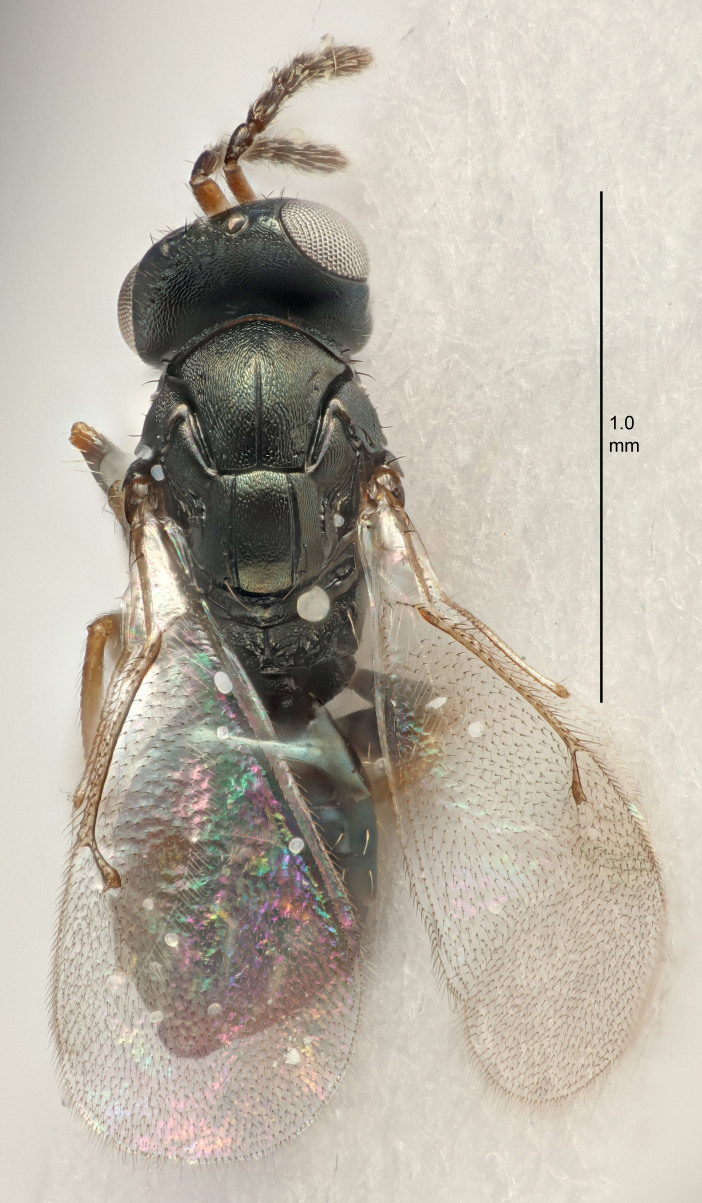
Holotype female, dorsal.

**Figure 15a.**
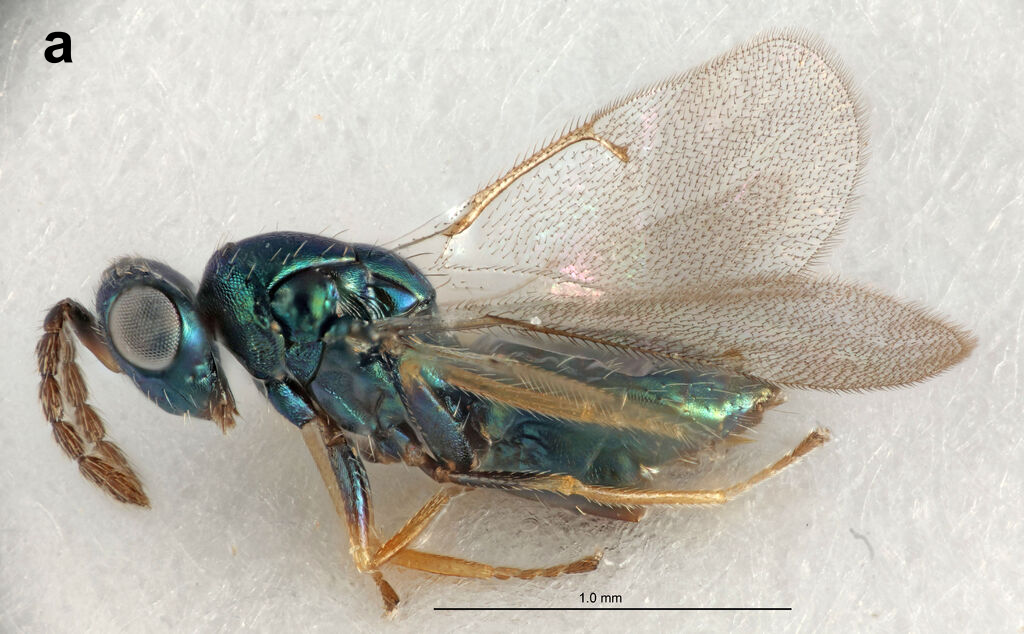
Holotype female, lateral.

**Figure 15b.**
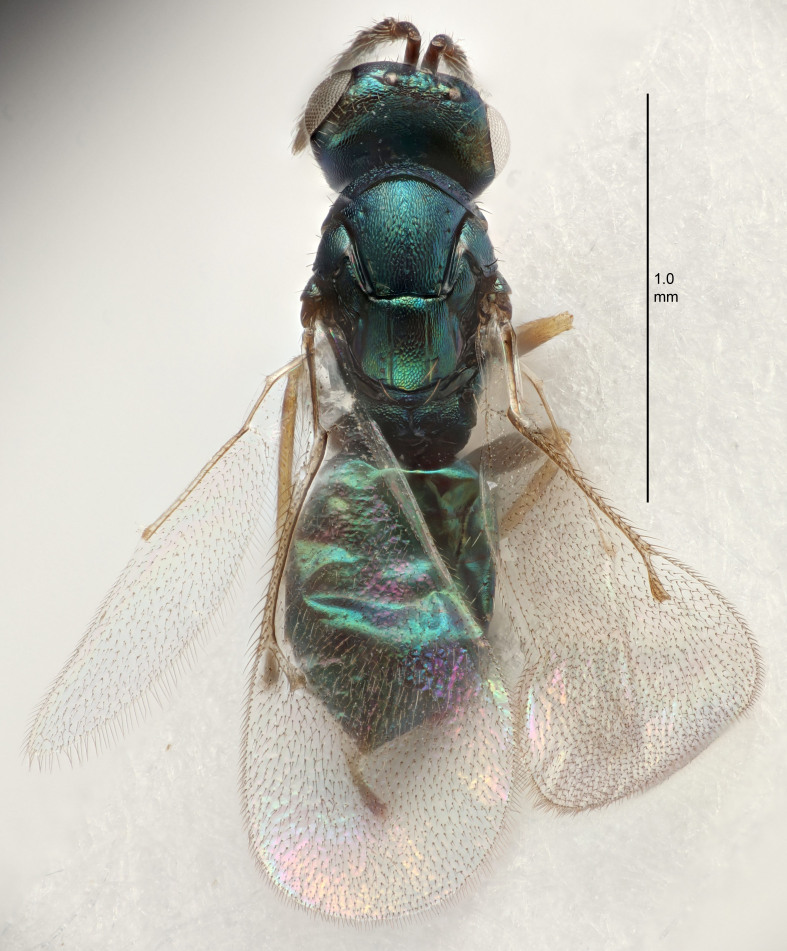
Holotype female, dorsal.

**Figure 15c.**
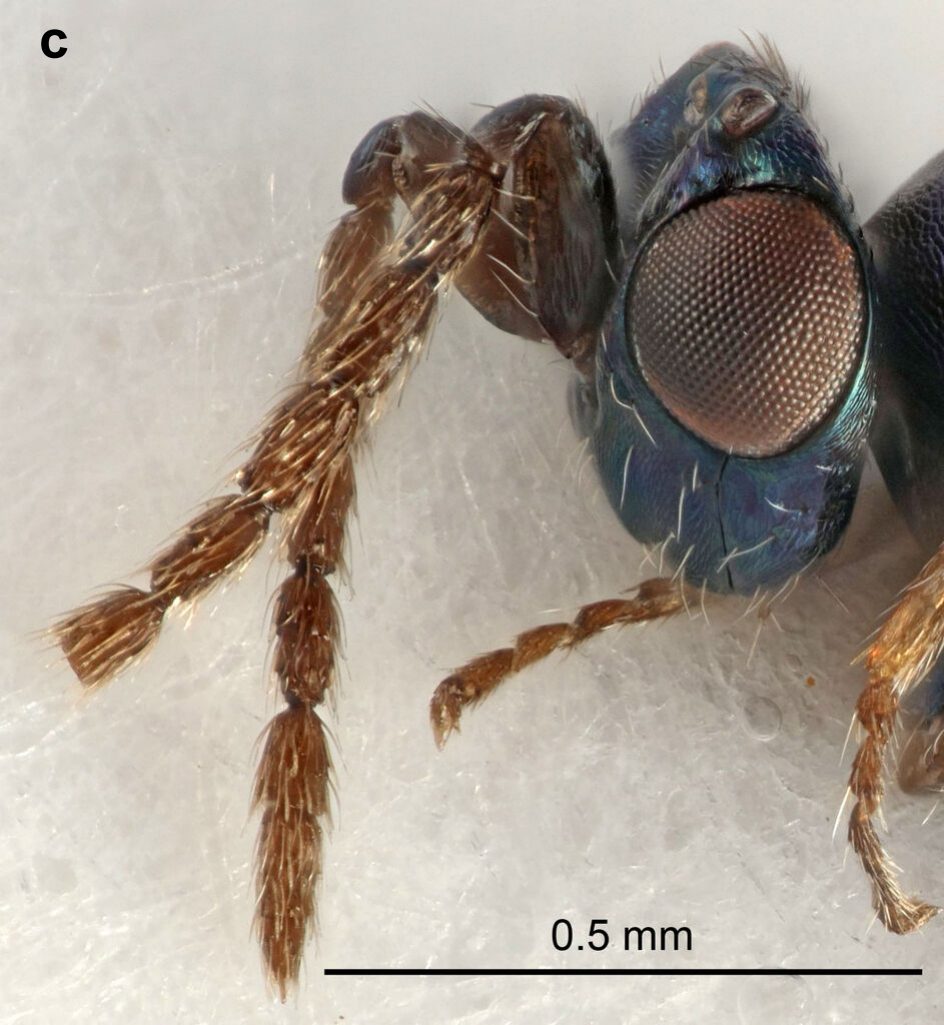
Paratype male, head+antenna, lateral.

**Figure 16a.**
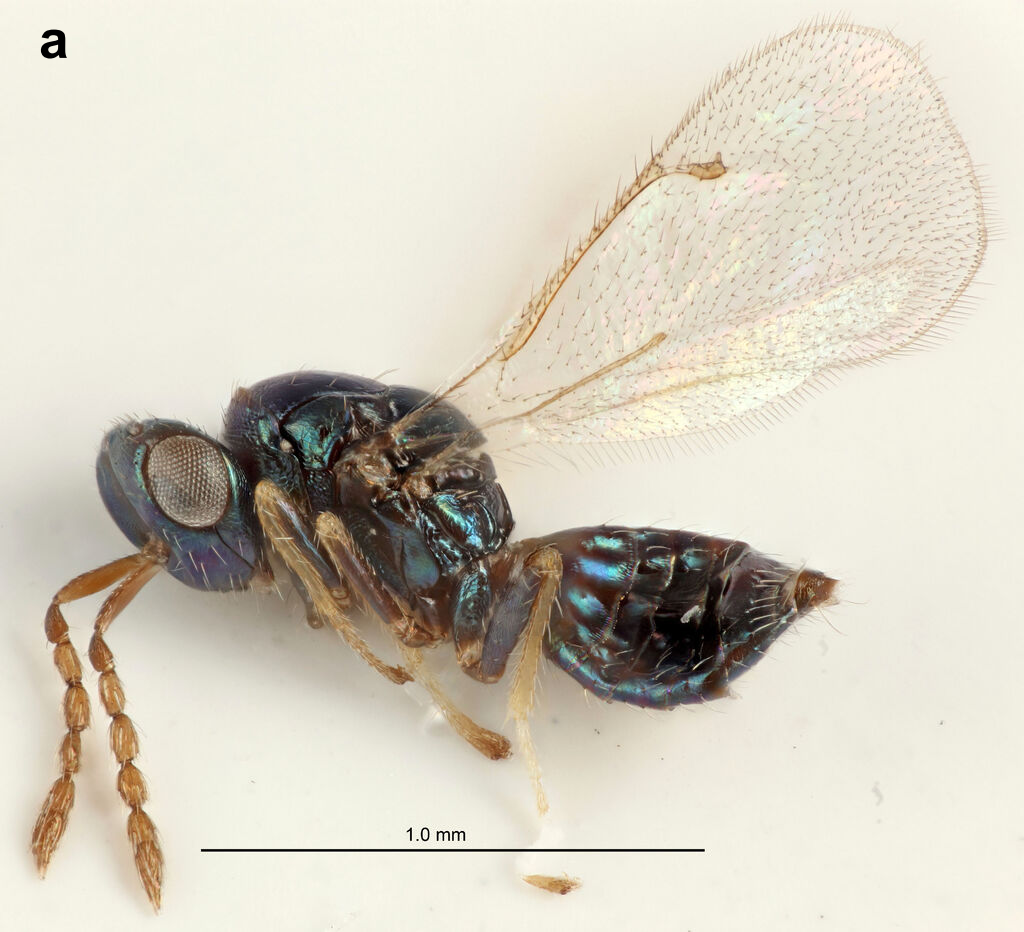
Holotype female, lateral.

**Figure 16b.**
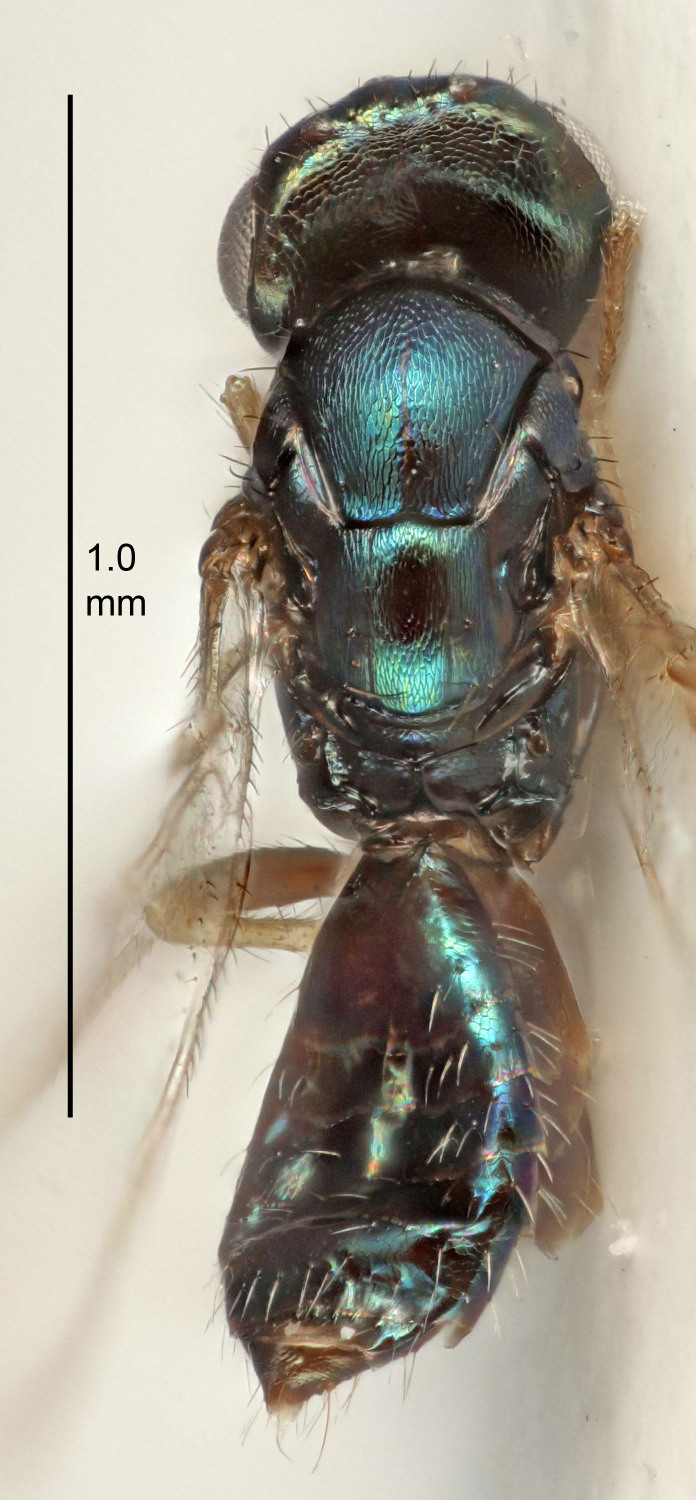
Holotype female, dorsal.

**Figure 17a.**
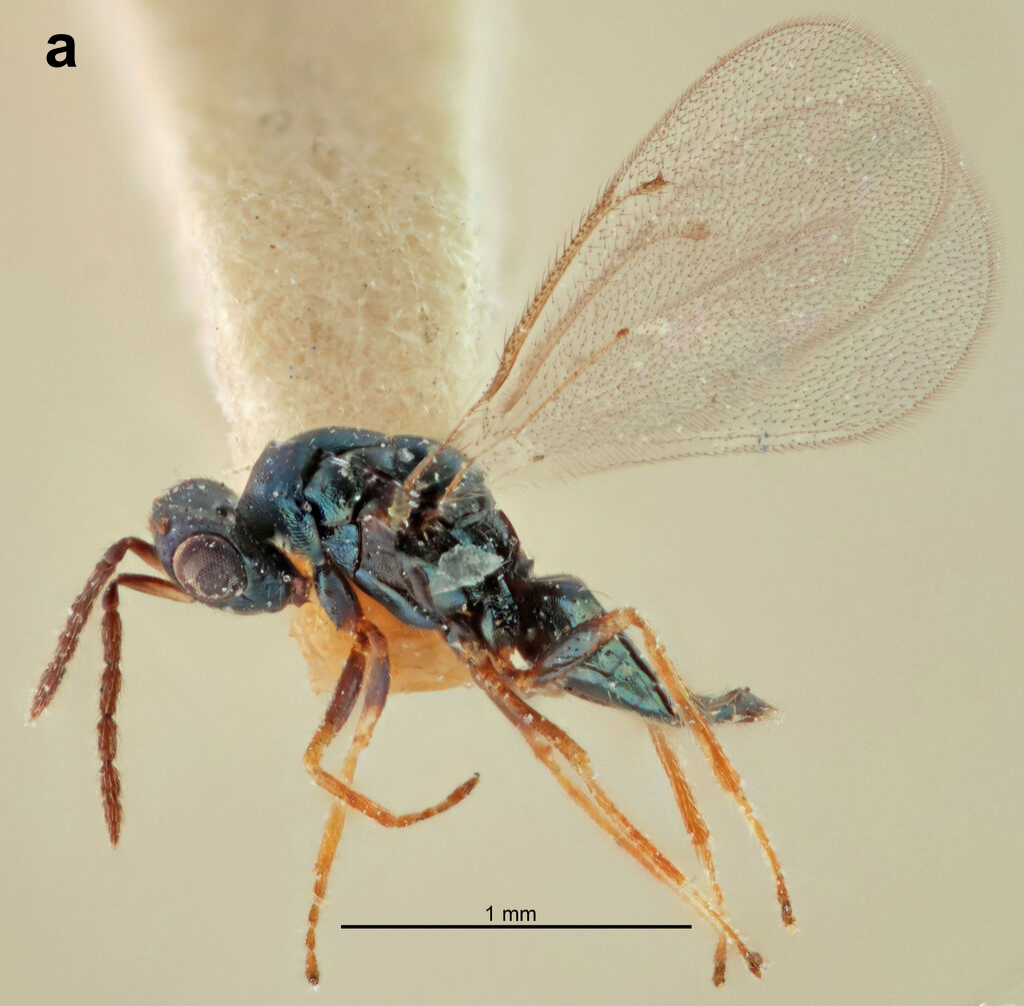
Holotype female, lateral.

**Figure 17b.**
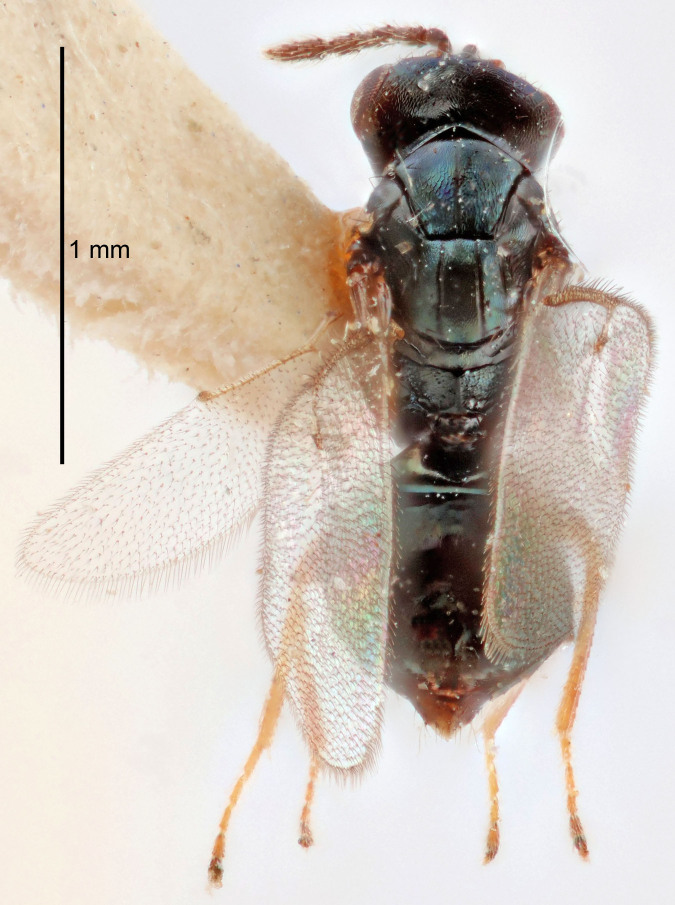
Holotype female, dorsal.

**Figure 18a.**
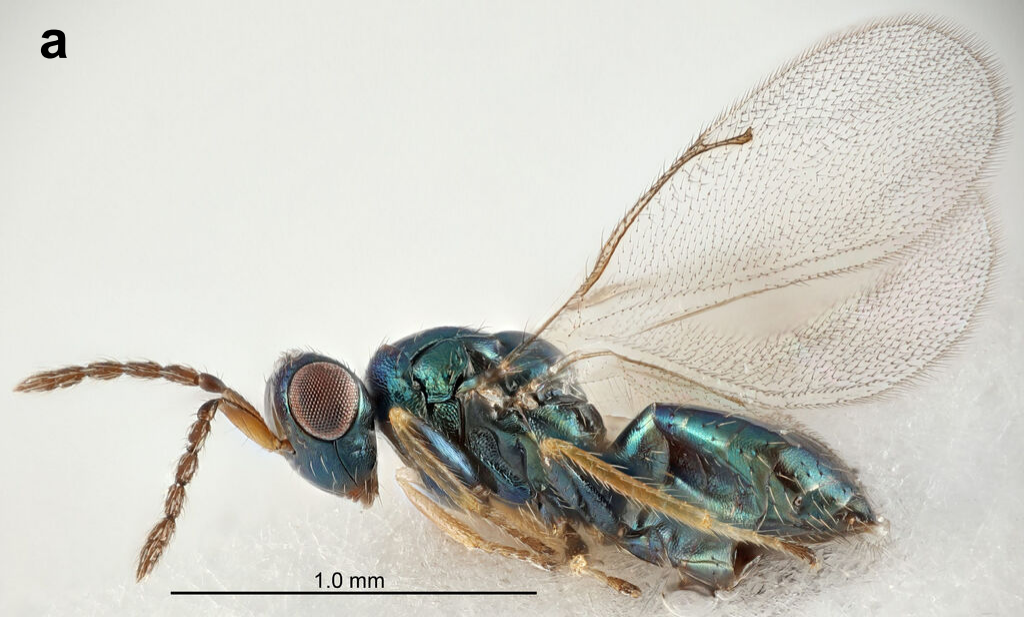
Holotype female, lateral.

**Figure 18b.**
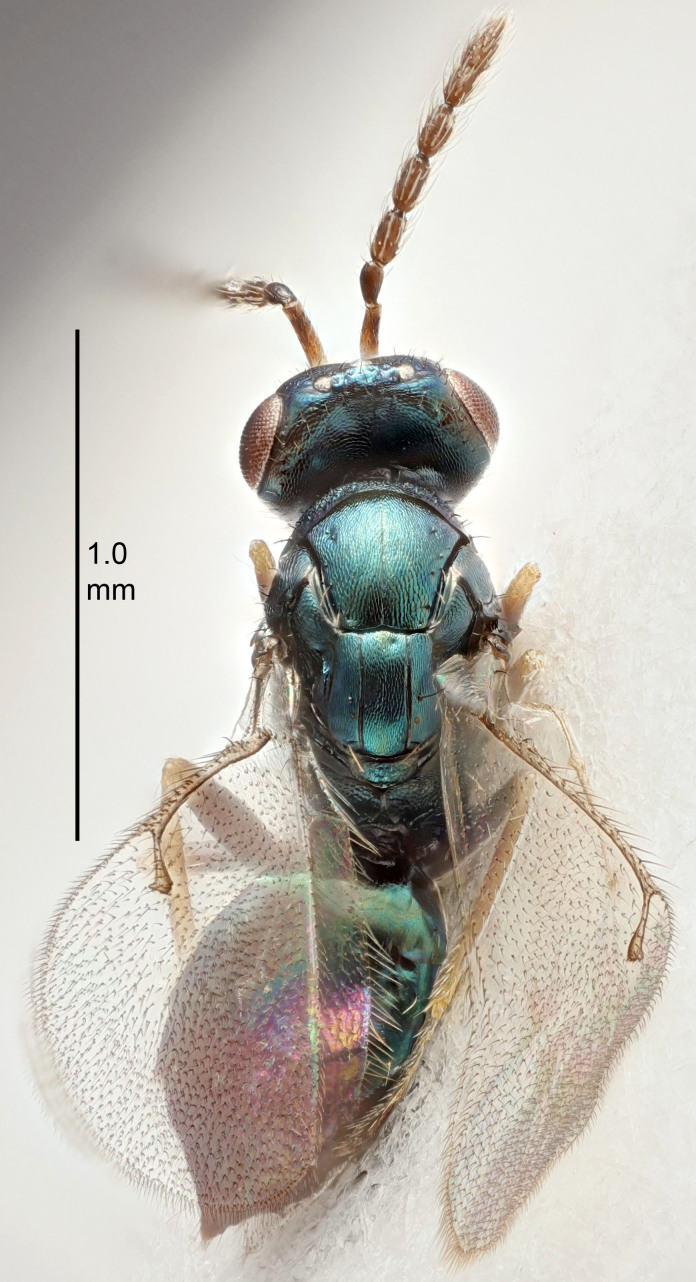
Holotype female, dorsal.

**Figure 19a.**
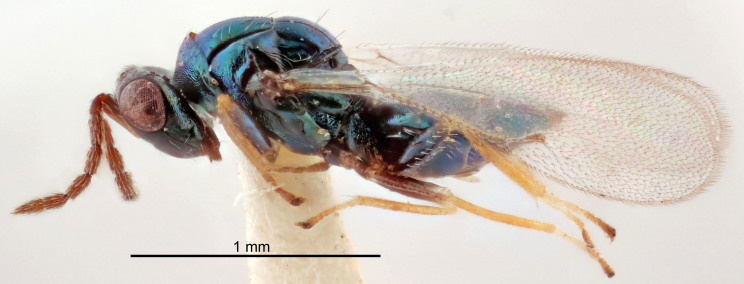
Holotype female, lateral.

**Figure 19b.**
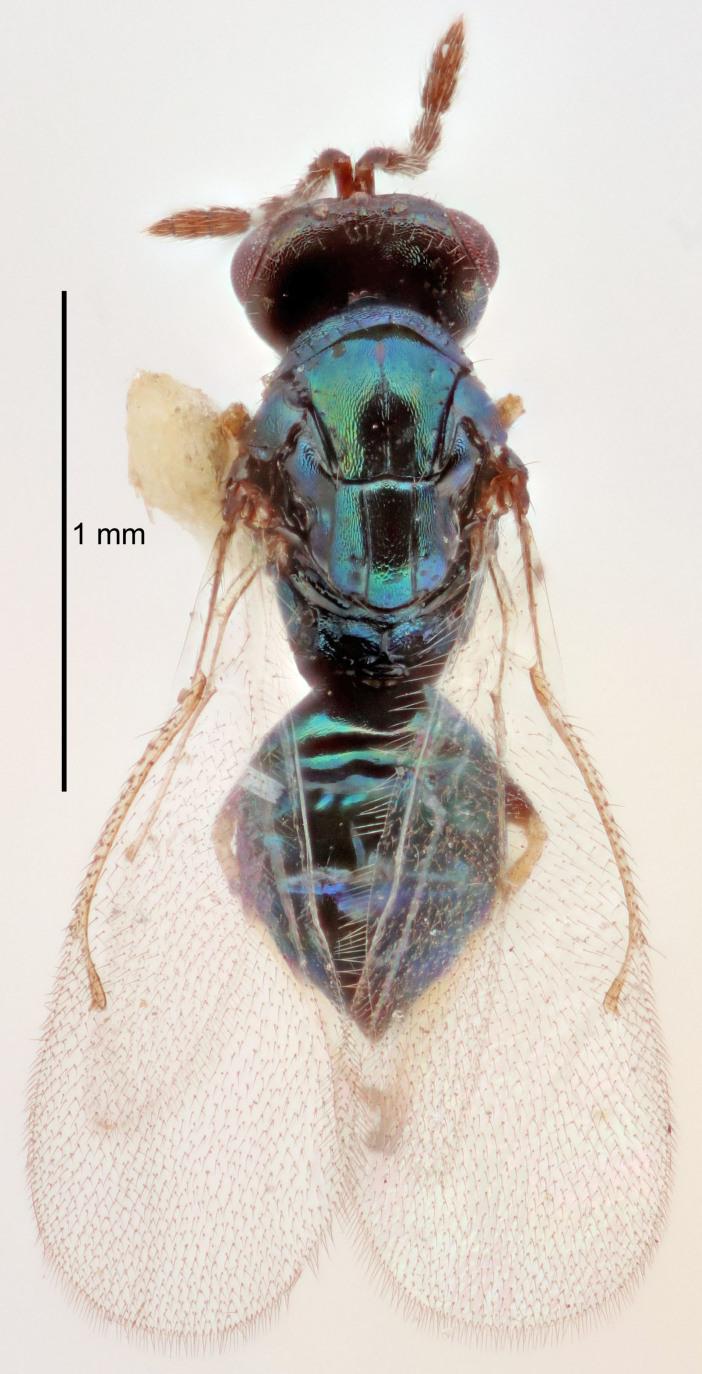
Holotype female, dorsal.

**Figure 20a.**
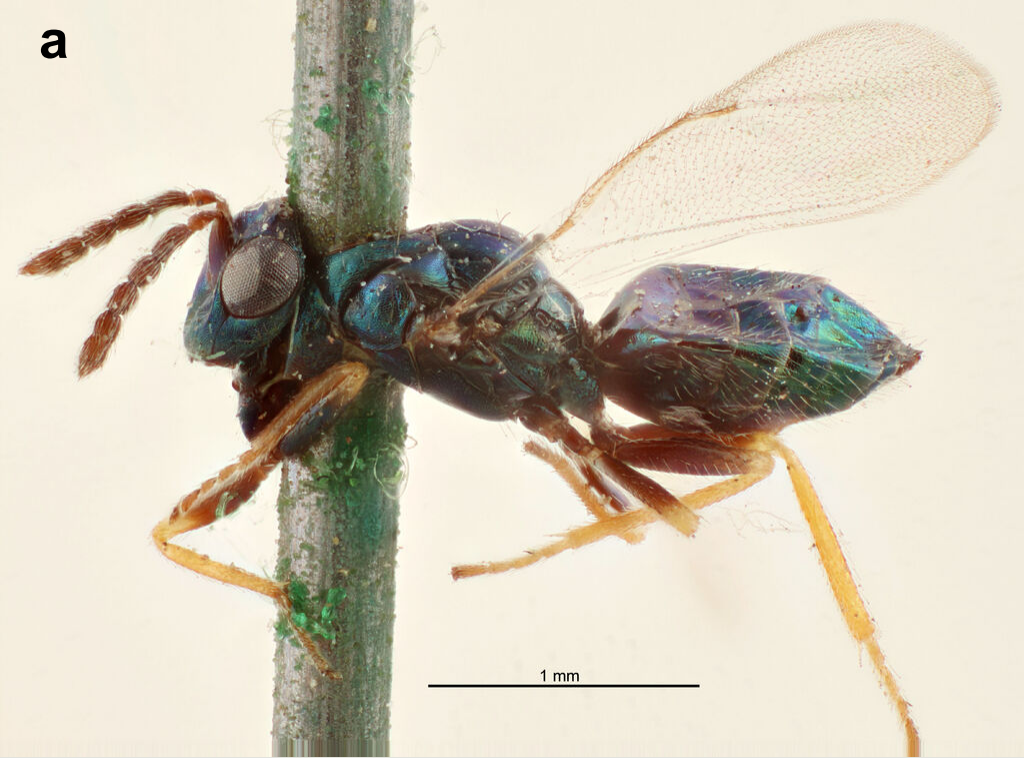
Lectotype female, lateral.

**Figure 20b.**
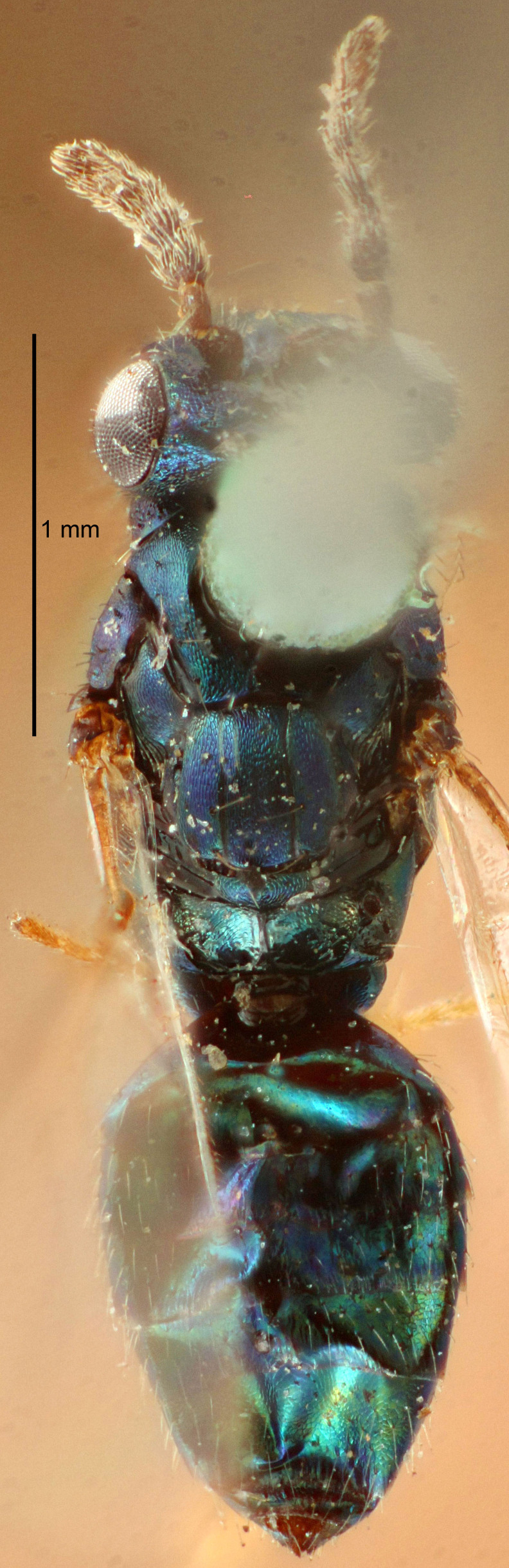
Lectotype female, dorsal.

**Figure 20c.**
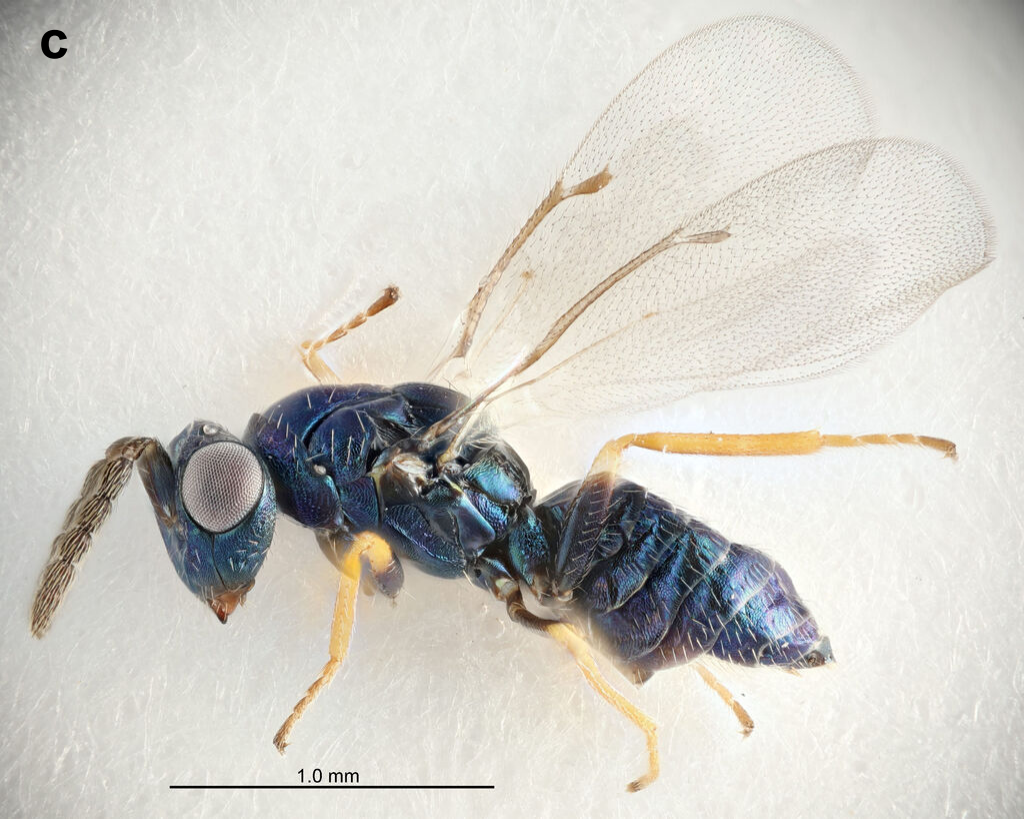
Nontype female, lateral.

**Figure 20d.**
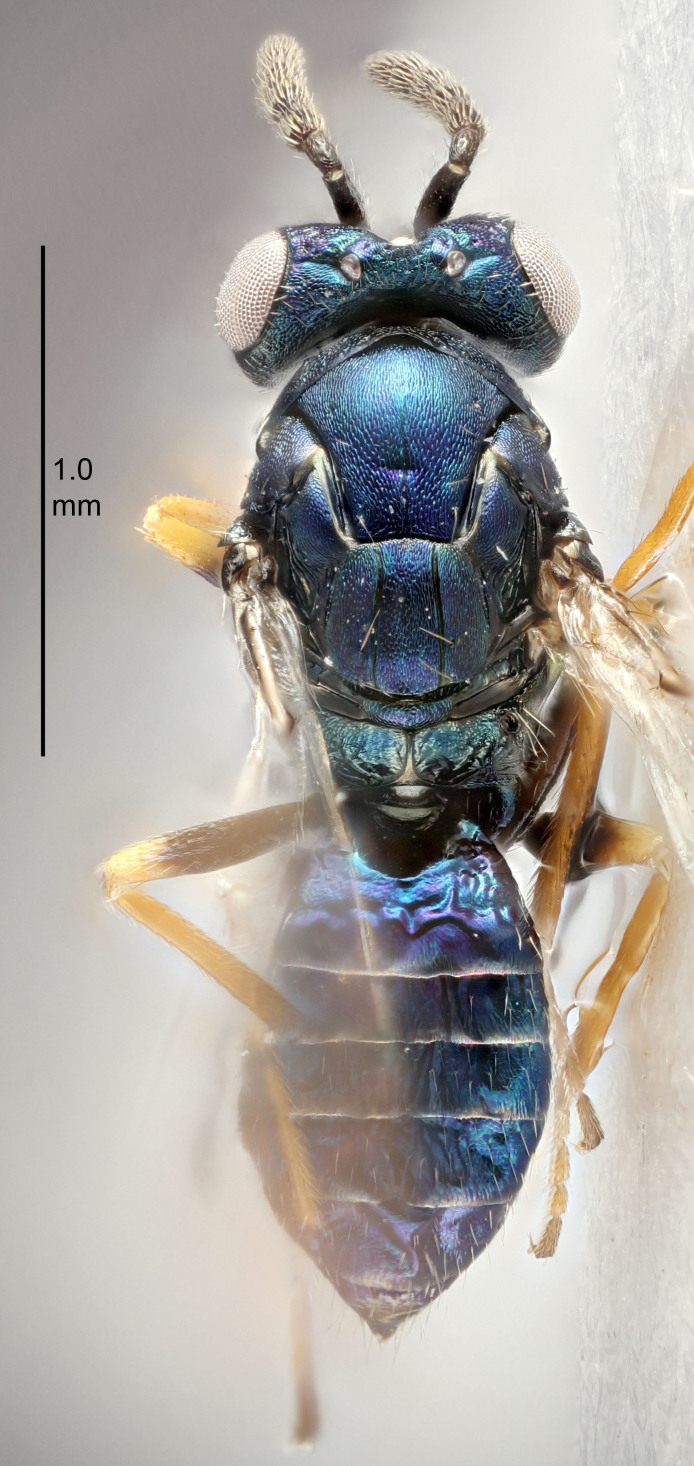
Nontype female, dorsal.

**Figure 20e.**
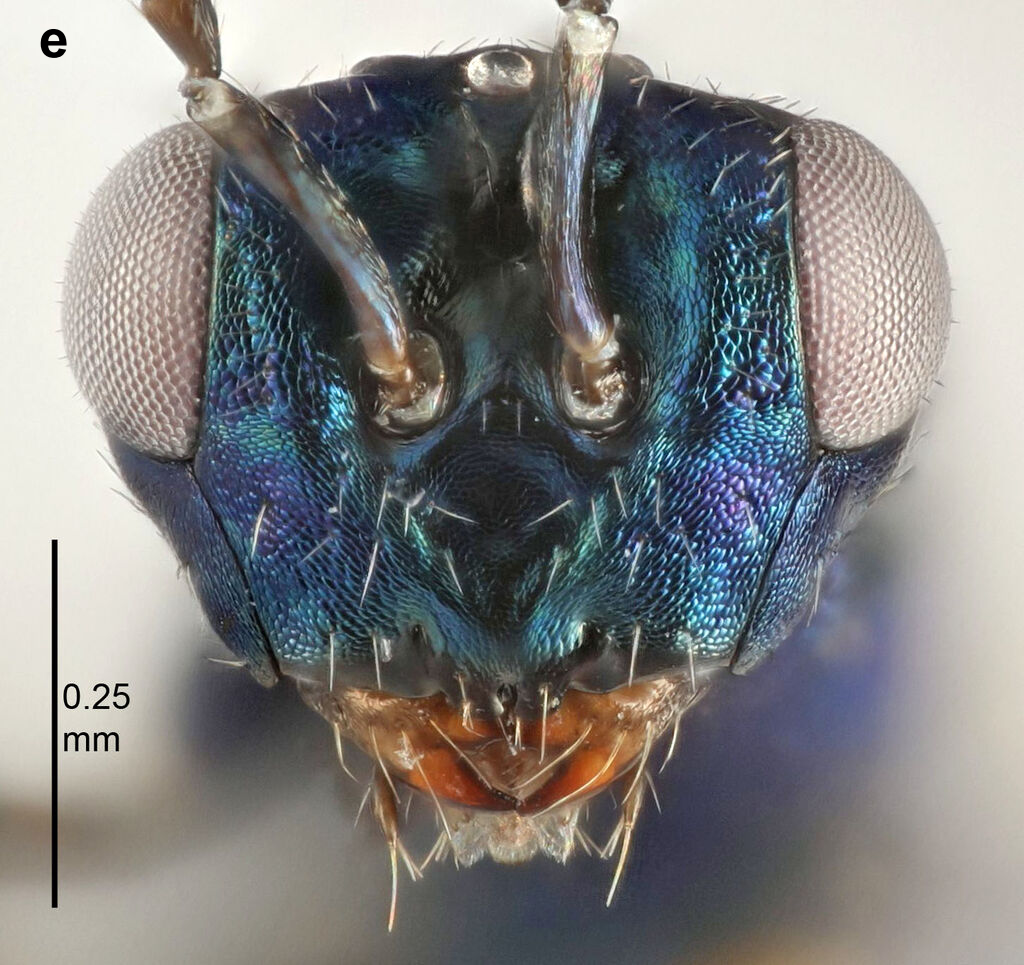
Nontype female, head frontal.

**Figure 20f.**
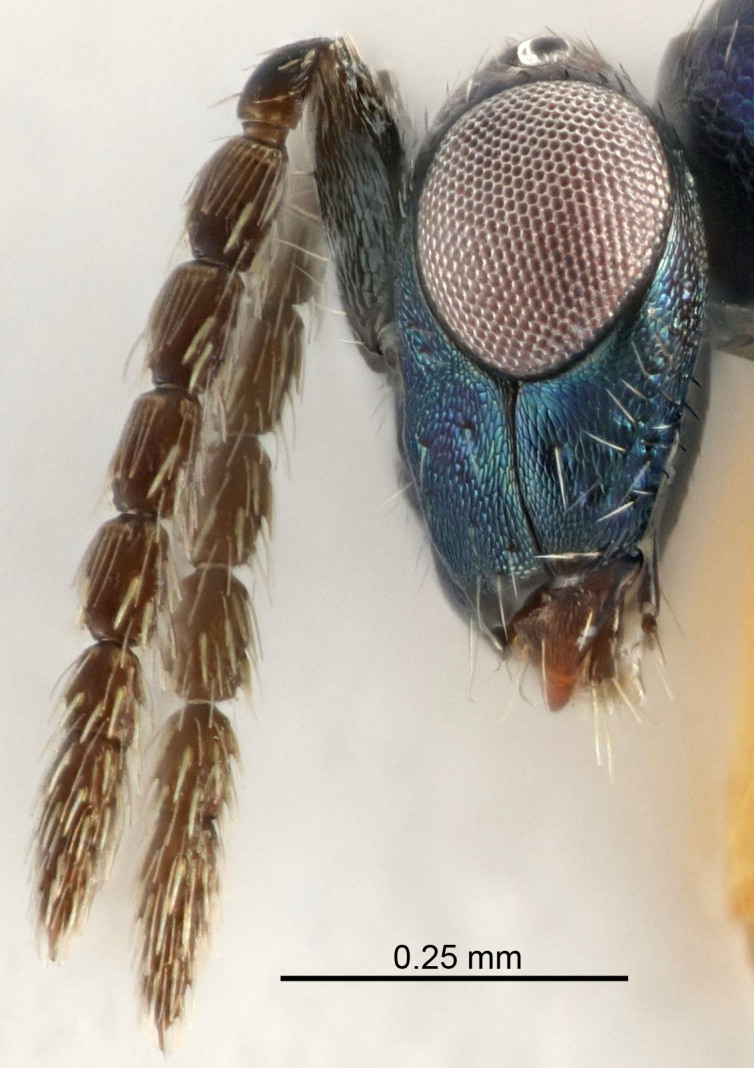
Nontype male, head and antenna lateral.

**Figure 21a.**
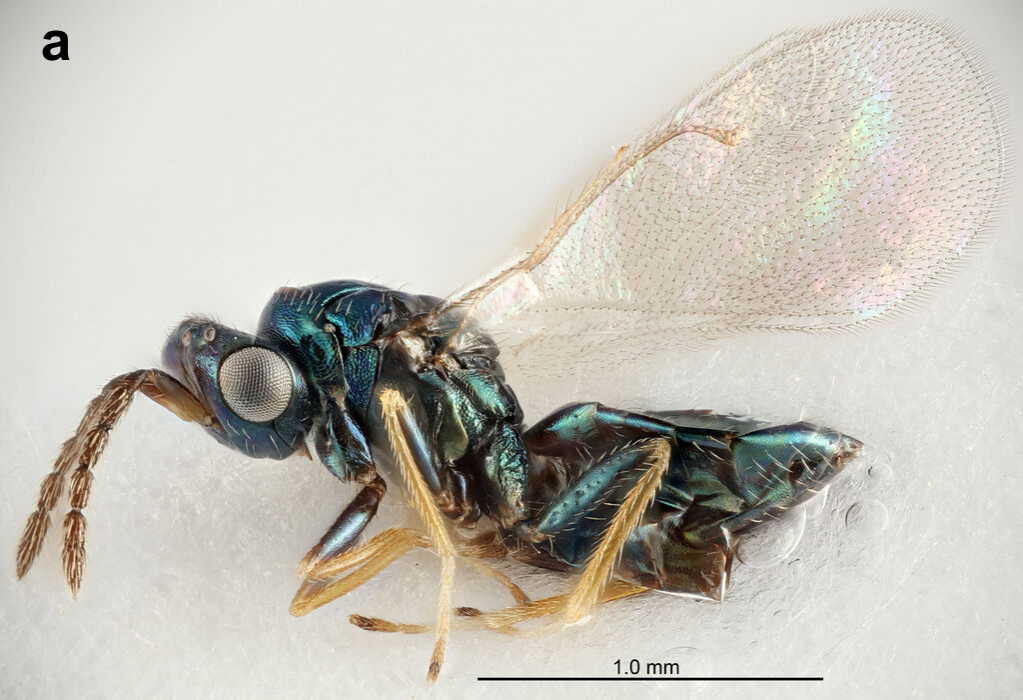
Holotype female, lateral.

**Figure 21b.**
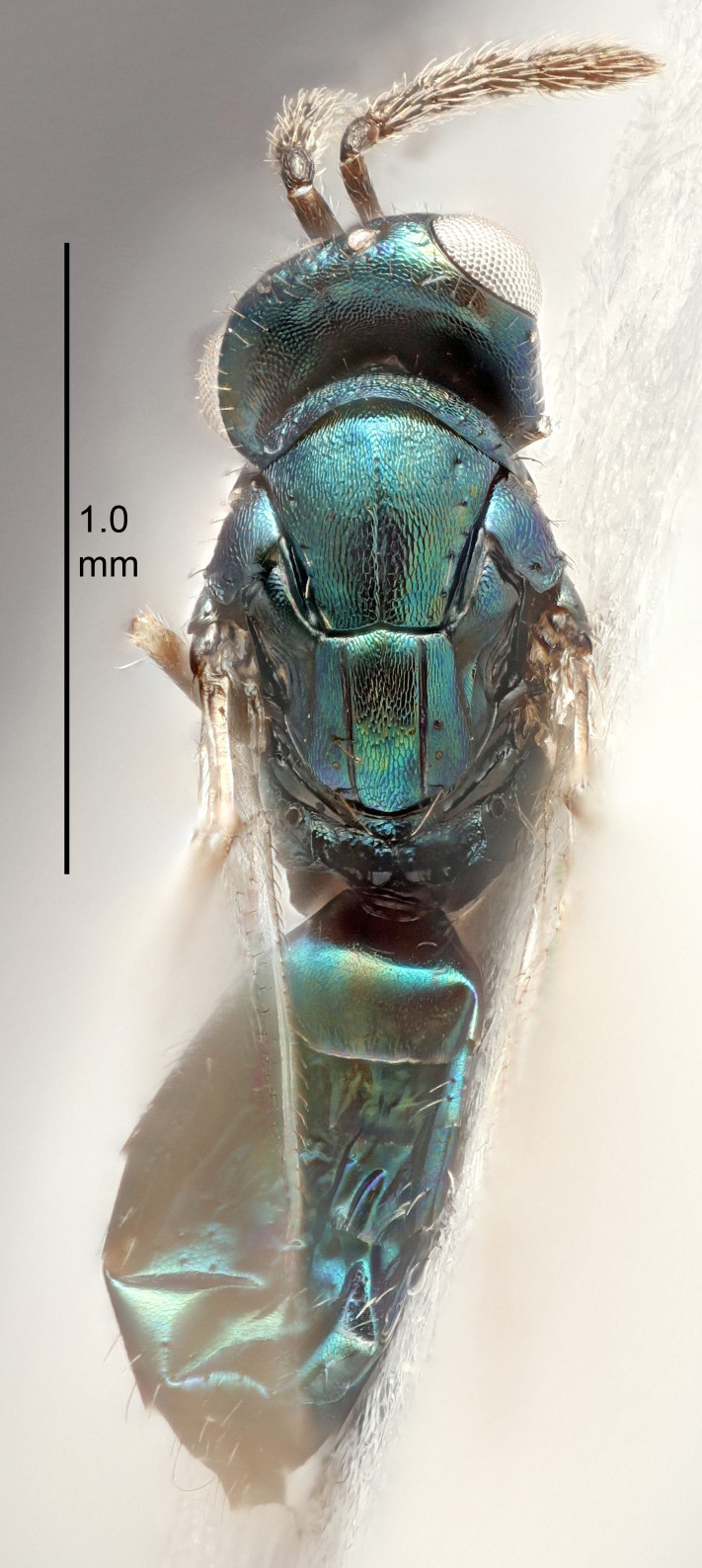
Holotype female, dorsal.

**Figure 21c.**
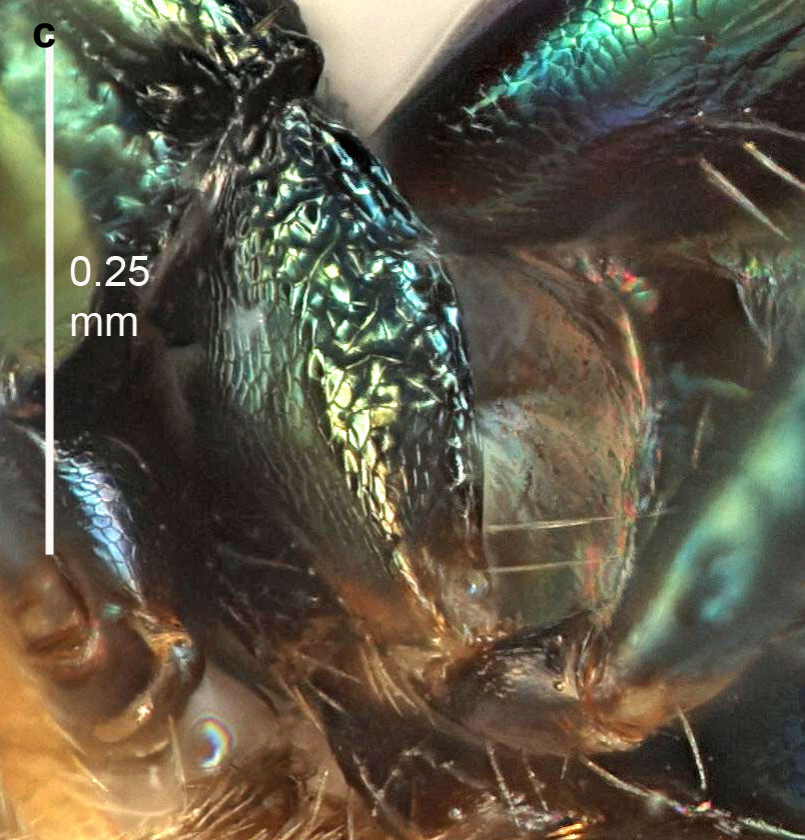
Holotype female, hind coxa, lateral.

**Figure 22a.**
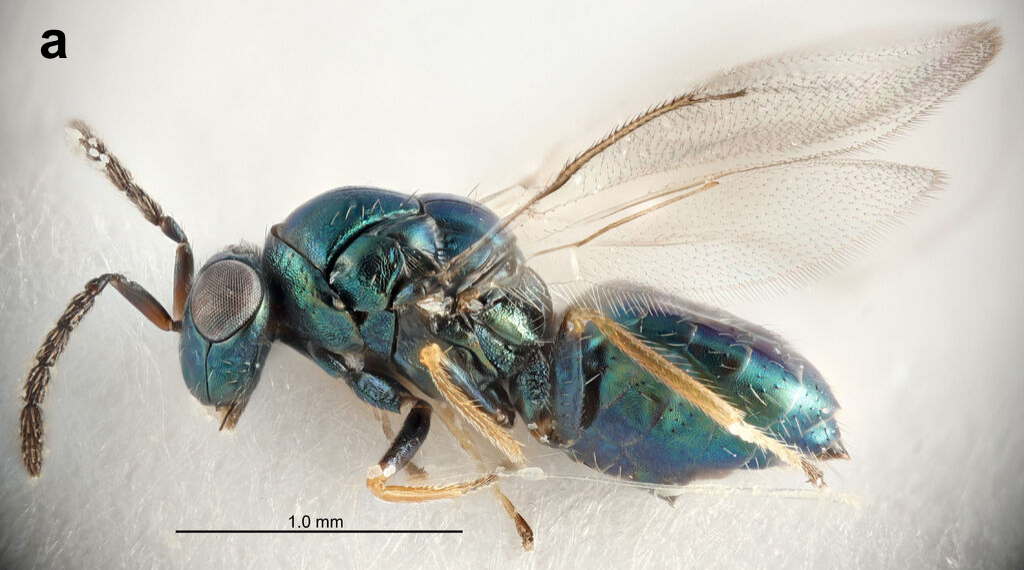
Holotype female, lateral.

**Figure 22b.**
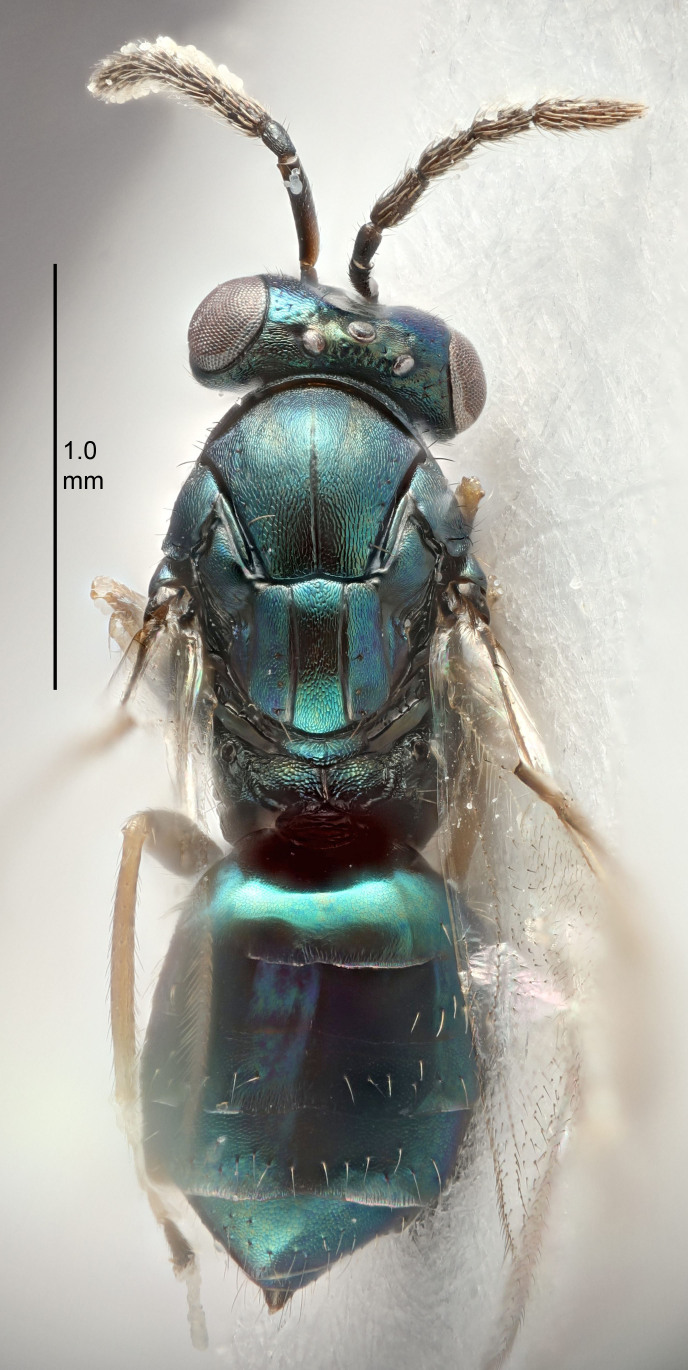
Holotype female, dorsal.

**Figure 22c.**
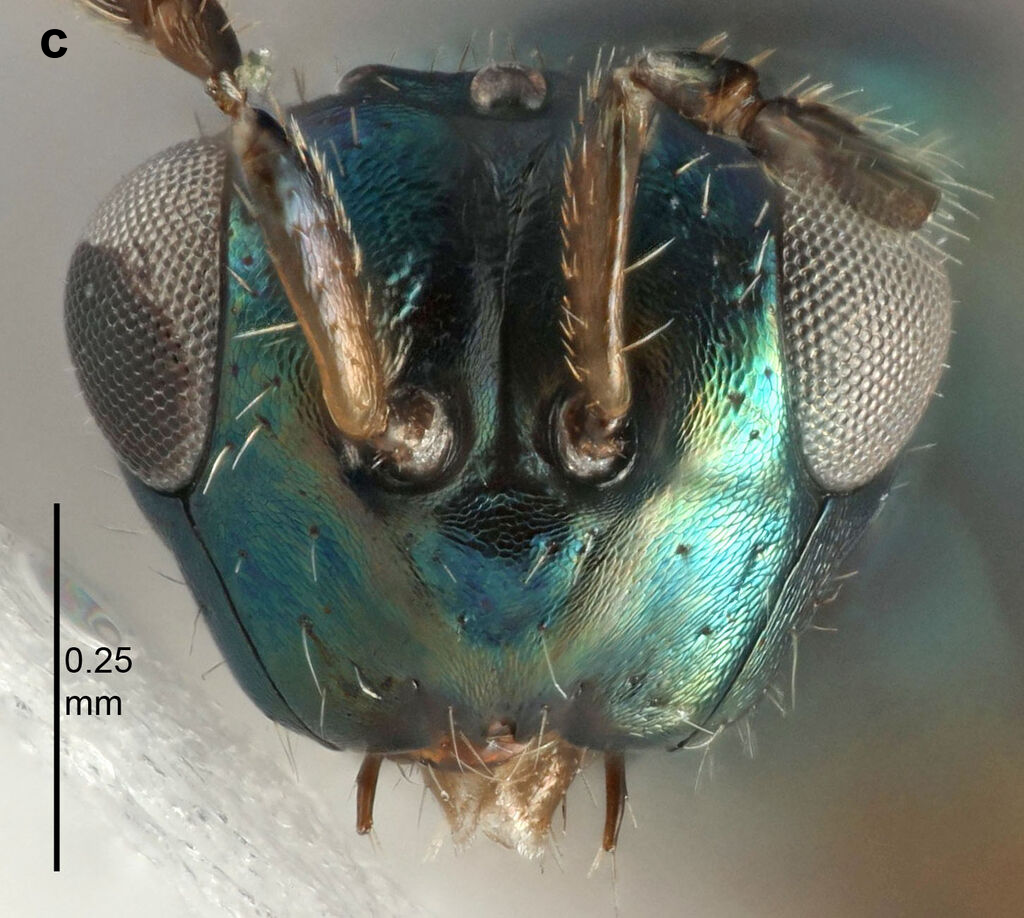
Paratype female, head frontal.

**Figure 22d.**
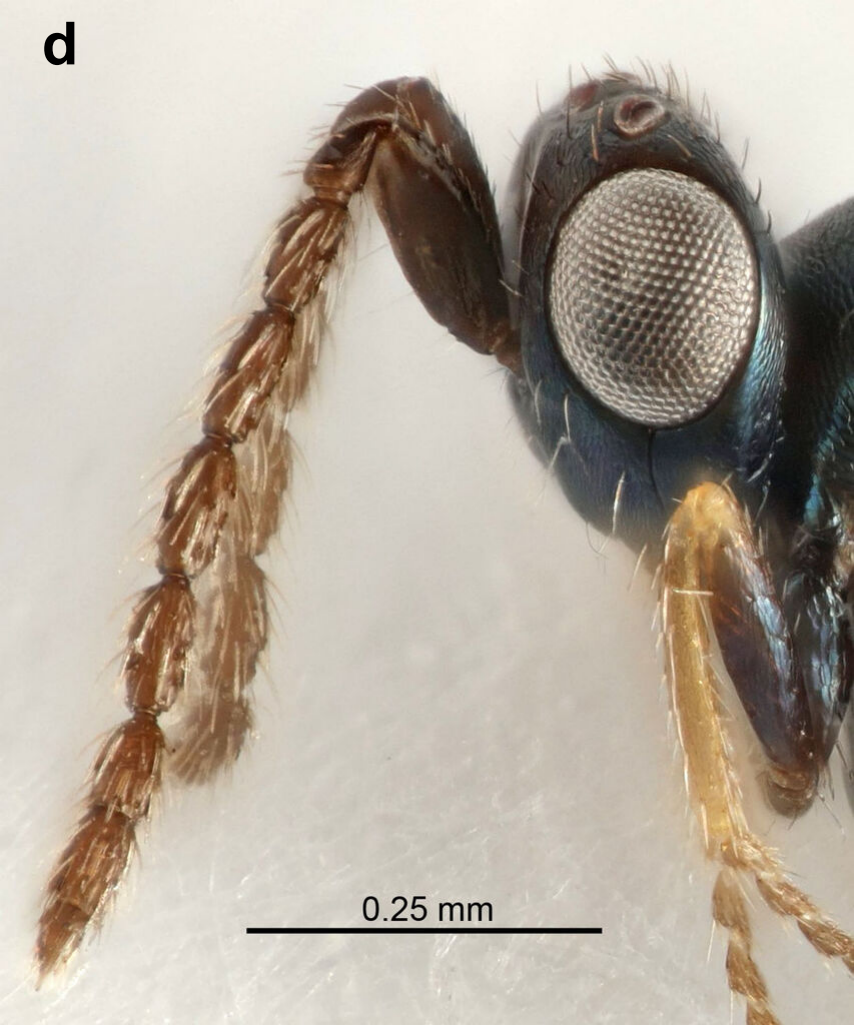
Paratype male, head and antenna lateral.

**Figure 23a.**
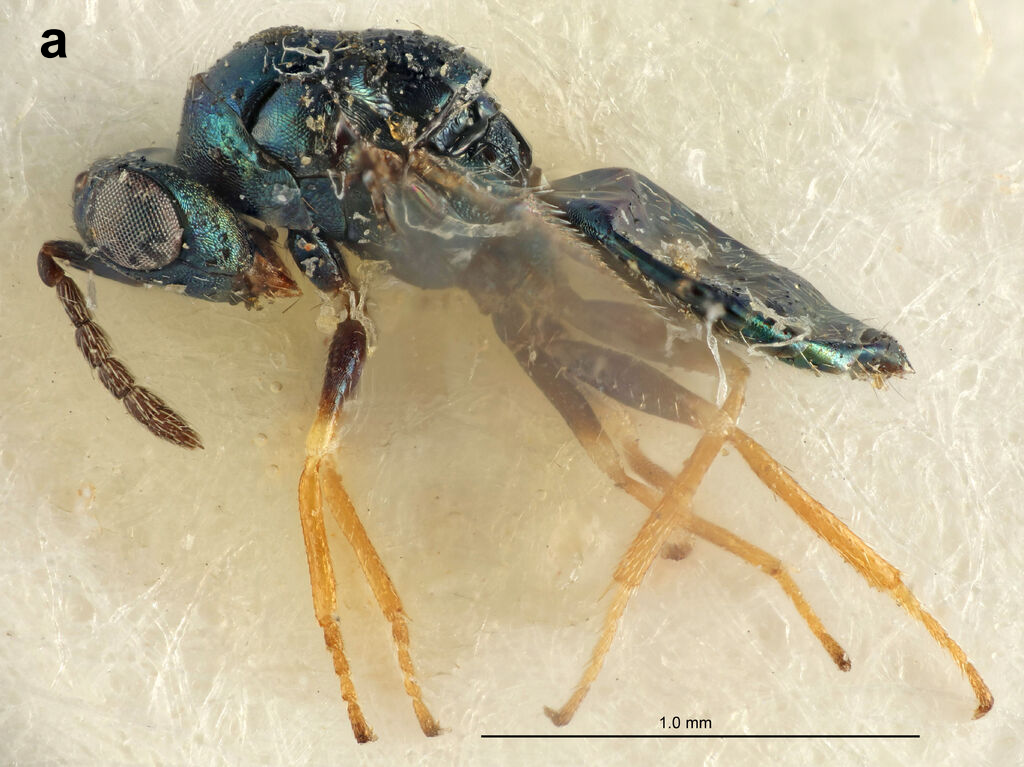
Holotype female, lateral.

**Figure 23b.**
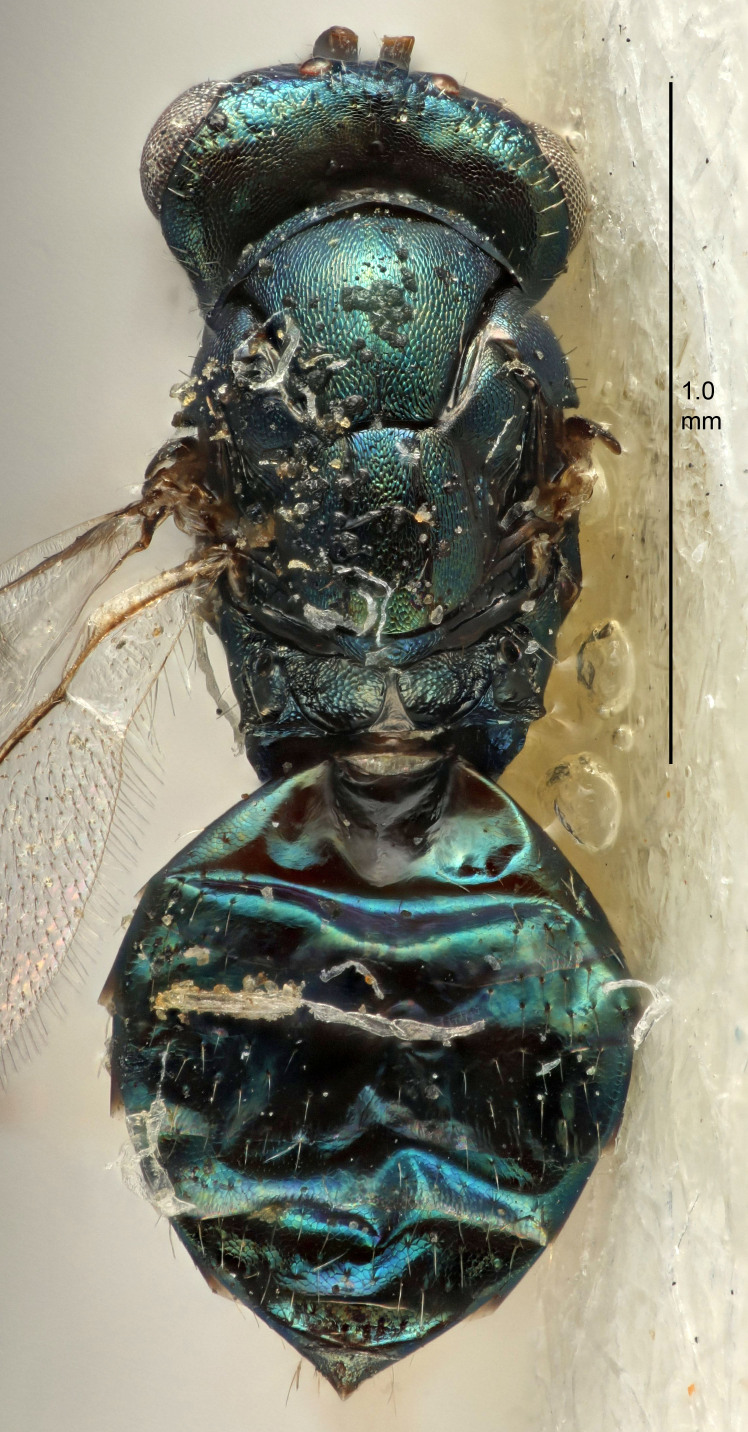
Holotype female, dorsal.

**Figure 24a.**
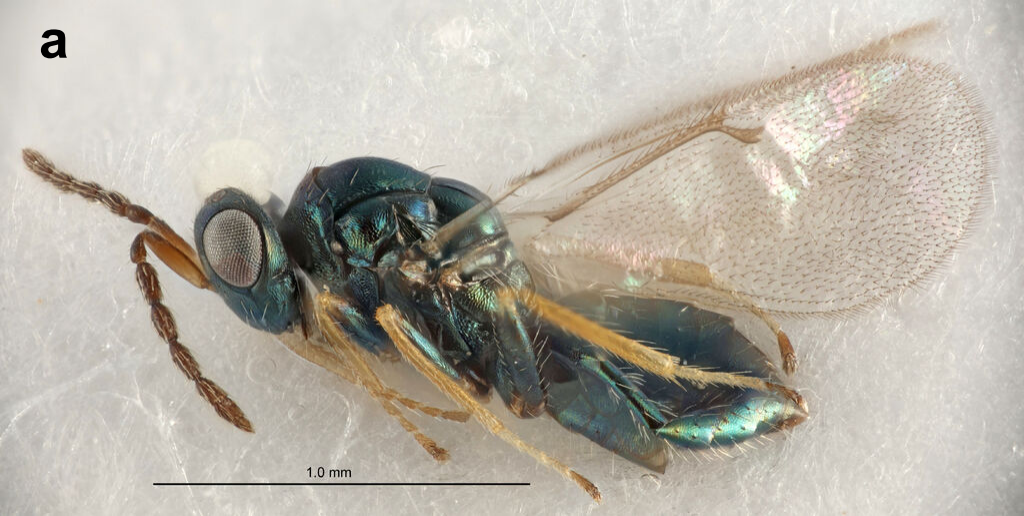
Holotype female, lateral.

**Figure 24b.**
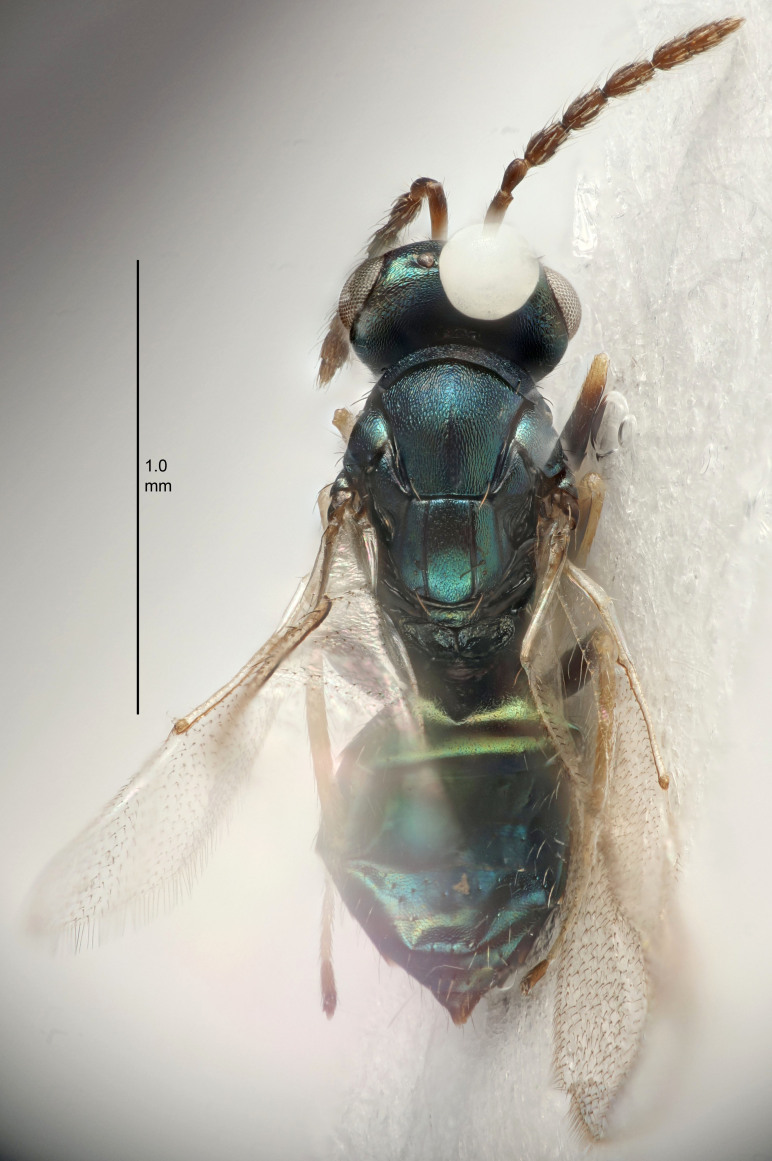
Holotype female, dorsal.

**Figure 25a.**
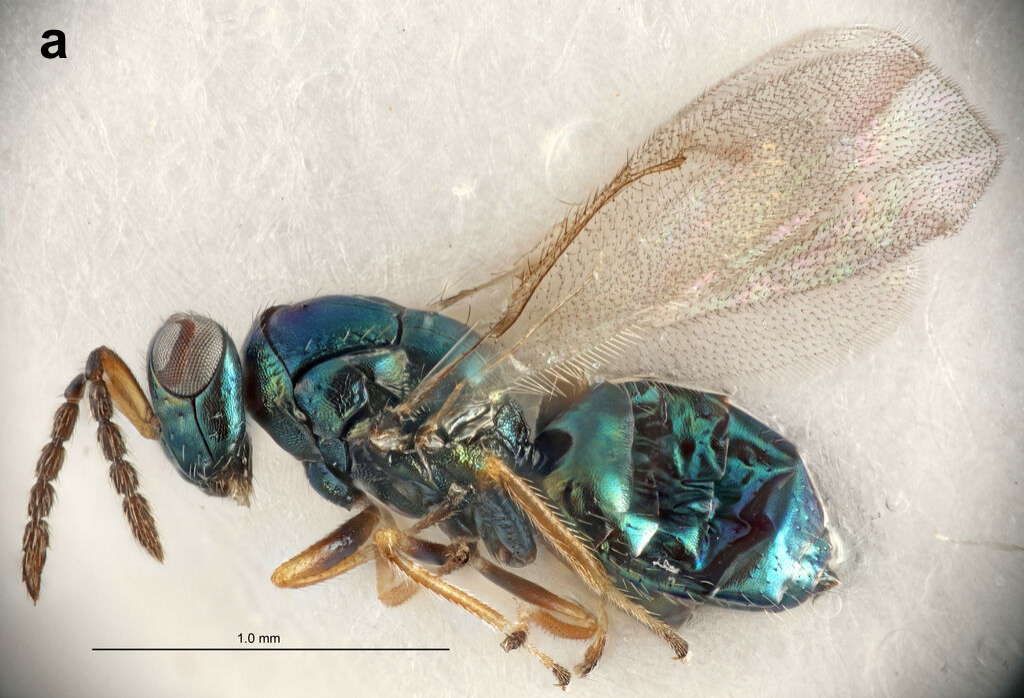
Holotype female, lateral.

**Figure 25b.**
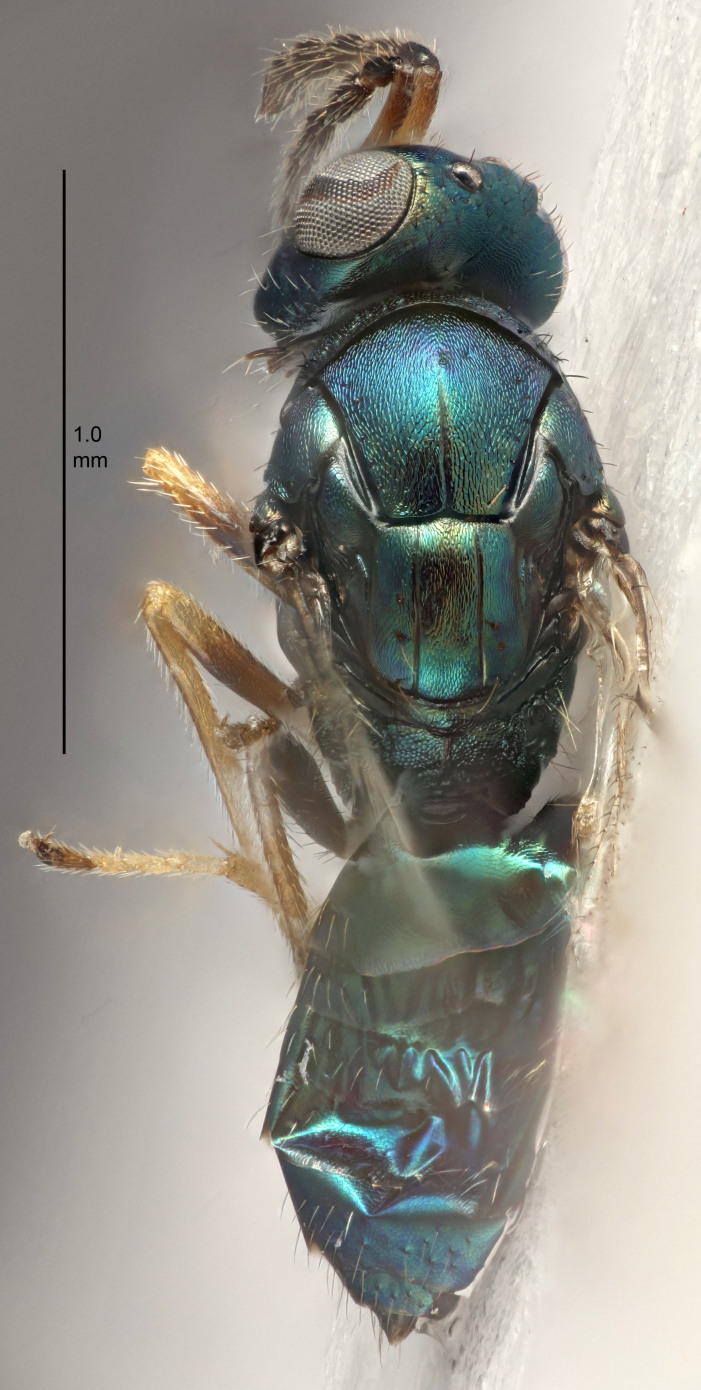
Holotype female, dorsal.

**Figure 26a.**
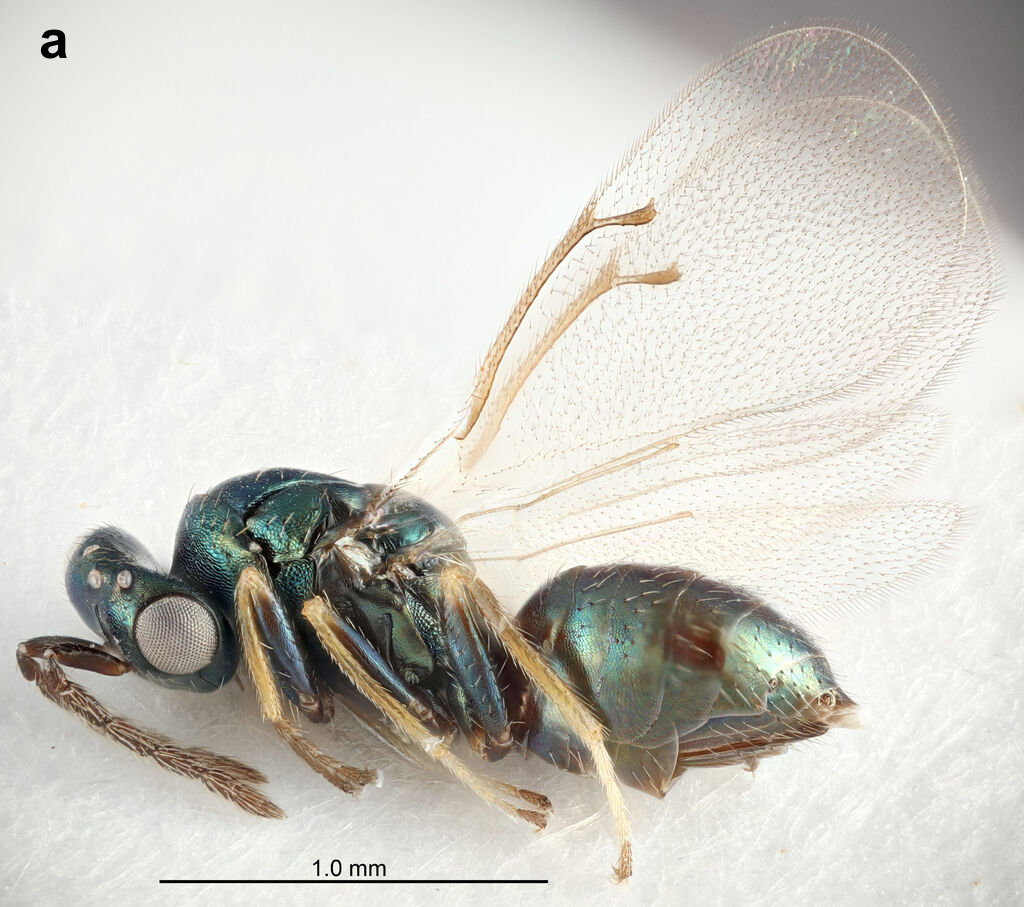
Holotype female, lateral.

**Figure 26b.**
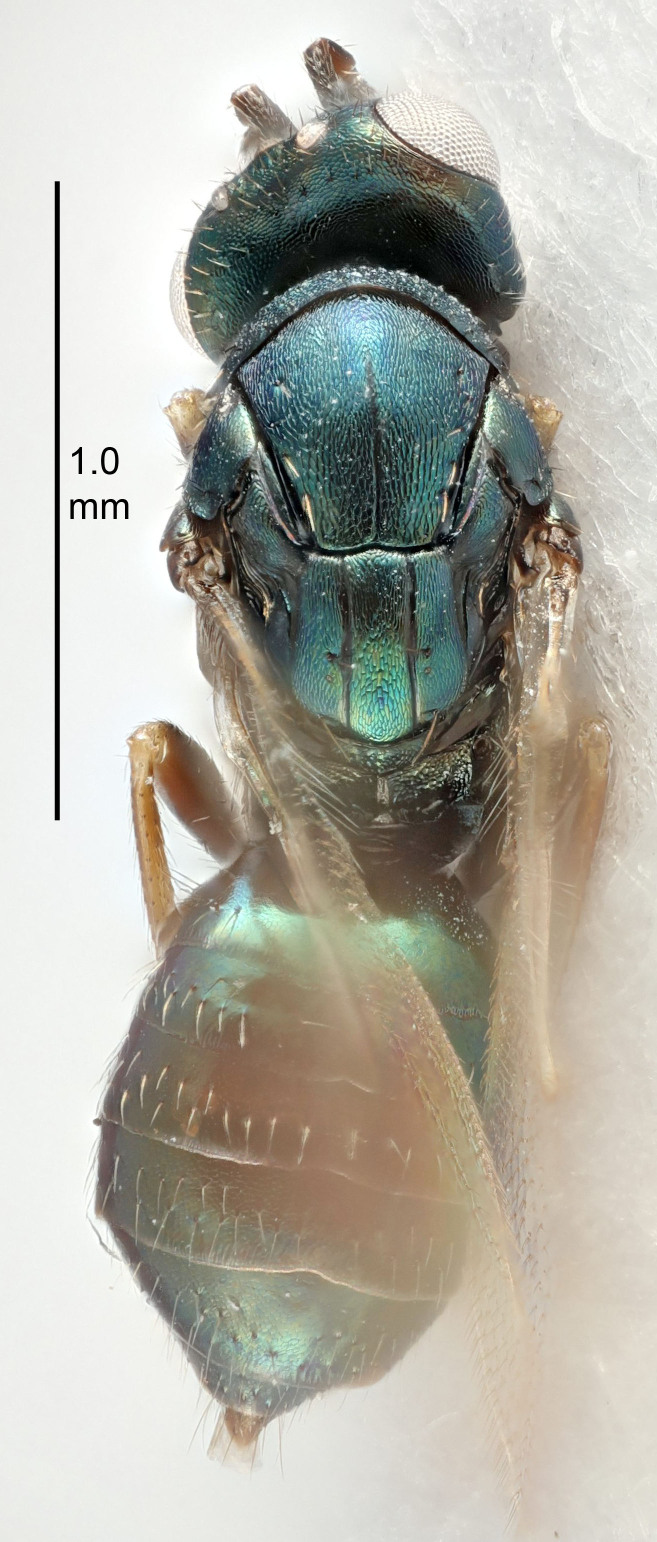
Holotype female, dorsal.

**Figure 27a.**
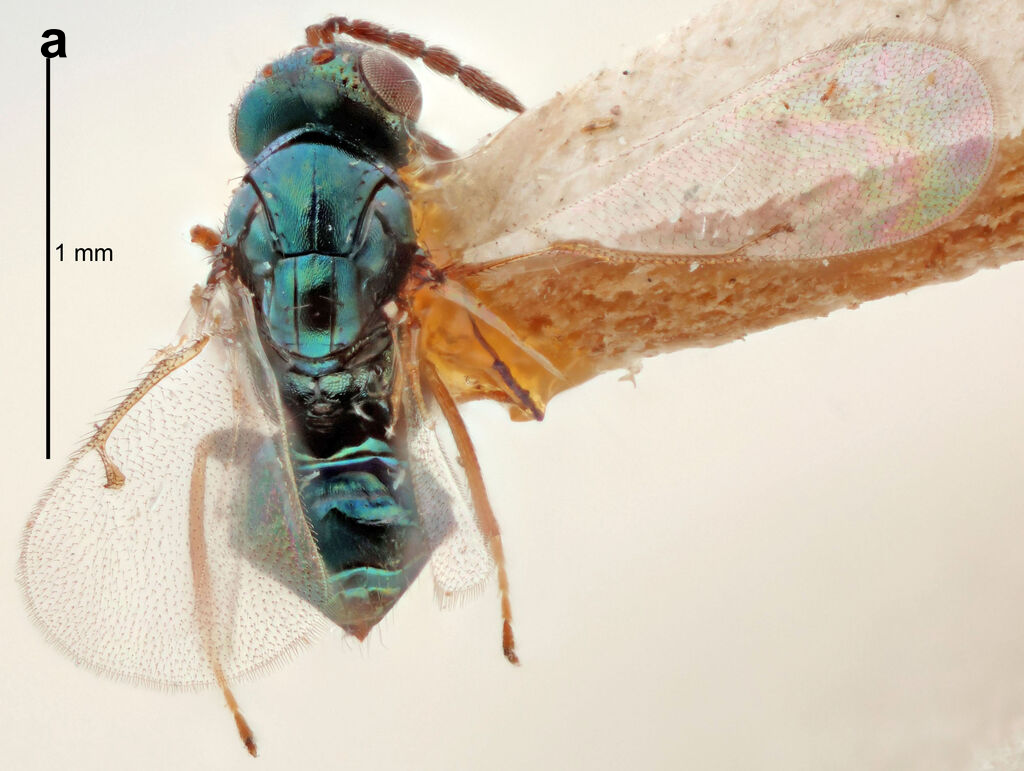
Holotype female, dorsal.

**Figure 27b.**
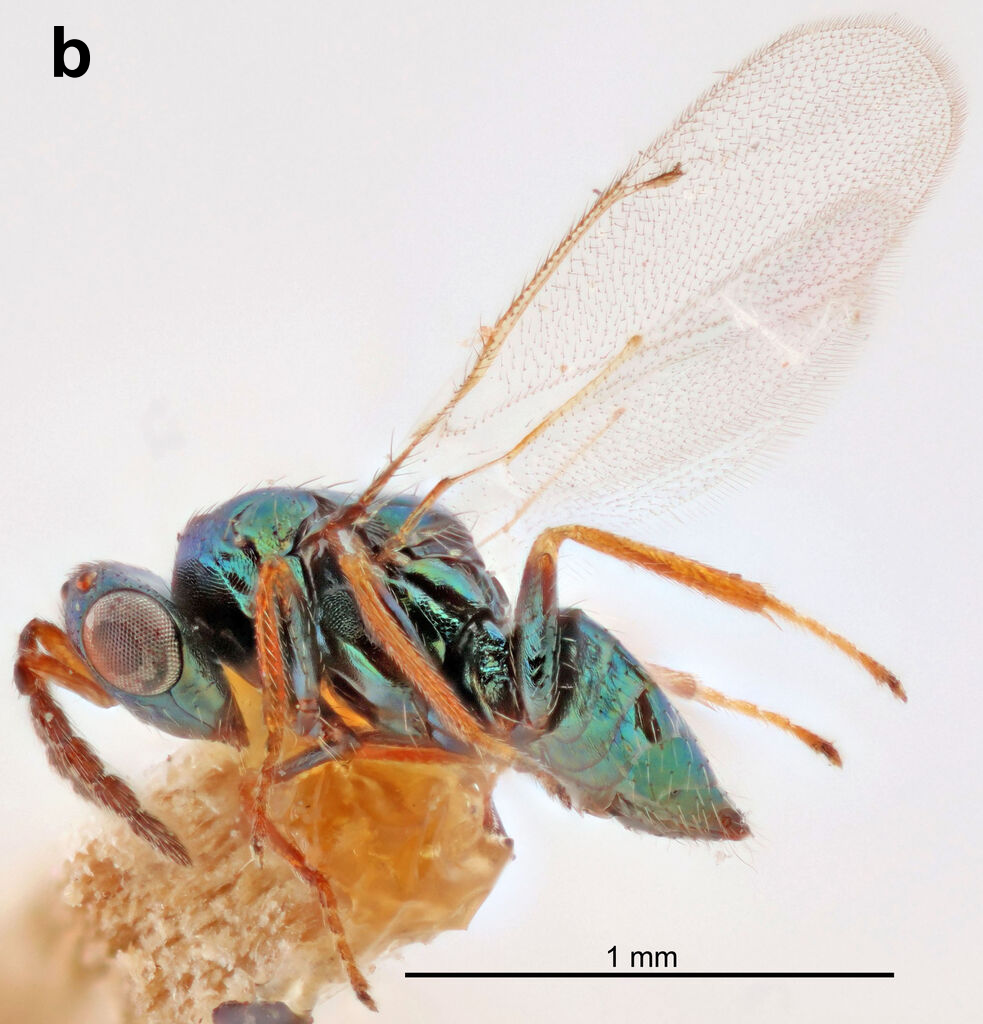
Holotype female, lateral.

**Figure 28a.**
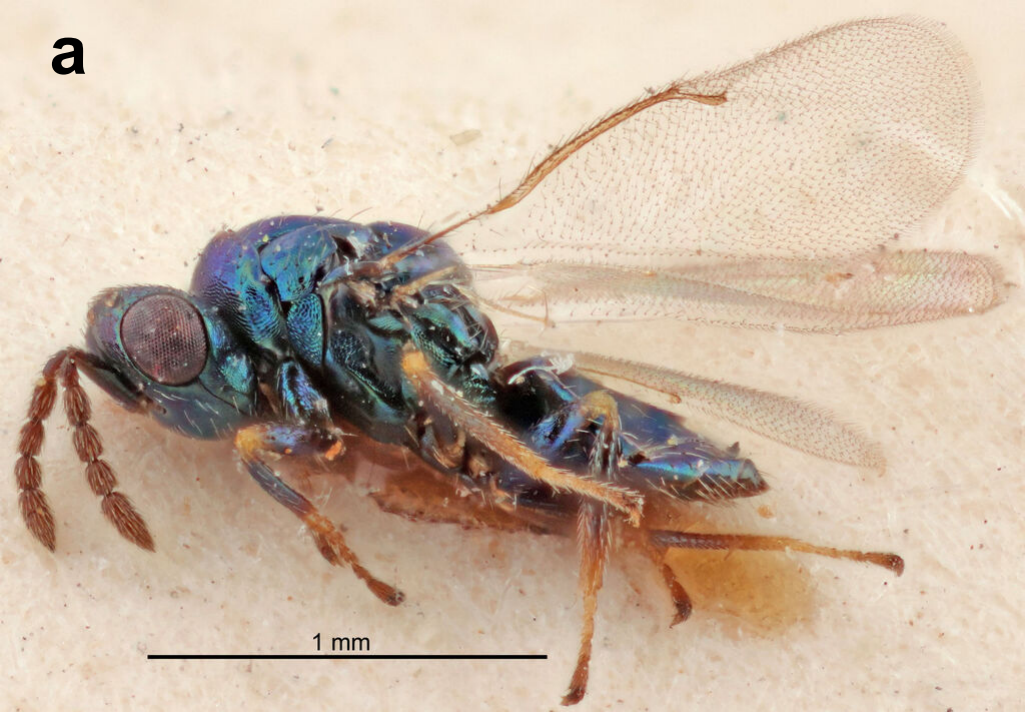
Neotype female, lateral.

**Figure 28b.**
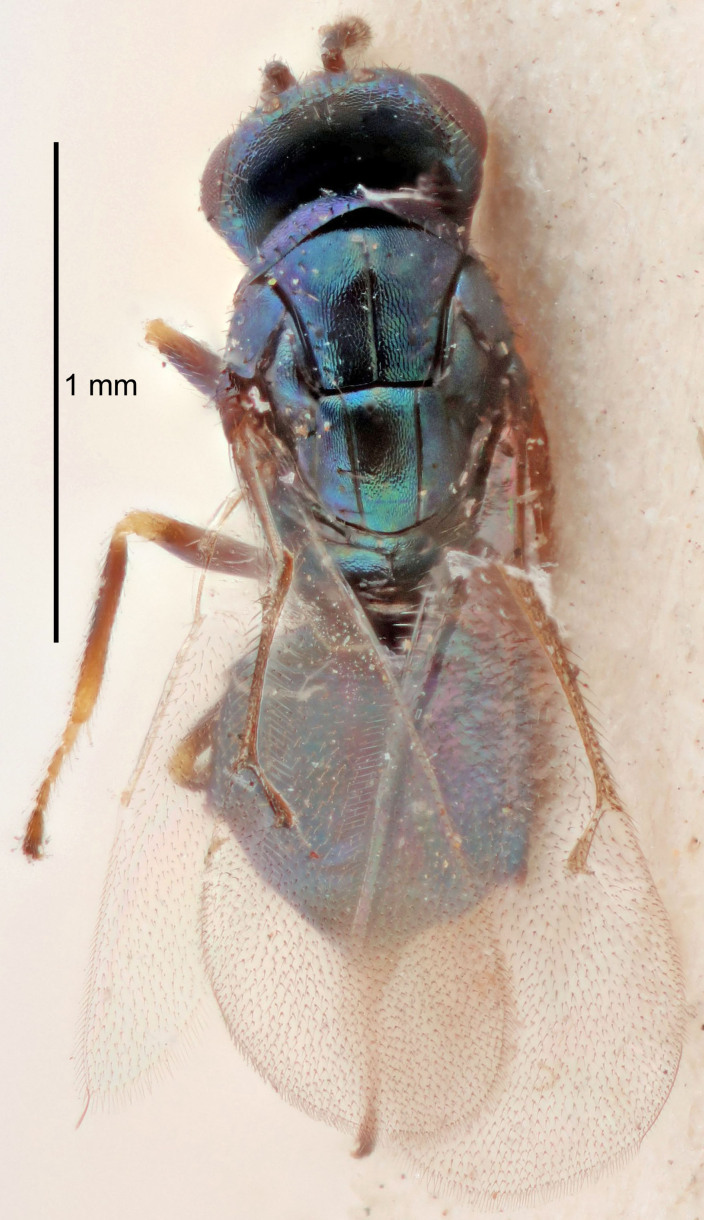
Neotype female, dorsal.

**Figure 28c.**
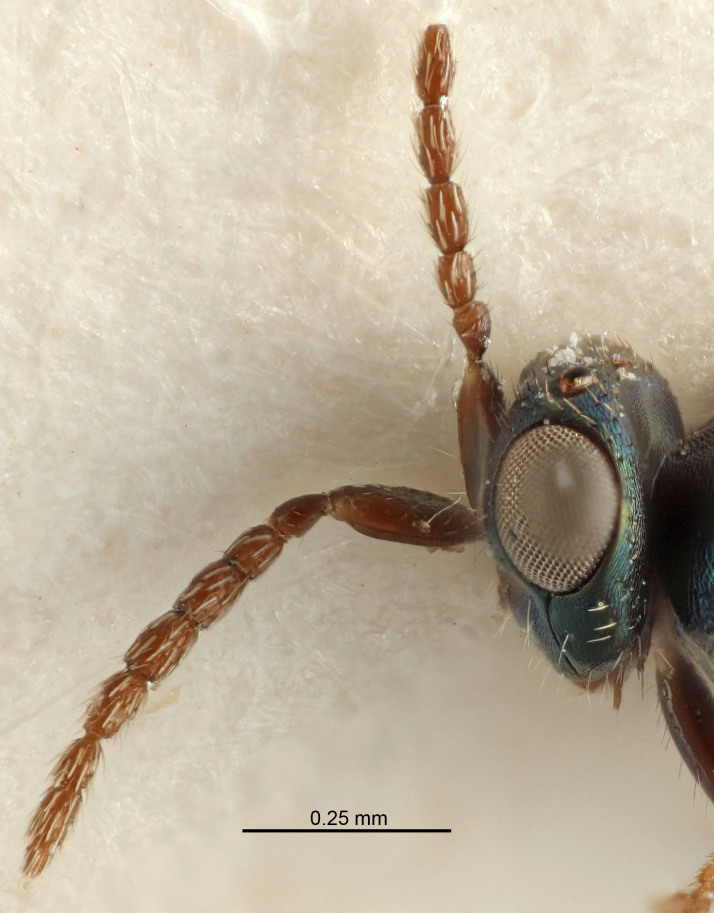
Nontype male, head and antenna lateral.

**Figure 29a.**
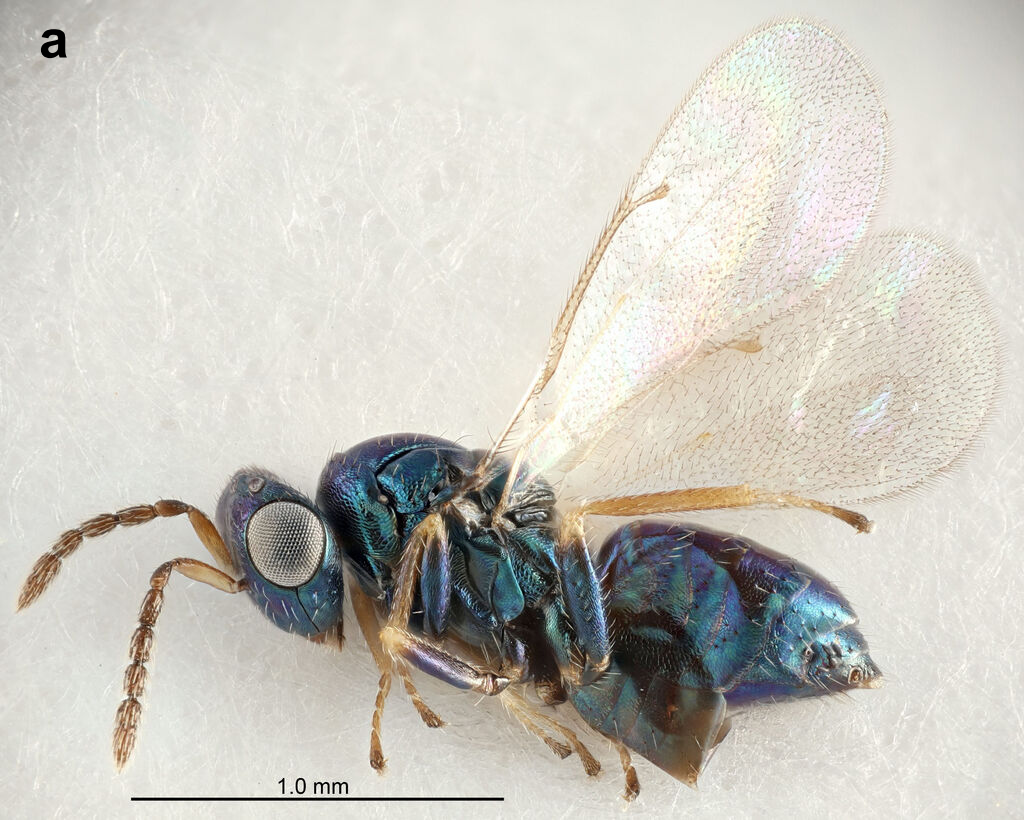
Holotype female, lateral.

**Figure 29b.**
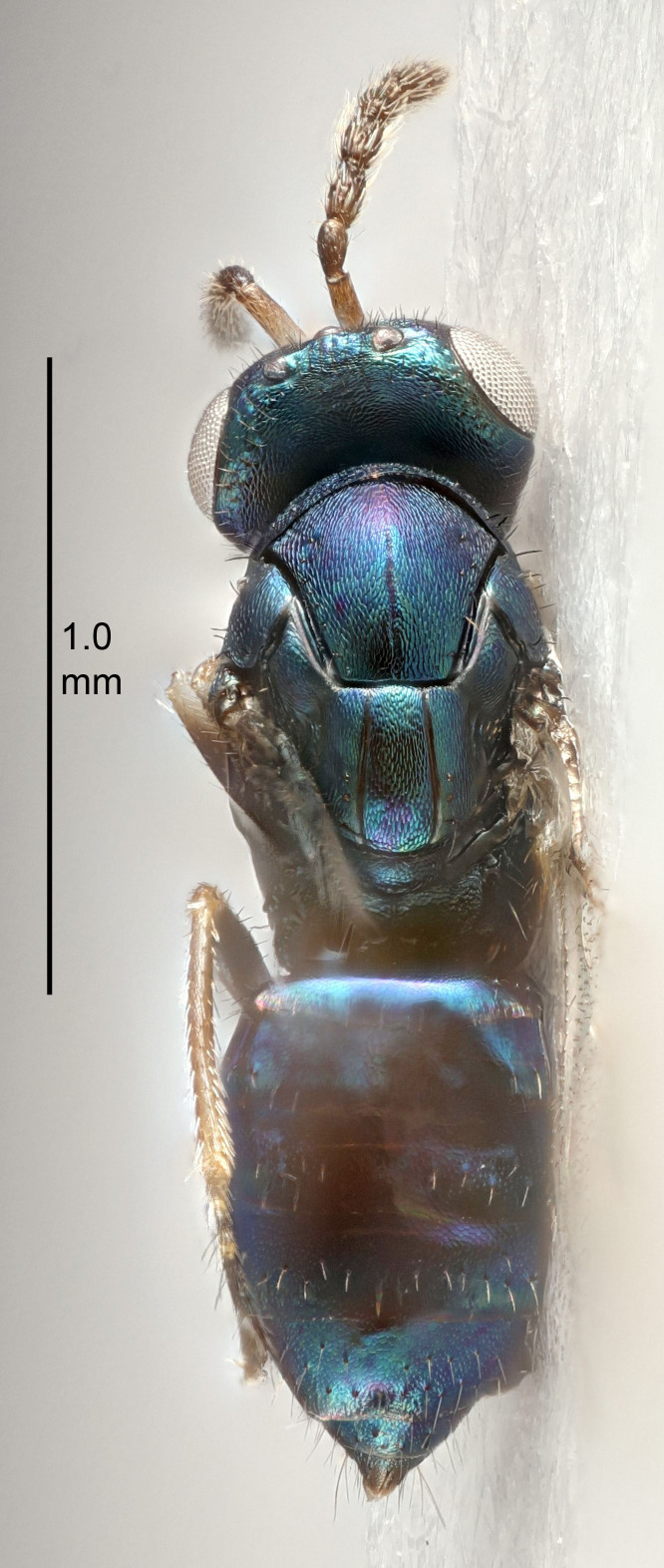
Holotype female, dorsal.

**Figure 29c.**
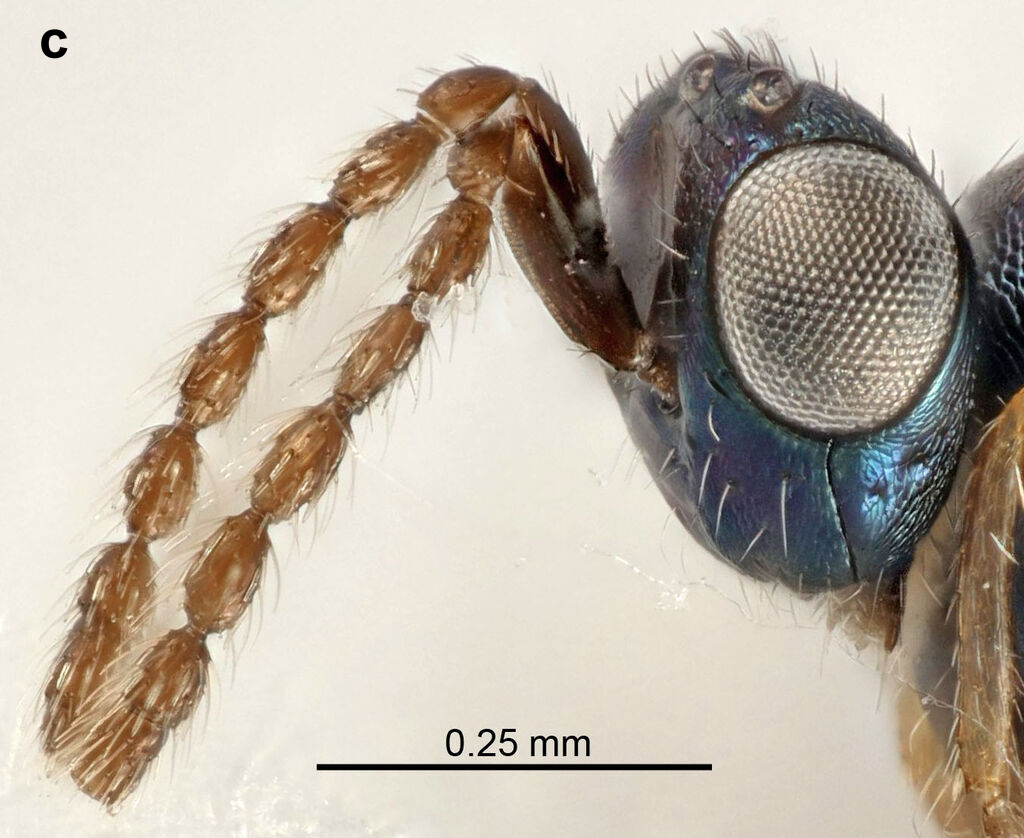
Paratype male, head and antenna lateral.

**Figure 30a.**
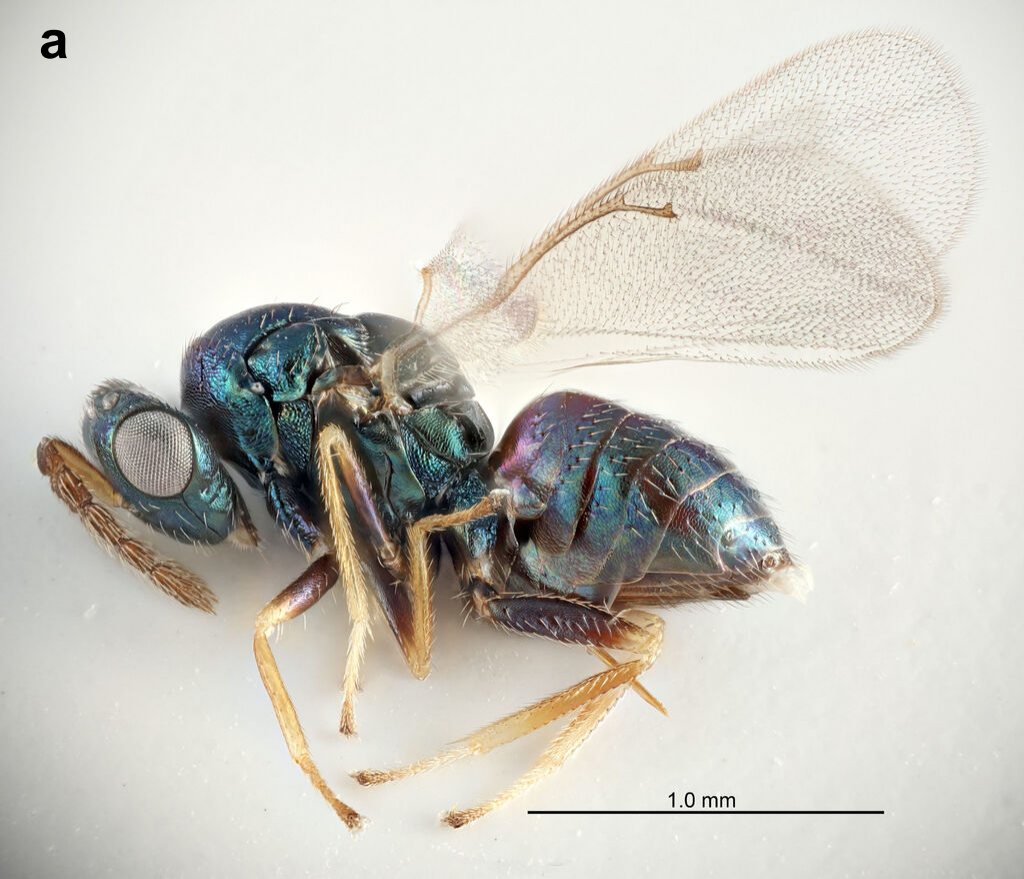
Holotype female, lateral.

**Figure 30b.**
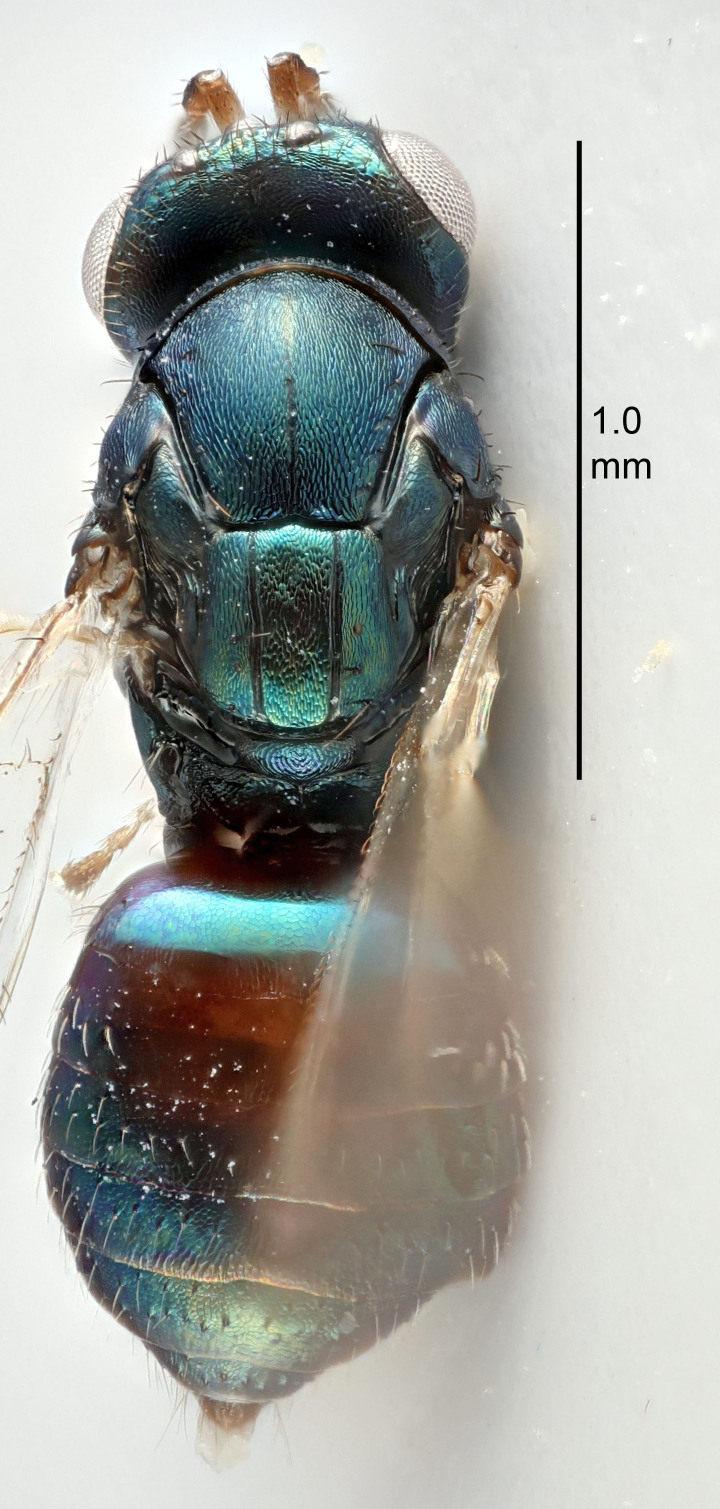
Holotype female, dorsal.

**Figure 31a.**
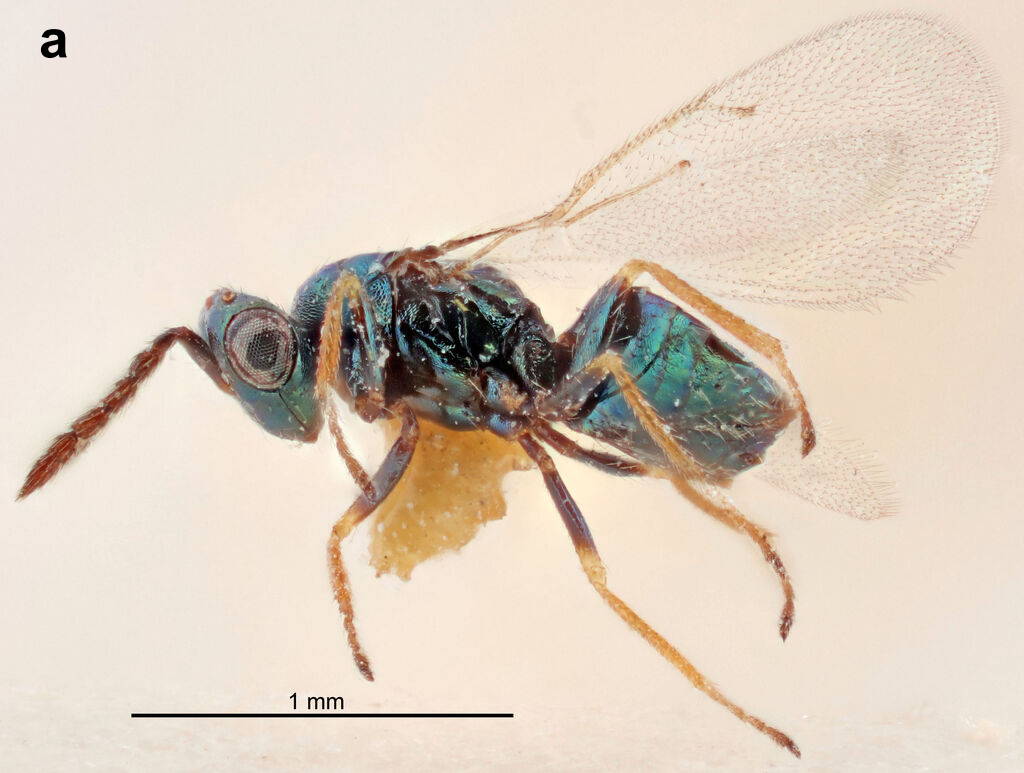
Holotype female, lateral.

**Figure 31b.**
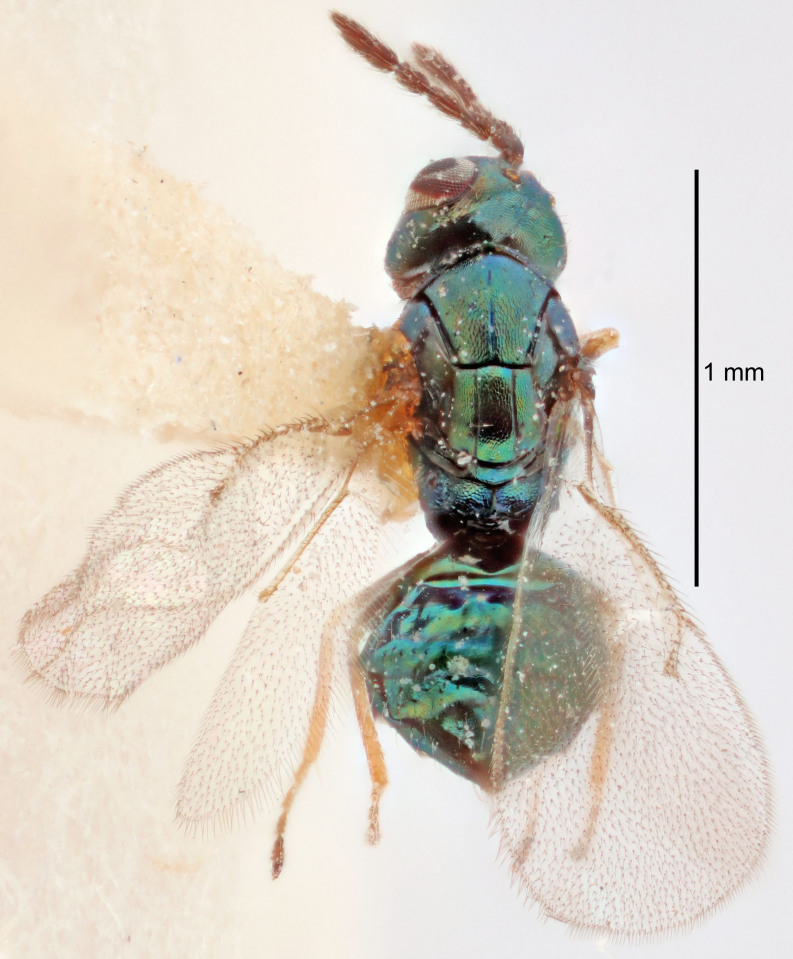
Holotype female, dorsal.

**Figure 32a.**
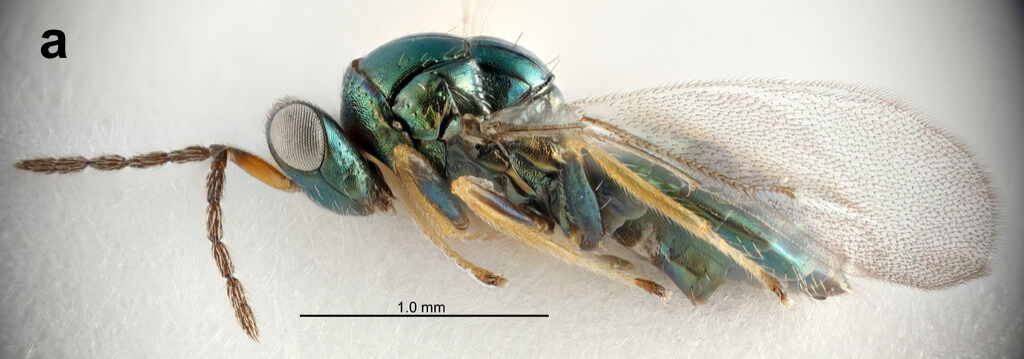
Holotype female, lateral.

**Figure 32b.**
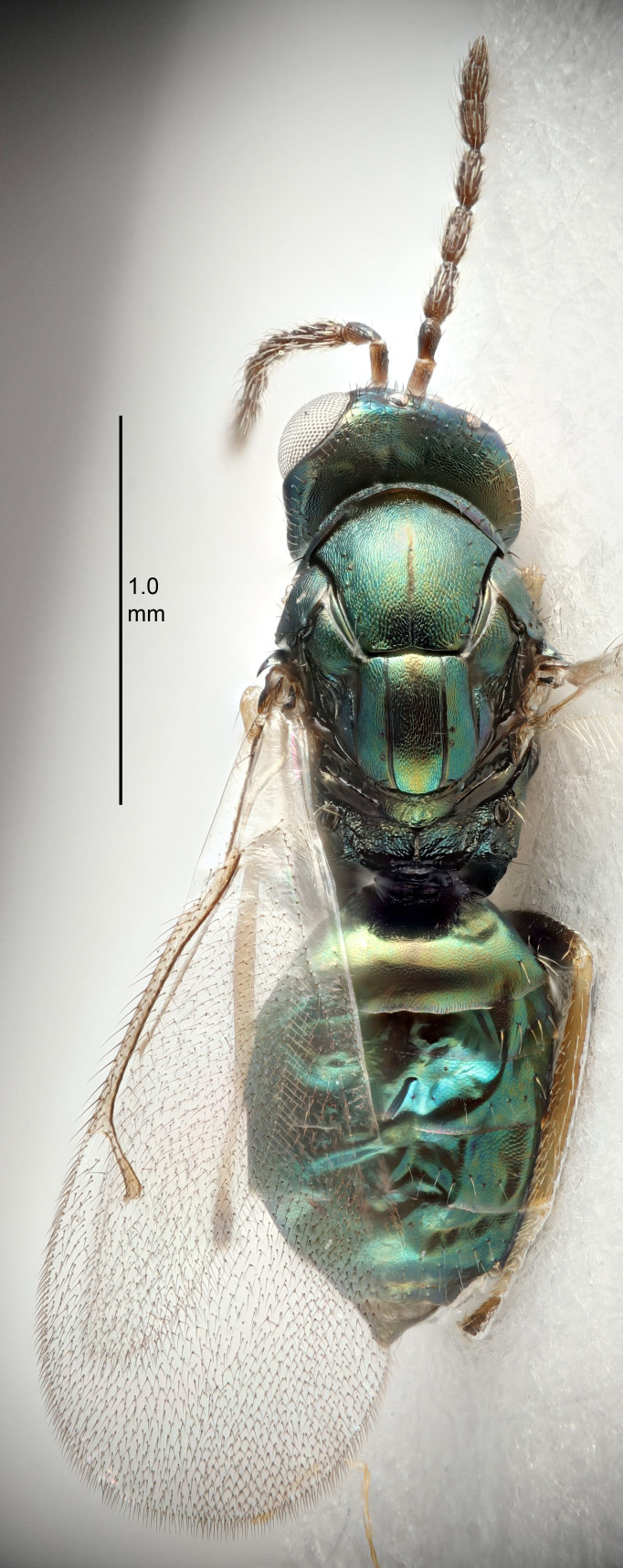
Holotype female, dorsal.

**Figure 32c.**
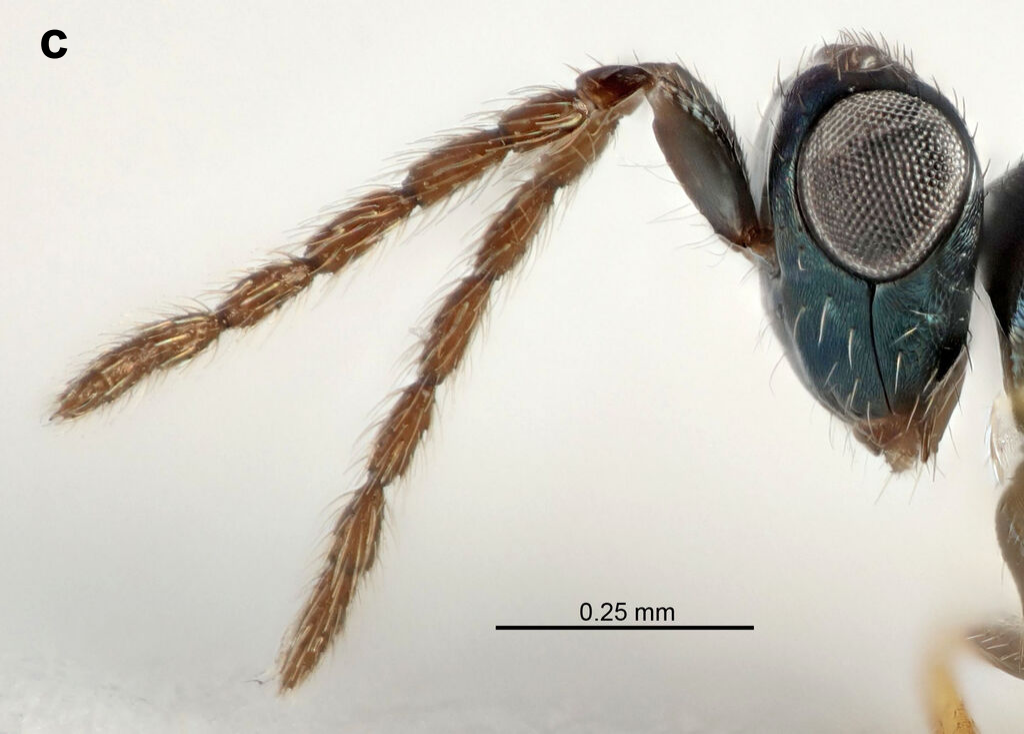
Paratype male, head and antenna lateral.

**Figure 33a.**
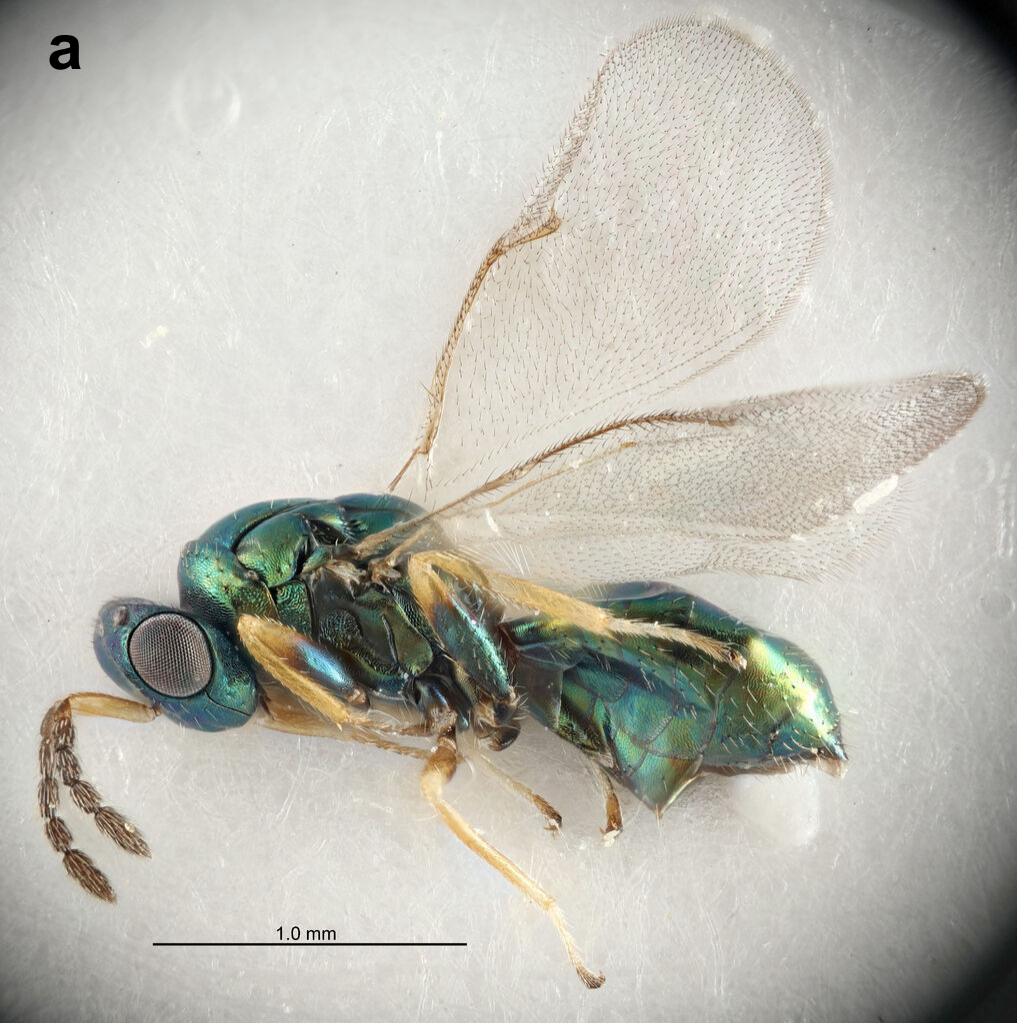
Holotype female, lateral.

**Figure 33b.**
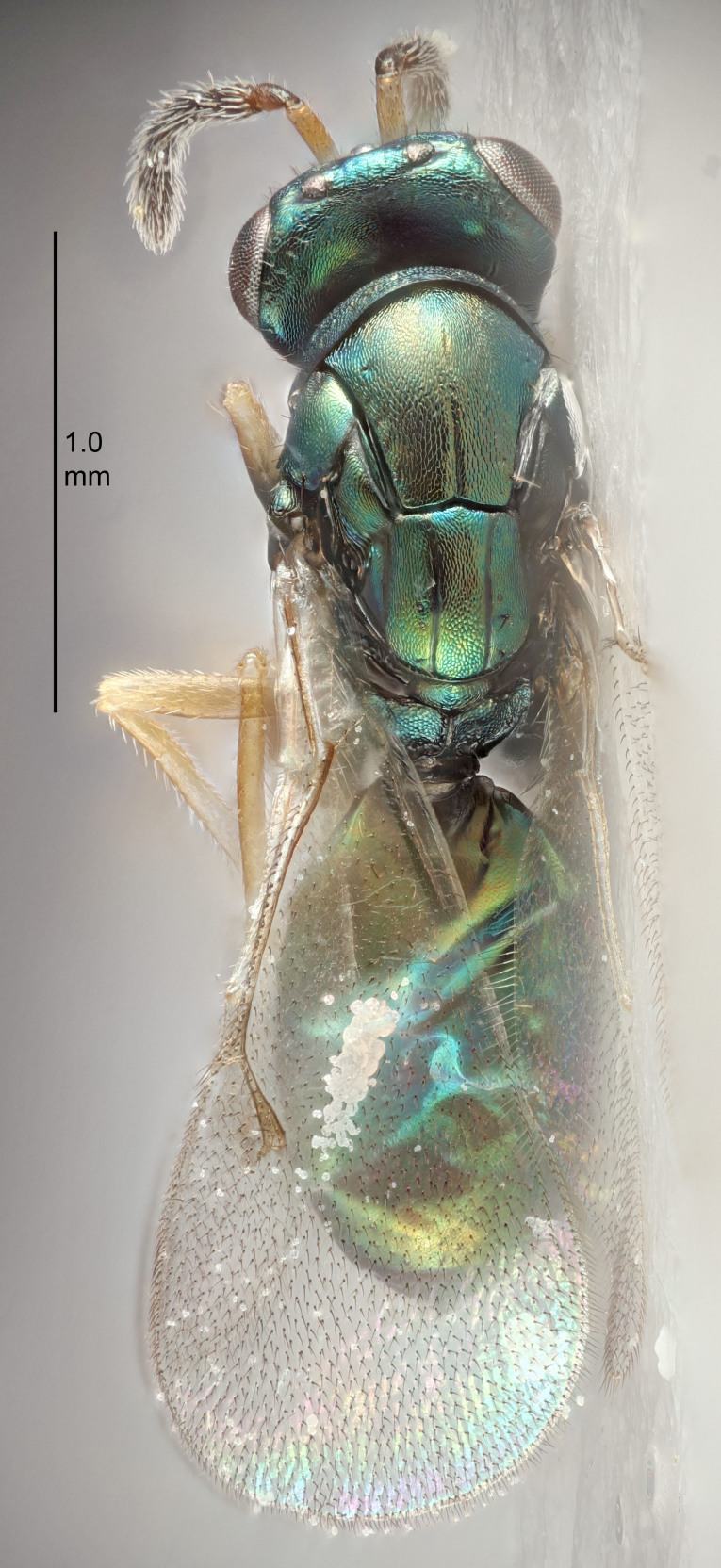
Holotype female, dorsal.

**Figure 33c.**
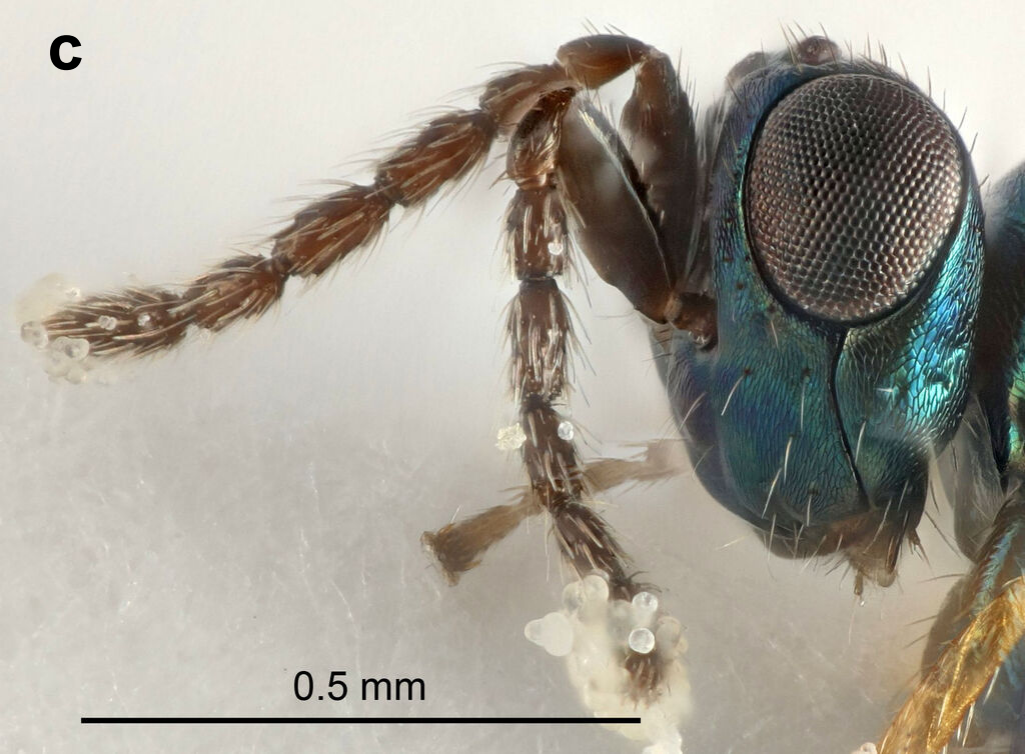
Paratype male, head and antenna lateral.

**Figure 34a.**
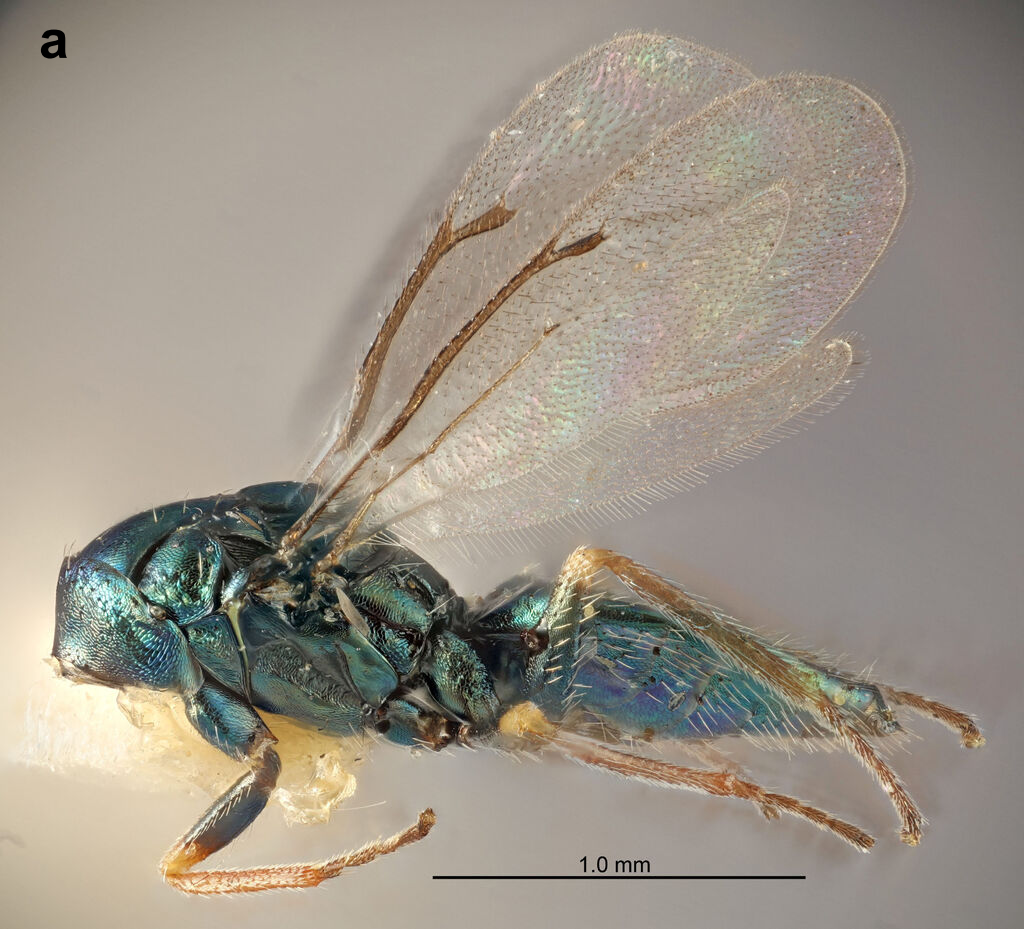
Holotype female, lateral (excl. head).

**Figure 34b.**
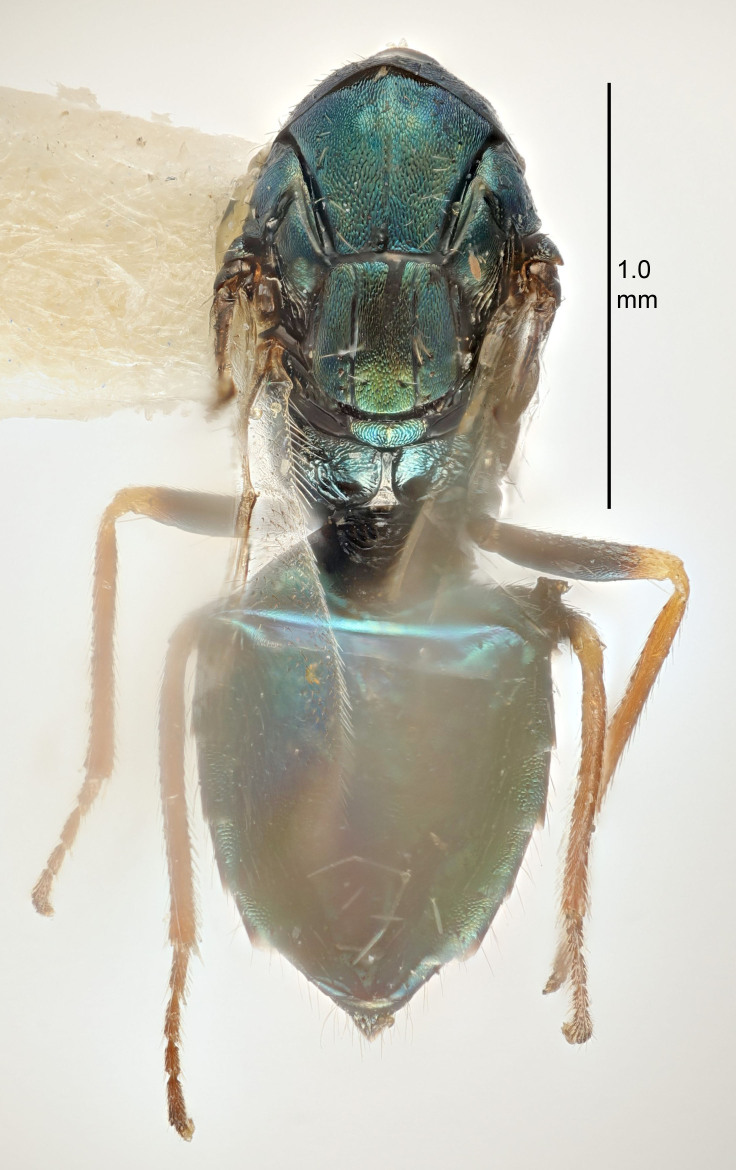
Holotype female, dorsal (excl. head).

**Figure 34c.**
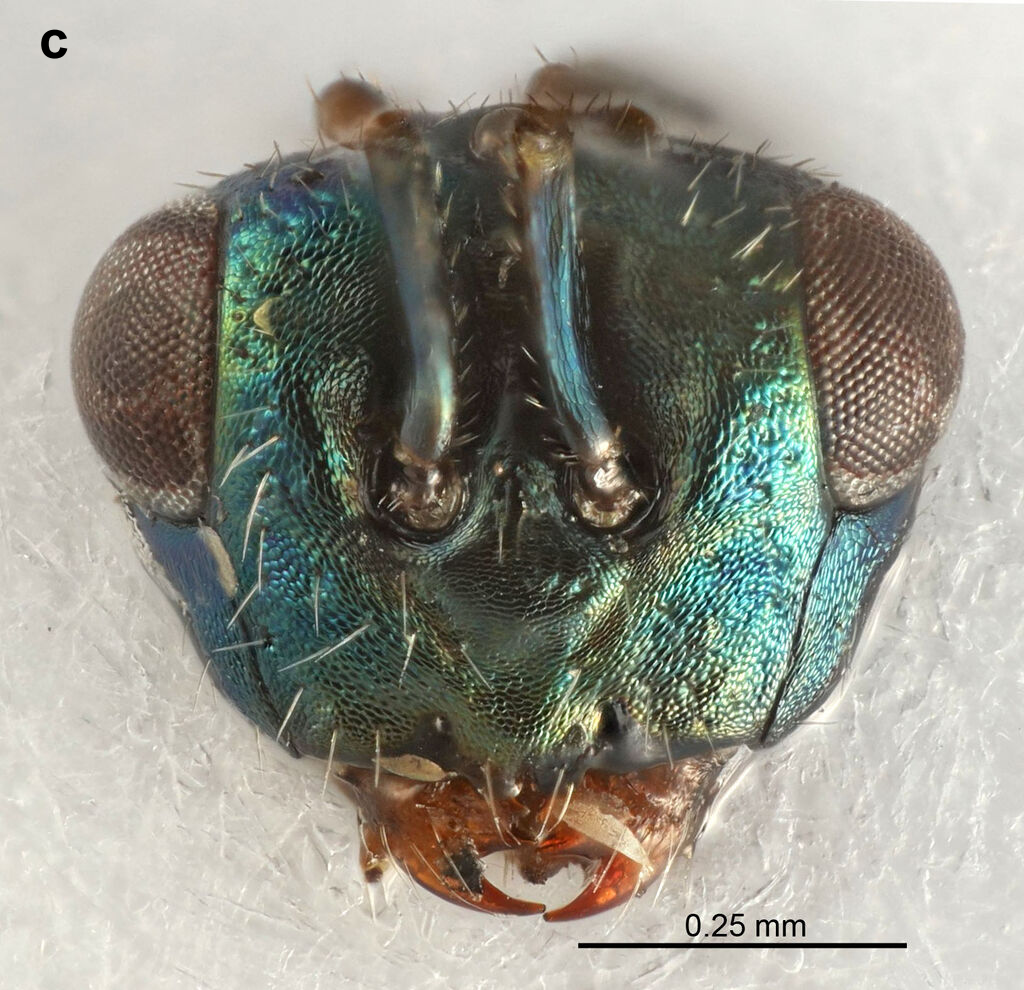
Holotype female, head frontal.

**Figure 34d.**
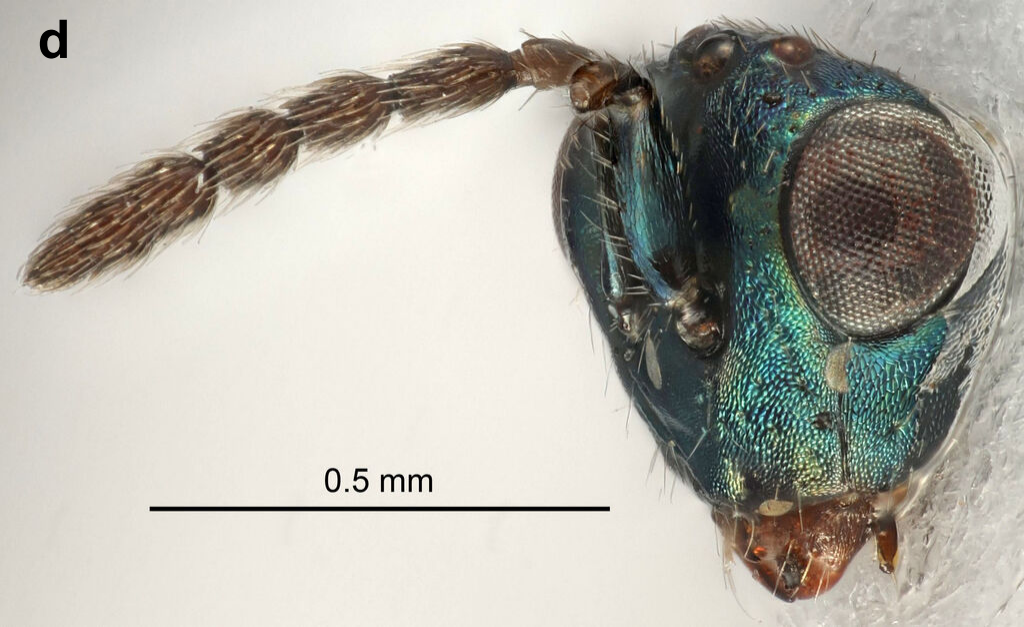
Holotype female, head and antenna lateral.

**Figure 35a.**
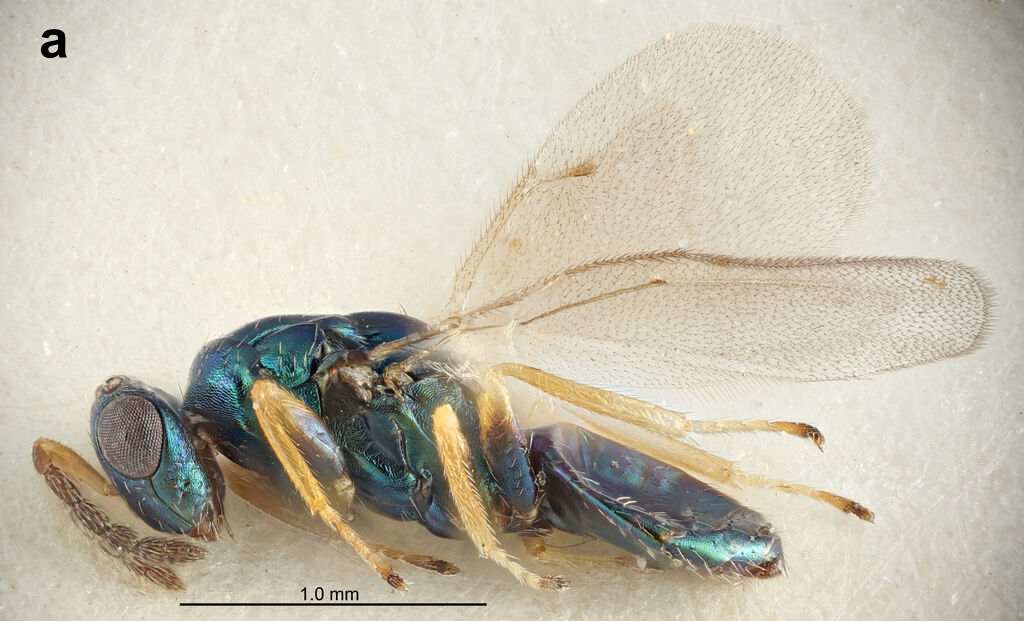
Holotype female, lateral.

**Figure 35b.**
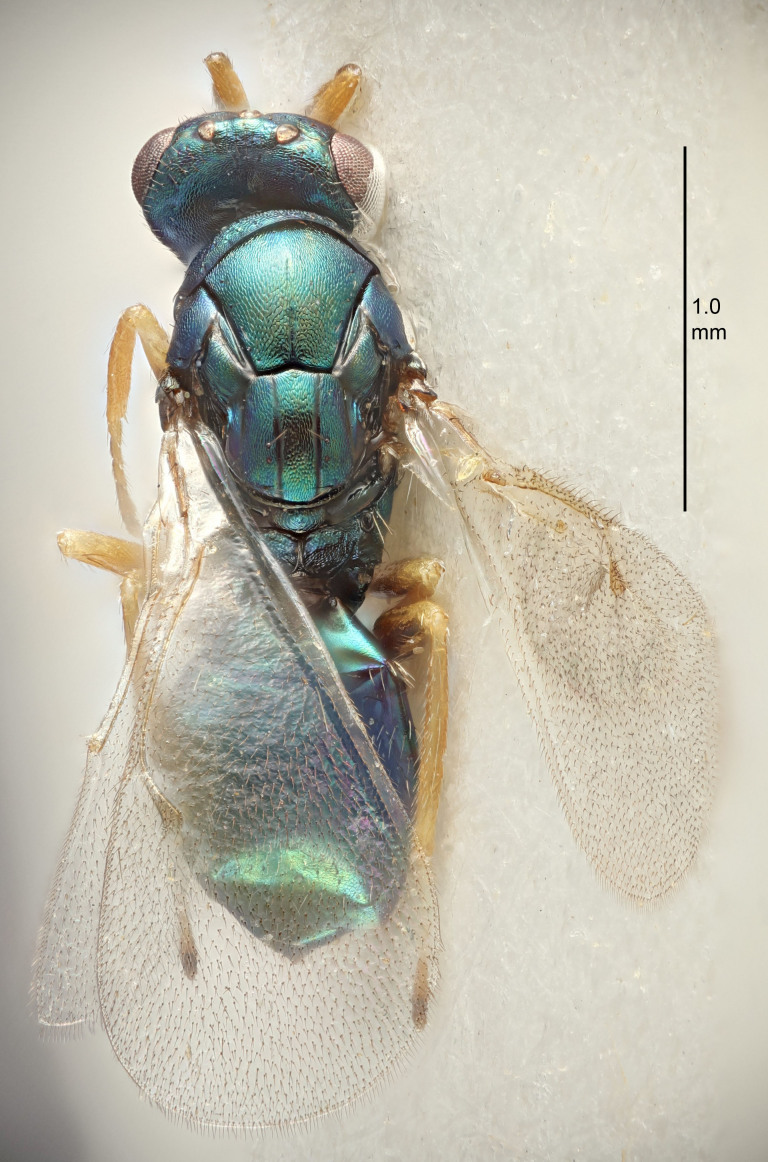
Holotype female, dorsal.

**Figure 36a.**
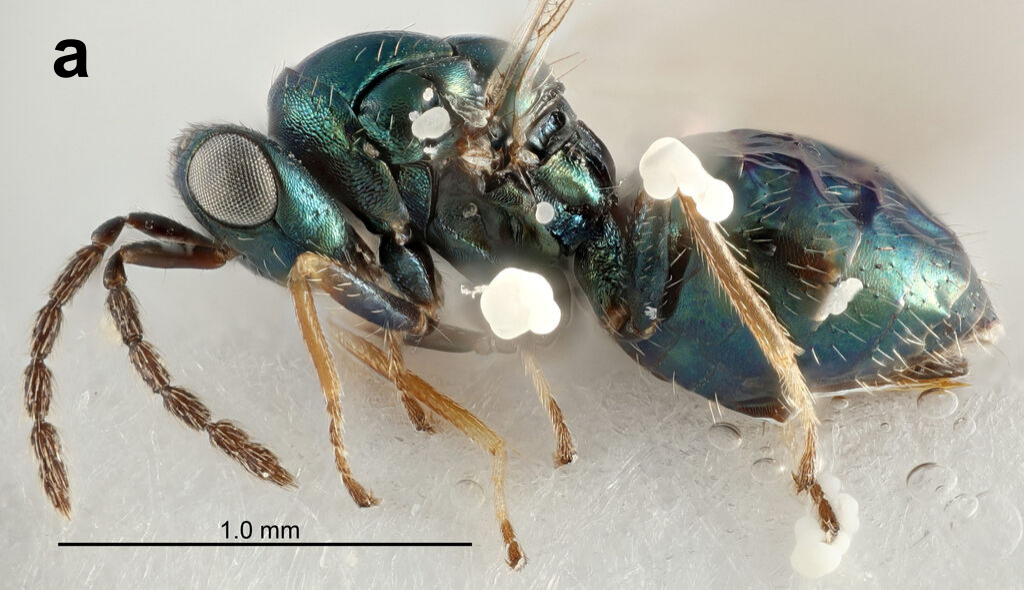
Holotype female, lateral.

**Figure 36b.**
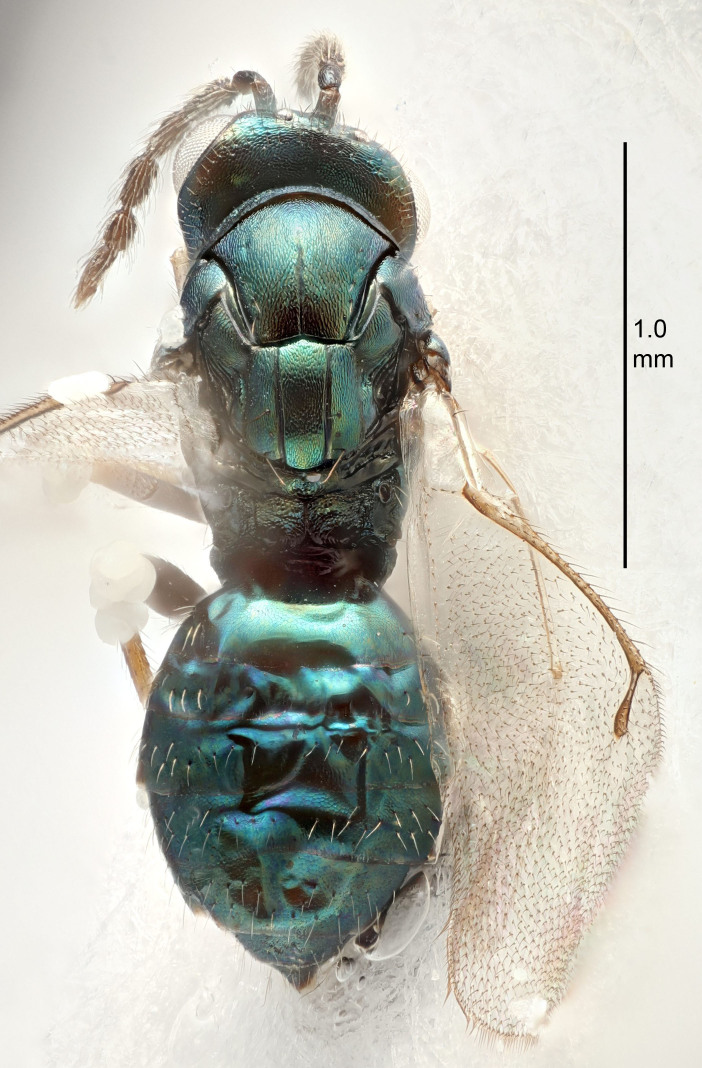
Holotype female, dorsal.

**Figure 36c.**
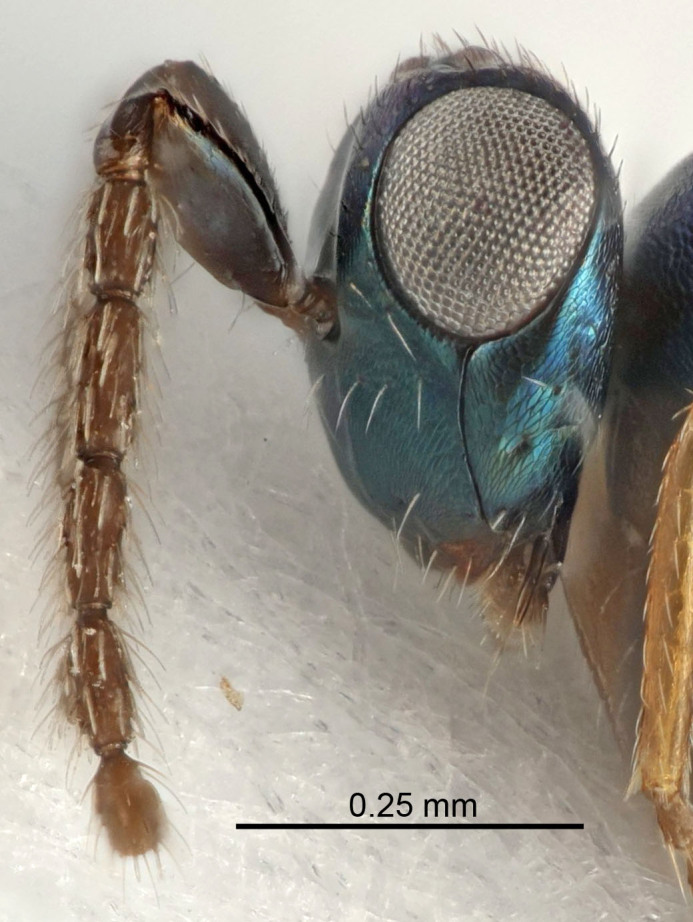
Paratype male, head and antenna lateral.

**Figure 37a.**
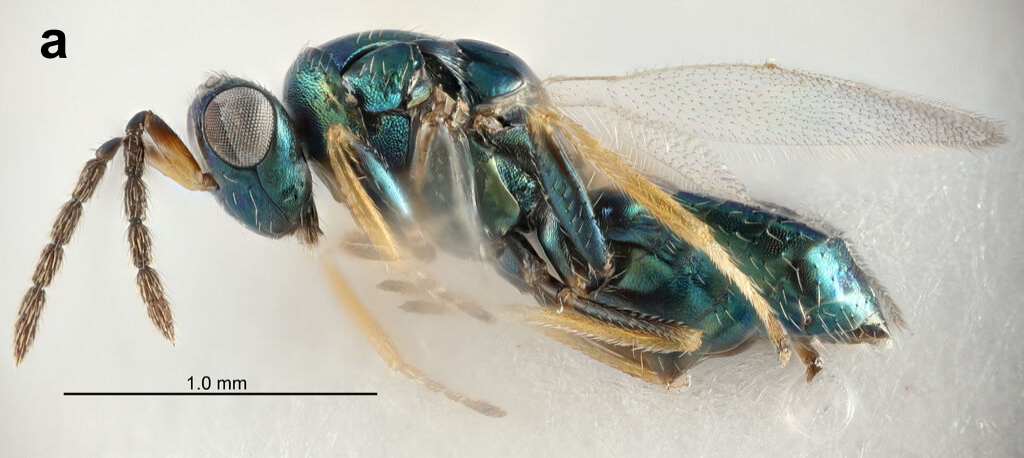
Holotype female, lateral.

**Figure 37b.**
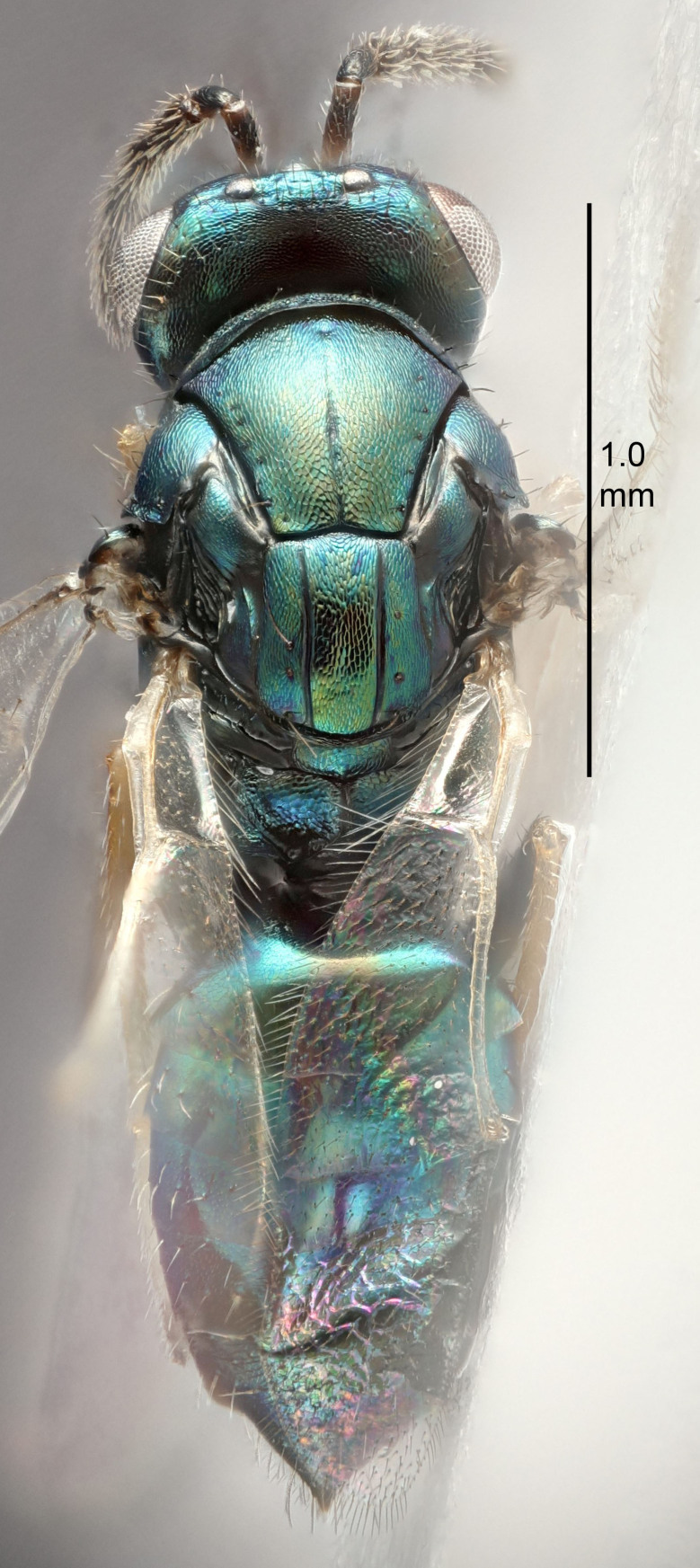
Holotype female, dorsal.

**Figure 37c.**
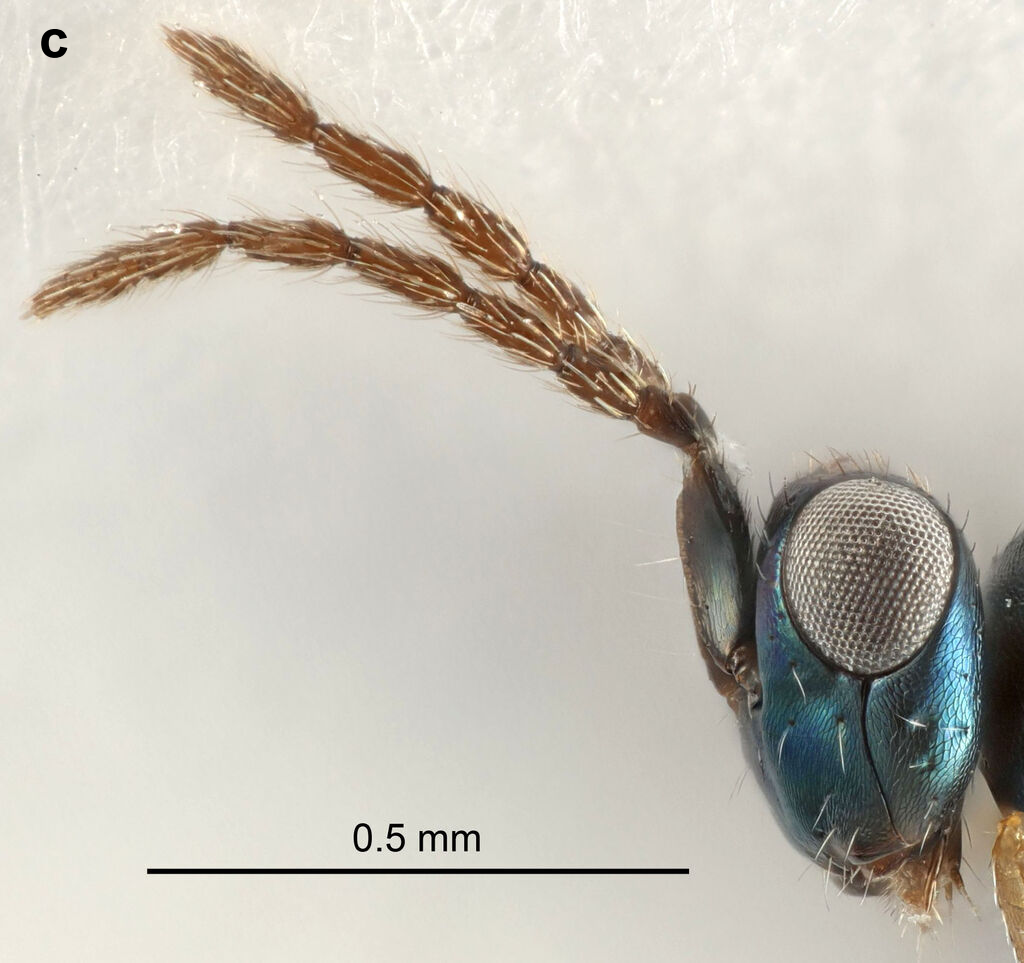
Paratype male, head and antenna lateral.

**Figure 38a.**
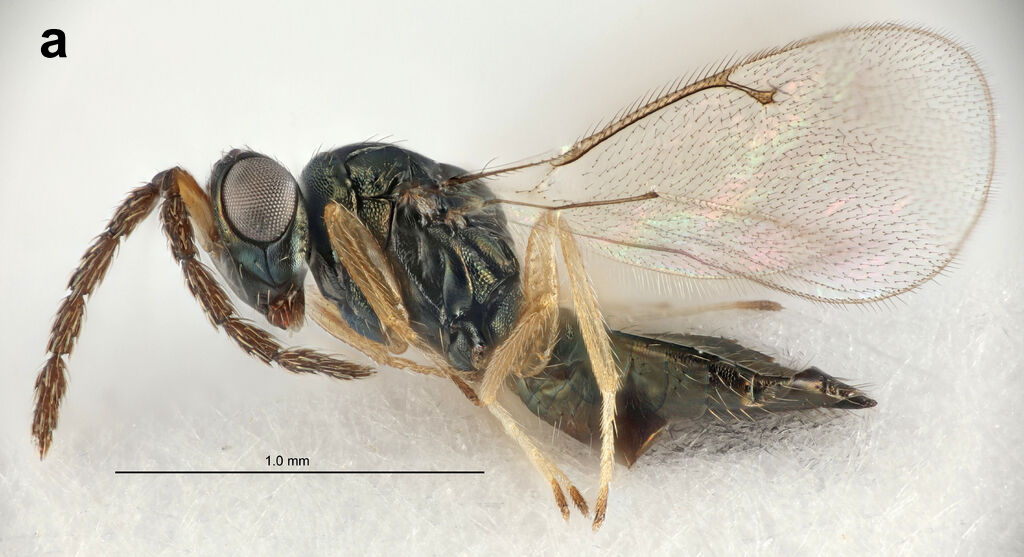
Holotype female, lateral.

**Figure 38b.**
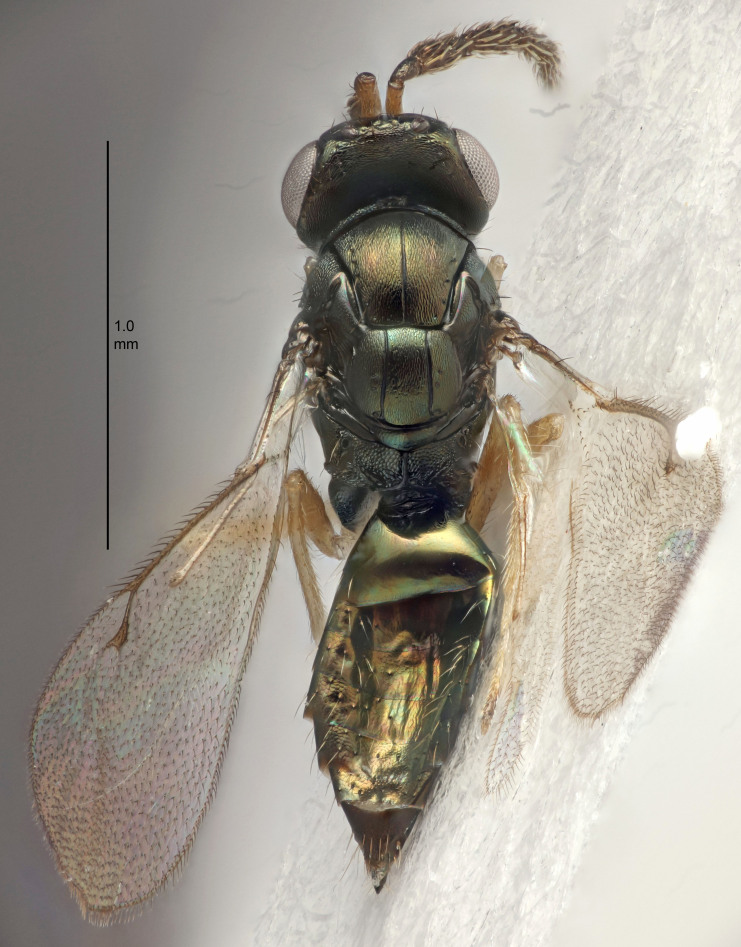
Holotype female, dorsal.

**Figure 38c.**
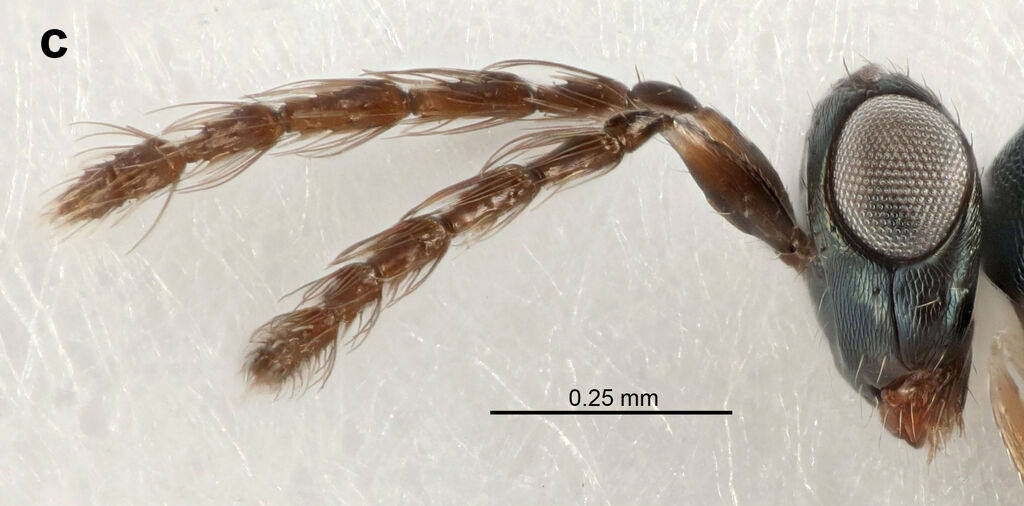
Paratype male, head & antenna lateral.

**Figure 39a.**
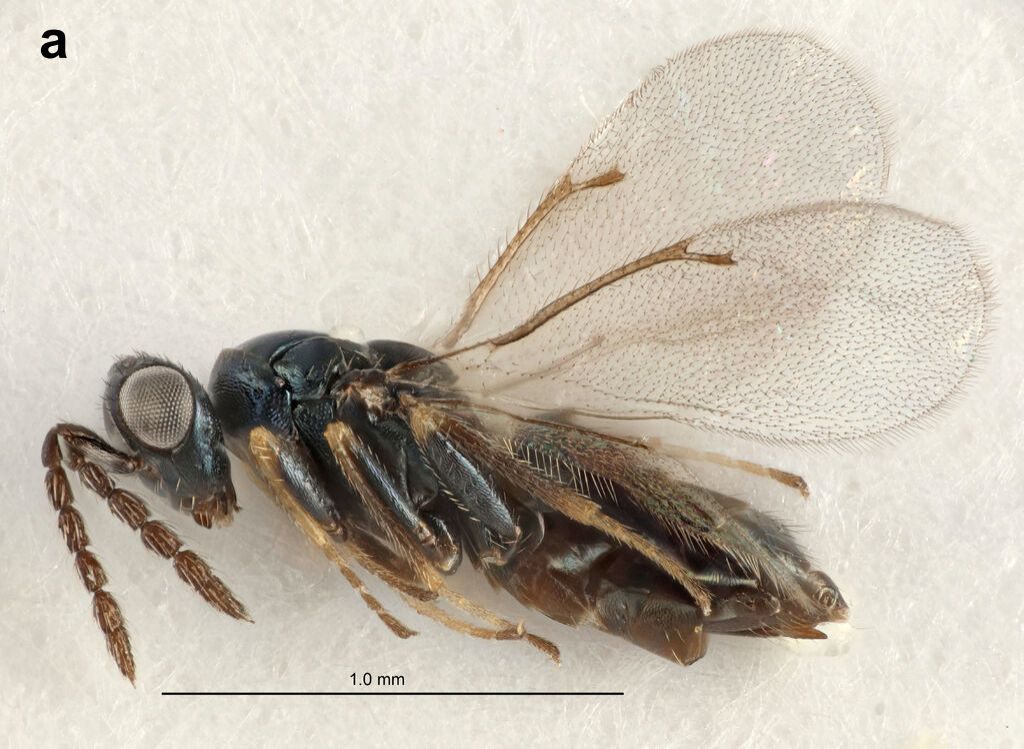
Holotype female, lateral.

**Figure 39b.**
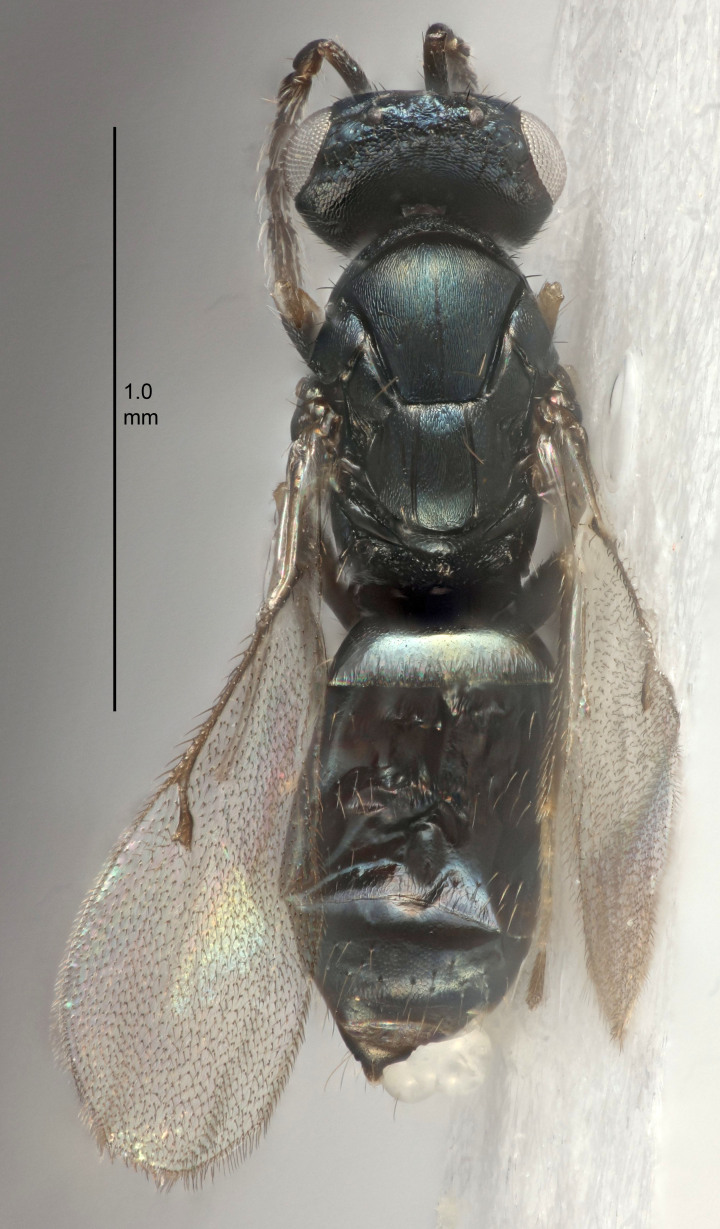
Holotype female, dorsal.

**Figure 39c.**
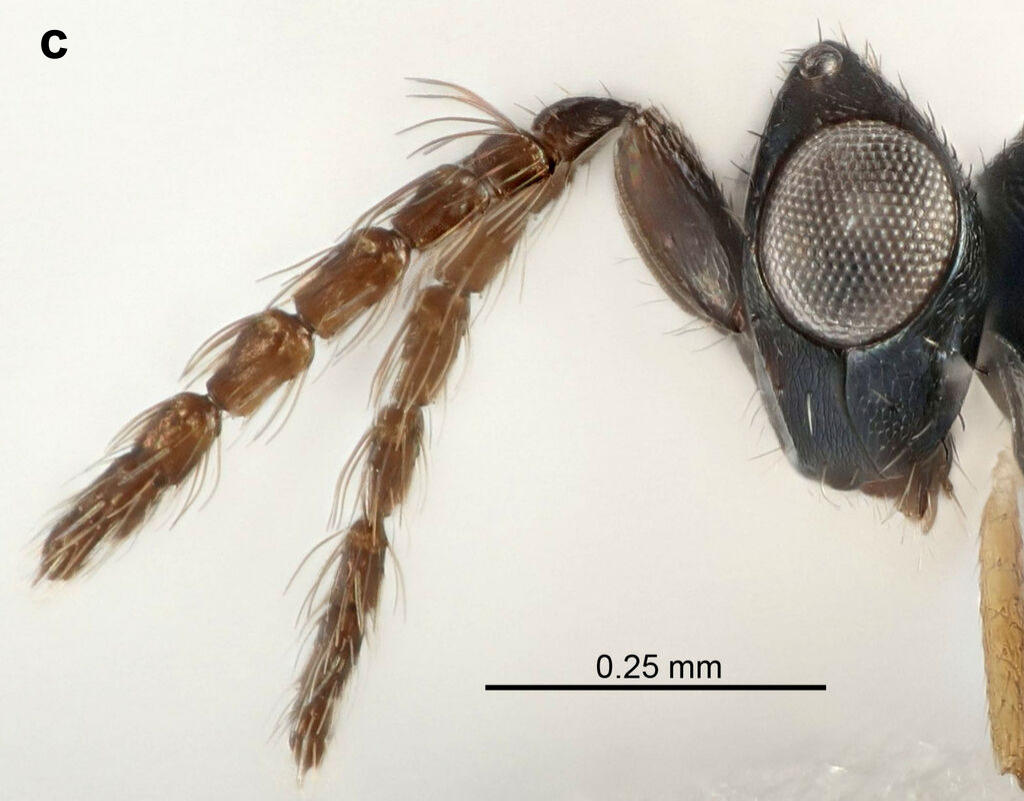
Paratype male, head and antenna lateral.

**Figure 40a.**
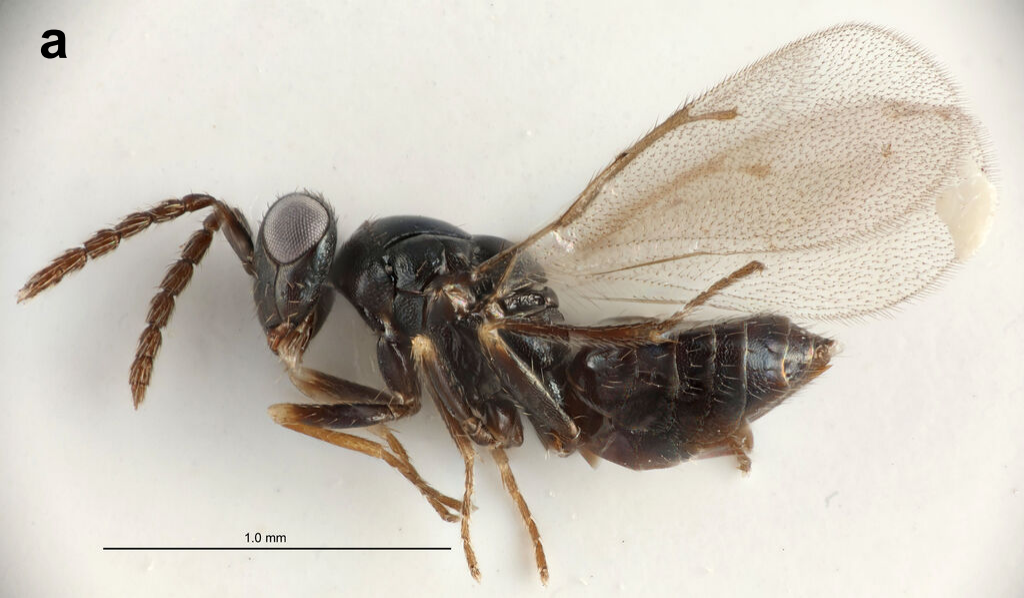
Holotype female, lateral.

**Figure 40b.**
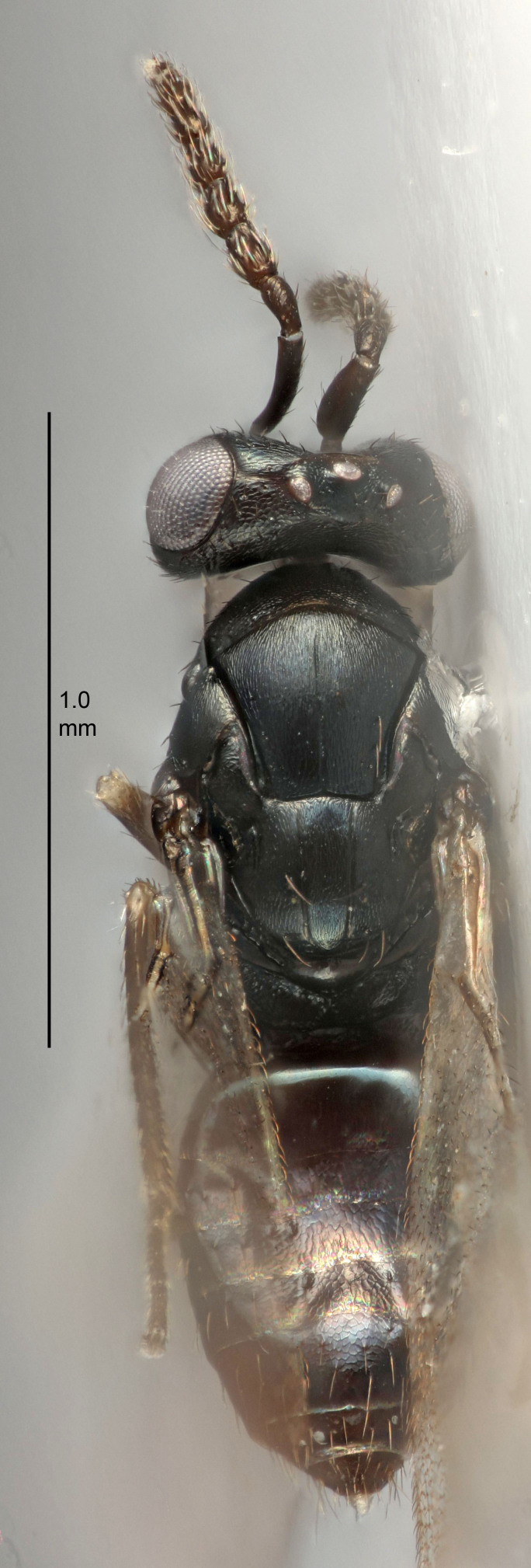
Holotype female, dorsal.

**Figure 41a.**
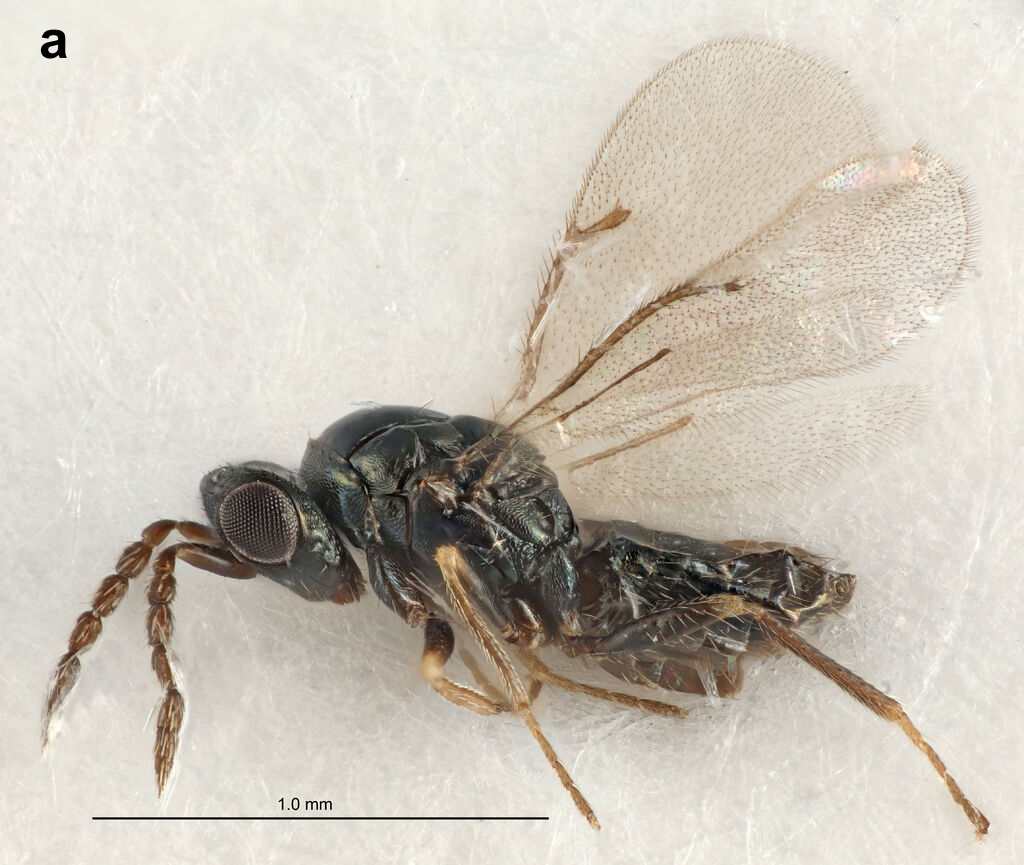
Holotype female, lateral.

**Figure 41b.**
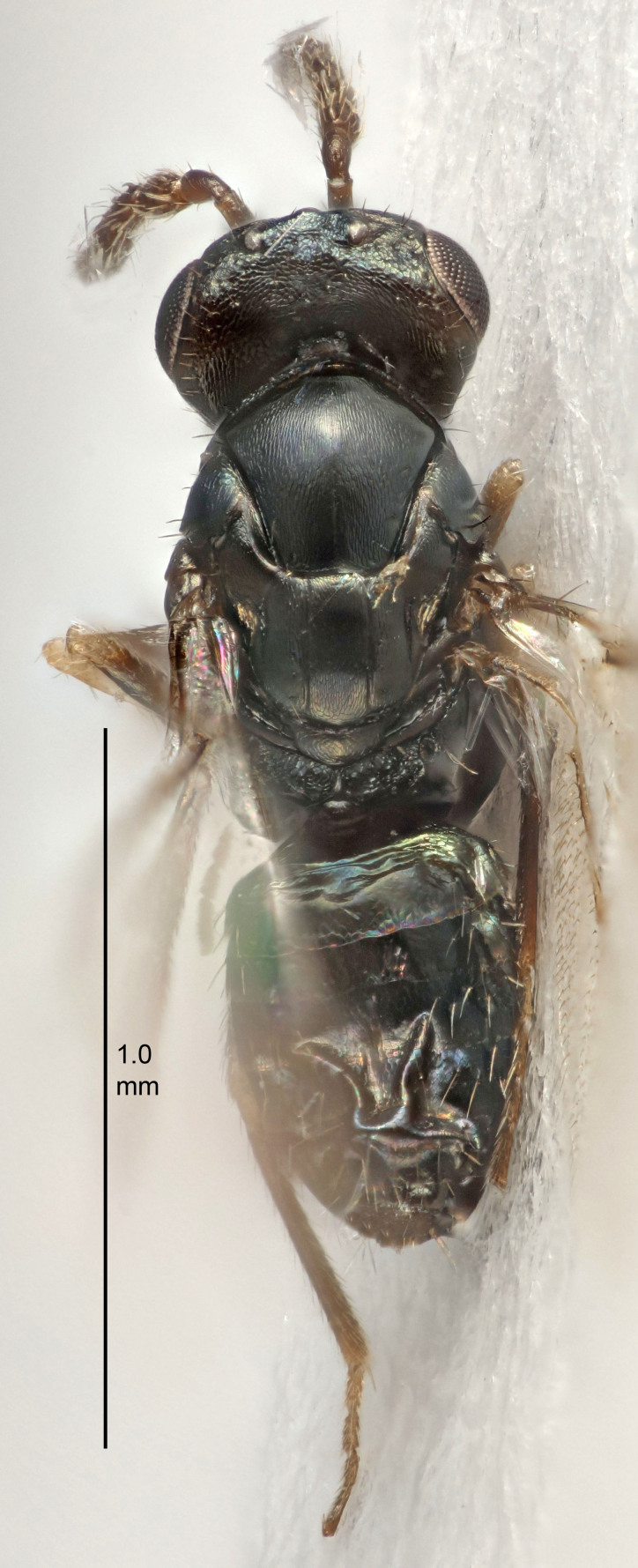
Holotype female, dorsal.

**Figure 41c.**
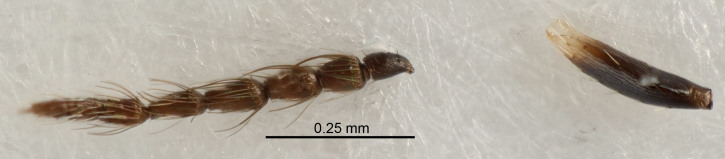
Paratype male, antenna.

**Figure 41d.**
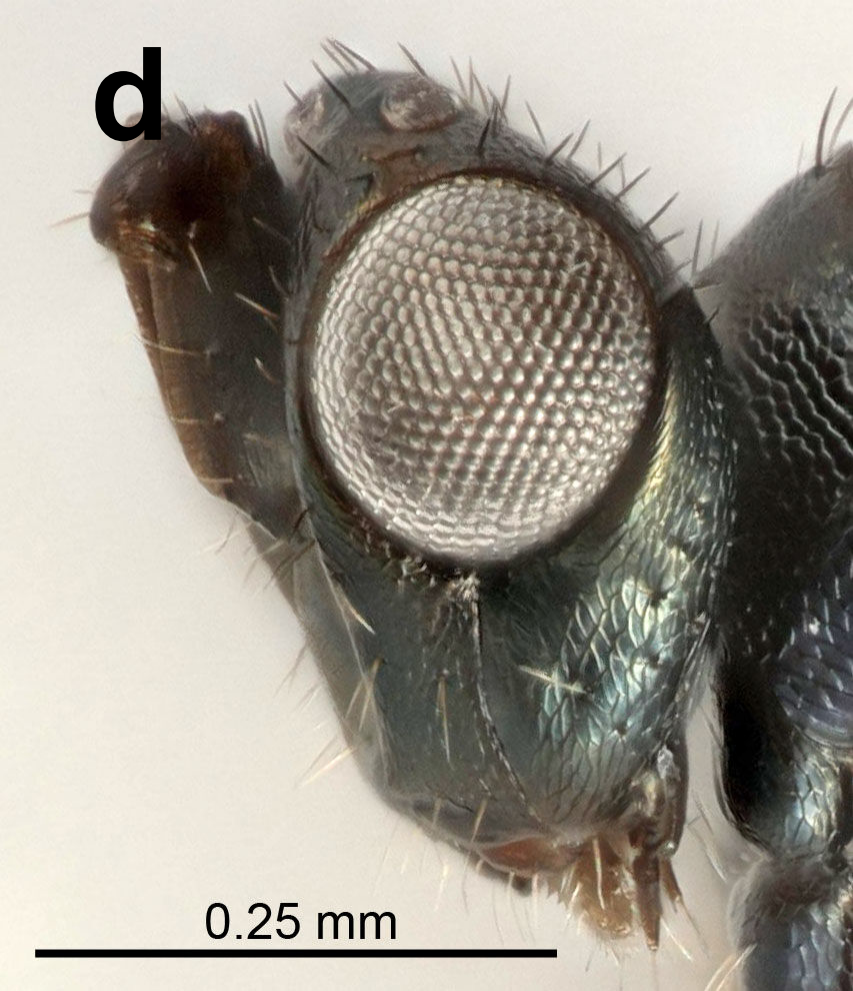
Paratype male, head lateral.

**Figure 42a.**
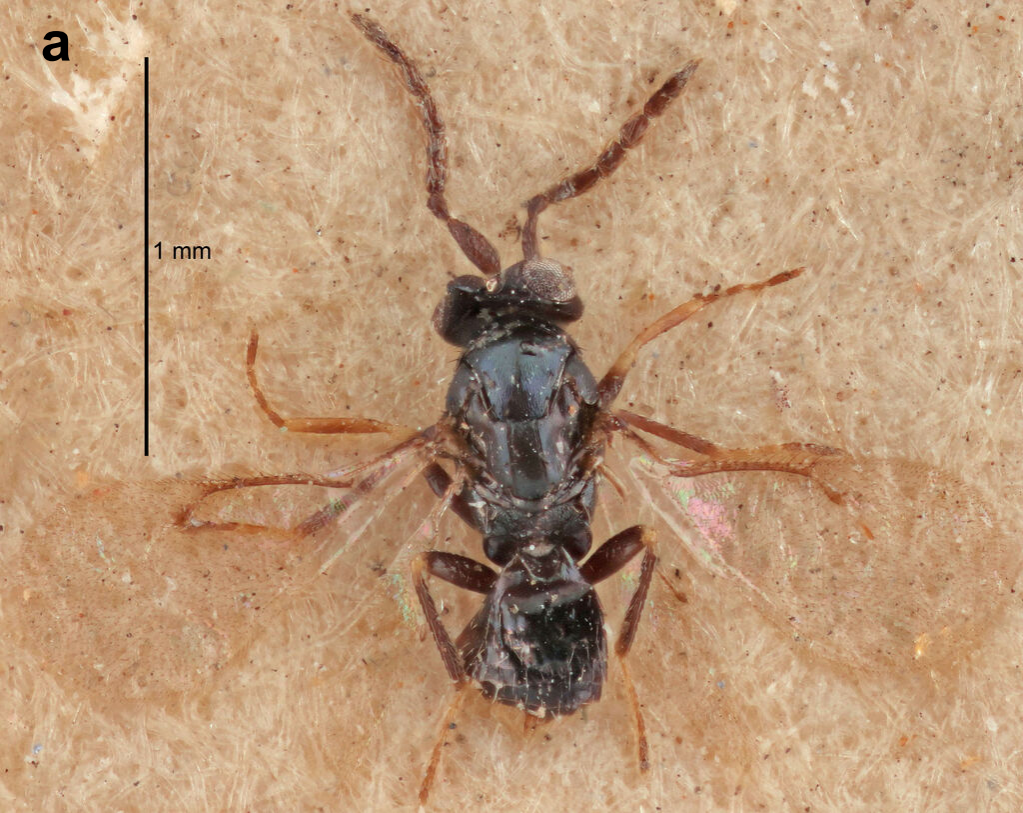
Lectotype male, dorsal.

**Figure 42b.**
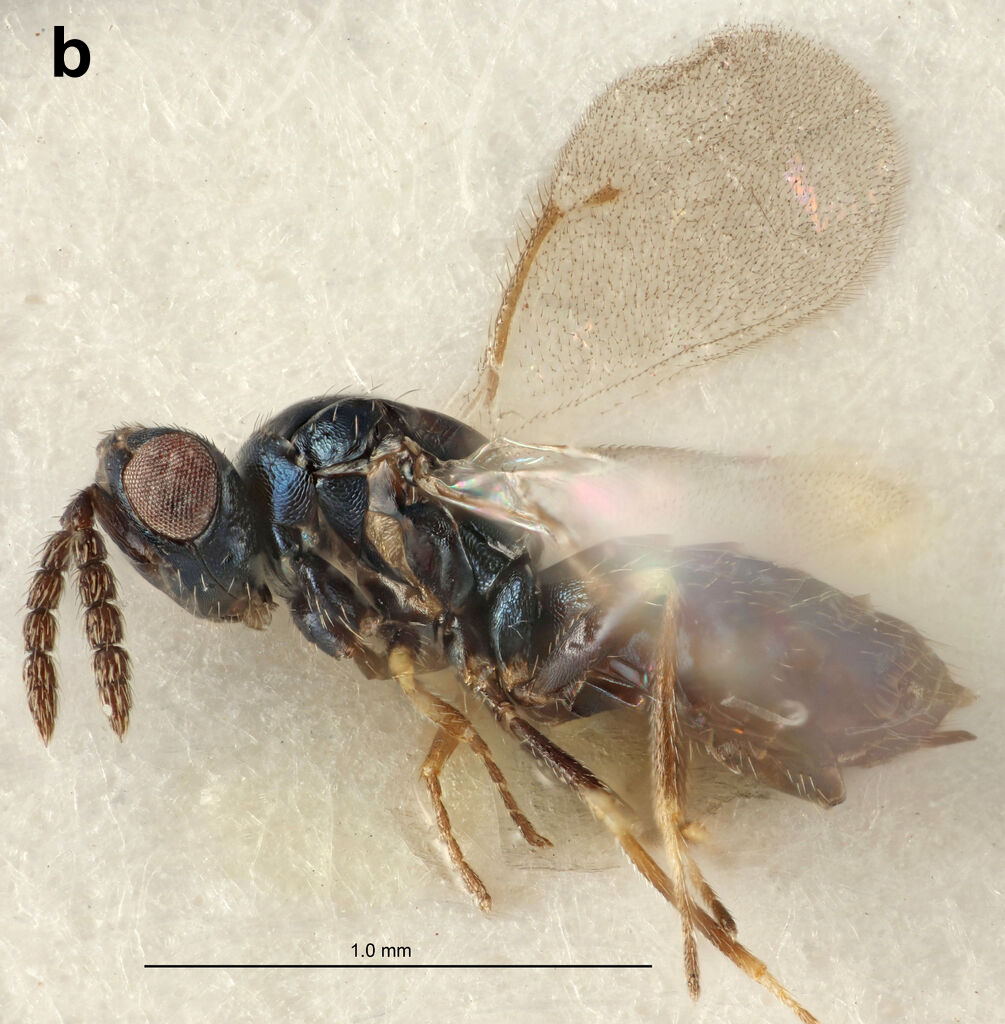
Nontype female, lateral.

**Figure 42c.**
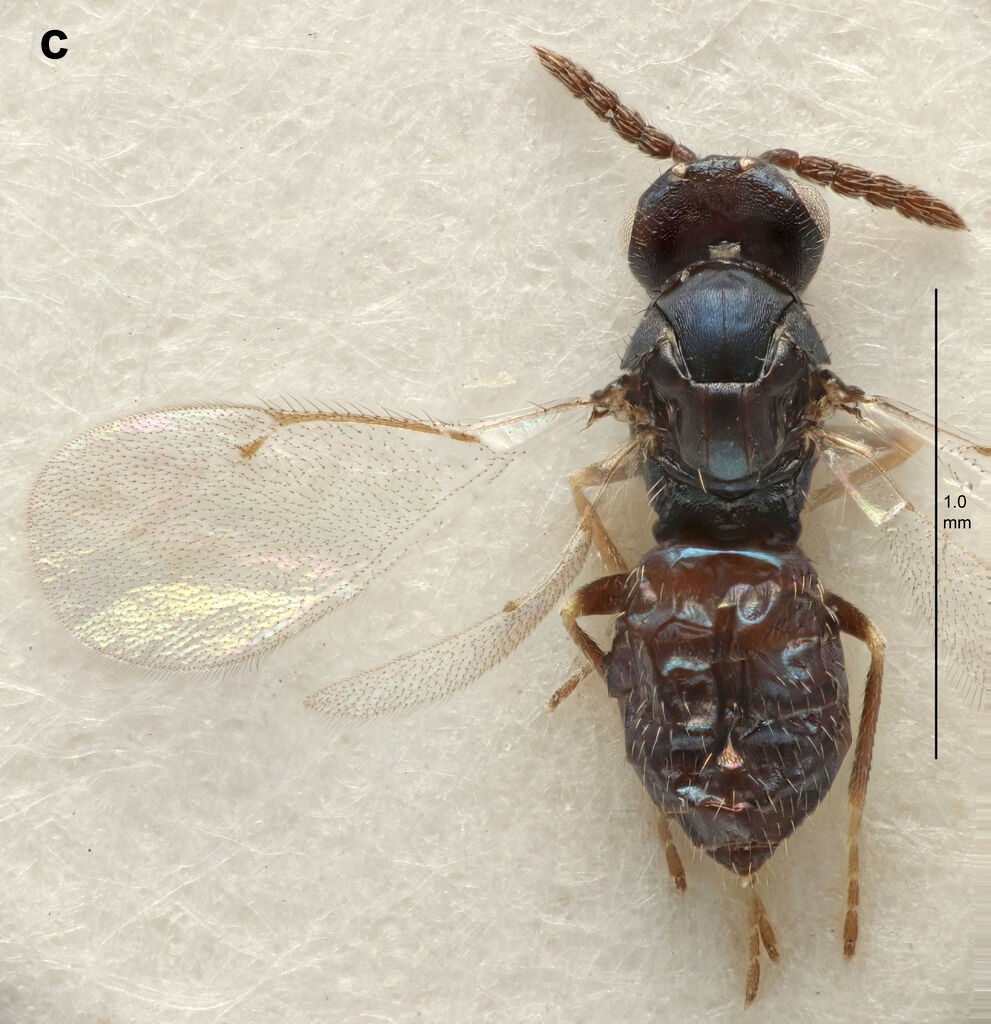
Nontype female, dorsal.

**Figure 42d.**
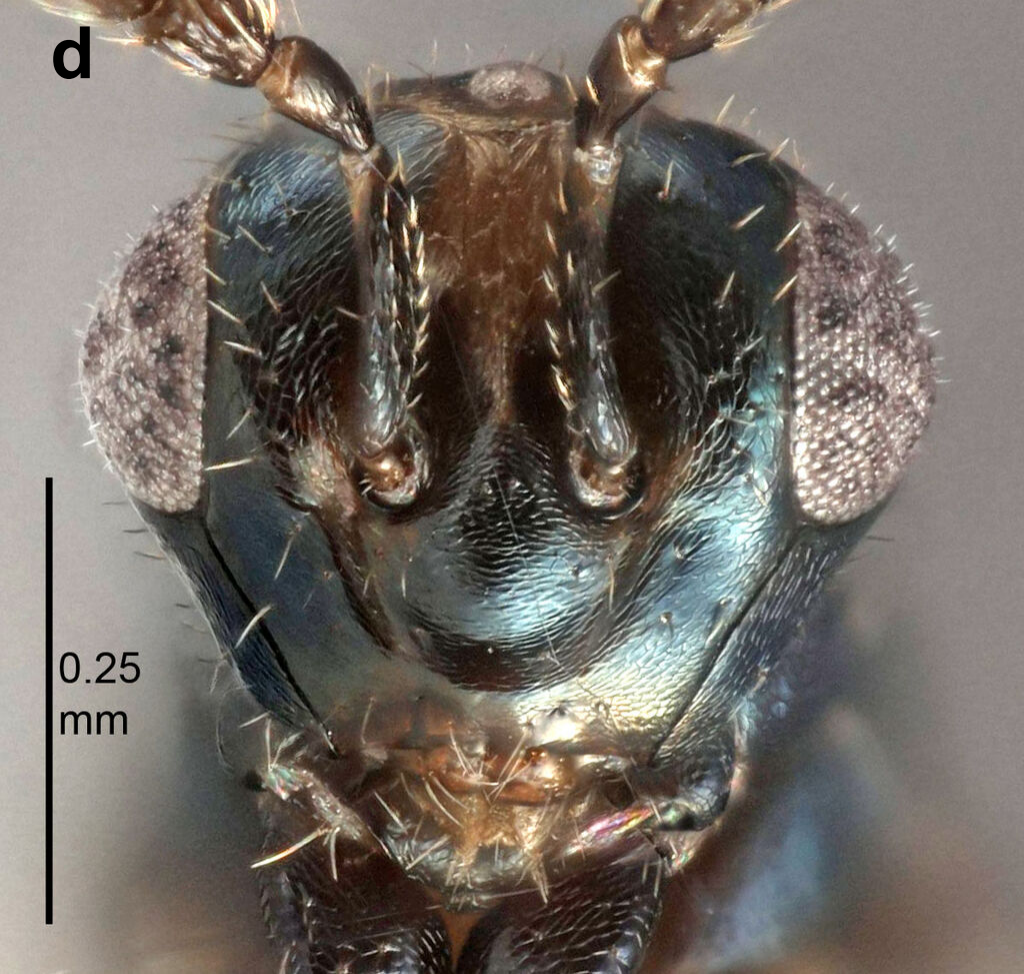
Nontype female, head frontal.

**Figure 42e.**
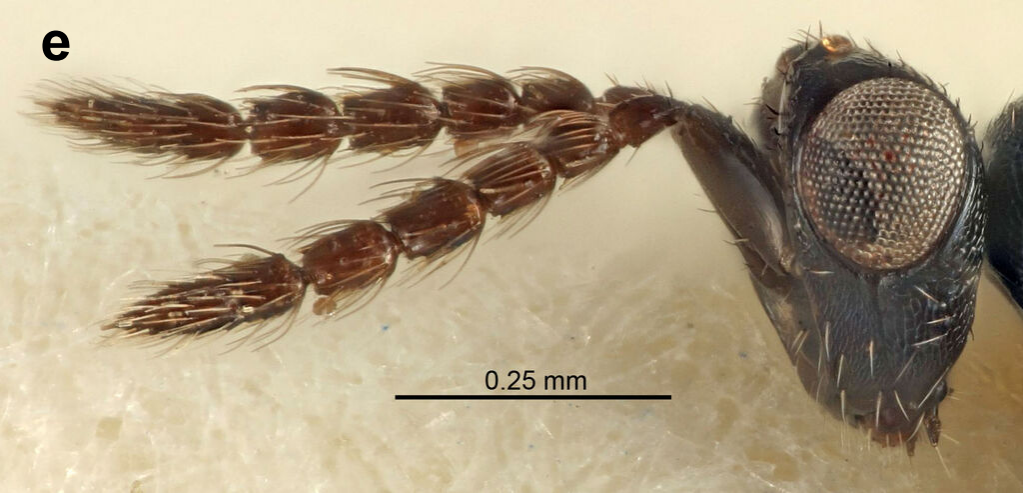
Nontype male, head and antenna, lateral.

**Figure 43a.**
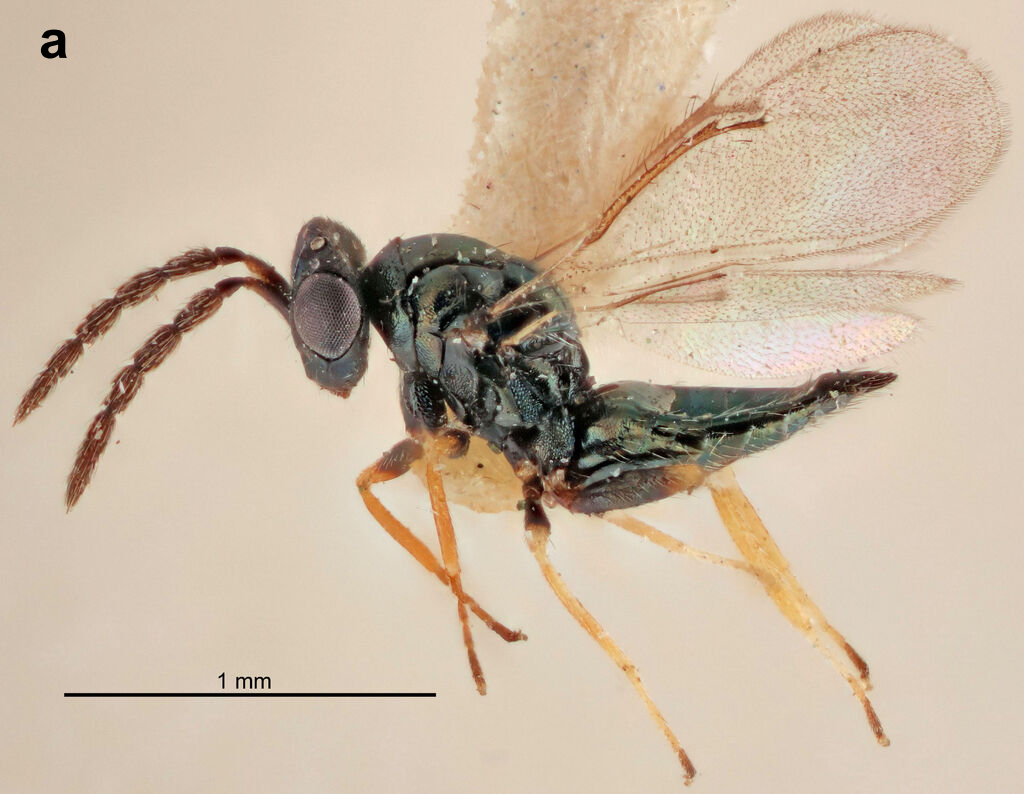
Holotype female, lateral.

**Figure 43b.**
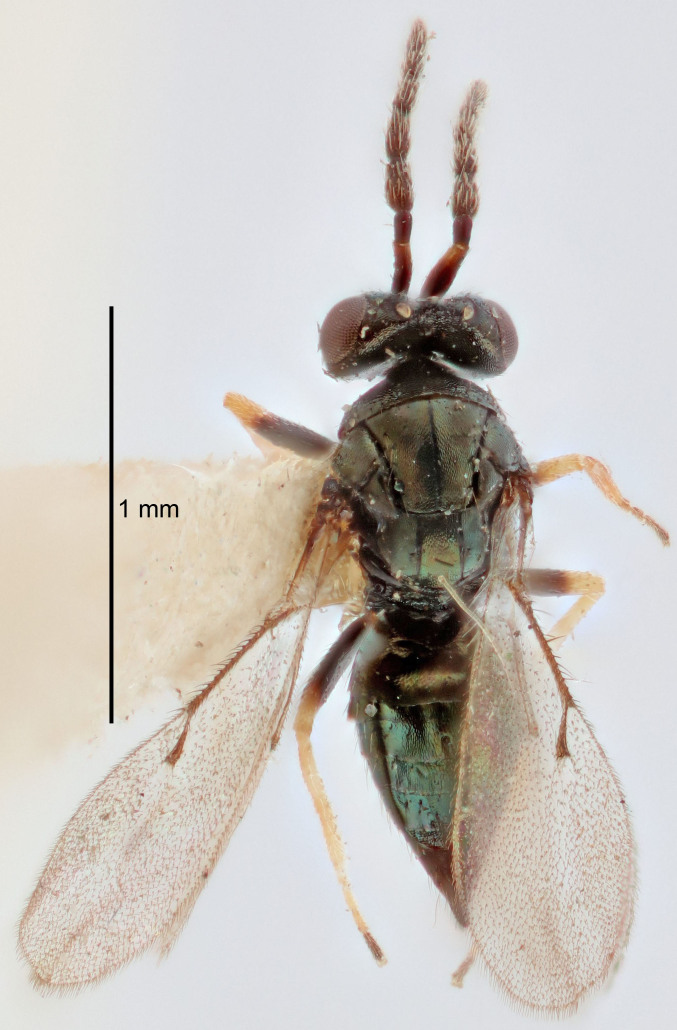
Holotype female, dorsal.

**Figure 44a.**
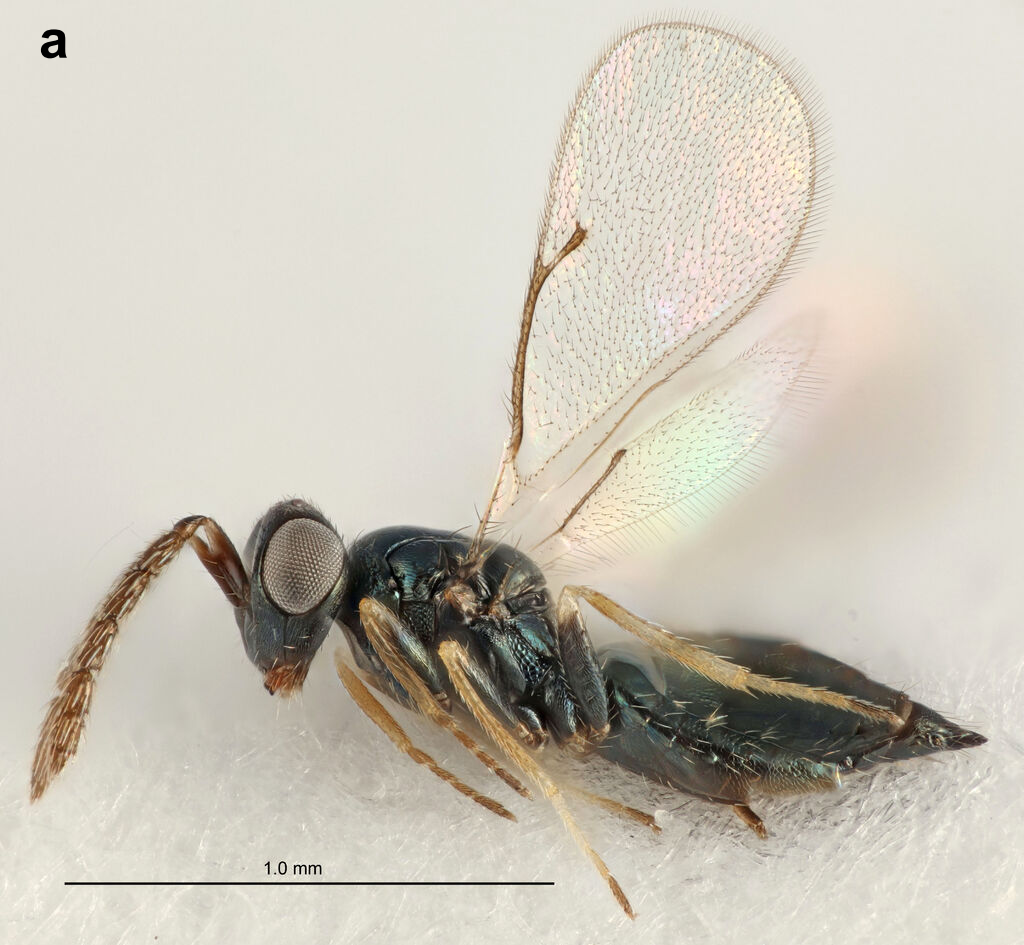
Holotype female, lateral.

**Figure 44b.**
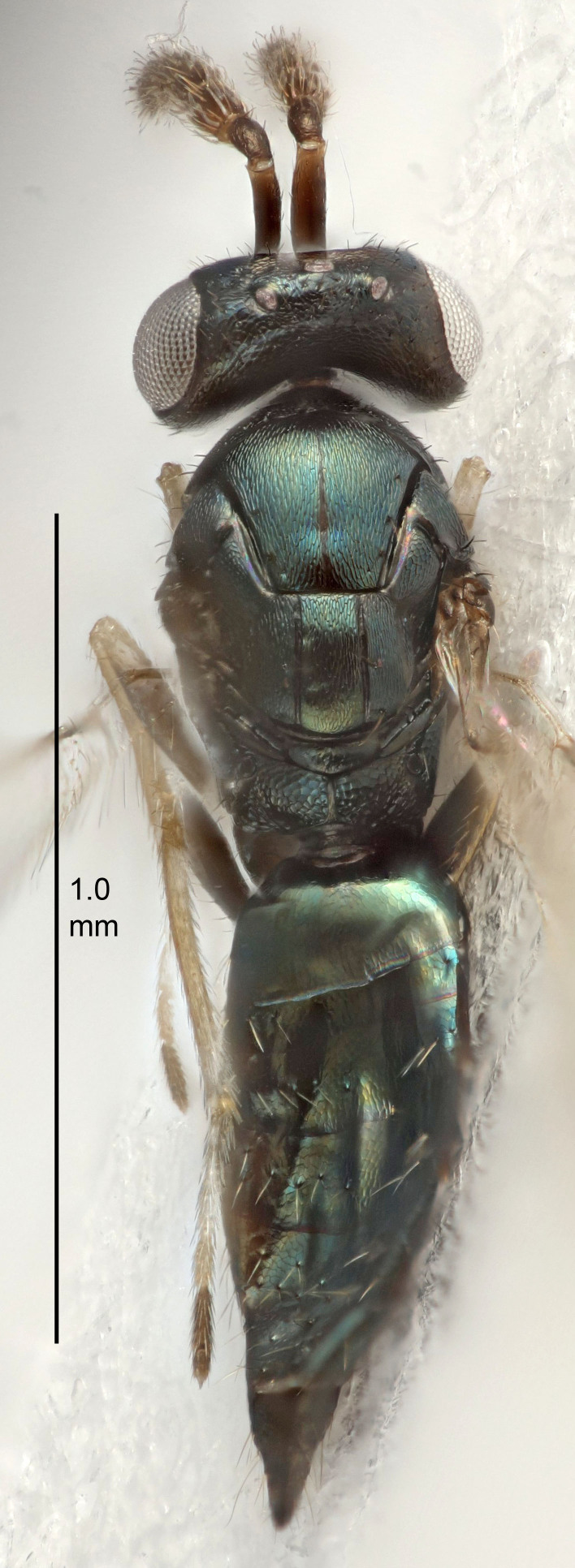
Holotype female, dorsal.

**Figure 45a.**
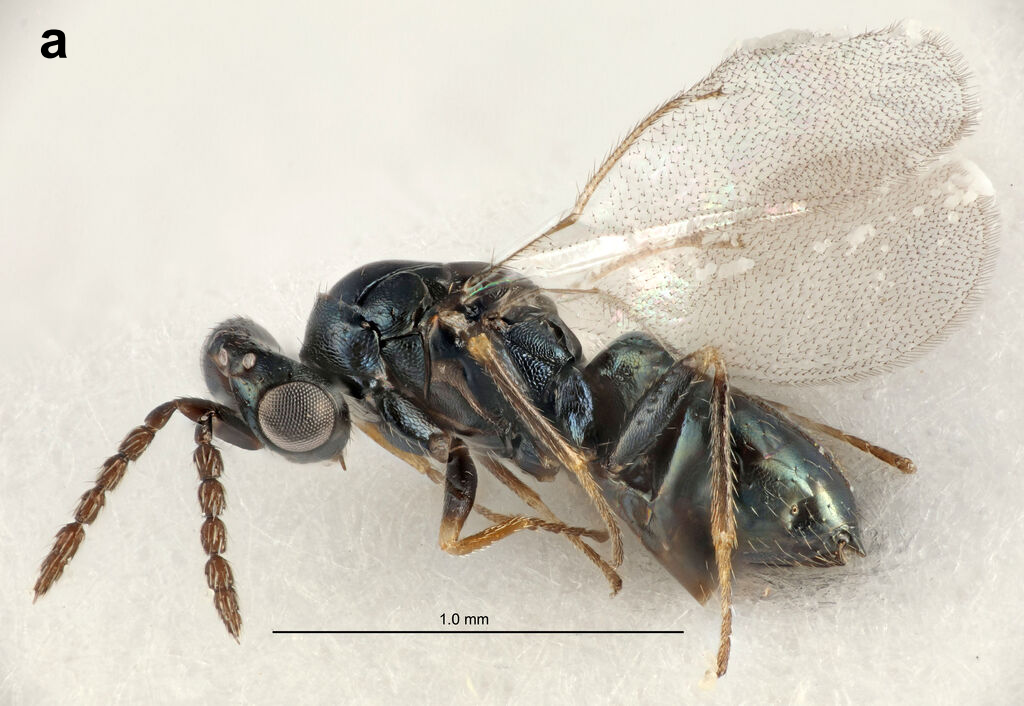
Holotype female, lateral.

**Figure 45b.**
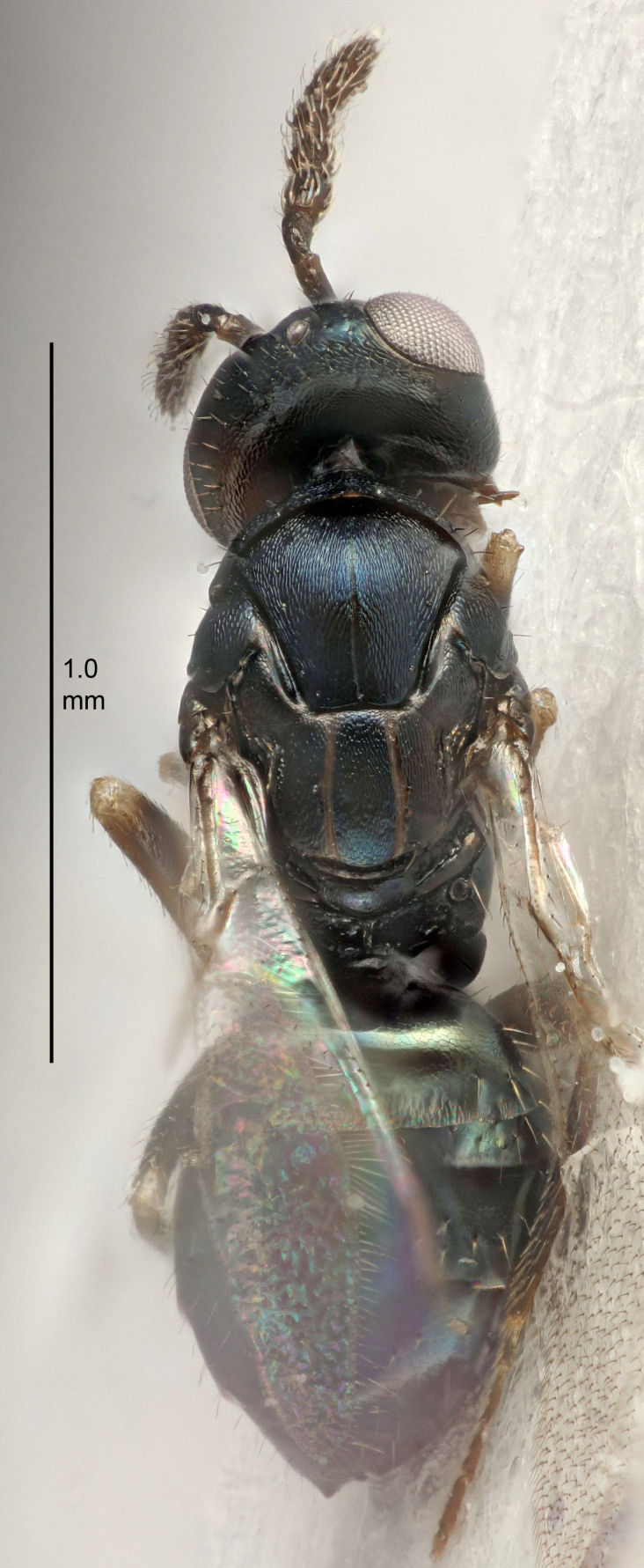
Holotype female, dorsal.

**Figure 46a.**
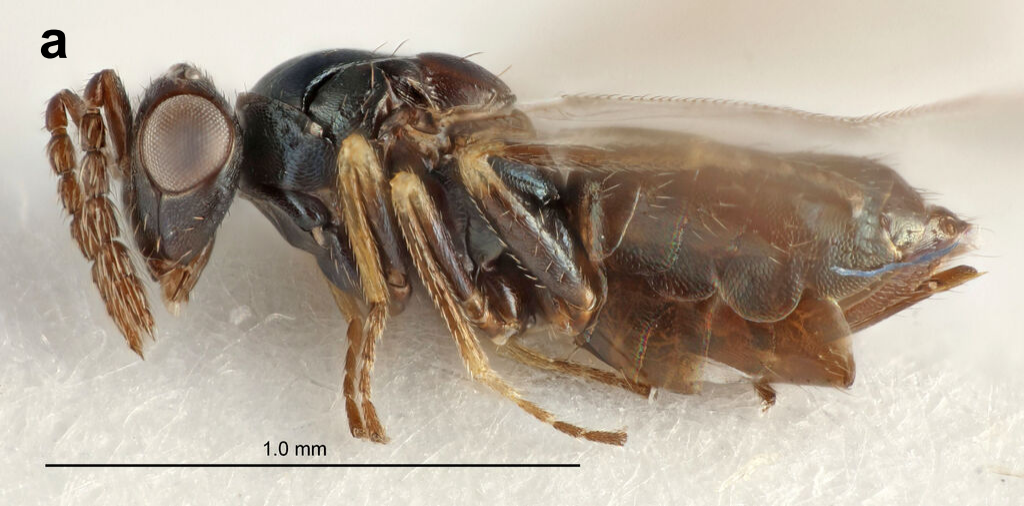
Holotype female, lateral.

**Figure 46b.**
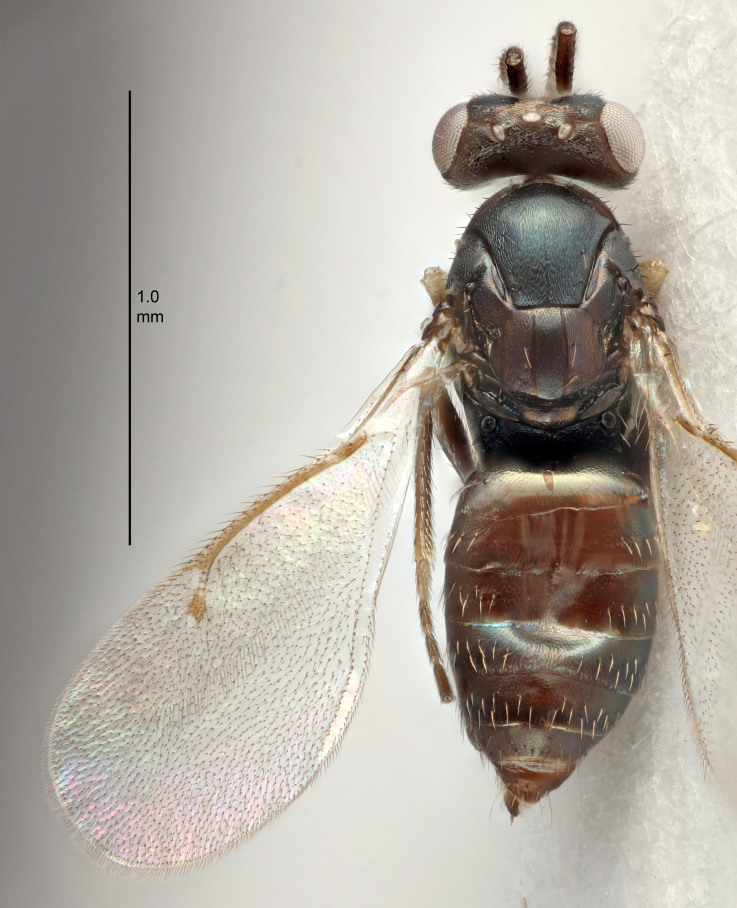
Holotype female, dorsal.

**Figure 47a.**
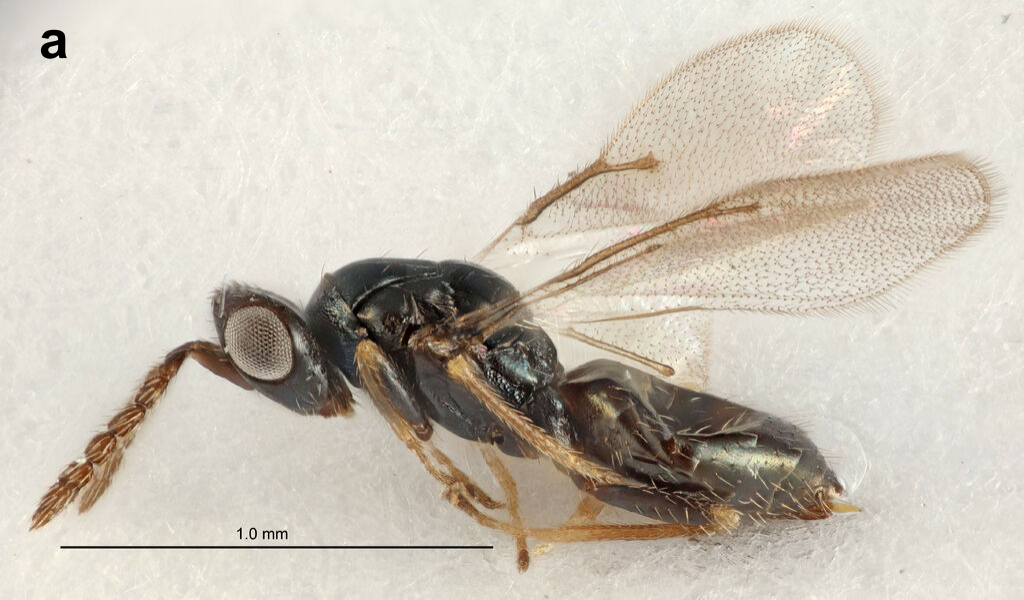
Holotype female, lateral.

**Figure 47b.**
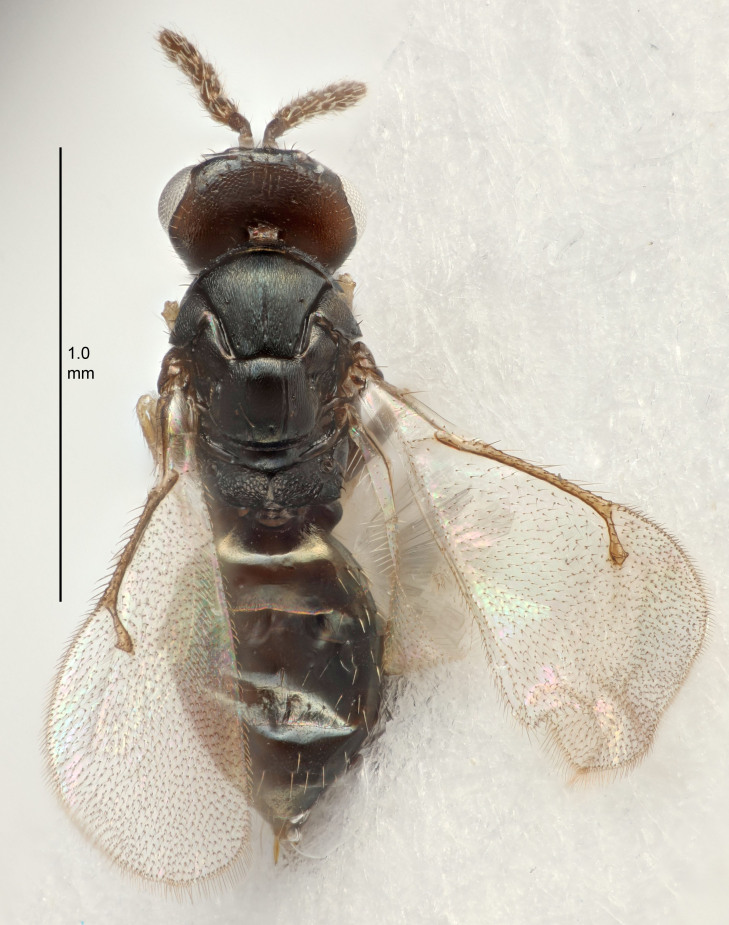
Holotype female, dorsal.

**Figure 48a.**
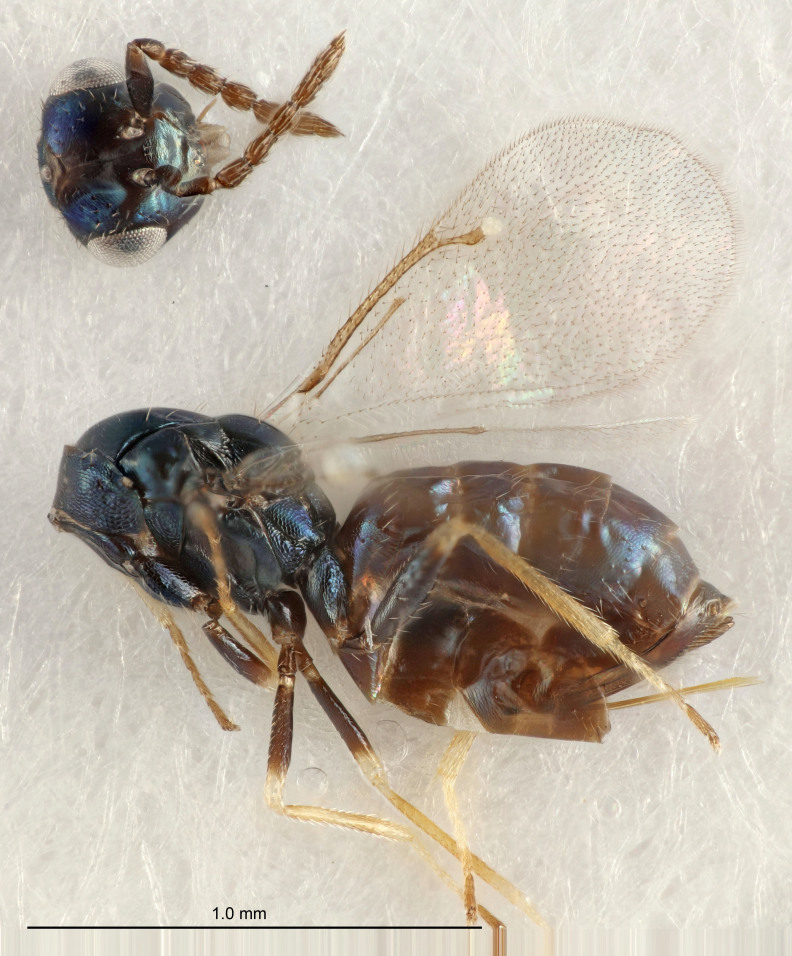
Holotype female, lateral.

**Figure 48b.**
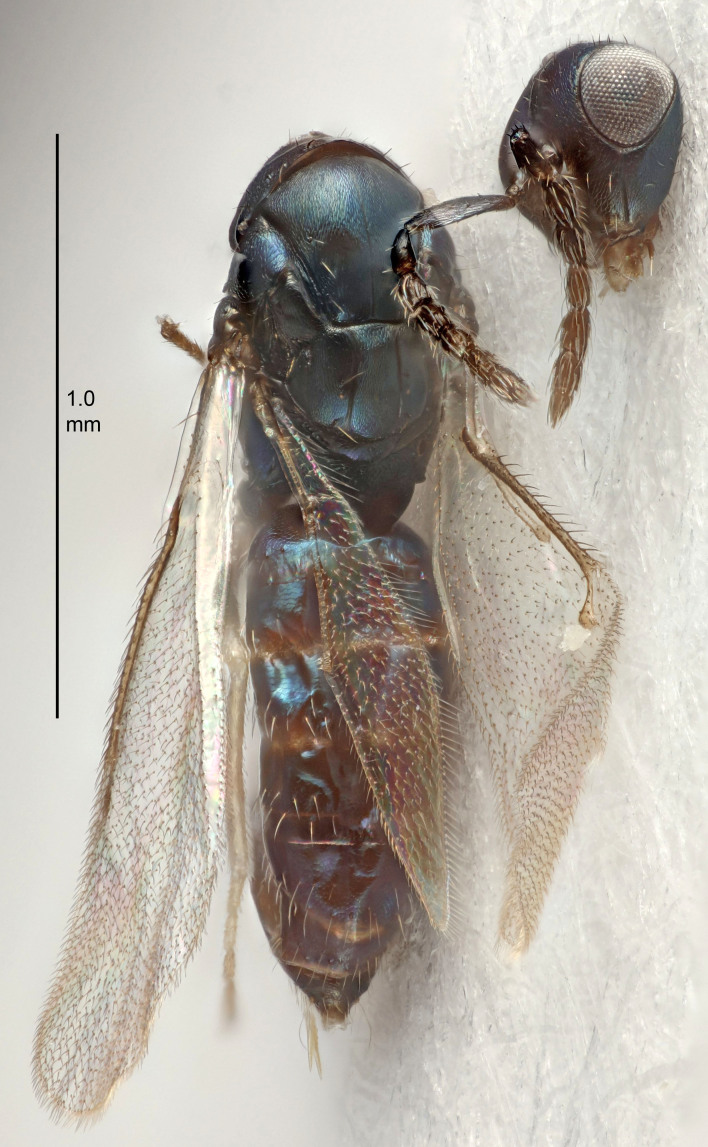
Holotype female, dorsal.

**Figure 49a.**
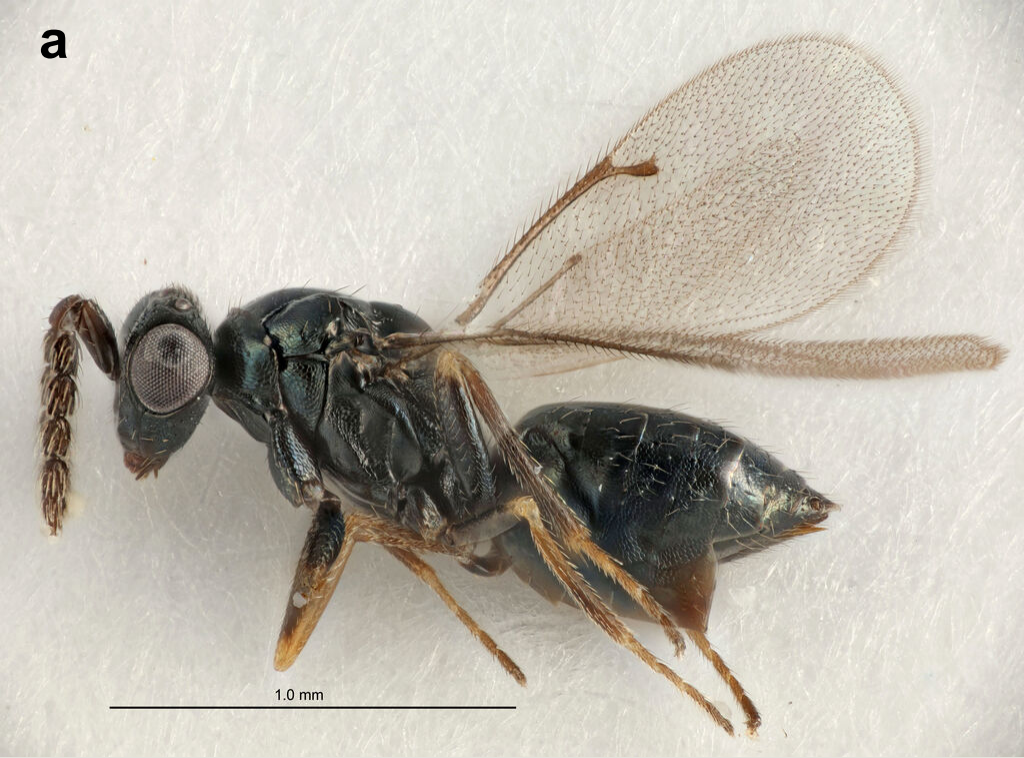
Holotype female, lateral.

**Figure 49b.**
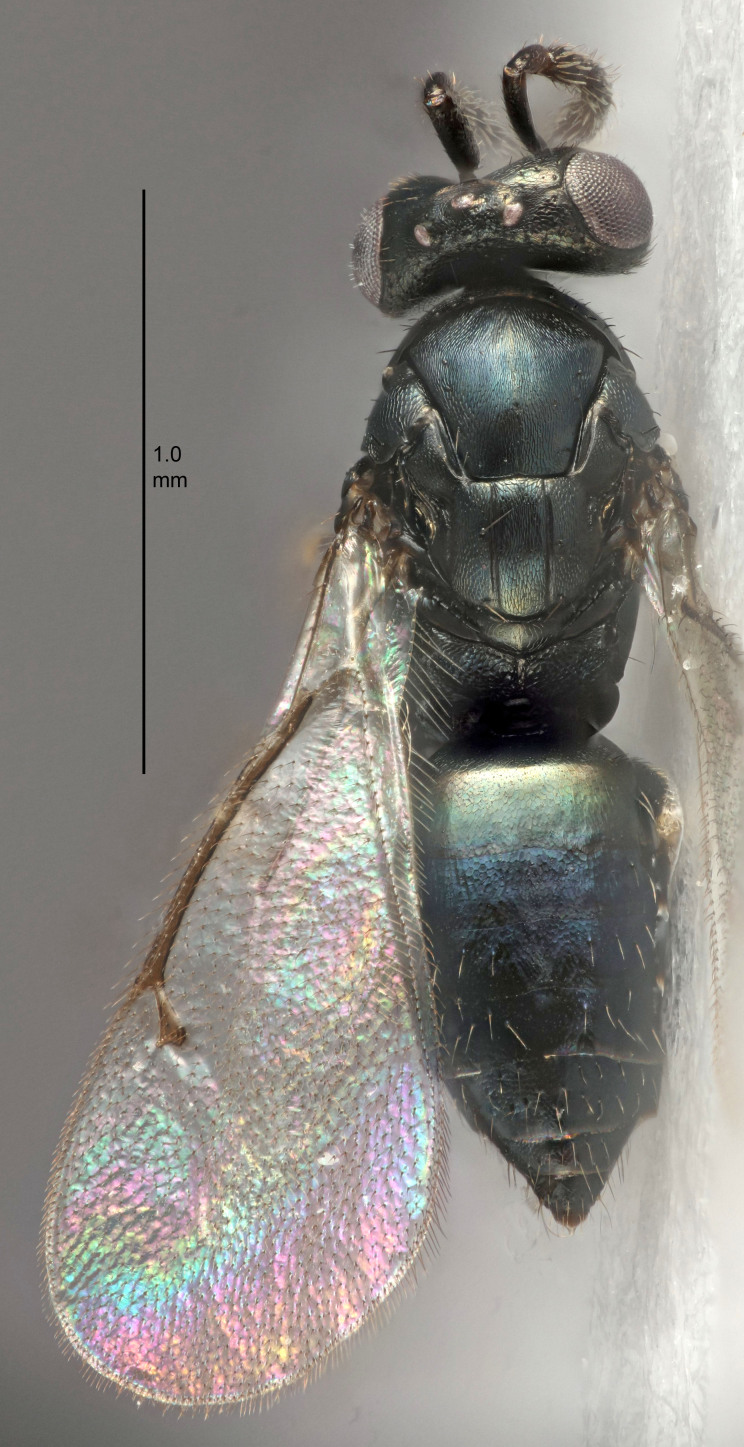
Holotype female, dorsal.

**Figure 49c.**
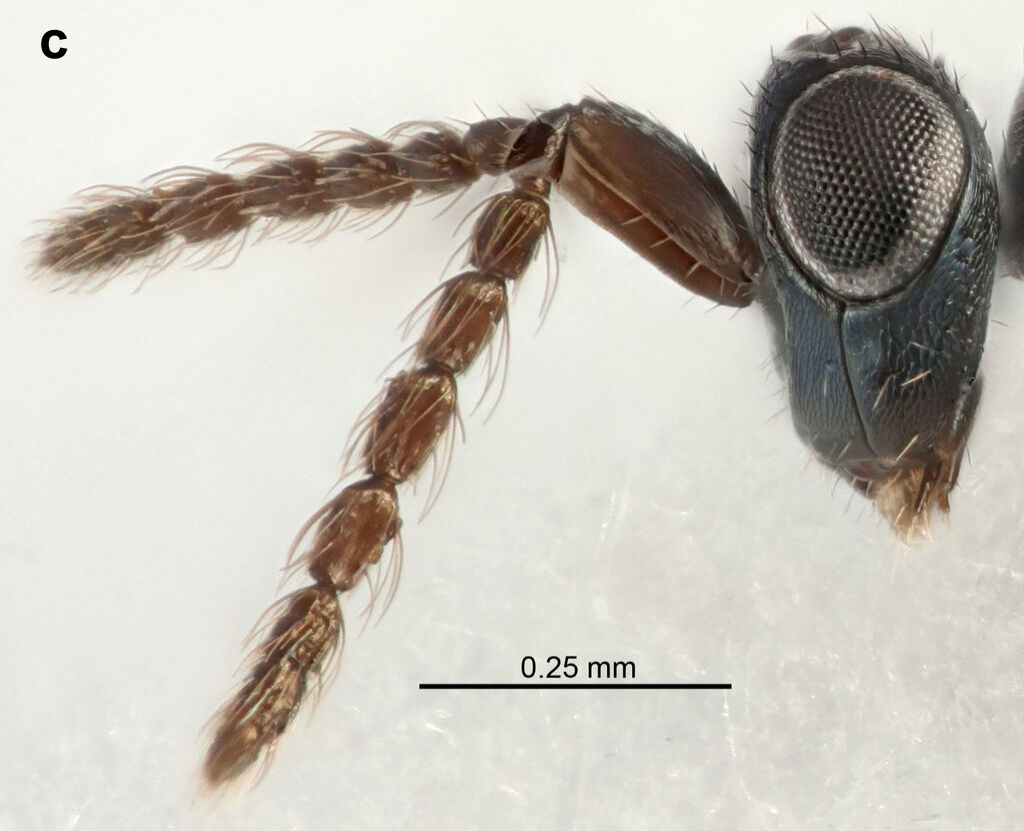
Paratype male, head and antenna lateral.

**Figure 50a.**
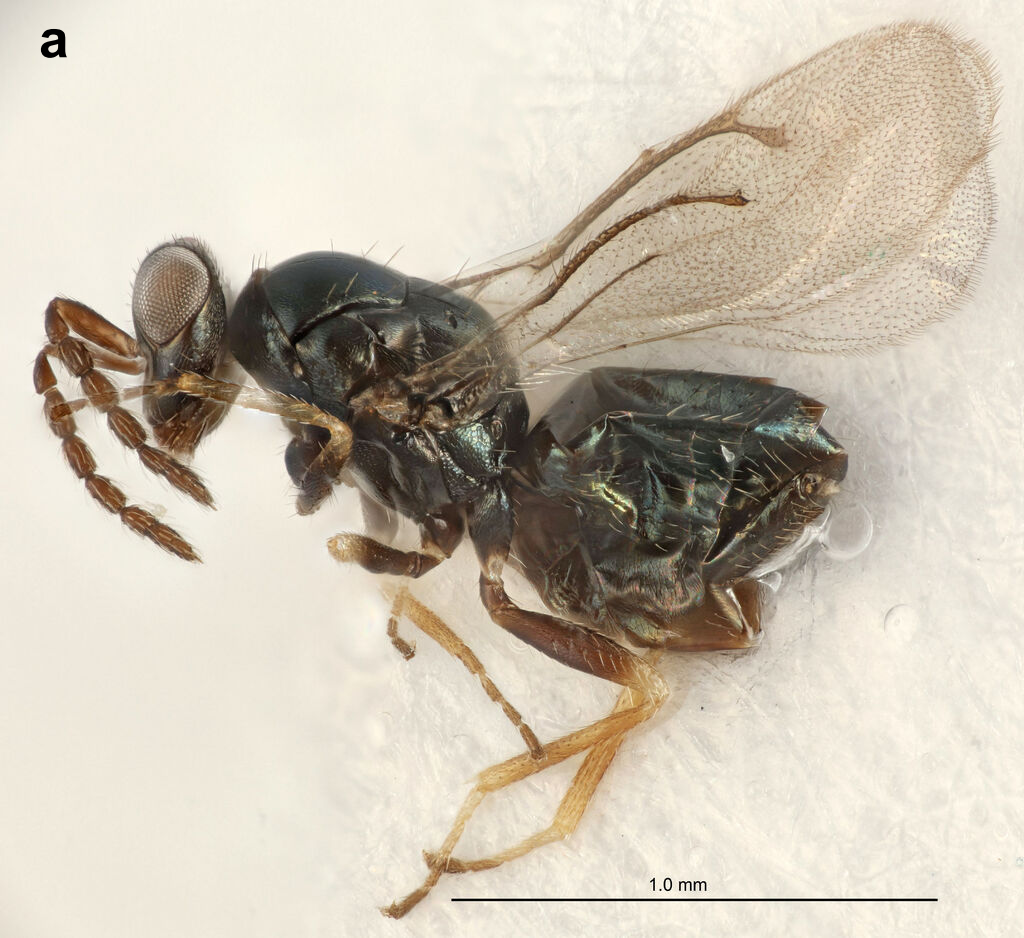
Holotype female, lateral.

**Figure 50b.**
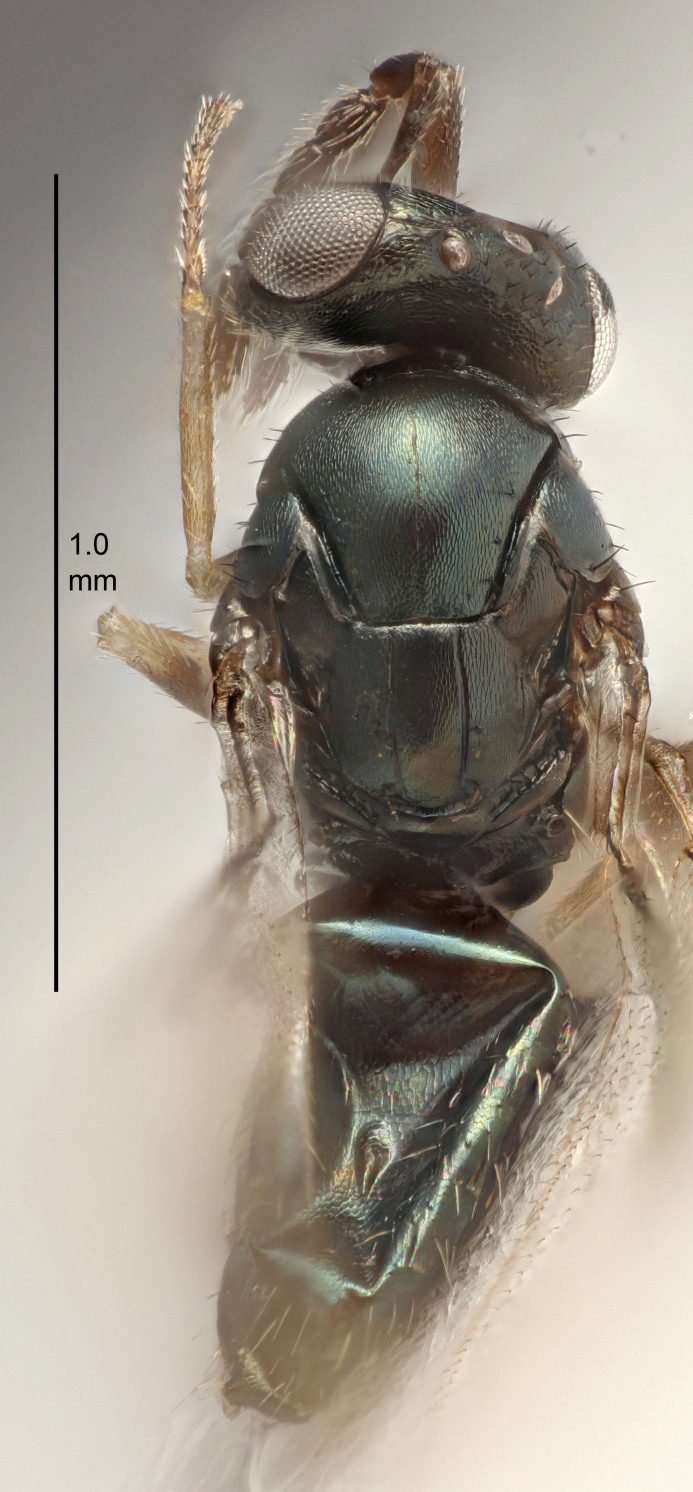
Holotype female, dorsal.

**Figure 51a.**
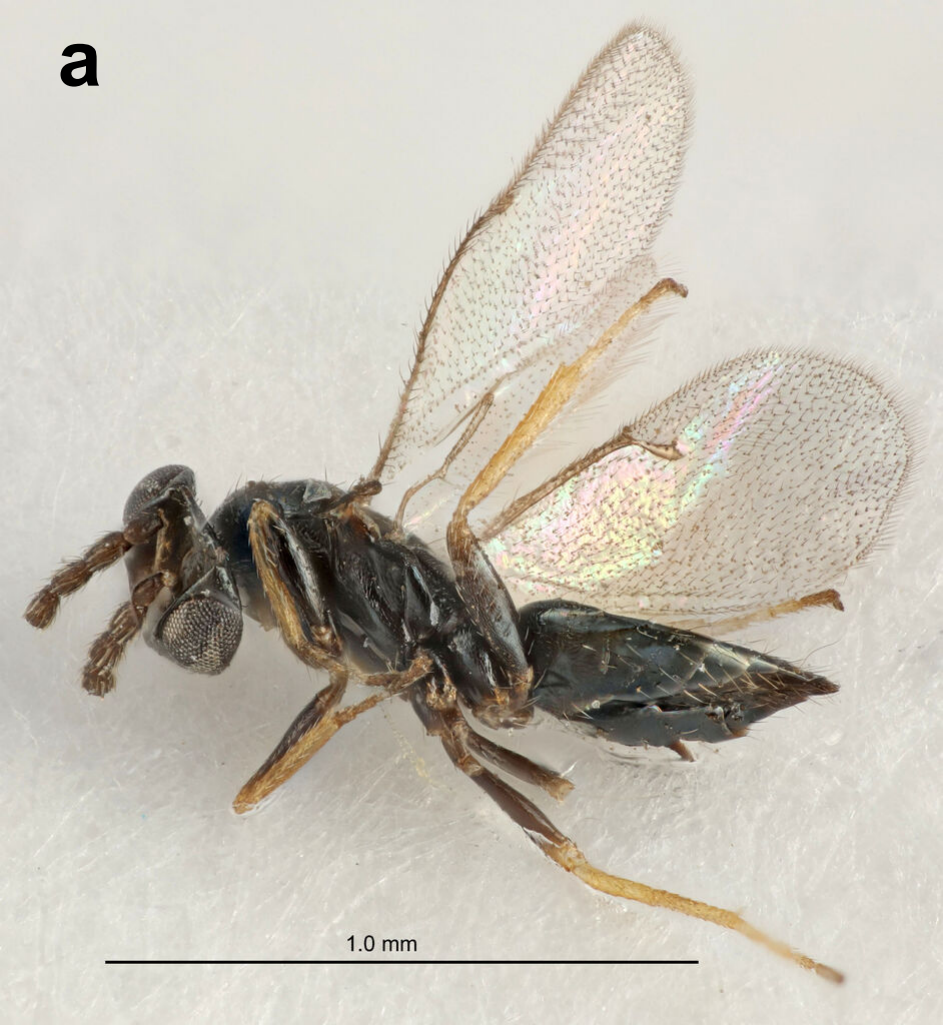
Holotype female, lateral.

**Figure 51b.**
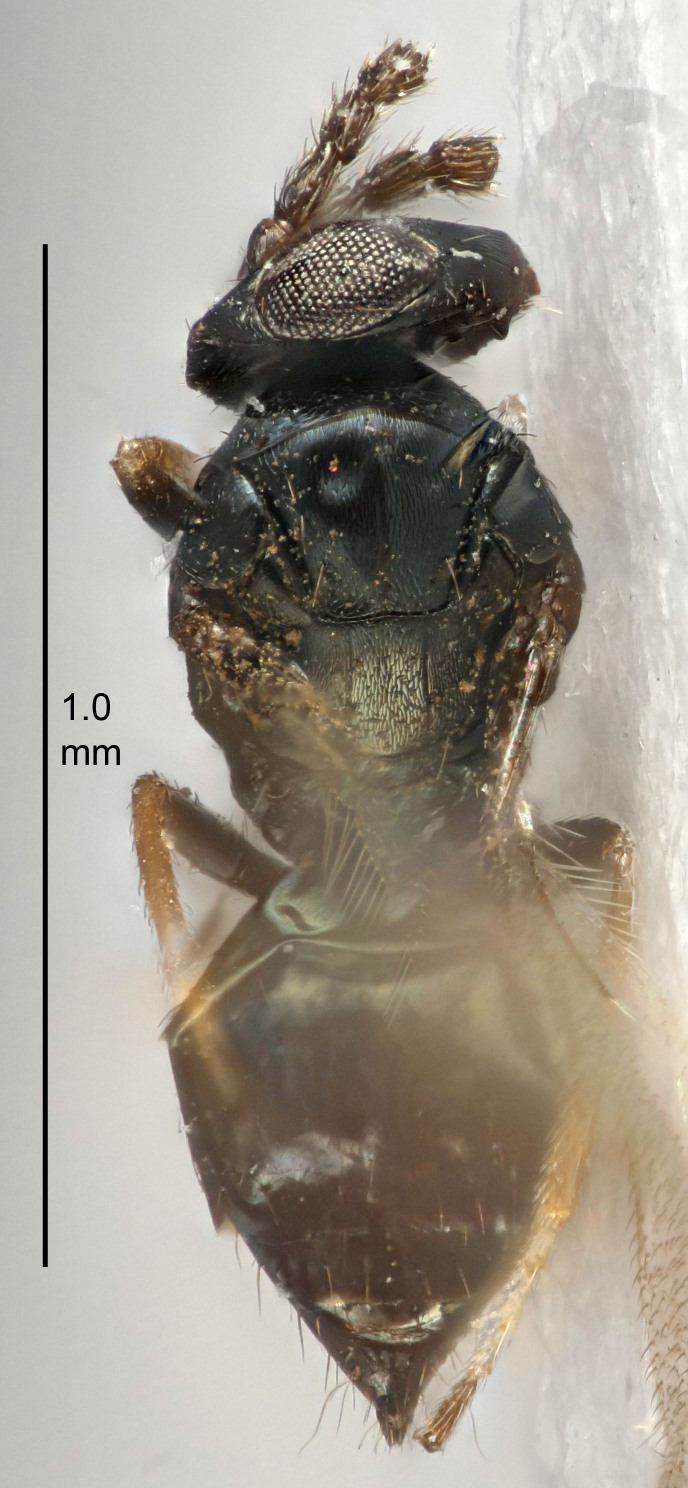
Holotype female, dorsal.

**Figure 51c.**
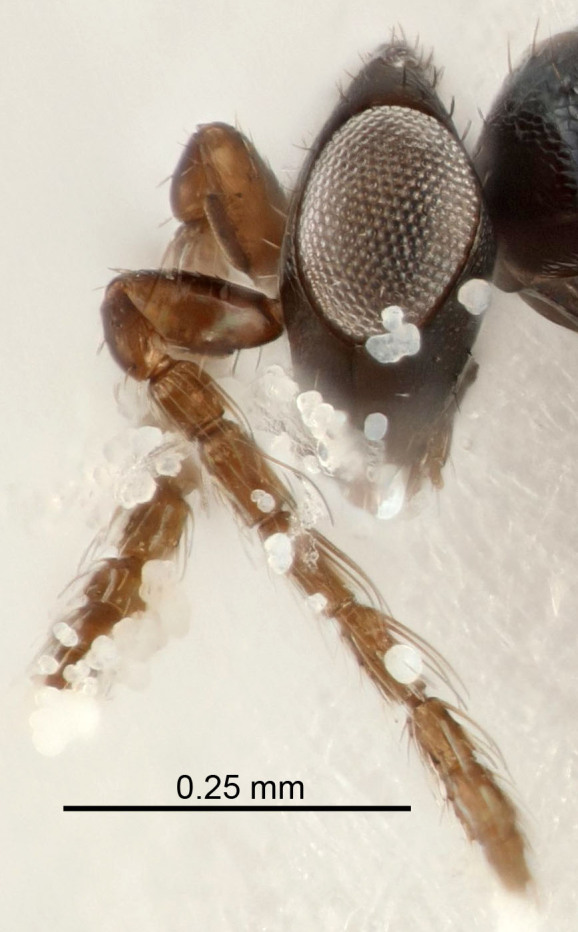
Paratype male, head and antenna lateral.

**Figure 52a.**
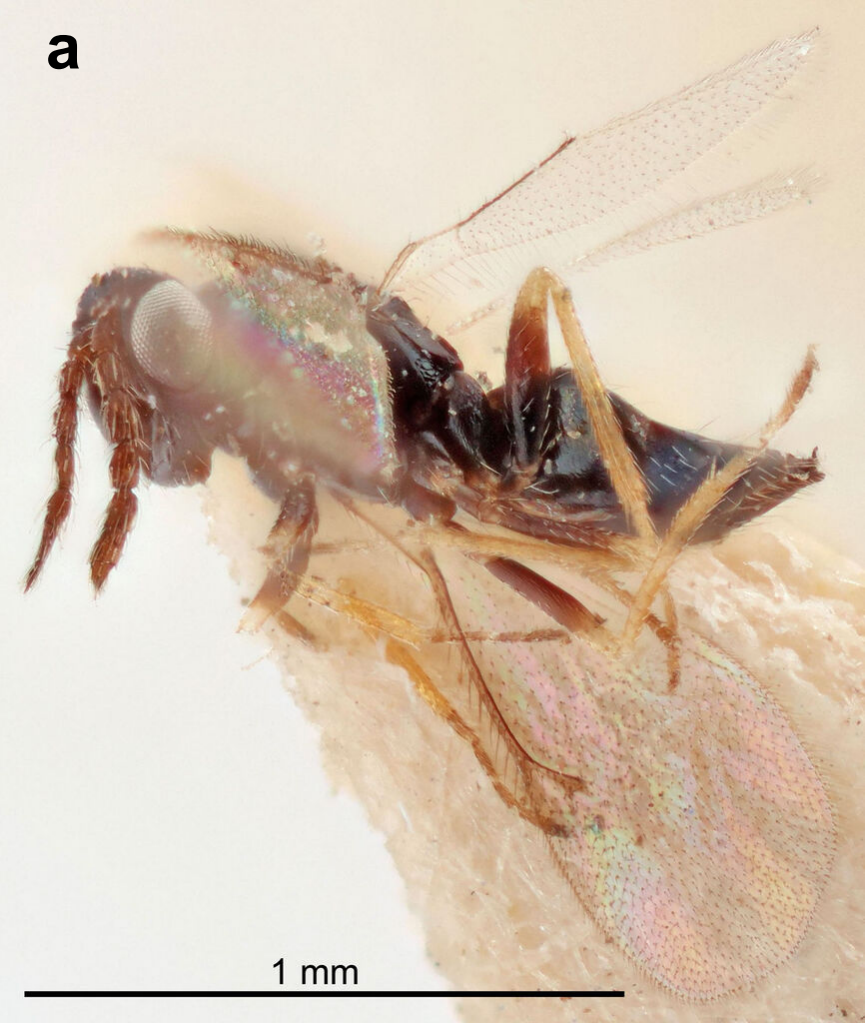
Paratype female, lateral.

**Figure 52b.**
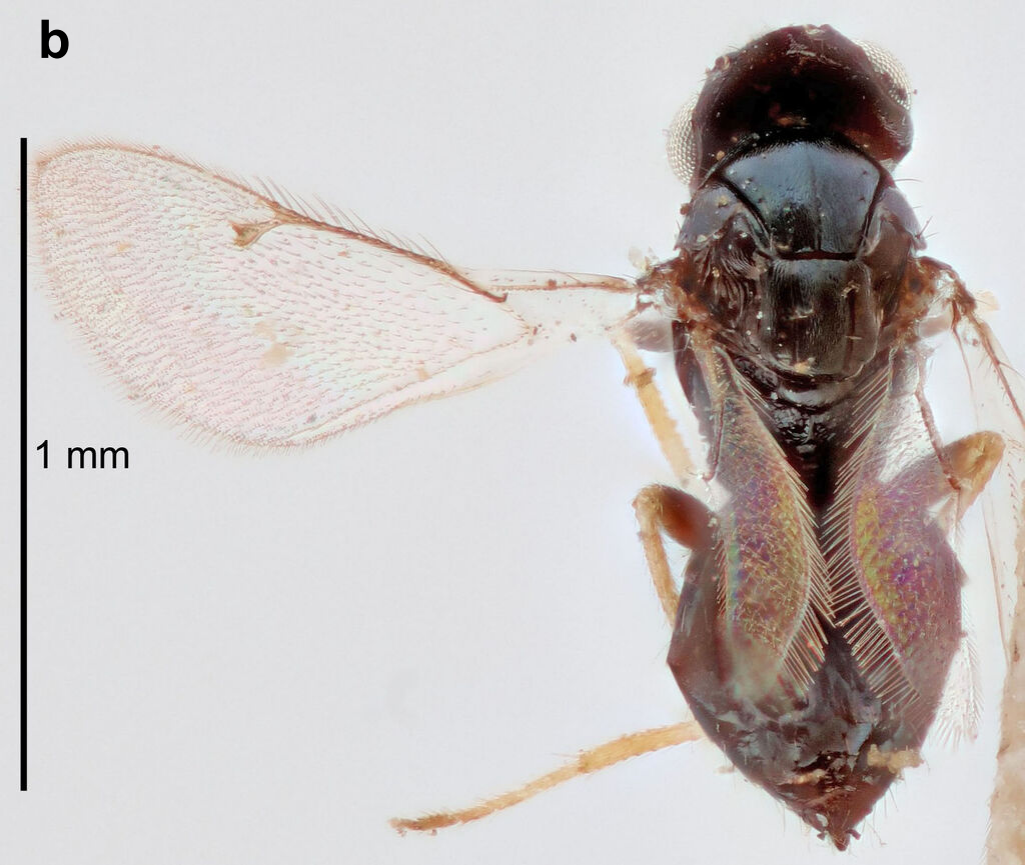
Paratype female, dorsal

**Figure 53a.**
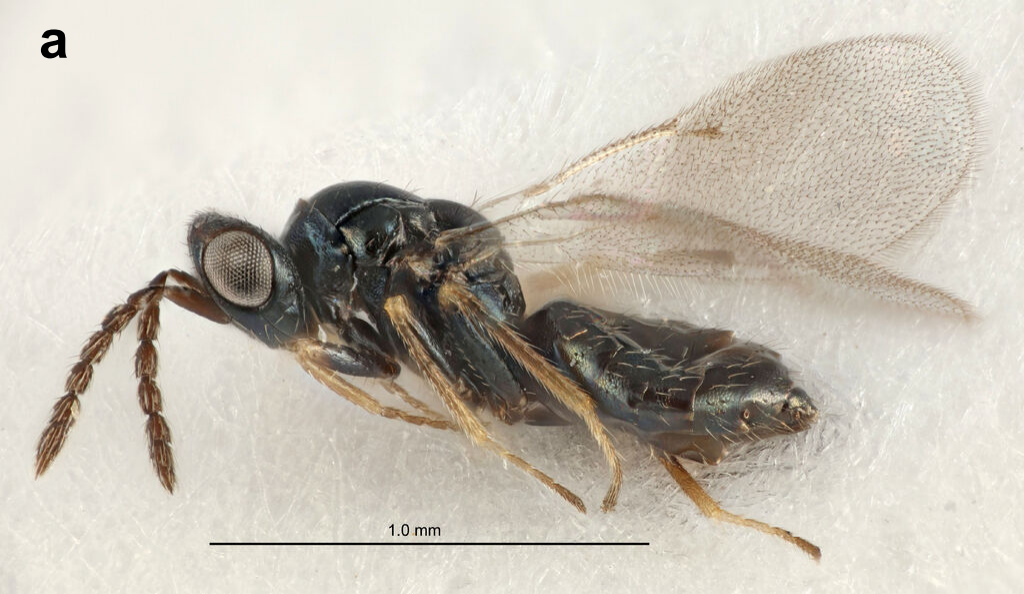
Holotype female, lateral.

**Figure 53b.**
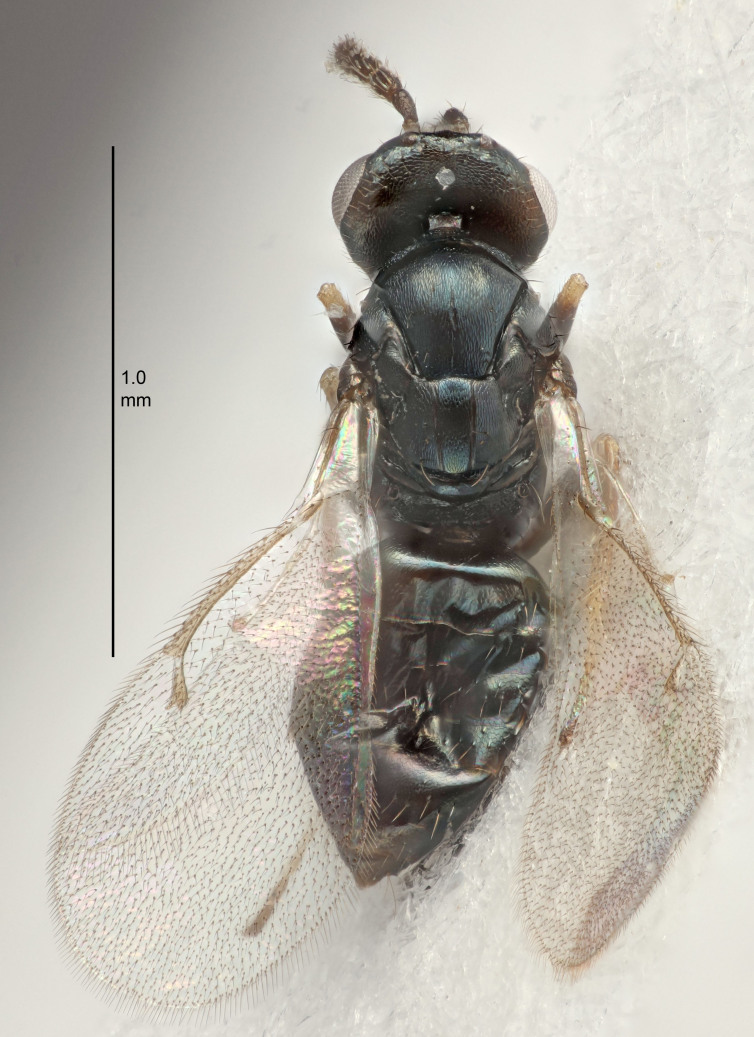
Holotype female, dorsal.

**Figure 54a.**
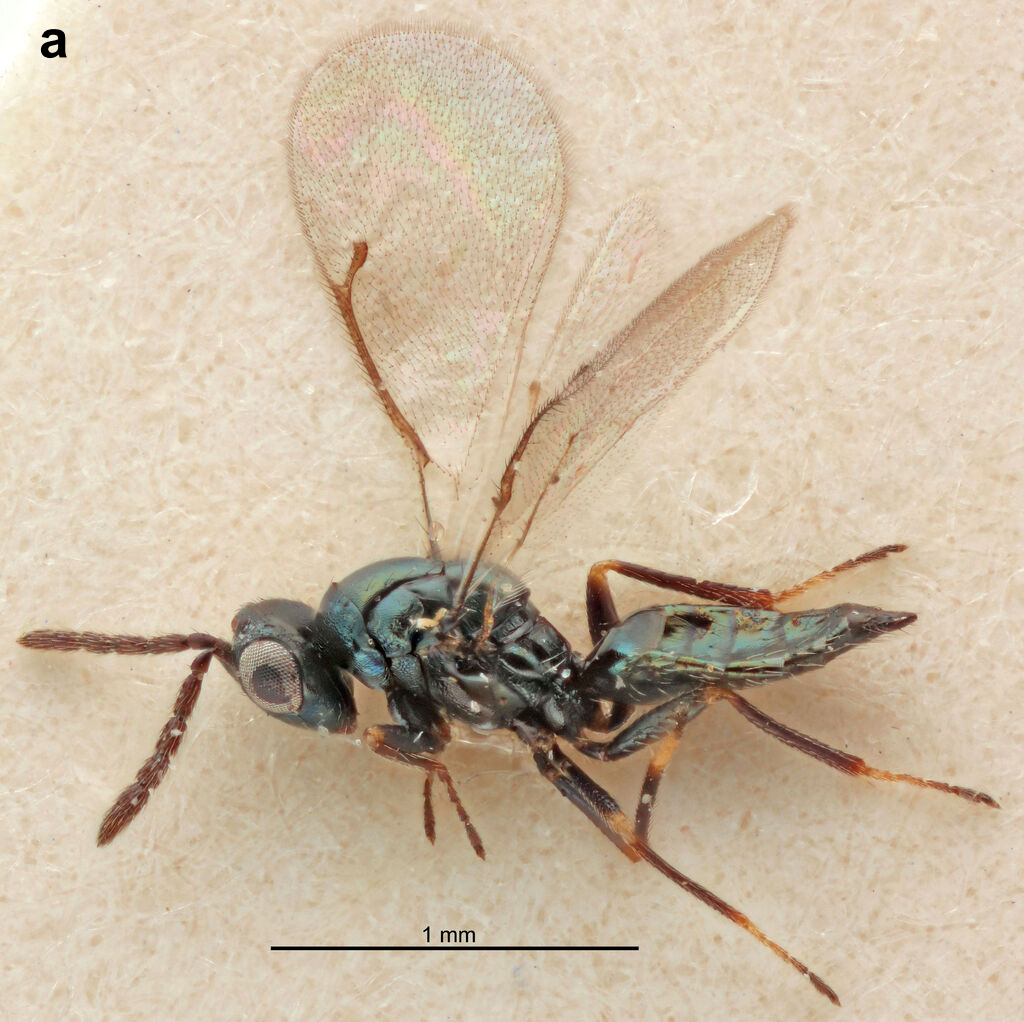
Female holotype, lateral.

**Figure 54b.**
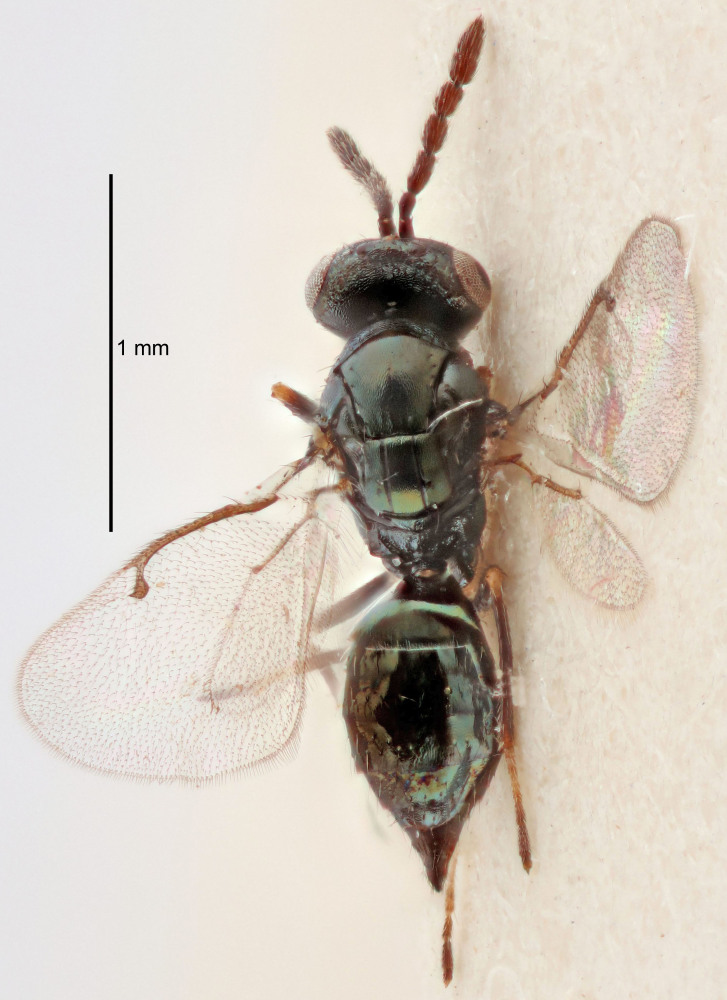
Female holotype, dorsal.

**Figure 55a.**
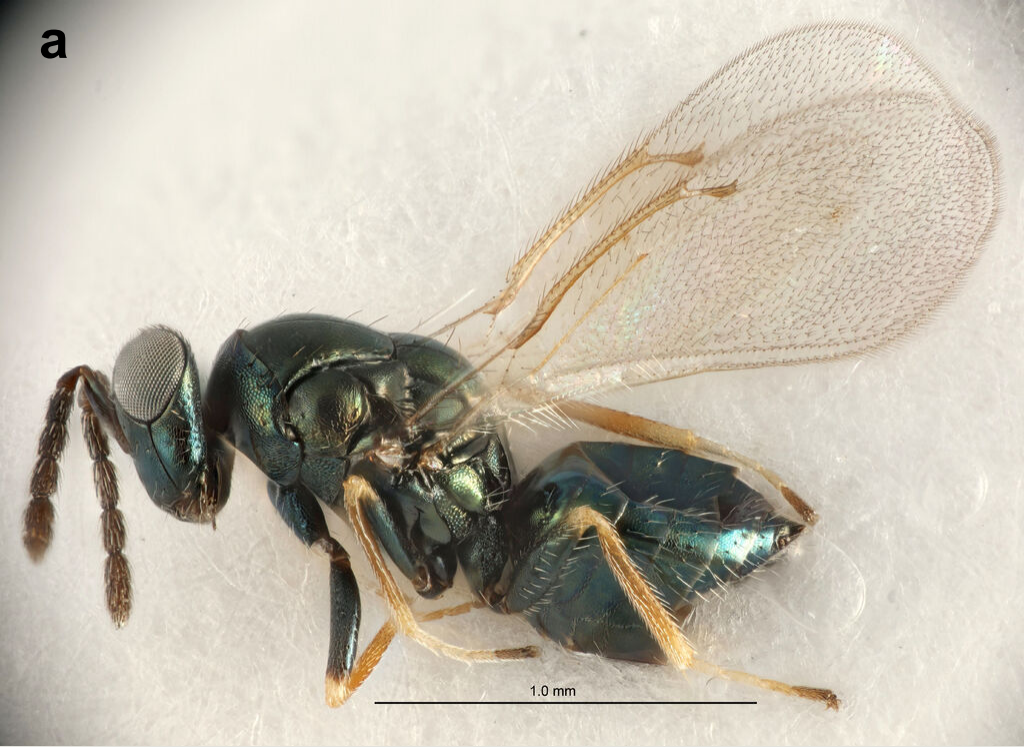
Holotype female, lateral.

**Figure 55b.**
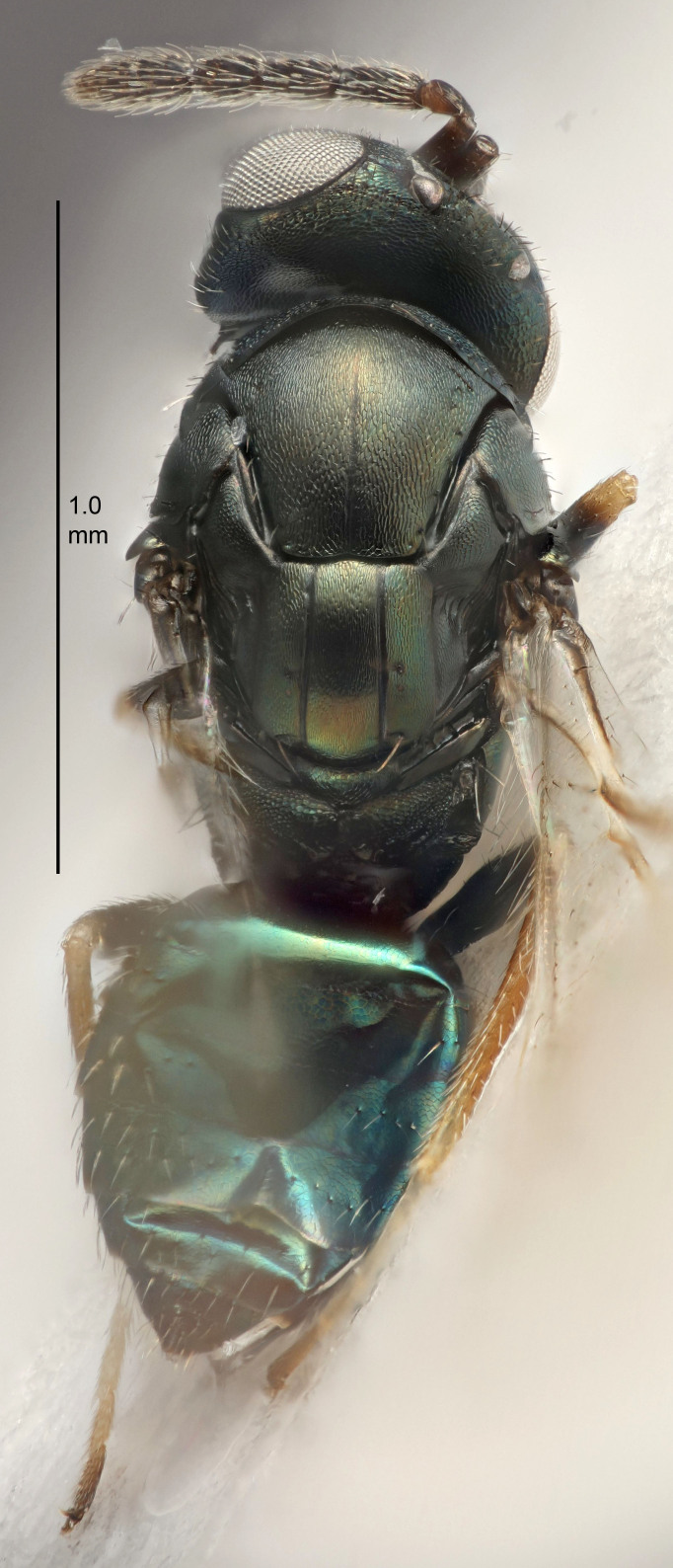
Holotype female, dorsal.

**Figure 55c.**
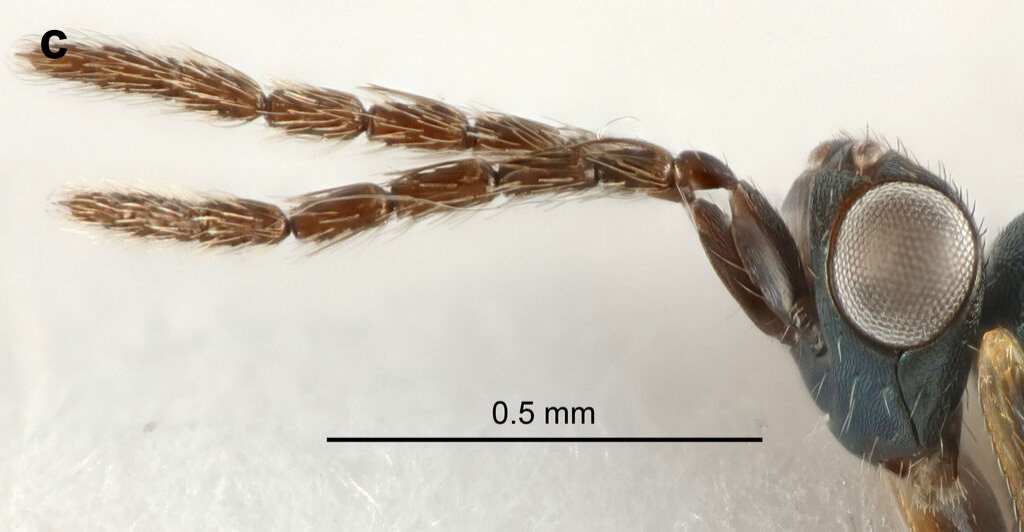
Paratype male, head antenna lateral.

**Figure 56a.**
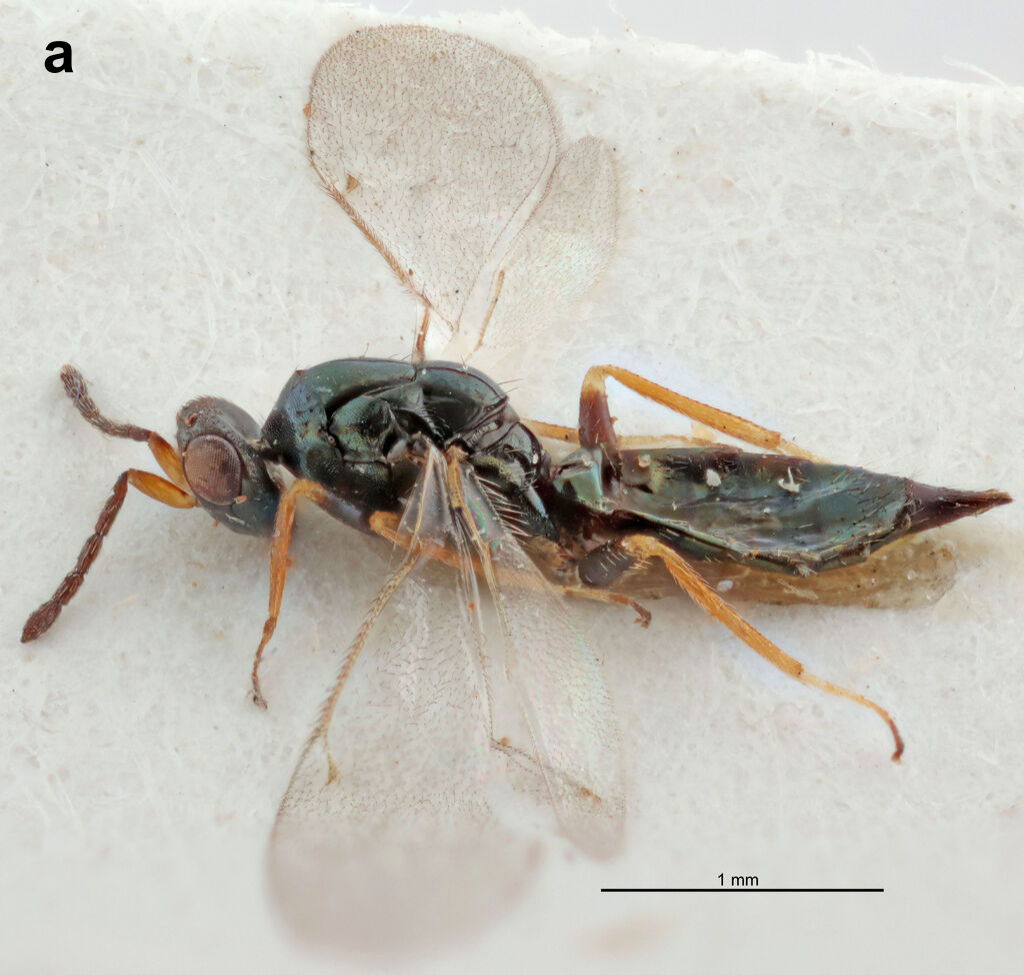
Holotype female, lateral.

**Figure 56b.**
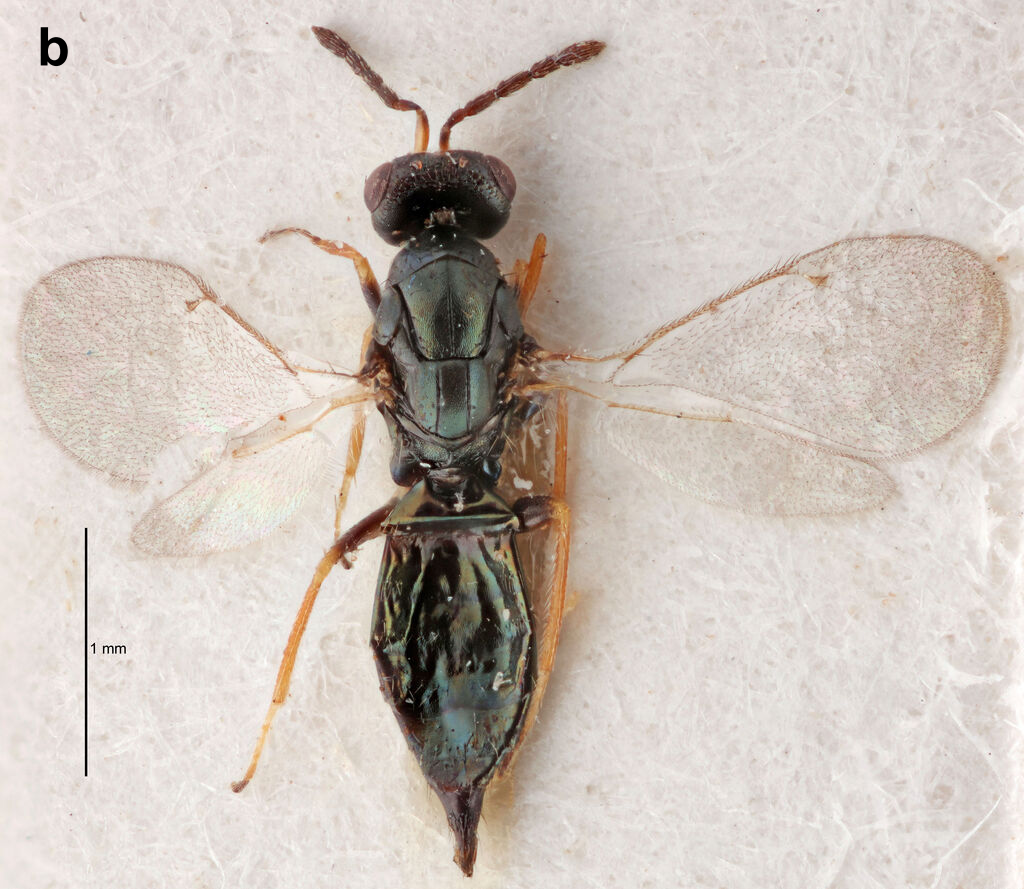
Holotype female, dorsal.

**Figure 56c.**
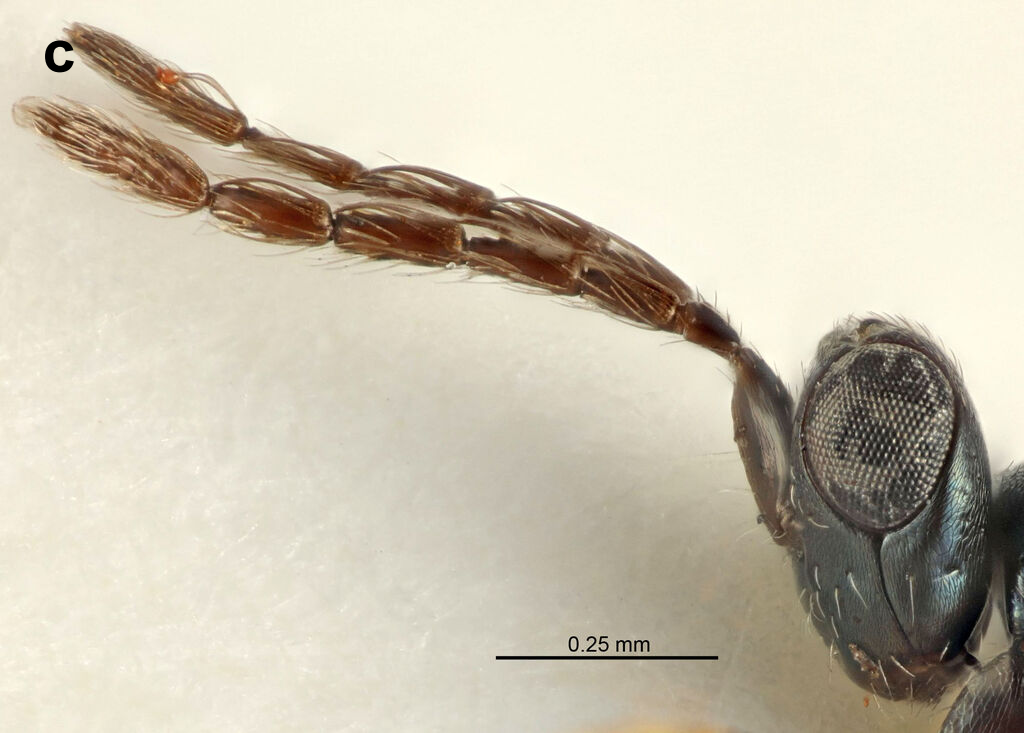
Nontype male, head antenna lateral.

**Figure 57a.**
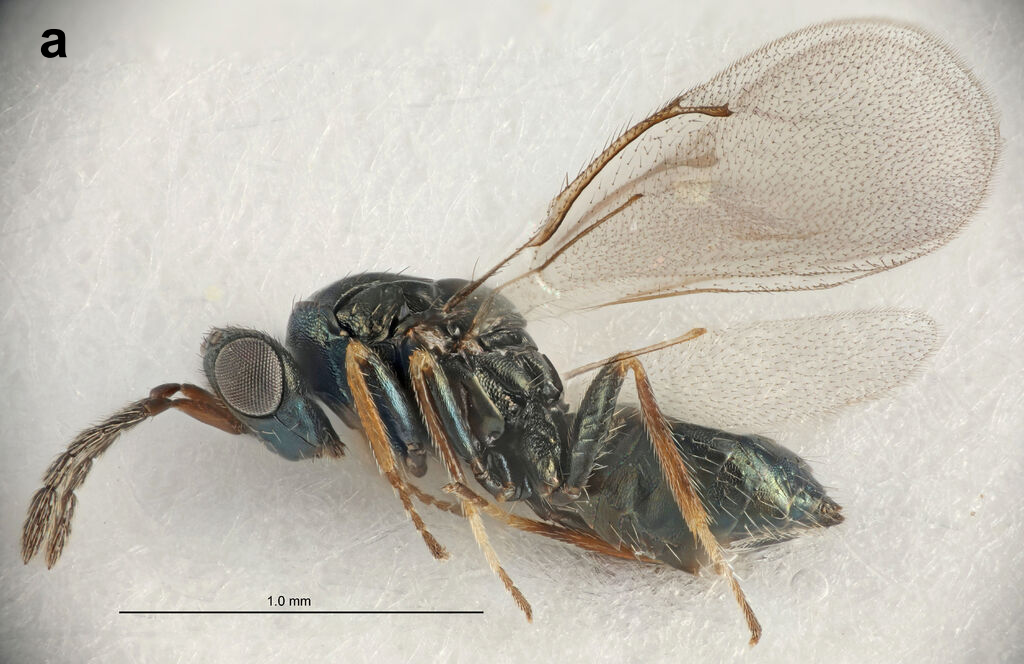
Holotype female, lateral.

**Figure 57b.**
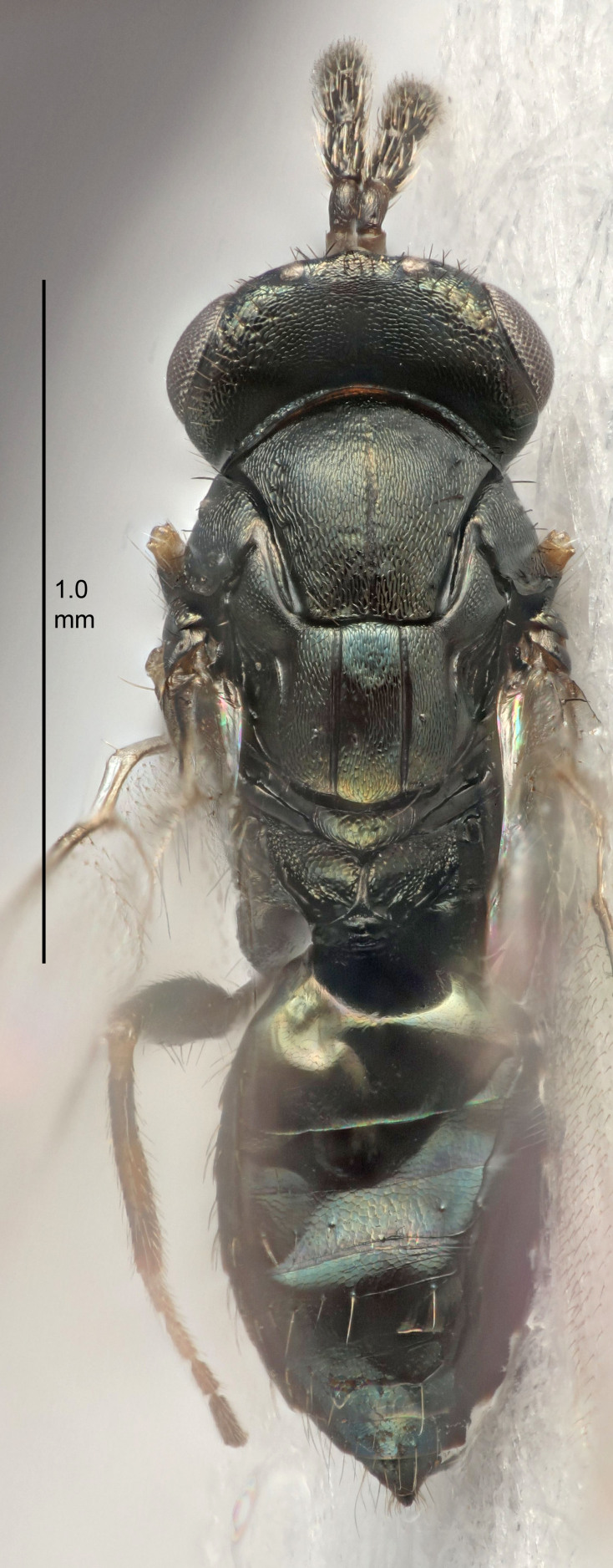
Holotype female, dorsal.

**Figure 58a.**
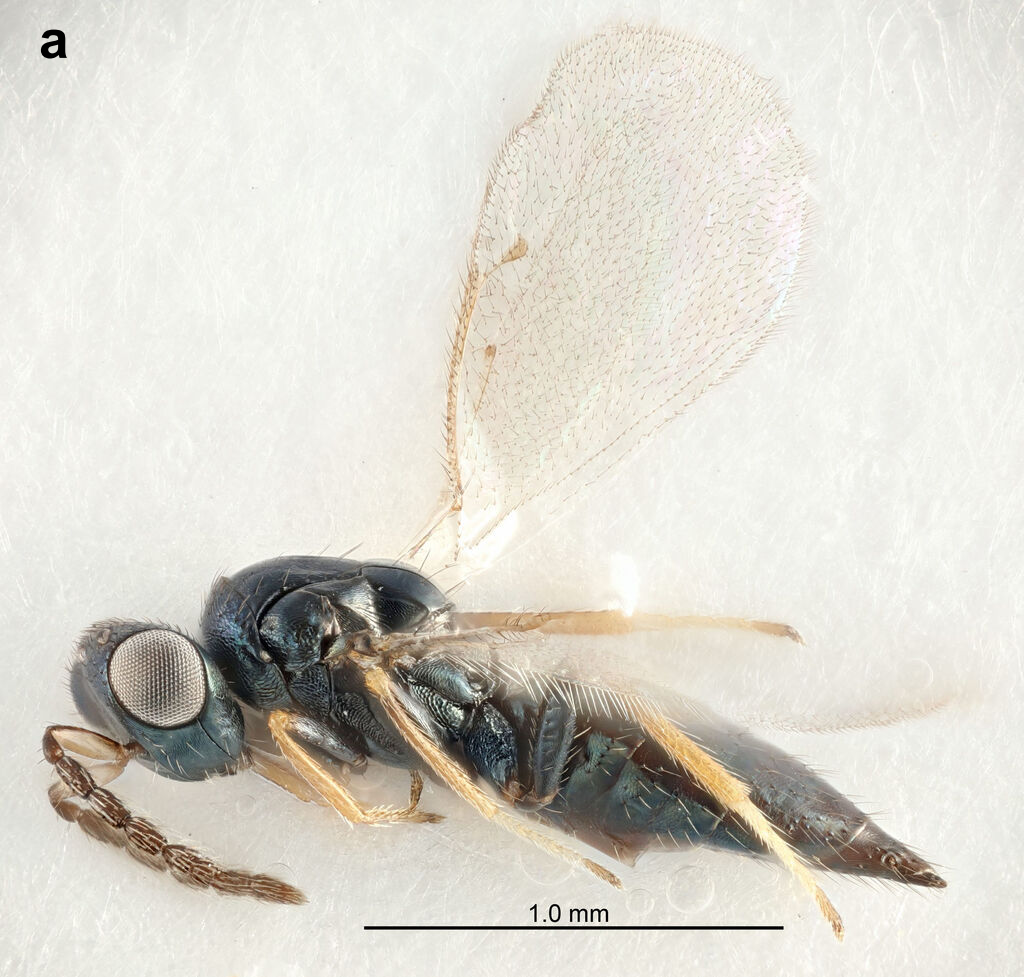
Holotype female, lateral.

**Figure 58b.**
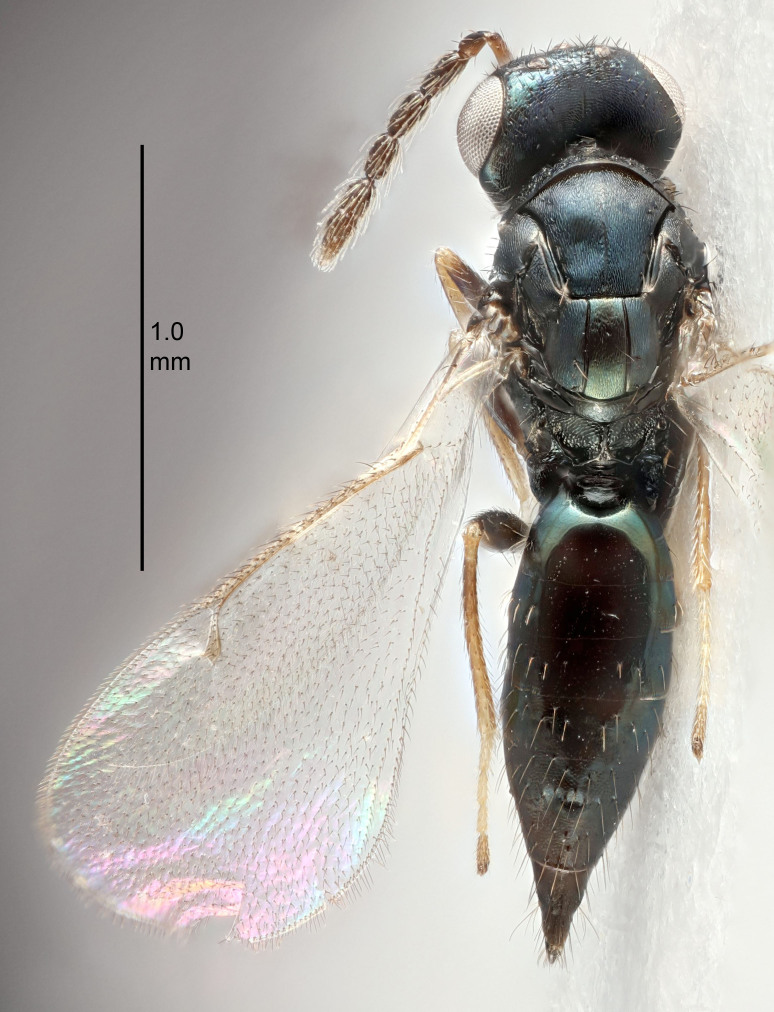
Holotype female, dorsal.

**Figure 59a.**
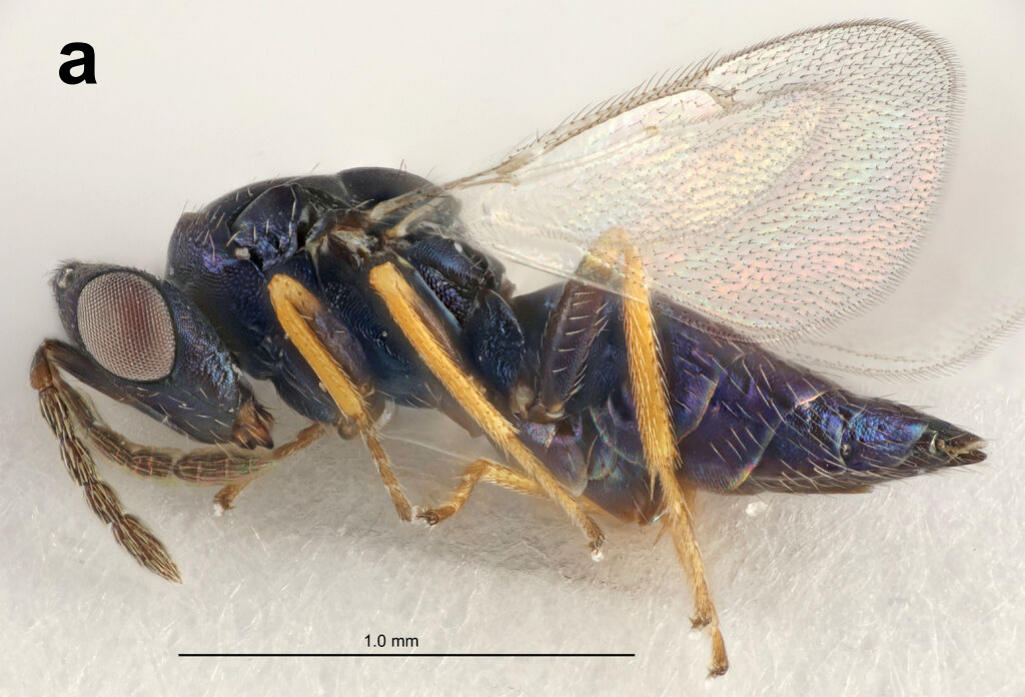
Nontype female, lateral.

**Figure 59b.**
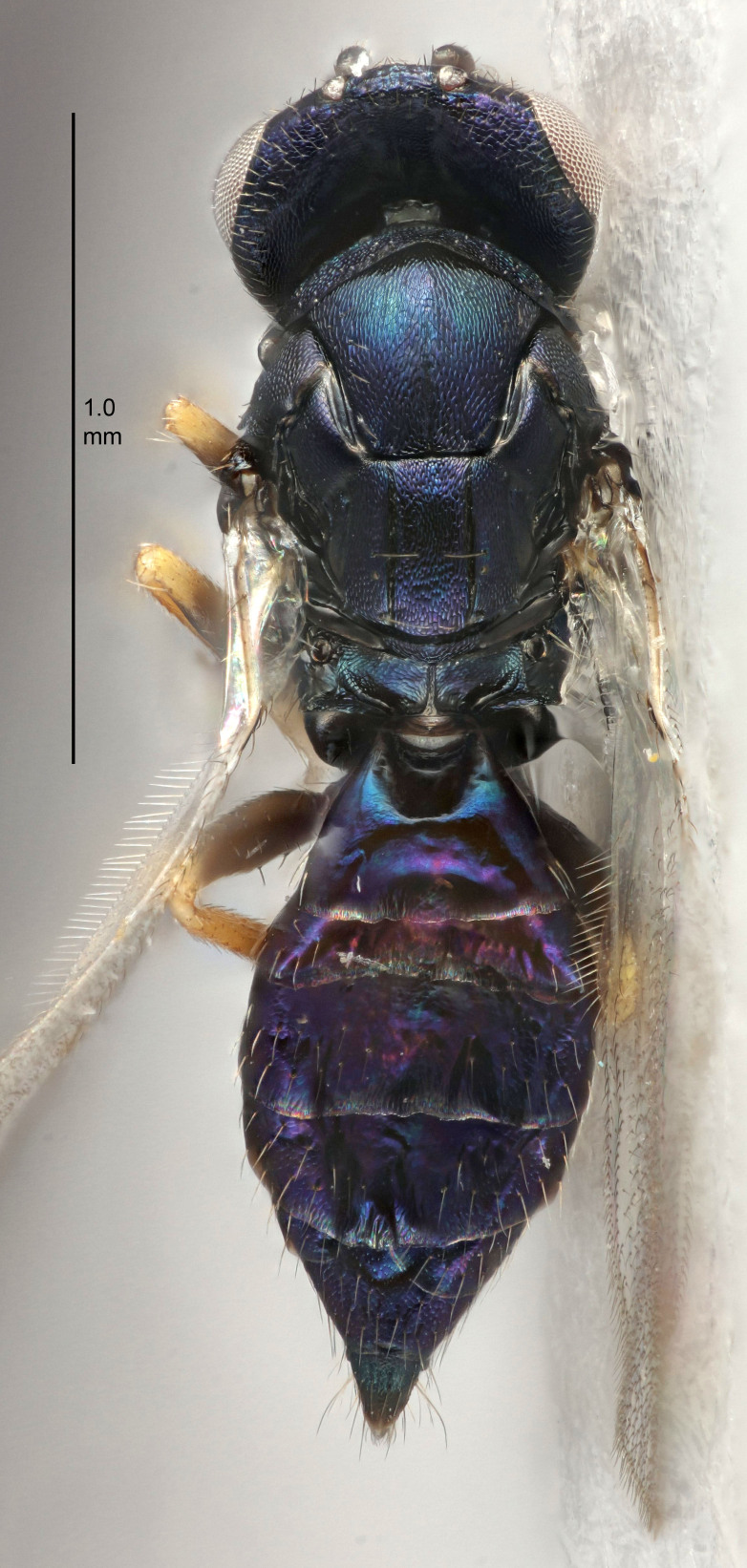
Nontype female, dorsal.

**Figure 59c.**
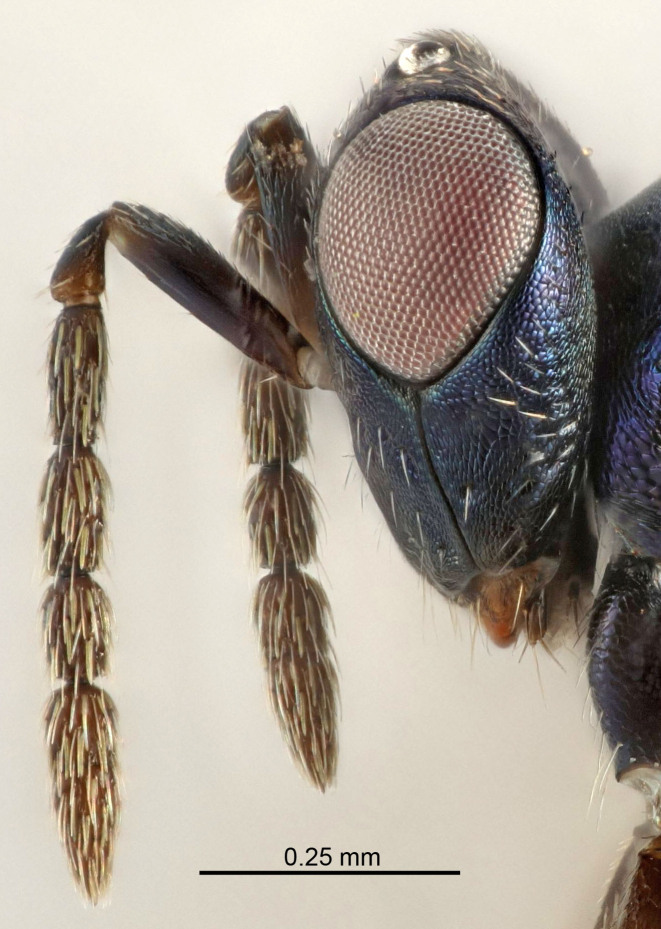
Nontype female, head and antenna lateral.

**Figure 59d.**
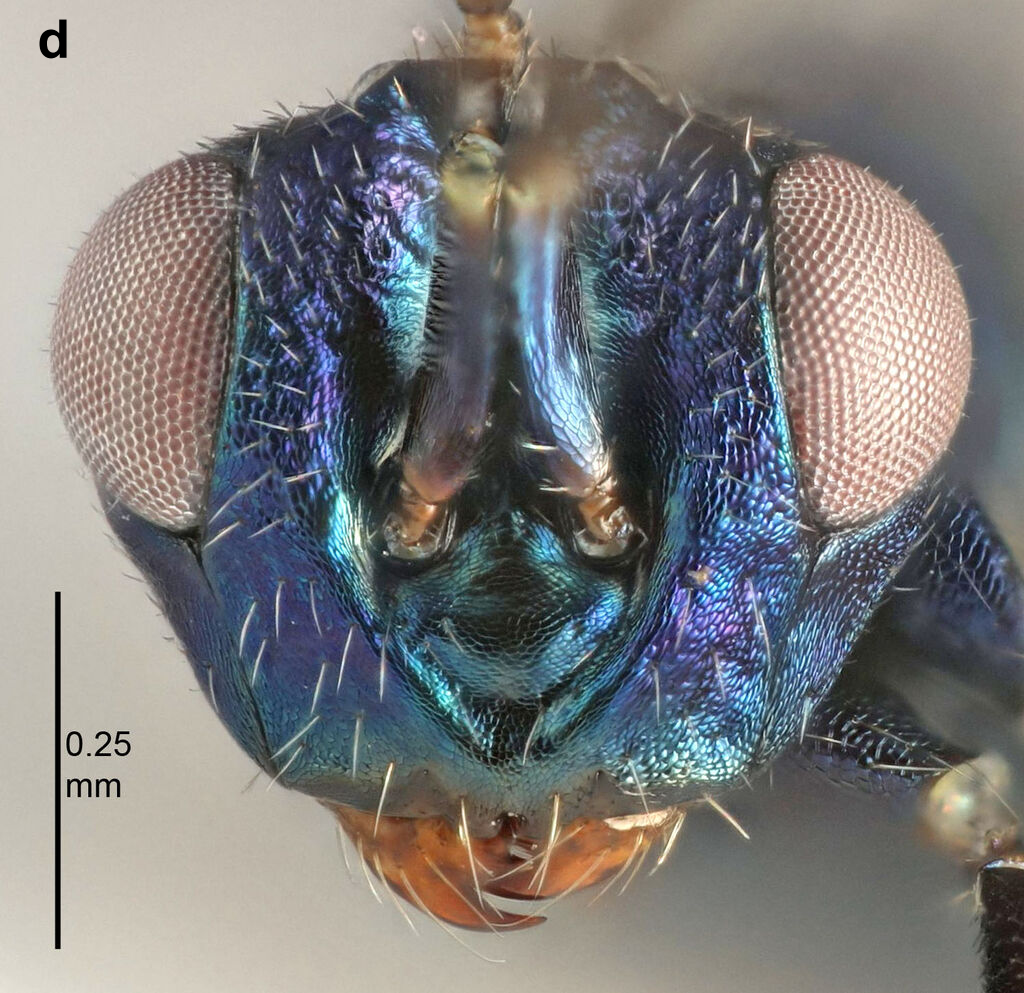
Nontype female, head frontal.

**Figure 59e.**
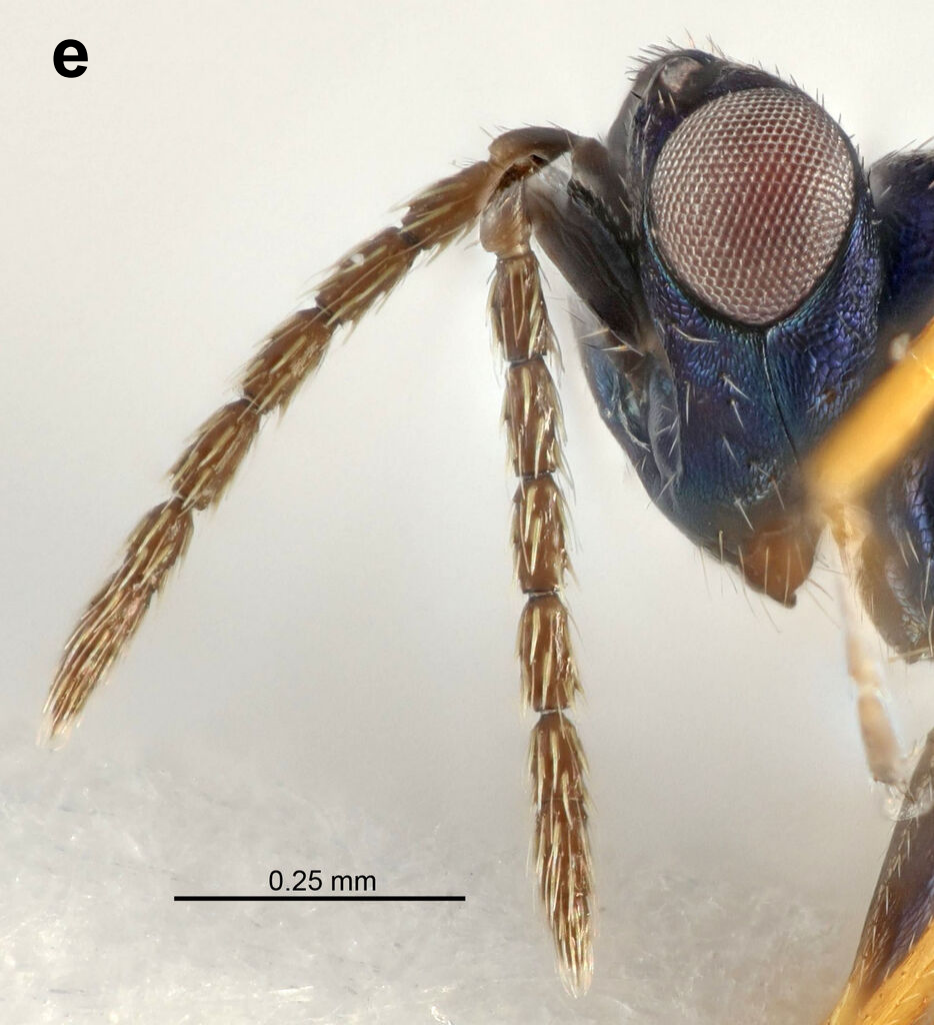
Nontype male, head and antenna lateral.

**Figure 60a.**
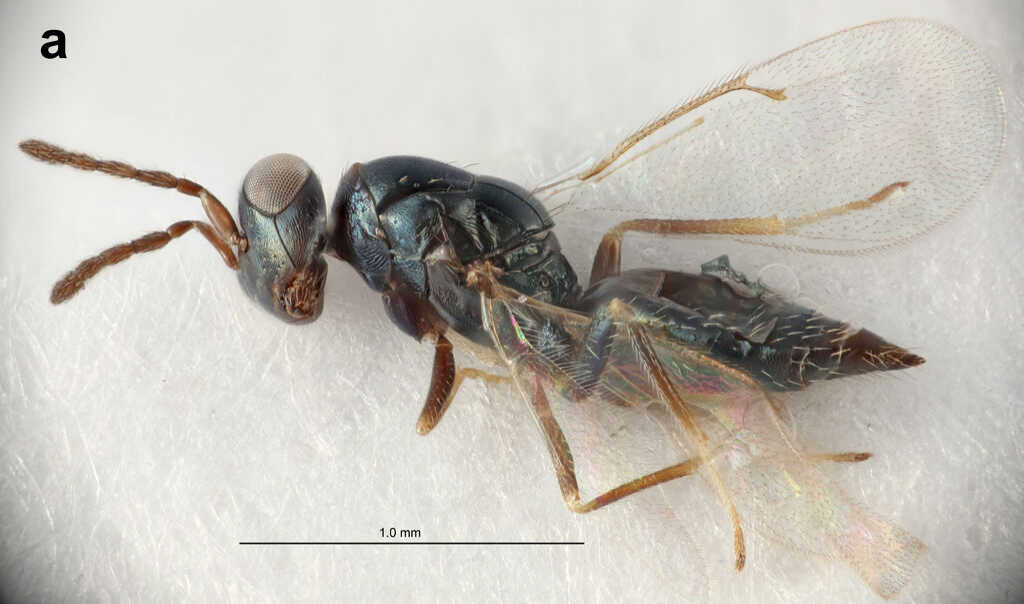
Holotype female, lateral.

**Figure 60b.**
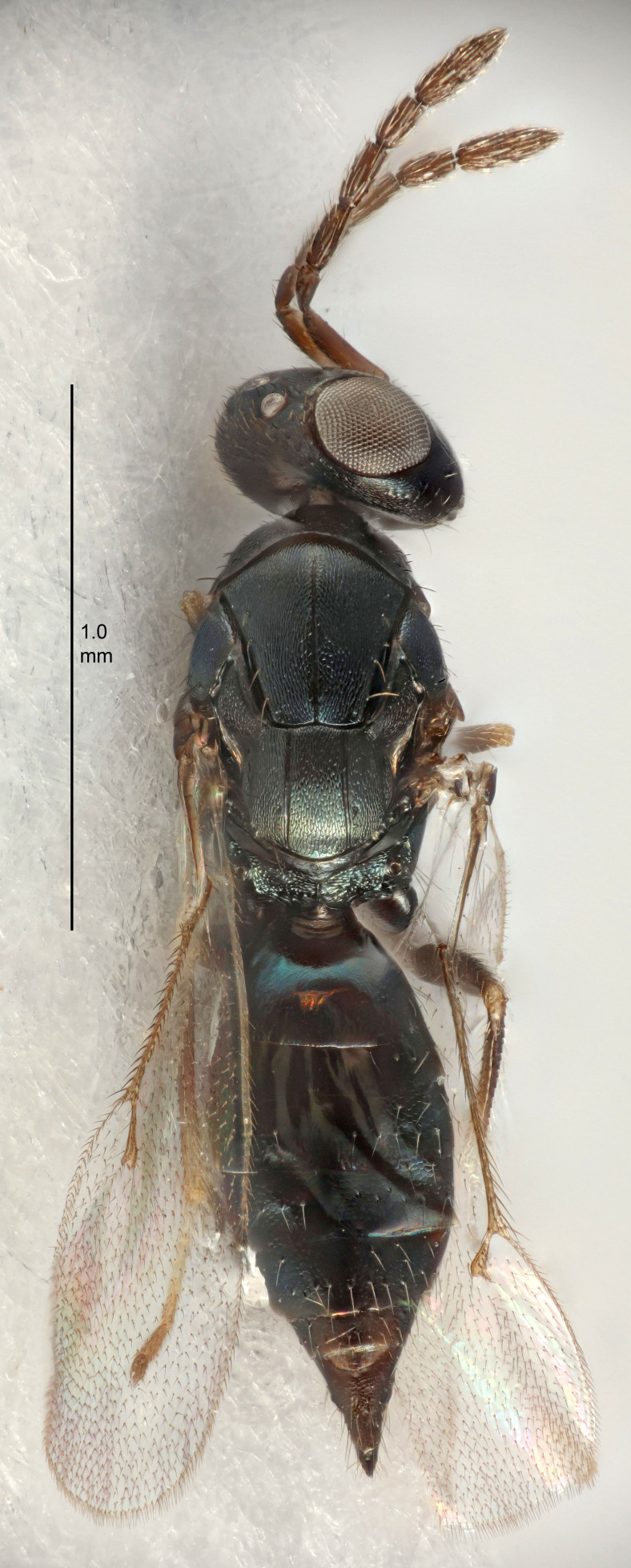
Holotype female, dorsal.

**Figure 61a.**
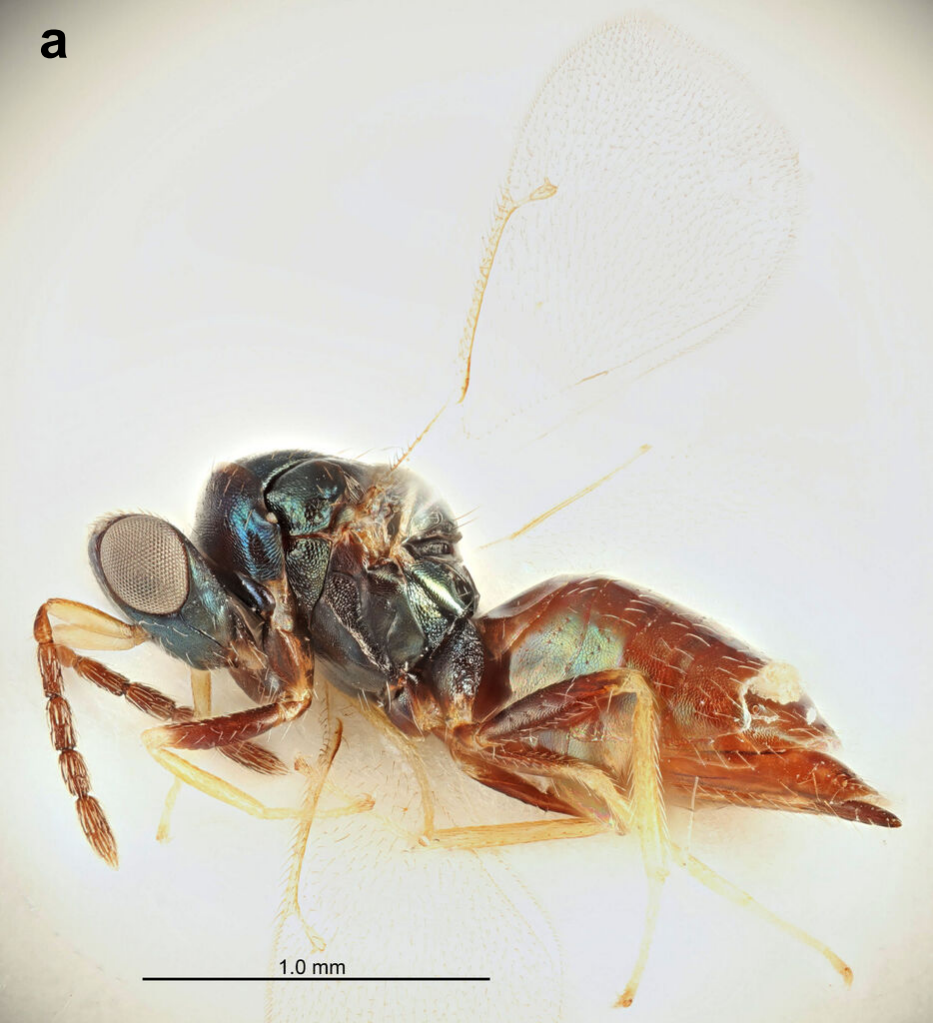
Holotype female, lateral.

**Figure 61b.**
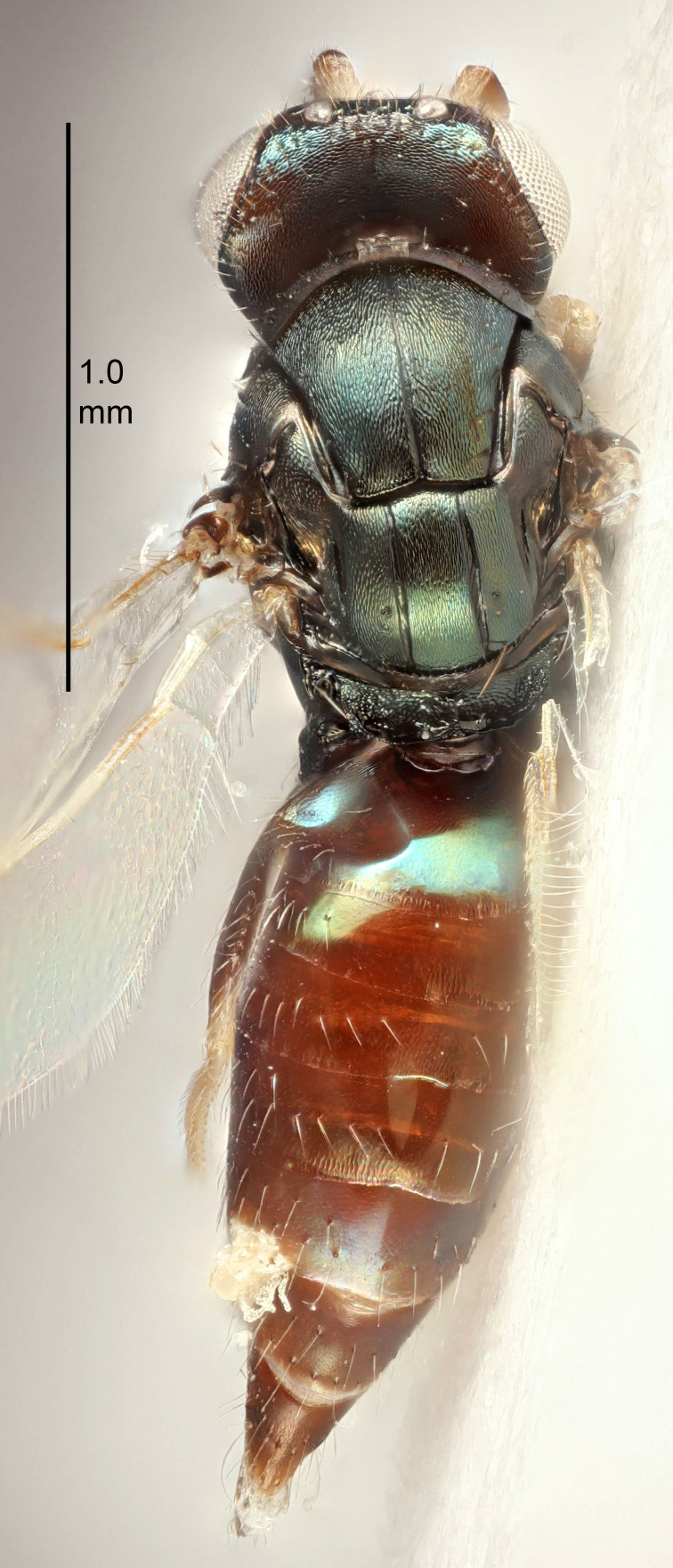
Holotype female, dorsal.

**Figure 62a.**
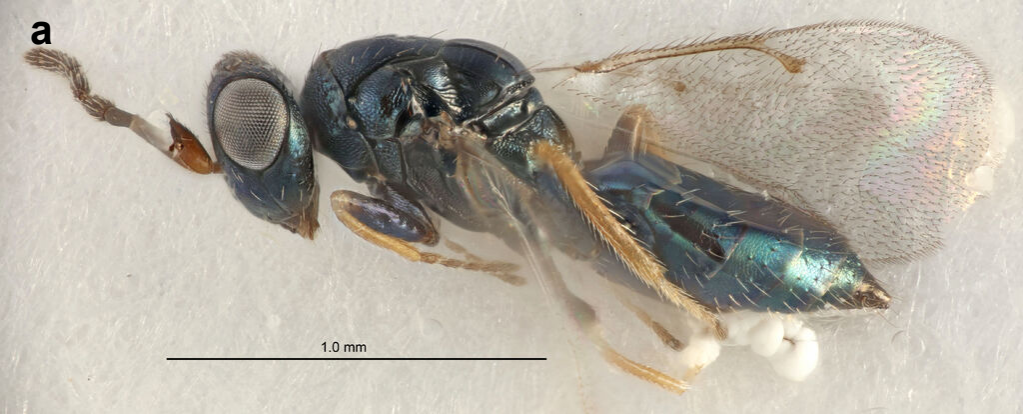
Holotype female, lateral.

**Figure 62b.**
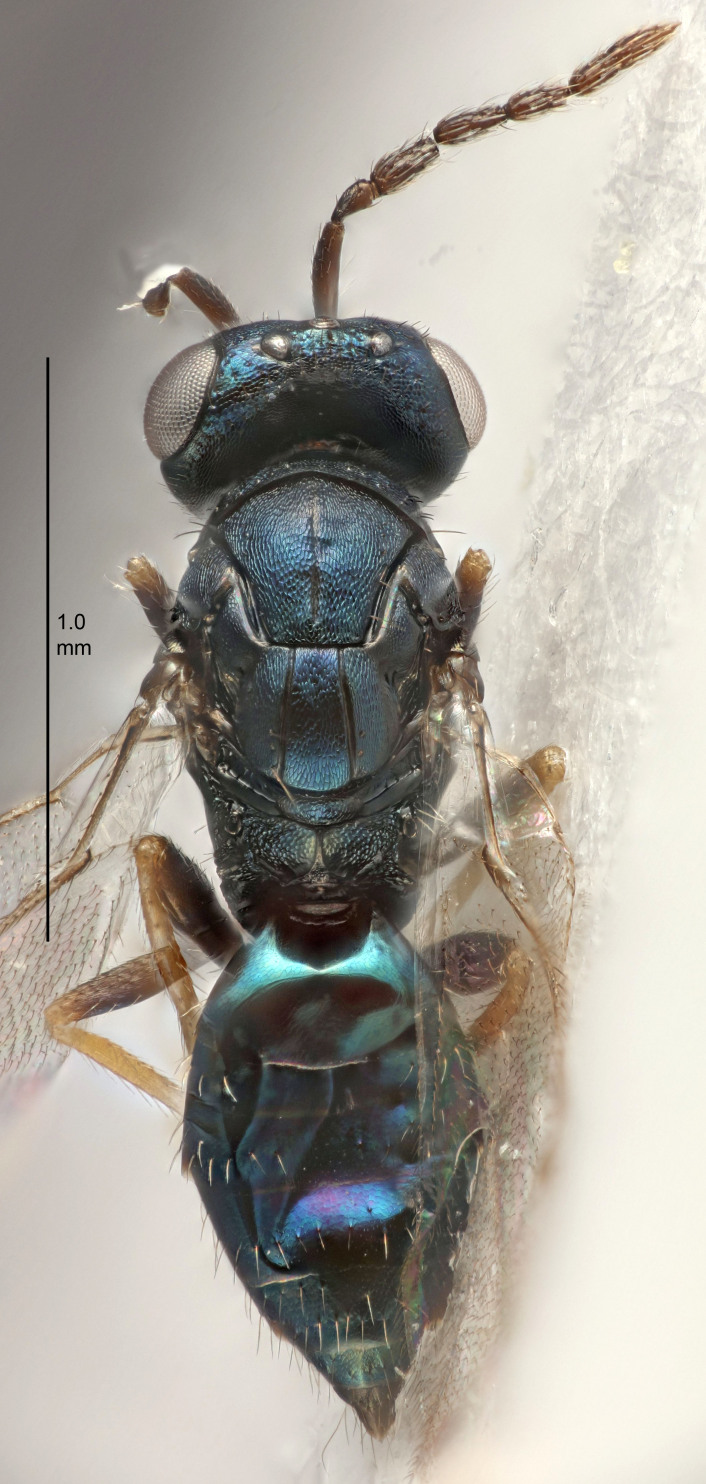
Holotype female, dorsal

**Figure 63a.**
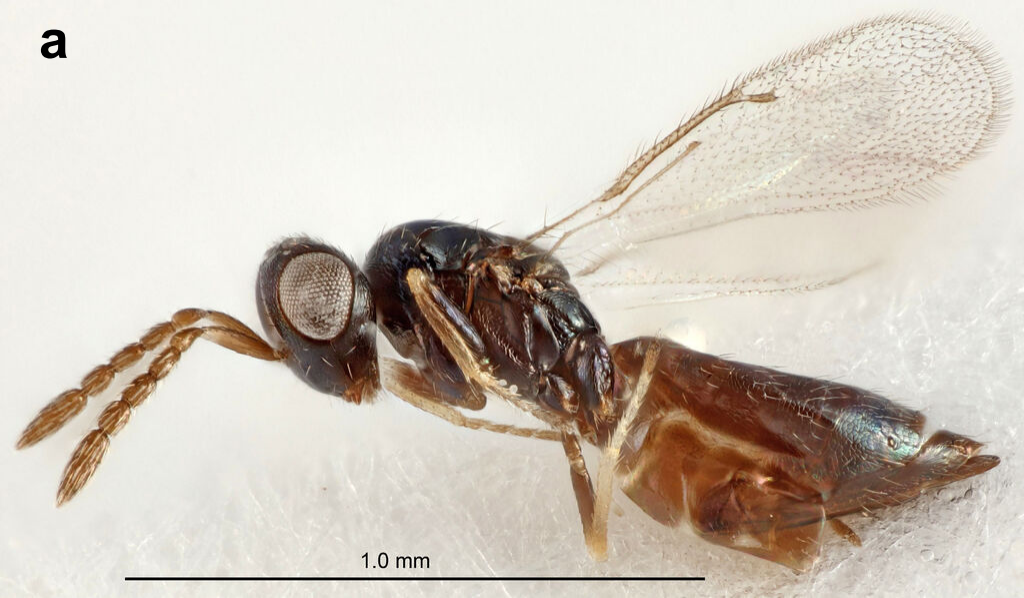
Holotype female, lateral.

**Figure 63b.**
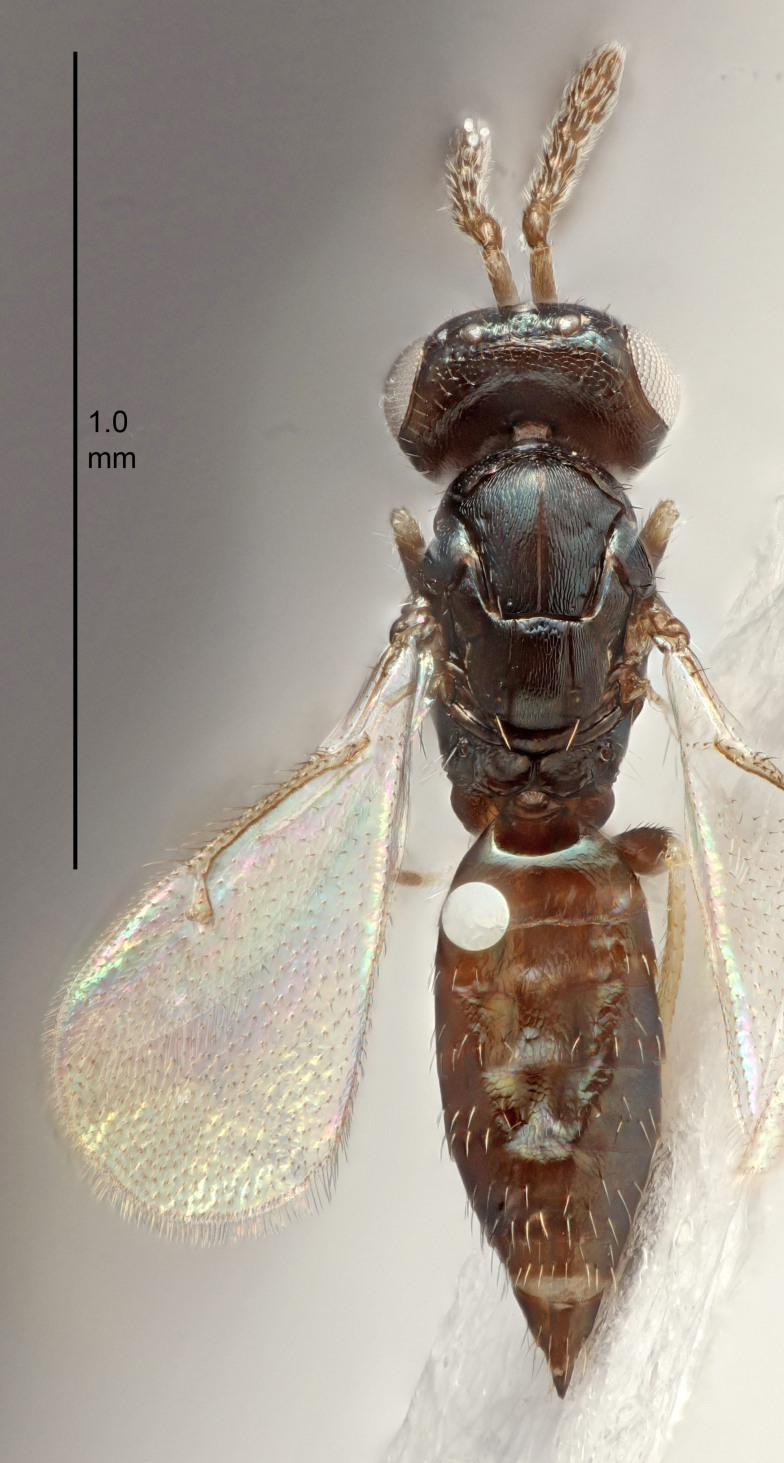
Holotype female, dorsal.

**Figure 63c.**
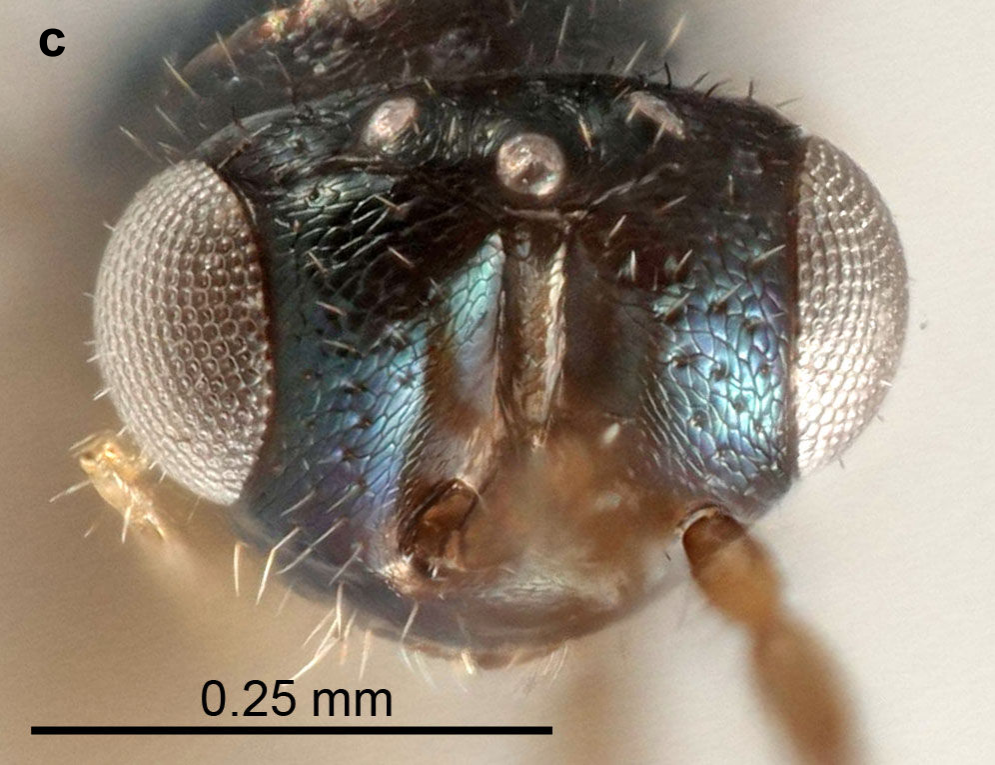
Holotype female, head frontal.

**Figure 64a.**
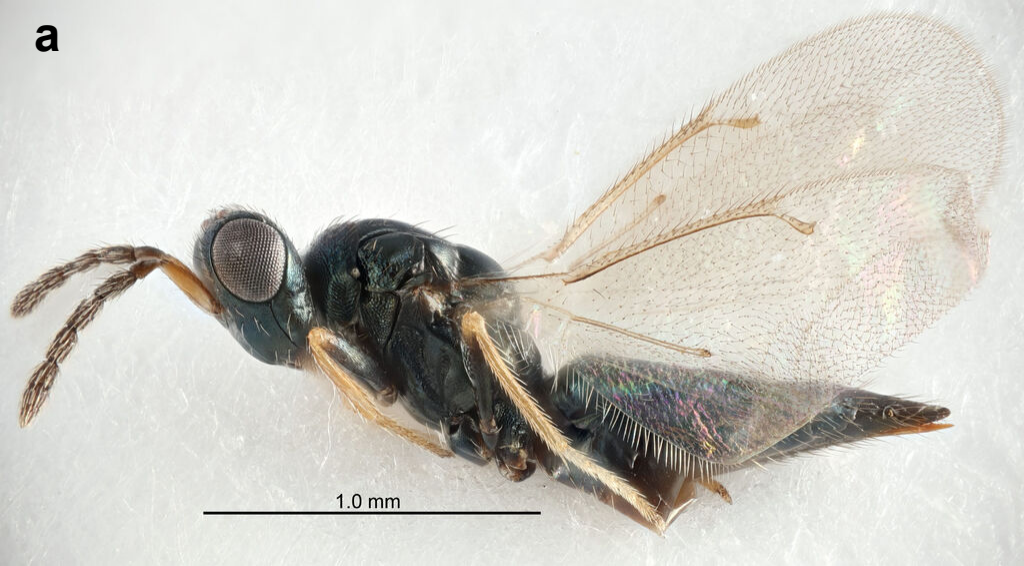
Holotype female, lateral.

**Figure 64b.**
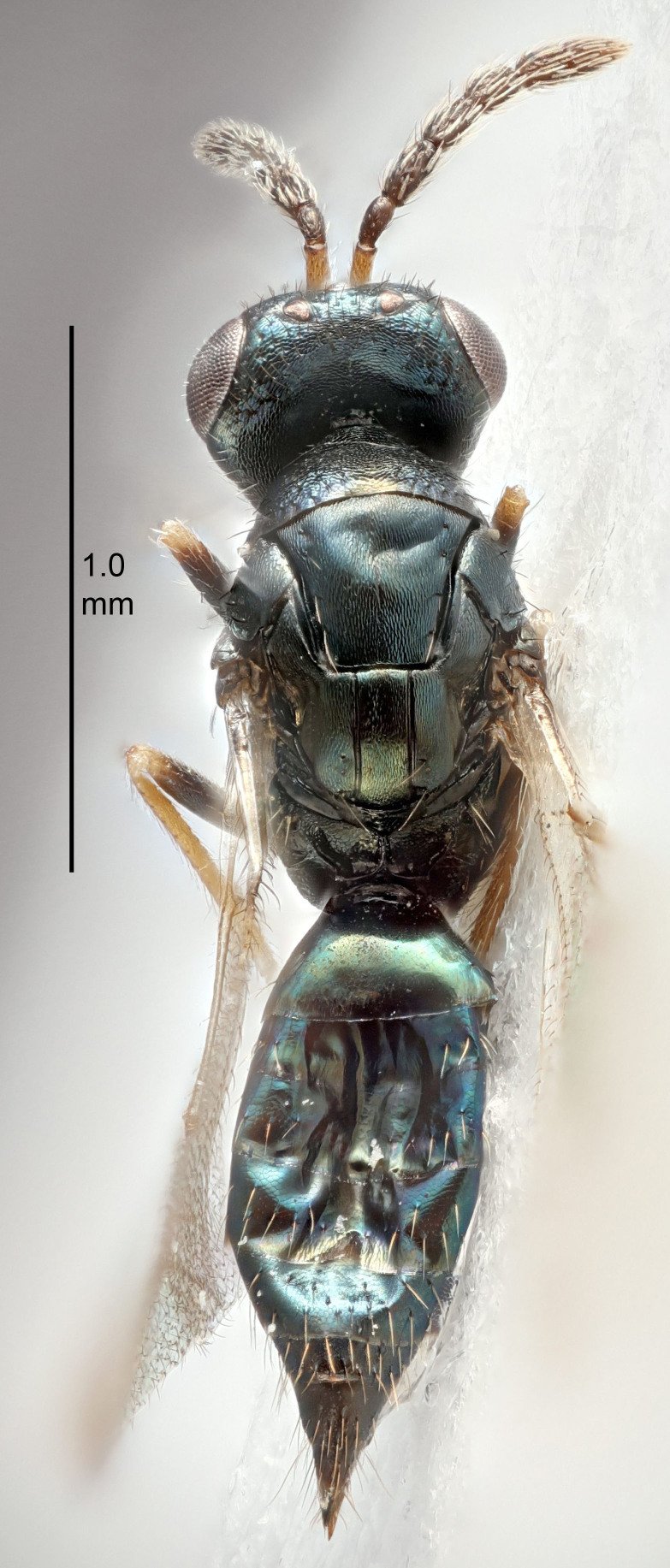
Holotype female, dorsal.

**Figure 65a.**
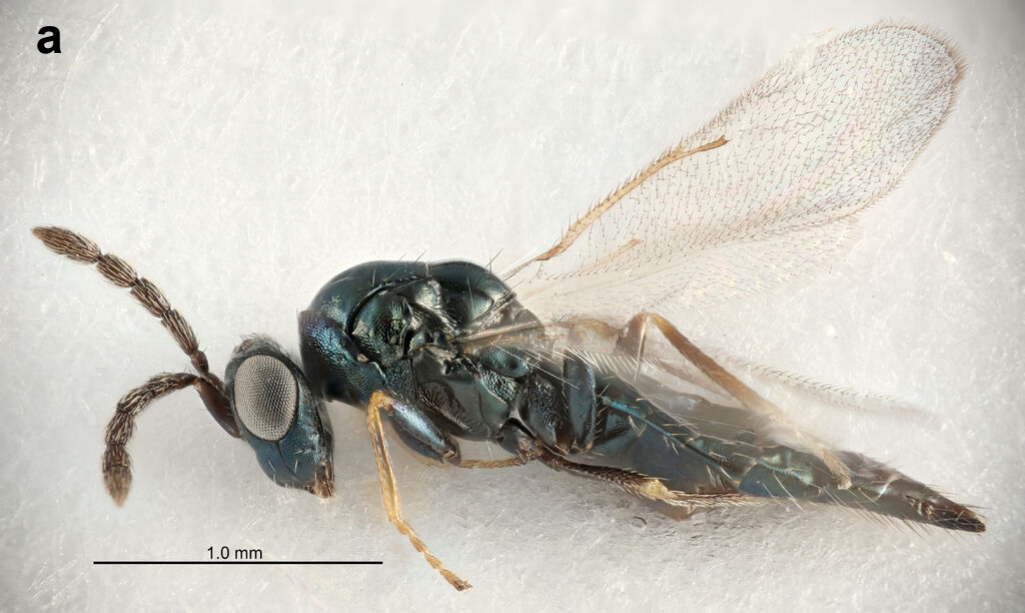
Holotype female, lateral.

**Figure 65b.**
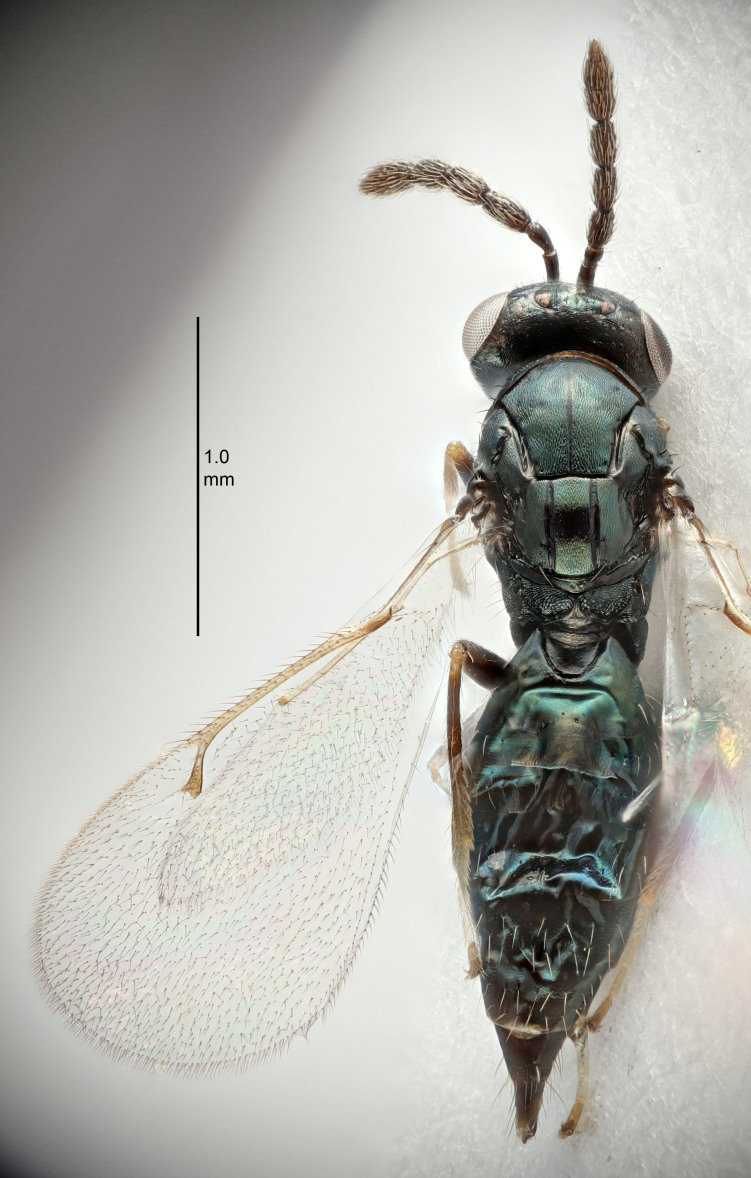
Holotype female, dorsal.

**Figure 66a.**
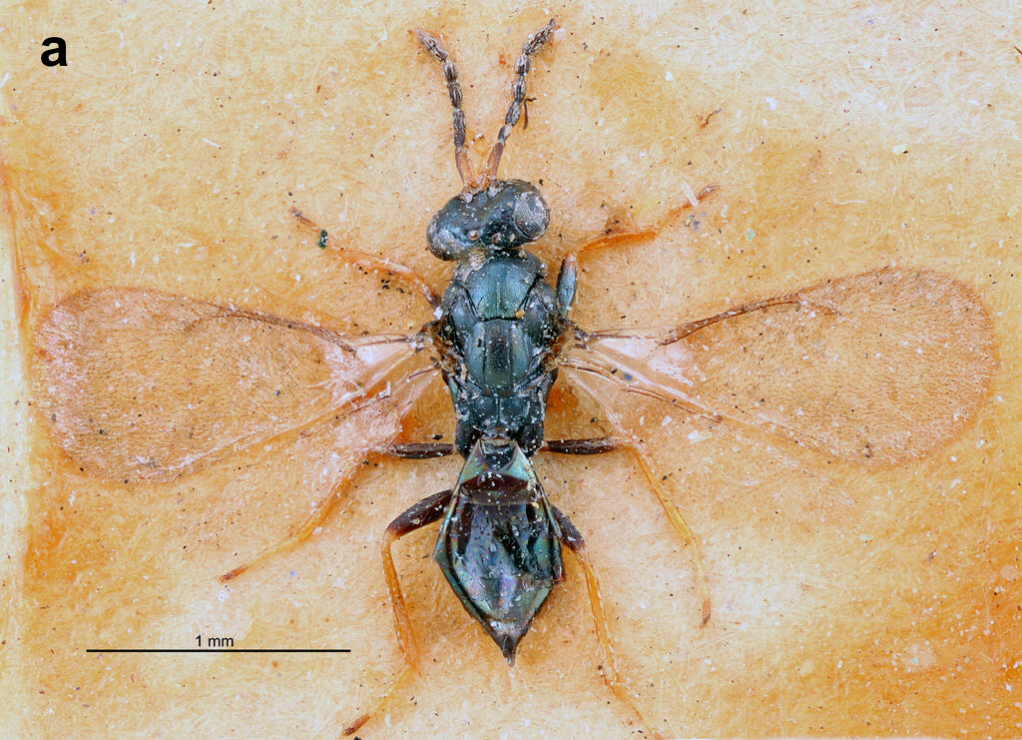
Holotype female, dorsal.

**Figure 66b.**
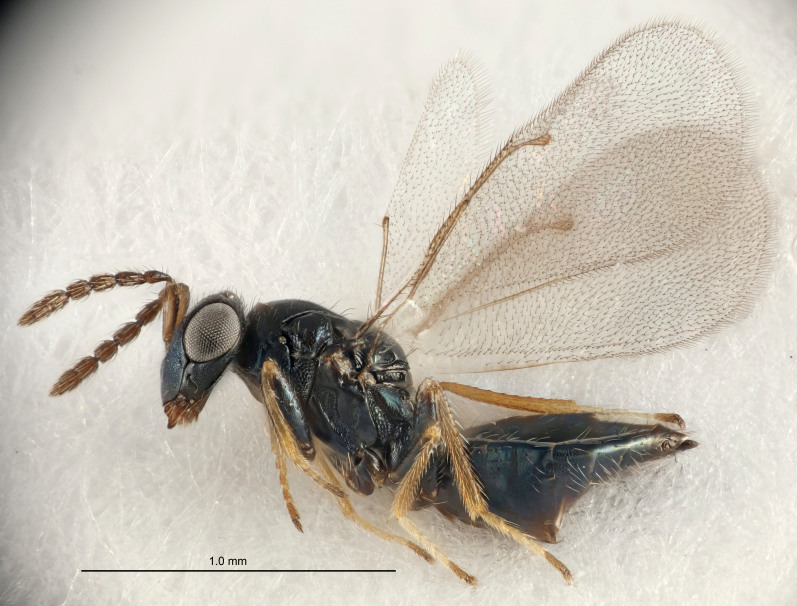
Nontype female, lateral.

**Figure 66c.**
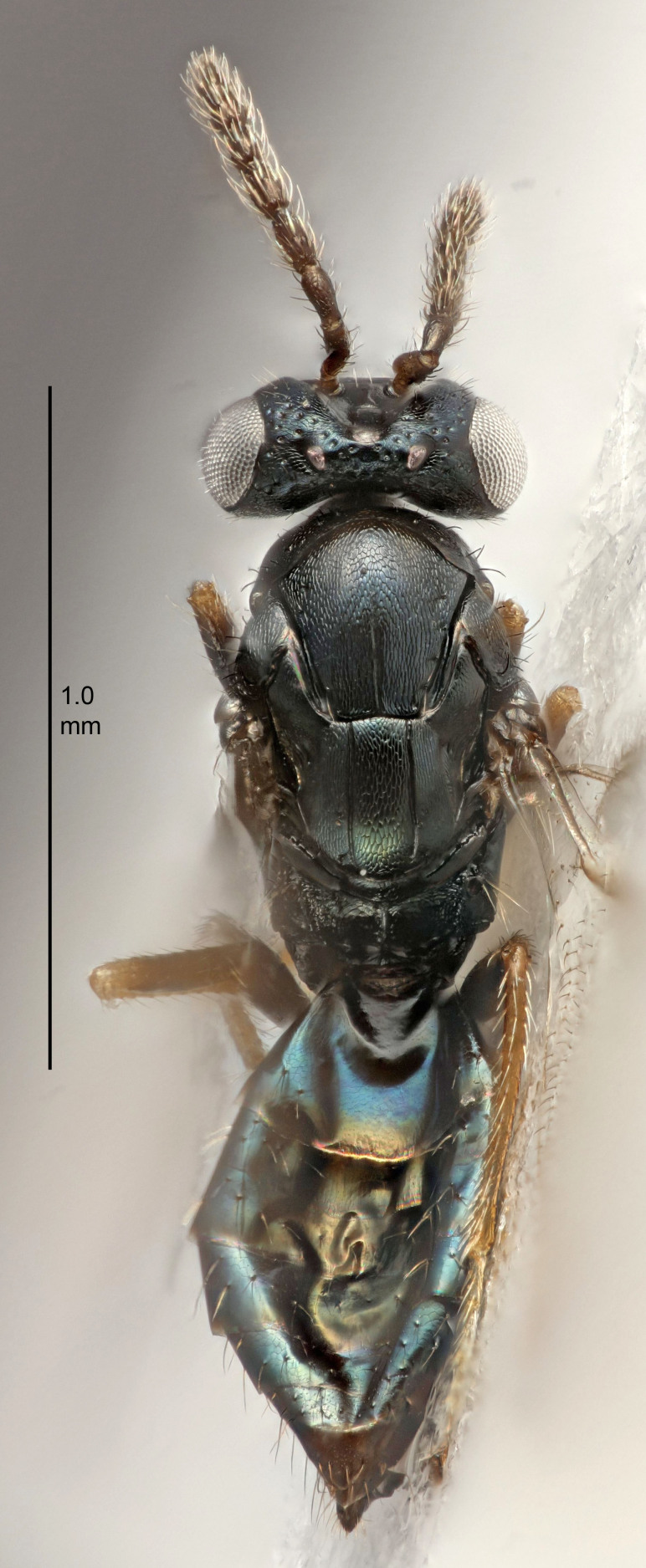
Nontype female, dorsal.

**Figure 66d.**
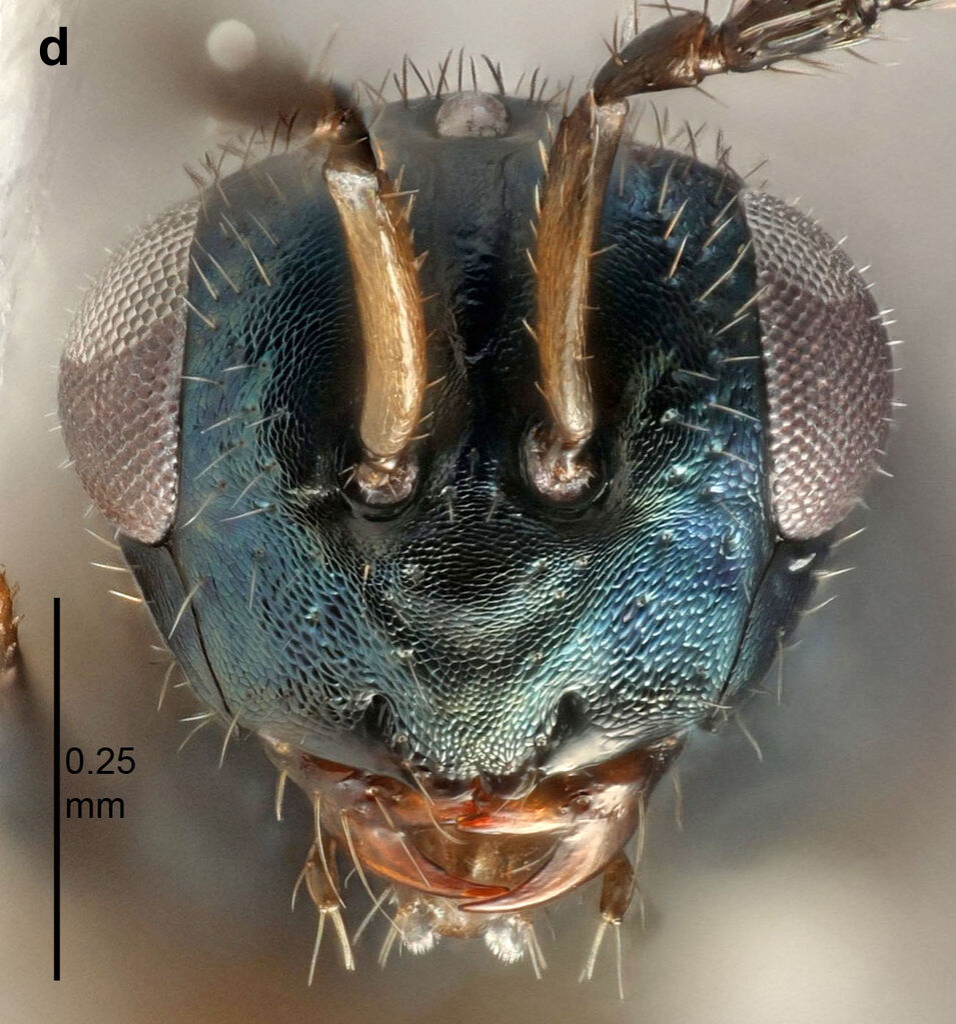
Nontype female, head frontal.

**Figure 66e.**
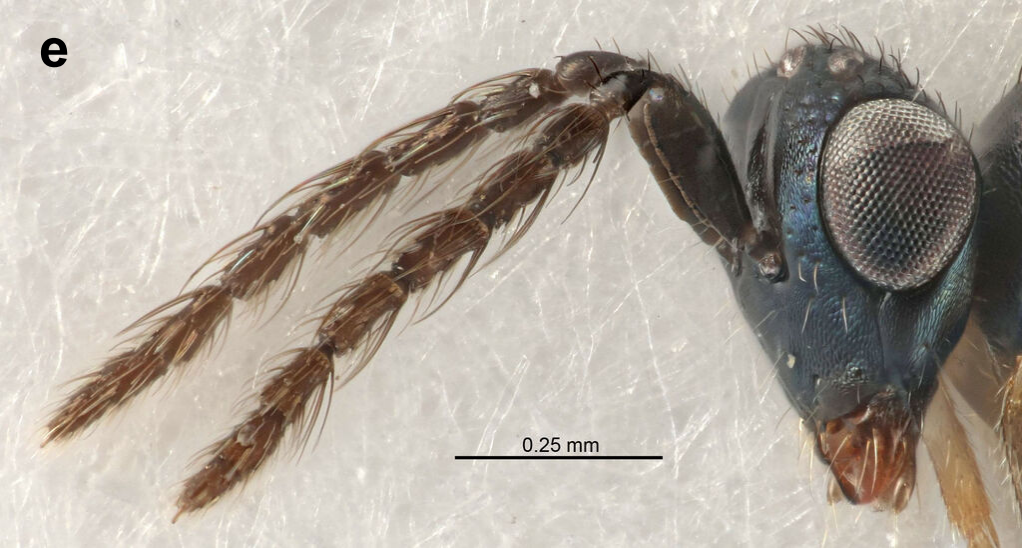
Nontype male, head and antenna lateral.

**Figure 67a.**
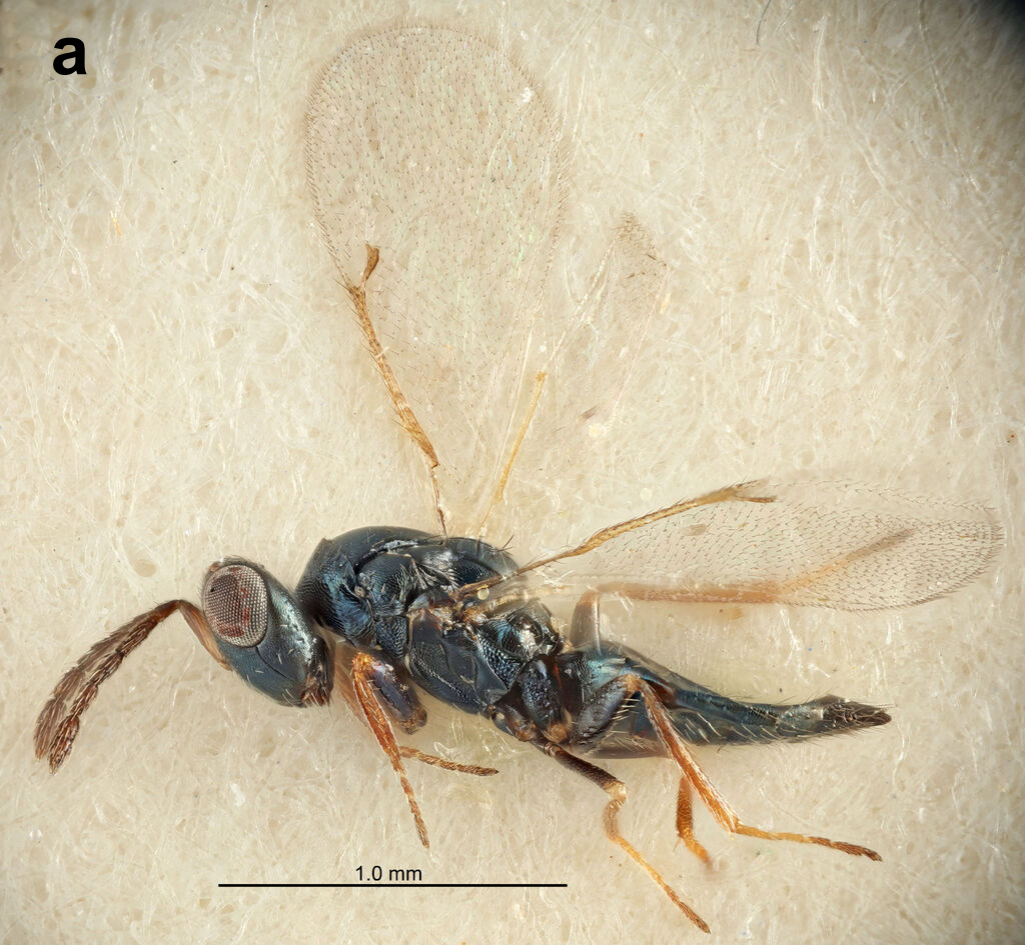
Nontype female, lateral.

**Figure 67b.**
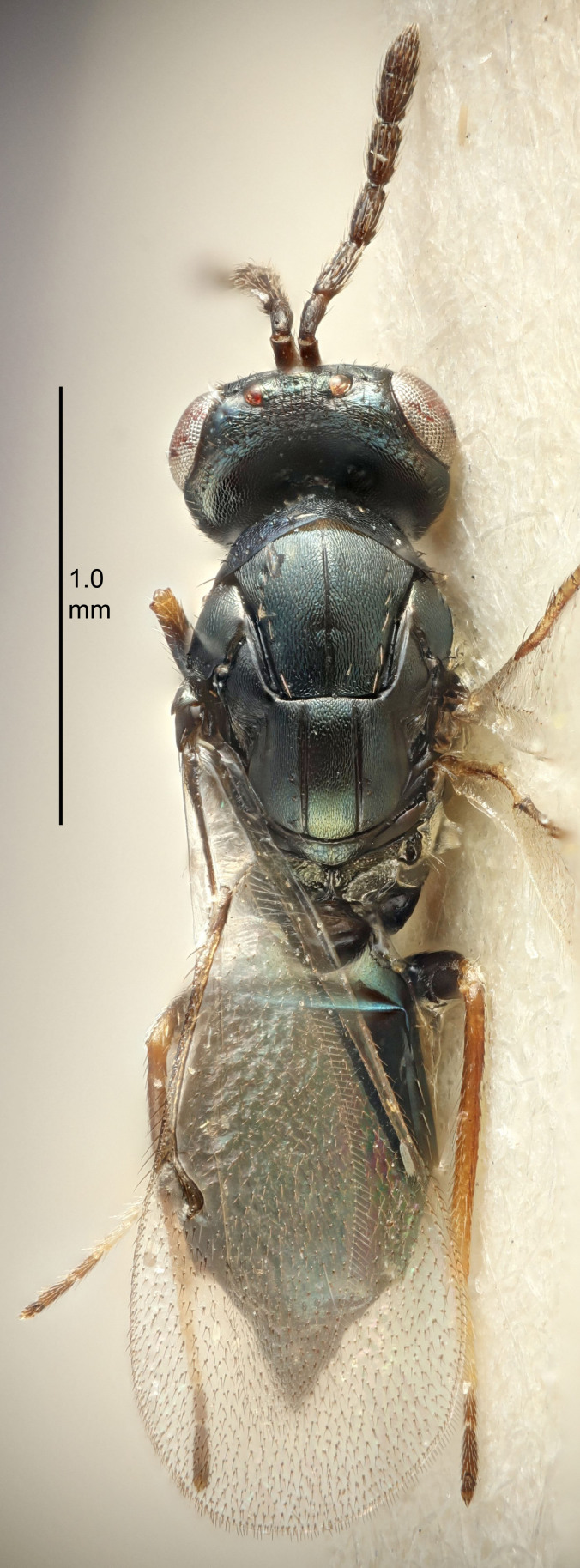
Nontype female, dorsal.

**Figure 67c.**
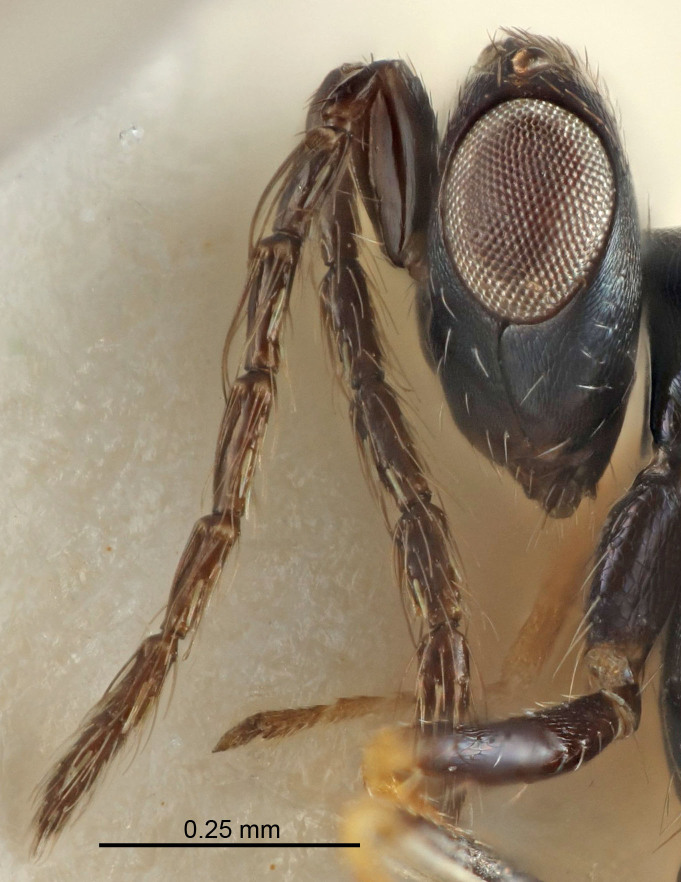
Nontype male, head and antenna lateral.

**Figure 68a.**
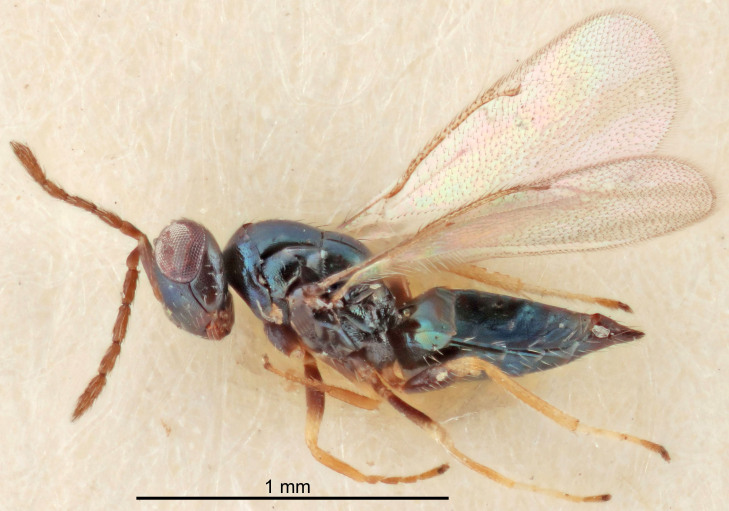
Holotype female, lateral.

**Figure 68b.**
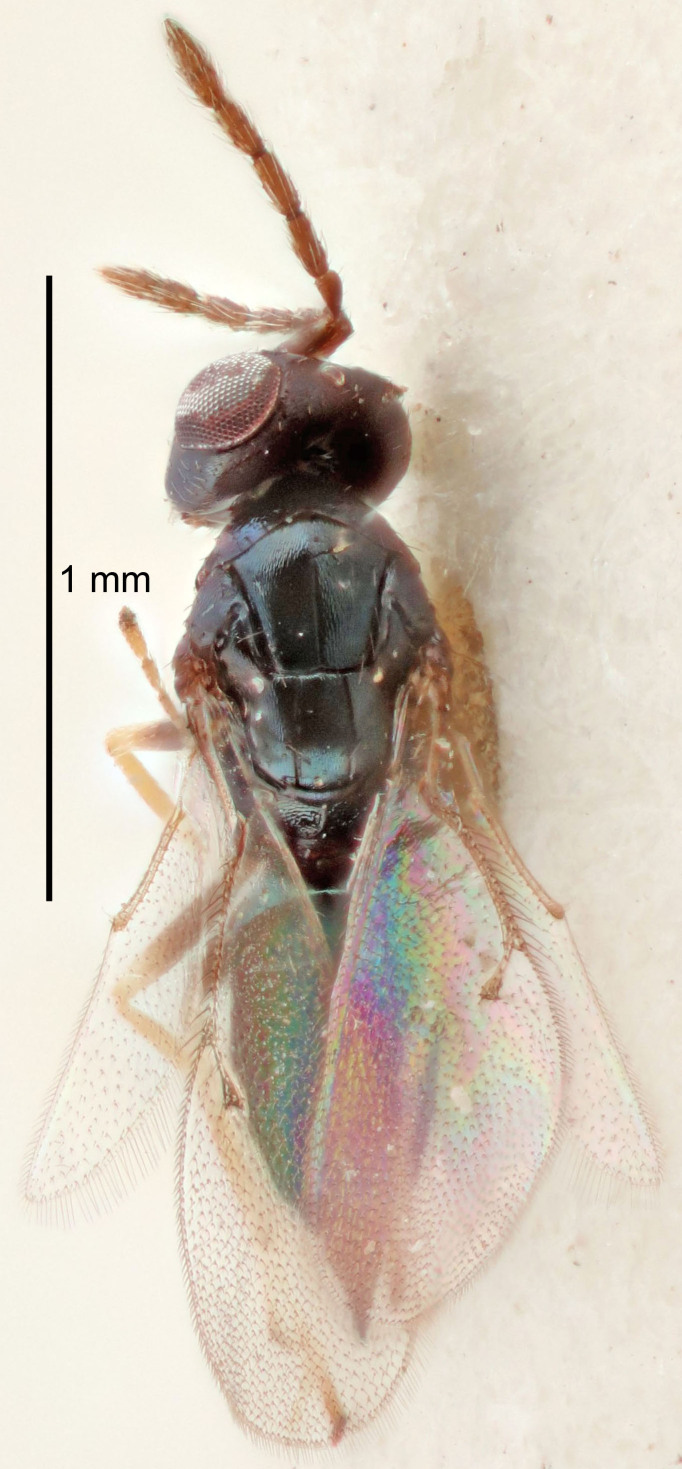
Holotype female, dorsal.

**Figure 69a.**
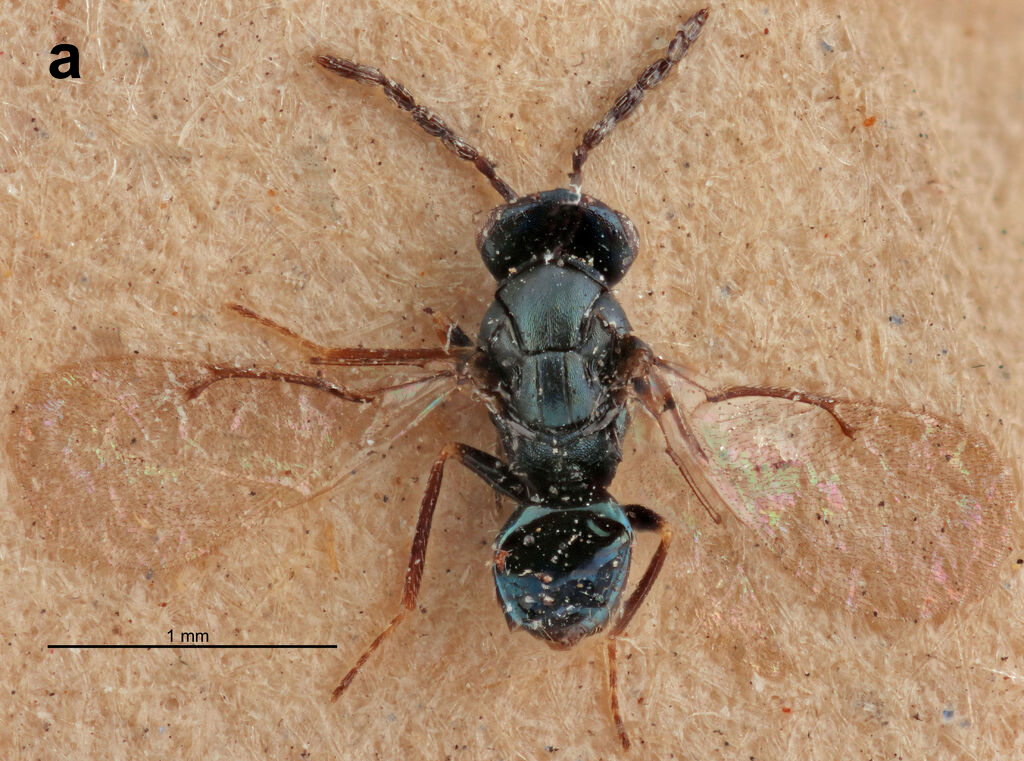
Lectotype female, dorsal.

**Figure 69b.**
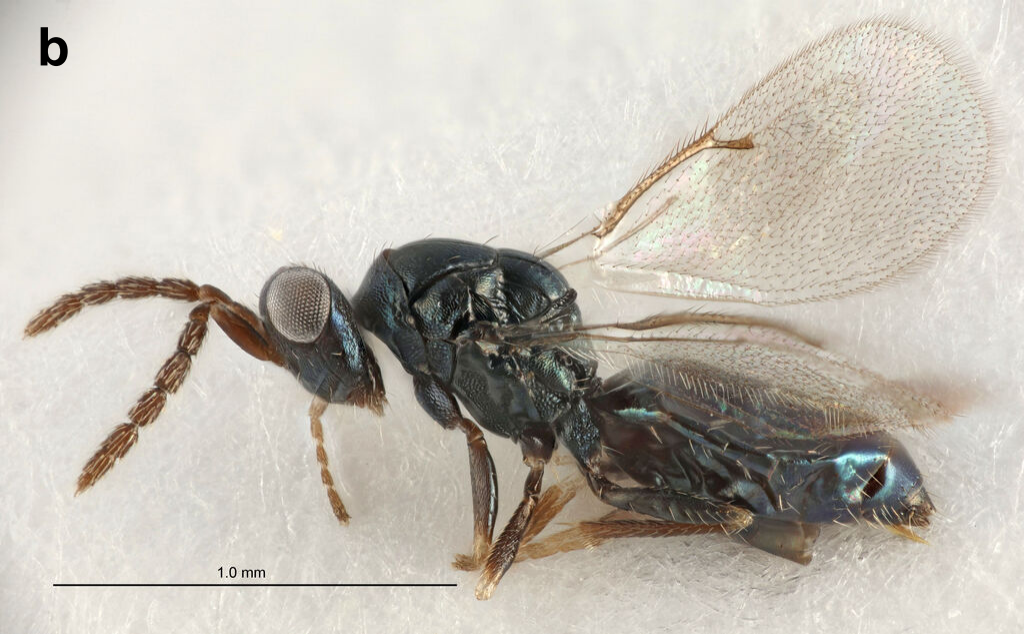
Nontype female, lateral.

**Figure 69c.**
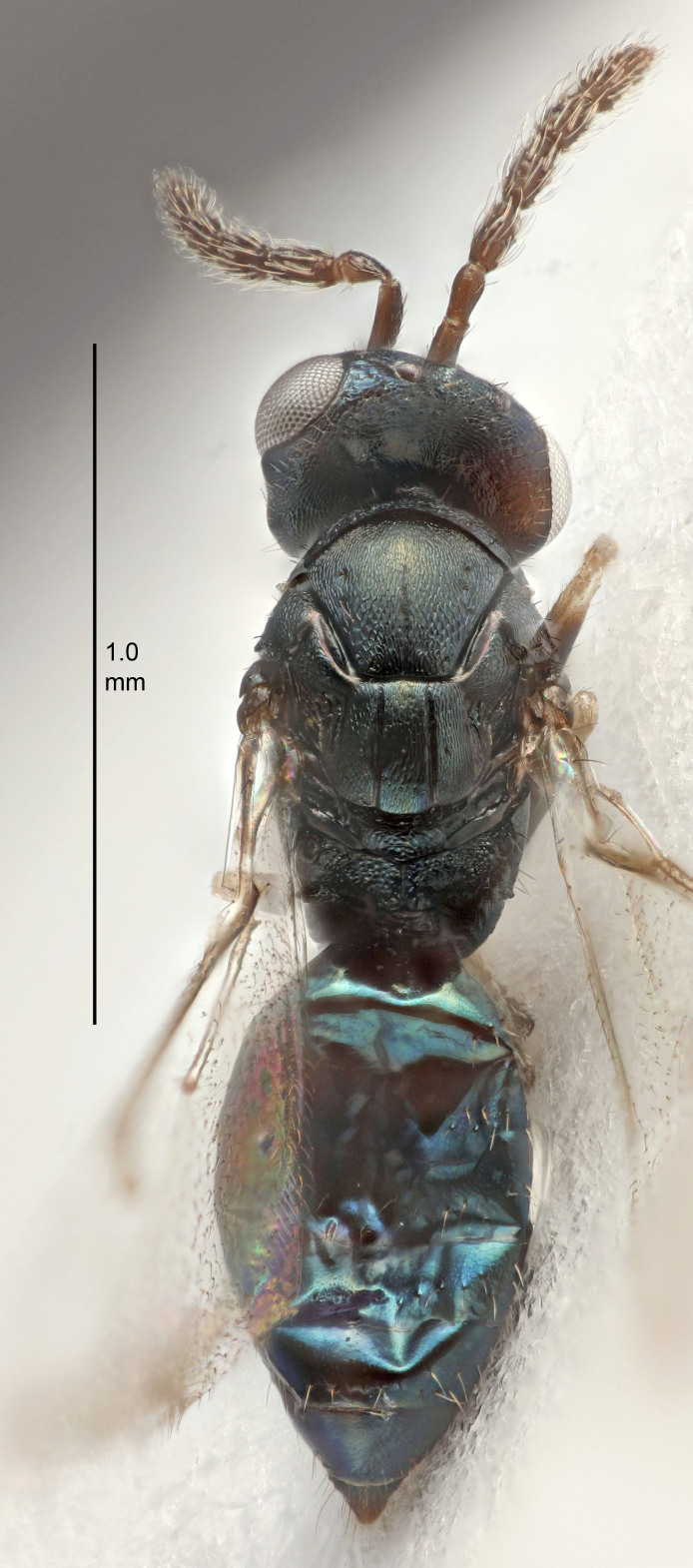
Nontype female, dorsal.

**Figure 69d.**
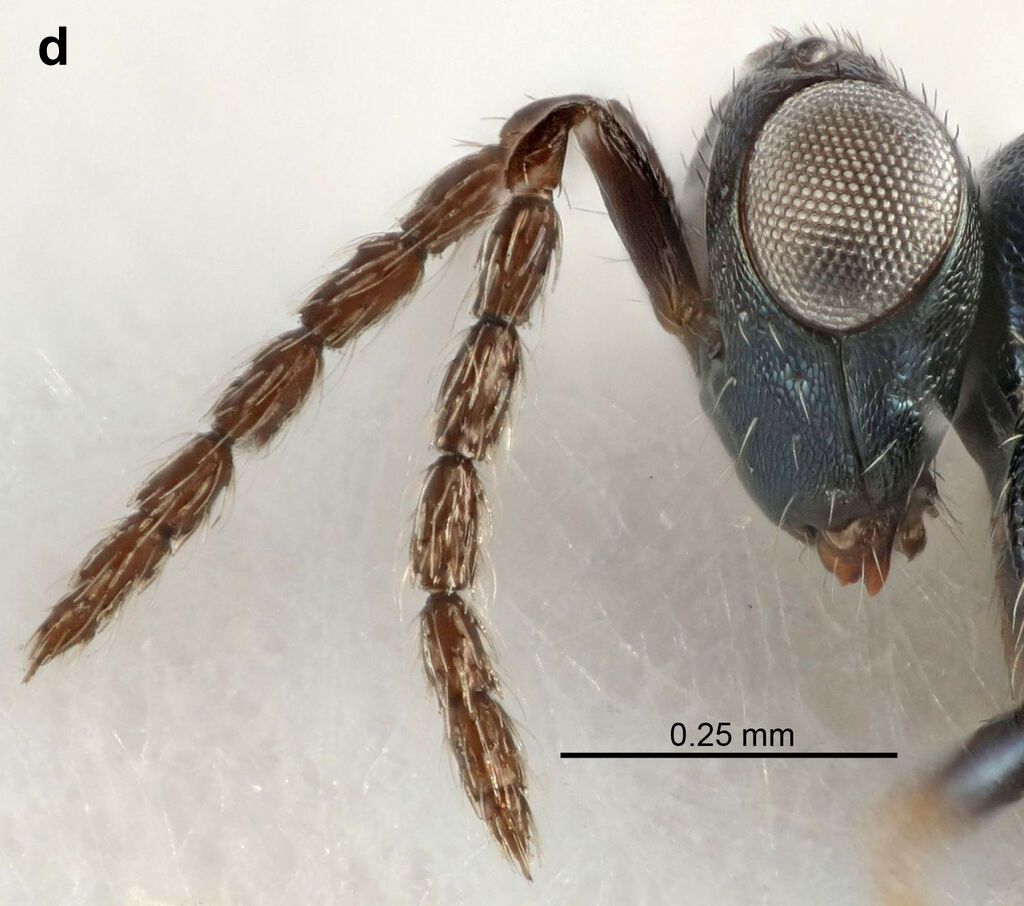
Nontype female, head and antenna lateral.

**Figure 69e.**
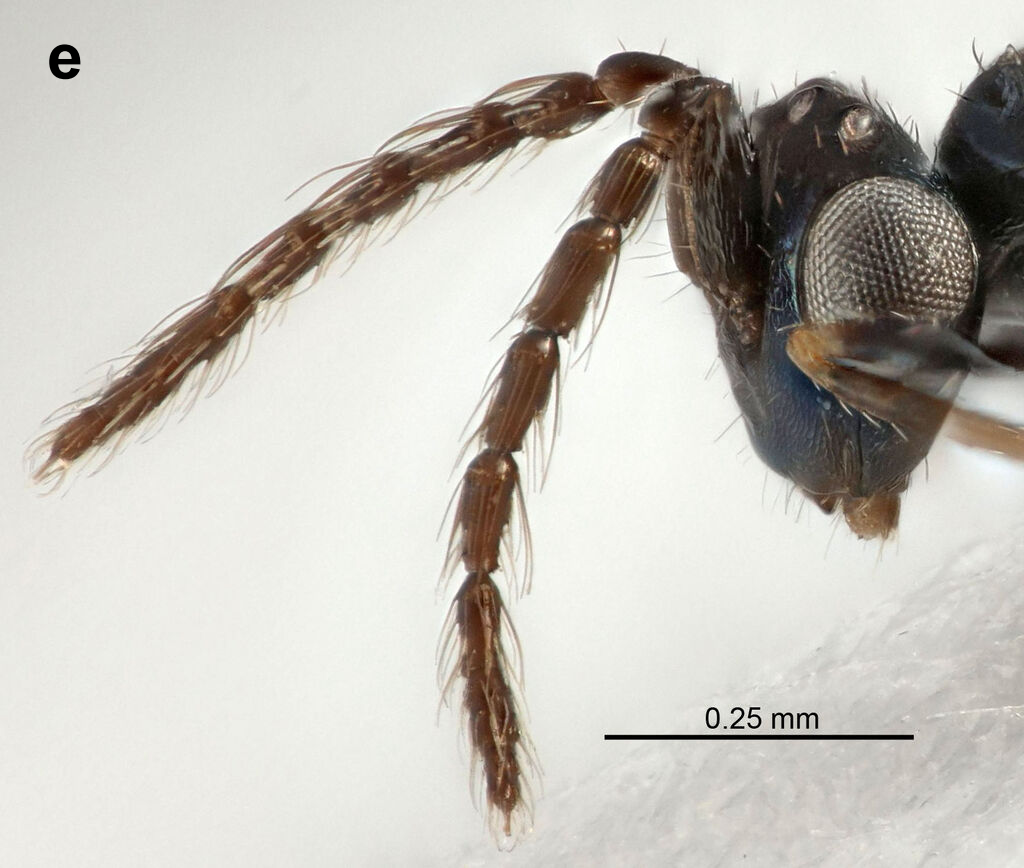
Nontype male, head and antenna lateral.

**Figure 70a.**
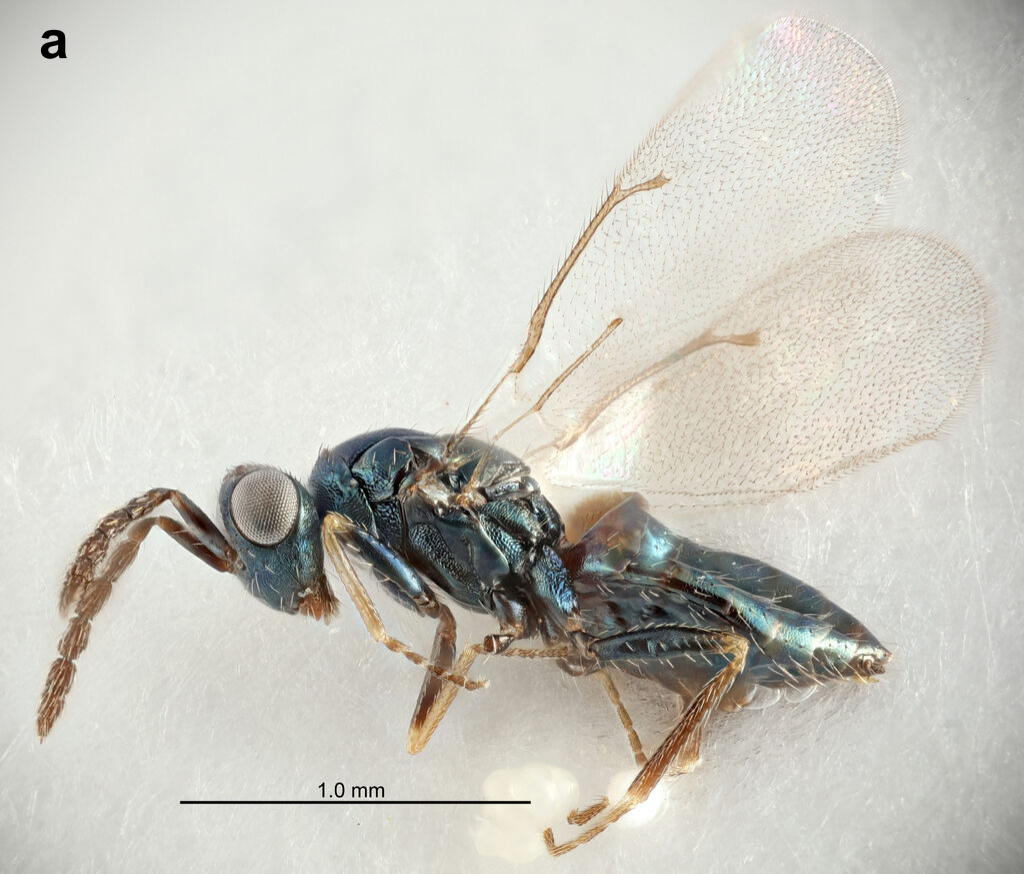
Holotype female, lateral.

**Figure 70b.**
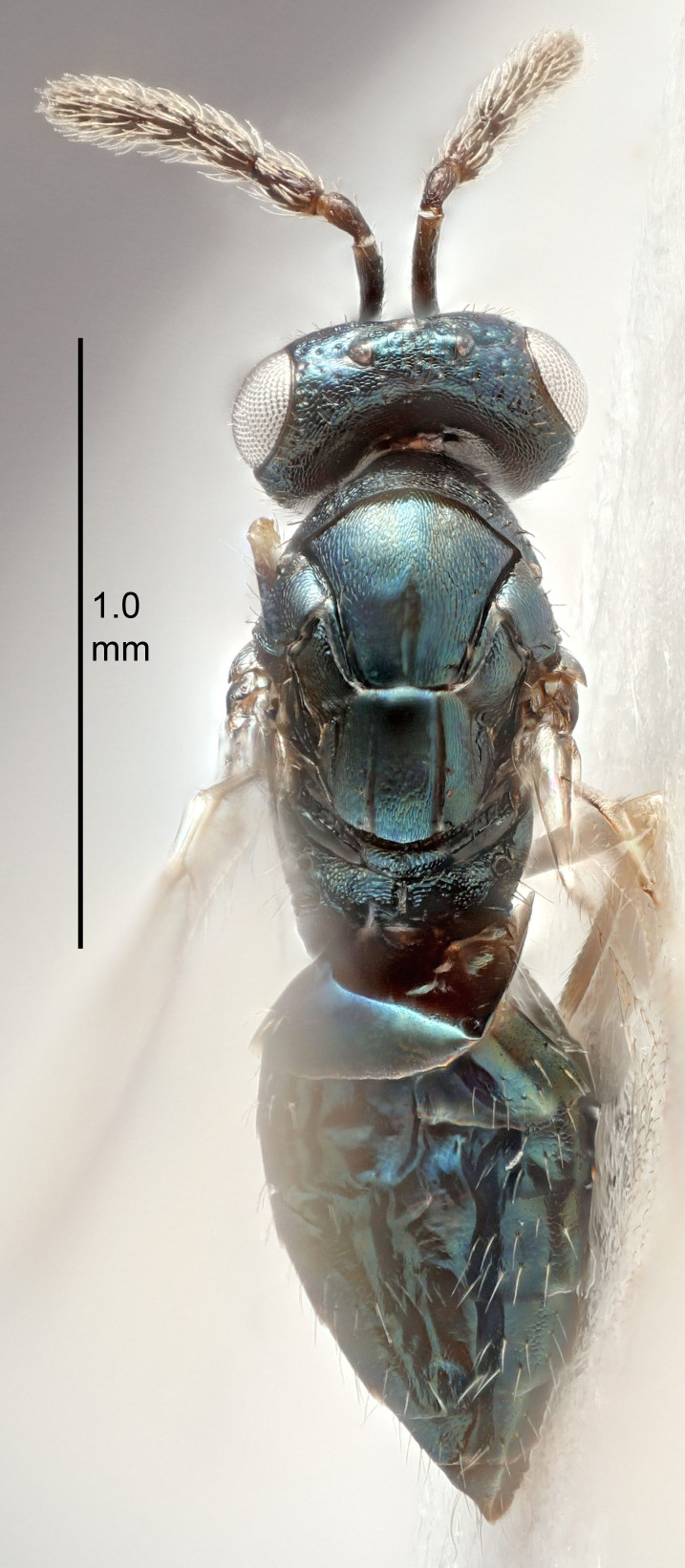
Holotype female, dorsal.

**Figure 70c.**
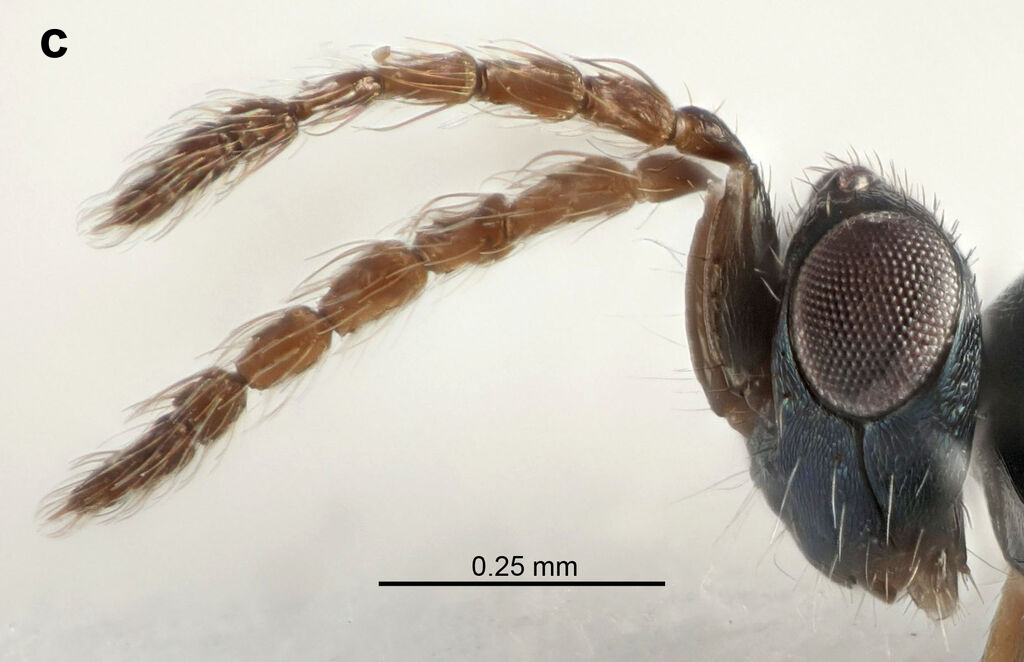
Paratype male, head and antenna lateral

**Figure 71a.**
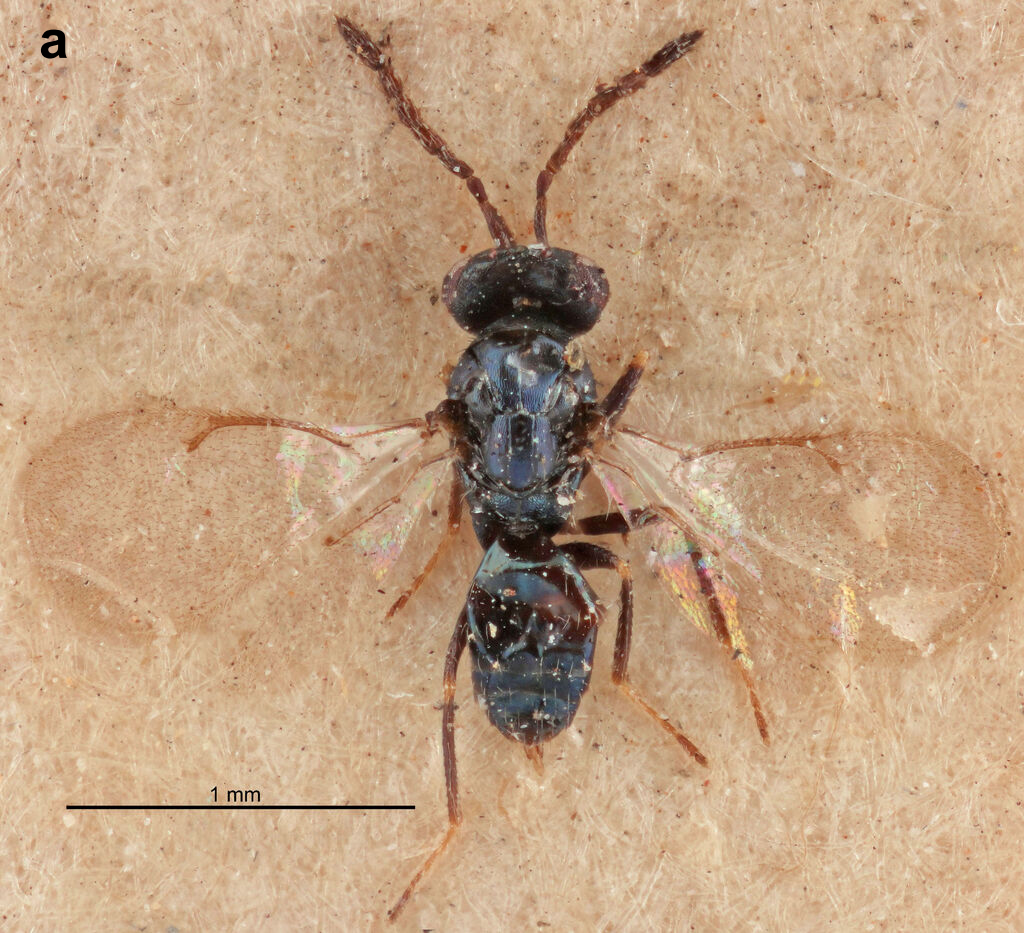
Lectotype male, dorsal.

**Figure 71b.**
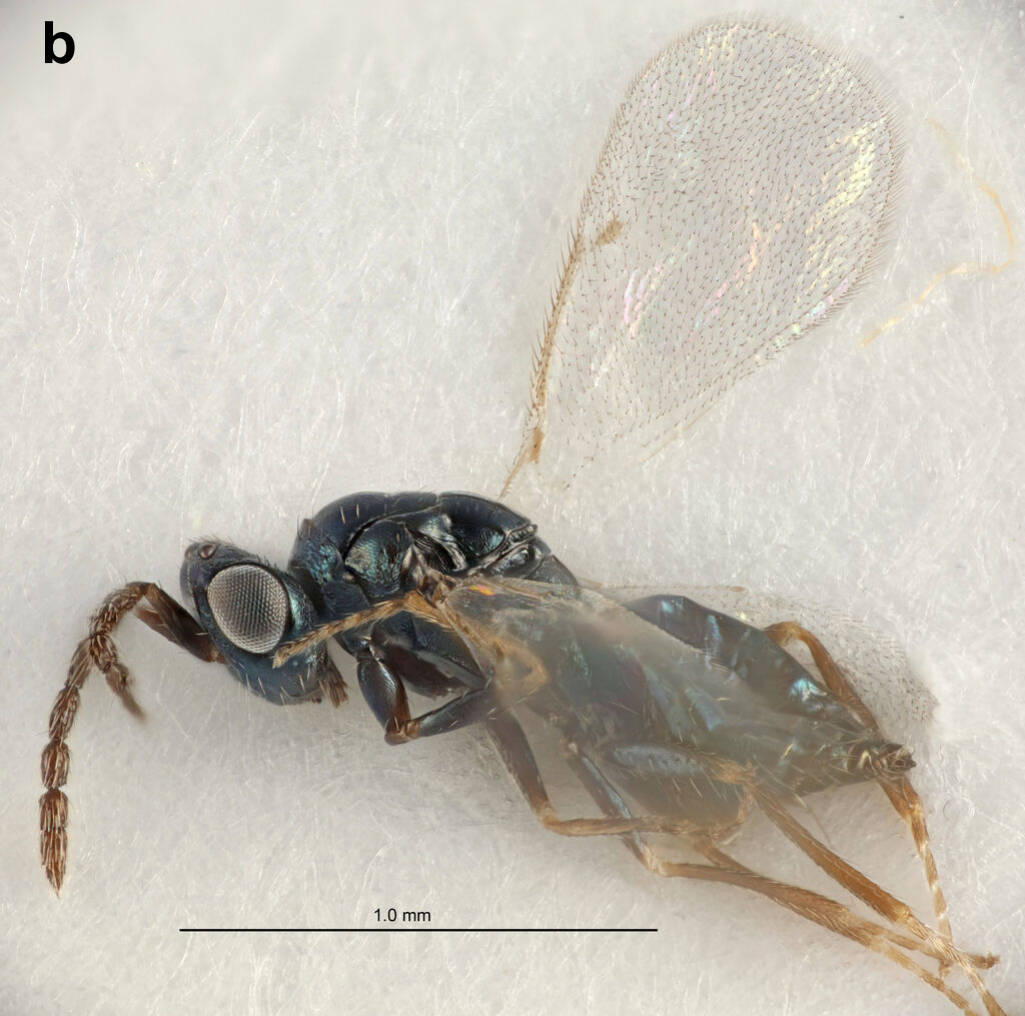
Nontype female, lateral.

**Figure 71c.**
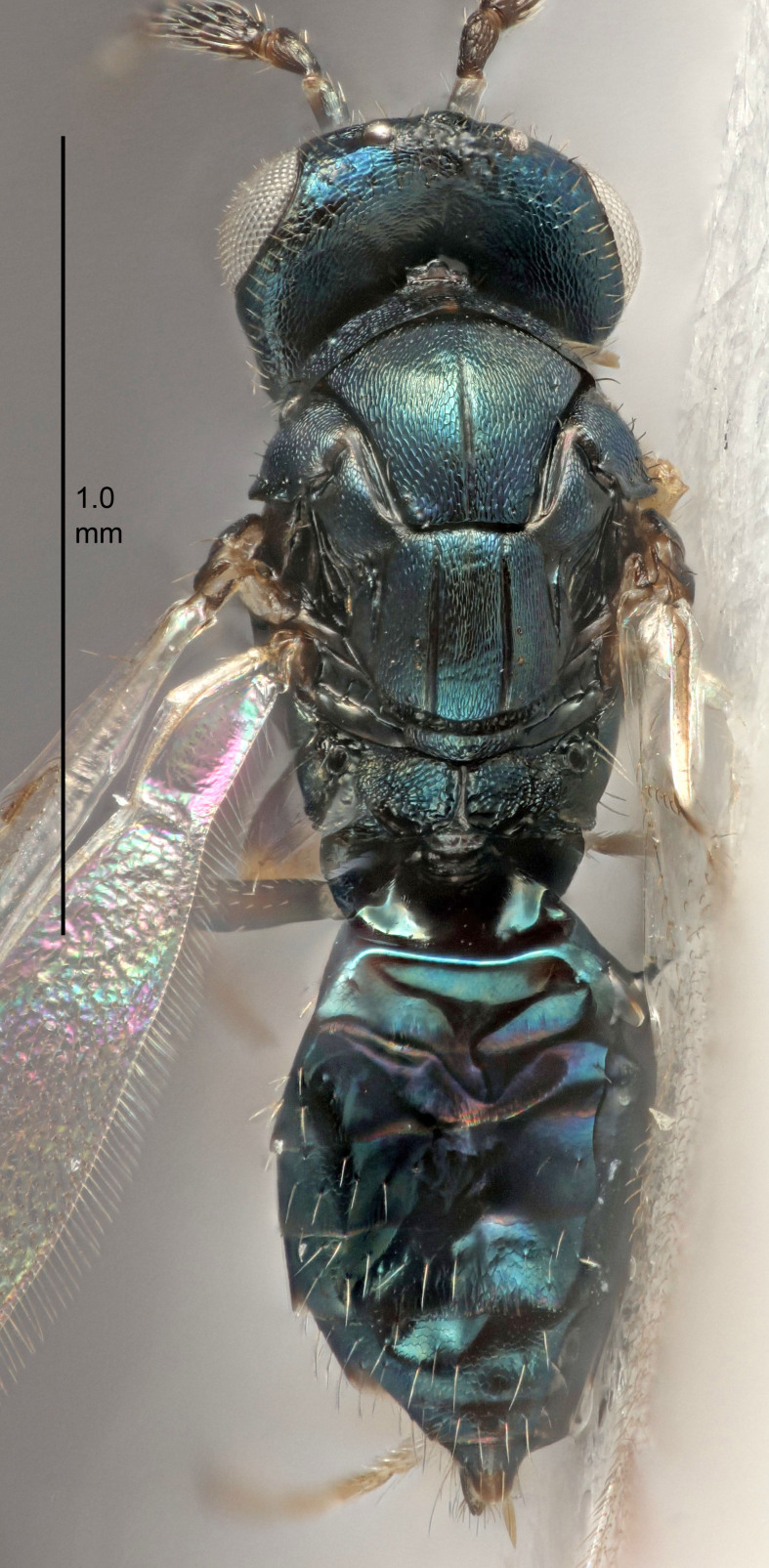
Nontype female, dorsal.

**Figure 71d.**
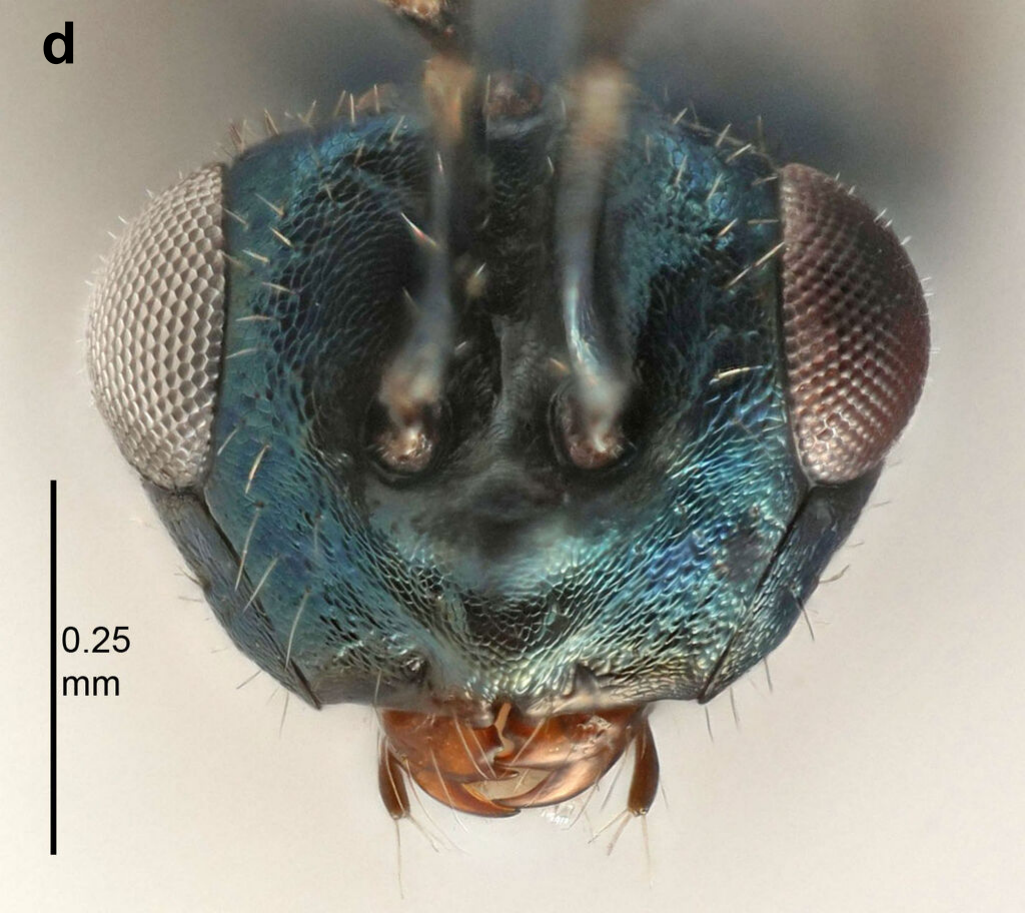
Nontype female, head and antenna lateral.

**Figure 71e.**
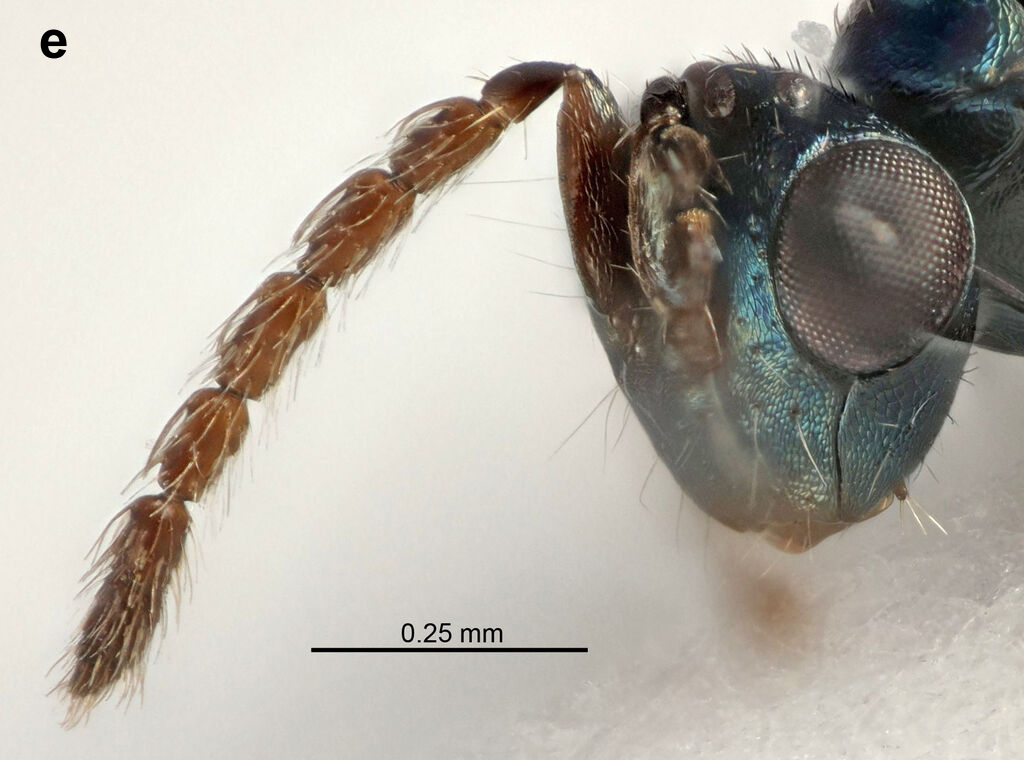
Nontype male, head and antenna lateral.

**Figure 72a.**
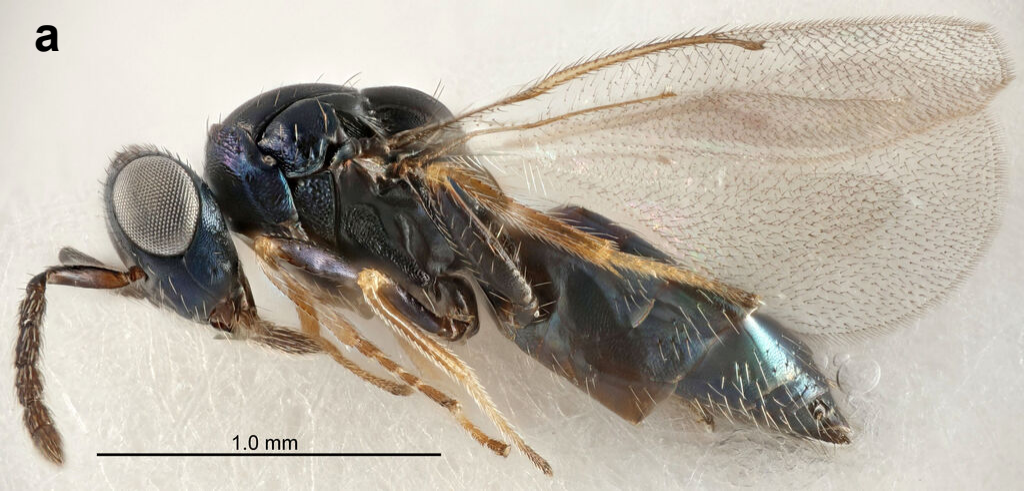
Holotype female, lateral.

**Figure 72b.**
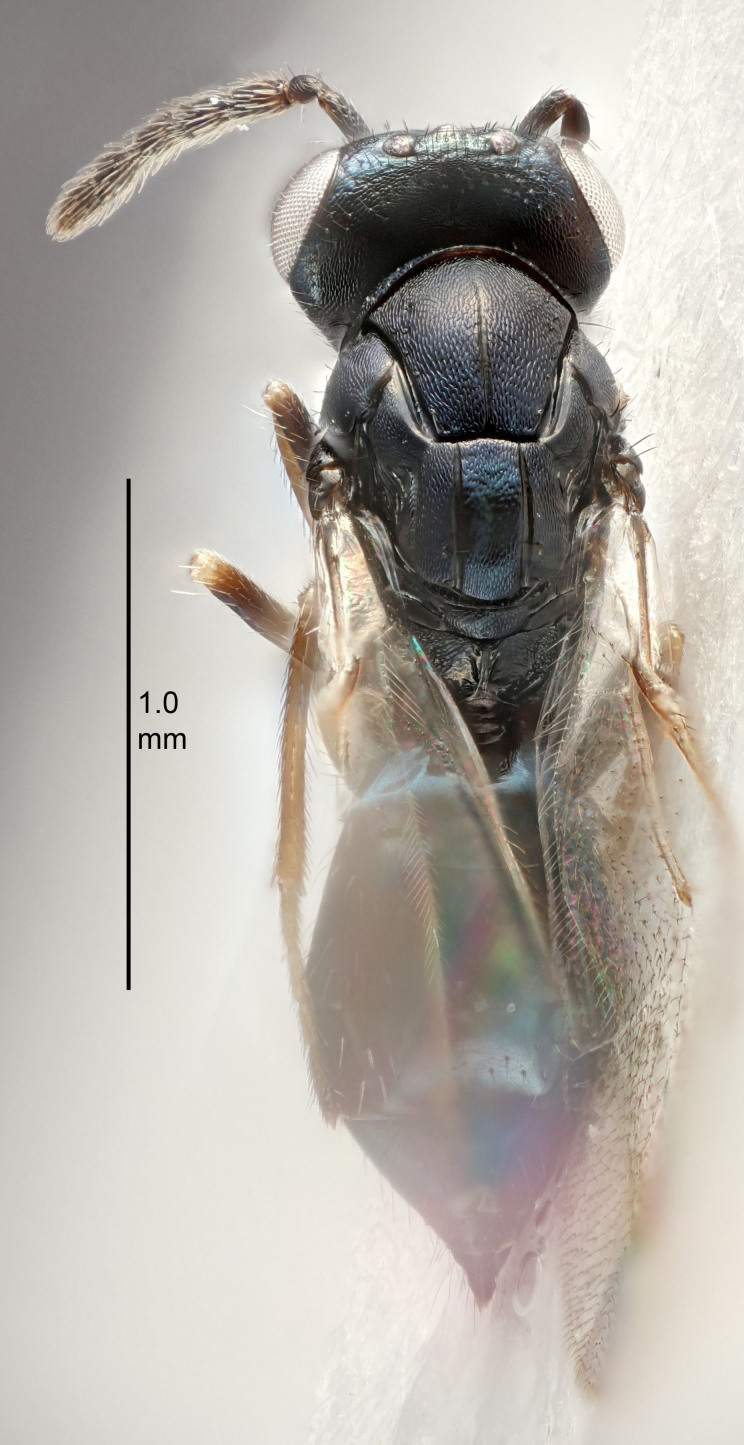
Holotype female, dorsal.

**Figure 73a.**
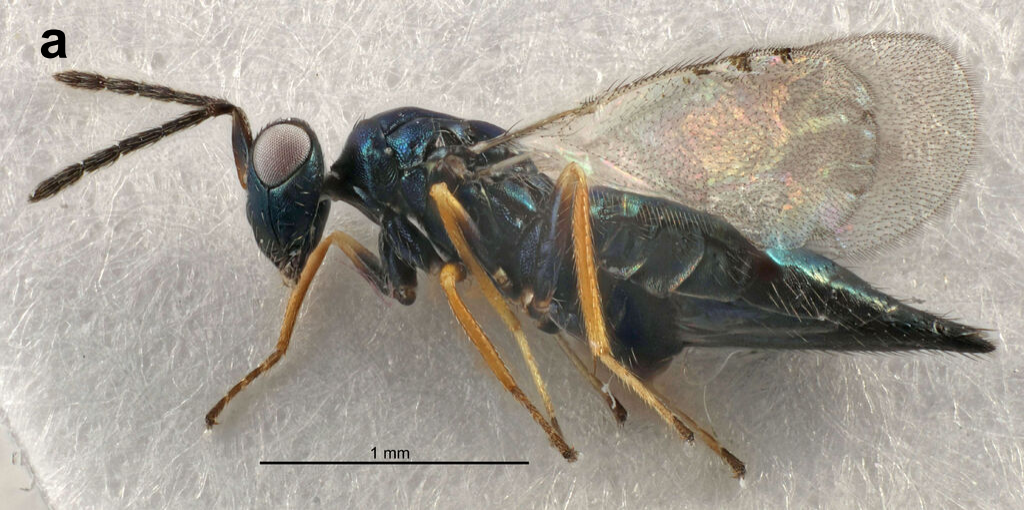
Nontype female, lateral.

**Figure 73b.**
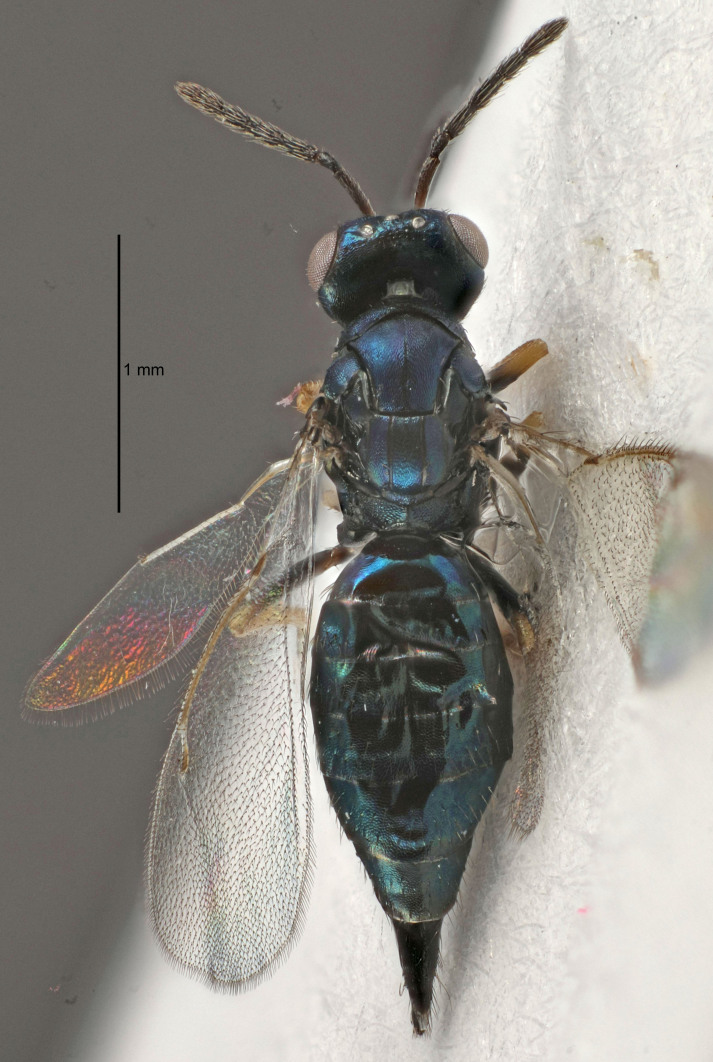
Nontype female, dorsal.

**Figure 73c.**
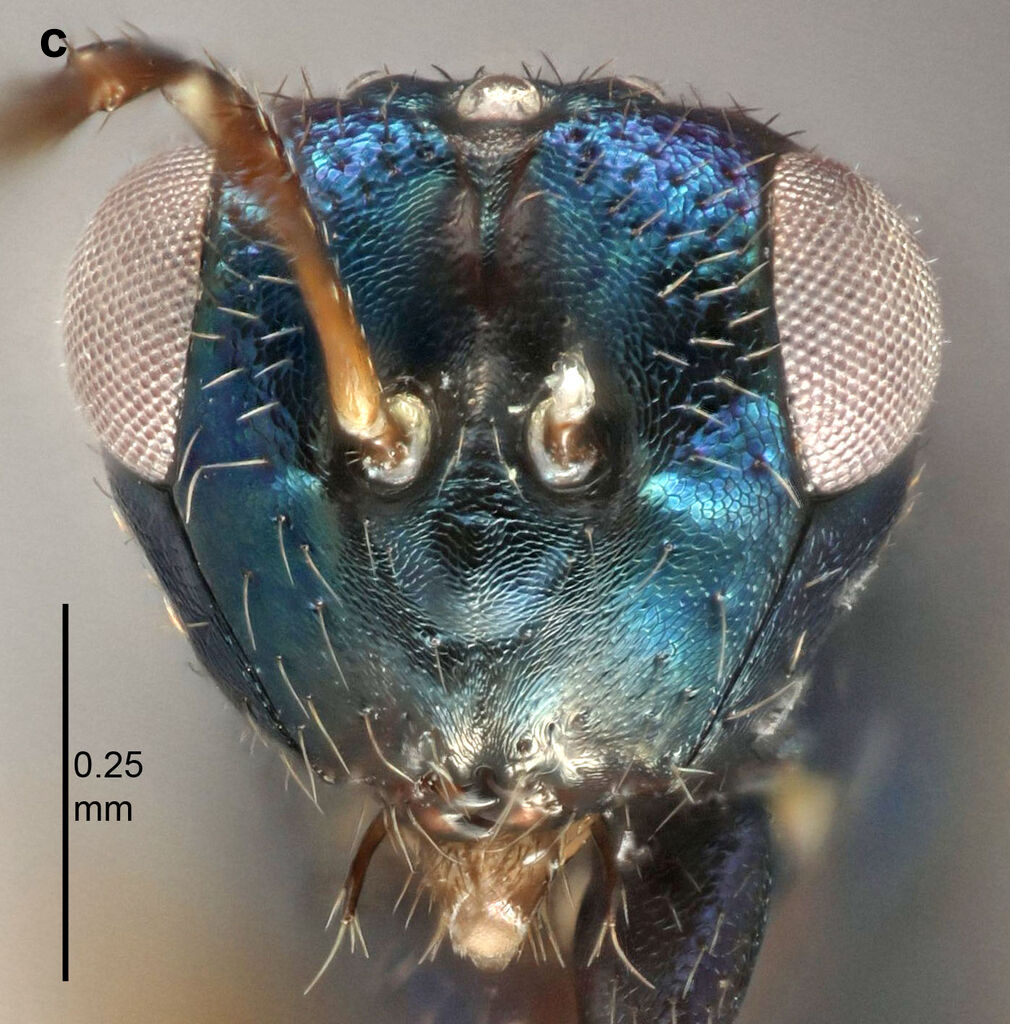
Nontype female, head frontal.

**Figure 73d.**
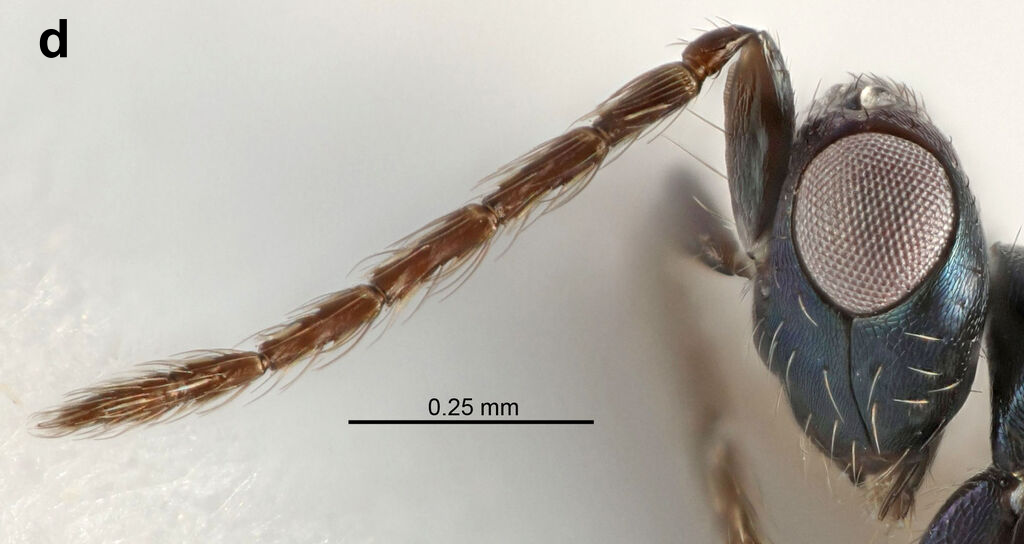
Nontype male, head and antenna lateral.

**Figure 74a.**
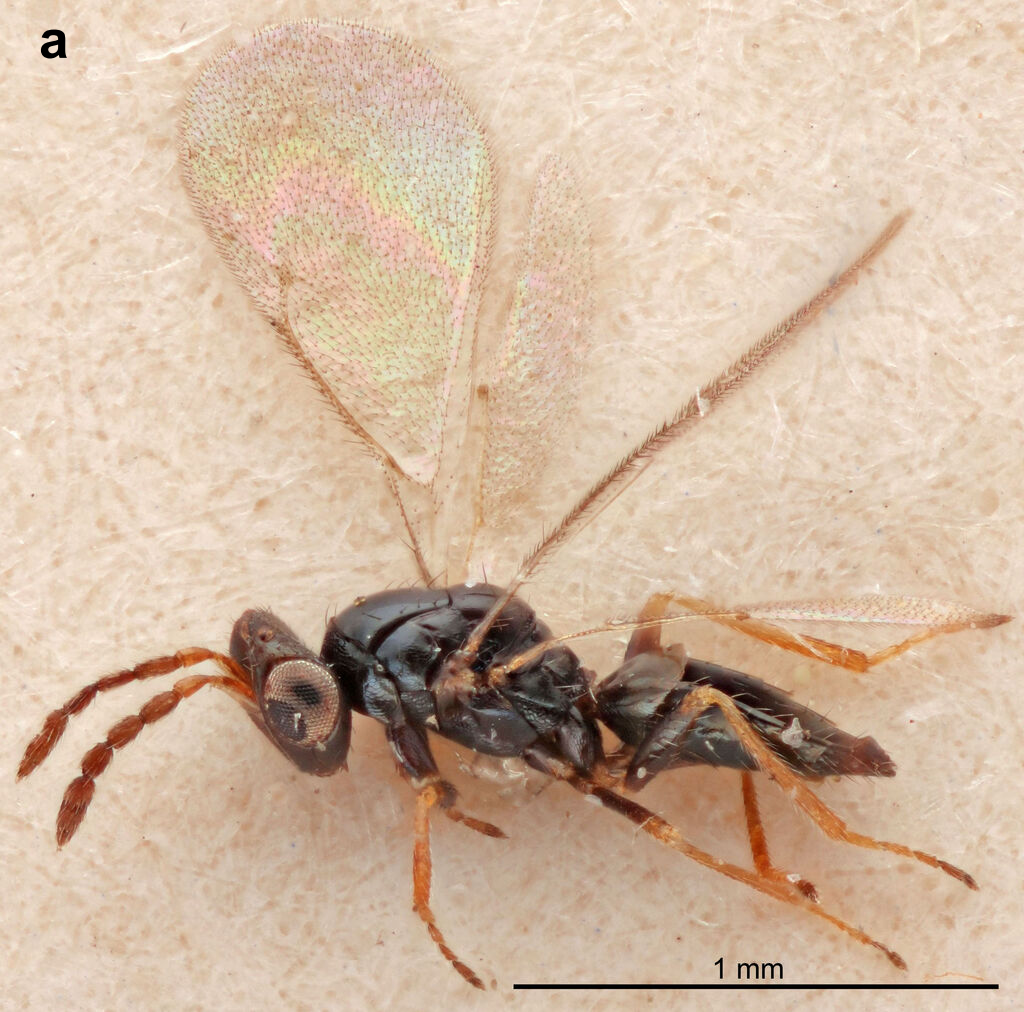
Holotype female, lateral.

**Figure 74b.**
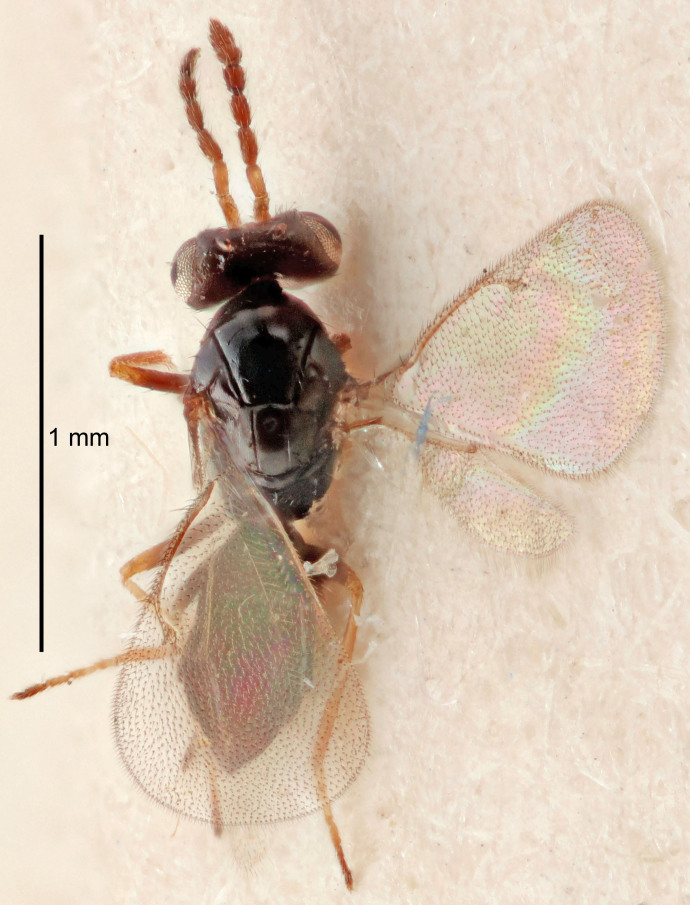
Holotype female, dorsal.

**Figure 75a.**
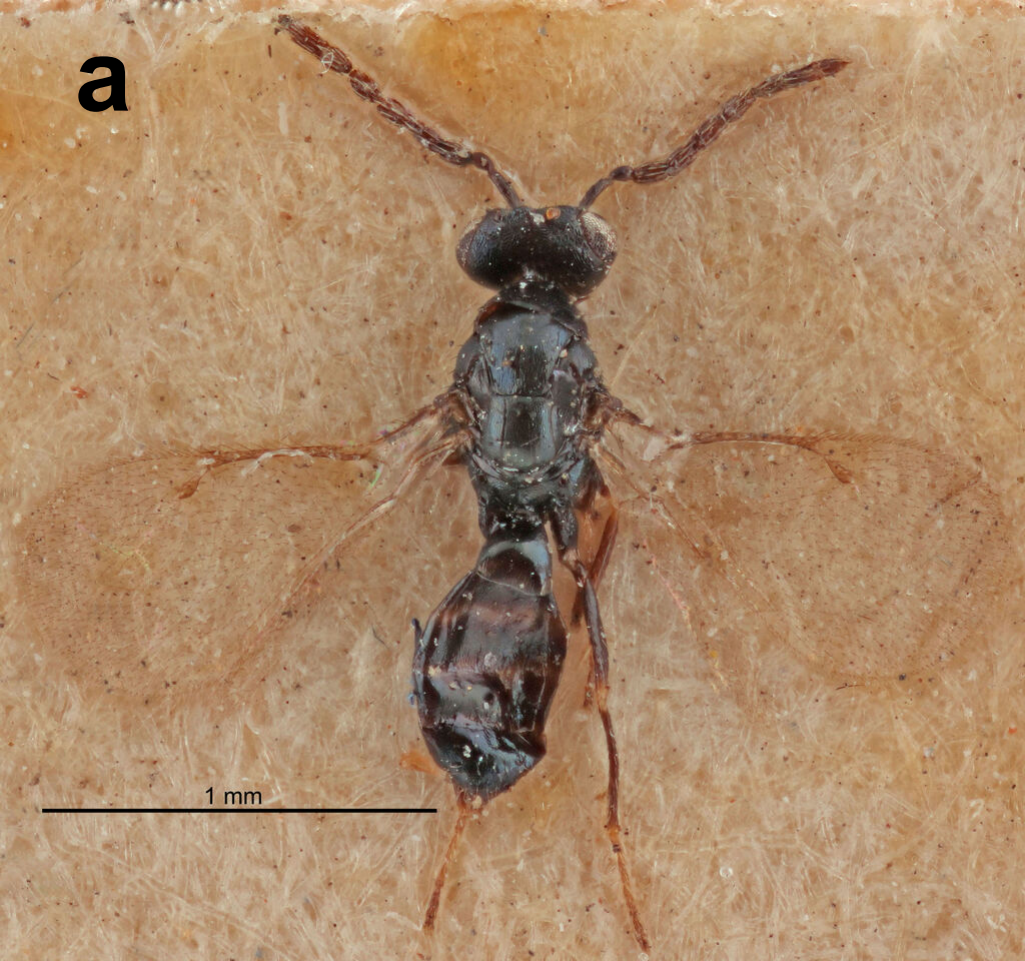
Lectotype male, dorsal.

**Figure 75b.**
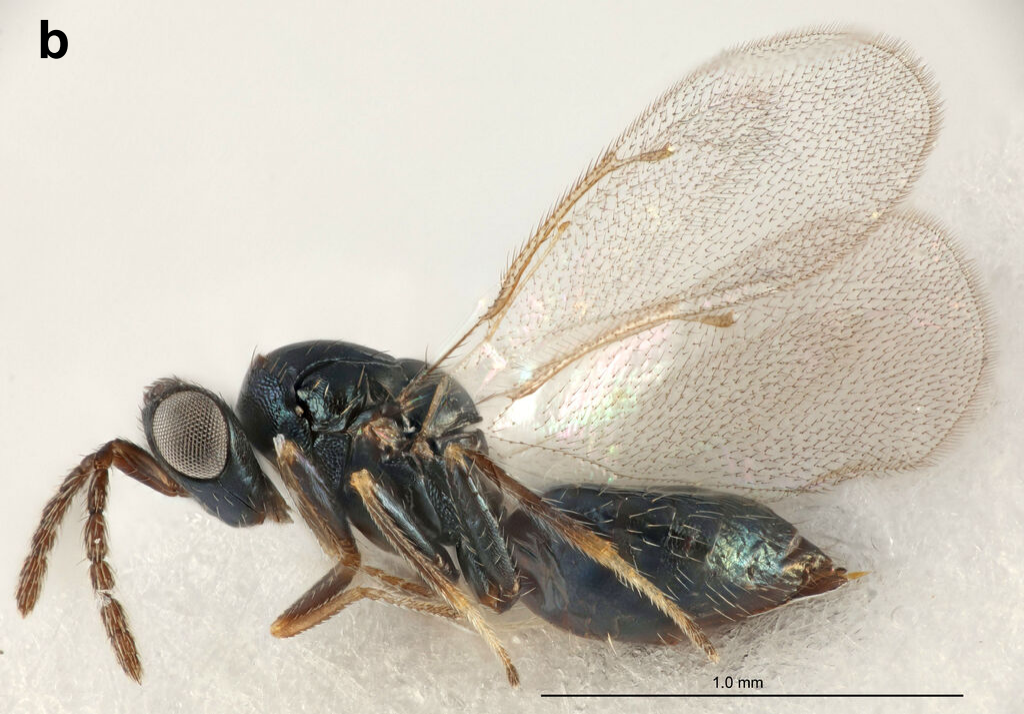
Nontype female, lateral.

**Figure 75c.**
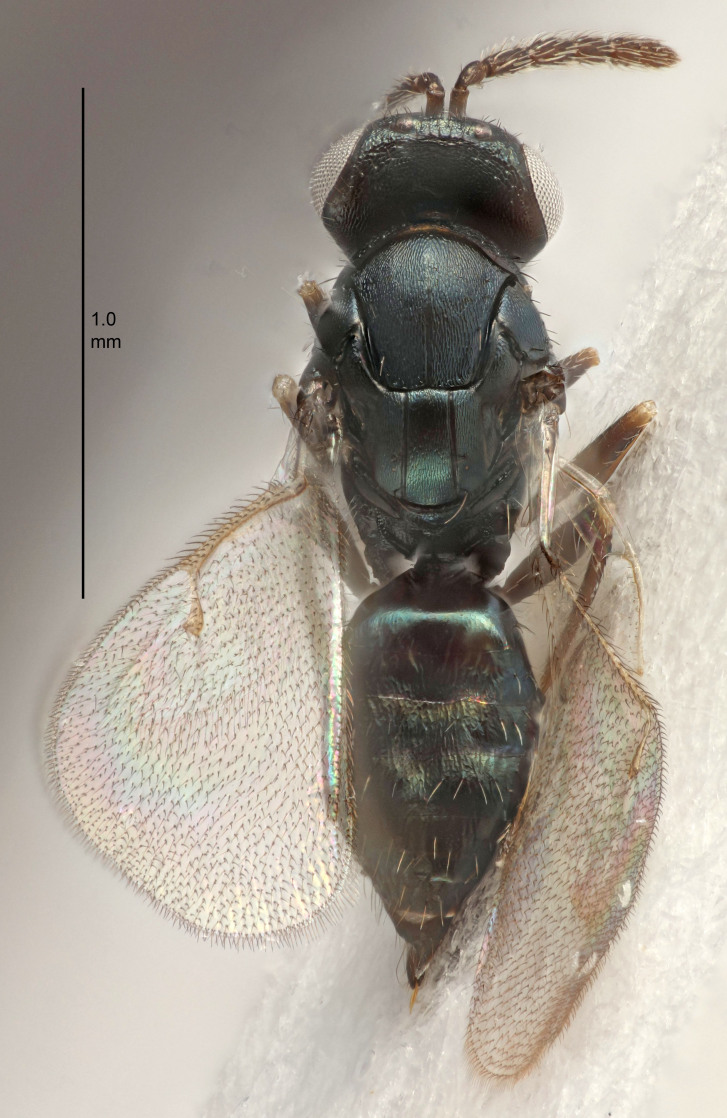
Nontype female, dorsal.

**Figure 75d.**
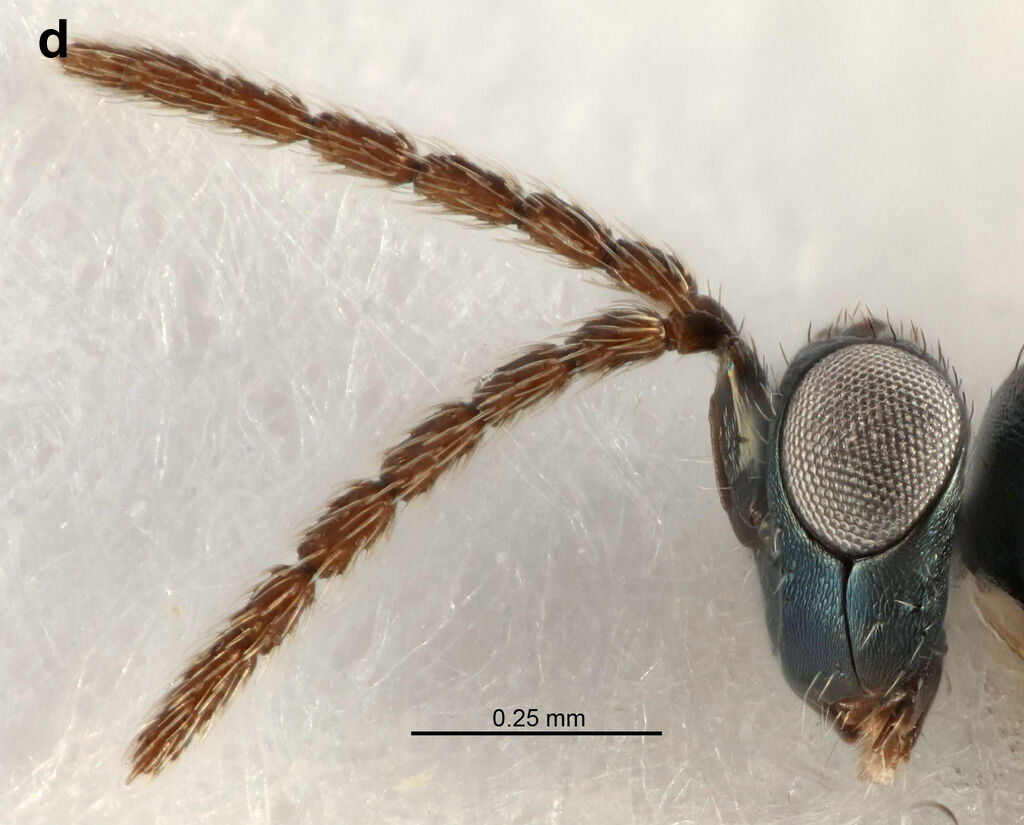
Nontype male, head and antenna lateral.

**Figure 76a.**
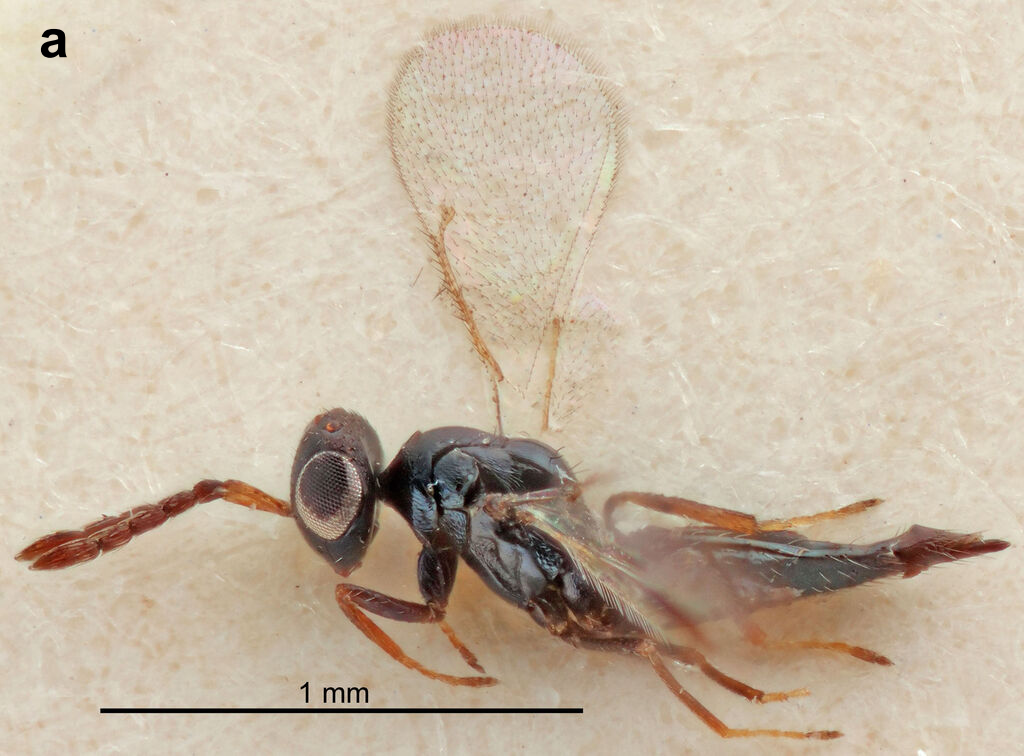
Holotype female, lateral.

**Figure 76b.**
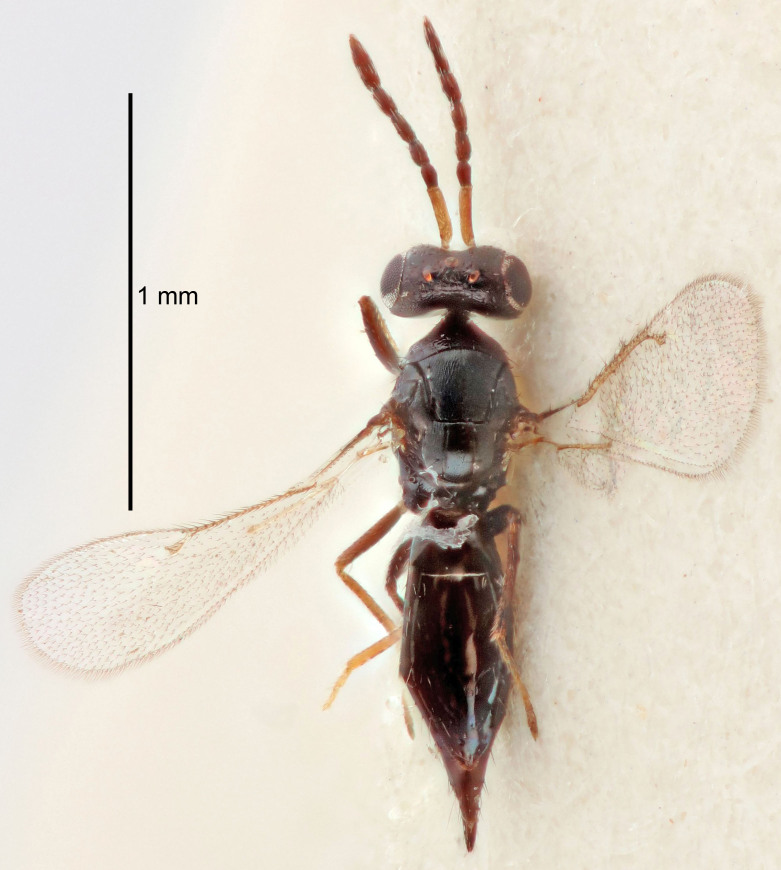
Holotype female, dorsal.

**Figure 77a.**
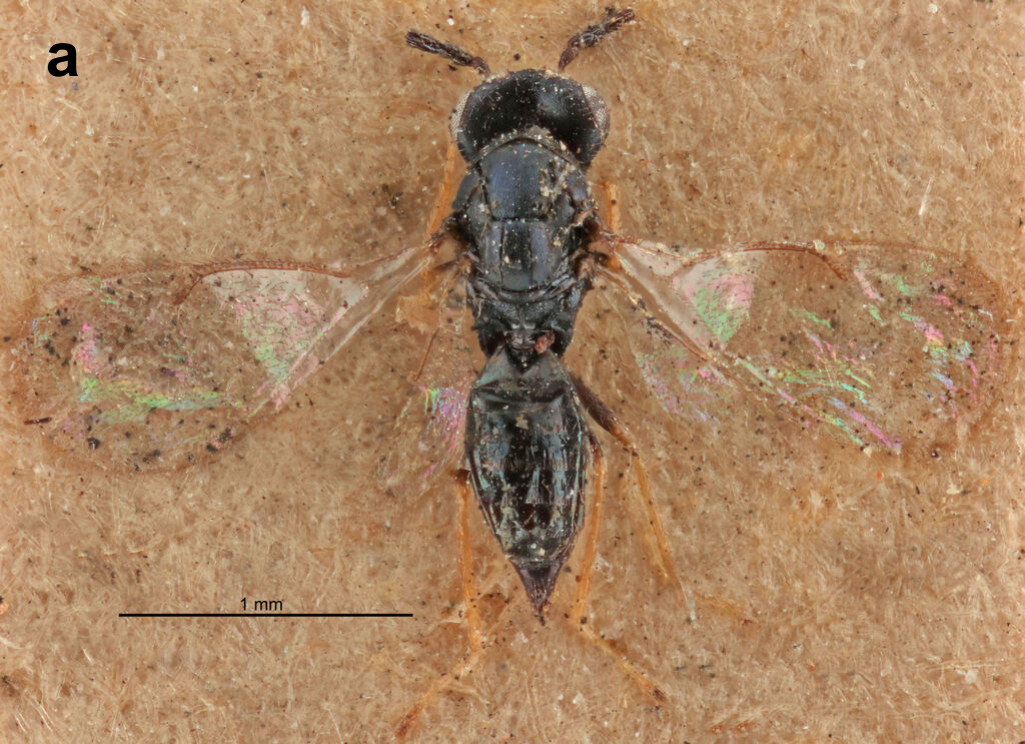
Lectotype female, dorsal.

**Figure 77b.**
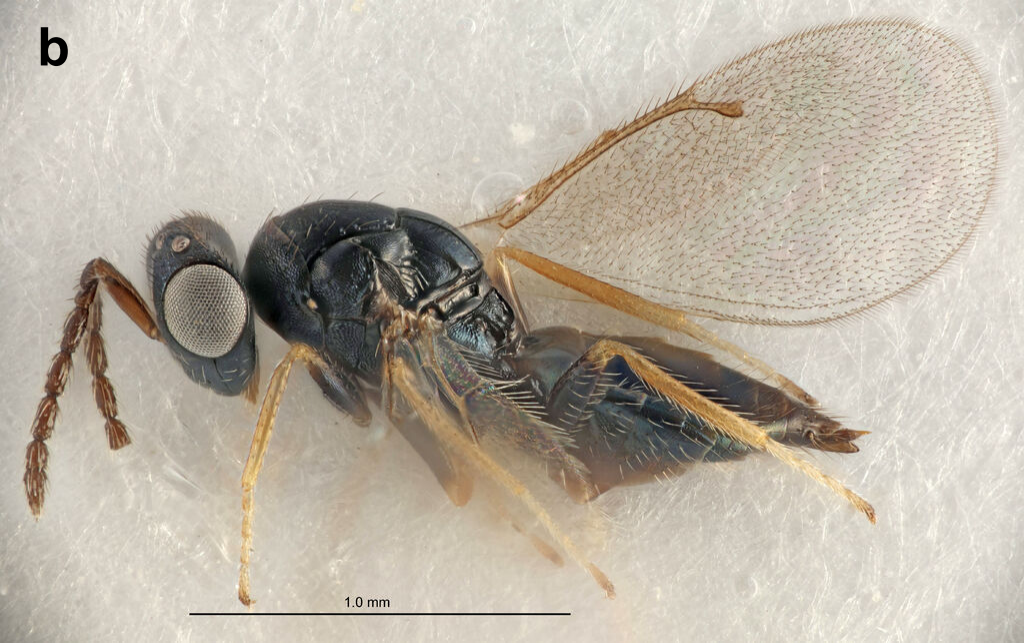
Nontype female, lateral.

**Figure 77c.**
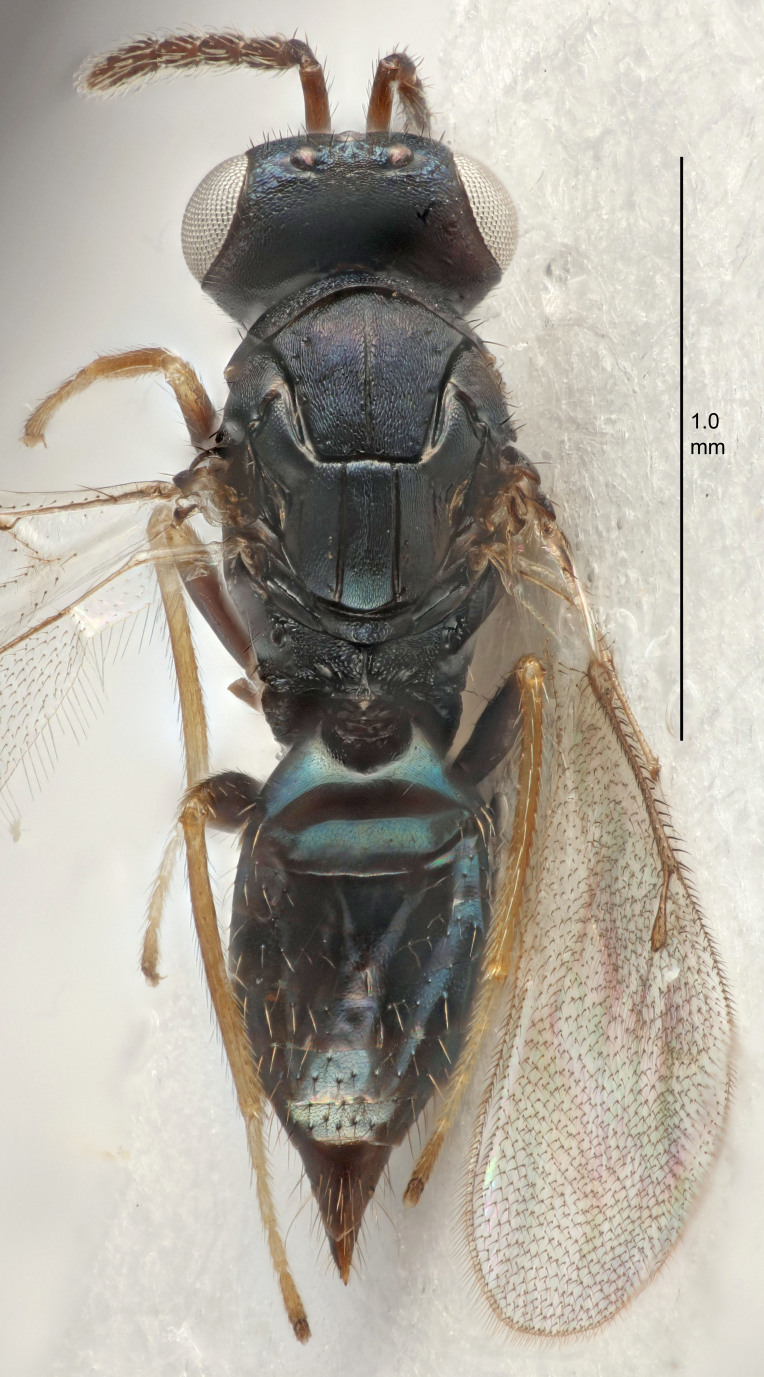
Nontype female, dorsal.

**Figure 77d.**
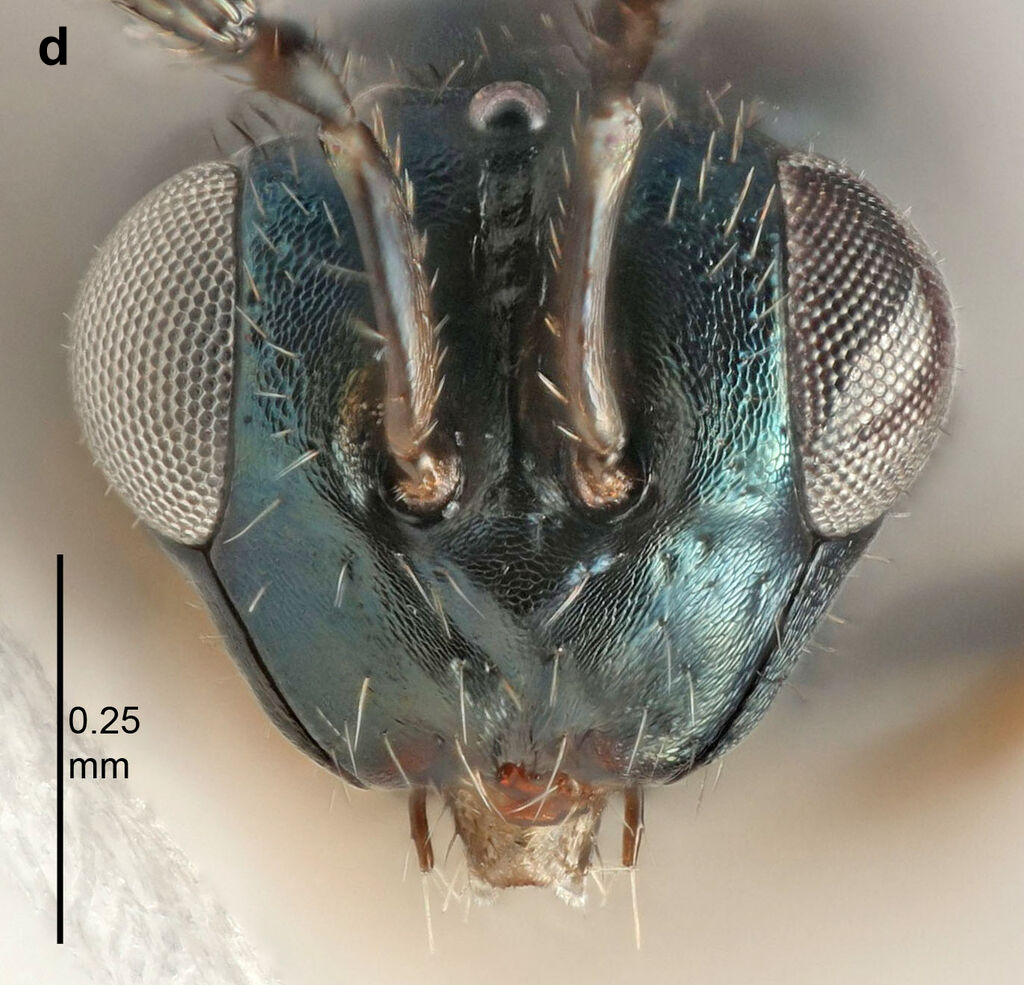
Nontype female, head frontal.

**Figure 77e.**
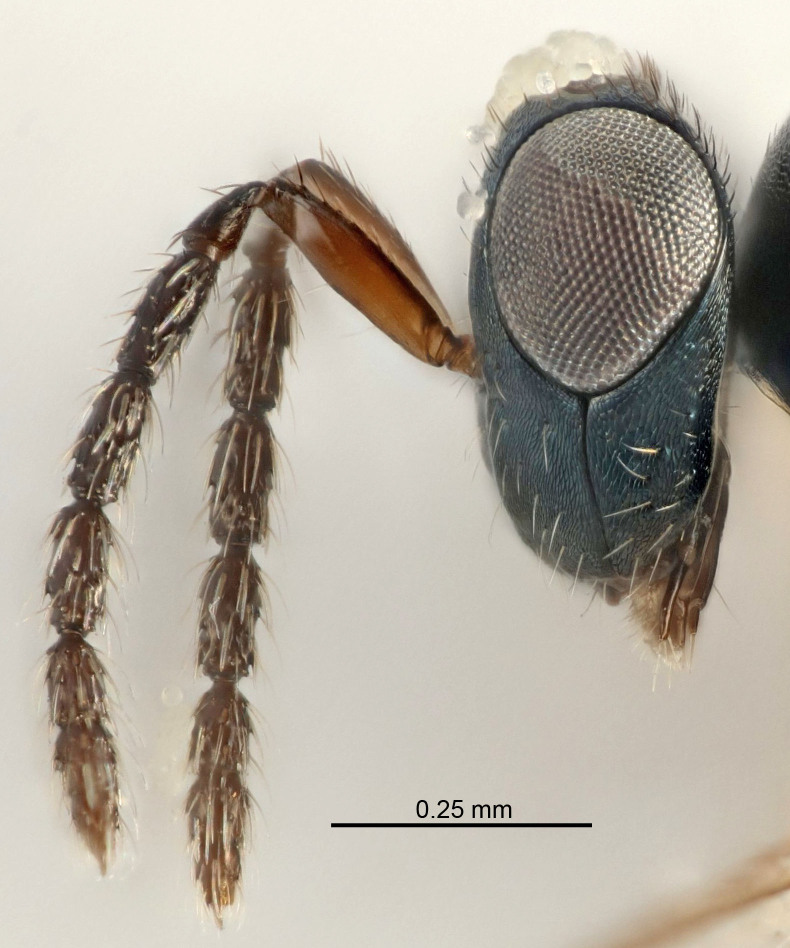
Nontype female, head and antenna lateral.

**Figure 77f.**
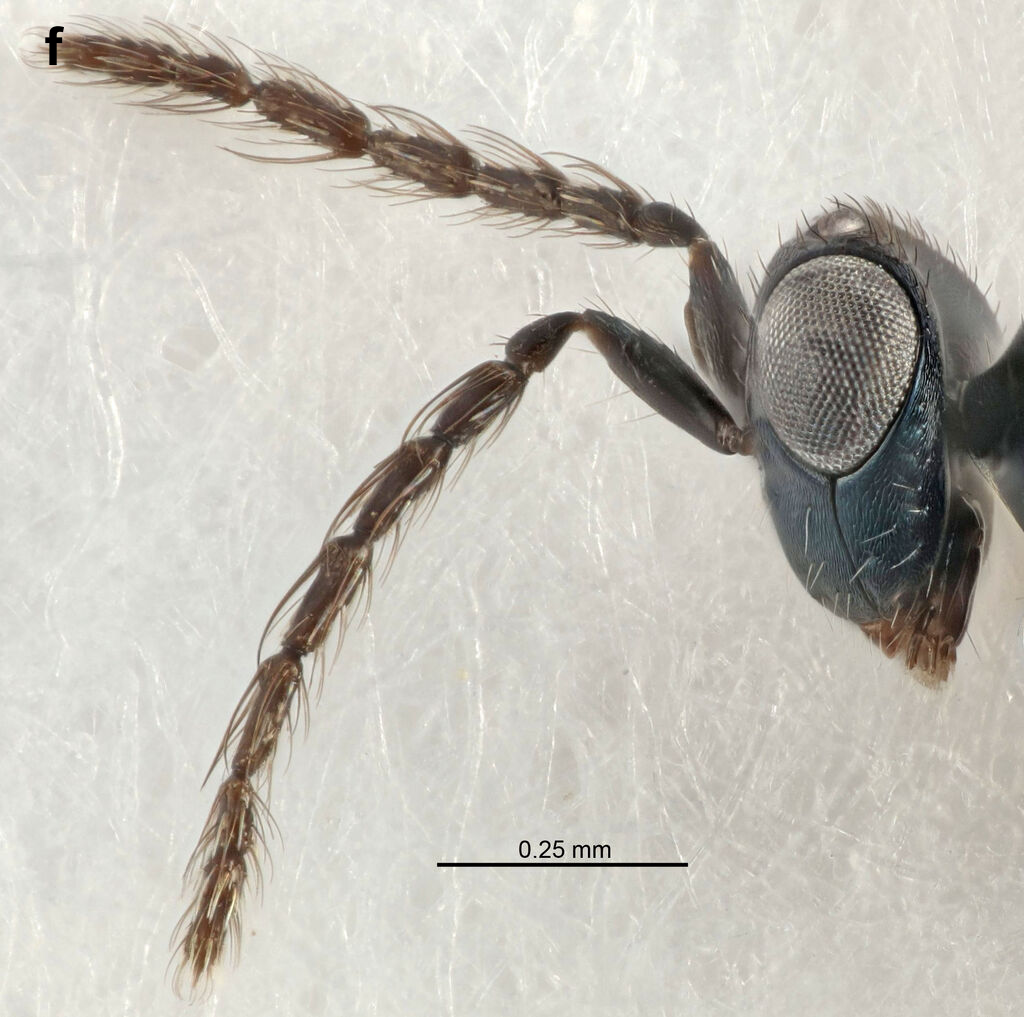
Nontype male, head and antenna lateral.

**Figure 78a.**
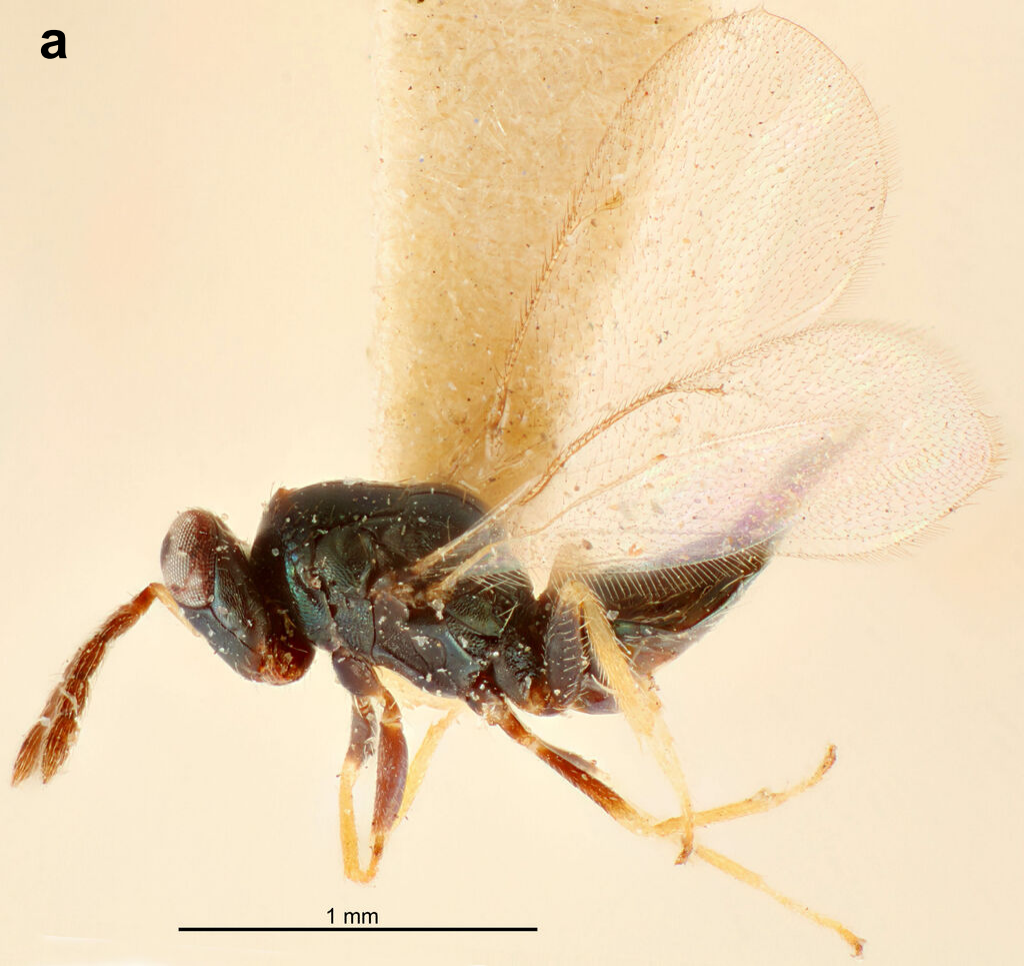
Holotype female, lateral.

**Figure 78b.**
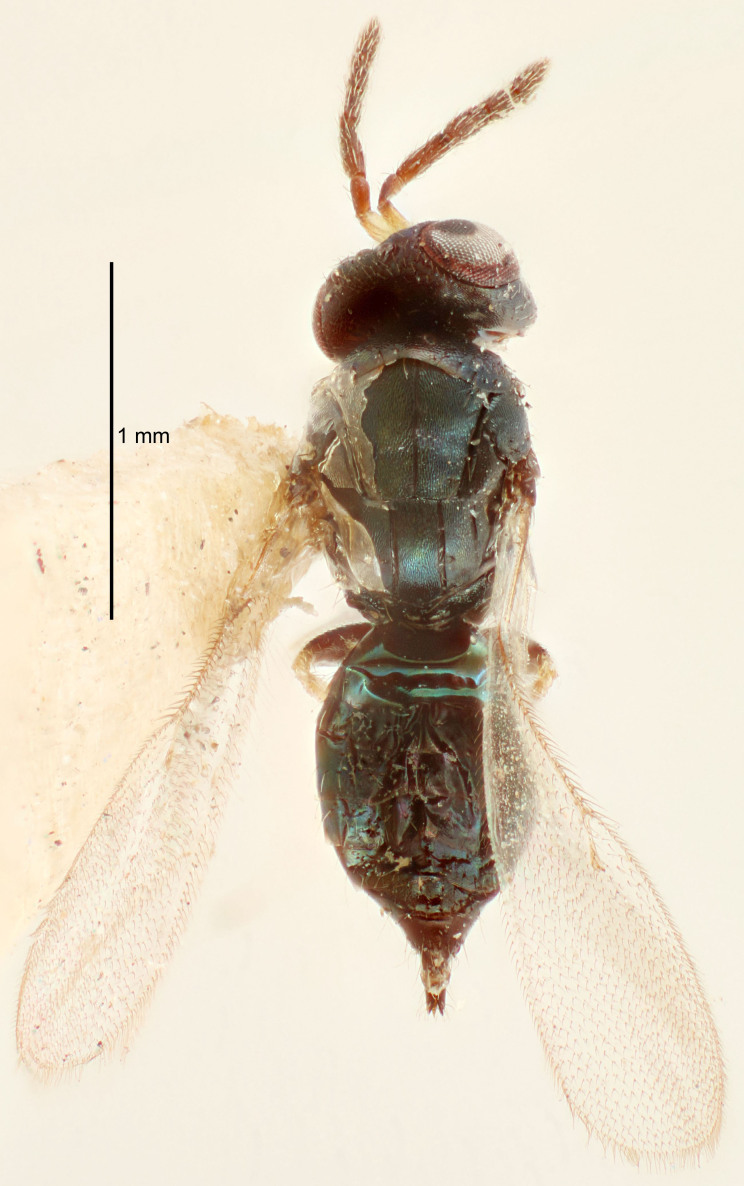
Holotype female, dorsal.

**Figure 79a.**
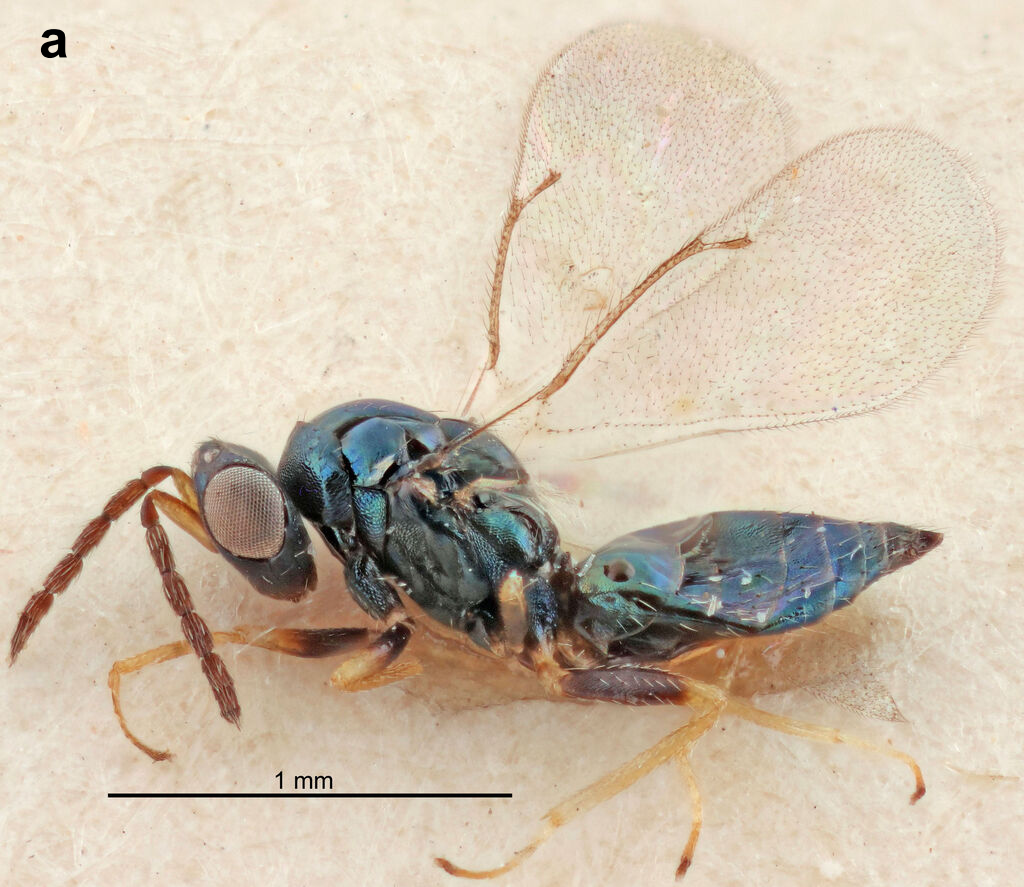
Holotype female, lateral.

**Figure 79b.**
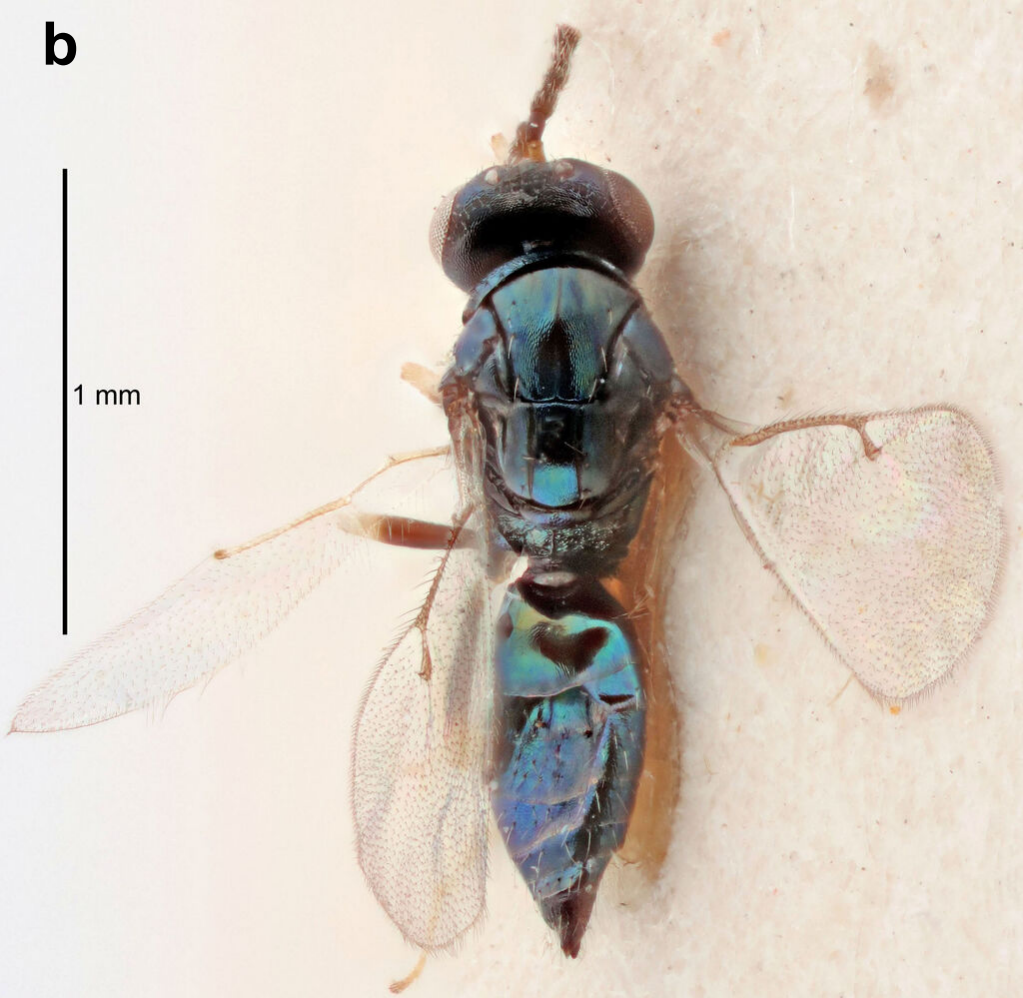
Holotype female, dorsal.

**Figure 80a.**
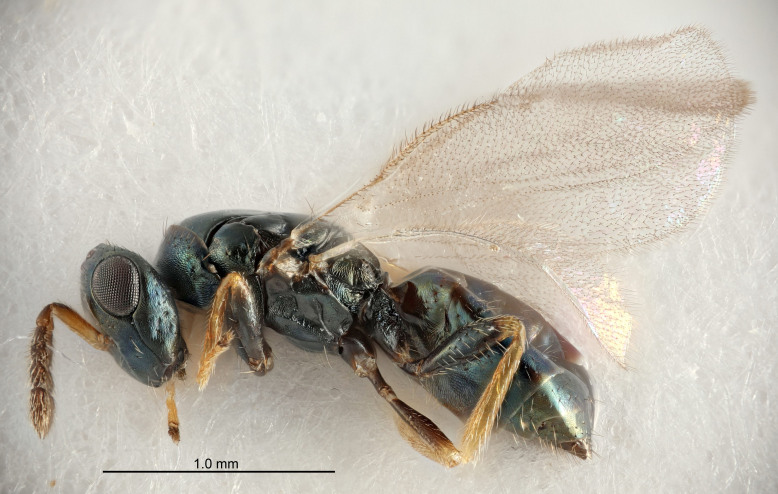
Holotype female, lateral.

**Figure 80b.**
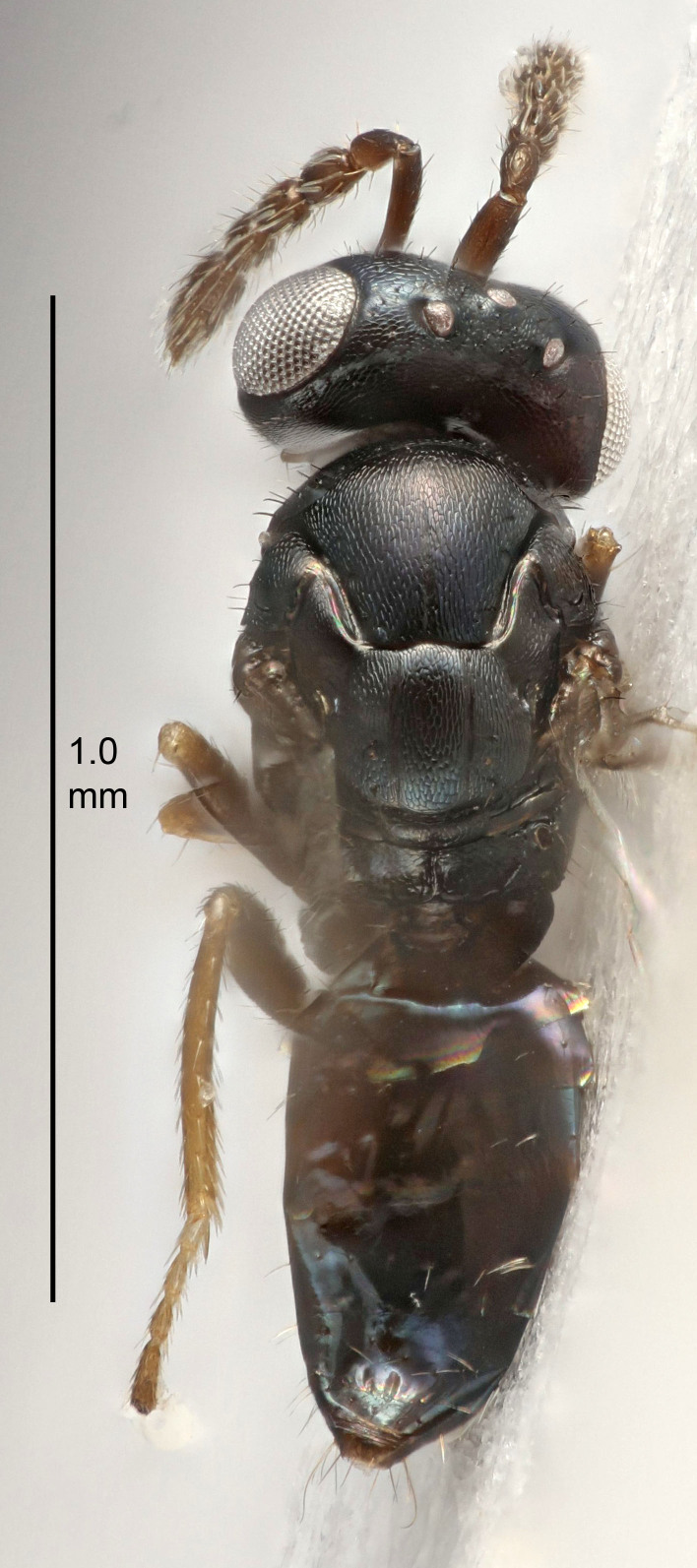
Holotype female, dorsal.

**Figure 81a.**
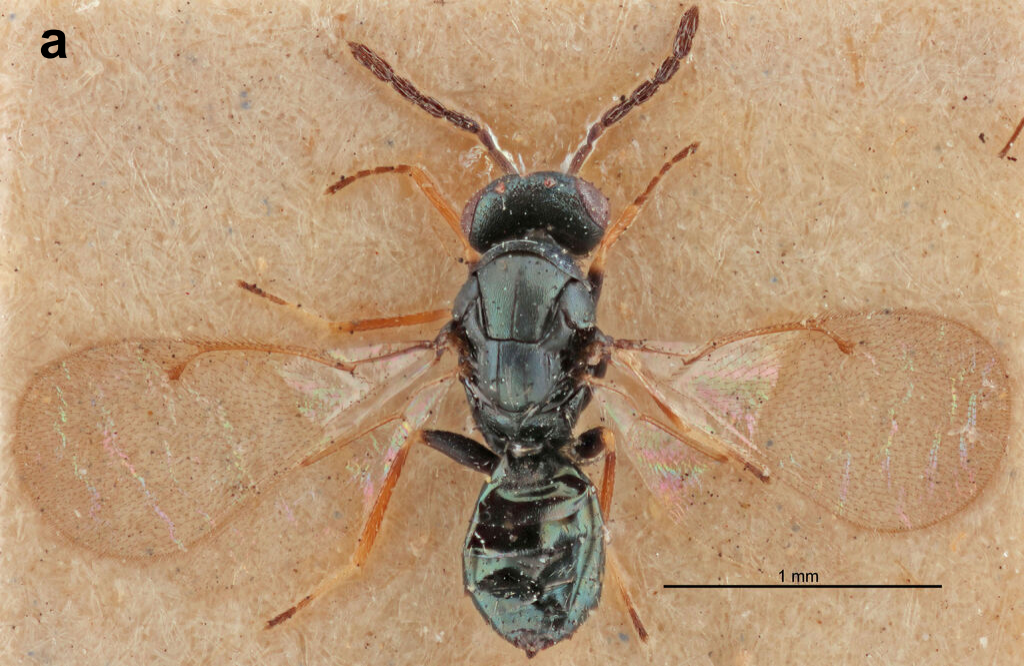
Neotype female, dorsal.

**Figure 81b.**
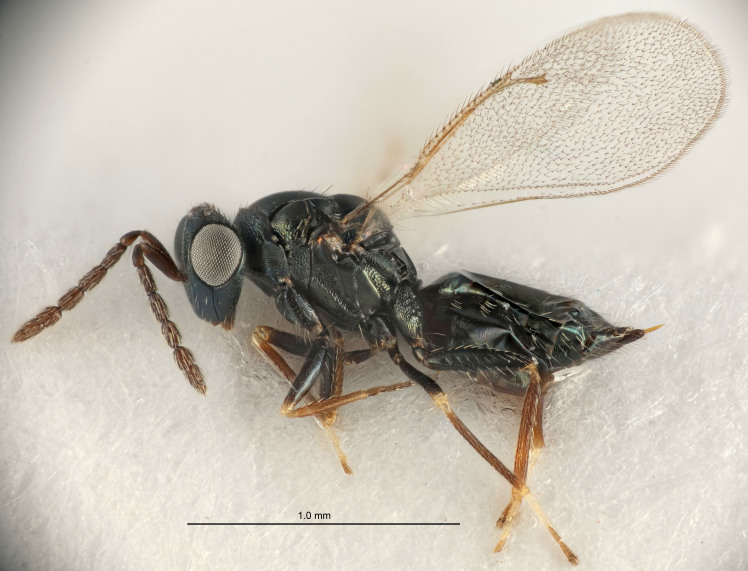
Nontype female, lateral.

**Figure 81c.**
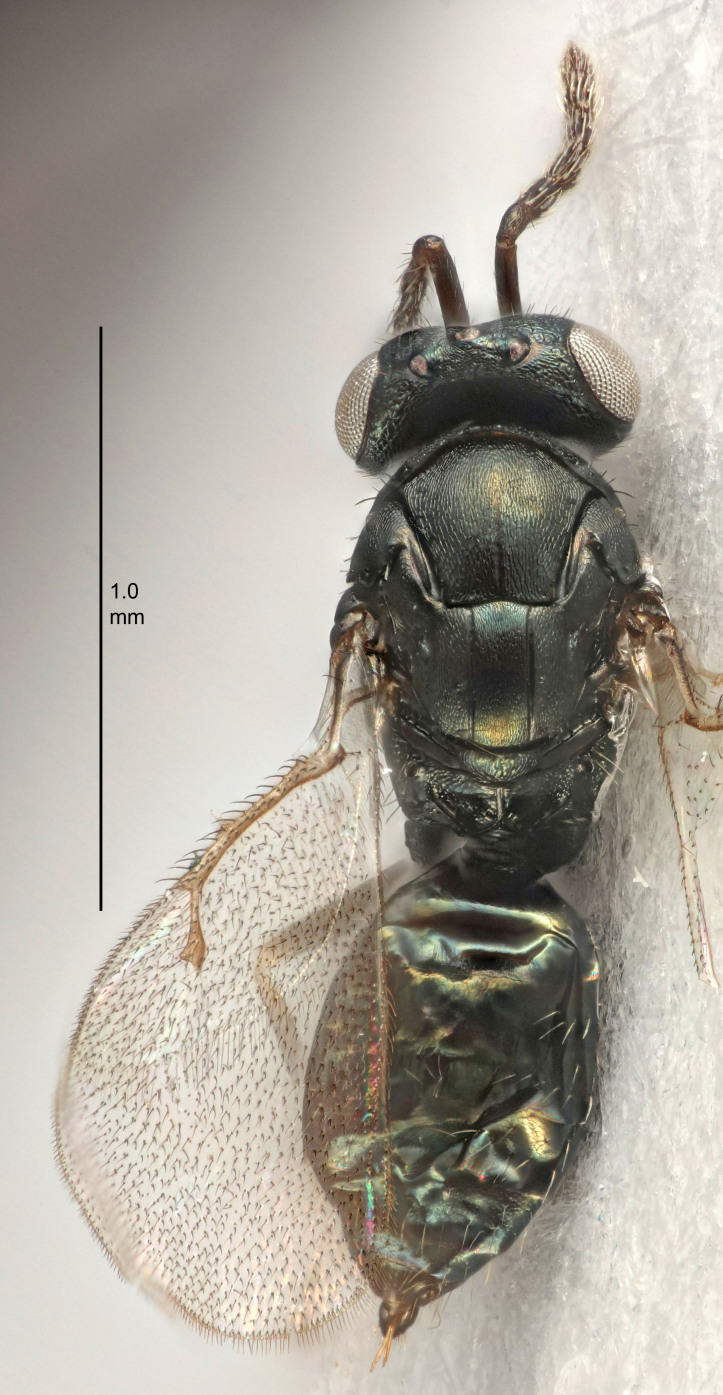
Nontype female, dorsal.

**Figure 81d.**
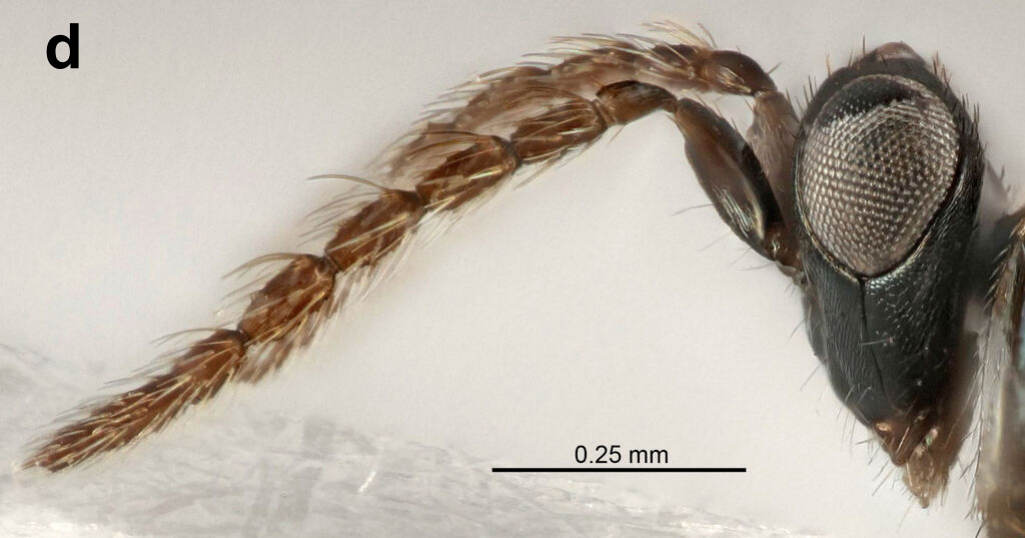
Nontype male, head and antenna lateral.

**Figure 82a.**
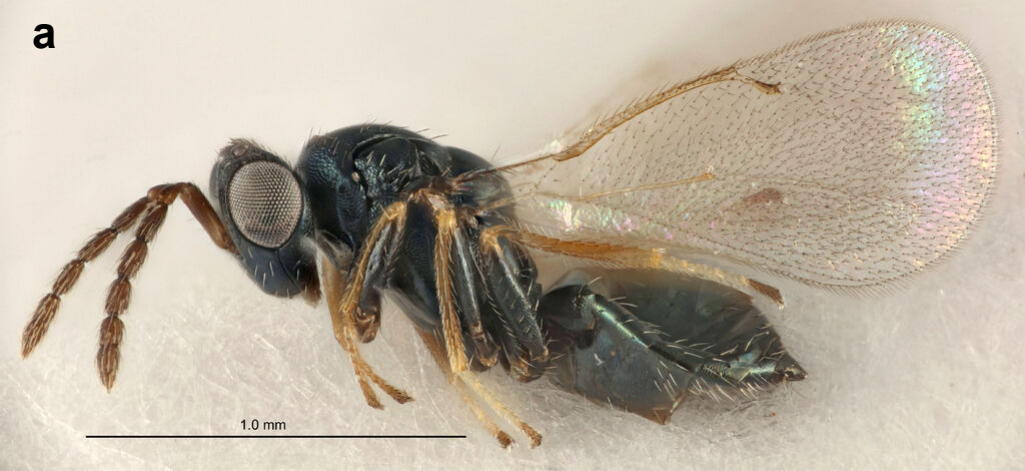
Holotype female, lateral.

**Figure 82b.**
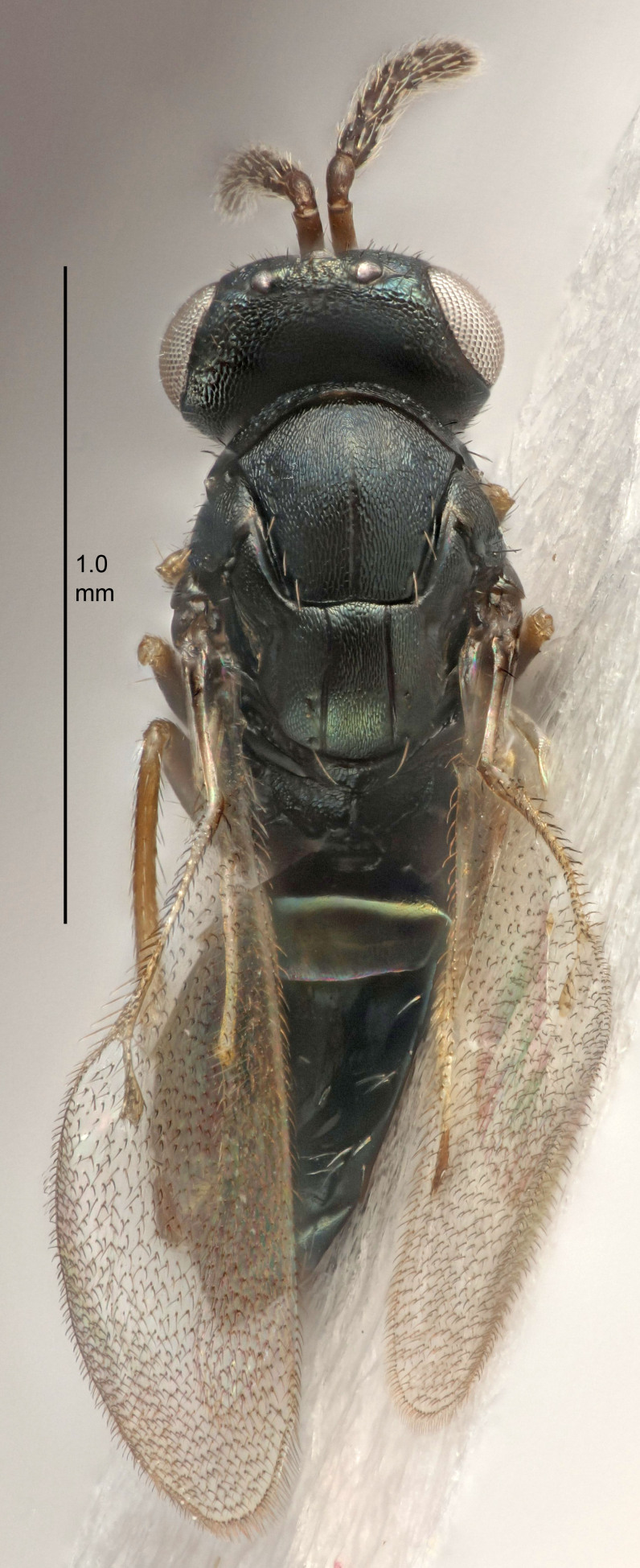
Holotype female, dorsal.

**Figure 83a.**
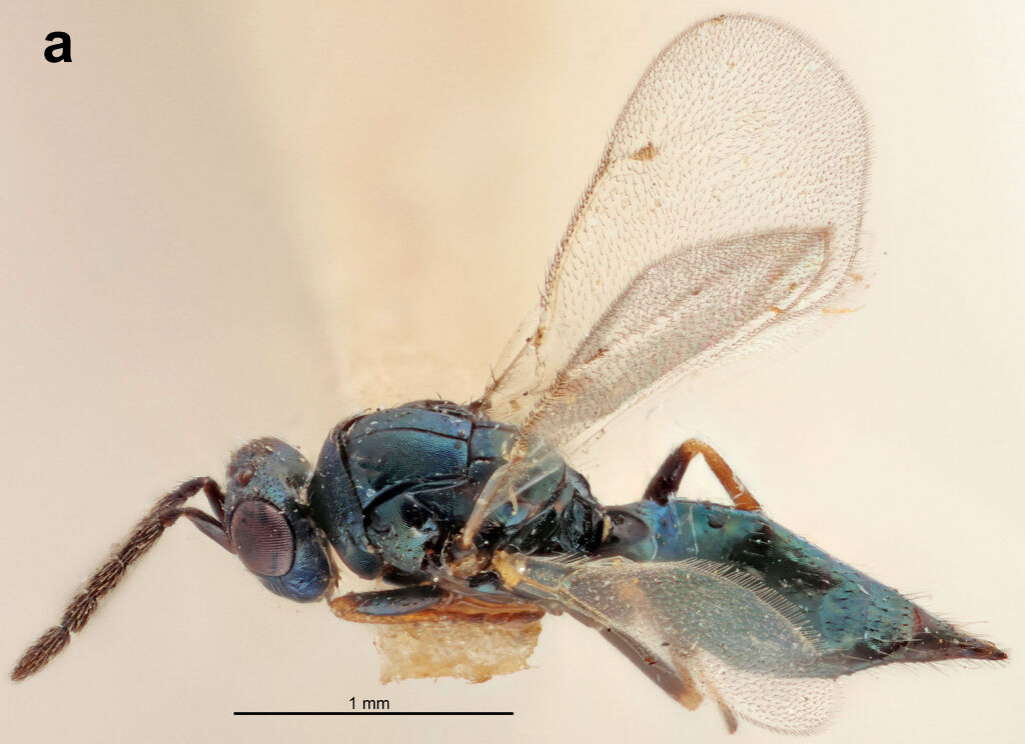
Holotype female, lateral.

**Figure 83b.**
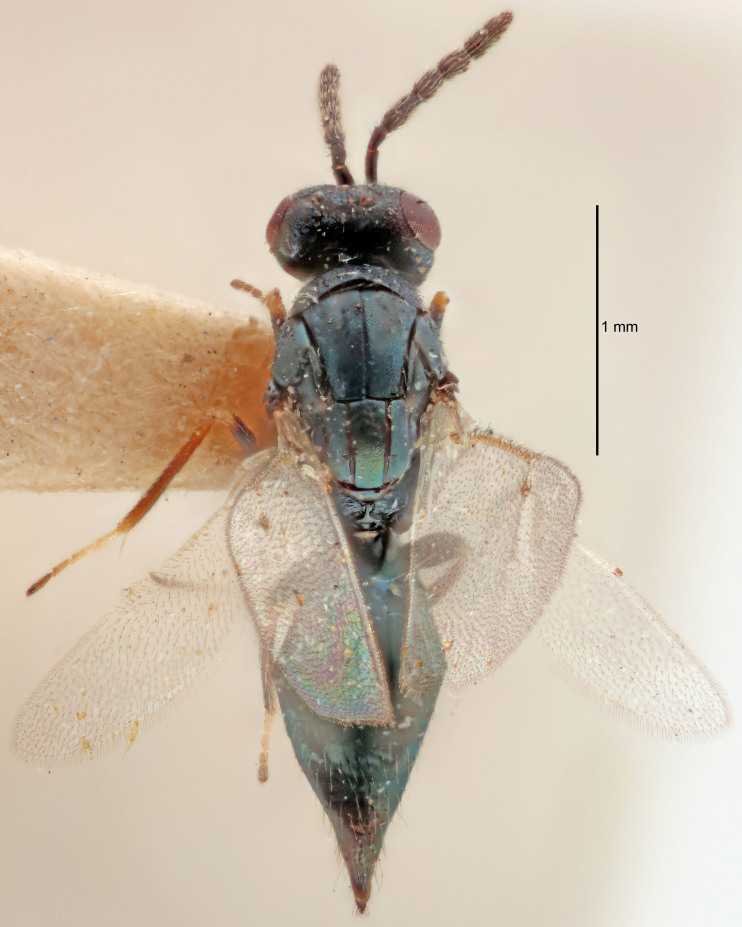
Holotype female, dorsal.

**Figure 83c.**
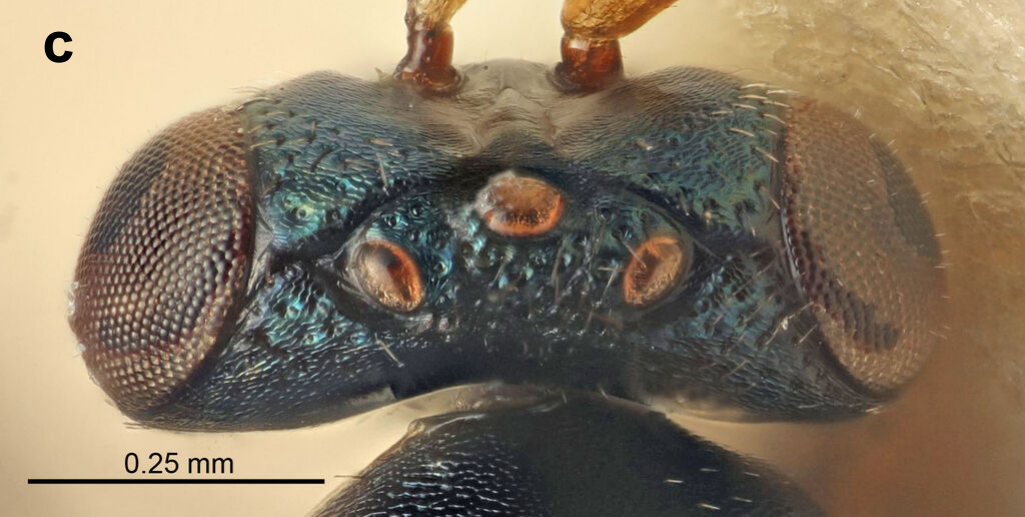
Nontype female, vertex.

**Figure 83d.**
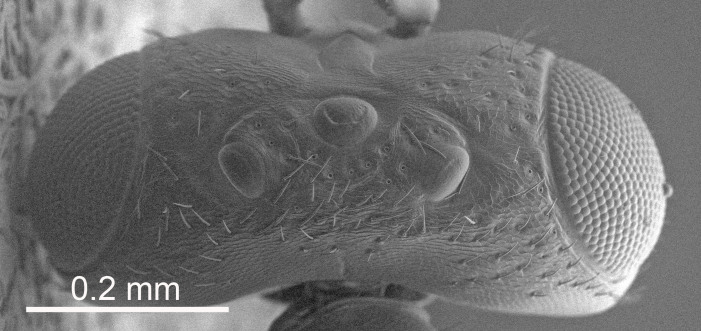
Nontype female, vertex.

**Figure 84a.**
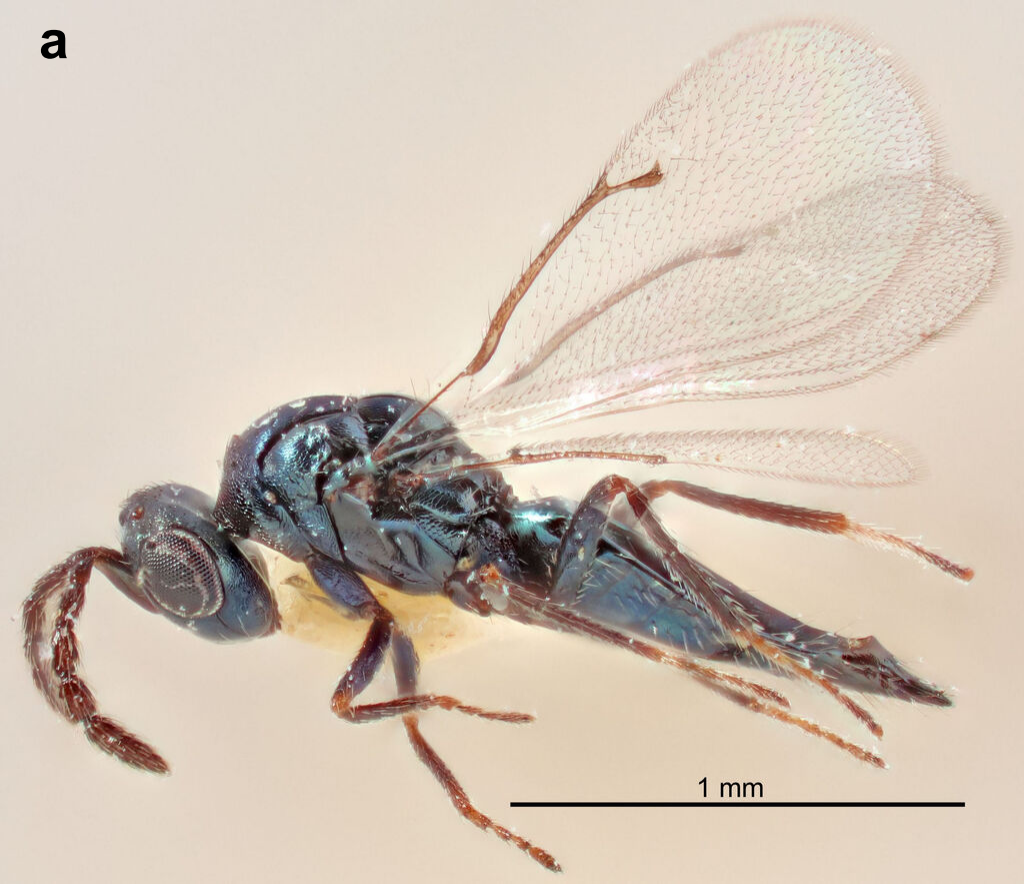
Holotype female, lateral.

**Figure 84b.**
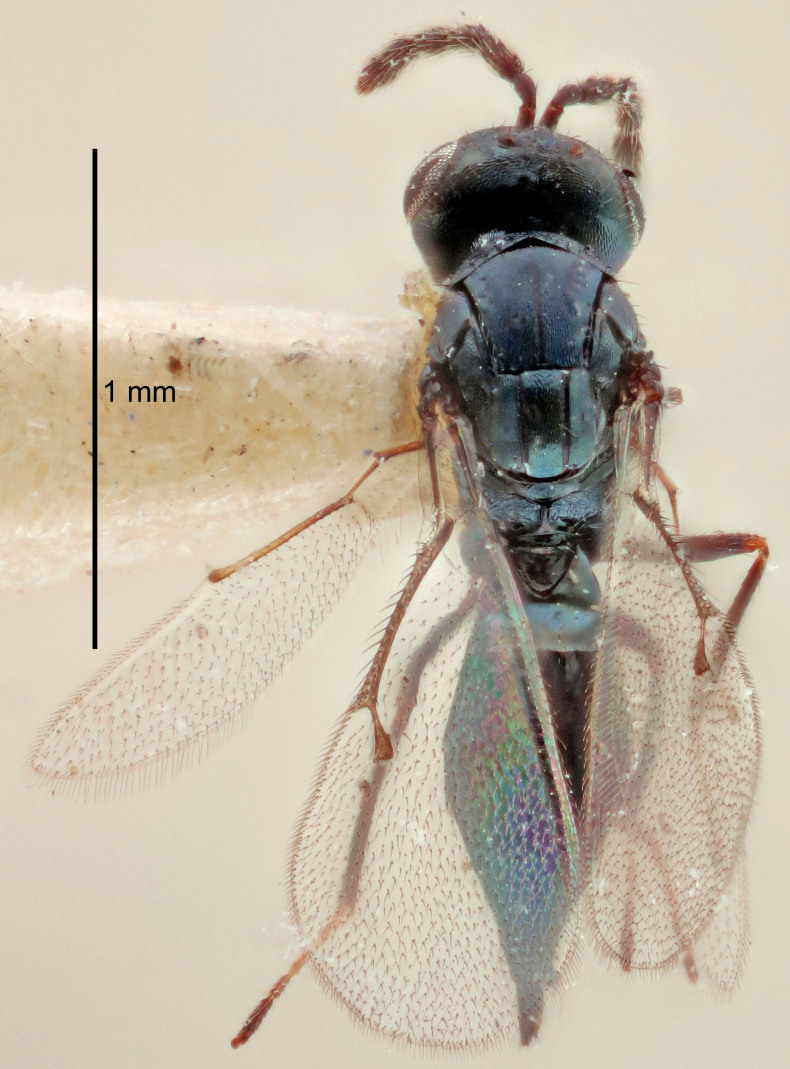
Holotype female, dorsal.

**Figure 85a.**
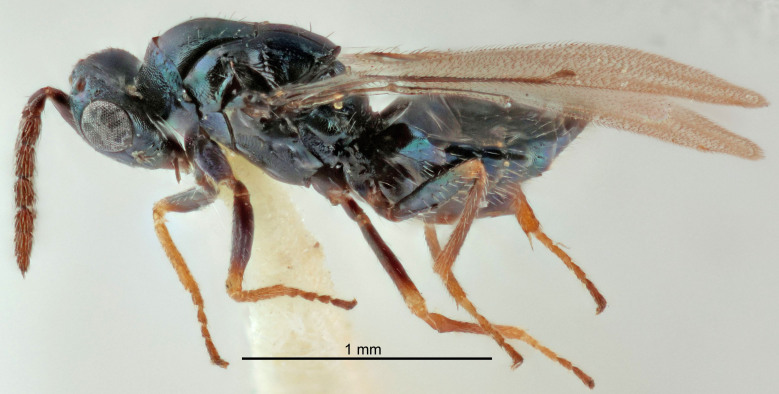
Holotype female, lateral.

**Figure 85b.**
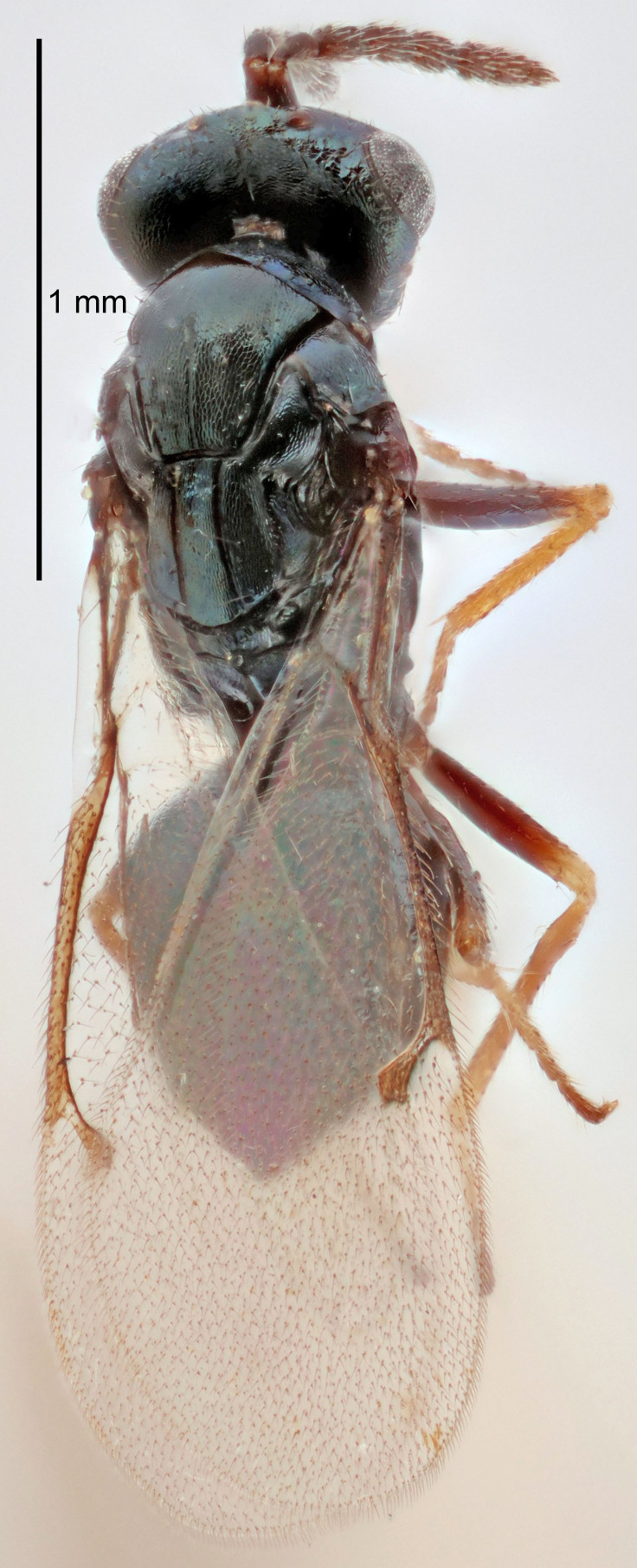
Holotype female, dorsal.

**Figure 86a.**
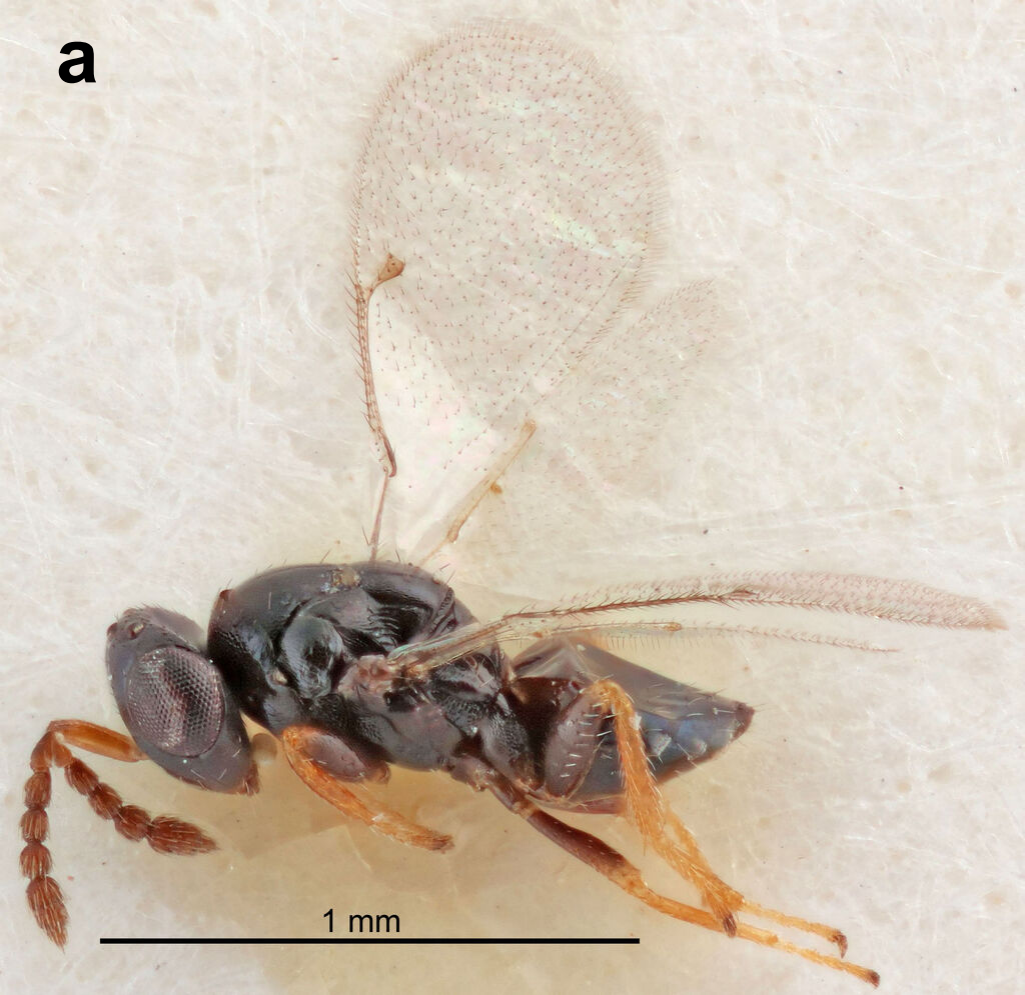
Holotype female, lateral.

**Figure 86b.**
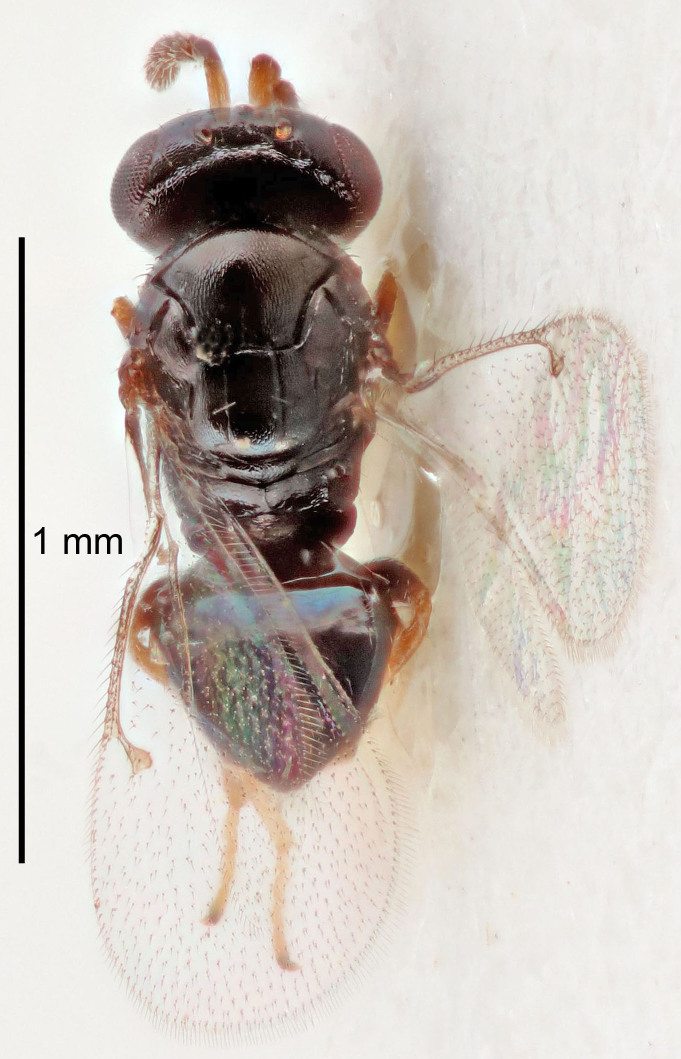
Holotype female, dorsal.

**Figure 86c.**
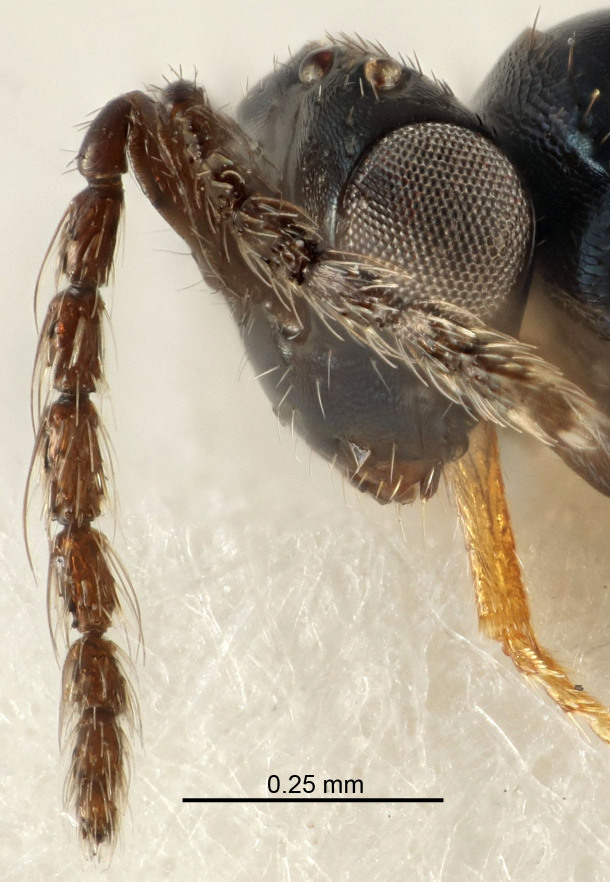
Nontype male, head and antenna lateral.

**Figure 87a.**
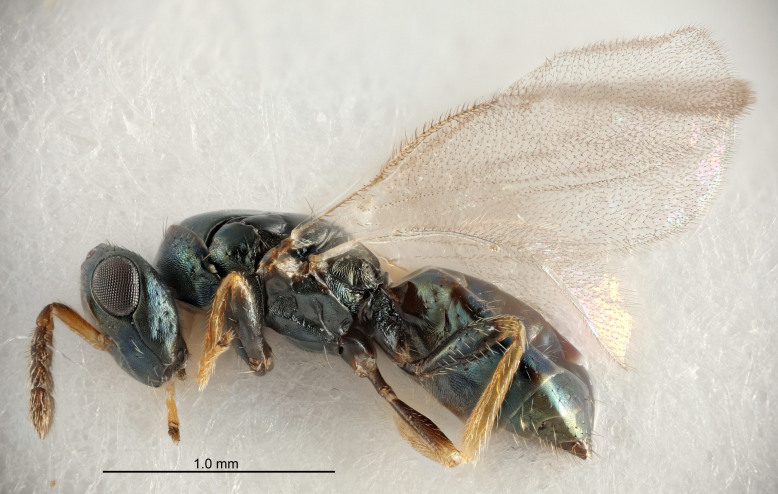
Holotype female, lateral.

**Figure 87b.**
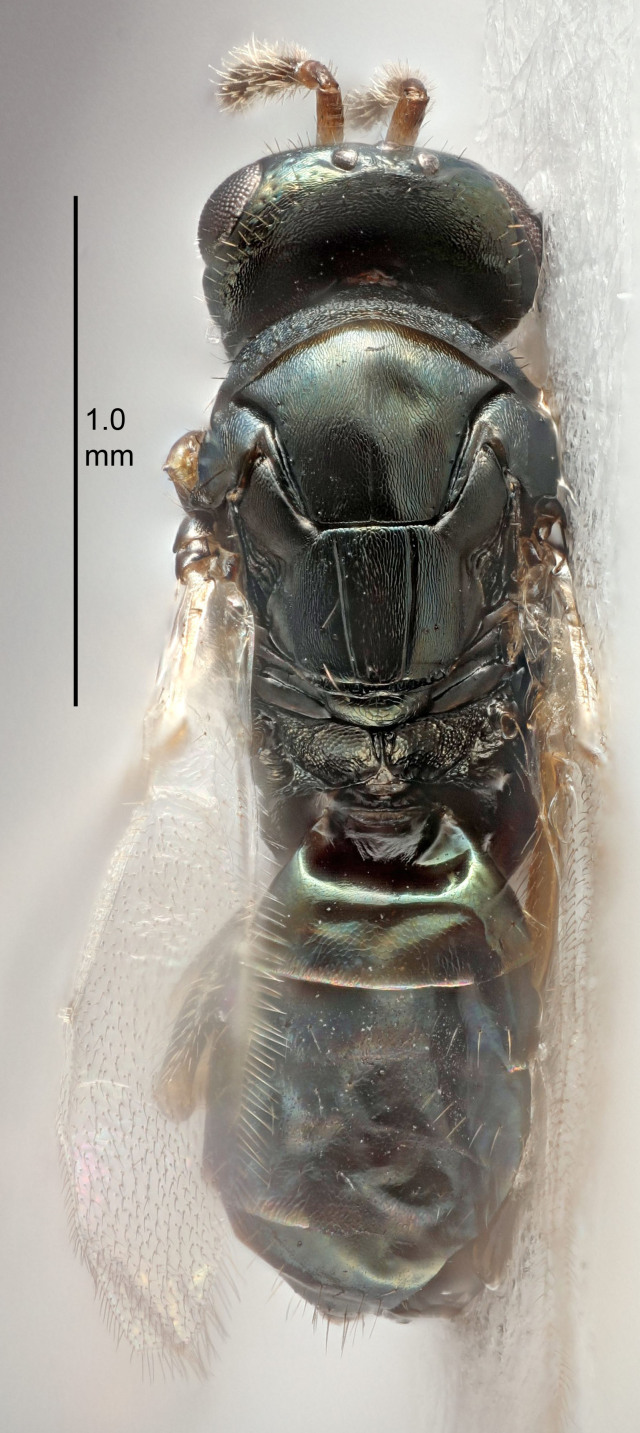
Holotype female, dorsal.

**Figure 87c.**
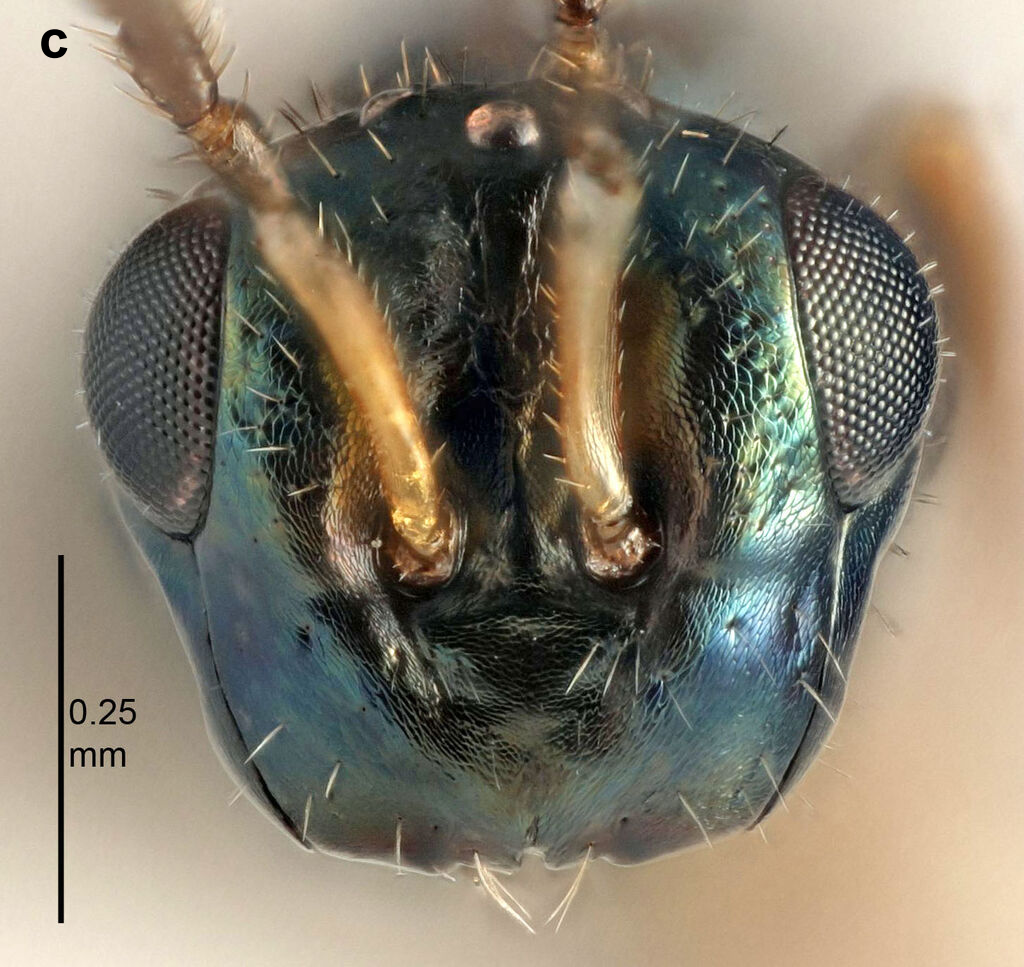
Paratype female, head frontal.

**Figure 88a.**
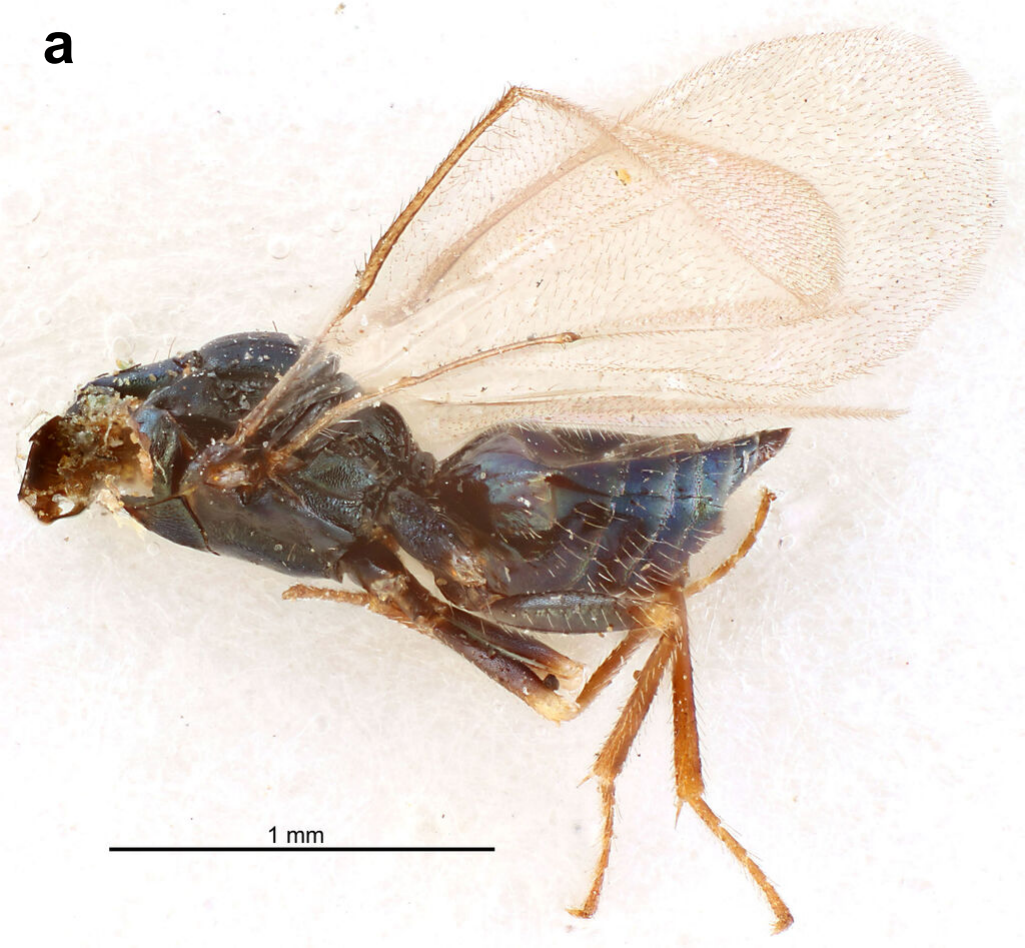
Lectotype female, body excl. head and prothorax lateral.

**Figure 88b.**
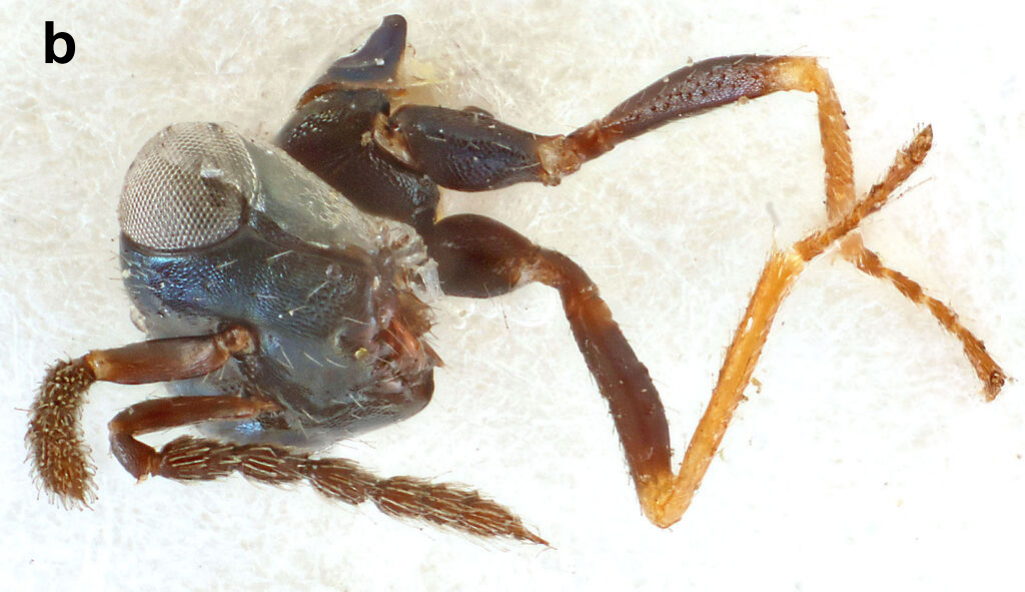
Lectotype female, head and prothorax lateral.

**Figure 88c.**
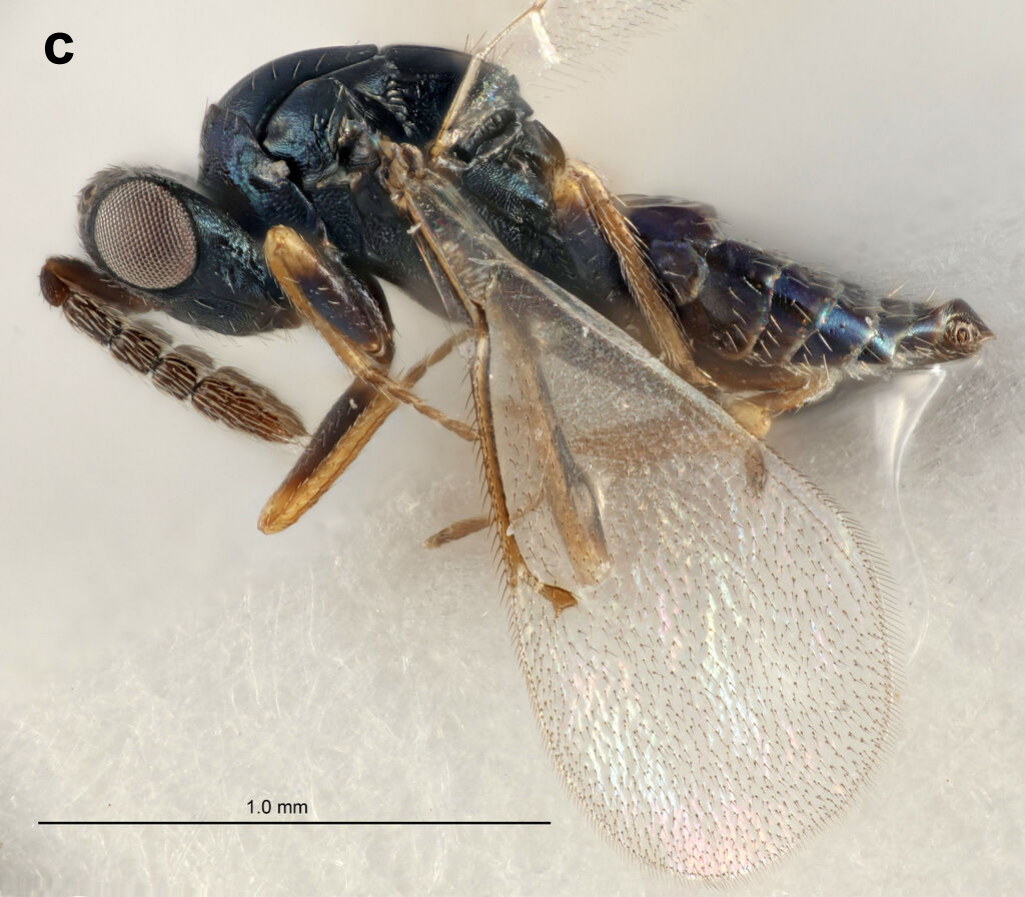
Nontype female, lateral.

**Figure 88d.**
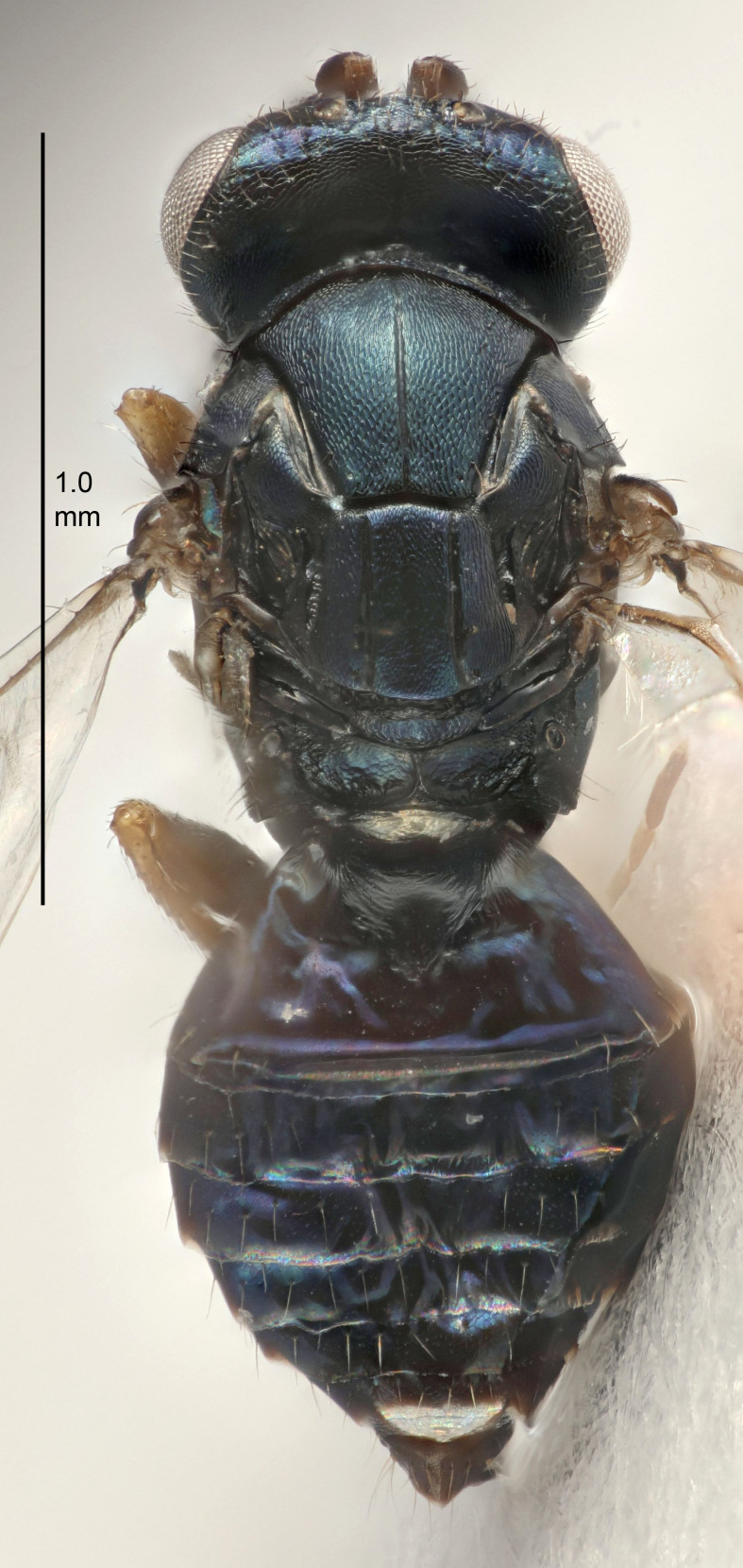
Nontype female, dorsal.

**Figure 88e.**
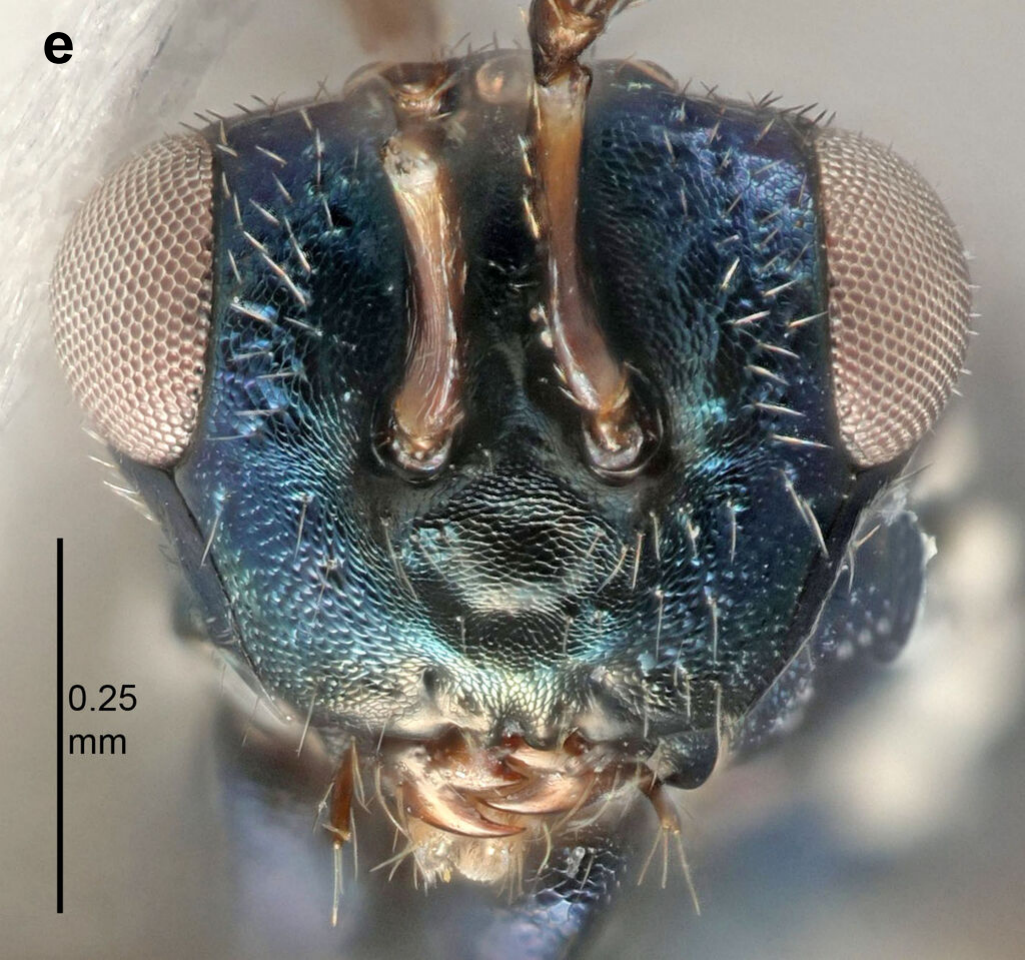
Nontype female, head frontal.

**Figure 88f.**
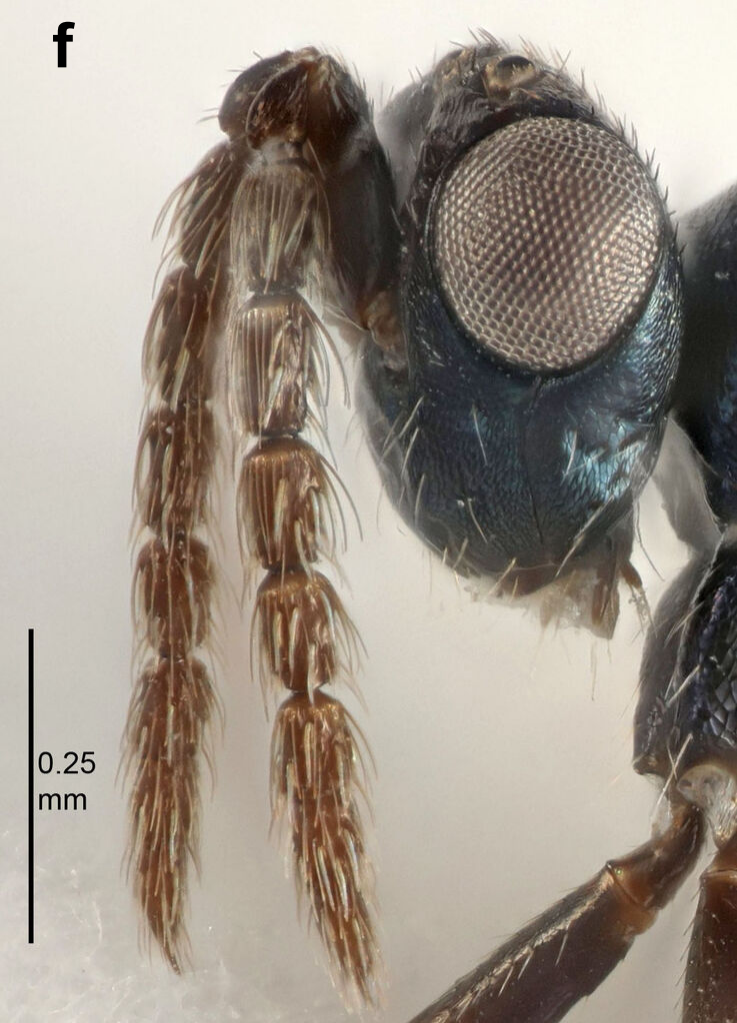
Nontype male, head and antenna lateral.

**Figure 89a.**
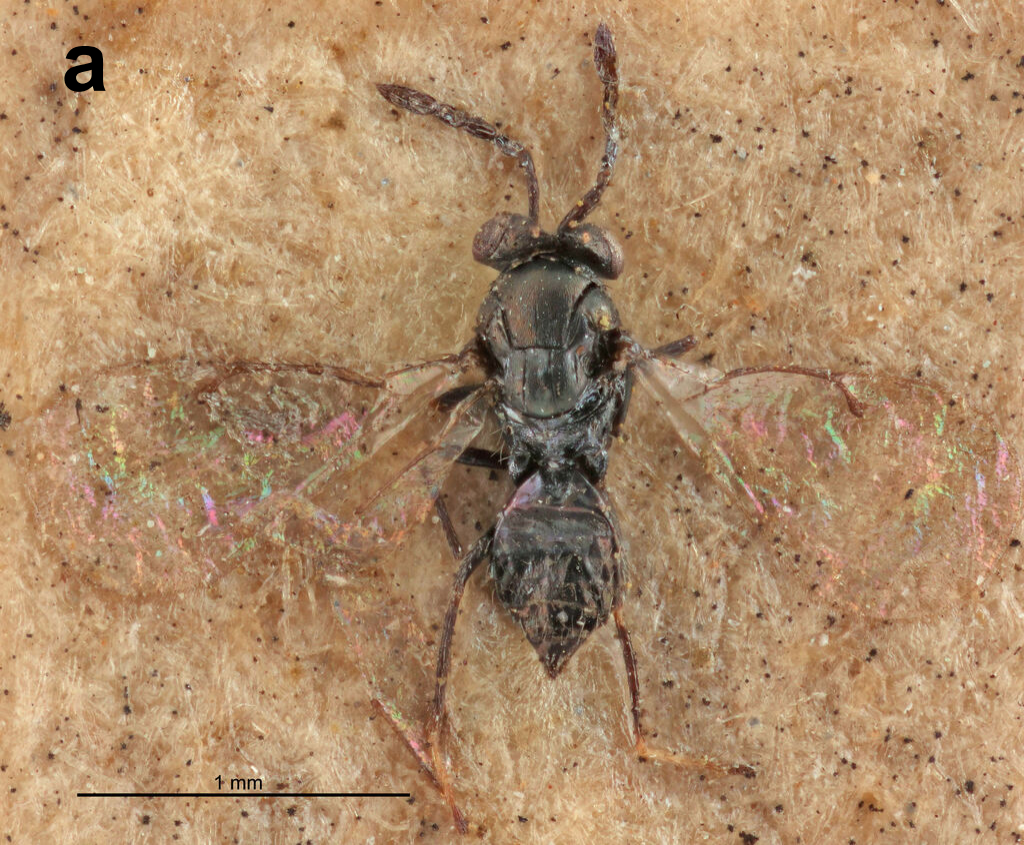
Lectotype female, dorsal.

**Figure 89b.**
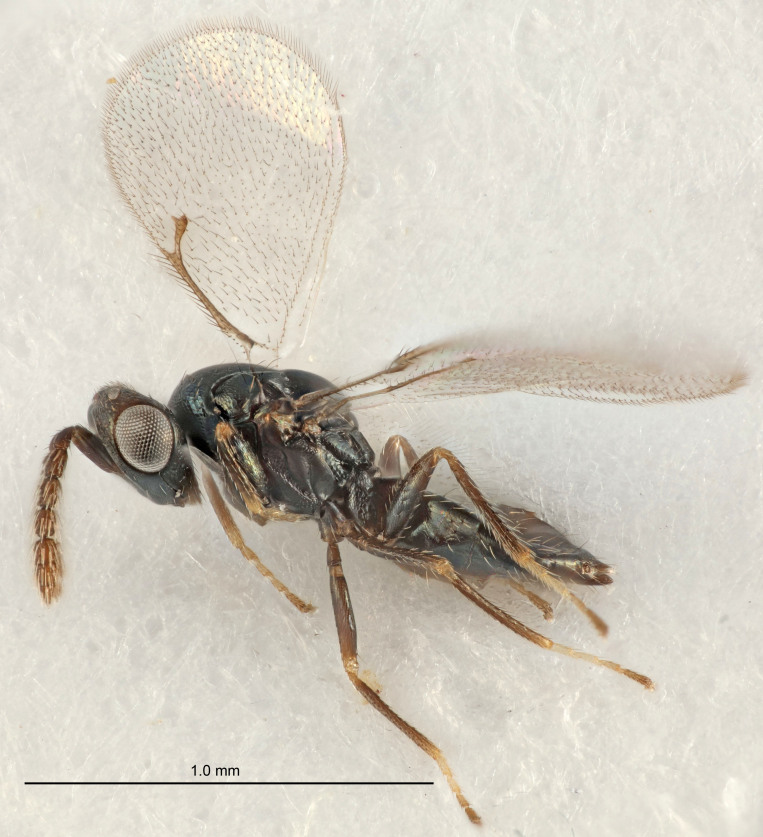
Nontype female, lateral.

**Figure 89c.**
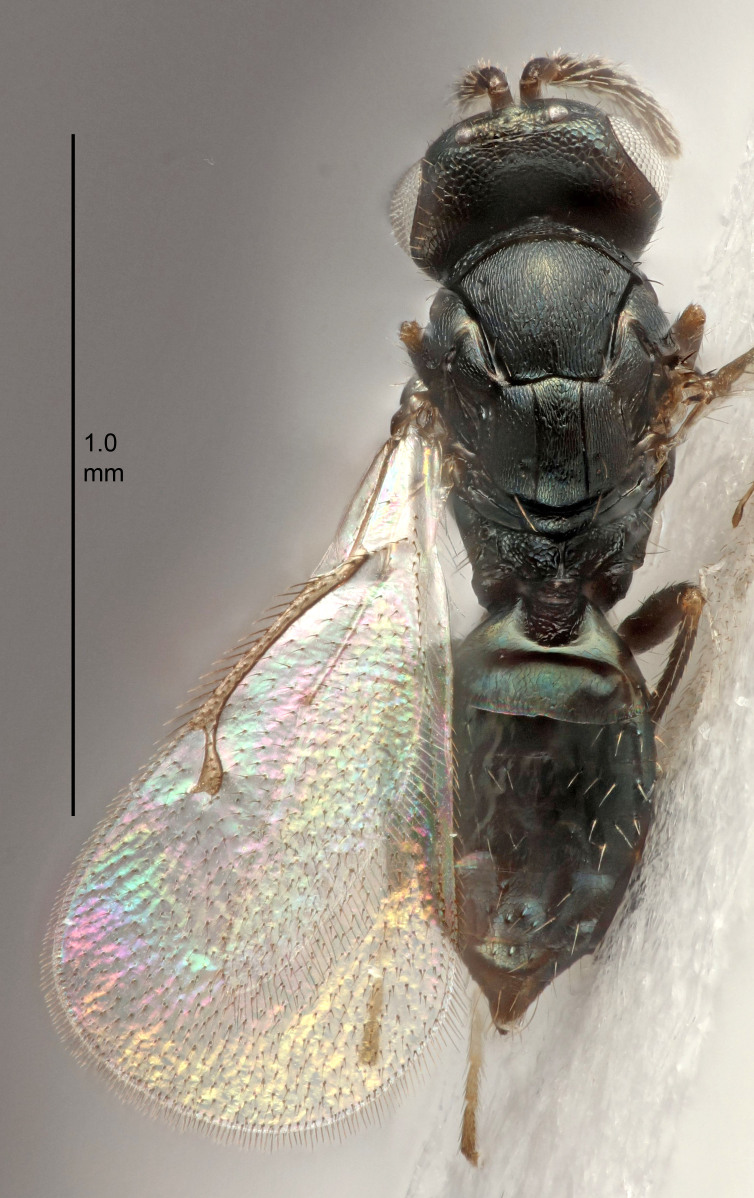
Nontype female, dorsal.

**Figure 90a.**
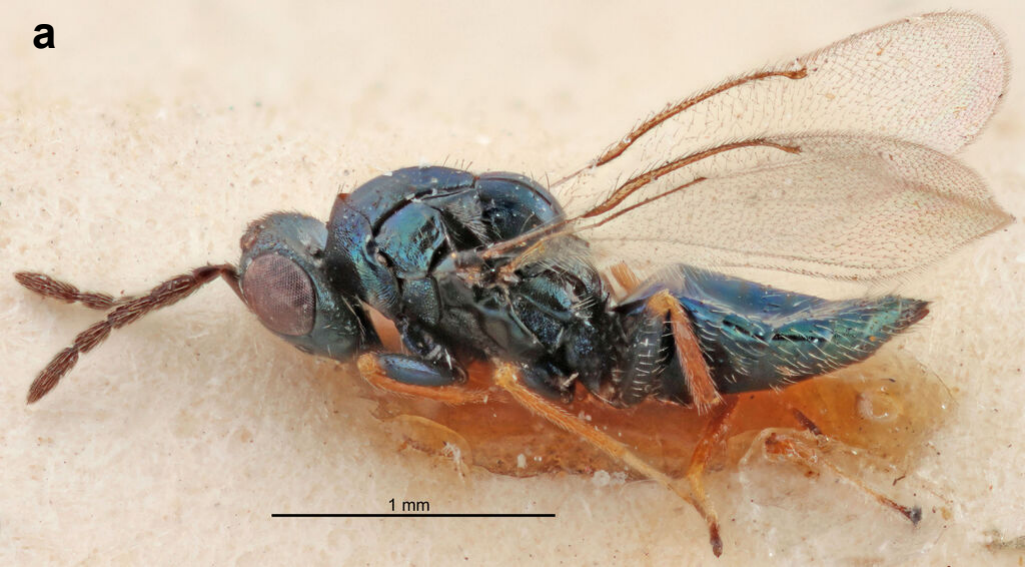
Holotype female, lateral.

**Figure 90b.**
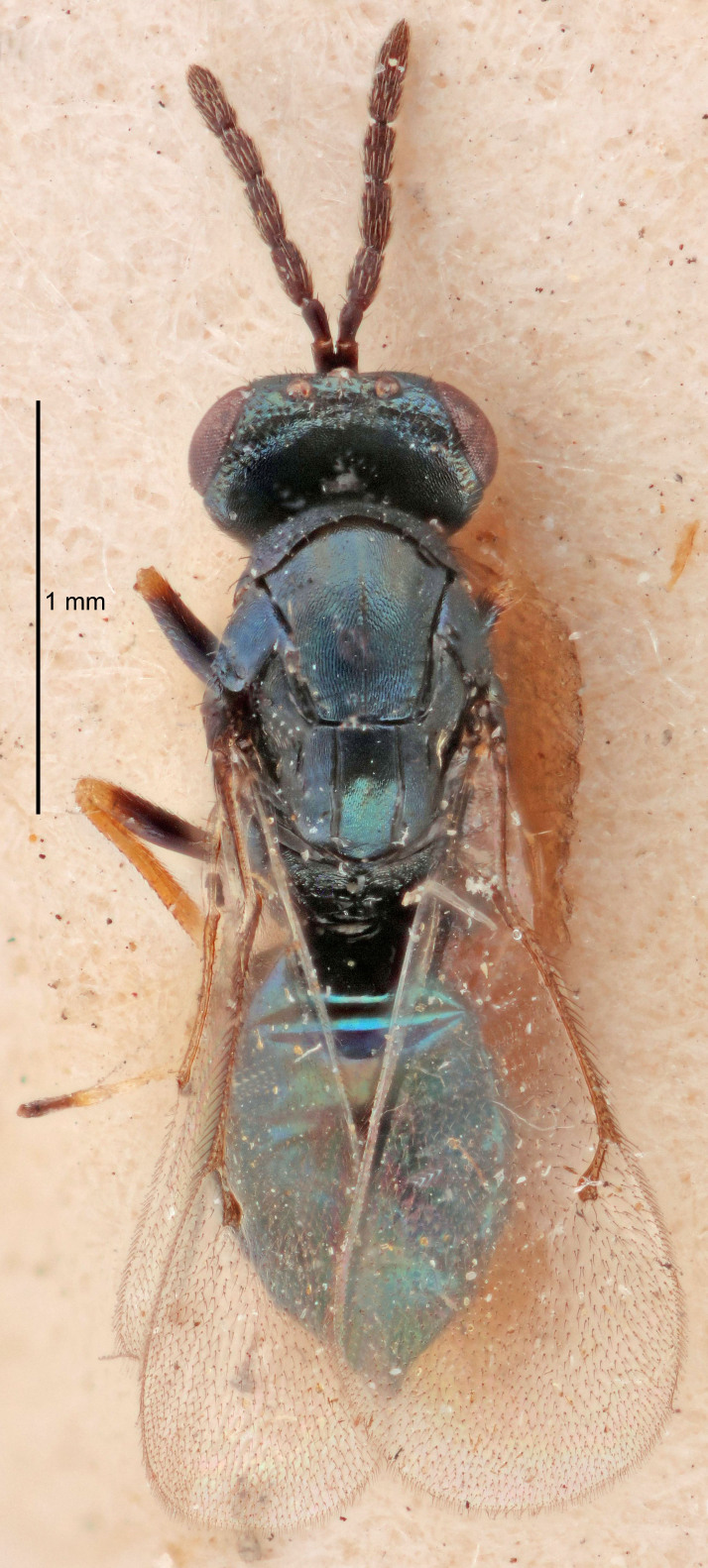
Holotype female, dorsal.

**Figure 91a.**
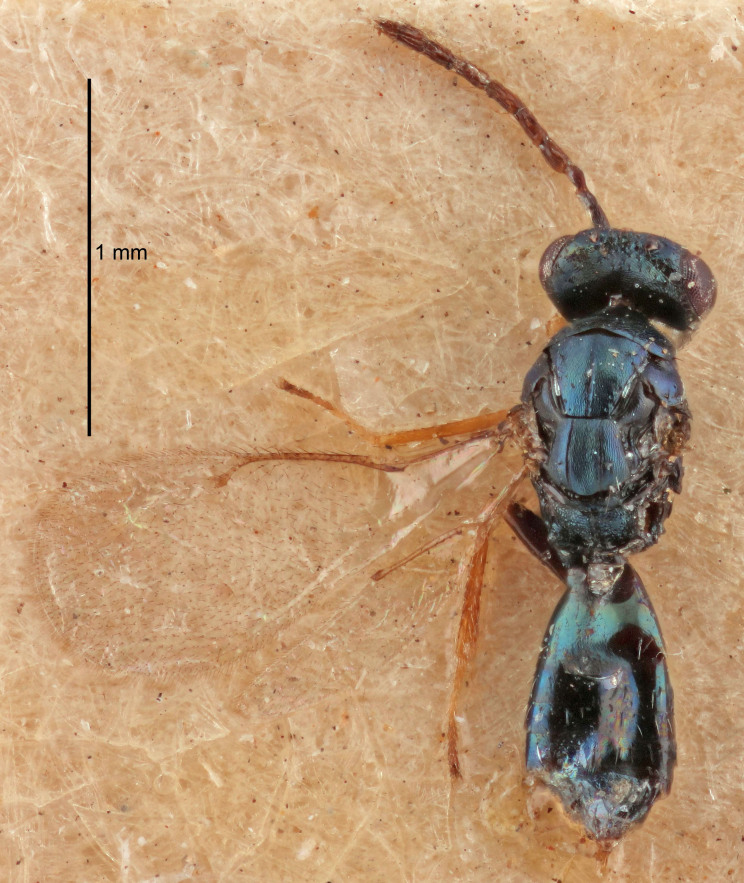
Lectotype male, dorsal.

**Figure 91b.**
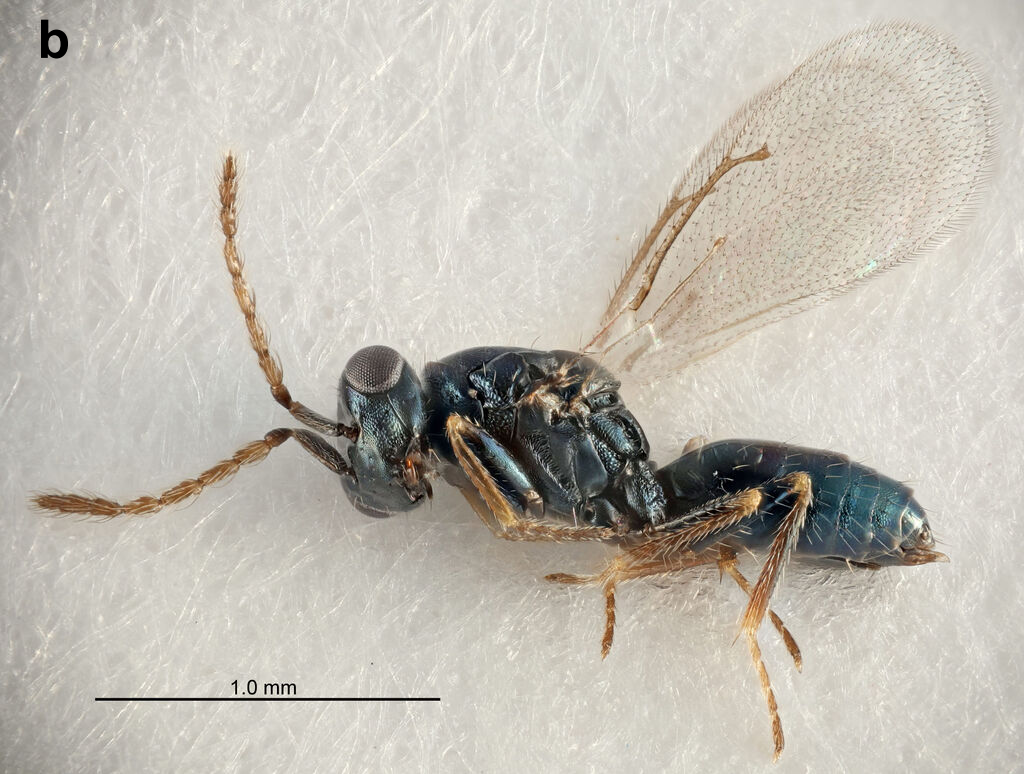
Nontype male, lateral.

**Figure 91c.**
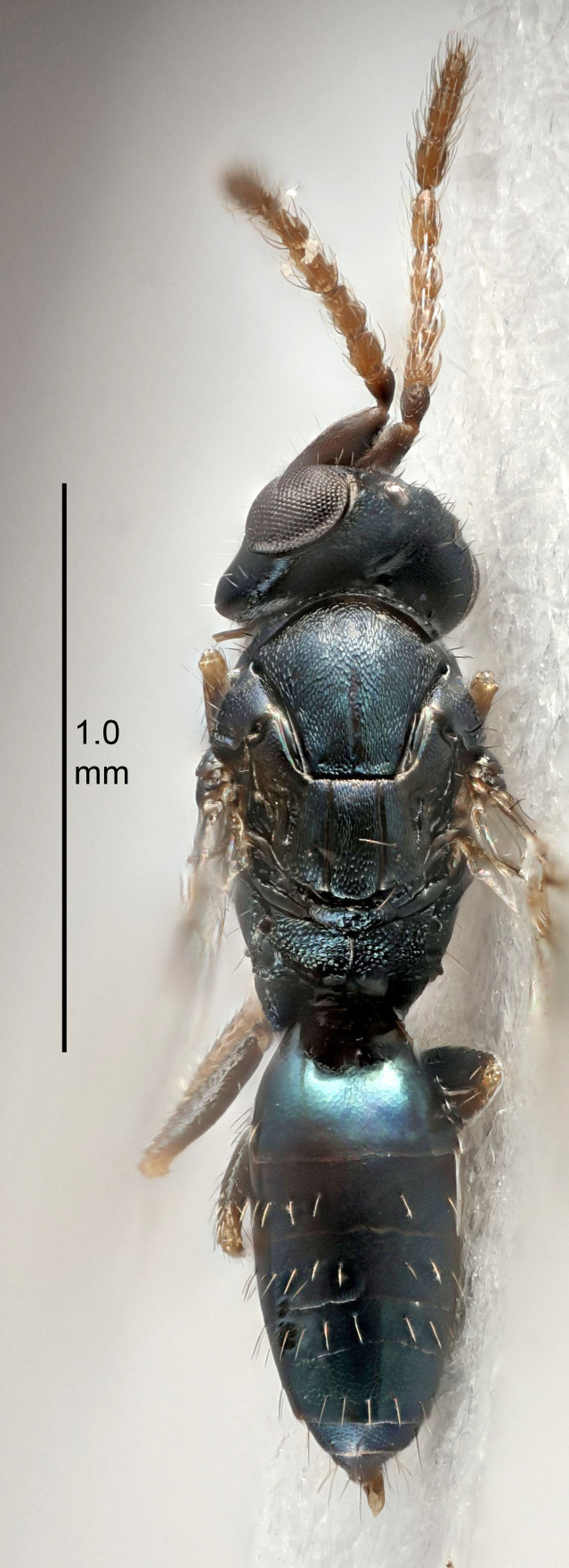
Nontype male, dorsal.

**Figure 91d.**
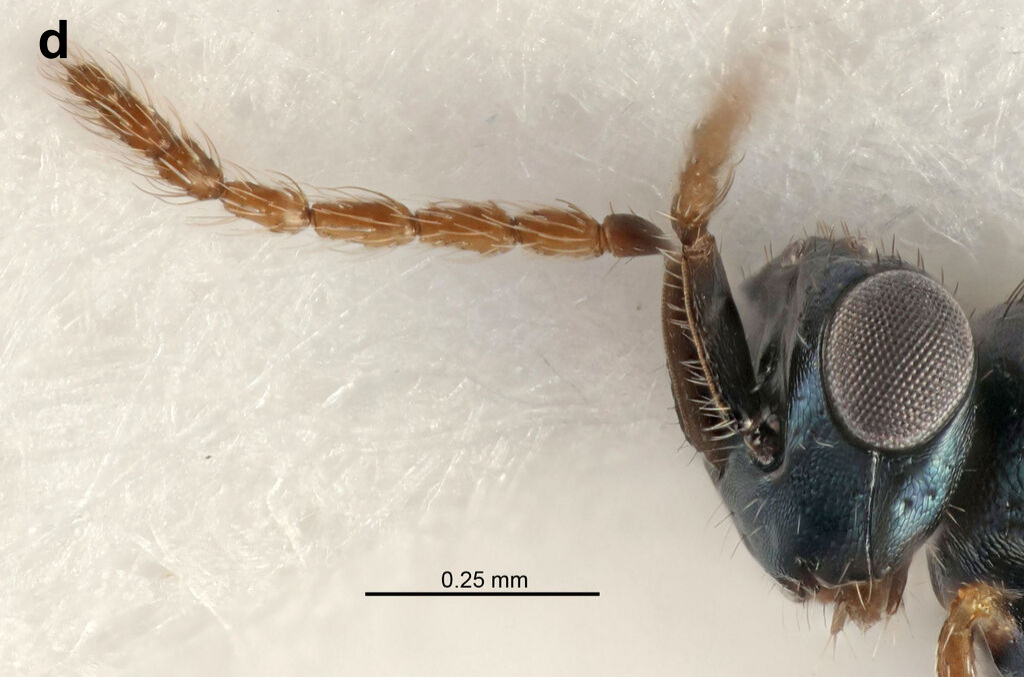
Nontype male, head and antenna lateral.

**Figure 91e.**
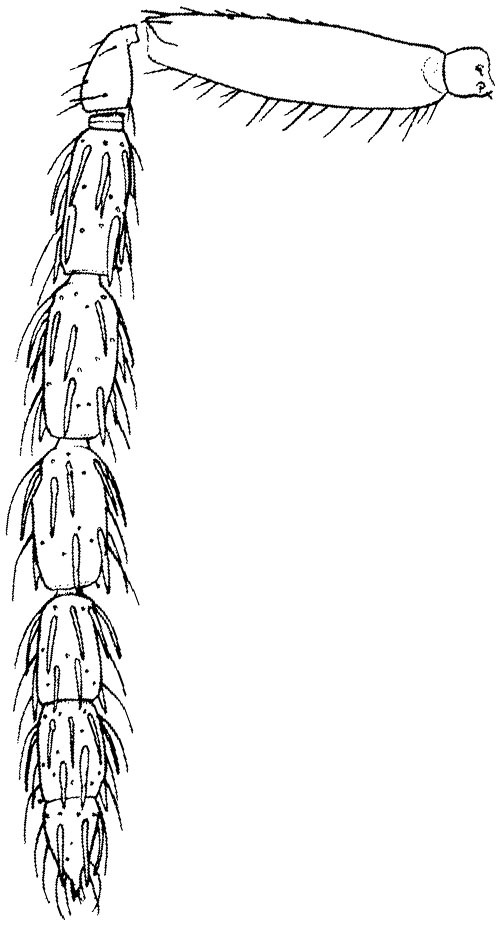
Female, antenna lateral, from Graham (1991).

**Figure 92a.**
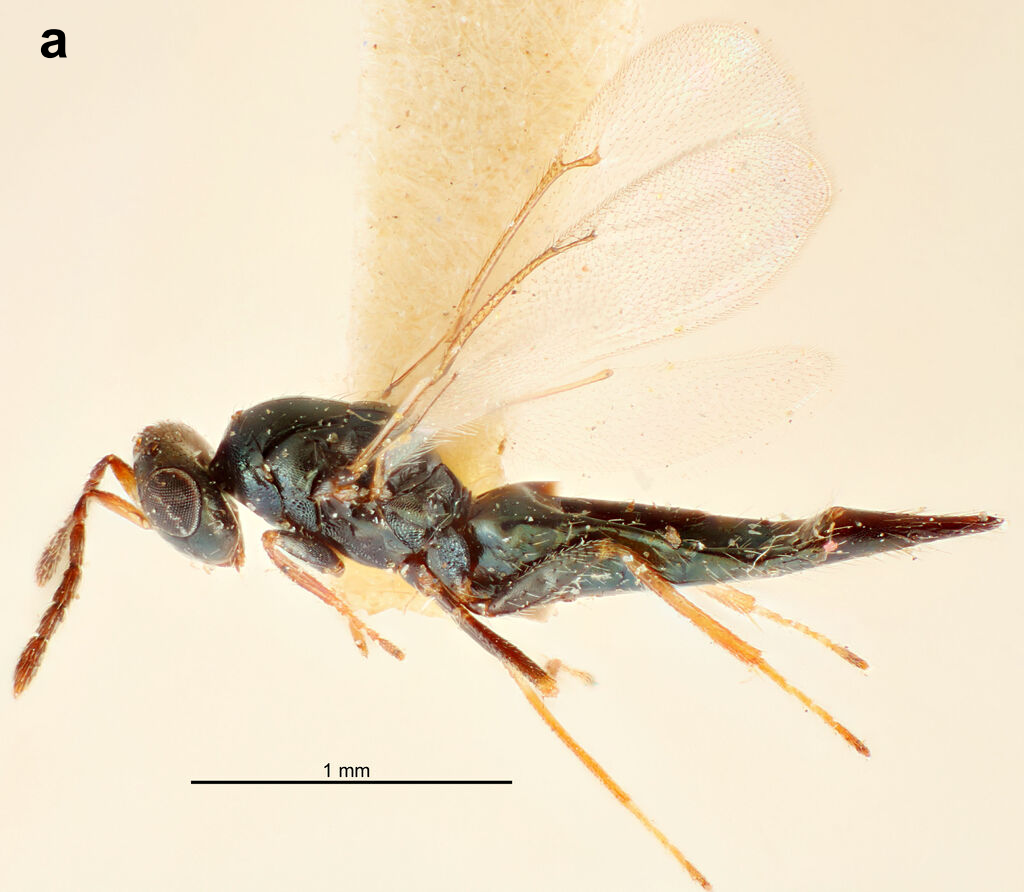
Holotype female, lateral.

**Figure 92b.**
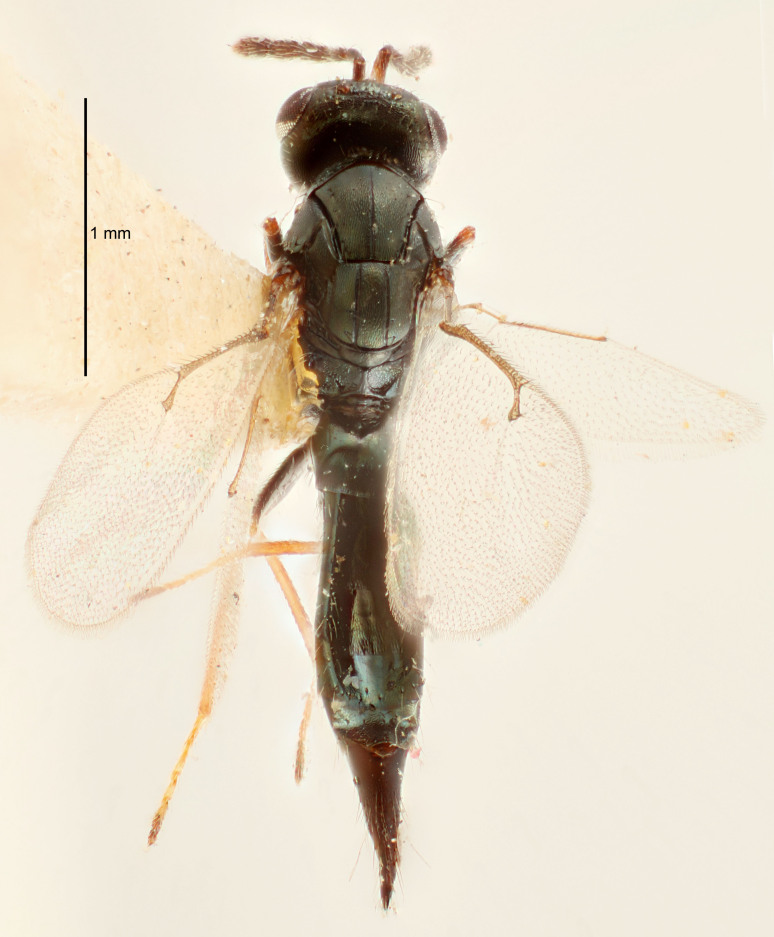
Holotype female, dorsal.

**Figure 93a.**
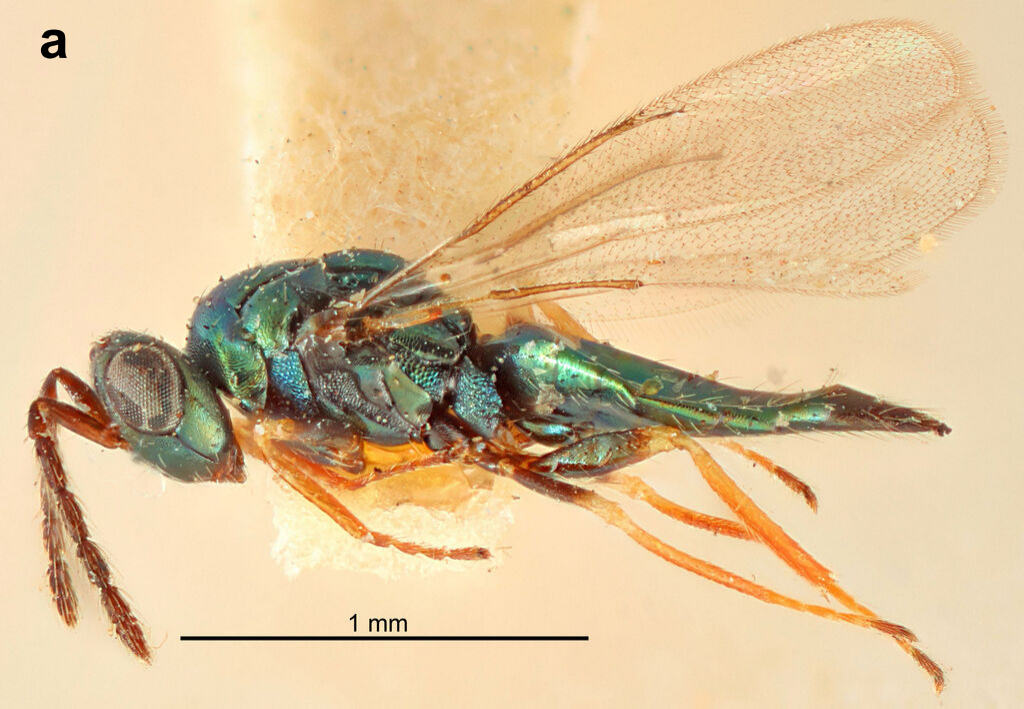
Holotype female, lateral.

**Figure 93b.**
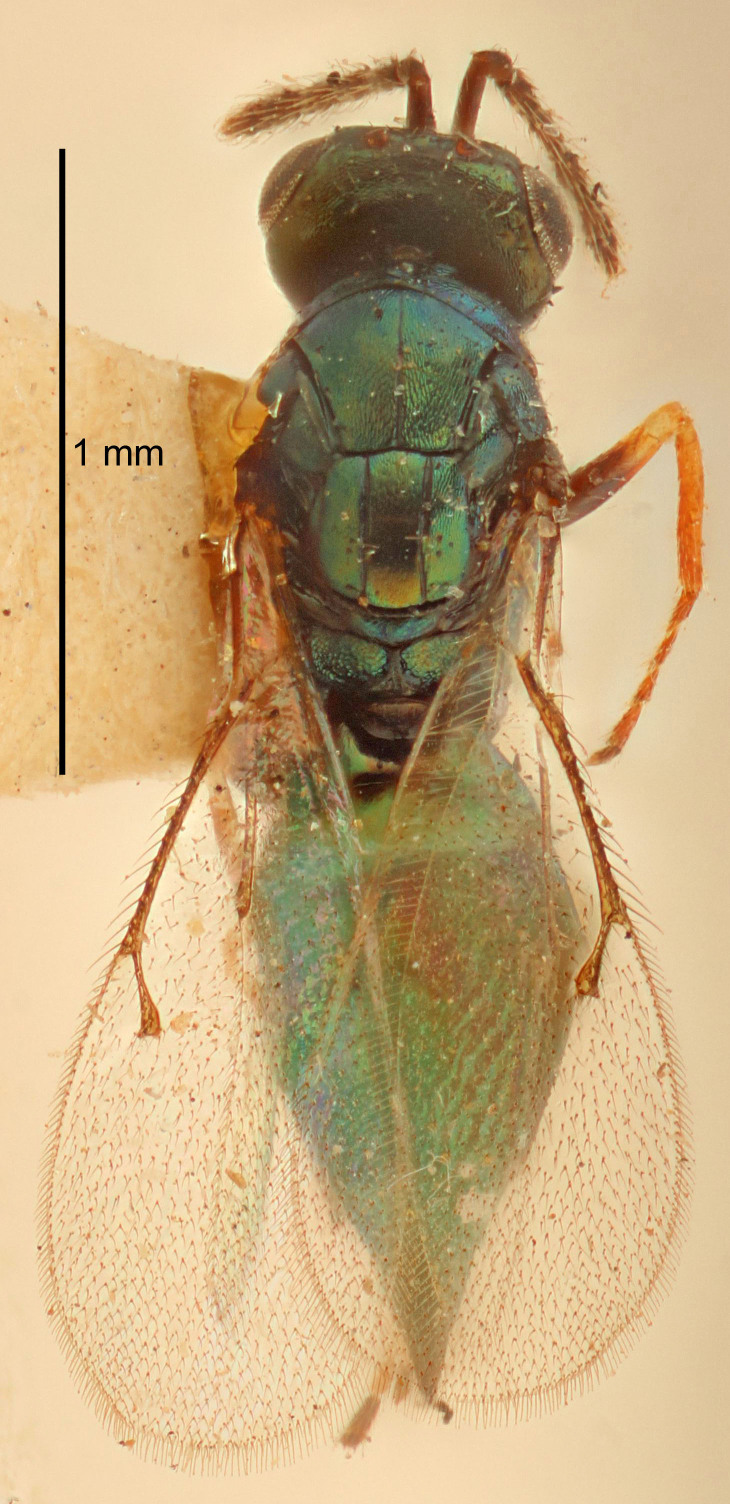
Holotype female, dorsal.

**Figure 93c.**
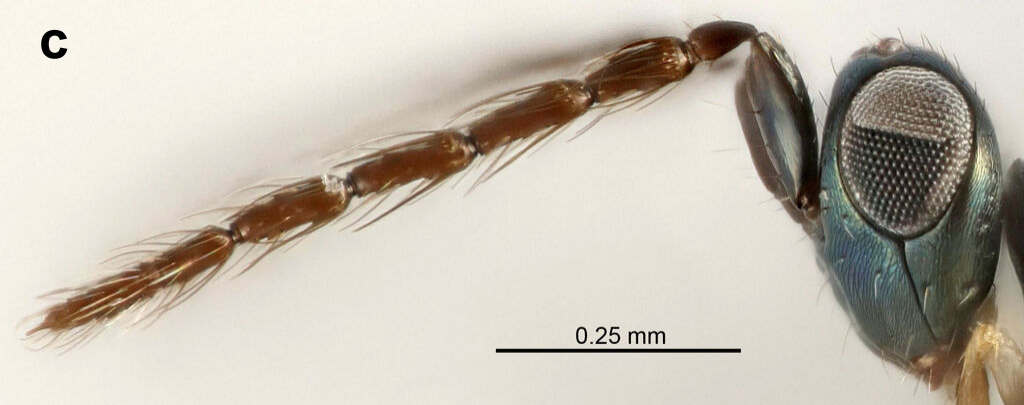
Nontype male, head and antenna lateral.

**Figure 94a.**
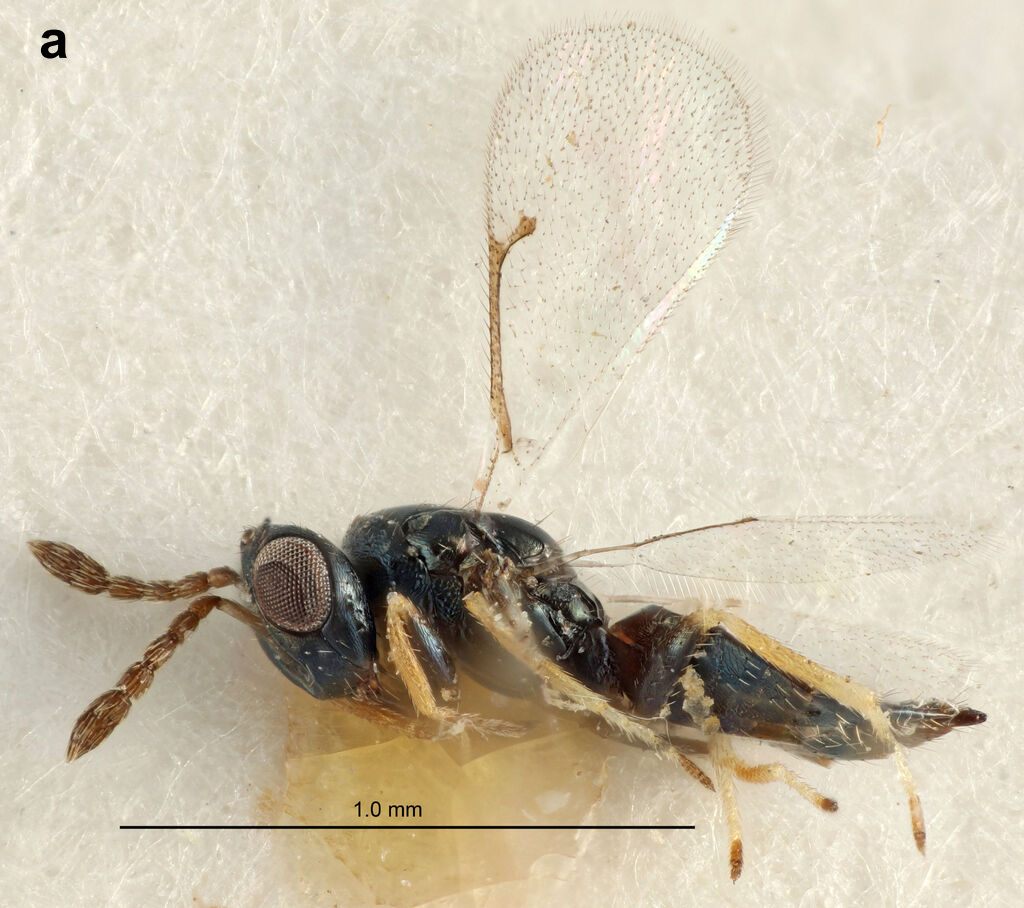
Paratype female, lateral.

**Figure 94b.**
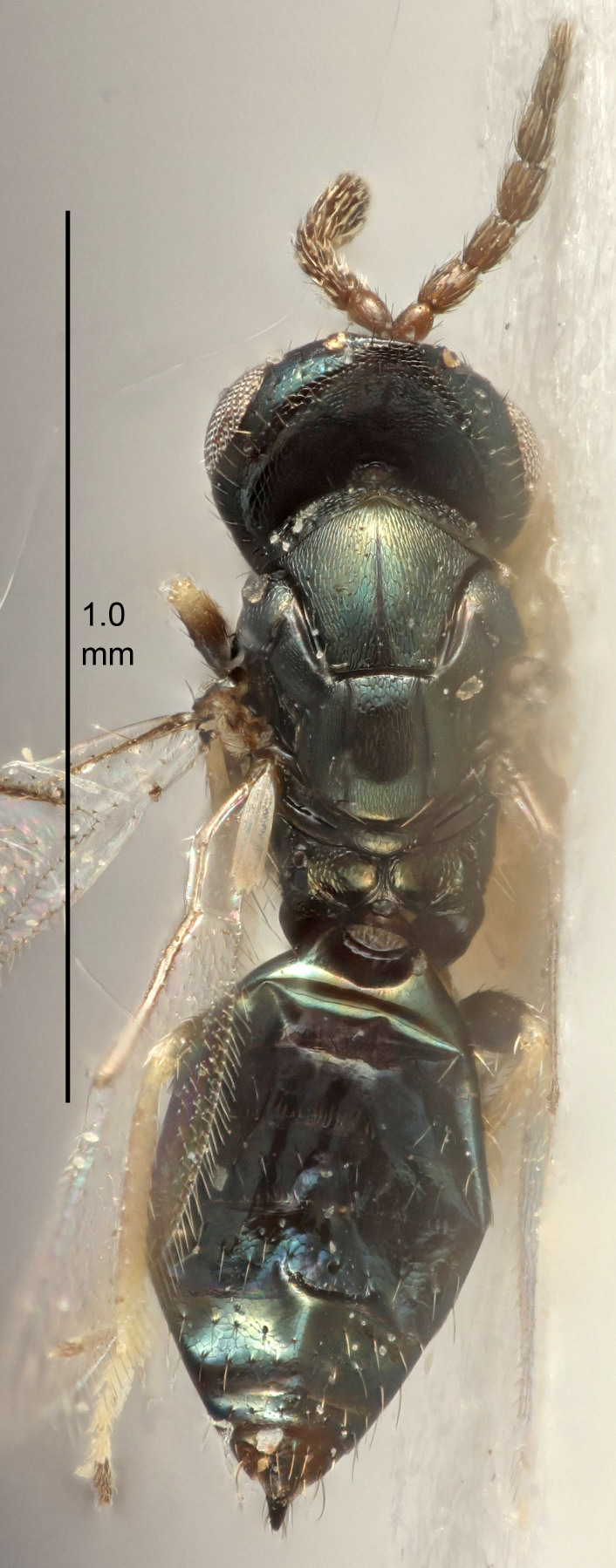
Paratype female, dorsal.

**Figure 95a.**
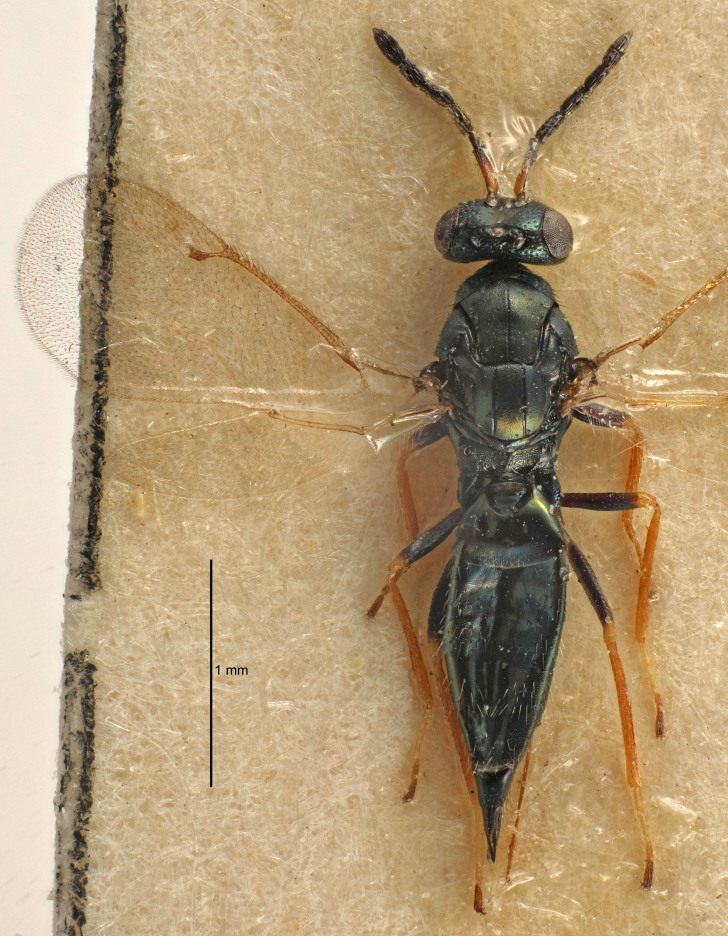
Nontype female, dorsal. Identified by J. Erdös.

**Figure 95b.**
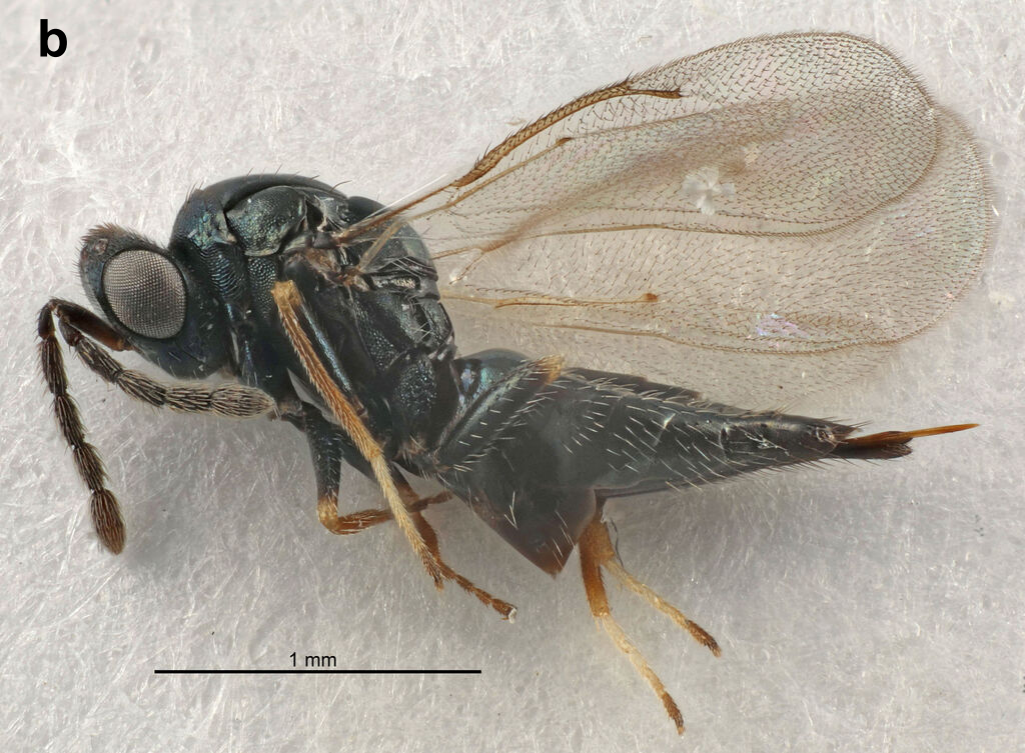
Nontype female, lateral.

**Figure 95c.**
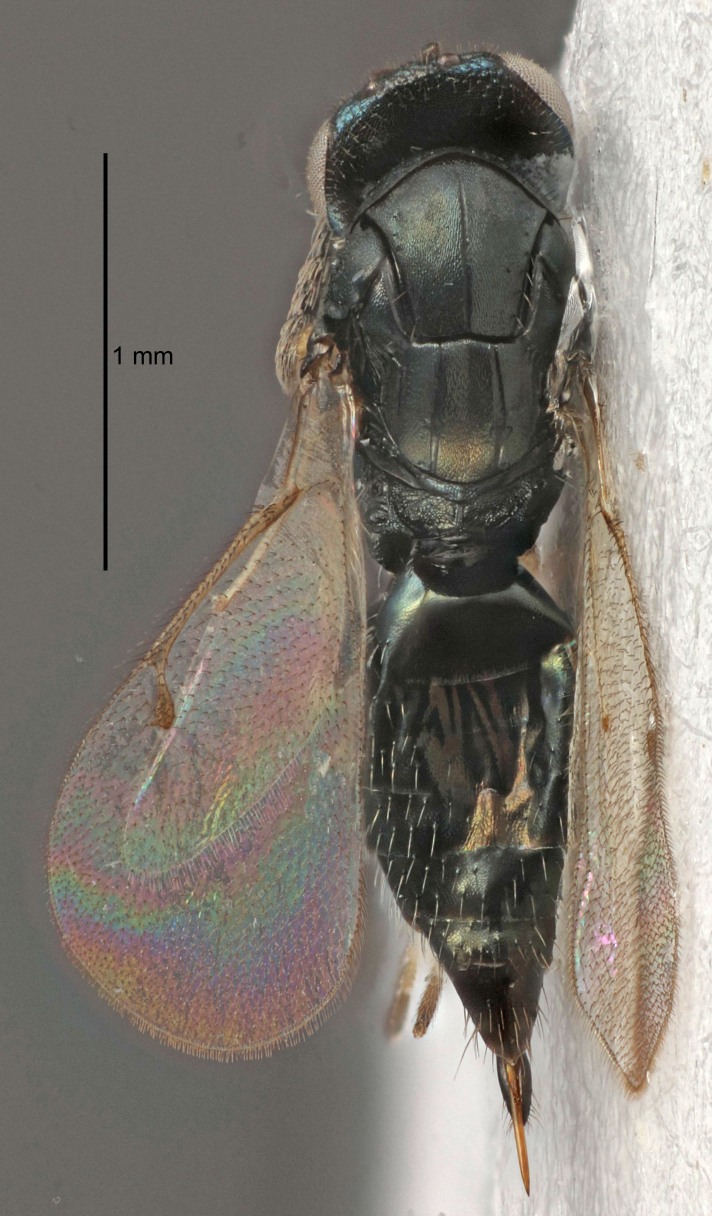
Nontype female, dorsal.

**Table 1.** *Tetrastichus* hosts

***Tetrastichus* species**	**Host**
*T. agrilocidus* Graham	*Agrilus* sp. (Coleoptera: Buprestidae), *Xylotrechus pantherinus* Savenius (Coleoptera: Cerambycidae) (Graham 1991)
*T. argei* sp. n.	Gregarious endoparasitoid on *Arge ustulata* (L.) (Hymenoptera: Argidae)
*T. atratulus* (Nees)	*Ptecticus tenebrifer* (Walker) (Diptera: Stratiomyiidae) (in Japan) (Graham 1991)
*T. brachyopae* Graham	Gregarious koinobiont endoparasitoid on *Brachyopa* spp. (Diptera: Syrphidae), clutch size varying from 7–18 specimens, with a strong female bias (van Eck et al. 2016). Recorded from *Brachyopa pilosa* Collin (Graham 1991), *B. bicolor* (Fallén) & *B. insensilis* Collin (van Eck et al. 2016)
*T. clito* (Walker)	*Cassida murraea* L., *C. rubiginosa* Müller, *C. deflorata* Suffrian (Graham 1991), *C. humeralis* Kraatz **new record**. All hosts are Coleoptera: Chrysomelidae
*T. coeruleus* (Nees)	*Crioceris asparagi* (Coleoptera: Chrysomelidae), emerges from host cocoons, but oviposits already in the host eggs (Graham 1991)
*T. crioceridis* Graham	*Crioceris duodecimpunctata* L. (Coleoptera: Chrysomelidae), egg-larval parasitoid (Graham 1983)
*T. cyprus* sp. n.	Reared from an unidentified Coleoptera and from an unidentified leaf miner on broad bean (*Vicia faba* L.)
*T. epilachnae* (Giard)	*Epilachna argus* (Geoffroy in Fourcroy), *E. chrysomelina* F., *Subcoccinella vigintiquattropunctata* L. (Coleoptera: Coccinellidae) (Graham 1991)
*T. halidayi* (Graham)	*Agropus ahrensi* Germar (Coleoptera: Chrysomelidae) (Graham 1991)
*T. heeringi* Delucchi	*Agrilus aurichalceus* Redtenbacher, *A. integerrimus* Ratzeburg, *A. viridis* (L.) (Coleoptera: Buprestidae) (Graham 1991)
*T. hylotomarum* (Bouché)	*Arge ochropus* (Gmelin), *A. pagana* (Panzer) (Hymenoptera: Argidae), *Athalia cordata* Audinet-Serville, *Cladius pectinicornis* (Geoffroy) (Hymenoptera: Tenthredinidae), parasitising host larvae and pupae (Graham 1991)
*T. julis* (Walker)	Lema (Oulema) spp. (Coleoptera: Chrysomelidae), gregarious endoparasitoid of host larva (Graham 1991)
*T. legionarius* Giraud	Gregarious endoparasitoid of larvae and pupae of *Lipara lucens* Meigen (Diptera: Chloropidae) (Graham 1991)
*T. leocrates* (Walker)	*Rhynchaenus alni* (L.) (Graham 1991), *R. testaceus* (Müller) (**new record**) (Coleoptera: Curculionidae)
*T. lyridice* (Walker)	*Plagiodera versicolora* (Laich.)? (Coleoptera: Chrysomelidae). This record is doubtful, needs checking (Graham 1991)
*T. macrops* (Graham)	Probably *Cis* spp. (Coleoptera: Cisiidae) in Polyporaceae (Graham 1991)
*T. melosomae* Graham	*Chrysomela vigintipunctata* (Scopoli) (Coleoptera: Chrysomelidae) (Graham 1991)
*T. miser* (Nees)	*Rhynchaenus alni* (L.), *R. fagi* (L.), *R. pilosus* (F.), *R. quercus* (F.), *R. salicis* (L.), *Ramphus oxyacanthae* (Marsham) (Coleoptera: Curculionidae) (Graham 1991)
*T. murcia* (Walker)	*Geosargus* sp. (Diptera: Stratiomyiidae), endoparasitoid emerging from pupa (Domenichini 1966)
*T. nymphae* sp. n.	Gregarious endoparasitoid on *Galerucella nymphaeae* (L.) (Coleoptera: Chrysomelidae)
*T. pilemostomae* Graham	*Pilemostoma fastuosa* (Schaller) (Coleoptera: Chrysomelidae) (Graham 1991)
*T. polyporinus* Askew	Possibly *Dacne* sp. (Askew 2007); *Triplax rufipes* Fabricius and *T. russica* (L.) **new records**. All hosts are Coleoptera: Erotylidae
*T. scardiae* sp. n.	*Scardia boletella* (Fabricius) (Lepidoptera: Tineidae)
*T. setifer* Thomson	*Lilioceris lilii* (Scopoli) (Gold et al. 2001) and *L. tibialis* (Villa) (Kenis et al. 2002) (Coleoptera: Chrysomelidae)
*T. solvae* Graham	*Xylomyia cabrerae* (Becker) (Diptera: Xylomyiidae) in twigs of *Euphorbia canariensis* (Graham 1991)
*T. telon* (Graham)	*Agrilus viridis* L. (Coleoptera: Buprestidae) (Domenichini 1966)
*T. temporalis* (Graham)	Associated with *Phalaris arundinacea* (Poaceae) (Graham 1991)
*T. ulmi* Erdös	*Scolytus rugulosus* (Müller) and *Leperisinus orni* Fuchs (Coleoptera: Scolytidae), *Agrilus* sp. and possibly *Anthaxia* sp. (Coleoptera: Buprestidae) (Graham 1991)

**Table 2.** Key to females of European species of *Tetrastichus*.

1.	Frons without a narrow median longitudinal carina below the median ocellus (Fig. 3a); hind coxae strongly reticulate to rugulose on the externo-dorsal surface (Fig. 3c)	**2**
-	Frons with a very narrow and distinct median longitudinal carina extending from between the toruli to near the median ocellus (Fig. 3b); hind coxae usually finely reticulate on the externo-dorsal surface (Fig. 3d)	**75**
2.	Head with mouth opening 2.0–2.2× malar space; mandibles (Fig. 3a) very large, with outer tooth falcate and separated by a wide gap from the two small inner teeth	**3**
-	Mouth opening 1.8–2.0× malar space in *coeruleus*, which has mandibles less large, head and mesosoma dark to bright blue or bluish-purple; in all the other species the mouth is at most 1.5× the malar space (usually hardly wider than the malar space)	**5**
3.	Gaster long ovate, about 1.8× as long as wide (Fig. 66c); ovipositor visible in dorsal view (Fig. 66c); cerci on gaster placed laterally and visible in dorsal view; body colour more dull (Fig. 66a, b, c)	***T. halidayi* (Graham)**
-	Gaster short ovate, 1.3–1.4× as long as wide (Figs 23b, 34b); ovipositor retracted and not visible in dorsal view (Figs 23b, 34b); cerci on gaster placed ventrally and not visible in dorsal view; body more metallic (Figs 23, 34)	**4**
4.	Antenna with scape 3.3× and antennal clava 2.7× as long as wide (Fig. 23a)	***T. cyprus* sp. n.**
-	Antenna with scape 3.1× and antennal clava 3.0× as long as wide (Fig. 34d)	***T. marcusgrahami* sp. n.**
5.	Eyes small (Fig. 87c), 0.9× as long as malar space; submedian grooves on mesoscutellum distinctly converging posteriorly (Fig. 87b); antenna in most specimens with F1 and F2 ± merged (Fig. 87a)	***T. scardiae* sp. n.**
-	Eye usually as long as or longer than malar space; submedian grooves on mesoscutellum usually ± parallel; antenna with distinct constriction between F1 and F2	**6**
6.	Antenna (Fig. 88b): clava with a very long, slightly tapering spicule, the clava itself much longer than F2+3; flagellum stout; funiculars subequal in length or differing only slightly, all less than twice as long as broad; scape virtually as long as an eye and reaching level of vertex; mouth opening 1.4–1.5× malar space	***T. setifer* Thomson**
-	Antennal clava usually with a relatively short spicule, if approaching the length seen in *T. setifer*, then clava is not or hardly longer than F2+3 and *either* the flagellum is less stout *or* the scape is longer than an eye and reaches above the vertex; width of mouth opening variable	**7**
7.	Width of mouth opening 1.3–2.0× malar space	.**8**
-	Width of mouth opening equal to or only slightly wider (at most 1.14×) than malar space	**18**
8.	Gaster 2.4–2.5× as long as wide (Fig. 65)	***T. gredius* sp. n.**
-	Gaster at most 2.1× as long as wide	**9**
9.	Ovipositor sheaths do not reach apex of gaster (as in Fig. 2b)	**10**
-	Ovipositor sheaths reach apex of gaster (as in Fig. 2a)	**12**
10.	Body bright metallic blue to bluish-purple (Fig. 20); antennal flagellum long (Fig. 20c), F3 1.3× as long as wide and clava (incl. spicule) 2.7–3.1× as long as wide	***T. coeruleus* (Nees)**
-	Body black with very weak metallic blue tinges (Figs 80, 86); antennal flagellum short, for example, F3 1.0–1.1× as long as wide and clava (incl. spicule) 2.2–2.5× as long as wide	**11**
11.	Head 2.5× as long as wide in dorsal view (Fig. 86b); pedicel+flagellum 0.9× as long as width of mesosoma; antenna (Fig. 86a) with F2 1.3× and clava 2.5× as long as wide and F1 0.9× as long as F2	***T. polyporinus* Askew**
-	Head 2.2× as long as wide in dorsal view (Fig. 80b); pedicel+flagellum 1.1× as long as width of mesosoma; antenna (Fig. 80a) with F2 1.1× and clava 2.2× as long as wide and F1 1.1× as long as F2	***T. minius* sp. n.**
12.	Antennae (e.g. Fig. 71b) with clava more acute, spicule relatively long and conspicuous; scape 1.0–1.2× length of eye, reaching to or above vertex	**13**
-	Antennae with clava subobtuse, spicule relatively short and inconspicuous (e.g. Fig. 59c); scape 0.9–1.0× length of eye, not reaching above vertex	**16**
13.	Antenna (Fig. 85a) with pedicel+flagellum about 1.2× width of mesoscutum, F1 1.6× as long as wide	***T. perkinsorum* Graham**
-	Antenna with pedicel+flagellum 1.4–1.5× width of mesoscutum, F1 2.0–2.2× as long as wide	**14**
14.	POL 2.2× OOL; fore wing with marginal vein 2.8× stigmal vein	***T. elodius* sp. n.**
-	POL 1.5–1.6× OOL; fore wing with marginal vein 3.2–3.5× stigmal vein	**15**
15.	Mesoscutellum with enclosed area between submedian grooves 2.7× as long as wide and distance between submedian grooves 1.5× distance between submedian grooves and sublateral grooves (Fig. 71c); fore wing with marginal vein 3.5× stigmal vein	***T. julis* (Walker)**
-	Mesoscutellum with enclosed area between submedian grooves 2.5× as long as wide and distance between submedian grooves 1.7× distance between submedian grooves and sublateral grooves (Fig. 70b); fore wing with marginal vein 3.2× stigmal vein	***T. inscitus* sp. n.**
16.	Antenna (Fig. 90) with F3 2.0–2.35× as long as wide; clava (incl. spicule) 0.8× as long as F2+F3	***T. sodalis* Graham**
-	Antenna (Figs 59c, 72a) with F3 1.4-1.8× as long as wide; clava (incl. spicule) 1.0–1.1× as long as F2+F3	**17**
17.	Flagellum longer (Fig. 72a), F2 2.0–2.1× and clava (incl. spicule) 3.6–3.8× as long as wide	***T. lanius* sp. n.**
-	Flagellum shorter (Fig. 59c), F2 1.5–1.8× and clava (incl. spicule) 2.8–2.9× as long as wide	***T. crioceridis* Graham**
18.	Eyes usually with long, conspicuous pubescence (Figs 5c, 7d), length of the setae 0.4–0.8× OD; setae on hind margin of pronotum and adnotaular setae relatively long and suberect (e.g. Fig. 7a); gaster short ovate to subcircular (e.g. Fig. 5b); body black non-metallic or with weak metallic tinges (Figs. 4–14)	**19**
-	Eyes with short and inconspicuous pubescence (e.g. Fig. 3a), length of the setae 0.2–0.35× OD; setae on hind margin of pronotum and adnotaular setae usually shorter, weaker and reclinate; shape of gaster and body colour variable	**29**
19.	Antenna (Fig. 12) with pedicel 1.35–1.5× length of F1; funicle stout, funiculars short, F1 and F2 1.1–1.25× as long as broad; mid-lobe of mesoscutum without a median line or with line indicated only near the mesoscutellum	***T. solvae* Graham**
-	Antenna with pedicel not or only slightly longer than F1; funicle stout or slender; F1 1.5–2.5×, F2 1.4–2.5×, as long as broad; mid-lobe of mesoscutum with median line distinct over at least posterior ½, sometimes complete (weak or absent in some specimens of *T. brachyopae* that differs from all other species in this group in having submedian grooves of mesoscutellum equidistant from each other and from sublateral grooves)	**20**
20.	Gaster (Fig. 11b, c) with ovipositor sheaths projecting distinctly beyond apex of Gt_7_, length of projecting part about equal to length of hind basitarsus; setae on eyes very long, 0.8× OD; distance between posterior ocelli relatively short, POL 1.5–1.6× OOL	***T. murcia* (Walker)**
-	Gaster with ovipositor sheaths not projecting beyond apex of Gt_7_ or projecting less than in alternate; setae on eyes shorter, 0.4–0.6× OD; POL 1.9–2.4× OOL	**21**
21.	Submedian grooves of mesoscutellum equidistant from each other and from sublateral grooves (Fig. 6a); gaster with ovipositor sheaths projecting beyond apex of gaster (Fig. 6a, b); antennal funicle (Fig. 6c) rather stout, its segments relatively short, F1 1.5–1.7×, F2 1.4–1.5× as long as broad	***T. brachyopae* Graham**
-	Submedian grooves of mesoscutellum nearer to sublateral grooves than to each other; gaster with ovipositor sheaths not or hardly projecting beyond apex of gaster; antennal funicle less stout, funiculars on average longer, F1 1.7–2.5×, F2 1.7–2.5× as long as broad (antenna of *T. antonjanssoni* short, but with distance between submedian grooves 1.7× the distance between submedian and sublateral grooves)	**22**
22.	Distance between submedian grooves 2.0× the distance between submedian and sublateral grooves (Fig. 14b); antennal clava (incl. spicule) 3.3× as long as wide (Fig. 14a)	***T. tartus* sp. n.**
-	Distance between submedian grooves 1.2–1.9× the distance between submedian and sublateral grooves; antennal clava (incl.spicule) 2.1–3.0× as long as wide	**23**
23.	Antennal flagellum longer (Fig. 8a), for example, F3 1.9× as long as wide	***T. intruitus* sp. n.**
-	Antennal flagellum shorter, F3 1.1–1.4× as long as wide	**24**
24.	Antennal clava (incl. spicule) 2.2–2.4× as long as wide	**25**
-	Antennal clava (incl. spicule) 2.7–3.2× as long as wide	**27**
25.	Antenna short (Fig. 4a) with F1 1.6×, F2 1.4×, F3 1.1× as long as wide	***T. antonjanssoni* sp. n.**
-	Antenna long with F1 2.0–2.3×, F2 1.7–1.8×, F3 1.2–1.3× as long as wide	**26**
26.	Antenna (Fig. 5a): pedicel+flagellum 1.15–1.35× width of mesoscutum, F1 2.0–2.5× and F2 1.6–2.0× as long as wide; apex of ovipositor sheaths does not reach apex of gaster	***T. atratulus* (Nees)**
-	Antenna (Fig. 7a): pedicel+flagellum 1.0–1.05× width of mesoscutum, F1 1.7–2.0× and F2 1.2–1.6× as long as wide; apex of ovipositor sheaths reach apex of gaster or slightly beyond apex	***T. dasyops* Graham**
27.	Antennal clava 3.4× as long as wide (Fig. 10a); POL/OOL = 2.4	***T. mixtus* sp. n.**
-	Antennal clava 2.7–2.8× as long as wide; POL/OOL = 1.9–2.2	**28**
28.	Ovipositor sheaths protruding beyond apex of gaster, visible in dorsal view (Fig. 13b)	***T. tacitus* sp. n.**
-	Ovipositor sheaths not protruding beyond apex of gaster, not visible in dorsal view (Fig. 9b)	***T. lacustrinus* sp. n.**
29.	Ovipositor sheaths retracted and do not reach apex of gaster (as in Fig. 2b); gaster at most 1.6× as long as broad, usually subcircular sometimes short ovate; not longer, usually broader than mesosoma; eyes relatively small, usually separated by 1.6–1.8× their own length; antennal scape virtually as long as, or even slightly longer than an eye (in specimens having undistorted head the scape sometimes reaches slightly above the vertex); most species are strongly metallic (e.g. Fig. 27)	**30**
-	Ovipositor sheaths reach apex of gaster or even extending slightly beyond it, so that their tips are visible in dorsal view (as in Fig. 2a); gaster variable in shape, rarely subcircular, usually ovate to lanceolate; eyes usually relatively larger; antennal scape usually shorter than an eye and not reaching above the vertex; species not as strongly metallic as in alternate	**50**
30.	Antenna (Fig. 31a) with F1 0.8× as long as F2 and only very slightly longer than the pedicel	***T. inaequalis* Graham**
-	Antennae with F1 0.9–1.2× as long as F2 and at least slightly longer than the pedicel	**31**
31.	Antennal flagellum very long and narrow (Fig. 91e), 1.6× as long as width of mesoscutum; ventral margin of scape with more than ten setae	***T. tachos* (Walker)**
-	Antennal flagellum 1.0–1.4× as long as width of mesoscutum; ventral margin of scape with fewer setae, usually at most six	**32**
32.	Mid and hind tibiae predominantly to completely dark brown to black (Fig. 28a, b)	***T. hylotomarum* (Bouché)**
-	Mid and hind tibiae predominantly to completely yellowish-brown to pale brown	**33**
33.	Mesoscutellum with enclosed space between submedian grooves 2.2× as long as wide (Fig. 24b)	***T. erinus* sp. n.**
-	Mesoscutellum with enclosed space between submedian grooves 2.5–3.8× as long as wide	**34**
34.	Mesoscutellum with enclosed space between submedian grooves 3.3–3.8× as long as wide	**35**
-	Mesoscutellum with enclosed space between submedian grooves 2.5–3.0× as long as wide	**38**
35.	Antenna (Fig. 35a) more stout, length/width F1, F2, F2, clava: 1.7, 1.6, 1.3, 2.5; mesoscutellum with enclosed space between submedian grooves 3.8× as long as wide (Fig. 35b)	***T. splendens* sp. n.**
-	Antenna more slender, length/width F1, F2, F2, clava: 2.0–2.3, 1.9–2.0, 1.7–1.8, 3.2–3.8; mesoscutellum with enclosed space between submedian grooves 3.3–3.4× as long as wide	**36**
36.	Antenna (Fig. 33a) shorter, F1 2.0× and clava 3.2× as long as wide	***T. johnnoyesi* sp. n.**
-	Antenna longer, F1 2.2–2.3× and clava 3.8–4.0× as long as wide	**37**
37.	Mesoscutellum 0.9× as long as wide with enclosed space between submedian grooves 3.3× as long as wide (Fig. 22b); fore wing with marginal vein 3.1× as long as stigmal vein	***T. cumulus* sp. n.**
-	Mesoscutellum 1.1× as long as wide with enclosed space between submedian grooves 3.1× as long as wide (Fig. 26b); fore wing with marginal vein 2.7× as long as stigmal vein	***T. flaccius* sp. n.**
38.	Malar space 1.0× height of eye	**39**
-	Malar space 0.7–0.9× height of eye	**40**
39.	POL/OOL = 1.6; F1 2.4× as long as wide (Fig. 37a)	***T. suecus* sp. n.**
-	POL/OOL = 2.1; F1 1.8× as long as wide (Fig. 25a)	***T. evexus* sp. n.**
40.	Antennal clava (incl. spicule) 3.0–3.4× as long as wide	**41**
-	Antennal clava (incl. spicule) 3.6–4.8× as long as wide	**44**
41.	Mesoscutellum with submedian grooves diverging towards posterior part (Fig. 29b)	***T. iasi* sp. n.**
-	Mesoscutellum with submedian grooves parallel (e.g. Fig. 27a)	**42**
42.	Scape 3.4–3.5× as long as wide, completely to predominantly dark and metallic (Fig. 15a)	***T. argei* sp. n.**
-	Scape 4.2–4.4× as long as wide, completely to predominantly yellowish-brown (Figs 27b, 30a)	**43**
43.	Mesoscutellum 1.1× as long as wide (Fig. 27a); mesoscutum and mesoscutellum with same colour (Fig. 27a)	***T. helviscapus* Graham**
-	Mesoscutellum 0.8× as long as wide (Fig. 30b); mesoscutum bluish and mesoscutellum greenish (Fig. 30b)	***T. illydris* sp. n.**
44.	Antennal clava 3.4–3.6× (3.4× in holotype, Fig. 19a) as long as wide	***T. coelarchus* Graham**
-	Antennal clava at least 3.8× as long as wide	**45**
45.	Antennal clava 3.8–4.1× as long as wide	**46**
-	Antennal clava 4.4–4.8× as long as wide	**49**
46.	Hind coxa with a strong and complete carina along posterior margin (Fig. 21c)	***T. cosidis* sp. n.**
-	Hind coxa with strong and irregular sculpture, but not a complete carina along posterior margin	**47**
47.	Antenna (Fig. 16a) with F2 1.9× as long as wide; ratio POL/OOL = 2.4	***T. asilis* sp. n.**
-	Antenna (Figs 18a, 32a) with F2 2.1–2.5× as long as wide; ratio POL/OOL = 1.9–2.1	**48**
48.	Antenna (Fig. 32a): F1 1.1× as wide and 2.0× as long as pedicel, F2 2.5× as long as wide; ratio POL/OOL = 2.1	***T. incanus* sp. n.**
-	Antenna (Fig. 18a): F1 1.0× as wide and 1.3× as long as pedicel, F2 2.1× as long as wide; ratio POL/OOL = 1.9	***T. calmius* sp. n.**
49.	Antennal clava longer and more slender (Fig. 17a), 4.6–4.8× as long as wide, with relatively weak constriction between C1 and C2	***T. brevicalcar Graham***
-	Antennal clava shorter and more stout (Fig. 36a), 4.4× as long as wide, with a strong constriction between C1 and C2	***T. sti* sp. n.**
50.	Gaster with last tergite 1.9–2.5× as long as broad; the gaster itself acuminate (Fig. 92), 2.9–5.0× as long as broad, 1.4–1.6× length of head+mesosoma, distinctly narrower than mesosoma; body bronze-black with, at most, some very weak greenish or bluish reflections (Fig. 92); antenna (Fig. 92a) with clava (incl. spicule) nearly 3× as long as broad, without constriction between C1and C2	***T. telon* (Graham)**
-	Gaster with last tergite very rarely 1.9× as long as broad, usually at most as long as broad, but if long and the gaster itself is almost as elongate as in *telon*, then either the body is brighter green to blue *or* the antennal clava is only about twice as long as broad	**51**
51.	Gaster lanceolate, at least slightly longer than head+mesosoma (Figs 73b, 93b); antennae with scape as long as or longer than an eye, in specimens with undistorted head reaching at least slightly above the vertex; pedicel 2.1–2.5× as long as broad; flagellum slender, at most slightly stouter than the pedicel; F3 1.9–2.8× as long as broad; clava (incl. spicule) 3.6–4× as long as broad, usually at least slightly stouter than F2+F3; malar space 0.8–1.0× length of eye; head at most 2.2× as broad as long; temples distinct, rounded; body relatively bright metallic green to blue (Figs 73, 93)	**52**
-	Either the gaster is ovate and about as long as mesosoma, *or* the antennal scape is slightly shorter than an eye *or* does not reach vertex, the pedicel is relatively shorter and the flagellum is less slender; antennal clava (incl. spicule) usually at most 3× as long as broad; malar space usually shorter; head usually more strongly transverse and with temples very short; body colour sometimes with different colour/shine	**53**
52.	Gaster (Fig. 73b) 2.6–3.3× as long as broad and nearly twice the length of mesosoma, last tergite 1.5–1.9× as long as broad; antenna (Fig. 73a) with scape somewhat longer than an eye; funiculars more elongate, F1 4.1–4.3×, F2 3.2–3.9×, F3 2.8–3.1× as long as wide; head, pronotum, mesoscutum and mesoscutellum duller, their sculpture slightly stronger; body colour blue (Fig. 73b)	***T. legionarius* Giraud**
-	Gaster (Fig. 93b) 1.9–2.3× as long as broad, less than twice as long as mesosoma; last tergite not or hardly longer than broad; antenna (Fig. 93a) with scape not or hardly longer than an eye, funiculars shorter, F1 2.6–2.9×, F2 2.1–2.8×, F3 1.9–2.4× as long as wide; head, pronotum, mesoscutum and mesoscutellum slightly more shiny, their sculpture slightly more delicate; body colour usually more greenish (Fig. 93)	***T. temporalis* (Graham)**
53.	Antennal clava (incl. spicule) distinctly to much shorter than F2+F3, without a constriction between C1and C2, obtuse or bluntly pointed; gaster at least slightly, usually distinctly, longer than head+mesosoma	**54**
-	*Either* antennal clava different (not or hardly shorter than F2+F3, *or* with a distinct constriction between C1and C2) *or* gaster at most as long as head+mesosoma	**58**
54.	Eyes larger, separated by 1.1–1.25× their length; temples virtually nil (Fig. 78b); antennal scape mainly to wholly and tibiae, yellow or yellowish-testaceous (Fig. 78a). Parasite of *Cis* on fungi	***T. macrops* (Graham)**
-	Eyes smaller, separated by 1.3–1.4× their length; temples 0.07–0.15× length of eyes; antennal scape and/or tibiae sometimes more or less infuscate. Parasites of Buprestidae, Scolytidae and Cerambycidae, or host unknown	**55**
55.	Antenna (Fig. 95b) with flagellum shorter and more stout, clava 2.0× as long as wide	***T. ulmi* Erdös**
-	Antenna with flagellum longer and more slender, clava 2.7–3.4× as long as wide	**56**
56.	Ratio POL/OOL = 2.3; antenna (Fig. 61a) with F1 2.4× and F2 2.1× as long as wide	***T. doczkali* sp. n.**
-	Ratio POL/OOL = 1.8–1.9; antenna (Figs 56a, 60b) with F1 2.6–2.9× and F2 2.6–3.0× as long as wide	**57**
57.	Antenna (Fig. 56a) with F1 2.9×, F2 3.0×, F3 2.3, clava 3.1× as long as wide; gaster longer (Fig. 56b), about 1.8× as long as mesosoma	***T. agrilocidus* Graham**
-	Antenna (Fig. 60b) with F1 2.6×, F2 2.6×, F3 1.9, clava 3.4× as long as wide; gaster shorter (Fig. 60b), 1.4× as long as mesosoma	***T. delvarei* sp. n.**
58.	Species with following combination of characters: antenna (Fig. 67a) with Fl 3.0–3.2× as long as broad and 0.7–0.8× length of clava (incl. spicule), the latter 0.8–0.9× as long as F2+F3; flagellum proximally hardly stouter than the pedicel; gaster (Fig. 67b) at most twice as long as broad (temples short but distinct, about 0.15× length of eyes)	***T. heeringi* Delucchi**
-	Antenna either with F1 relatively shorter *or* with clava (incl. spicule) almost as long as F2+F3 *or* with flagellum proximally distinctly stouter than the pedicel; gaster sometimes more elongate	**59**
59.	Gaster longer, at least 2.2× as long as broad	**60**
-	Gaster shorter, at most 2× as long as broad	**65**
60.	Antenna (Fig. 76a) with sensilla on F1–F3 sparse, in one (sometimes irregular) row on each funicular; clava without a constriction between C1and C2	***T. leptosoma* Graham**
-	Antenna with sensilla of funicle less sparse, in two partly overlapping rows on at least F1 and F2; clava with a distinct constriction between C1and C2	**61**
61.	Flagellum more slender (Fig. 68a), for example, F3 2.8× and clava (incl. spicule) 5.0× as long as wide	***T. heterus* Graham**
-	Flagellum more stout, for example, F3 1.6–2.2× and clava (incl. spicule) 2.6–4.5× as long as wide	**62**
62.	Ocellar triangle encircled by groove (Fig. 83c, d); vertex with numerous piliferous punctures (Fig. 83c, d); antennal clava with apex blunt (Fig. 83a); F1 2.2–2.4× as long as wide; funiculars more hairy and with more sensilla, sensilla usually in three rows (Fig. 83a) (large species, 2.4–3.1 mm)	***T. pachycerus* Graham**
-	Ocellar triangle not encircled by groove; vertex with few piliferous punctures or none; antennal clava with apex pointed; F1 1.8–2.2× as long as wide; funiculars less hairy and with fewer sensilla, sensilla usually in two rows	**63**
63.	Scape (Fig. 64a) 3.3× as long as wide	***T. fadus* sp. n.**
-	Scape 4.0–4.2× as long as wide	**64**
64.	Antennal scape, tibiae and wing veins black to dark brown (Fig. 84); head 2.14× as wide as long (dorsal view)	***T. paululus* Graham**
-	Antennal scape, tibiae and wing mainly yellowish-brown and wing veins yellowish-white (Fig. 58) ; head 2.30× as wide as long (dorsal view)	***T. broncus* sp. n.**
65.	Species with following combination of characters: head 2.1× as broad as long (dorsal view), with temples distinct; submedian grooves of mesoscutellum 1.8–2.0× as far apart as their distance from sublateral grooves (Fig. 94b); costal cell of fore wing narrow, 11–12.5× as long as broad; antennal scape mainly yellow; propodeum (Fig. 94b) shiny, its sculpture rather weak, sometimes partly smooth; gaster (Fig. 94b) 1.7–2.0× as long as broad	***T. theoi* Graham**
-	Not with above combination of characters: either head more transverse with temples shorter *or* submedian grooves of mesoscutellum less far apart *or* costal cell of fore wing broader *or* antennal scape mainly to wholly black *or* propodeum distinctly reticulate	**66**
66.	Spicule on antennal clava 0.5× as long as C3 (Fig. 69d), scape 1.06× as long as an eye, reaching nearly to level of vertex, clava (incl. spicule) as long as F2+F3	***T. ilithyia* (Walker)**
-	Spicule on antennal clava usually 0.2–0.3× as long as C3; if longer, then antennal scape shorter than an eye, not reaching vertex; clava (incl. spicule) sometimes shorter than F2+F3	**67**
67.	Fore wing with costal cell very narrow, 13–17× as long as broad (Fig. 79a); antenna (Fig. 79a) with claval spicule about 0.5× length of C3, scape yellow; body bright green to blue (Fig. 79)	***T. melasomae* Graham**
-	Fore wing with costal cell broader; antenna with claval spicule relatively shorter, at most 0.4× length of C3, scape often black or partly infuscate; body sometimes dark blue or olive-greenish	**68**
68.	Antenna (Fig. 77e) with pedicel+flagellum 1.3–1.4× width of mesoscutum; setae of flagellum long and standing out at a greater angle; clava with a distinct constriction between C1 and C2 and 0.9× as long as F2+F3; scape more or less testaceous; gaster (Fig. 77a, c) long-ovate, 1.6–2.0× as long as broad, usually slightly longer than mesosoma; last tergite usually as long as broad, occasionally slightly transverse	***T. lyridice* (Walker)**
-	Antennae with pedicel+flagellum 1.1–1.3× width of mesoscutum; setae of flagellum standing out less strongly and tending to be shorter; clava either with a very weak constriction between C1and C2 or else at least as long as F2+F3; scape often black/dark brown; gaster subcircular to ovate, 1.2–1.9× as long as broad, shorter than to slightly longer than mesosoma; last tergite usually at least slightly broader than long	**69**
69.	Gaster 1.8–1.9× as long as wide, nearly or just as long as head+mesosoma, strongly acute at apex, Gt_7_ 0.8–1.0× as long as broad	**70**
-	Gaster not more than 1.6× as long as broad, not longer than mesosoma, except in some *sinope* which has gaster only slightly longer than mesosoma and Gt_7_ 0.4–0.6× as long as wide)	**71**
70.	Scape 0.9–1.0× as long as eye; flagellum longer (Fig. 54a), for example, F1 1.9–2.3× as long as wide	***T. acutiusculus* Graham**
-	Scape 0.8× as long as eye; flagellum shorter (Fig. 75b), for example, F1 1.5–1.6× as long as wide	***T. leocrates* (Walker)**
71.	Distance between submedian grooves 1.1–1.2× the distance between submedian and sublateral grooves (Fig. 89a, c); hind tibia and antennal scape dark brown to black (Fig. 89b)	***T. sinope* (Walker)**
-	Distance between submedian grooves 1.4–1.8× the distance between submedian and sublateral grooves; hind tibia usually yellowish-brown, very seldom dark, colour of scape yellowish-brown to black	**72**
72.	POL 2.2× OOL	**73**
-	POL 1.7–1.9× OOL	**74**
73.	Marginal vein 3.3× as long as stigmal vein; antennal clava 3.6× as long as wide (Fig. 57a)	***T. ballotus* sp. n.**
-	Marginal vein 2.8× as long as stigmal vein; antennal clava 3.3× as long as wide (Fig. 82a)	***T. nataliedaleskeyae* sp. n.**
74.	Antenna (Fig. 81a, b) with F1 2.0×, F3 1.7× and clava 3.0× as long as wide; mesoscutellum with distance between submedian grooves 1.8× distance between submedian and sublateral grooves (Fig. 81a, c)	***T. miser* Walker**
-	Antenna (Fig. 55a) with F1 2.3×, F3 2.0× and clava 3.3× as long as wide; mesoscutellum with distance between submedian grooves 1.4× distance between submedian and sublateral grooves (Fig. 55b)	***T. agonus* sp. n.**
75.	Frons with median carina wide with sides parallel (Fig. 63c)	***T. enodis* sp. n.**
-	Frons with median carina narrow with sides converging towards median ocellus	**76**
76.	Antenna (Fig. 74a) with pedicel yellowish-brown, same colour as scape; body (Fig. 74) black with very weak bronze and bluish tinges; mesosoma dorsally (except propodeum) strongly shiny, with very fine reticulation (Fig. 74b); median line of mesoscutum very weak or missing (Fig. 74b)	***T. leionotus* Graham**
-	Antenna with pedicel brown to black, body colour variable; mesosoma dorsally with more distinct reticulation; median line of mesoscutum variable	**77**
77.	Ovipositor sheaths reach beyond apex of Gt_7_ and are visible in dorsal view (as in Fig. 2a)	**78**
-	Ovipositor sheaths do not reach apex of Gt_7_ and are not visible in dorsal view (as in Fig. 2b)	**82**
78.	Hind coxa with a strong and sharp carina along posterior margin (as in Fig. 21c)	***T. argutus* sp. n.**
-	Hind coxa without a strong and sharp carina along posterior margin	**79**
79.	Mesoscutellum ± flattened (Fig. 51a, b), 1.0× as long as wide, distance between submedian grooves 2.1× the distance between submedian and sublateral grooves	***T. nymphae* sp. n.**
-	Mesoscutellum more convex, 0.8× as long as wide, distance between submedian grooves 1.2–1.9× the distance between submedian and sublateral grooves	**80**
80.	Antenna (Fig. 52a) 1.5× as long as width of mesoscutum and clava 4.5× as long as wide; fore wing with marginal vein 3.3× as long as stigmal vein	***T. pilemostomae* Graham**
-	Antenna 1.7–1.8× as long as width of mesoscutum and clava 3.7–3.8× as long as wide; fore wing with marginal vein 2.5–2.9× as long as stigmal vein	**81**
81.	Mesoscutellum with distance between submedian grooves 1.2× the distance between submedian and sublateral grooves and with enclosed area between submedian grooves 2.3× as long as wide (Fig. 43b)	***T. decrescens* Graham**
-	Mesoscutellum with distance between submedian grooves 1.6× the distance between submedian and sublateral grooves and with enclosed area between submedian grooves 1.9× as long as wide (Fig. 44b)	***T. elanus* sp. n.**
82.	Body bright metallic blue to bluish-green (Fig. 79); malar space 0.5–0.55× length of eye	***T. melasomae* Graham**
-	Body dull, at most, with weak metallic tinges; malar space 0.72–0.86× length of eye	**83**
83.	Antenna short (Fig. 42b), at most, 1.1× as long as width of mesoscutum	**84**
-	Antenna at least 1.2× as long as width of mesoscutum, but usually much longer	**85**
84.	Gaster as long as or slightly longer than mesosoma; hosts: *Cassida* spp. (Coleoptera: Chrysomelidae)	***T. clito* (Walker)**
-	Gaster as long as head+mesosoma or slightly longer; hosts: Coccinellidae spp. (Coleoptera)	***T. epilachnae* (Giard)**
85.	Distance between submedian grooves 1.7–1.9× the distance between submedian and sublateral grooves (Figs 39b, 45b)	**86**
-	Distance between submedian grooves 1.0–1.4× the distance between submedian and sublateral grooves	**87**
86.	Ratio POL/OOL = 2.3	***T. bledius* sp. n.**
-	Ratio POL/OOL = 1.7	***T. ennis* sp. n.**
87.	Scape 0.9× as long as length of eye	**88**
-	Scape 1.0–1.1× as long as length of eye	**90**
88.	Antenna (Fig. 40a) with F1 1.8× as long as wide, widths F1/pedicel = 1.1; mesoscutellum with enclosed space between submedian grooves 3.1× as long as wide (Fig. 40b)	***T. calcarius* sp. n.**
-	Antenna with F1 1.3–1.5× as long as wide, widths F1/pedicel = 1.3–1.4; mesoscutellum with enclosed space between submedian grooves 2.6–2.7× as long as wide	**89**
89.	Mouth opening 1.1× malar space	***T. lixalius* sp. n.**
-	Mouth opening 0.9× malar space	***T. pixius* sp. n.**
90.	Antenna with F3 1.3–1.4× as long as wide	**91**
-	Antenna with F3 1.7–1.8× as long as wide	**93**
91.	Malar space 1.1× height of eye; mesoscutellum with enclosed space between submedian grooves 2.8× as long as wide and distance between submedian grooves 1.0× the distance between submedian and sublateral grooves (Fig. 46b)	***T. fenrisi* sp. n.**
-	Malar space 0.7–0.8× height of eye; mesoscutellum with enclosed space between submedian grooves 2.3–2.4× as long as wide and distance between submedian grooves 1.4× the distance between submedian and sublateral grooves	**92**
92.	Antenna (Fig. 48) longer, F2 1.7× and clava 4.2× as long as wide; POL 1.4× OOL	***T. lazius* sp. n.**
-	Antenna (Fig. 47a) shorter, F2 1.4× and clava 3.7× as long as wide; POL 1.7× OOL	***T. ladrus* sp. n.**
93.	Antenna with clava 4.2× as long as wide (Fig. 41a)	***T. clisius* sp. n.**
-	Antenna with clava 3.8× as long as wide (Fig. 50a)	***T. lycus* sp. n.**

**Table 3.** Key to males of European species of *Tetrastichus*.

1.	Frons with a weak median longitudinal carina reaching from between the antennal toruli to not more than half way towards the median ocellus, above this, there is a flat oblong to sublinear scrobal area, defined laterally by sutures which tend to converge ventrally to join the median carina (Fig. 3a); hind coxae strongly reticulate to rugulose on the externo-dorsal surface (Fig. 3c)	**2**
-	Frons with a more or less distinct median longitudinal carina extending from between the toruli to near the median ocellus, the sutures which define the scrobal area laterally tend to diverge ventrally, away from the median carina (Fig. 3b); hind coxae finely reticulate on the externo-dorsal surface (Fig. 3d)	**40**
2.	Antennae with each funicular segment with an externo-dorsal, sub-basal compact whorl of long setae, setae attached in a single tight row (Fig. 2d)	**3**
-	Antennae with funiculars without compact sub-basal whorls of long setae, i.e. with scattered setae throughout (Fig. 2e)	**29**
3.	Mouth 2.0–2.2× malar space and mandibles large (as in Fig. 3a), with outer tooth falcate and very acute, widely separated from the two small inner teeth which are subacute	**4**
-	Mouth at most 1.9× malar space, if 2.0× (some specimens of *T. coeruleus*, then mandibles smaller than in alternate); mandibles relatively smaller, middle tooth usually subobtuse, inner tooth obtuse or almost truncate	**5**
4.	Body weakly metallic and dull (as in Fig. 66)	***T. halidayi* (Graham)**
-	Body strongly metallic bluish-green (as in Figs 23, 34a, b)	***T. cyprus & T. marcusgrahami* spp.n.** (the males of these species are unknown, this is a tentative placement)
5.	Antennal scape with 10–12 very short setae on its front edge (Fig. 91d)	***T. tachos* (Walker)**
-	Antennal scape with fewer (usually 4–6) moderately long to very long setae on its front edge	**6**
6.	Scape with ventral plaque only 0.5–0.7× length of scape (Figs 73d, 93c), scape at least slightly longer than an eye and reaching well above the vertex; length of setae on front edge of scape about equal to width of scape; pedicel+flagellum 2x width of mesosoma; head with temples rather distinct, about 0.15× length of eye in dorsal view; head only 2.1–2.25× as broad as long in dorsal view	**7**
-	Antenna either with ventral plaque of scape relatively longer *or*, if short (0.5× length of scape), then scape not longer than an eye *or* pedicel+flagellum at most 1.9× width of mesoscutum; setae on front edge of scape often shorter; head usually with temples very short or extremely short, rarely 0.15× length of eyes; the head itself usually more transverse	**8**
7.	Antenna (Fig. 73d) with whorled setae of funiculars not reaching the tip of the segment attached to; length of longest setae on front edge of scape tending to be slightly greater than width of scape	***T. legionarius* Giraud**
-	Antenna (Fig. 93c) with whorled setae of funiculars reaching level with the tip of the segment attached to or slightly beyond tip; length of longest setae on front edge of scape equal to width of scape	***T. temporalis* (Graham)**
8.	Mouth opening 1.5–2.0× malar space	**9**
-	Mouth opening at most 1.4× malar space, usually distinctly less than this	**12**
9.	Antenna (Fig. 20f) with length of whorled setae on funiculars about 0.5–0.7× the length of the segment attached to; body bright metallic blue to bluish-purple (as in Fig. 20a, b, c)	***T. coeruleus* (Nees)**
-	Antenna with length of whorled setae on funiculars at least 1.2× the length of the segment attached to; body not so bright metallic	**10**
10.	Length of whorled setae on funiculars at least 1.4× the length of the segment attached to (Fig. 86c); F4 2.0× as long as wide (Fig. 86c)	***T. polyporinus* Askew**
-	Length of whorled setae on funiculars about 1.0–1.2× the length of the segment attached to; F4 1.6–1.8× as long as wide	**11**
11.	Antenna (Figs 70c, 71e) longer, 1.8–1.9× as long as width of mesoscutum and F1 1.8–1.9× as long as wide	***T. julis* (Walker) & *T. inscitus* sp. n.** (males are not possible to separate by morphology)
-	Antenna (Fig. 88f) shorter, 1.4× as long as width of mesoscutum and F1 1.5× as long as wide	***T. setifer* Thomson**
12.	Setae on vertex long (as in Fig. 5c), 0.8–1.0× OD; eyes in most species with relatively long and conspicuous pubescence (as in Fig. 7d), length of the setae 0.3–0.6× OD; body black with very weak bronze or bluish tints (Figs 4–14)	**13**
-	Setae on vertex short, 0.3× OD; eyes with short or very short pubescence, length of the setae at most 0.25× OD; body sometimes with brighter metallic tints	**20**
13.	Antenna with pedicel 1.25× as long as F1, the latter only 1.5× as long as broad	***T. solvae* Graham**
-	Antennae with pedicel about as long as or shorter than F1; F1 1.4–2.0× as long as broad	**14**
14.	Submedian grooves of mesoscutellum equidistant from each other and from sublateral grooves (as in Fig. 6a)	***T. brachyopae* Graham**
-	Submedian grooves of mesoscutellum nearer to sublateral grooves than to each other	**15**
15.	Distance between submedian grooves 2.0× the distance between submedian and sublateral grooves (as in Fig. 14b)	***T. tartus* sp. n.** (the male of this species is unknown, but is tentatively keyed out here)
-	Distance between submedian grooves 1.2–1.7× the distance between submedian and sublateral grooves	**16**
16.	Antenna (Fig. 11a) with pedicel+flagellum about 1.25× width of mesoscutum; F2–F4 each, at most, 1.6× as long as broad; eyes with very long setae, length about 0.8× OD	***T. murcia* (Walker)**
-	Antenna with pedicel+flagellum 1.48–1.6× width of mesoscutum; F2–F4 each, at least, twice as long as broad; eyes with length of setae 0.30–0.55× OD	**17**
17.	Clava (incl.spicule) 3.3–4.5× as long as wide	**18**
-	Clava (incl. spicule) 5.3–6× as long as wide	**19**
18.	Clava (incl.spicule) 3.3× as long as wide (Fig. 7c); setae on eyes longer (as in Fig. 7d), 0.5× OD	***T. dasyops* Graham**
-	Clava (incl.spicule) 4.3–4.5× as long as wide (Fig. 5d); setae on eyes shorter (as in Fig. 5c), 0.3× OD	***T. atratulus* (Nees)**
19.	Antenna with F4 2.3× and scape 2.9× as long as wide (Fig. 13c)	***T. tacitus* sp. n.**
-	Antenna with F4 2.7× and scape 3.2× as long as wide (Fig. 10c)	***T. mixtus* sp. n.**
20.	Eyes small (as in Fig. 87c), 1.0× as long as malar space; submedian grooves on mesoscutellum converging towards posterior part (as in Fig. 87b)	***T. scardiae* sp. n.**
-	Eyes longer than malar space (1.1–1.2×); submedian grooves on mesoscutellum ± parallel	**21**
21.	Antenna with length of longest seta on front edge of scape 1.1–1.5× width of scape; C1and C2 each at most 1.7× as long as broad	**22**
-	Antenna with length of longest seta on front edge of scape less than or hardly as long as width of scape; C1and C2 often relatively longer	**23**
22.	Mouth about 1.4× malar space; ventral plaque of antennal scape (Fig. 71e) about 0.7× length of scape	***T. julis* (Walker)**
-	Mouth 1.0–1.1× malar space; ventral plaque of antennal scape (Fig. 77f) 0.50–0.55× length of scape	***T. lyridice* (Walker)**
23.	Eyes small, separated by 1.6–1.85× their length; antennal scape as long as or somewhat longer than an eye, reaching above level of vertex	**24**
-	Eyes larger, separated by 1.35–1.5× their length; antennal scape either not quite as long as an eye or, if as long, then not reaching above level of vertex	**25**
24.	Antenna with scape about 1.25× length of eye; the whorled dark setae of each funicular segment reach to level of tip of the segment attached to; scape about 2.8× as long as broad; eyes separated by 1.85× their length	***T. perkinsorum* Graham**
-	Antenna (Fig. 69e) with scape 1.14× length to an eye; the whorled setae of each funicular segment reach slightly beyond the tip of the segment attached to; scape 2.4–2.5× as long as broad; eyes separated by 1.6–1.65× their length	***T. ilithyia* (Walker)**
25.	Antenna with whorled setae of funiculars at most just reaching the tip of funicular attached to, scape 2.7–3.2× as long as broad	**26**
-	Antenna with whorled setae of funiculars reaching slightly beyond the tip of funicular attached to, scape 2.4–2.8× as long as broad	**27**
26.	Antenna (Fig. 81d) with F1 not or hardly shorter than F2; clava with short spicule	***T. miser* (Nees)**
-	Antenna with F1 distinctly shorter than F2; clava with long spicule	***T. sinope* (Walker)**
27.	Flagellomeres shorter (Fig. 55c), F1 1.5×, F2 1.9×, F3 and F4 each 2.0× as long as wide	***T. agonus* sp. n.**
-	Flagellomeres longer, F1 2.1–2.5×, F2 2.4–2.9×, F3 2.7–3.0×, F4 2.5–2.6× as long as wide	**28**
28.	Body length 1.8–2.1 mm; antenna (Fig. 56c) with length of longest seta on front edge of scape nearly equal in length to width of scape	***T. agrilocidus* Graham**
-	Body length 1.2–1.8 mm; antenna (Fig. 67c) with length of longest seta on front edge of scape somewhat shorter than width of scape	***T. heeringi* Delucchi**
29.	Mouth opening 1.4–1.6× malar space	***T. crioceridis* Graham**
-	Mouth opening 1.0–1.2× malar space	**30**
30.	Scape 0.9–1.0× length of eye; scape with ventral plaque 0.6× length of scape; body dull metallic (blue, coppery or green) to non-metallic	**31**
-	Scape 1.0–1.2× length of eye; scape with ventral plaque 0.7–0.8× length of scape; body bright metallic green or golden-green	**33**
31.	Antenna (Fig. 8c) with pedicel+flagellum 2.0× as long as width of mesoscutum, F1 to F4 each 2.3–2.4× as long as wide	***T. intruitus* sp. n.**
-	Antenna (Figs 29c, 75d) with pedicel+flagellum 1.6–1.7× as long as width of mesoscutum and F1 1.5–1.6×, F2 1.6–1.9×, F3 and F4 each 1.8–1.9× as long as wide	**32**
32.	Antenna (Fig. 75d) with scape 2.7× and clava 4.6× as long as wide; head 2.4× as wide as long in dorsal view and 1.4× in frontal view	***T. leocrates* (Walker)**
-	Antenna (Fig. 29c) with scape 3.2× and clava 3.3× as long as wide; head 2.2× as wide as long in dorsal view and 1.2× in frontal view	***T. iasi* sp. n.**
33.	Antennal clava (incl. spicule) 4.9–5.6× as long as wide	**34**
-	Antennal clava (incl. spicule) 3.3–4.4× as long as wide	**36**
34.	Antennal scape 2.2× as long as wide (Fig. 22d)	***T. cumulus* sp. n.**
-	Antennal scape 2.7–2.8× as long as wide	**35**
35.	Antennal clava (incl. spicule) 5.6× and F1 2.5× as long as wide	***T. brevicalcar* Graham**
-	Antennal clava (incl. spicule) 4.9× and F1 1.9× as long as wide (Fig. 32c)	***T. incanus* sp. n.**
36.	Antennal scape 2.4–2.6× as long as wide	**37**
-	Antennal scape 3.0× as long as wide	**38**
37.	Antenna (Fig. 15c) with clava (incl. spicule) 4.4×, F1 1.7×, F2–F4 each 2.0–2.2×, scape 2.4× as long as wide	***T. argei* sp. n.**
-	Antenna (Fig. 28c) with clava (incl. spicule) 4.0×, F1 1.4×, F2 1.6×, F3 1.7×, F4 1.8×, scape 2.6× as long as wide	***T. hylotomarum* (Bouché)**
38.	Antenna (Fig. 33c) with F1 1.2× as long as wide; eye length/malar space = 1.25	***T. johnnoyesi* sp. n.**
-	Antenna with F1 1.6× as long as wide; eye length/malar space = 1.10–1.19	**39**
39.	Antenna (Fig. 36c) with F2 and F4 each 2.4–2.5× as long as wide	***T. sti* sp. n.**
-	Antenna (Fig. 37c) with F2 2.0× and F4 2.2× as long as wide	***T. suecus* sp. n.**
40.	Scape narrow at apex and with short ventral plaque (Figs 38c, 51c), 0.3–0.4× length of scape	**41**
-	Scape wide at apex and with long ventral plaque (e.g. Fig. 42e), 0.7–0.9× length of scape	**42**
41.	Antenna (Fig. 38c) with F1 1.7× as long as pedicel; hind coxa with a strong and sharp carina along posterior margin (as in Fig. 21c)	***T. argutus* sp. n.**
-	Antenna (Fig. 51c) with F1 0.9× as long as pedicel; hind coxa without a strong and sharp carina along posterior margin	***T. nymphae* sp. n.**
42.	Whorled setae on F1 short, 1.1× as long as length of F1	**43**
-	Whorled setae on F1 1.4–1.8× as long as length of F1	**44**
43.	Antenna (Fig. 42e): scape with ventral plaque 0.65–0.75× length of scape; pedicel 1.6–1.7× as long as broad; hosts: *Cassida* spp. (Coleoptera: Chrysomelidae)	***T. clito* (Walker)**
-	Scape with ventral plaque 0.5–0.6× length of scape; pedicel 1.8–1.9× as long as broad; hosts: Coccinellidae spp. (Coleoptera)	***T. epilachnae* (Giard)**
44.	Scape 1.1× as long as an eye and 2.1× as long as wide (Fig. 41c, d)	***T. clisius* sp. n.**
-	Scape 1.3–1.4× as long as an eye and 2.4–2.6× as long as wide	
45.	Antenna (Fig. 49c) with pedicel 1.0× as long as length of F1, scape 2.6× as long as wide	***T. lixalius* sp. n.**
-	Antenna (Fig. 39c) with pedicel 1.2× as long as length of F1, scape 2.4× as long as wide	***T. bledius* sp. n.**
